# Cross-dehydrogenative coupling enables enantioselective access to CF_3_-substituted all-carbon quaternary stereocenters†

**DOI:** 10.1039/c9sc05894j

**Published:** 2020-01-29

**Authors:** Xiaoguang Pan, Zehua Wang, Linglong Kan, Ying Mao, Yasheng Zhu, Lei Liu

**Affiliations:** School of Pharmaceutical Sciences, Shandong University Jinan 250012 China leiliu@sdu.edu.cn; School of Chemistry and Chemical Engineering, Shandong University Jinan 250100 China

## Abstract

A cross-dehydrogenative coupling strategy for enantioselective access to acyclic CF_3_-substituted all-carbon quaternary stereocenters has been established. By using catalytic DDQ with MnO_2_ as an inexpensive terminal oxidant, asymmetric cross coupling of racemic δ-CF_3_-substituted phenols with indoles proceeded smoothly, providing CF_3_-bearing all-carbon quaternary stereocenters with excellent chemo- and enantioselectivities. The generality of the strategy is further demonstrated by efficient construction of all-carbon quaternary stereocenters bearing other polyfluoroalkyl and perfluoroalkyl groups such as CF_2_Cl, C_2_F_5_, and C_3_F_7_.

## Abstract 2

A cross-dehydrogenative coupling (CDC) strategy for enantioselective access to acyclic CF_3_-substituted all-carbon quaternary stereocenters has been established.

## Graphical abstract

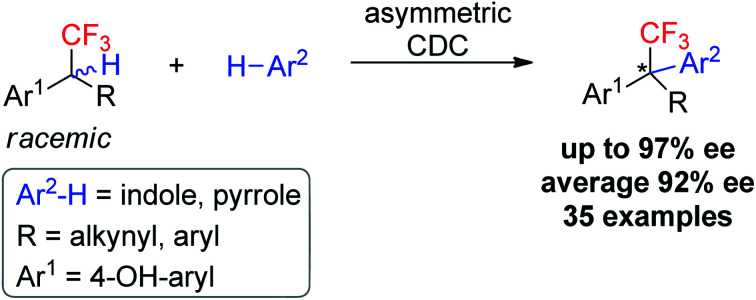

## Introduction

**Scheme 1:**
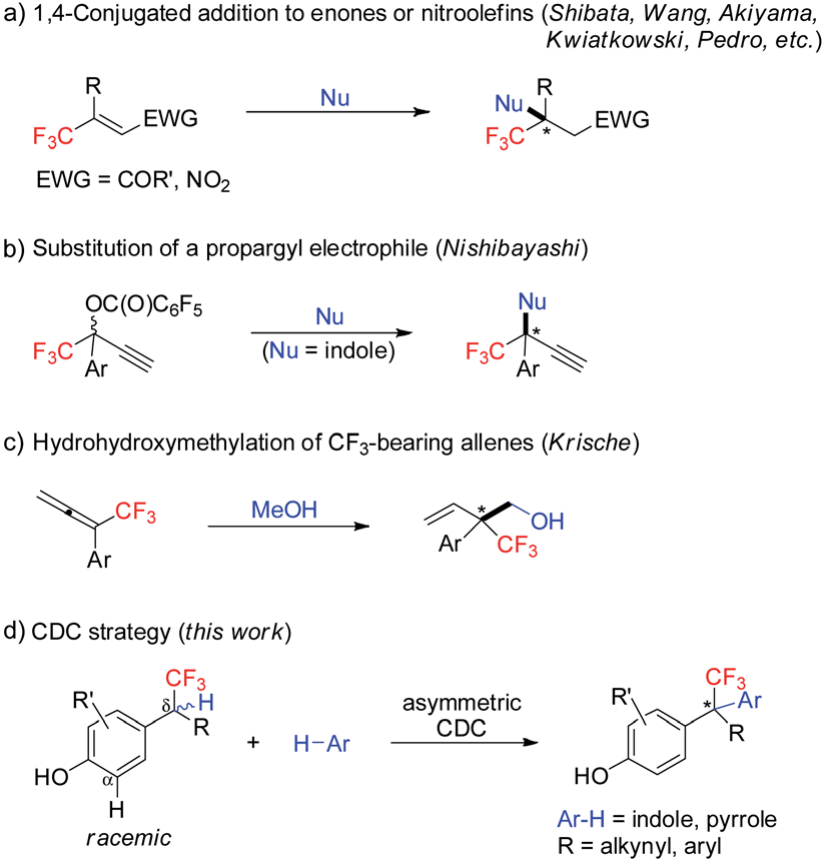
Overview of enantioselective access to acyclic CF_3_-substituted all-carbon quaternary stereocenters.

Enantiopure molecules bearing a trifluoromethyl-containing stereogenic center often possess desirable properties.^1^ Therefore, practical and robust approaches for their enantioselective synthesis are highly attractive.^2^ On the other hand, the catalytic enantioselective construction of all-carbon quaternary stereocenters remains one of the great challenges in organic chemistry.^3^ In this context, enantioselective construction of acyclic CF_3_-substituted all-carbon quaternary centers is particularly daunting.^4–7^ Since the seminal work by Shibata, current strategies are restricted to 1,4-conjugate addition to β,β-disubstituted CF_3_-enones or nitroolefins (Scheme 1a),^4^ and two isolated methods including substitution of a propargyl electrophile (Scheme 1b),^5^ and hydrohydroxymethylation of CF_3_-bearing allenes (Scheme 1c).^6^ All of these methods rely on reactive functional groups, and extra steps are usually involved for their incorporation. Developing a strategically different C–H functionalization approach for enantioselective construction of CF_3_-bearing all-carbon quaternary centers is highly desirable.

Enantioselective cross-dehydrogenative coupling (CDC) of two easily accessible C–H substrates represents a straightforward and economical approach in organic synthesis.^8^ Existing studies predominantly focused on C–H bonds adjacent to a heteroatom.^9,10^ But surprisingly, asymmetric CDC involving functionalization of acyclic benzylic C–H bonds has rarely been explored.^11,12^ Elegant works from the groups of Cozzi and Gong reported enantioselective CDC of 3-arylmethylindoles with aldehydes^11*a*^ and malonates.^11*b*^ In addition, CDC technology for enantioselective construction of all-carbon quaternary stereocenters has remained elusive.^13^ Recently, our group disclosed a chiral imidodiphosphoric acid catalyzed asymmetric CDC of 2,2-diarylacetonitriles with (hetero)arenes, furnishing triarylmethanes bearing all-carbon quaternary stereocenters with excellent enantioselectivity.^13*c*^ Given the importance of optically active hetero-di- and hetero-triarylmethanes in chemistry, biology, material science, and medicine,^14^ we decided to explore the asymmetric CDC of racemic *p*-hydroxybenzyl CF_3_ moieties with heteroarenes for construction of these motifs containing CF_3_-substituted all-carbon quaternary stereocenters (Scheme 1d).

Three main challenges might obstruct the reaction design. First, regioselective oxidation of C_δ_–H bond adjacent to strong electron-withdrawing CF_3_ group is difficult to achieve, which might be accompanied by competitive oxidation of C_α_–H bond to 1,2-benzoquinones.^15^ Second, even if the expected oxidation proceeded smoothly, the CDC reaction might still be precluded by the potential incompatibility of electron-rich heteroarenes with strongly oxidative conditions. Third, the oxidized intermediate is expected to be highly unstable δ-CF_3_-substituted *para*-quinone methide (*p*-QM).^16^ Effective and enantioselective addition to highly congested CF_3_-substituted carbon of reactive *p*-QM intermediate under strongly oxidative conditions is substantially challenging.^17–19^

## Results and discussion

Initially, chiral phosphoric acid catalyzed asymmetric CDC of *p*-hydroxybenzyl CF_3_**1a** with indole **2a** was selected as a model reaction for optimization (Table 1).^20^ To explore a suitable oxidation system, the reaction involving an initial oxidation of **1a** followed by **3a** catalyzed nucleophilic addition of **2a** was conducted in a two-step, one-pot manner. As expected, efficient oxidation of **1a** proved to be challenging. Common reagents for phenol oxidation, such as K_3_Fe(CN)_6_, (NH_4_)_2_S_2_O_8_, Ag_2_O, PhI(OAc)_2_, and MnO_2_, proved to be futile (entries 1 and 2, Table 1). Reaction with DDQ provided expected **4a** in 10% yield with 26% ee, though the majority of **1a** (83%) was recovered (entry 3, Table 1). Increasing the loading of DDQ did not improve oxidation conversion (entry 4, Table 1). We envisioned that oxidation of **1a** with DDQ might be a reversible process, and adopting a DDQ-catalyzed oxidation system might be beneficial for breaking the equilibrium and driving the oxidation process. A screen of terminal oxidants towards metal oxides revealed that use of DDQ (25 mol%) as catalyst and MnO_2_ as stoichiometric oxidant furnished a complete and clean oxidation, and the CDC process afforded **4a** in 70% yield with 55% ee (entries 5–7, Table 1).^21^ Reversal of the procedure by adding all the components prior to oxidation gave an inferior result (entry 8, Table 1). Optimization of chiral phosphoric acid catalysts identified **3c** to be optimal (entries 7 and 9–12, Table 1). K_2_CO_3_ as a basic additive proved to be beneficial for improving the enantiocontrol (entry 13, Table 1). Increasing the loading of **2a** was beneficial to the reaction by slowing down the nucleophilic addition process, and **4a** was isolated in 86% yield with 93% ee (entry 14, Table 1).

**Table 1:** Optimization of the reaction conditions^a^

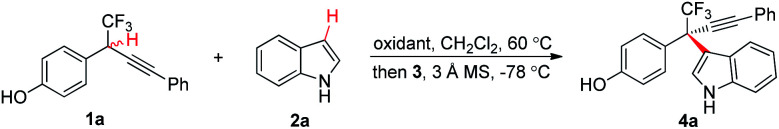				
Entry	Oxidant	Catalyst	Yield^b^ (%)	ee^c^ (%)
1^d^	Oxidant	**3a**	<5	n.d.
2	MnO_2_	**3a**	<5	n.d.
3	DDQ	**3a**	10	26
4^e^	DDQ	**3a**	12	21
5^f^	DDQ/FeCl_3_	**3a**	<5	n.d.
6^f^	DDQ/Mn(OAc)_3_	**3a**	45	39
7^f^	DDQ/MnO_2_	**3a**	70	55
8^f^^,^^g^	DDQ/MnO_2_	**3a**	32	9
9^f^	DDQ/MnO_2_	**3b**	<5	n.d.
10^f^	DDQ/MnO_2_	**3c**	78	85
11^f^	DDQ/MnO_2_	**3d**	<5	n.d.
12^f^	DDQ/MnO_2_	**3e**	36	41
13^f^^,^^h^	DDQ/MnO_2_	**3c**	83	88
14^f^^,^^h^^,^^i^	DDQ/MnO_2_	**3c**	86	93
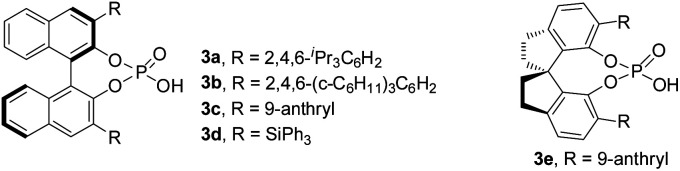				

a Reaction conditions: **1a** (0.1 mmol) and oxidant (0.12 mmol) in CH_2_Cl_2_ at 60 °C for 8 h, followed by **2a** (0.1 mmol), **3** (5 mol%), 3 Å molecular sieves (20 mg) at −78 °C for 1 h.

b Yield of isolated product.

c Determined by chiral HPLC analysis.

d K_3_Fe(CN)_6_, (NH_4_)_2_S_2_O_8_, Ag_2_O, and PhI(OAc)_2_ as oxidant.

e 2.0 equiv. of DDQ used.

f 25 mol% DDQ with 3.0 equiv. of terminal oxidant.

g **2a** and **3a** added before oxidation.

h 2.0 equiv. of K_2_CO_3_ as additive.

i **2a** (0.3 mmol) used. n.d. = not determined.

**Scheme 2:**
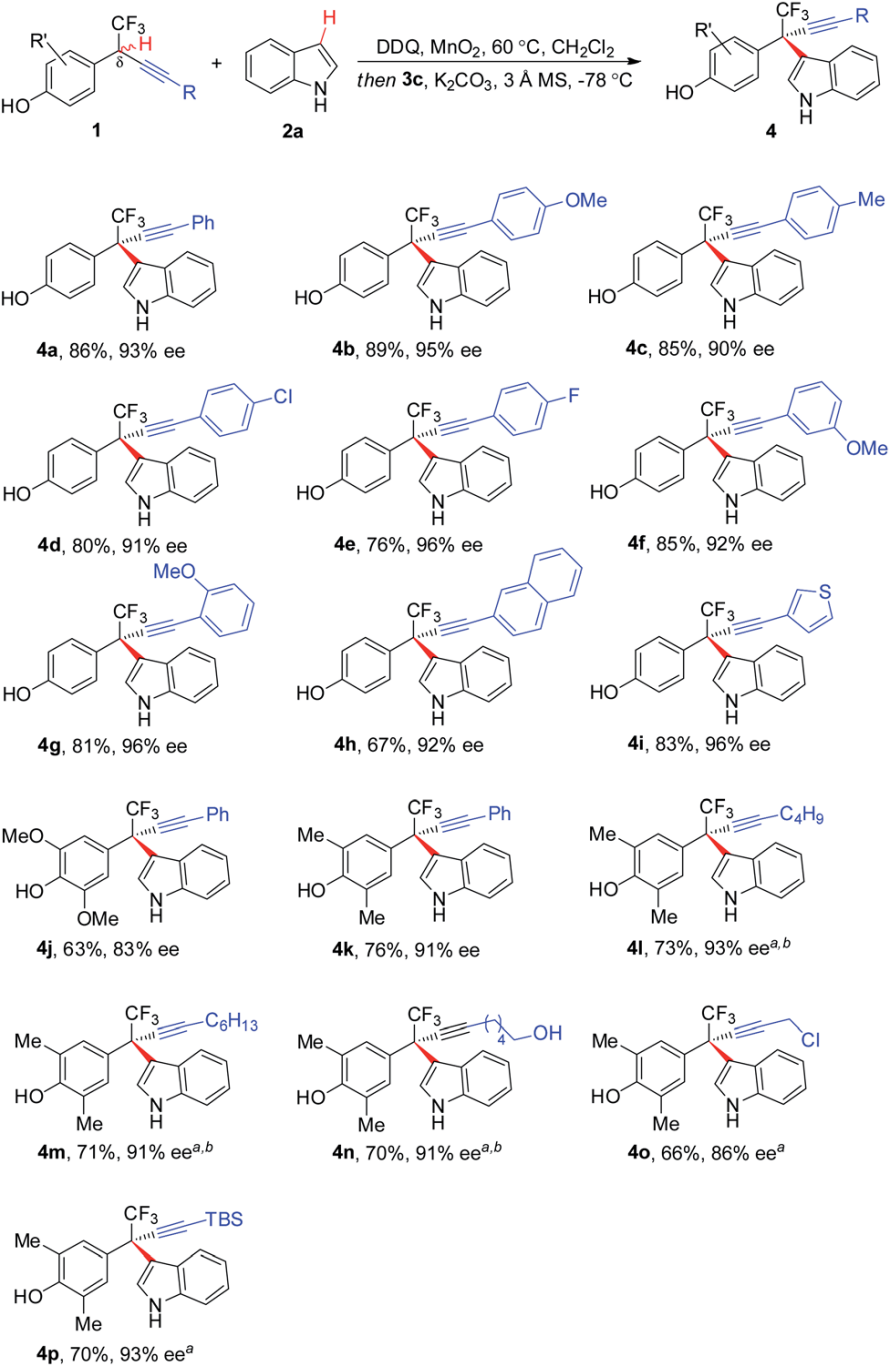
Scope of δ-alkynyl-substituted substrates for CF_3_-containing hetero-diarylmethanes. ^*a*^Reaction with **3b** (5 mol%) and **2a** (1.1 equiv.) without K_2_CO_3_ additive. ^*b*^Asymmetric nucleophilic addition of **2a** was performed at 0 °C.

The scope of asymmetric CDC of δ-CF_3_-δ-alkynyl substituted **1** with **2a** was investigated (Scheme 2). In general, substrates bearing a wide range of electronically varied aryl acetylenes with different substitution patterns were tolerated, affording respective hetero-diarylmethanes **4a–4g**, **4j**, and **4k** in good yields with excellent ee. Polyarene naphthalene substituted acetylene **1h** and thiophene substituted **1i** proved to be competent coupling partners. δ-Alkyl acetylene-containing **1l–1p** were also compatible with the asymmetric CDC process, furnishing corresponding **4l–4p** in 66–73% yields with up to 93% ee. The process exhibited a good functional group tolerance, with common functionalities like halides (**4d** and **4e**), primary alcohol (**4n**), propargyl chloride (**4o**), and silyl group (**4p**) well tolerated for further manipulation.

**Scheme 3:**
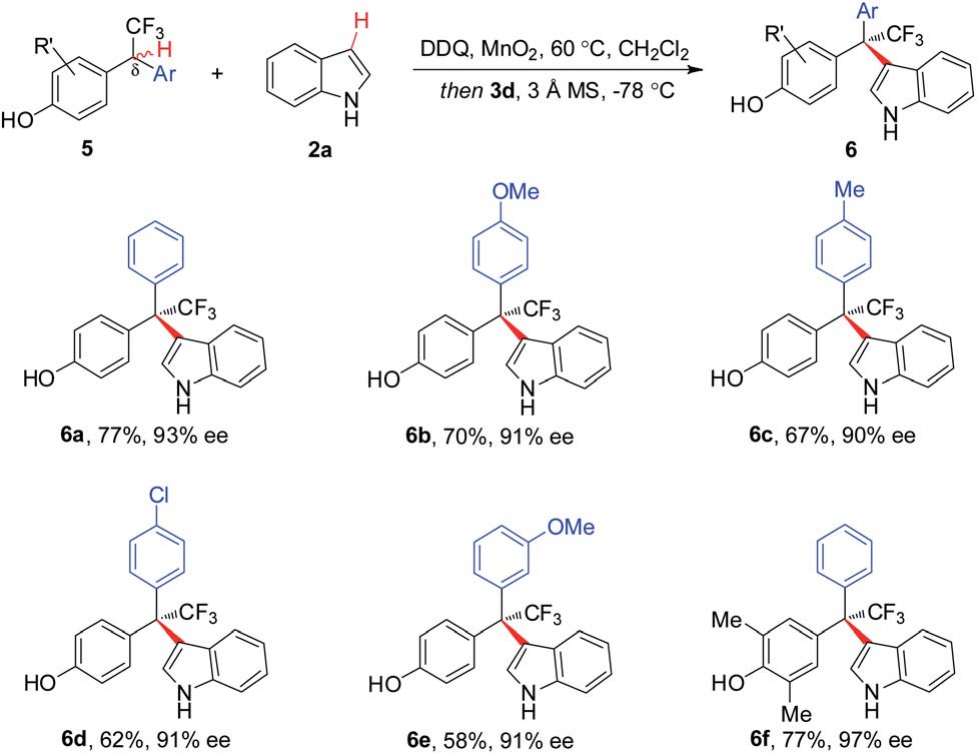
Scope of δ-aryl-substituted substrates for CF_3_-containing hetero-triarylmethanes. ^a^Reaction with **2a** (1.1 equiv.).

Asymmetric CDC of a variety of δ-aryl substituted **5** with **2a** proceeded smoothly, affording respective CF_3_-bearing hetero-triarylmethanes **6a–6f** with 90–97% ee (Scheme 3). To our knowledge, this is the first example of direct and asymmetric construction of CF_3_-substituted tri-arylmethanes.^5^

**Scheme 4:**
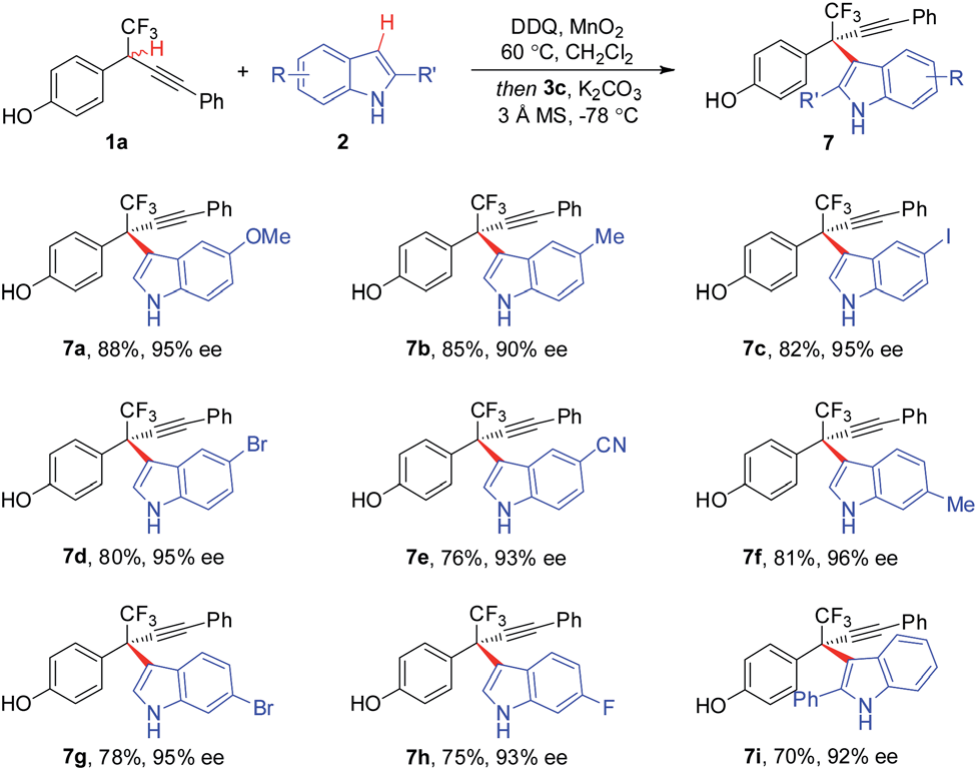
Scope of the indole components.

**Scheme 5:**
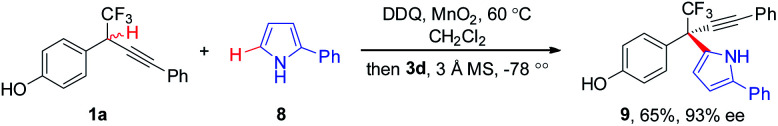
Asymmetric CDC with 2-substituted pyrrole. ^a^1.0 equiv. of **8** used.

The substituent effect of indoles was next evaluated (Scheme 4). A broad range of indoles **2** bearing either electron-donating or -withdrawing groups at different positions (C_4_, C_5_, C_6_, and C_7_) on aryl rings participated in the CDC process, furnishing corresponding **7a–7h** in 75–88% yields with 90–96% ee. Additionally, C_2_-substituted indoles proved to be competent components, as demonstrated by the generation of **7i** in 70% yield with 92% ee. Besides indole moieties, 2-substituted pyrroles were also identified to be suitable coupling partners in asymmetric CDC reaction, as illustrated by the formation of **9** in 65% yield with 93% ee (Scheme 5). While the scope of 2-substituted pyrroles was not exclusively explored, the result afforded a proof-of-concept for the modularity of the method for asymmetric preparation of diversely functionalized hetero-diarylmethanes bearing CF_3_-substituted all-carbon quaternary stereocenters.

**Scheme 6:**
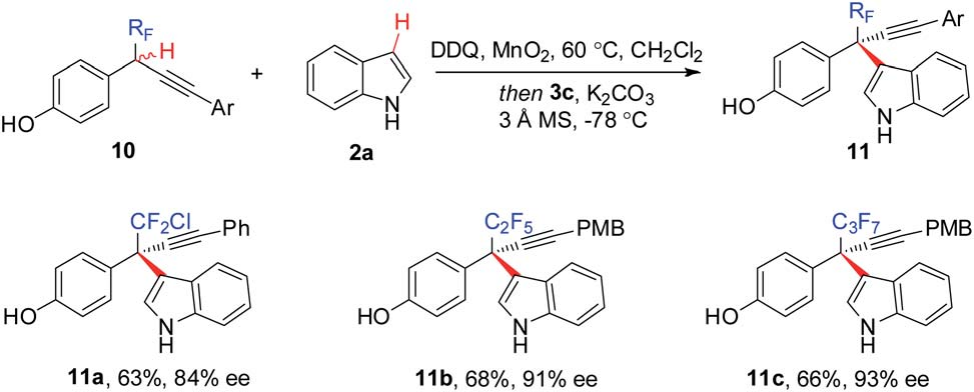
Enantioselective access to other polyfluoroalkyl-bearing all-carbon quaternary stereocenters.

The generality of the CDC approach is further demonstrated by enantioselective construction of all-carbon quaternary stereocenters bearing other polyfluoroalkyl or perfluoroalkyl groups, such as CF_2_Cl (**11a**), C_2_F_5_ (**11b**), and C_3_F_7_ (**11c**) (Scheme 6).

**Scheme 7:**
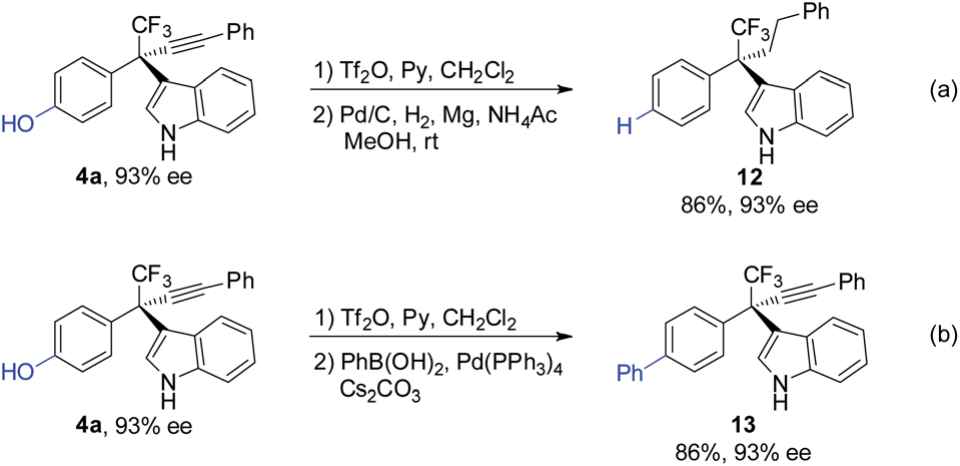
Representative product transformations.

The synthetic utilities of the method were next examined (Scheme 7). The phenolic hydroxyl group in **4a** was removed through triflation followed by hydrogenation affording **12** in 86% yield (Scheme 7a). Phenol **4a** can also undergo triflation followed by palladium-catalyzed cross-coupling reaction, furnishing biaryl **13** efficiently (Scheme 7b). Notably, the ee values of the products remain highly reserved in these processes.

**Scheme 8:**
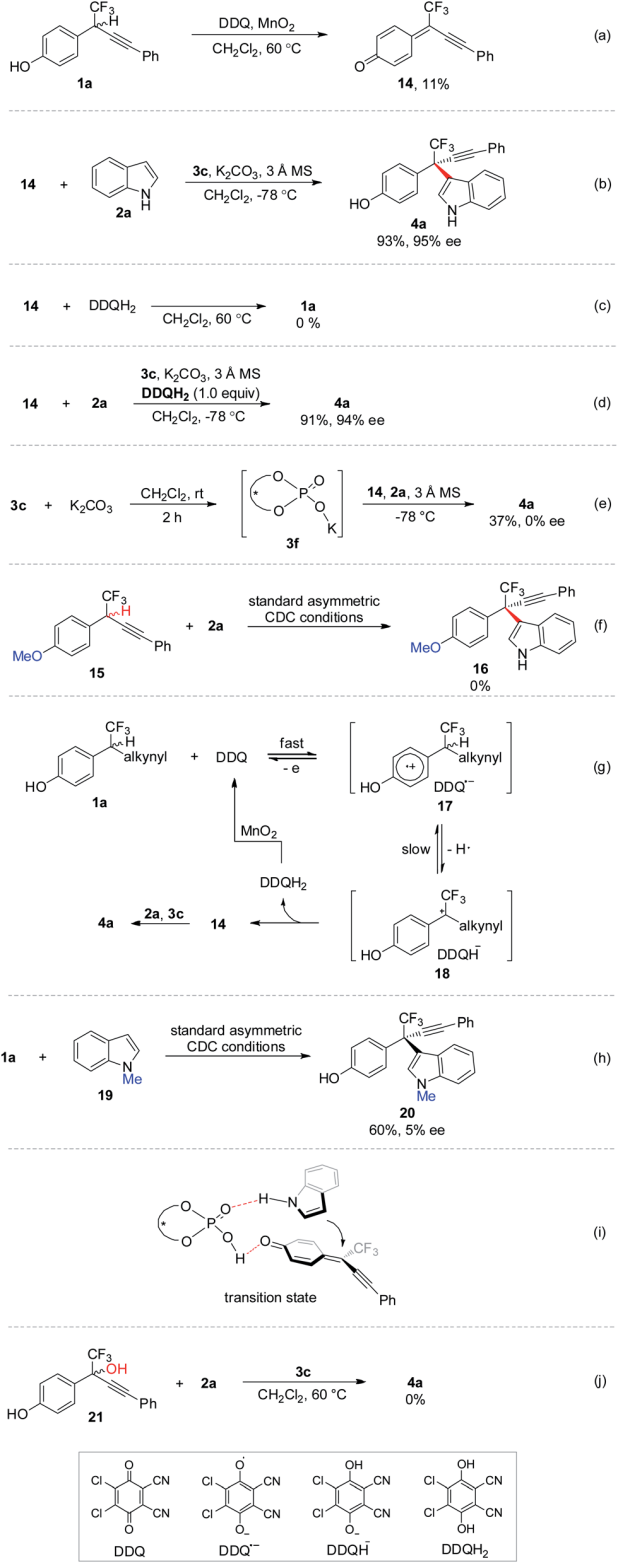
Control experiments and the proposed mechanism.

Control experiments were performed to gain further insights into the mechanism (Scheme 8). Upon completion of oxidation of **1a**, δ-CF_3_-δ-alkynyl-substituted *p*-QM **14** was isolated in 11% yield (Scheme 8a). The low yield might be ascribed to the poor stability of δ-CF_3_-substituted *p*-QM compound. Subjecting **14** to standard CDC conditions in the absence of oxidation elements furnished **4a** with comparable yield and ee to those observed in the one-pot process, thus indicating the intermediacy of *p*-QM **14** (Scheme 8b). No invertible reaction was observed for **14** and 2,3-dichloro-4,5-dicyanohydroquinone (DDQH_2_), the reduction product of DDQ (Scheme 8c). The asymmetric 1,6-conjugate addition to **14** was not influenced by introducing stoichiometric amount of acidic DDQH_2_ (Scheme 8d). K_2_CO_3_ was found to be beneficial for improving the enantiocontrol (entry 13, Table 1). Accordingly, chiral potassium-organophosphate **3f** was prepared *in situ* for real catalyst identification (Scheme 8e). No enantioselective catalytic reactivity was observed for **3f**, implying that **3c** but not **3f** should be the real catalyst, and the hydroxyl group in chiral phosphoric acid is requisite. No reaction was observed for *p*-methoxybenzyl CF_3_**15**, indicating the significance of the hydroxyl moiety in the *in situ* formation of *p*-QM intermediate (Scheme 8f). According to the above experiments, a plausible mechanism was recommended (Scheme 8g). Racemic *p*-hydroxybenzyl CF_3_ moiety **1a** might be oxidized by catalytic amount of DDQ, giving *p*-QM **14** together with the generation of DDQH_2_. Stoichiometric MnO_2_ as terminal oxidant proved to be crucial to the complete oxidation of **1a** to **14** by converting DDQH_2_ to DDQ for the catalytic cycle.^21^ Chiral phosphoric acid **3c** catalyzed asymmetric 1,6-conjugate addition of indole **2a** to **14** yielding expected **4a**.^22^ Asymmetric CDC of **1a** with *N*-methyl protected indole **19** provided inferior ee to unprotected **2a**, implying that the N–H moiety might act as a hydrogen bond donor (Scheme 8h). A plausible transition state was proposed in Scheme 8i, in which chiral phosphoric acid acts as a bifunctional role for activation of both coupling partners and remote stereocontrol by hydrogen bonding. *p*-QM intermediates can be generated *in situ* through chiral phosphoric acid catalyzed dehydration of *p*-hydroxybenzyl alcohols.^17^ Accordingly, δ-CF_3_-substituted *p*-hydroxybenzyl alcohol **21** was subjected to the CDC condition without oxidation elements (Scheme 8j). However, no reaction was observed even at an elevated temperature, thus demonstrating the uniqueness of the oxidation strategy in generating unstable δ-CF_3_-substituted *p*-QM intermediates.

## Conclusions

In summary, CDC strategy for enantioselective construction of CF_3_-substituted all-carbon quaternary stereocenters has been established for the first time. By using catalytic DDQ with MnO_2_ as an inexpensive terminal oxidant, asymmetric cross-coupling of racemic *p*-hydroxybenzyl CF_3_ moieties with indoles and pyrroles proceeded smoothly, providing acyclic CF_3_-bearing all-carbon quaternary centers with excellent chemo- and enantioselectivity. The generality of the strategy is further demonstrated by efficient formation of all-carbon quaternary centers bearing other polyfluoroalkyl and perfluoroalkyl groups such as CF_2_Cl, C_2_F_5_, and C_3_F_7_. We envisioned that the strategically different approach described here will provide an attractive platform for enantioselective access to all-carbon quaternary stereocenters bearing diverse perfluoroalkyl groups that are otherwise difficult to be prepared by existing methods.

## Conflicts of interest

There are no conflicts to declare.

## Supplementary Material

SC-011-C9SC05894J-s001
