# Supplementary material for: Cross-dehydrogenative coupling enables enantioselective access to CF3-substituted all-carbon quaternary stereocenters
Source: Chem Sci. 2020 Jan 29;11(9):2414–9. doi: 10.1039/c9sc05894j (PMC8157275; doi:10.1039/c9sc05894j)
Supplement: SC-011-C9SC05894J-s001 [file SC-011-C9SC05894J-s001.pdf]

Electronic Supplementary Material (ESI) for Chemical Science.
This journal is © The Royal Society of Chemistry 2020

Supporting Information

Cross-Dehydrogenative Coupling Enables Enantioselective Access to CF_3-Substituted All-Carbon Quaternary Stereocenters

Xiaoguang Pan, Zehua Wang, Linglong Kan, Ying Mao, Yasheng Zhu, and Lei Liu*

Table of contents

Table of contents S1

General information S2

Substrate preparation S3

General procedure S13

Absolute configuration determination S32

Synthetic application S37

Mechanistic studies S39

References S42

NMR spectra S43

HPLC spectra for ee determination S143

General information

Proton (^{1}H NMR) nuclear magnetic resonance spectra were recorded at 500 or 600 MHz respectively. Carbon (^{13}C NMR) nuclear magnetic resonance spectra were recorded at 126 or 151 MHz respectively. Fluorine (^{19}F NMR) nuclear magnetic resonance spectra were recorded at 471 MHz respectively. The chemical shifts are given in parts per million (ppm) on the delta (δ) scale. The solvent peak was used as a reference value, for ^{1}H NMR: $CDCl_3$ = 7.26 ppm, $(CD_3)_2SO$ = 2.50 ppm, CD_3OD = 3.31 ppm, C_6D_6 = 7.16 ppm; for ^{13}C NMR: $CDCl_3$ = 77.23 ppm, $(CD_3)_2SO$ = 39.51 ppm, CD_3OD = 49.0 ppm, C_6D_6 = 128.06 ppm. Analytical TLC was performed on precoated silica gel GF254 plates. Column chromatography was carried out on silica gel (200–300 mesh). HRMS were carried out on an Orbitrap analyzer. UV spectra were obtained with an Agilent 8453E UV-Visible spectroscopy system. CD spectra were obtained on a Chirascan spectropolarimeter. Optical rotations were measured using a 2.5mL cell with a 10 cm path length on Hanon P850 Automatic Polarimeter and concentrations (c) were reported in $g\times(100\ mL)^{-1}$. Enantiometric excesses were determined by HPLC using a Daicel Chiralpak and Chiralcel column with hexane/i-PrOH as the eluent on Dionex instrument.

Substrate Preparation

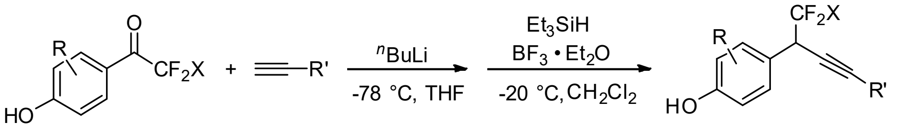

(I)

S1 **S2** **1** or **10**

X= F, Cl, CF_3, C_2F_5

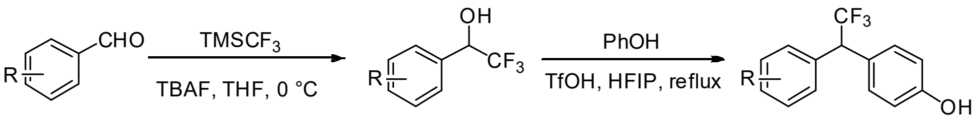

(II)

S3 **S4** **5**

Procedure I for preparation of substrate 1 or 10[1-2]

To a solution of terminal alkyne **S2** (6.3 mmol, 2.1 equiv) in anhydrous THF at -78 °C under N_2 was added ^{n}BuLi (6 mmol, 2.4 mL, 2.5 M in hexane, 2.0 equiv) dropwise. The reaction was stirred at the same temperature for 1 h. Then a solution of the corresponding ketone **S1** (3 mmol, 1.0 equiv) in anhydrous THF (5 mL) was added to the mixture dropwise. The reaction was stirred at -78 °C for 15 min and then it was warmed up to room temperature slowly and stirred overnight. Upon completion, the mixture was quenched dropwise by a saturated aqueous NH_4Cl solution (5 mL). The organic layer was extracted with ethyl acetate (3×15 mL) and the combined organic layers were washed with saturated aqueous NaCl solution, dried over anhydrous $MgSO_4$, filtered and removed under vacuum. Then the residue was dissolved in anhydrous CH_2Cl_2 (15 mL) at -20 °C and Et_3SiH (12 mmol, 4.0 equiv) and $BF_3 \cdot Et_2O$ (7.5 mmol, 2.5 equiv) were added to the solution. The reaction was stirred at the same temperature and monitored by TLC until the complete conversion. Then the reaction was quenched by saturated aqueous $NaHCO_3$ solution (10 mL). The organic layer was extracted with CH_2Cl_2 (3×15 mL) and the combined organic layers were washed with saturated aqueous NaCl solution, dried over anhydrous $MgSO_4$, filtered and removed under vacuum. The residue was purified by a column chromatography on silica gel using ethyl acetate/petroleum ether as eluent to give the desired product **1** or **10**.

Procedure II for preparation of substrate 5[3-4]

To a solution of aromatic aldehyde **S3** (2.0 mmol, 1.0 equiv) in anhydrous THF at 0 °C under N_2 was added $TMSCF_3$ (2.4 mmol, 1.2 equiv) and TBAF (2.4 mmol, 2.4 mL, 1.0 mol/L in THF, 1.2 equiv) dropwise. Then the reaction was warmed to room temperature and monitored by TLC until the complete conversion. Then the mixture was quenched dropwise by a saturated aqueous NH_4Cl solution (5 mL). The organic layer was extracted with ethyl acetate (3×15 mL) and the combined organic layers were washed with saturated aqueous NaCl solution, dried over anhydrous $MgSO_4$, filtered and removed under vacuum. The residue was purified by a column chromatography on silica gel using ethyl acetate/petroleum ether as eluent to give the desired product **S4**. Then the product **S4** was dissolved in HFIP (5 mL) and TfOH (0.05 equiv) and PhOH (3.0 equiv) were added to the solution. The mixture was heated to reflux and monitored by TLC until the complete conversion. Then the reaction was quenched by saturated aqueous $NaHCO_3$ solution (10 mL). The organic layer was extracted with CH_2Cl_2 (3×15 mL) and the combined organic layers were washed with saturated aqueous NaCl solution, dried over anhydrous $MgSO_4$, filtered and removed under vacuum. The residue was purified by a column chromatography on silica gel using ethyl acetate/petroleum ether as eluent to give the desired product **5**.

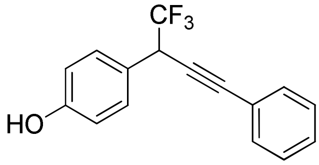

4-(1,1,1-Trifluoro-4-phenylbut-3-yn-2-yl)phenol (1a)

^{1}H NMR (500 MHz, $CDCl_3$) δ 7.52 (d, J = 7.4 Hz, 2H), 7.44 (d, J = 8.3 Hz, 2H), 7.39 – 7.32 (m, 3H), 6.88 (d, J = 8.4 Hz, 2H), 5.41 (brs, 1H), 4.52 (q, J = 8.0 Hz, 1H); ^{13}C NMR (126 MHz, $CDCl_3$) δ 156.2, 132.1, 131.0, 129.0, 128.6, 124.6 (q, J = 280.4 Hz), 124.4, 122.4, 115.8, 85.8, 82.1 (q, J = 3.2 Hz), 43.5 (q, J = 31.6 Hz); ^{19}F NMR (471 MHz, $CDCl_3$) δ -70.8; HRMS (ESI) m/z calculated for $C_{16}H_{10}F_3O$ $[M - H]^-$ 275.0689, found 275.0679.

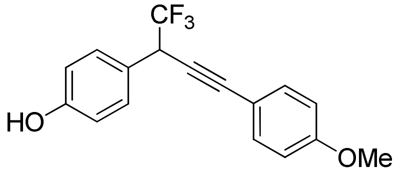

4-(1,1,1-Trifluoro-4-(4-methoxyphenyl)but-3-yn-2-yl)phenol (1b)

^{1}H NMR (500 MHz, $CDCl_3$) δ 7.46 – 7.38 (m, 4H), 6.88 – 6.82 (m, 4H), 4.48 (q, *J* = 8.0 Hz, 1H), 3.82 (s, 3H); ^{13}C NMR (126 MHz, $CDCl_3$) δ 160.1, 156.2, 133.6, 131.0, 124.6 (q, *J* = 280.4 Hz), 124.6, 115.7, 114.5, 114.2, 85.6, 80.6 (q, *J* = 3.3 Hz), 55.5, 43.5 (q, *J* = 31.5 Hz); ^{19}F NMR (471 MHz, $CDCl_3$) δ -70.9; HRMS (ESI) m/z calculated for $C_{17}H_{12}F_3O_2$ $[M - H]^-$ 305.0795, found 305.0803.

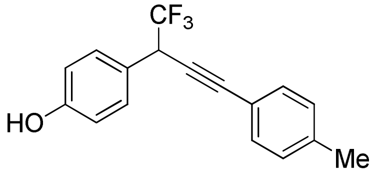

4-(1,1,1-Trifluoro-4-(p-tolyl)but-3-yn-2-yl)phenol (1c)

^{1}H NMR (500 MHz, $CDCl_3$) δ 7.46 – 7.36 (m, 4H), 7.15 (d, *J* = 7.9 Hz, 2H), 6.87 (d, *J* = 8.5 Hz, 2H), 5.11 (brs, 1H), 4.50 (q, *J* = 8.0 Hz, 1H), 2.37 (s, 3H); ^{13}C NMR (126 MHz, $CDCl_3$) δ 156.2, 139.2, 132.0, 131.0, 129.3, 124.6 (q, *J* = 280.4 Hz), 124.5, 119.3, 115.8, 85.9, 81.3 (q, *J* = 3.3 Hz), 43.5 (q, *J* = 31.5 Hz), 21.7; ^{19}F NMR (471 MHz, $CDCl_3$) δ -70.8; HRMS (ESI) m/z calculated for $C_{17}H_{12}F_3O$ $[M - H]^-$ 289.0846, found 289.0843.

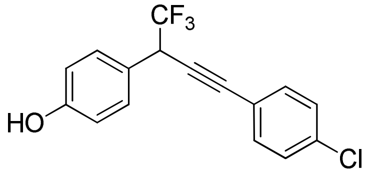

4-(4-(4-Chlorophenyl)-1,1,1-trifluorobut-3-yn-2-yl)phenol (1d)

^{1}H NMR (500 MHz, $CDCl_3$) δ 7.44 – 7.38 (m, 4H), 7.31 (d, *J* = 8.6 Hz, 2H), 6.88 (d, *J* = 8.6 Hz, 2H), 5.33 (brs, 1H), 4.49 (q, *J* = 8.0 Hz, 1H); ^{13}C NMR (126 MHz, $CDCl_3$) δ 156.3, 135.1, 133.3, 130.9, 128.9, 124.5 (q, *J* = 280.4 Hz), 124.1, 120.8, 115.9, 84.6, 83.1 (q, *J* = 3.3 Hz), 43.5 (q, *J* = 31.6 Hz); ^{19}F NMR (471 MHz, $CDCl_3$) δ -70.8; HRMS (ESI) m/z calculated for $C_{16}H_9ClF_3O$ $[M - H]^-$ 309.0300, found 309.0291.

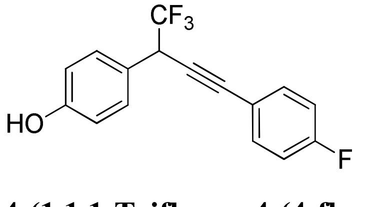

4-(1,1,1-Trifluoro-4-(4-fluorophenyl)but-3-yn-2-yl)phenol (1e)

^{1}H NMR (500 MHz, $CDCl_3$) δ 7.53 – 7.44 (m, 2H), 7.40 (d, *J* = 8.4 Hz, 2H), 7.09 – 6.98 (m, 2H), 6.87 (d, *J* = 8.4 Hz, 2H), 5.05 (brs, 1H), 4.48 (q, *J* = 8.0 Hz, 1H); ^{13}C NMR (126 MHz, $CDCl_3$) δ 163.0 (d, *J* = 250.1 Hz), 156.3, 134.1 (d, *J* = 8.5 Hz), 130.9, 124.5 (q, *J* = 280.4 Hz), 124.2, 118.4 (d, *J* = 3.5 Hz), 115.9 (d, *J* = 22.1 Hz), 115.8, 84.7, 81.8 (q, *J* = 1.7 Hz), 43.5 (q, *J* = 31.6 Hz); ^{19}F NMR (471 MHz, $CDCl_3$) δ -70.8, -110.1; HRMS (ESI) m/z calculated for $C_{16}H_9F_4O$ $[M - H]^-$ 293.0595, found 293.0587.

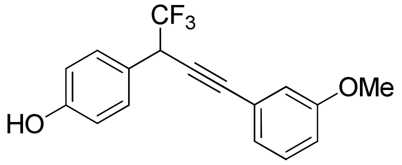

4-(1,1,1-Trifluoro-4-(3-methoxyphenyl)but-3-yn-2-yl)phenol (1f)

^{1}H NMR (500 MHz, $CDCl_3$) δ 7.38 (d, *J* = 8.3 Hz, 2H), 7.24 – 7.18 (m, 1H), 7.09 – 7.04 (m, 1H), 7.01 – 6.98 (m, 1H), 6.91 – 6.87 (m, 1H), 6.83 (d, *J* = 8.6 Hz, 2H), 5.26 (brs, 1H), 4.46 (q, *J* = 8.0 Hz, 1H), 3.78 (s, 3H); ^{13}C NMR (126 MHz, $CDCl_3$) δ 159.4, 156.2, 131.0, 129.7, 124.7, 124.6 (q, *J* = 280.4 Hz), 124.3, 123.4, 116.9, 115.8, 115.6, 85.6, 81.9 (q, *J* = 3.3 Hz), 55.6, 43.5 (q, *J* = 31.6 Hz); ^{19}F NMR (471 MHz, $CDCl_3$) δ -70.7; HRMS (ESI) m/z calculated for $C_{17}H_{12}F_3O_2$ $[M - H]^-$ 305.0795, found 305.0788.

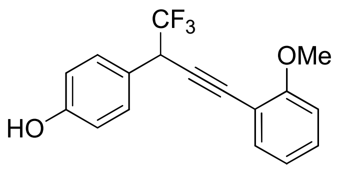

4-(1,1,1-Trifluoro-4-(2-methoxyphenyl)but-3-yn-2-yl)phenol (1g)

^{1}H NMR (500 MHz, $CDCl_3$) δ 7.49 – 7.42 (m, 3H), 7.35 – 7.30 (m, 1H), 6.96 – 6.90 (m, 1H), 6.89 (d, *J* = 8.4 Hz, 1H), 6.84 (d, *J* = 8.7 Hz, 2H), 5.08 (brs, 1H), 4.54 (q, *J* = 8.0 Hz, 1H), 3.88 (s, 3H); ^{13}C NMR (126 MHz, $CDCl_3$) δ 160.7, 156.2, 134.0, 131.1, 130.4, 124.6 (q, *J* = 280.5 Hz), 124.5, 120.7, 115.7, 111.7, 111.1, 86.1 (q, *J* = 3.4 Hz), 82.2, 56.1, 43.7 (q, *J* = 31.4 Hz); ^{19}F NMR (471 MHz, $CDCl_3$) δ -70.8; HRMS (ESI) m/z calculated for $C_{17}H_{12}F_3O_2$ $[M - H]^-$ 305.0795, found 305.0797.

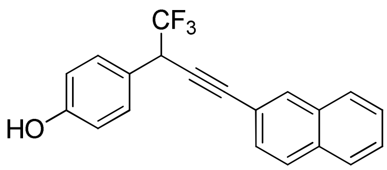

4-(1,1,1-Trifluoro-4-(naphthalen-2-yl)but-3-yn-2-yl)phenol (1h)

^{1}H NMR (500 MHz, $CDCl_3$) δ 8.04 (s, 1H), 7.85 – 7.78 (m, 3H), 7.56 – 7.49 (m, 3H), 7.47 (d, *J* = 8.5 Hz, 2H), 6.89 (d, *J* = 8.7 Hz, 2H), 5.21 (brs, 1H), 4.56 (q, *J* = 8.0 Hz, 1H); ^{13}C NMR (126 MHz, $CDCl_3$) δ 156.3, 133.2, 133.1, 132.2, 131.0, 128.6, 128.3, 128.0, 128.0, 127.1, 126.9, 124.6 (q, *J* = 280.5 Hz), 124.3, 119.6, 115.8, 86.1, 82.3 (q, *J* = 3.3 Hz), 43.6 (q, *J* = 31.5 Hz); ^{19}F NMR (471 MHz, $CDCl_3$) δ -70.7; HRMS (ESI) m/z calculated for $C_{20}H_{12}F_3O$ $[M - H]^-$ 325.0846, found 325.0843.

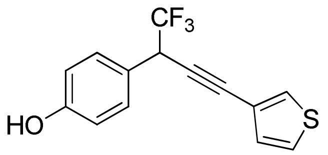

4-(1,1,1-Trifluoro-4-(thiophen-3-yl)but-3-yn-2-yl)phenol (1i)

^{1}H NMR (500 MHz, DMSO-*d*$_6$) δ 9.66 (brs, 1H), 7.93 – 7.85 (m, 1H), 7.65 – 7.59 (m, 1H), 7.35 (d, *J* = 8.5 Hz, 2H), 7.24 – 7.19 (m, 1H), 6.82 (d, *J* = 8.6 Hz, 2H), 5.21 (q, *J* = 8.7 Hz, 1H); ^{13}C NMR (126 MHz, DMSO-*d*$_6$) δ 157.9, 130.7, 130.4, 129.6, 127.0, 124.8 (q, *J* = 280.0 Hz), 121.7, 120.2, 115.5, 82.2 (q, *J* = 3.1 Hz), 80.0, 41.3 (q, *J* = 30.5 Hz); ^{19}F NMR (471 MHz, Acetone-*d*$_6$) δ -71.4; HRMS (ESI) m/z calculated for $C_{14}H_8F_3OS$ $[M - H]^-$ 281.0253, found 281.0248.

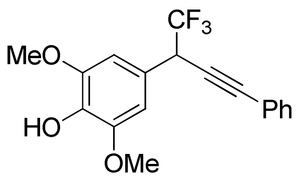

2,6-Dimethoxy-4-(1,1,1-trifluoro-4-phenylbut-3-yn-2-yl)phenol (1j)

^{1}H NMR (500 MHz, CD_3OD) δ 7.49 – 7.44 (m, 2H), 7.39 – 7.32 (m, 3H), 6.84 (s, 2H), 4.85 (brs, 1H), 4.79 (q, *J* = 8.2 Hz, 1H), 3.86 (s, 6H); ^{13}C NMR (126 MHz, CD_3OD) δ 149.2, 137.4, 132.7, 129.9, 129.6, 126.1 (q, *J* = 279.5 Hz),123.8, 123.5, 107.9, 86.3, 83.3 (q, *J* = 3.4 Hz), 56.9, 44.3 (q, *J* = 31.3 Hz); ^{19}F NMR (471 MHz, CD_3OD) δ -72.1; HRMS (ESI) m/z calculated for $C_{18}H_{14}F_3O_3$ $[M - H]^-$ 335.0901, found 335.0908.

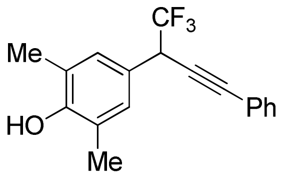

2,6-Dimethyl-4-(1,1,1-trifluoro-4-phenylbut-3-yn-2-yl)phenol (1k)

^{1}H NMR (500 MHz, $CDCl_3$) δ 7.54 – 7.47 (m, 2H), 7.38 – 7.31 (m, 3H), 7.15 (s, 2H), 4.70 (brs, 1H), 4.43 (q, *J* = 8.1 Hz, 1H), 2.28 (s, 6H); ^{13}C NMR (126 MHz, $CDCl_3$) δ 152.9, 132.1, 129.7, 128.9, 128.5, 126.9 (q, *J* = 280.7 Hz), 123.5, 122.6, 85.5, 82.4 (q, *J* = 3.1 Hz), 43.6 (q, *J* = 31.4 Hz), 16.1; ^{19}F NMR (471 MHz, $CDCl_3$) δ -70.7; HRMS (ESI) m/z calculated for $C_{18}H_{14}F_3O$ $[M - H]^-$ 303.1002, found 303.0998.

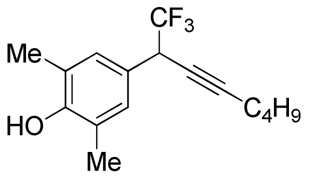

2,6-Dimethyl-4-(1,1,1-trifluorooct-3-yn-2-yl)phenol (1l)

^{1}H NMR (500 MHz, $CDCl_3$) δ 7.08 (s, 2H), 4.69 (brs, 1H), 4.18 (qt, *J* = 8.2, 2.2 Hz, 1H), 2.30 – 2.24 (m, 8H), 1.59 – 1.52 (m, 2H), 1.50 – 1.42 (m, 2H), 0.94 (t, *J* = 7.3 Hz, 3H); ^{13}C NMR (126 MHz, $CDCl_3$) δ 152.7, 129.6, 124.9 (q, *J* = 280.1 Hz), 124.2, 123.3, 86.2, 73.2 (q, *J* = 3.2 Hz), 43.0 (q, *J* = 31.1 Hz), 30.8, 22.1, 18.6, 16.1, 13.8; ^{19}F NMR (471 MHz, $CDCl_3$) δ -71.1;HRMS (ESI) m/z calculated for $C_{16}H_{18}F_3O$ $[M - H]^-$ 283.1315, found 283.1321.

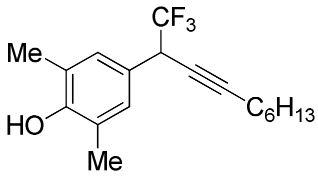

2,6-Dimethyl-4-(1,1,1-trifluorodec-3-yn-2-yl)phenol (1m)

^{1}H NMR (500 MHz, $CDCl_3$) δ 7.07 (s, 2H), 4.68 (brs, 1H), 4.17 (qt, *J* = 8.2, 2.2 Hz, 1H), 2.27 – 2.24 (m, 8H), 1.58 – 1.51 (m, 2H), 1.45 – 1.38 (m, 2H), 1.33 – 1.26 (m, 4H), 0.89 (t, *J* = 7.0 Hz, 3H); ^{13}C NMR (126 MHz, $CDCl_3$) δ 152.7, 129.6, 124.9 (q, *J* = 280.1 Hz), 124.2, 123.3, 86.3, 73.2 (q, *J* = 3.5 Hz), 43.0 (q, *J* = 30.9 Hz), 31.5, 28.7, 28.7, 22.8, 18.9, 16.1, 14.2; ^{19}F NMR (471 MHz, $CDCl_3$) δ -71.2; HRMS (ESI) m/z calculated for $C_{18}H_{22}F_3O$ $[M - H]^-$ 311.1628, found 311.1620.

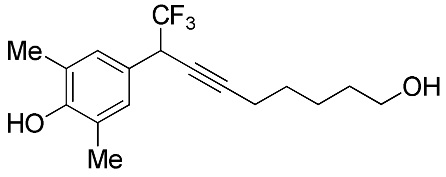

2,6-Dimethyl-4-(1,1,1-trifluoro-9-hydroxynon-3-yn-2-yl)phenol (1n)

^{1}H NMR (500 MHz, $CDCl_3$) δ 7.06 (s, 2H), 4.20 – 4.12 (m, 1H), 3.66 (t, J = 6.5 Hz, 2H), 2.30 – 2.23 (m, 8H), 1.63 – 1.55 (m, 4H), 1.52 – 1.47 (m, 2H); ^{13}C NMR (126 MHz, $CDCl_3$) δ 152.6, 129.4, 124.7 (q, J = 280.2 Hz), 123.8, 123.3, 85.7, 73.3 (q, J = 3.8 Hz), 62.9, 42.7 (q, J = 31.2 Hz), 32.2, 28.2, 24.9, 18.7, 16.0; ^{19}F NMR (471 MHz, $CDCl_3$) δ -71.1; HRMS (ESI) m/z calculated for $C_{17}H_{20}F_3O_2$ [M - H]$^-$ 313.1421, found 313.1423.

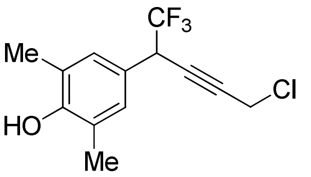

4-(5-Chloro-1,1,1-trifluoropent-3-yn-2-yl)-2,6-dimethylphenol (1o)

^{1}H NMR (500 MHz, $CDCl_3$) δ 7.04 (s, 2H), 4.28 – 4.23 (m, 1H), 4.21 (d, J = 2.1 Hz, 2H), 2.26 (s, 6H); ^{13}C NMR (126 MHz, $CDCl_3$) δ 153.0, 129.6, 124.4 (q, J = 280.4 Hz), 123.6, 122.7, 80.3, 79.8 (q, J = 3.0 Hz), 43.0 (q, J = 31.6 Hz), 30.4, 16.1; ^{19}F NMR (471 MHz, $CDCl_3$) δ -70.4; HRMS (ESI) m/z calculated for $C_{13}H_{11}ClF_3O$ [M - H]$^-$ 275.0456, found 275.0449.

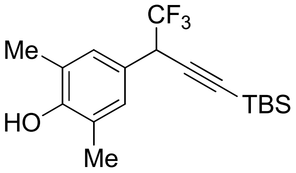

4-(4-(Tert-butyldimethylsilyl)-1,1,1-trifluorobut-3-yn-2-yl)-2,6-dimethylphenol (1p)

^{1}H NMR (500 MHz, $CDCl_3$) δ 7.09 (s, 2H), 4.70 (brs, 1H), 4.23 (q, J = 8.1 Hz, 1H), 2.26 (s, 6H), 0.98 (s, 9H), 0.15 (s, 3H), 0.15 (s, 3H); ^{13}C NMR (126 MHz, $CDCl_3$) δ 152.8, 129.8, 124.5 (q, J = 280.4 Hz),123.4, 123.3, 99.0 (q, J = 3.1 Hz),89.5, 43.8 (q, J = 31.2 Hz), 26.2, 16.9, 16.1, -4.6; ^{19}F NMR (471 MHz, $CDCl_3$) δ -70.9; HRMS (ESI) m/z calculated for $C_{18}H_{24}F_3OSi$ [M - H]$^-$341.1554, found 341.1568.

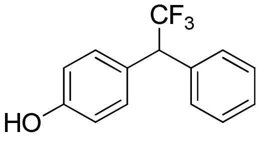

4-(2,2,2-Trifluoro-1-phenylethyl)phenol (5a)

^{1}H NMR (600 MHz, $CDCl_3$) δ 7.41 – 7.28 (m, 5H), 7.24 (d, *J* = 8.3 Hz, 2H), 6.81 (d, *J* = 8.6 Hz, 2H), 4.63 (q, *J* = 10.0 Hz, 1H); ^{13}C NMR (151 MHz, $CDCl_3$) δ 155.3, 135.8 (q, *J* = 1.0 Hz), 130.7, 129.2, 128.9, 128.0, 128.0 (q, *J* = 1.4 Hz), 126.4 (q, *J* = 280.4 Hz, 2H), 115.8, 54.9 (q, *J* = 27.5 Hz); ^{19}F NMR (471 MHz, $CDCl_3$) δ -66.1; HRMS (ESI) m/z calculated for $C_{14}H_{10}F_3O$ $[M - H]^-$ 251.0689, found 251.0694.

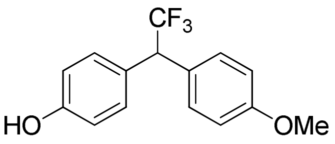

4-(2,2,2-Trifluoro-1-(4-methoxyphenyl)ethyl)phenol (5b)

^{1}H NMR (500 MHz, CD_3OD) δ 7.27 (d, *J* = 8.7 Hz, 2H), 7.19 (d, *J* = 8.5 Hz, 2H), 6.85 (d, *J* = 8.8 Hz, 2H), 6.76 (d, *J* = 8.7 Hz, 2H), 4.67 (q, *J* = 10.4 Hz, 1H), 3.72 (s, 3H); ^{13}C NMR (126 MHz, CD_3OD) δ 160.6, 158.1, 131.2, 131.2, 129.5, 128.1, 128.1 (q, *J* = 279.5 Hz), 116.3, 114.9, 55.6, 54.8 (q, *J* = 27.4 Hz); ^{19}F NMR (471 MHz, CD_3OD) δ -67.8; HRMS (ESI) m/z calculated for $C_{15}H_{12}F_3O_2$ $[M - H]^-$ 281.0795, found 281.0803.

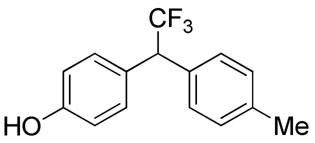

4-(2,2,2-Trifluoro-1-(p-tolyl)ethyl)phenol (5c)

^{1}H NMR (500 MHz, $CDCl_3$) δ 7.30 – 7.24 (m, 4H), 7.19 (d, *J* = 7.9 Hz, 2H), 6.82 (d, *J* = 8.6 Hz, 2H),5.19 (brs, 1H),4.61 (q, *J* = 10.0 Hz, 1H), 2.36 (s, 3H); ^{13}C NMR (126 MHz, $CDCl_3$) δ 155.2, 137.8, 132.9, 130.6, 129.6, 129.0, 128.2 (q, *J* = 1.5 Hz), 126.5 (q, *J* = 280.4 Hz), 115.7, 54.6 (q, *J* = 27.5 Hz), 21.2; ^{19}F NMR (471 MHz, $CDCl_3$) δ -66.2; HRMS (ESI) m/z calculated for $C_{15}H_{12}F_3O$ $[M - H]^-$ 265.0846, found 265.0839.

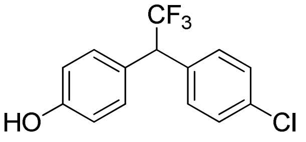

4-(1-(4-Chlorophenyl)-2,2,2-trifluoroethyl)phenol (5d)

^{1}H NMR (500 MHz, $CDCl_3$) δ 7.34 – 7.27 (m, 4H), 7.20 (d, *J* = 8.6 Hz, 2H), 6.82 (d, *J* = 8.7 Hz, 2H), 5.08 (brs, 1H), 4.60 (q, *J* = 9.8 Hz, 1H); ^{13}C NMR (126 MHz, $CDCl_3$) δ 155.6, 134.4, 134.1, 130.6, 130.6, 129.1, 127.4, 126.2 (q, *J* = 280.4 Hz), 115.9, 54.3 (q, *J* = 27.8 Hz); ^{19}F NMR (471 MHz, $CDCl_3$) δ -66.3; HRMS (ESI) m/z calculated for $C_{14}H_9ClF_3O$ $[M - H]^-$ 285.0300, found 285.0306.

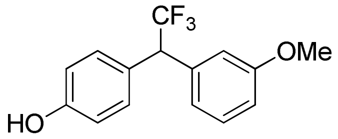

4-(2,2,2-Trifluoro-1-(3-methoxyphenyl)ethyl)phenol (5e)

^{1}H NMR (500 MHz, $CDCl_3$) δ 7.28 – 7.23 (m, 1H), 7.21 (d, *J* = 8.6 Hz, 2H), 6.96 (d, *J* = 7.7 Hz, 1H), 6.91 (s, 1H), 6.86 – 6.82 (m, 1H), 6.76 (d, *J* = 8.7 Hz, 2H), 5.53 (brs, 1H), 4.57 (q, *J* = 9.9 Hz, 1H), 3.77 (s, 3H); ^{13}C NMR (126 MHz, $CDCl_3$) δ 159.8, 155.4, 137.3, 130.6, 129.9, 127.8, 126.4 (q, *J* = 280.5 Hz), 121.7, 115.8, 115.5, 113.1, 55.5, 54.9 (q, *J* = 27.6 Hz); ^{19}F NMR (471 MHz, $CDCl_3$) δ -66.0; HRMS (ESI) m/z calculated for $C_{15}H_{12}F_3O_2$ $[M - H]^-$ 281.0795, found 281.0799.

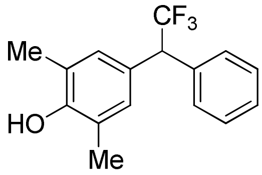

2,6-Dimethyl-4-(2,2,2-trifluoro-1-phenylethyl)phenol (5f)

^{1}H NMR (600 MHz, $CDCl_3$) δ 7.42 – 7.34 (m, 4H), 7.34 – 7.30 (m, 1H), 7.00 (s, 2H), 4.65 (brs, 1H), 4.57 (q, *J* = 10.1 Hz, 1H), 2.24 (s, 6H); ^{13}C NMR (126 MHz, $CDCl_3$) δ 152.1, 136.1 (q, *J* = 1.2 Hz), 129.5, 129.2, 128.9, 127.9, 127.2 (q, *J* = 1.3 Hz), 126.6 (q, *J* = 280.5 Hz), 123.5, 55.1 (q, *J* = 27.4 Hz), 16.2; ^{19}F NMR (471 MHz, $CDCl_3$) δ -66.0; HRMS (ESI) m/z calculated for $C_{16}H_{14}F_3O$ $[M - H]^-$ 279.1002, found 279.0993.

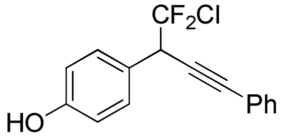

4-(1-Chloro-1,1-difluoro-4-phenylbut-3-yn-2-yl)phenol (10a)

^{1}H NMR (500 MHz, CD_3OD) δ 7.51 – 7.42 (m, 2H), 7.38 (d, 2H), 7.35 – 7.26 (m, 3H), 6.85 – 6.81 (m, 2H), 4.84 – 4.73 (m, 1H); ^{13}C NMR (126 MHz, CD_3OD) δ 159.1, 132.6, 132.1, 130.2 (t, *J* = 295.0 Hz), 129.8, 129.5, 124.8, 123.6, 116.2, 86.7, 84.4 (t, *J* = 4.5 Hz), 50.4 (t, *J* = 27.2 Hz); ^{19}F NMR (471 MHz, CD_3OD) δ -51.64 – -54.25 (m, 2F); HRMS (ESI) m/z calculated for $C_{16}H_{10}ClF_2O$ $[M - H]^-$ 291.0394, found 291.0399.

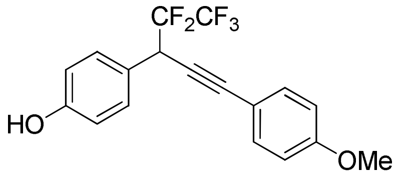

4-(4,4,5,5,5-Pentafluoro-1-(4-methoxyphenyl)pent-1-yn-3-yl)phenol (10b)

^{1}H NMR (500 MHz, CD_3OD) δ 7.38 – 7.31 (m, 4H), 6.89 – 6.86 (m, 2H), 6.82 (d, *J* = 8.6 Hz, 2H), 4.75 – 4.65 (m, 1H), 3.77 (s, 3H); ^{13}C NMR (126 MHz, CD_3OD) δ 161.5, 159.2, 134.1, 132.0, 123.5, 125.4 – 116.1 (m), 116.4, 115.5, 115.1, 86.8, 81.3 (dd, *J* = 9.3, 2.5 Hz),55.8, 41.6 (dd, *J* = 26.6, 22.4 Hz); ^{19}F NMR (471 MHz, CD_3OD) δ -82.3 – -82.5 (m, 3F), -115.7 – -122.6 (m, 2F); HRMS (ESI) m/z calculated for $C_{18}H_{12}F_5O_2$ $[M - H]^-$ 355.0763, found 355.0755.

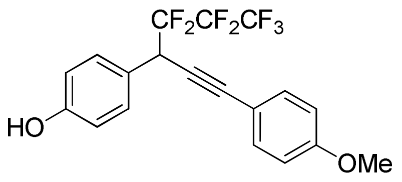

4-(4,4,5,5,6,6,6-Heptafluoro-1-(4-methoxyphenyl)hex-1-yn-3-yl)phenol (10c)

^{1}H NMR (500 MHz, $CDCl_3$) δ 7.44 – 7.39 (m, 4H), 6.89 – 6.85 (m, 4H), 5.48 (brs, 1H), 4.61 – 4.52 (m, 1H), 3.82 (s, 3H); ^{13}C NMR (126 MHz, $CDCl_3$) δ 160.1, 156.3, 133.5, 131.6, 123.8, 115.7, 114.6, 121.99 – 106.96 (m), 114.2, 86.3, 80.3 (d, *J* = 9.8 Hz), 55.5, 41.1 (dd, *J* = 27.7, 22.6 Hz); ^{19}F NMR (471 MHz, $CDCl_3$) δ -80.6 – -80.7 (m, 3F), -111.5 – -112.3 (m, 1F), -116.5 – -117.3 (m,1F), -122.6 – -125.3 (m, 2F); HRMS (ESI) m/z calculated for $C_{19}H_{12}F_7O_2$ $[M - H]^-$ 405.0731, found 405.0744.

General procedure

General procedure for asymmetric CDC of racemic *p*-hydroxybenzyl CF_3 moieties with heteroarenes

A mixture of **1** or **5** (0.1 mmol, 1.0 equiv), activated MnO_2 (0.3 mmol, 3.0 equiv), and DDQ (0.025 mmol, 0.25 equiv) in anhydrous CH_2Cl_2 (1.0 mL) was stirred at 60 °C in a sealed tube and monitored by TLC. Upon starting material consumption, the mixture was cooled to -78 °C directly and stirred at the same temperature for 15 min. (Note: the paraquinomethide could be stable in this system.) Then 3Å molecular sieves (20 mg), K_2CO_3 (0.2 mmol, 2.0 equiv), **2a** (0.3 mmol, 3.0 equiv) and catalyst **3c** (0.005 mmol, 0.05 equiv) were added in sequence quickly. (Attention: the order of addition is important.) Then the mixture was stirred at the same temperature and monitored by TLC. Upon completion, the reaction was warm to room temperature and a solution of saturated aqueous NaCl solution (1 ml) was added to the mixture. Then the mixture was extracted with EtOAc, and the combined organic layer was dried over $MgSO_4$, filtered and evaporated under vacuum. The residue was purified by a flash column chromatography to give the desired product.

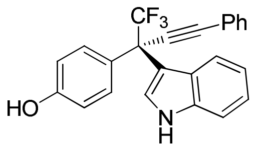

(*S*)-4-(1,1,1-Trifluoro-2-(1*H*-indol-3-yl)-4-phenylbut-3-yn-2-yl)phenol (4a)

It was purified by silica gel chromatography using EtOAc/petroleum ether (1:4). Yield: 86% (33.7 mg). ^{1}H NMR (600 MHz, $CDCl_3$) δ 8.17 (brs, 1H), 7.53 (d, *J* = 8.6 Hz, 2H), 7.50 – 7.45 (m, 2H), 7.42 – 7.27 (m, 6H), 7.20 – 7.15 (m, 1H), 7.01 – 6.96 (m, 1H), 6.77 (d, *J* = 8.8 Hz, 2H); ^{13}C NMR (151 MHz, $CDCl_3$) δ 155.7, 136.6, 132.1, 130.7, 129.1, 128.8, 128.5, 125.8 (q, *J* = 284.2 Hz), 125.7, 123.0 (q, *J* = 2.6 Hz), 122.6, 122.6, 121.3, 120.1, 115.2, 112.6, 111.4, 86.5, 86.2 (q, *J* = 1.5 Hz), 51.2 (q, *J* = 29.3 Hz); ^{19}F NMR (471 MHz, $CDCl_3$) δ -70.5; HRMS (ESI) m/z calculated for $C_{24}H_{15}F_3NO$ $[M - H]^-$ 390.1111, found 390.1116; HPLC: the ee value was determined by HPLC analysis (Chiralpak AS-H, *i*-PrOH/hexane = 15/85, 1.0 mL/min, 227 nm), retention time: t_{minor} = 13.790 min, t_{major} = 16.100 min, ee = 92.6%; $[\alpha]_D^{30}$ = -96.5 (c = 0.98, $CHCl_3$).

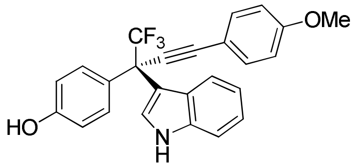

(*S*)-4-(1,1,1-Trifluoro-2-(1*H*-indol-3-yl)-4-(4-methoxyphenyl)but-3-yn-2-yl)phenol (4b)

It was purified by silica gel chromatography using EtOAc/petroleum ether (1:4). Yield: 89% (37.5 mg). ^{1}H NMR (600 MHz, $CDCl_3$) δ 8.17 (brs, 1H), 7.53 (d, *J* = 8.6 Hz, 2H), 7.43 – 7.31 (m, 5H), 7.18 – 7.15 (m, 1H), 6.99 – 6.95 (m, 1H), 6.82 (d, *J* = 8.9 Hz, 2H), 6.76 (d, *J* = 8.8 Hz, 2H), 3.80 (s, 3H); ^{13}C NMR (151 MHz, $CDCl_3$) δ 160.0, 155.7, 136.6, 133.5, 130.7, 129.3, 125.8 (q, *J* = 284.0 Hz), 125.7, 123.0 (q, *J* = 2.4 Hz), 122.6, 121.3, 120.0, 115.1, 114.8, 114.1, 112.8, 111.3, 86.4, 84.8 (q, *J* = 1.5 Hz), 55.5, 51.2 (q, *J* = 29.1 Hz); ^{19}F NMR (471 MHz, $CDCl_3$) δ -70.7; HRMS (ESI) m/z calculated for $C_{25}H_{17}F_3NO_2$ [M - H]$^-$ 420.1217, found 420.1211; HPLC: the ee value was determined by HPLC analysis (Chiralpak AS-H, *i*-PrOH/hexane = 15/85, 1.0 mL/min, 266 nm), retention time: t_{minor} = 12.620 min, t_{major} = 15.593 min, ee = 95.1%; $[\alpha]_D^{30}$ = -119.3 (c = 1.00, $CHCl_3$).

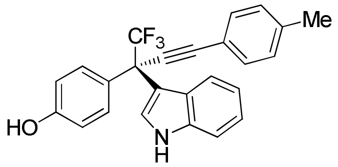

(*S*)-4-(1,1,1-Trifluoro-2-(1*H*-indol-3-yl)-4-(p-tolyl)but-3-yn-2-yl)phenol (4c)

It was purified by silica gel chromatography using EtOAc/petroleum ether (1:4). Yield: 85% (34.4 mg). ^{1}H NMR (500 MHz, CD_3OD) δ 7.42 (d, *J* = 8.7 Hz, 2H), 7.39 – 7.34 (m, 2H), 7.34 – 7.28 (m, 2H), 7.23 – 7.19 (m, 1H), 7.17 – 7.12 (m, 2H), 7.10 – 7.04 (m, 1H), 6.88 – 6.82 (m, 1H), 6.73 (d, *J* = 8.8 Hz, 2H), 2.32 (s, 3H); ^{13}C NMR (126 MHz, CD_3OD) δ 158.6, 140.1, 138.3, 132.6, 131.3, 130.2, 129.0, 127.2 (q, *J* = 282.9 Hz), 126.8, 124.2 (q, *J* = 2.5 Hz), 122.7, 121.7, 120.8, 119.9, 115.7, 112.7, 112.4, 87.2, 87.0 (q, *J* = 1.7 Hz), 52.4 (q, *J* = 29.1 Hz), 21.4; ^{19}F NMR (471 MHz, CD_3OD) δ -71.9; HRMS (ESI) m/z calculated for $C_{25}H_{17}F_3NO$ [M - H]$^-$ 404.1268, found 404.1280; HPLC: the ee value was determined by HPLC analysis (Chiralpak AS-H, *i*-PrOH/hexane = 15/85, 1.0 mL/min, 258 nm), retention time: t_{minor} = 14.363 min, t_{major} = 17.240 min, ee = 89.9%; $[\alpha]_D^{30}$ = -95.3 (c = 1.00, $CHCl_3$).

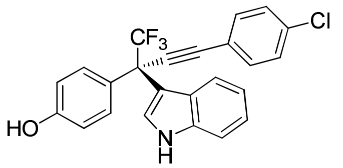

(*S*)-4-(4-(4-Chlorophenyl)-1,1,1-trifluoro-2-(1*H*-indol-3-yl)but-3-yn-2-yl)phenol (4d)

It was purified by silica gel chromatography using EtOAc/petroleum ether (1:4). Yield: 80% (34.0 mg). ^{1}H NMR (500 MHz, $CDCl_3$) δ 8.20 (brs, 1H), 7.50 (d, *J* = 8.6 Hz, 2H), 7.42 – 7.36 (m, 4H), 7.32 – 7.26 (m, 3H), 7.21 – 7.15 (m, 1H), 7.01 – 6.95 (m, 1H), 6.77 (d, *J* = 8.8 Hz, 2H); ^{13}C NMR (126 MHz, $CDCl_3$) δ 155.6, 136.4, 134.7, 133.1, 130.4, 128.6, 128.6, 125.5 (q, *J* = 284.2 Hz), 125.5, 122.9 (q, *J* = 2.6 H), 122.5, 121.0, 120.9, 119.9, 115.0, 112.2, 111.2, 87.1 (q, *J* = 1.5 Hz), 85.18, 51.1 (q, *J* = 29.4 Hz); ^{19}F NMR (471 MHz, $CDCl_3$) δ -70.6; HRMS (ESI) m/z calculated for $C_{24}H_{14}ClF_3NO$ $[M - H]^-$ 424.0721, found 424.0715; HPLC: the ee value was determined by HPLC analysis (Chiralpak AS-H, *i*-PrOH/hexane = 15/85, 1.0 mL/min, 253 nm), retention time: t_{minor} = 12.620 min, t_{major} = 15.593 min, ee = 91.2%; $[\alpha]_D^{30}$ = -125.8 (c = 1.02, $CHCl_3$).

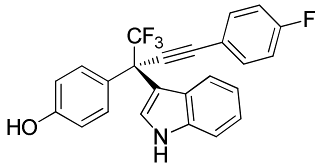

(*S*)-4-(1,1,1-Trifluoro-4-(4-fluorophenyl)-2-(1*H*-indol-3-yl)but-3-yn-2-yl)phenol (4e)

It was purified by silica gel chromatography using EtOAc/petroleum ether (1:4). Yield: 76% (31.0 mg). ^{1}H NMR (500 MHz, $CDCl_3$) δ 8.19 (brs, 1H), 7.51 (d, *J* = 8.6 Hz, 2H), 7.47 – 7.42 (m, 2H), 7.42 – 7.37 (m, 2H), 7.33 – 7.29 (m, 1H), 7.19 – 7.15 (m, 1H), 7.03 – 6.93 (m, 3H), 6.77 (d, *J* = 8.8 Hz, 2H), 4.83 (brs, 1H); ^{13}C NMR (126 MHz, $CDCl_3$) δ 162.9 (d, *J* = 249.8 Hz), 155.8, 136.6, 134.0 (d, *J* = 8.4 Hz), 130.7, 129.0, 125.7 (q, *J* = 284.0 Hz), 125.7, 123.0 (q, *J* = 2.6 Hz), 122.7, 121.2, 120.1, 118.7 (d, *J* = 3.5 Hz), 115.8 (d, *J* = 22.1 Hz), 115.2, 112.5, 111.4, 86.0 (q, *J* = 1.6 Hz), 85.5, 51.2 (q, *J* = 29.1 Hz); ^{19}F NMR (471 MHz, $CDCl_3$) δ -70.7, -110.4; HRMS (ESI) m/z calculated for $C_{24}H_{14}F_4NO$ $[M - H]^-$ 408.1017, found 408.1008; HPLC: the ee value was determined by HPLC analysis (Chiralpak AS-H, *i*-PrOH/hexane = 15/85, 1.0 mL/min, 215 nm), retention time: t_{minor} = 13.690 min, t_{major} = 16.470 min, ee = 95.8%; $[\alpha]_D^{30}$ = -105.5 (c = 0.98, $CHCl_3$).

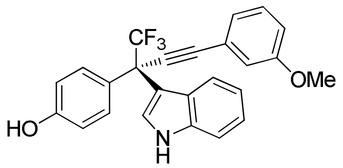

(*S*)-4-(1,1,1-Trifluoro-2-(1*H*-indol-3-yl)-4-(3-methoxyphenyl)but-3-yn-2-yl)phenol (4f)

It was purified by silica gel chromatography using EtOAc/petroleum ether (1:4). Yield: 85% (35.6 mg). ^{1}H NMR (500 MHz, $CDCl_3$) δ 8.17 (brs, 1H), 7.53 (d, *J* = 8.6 Hz, 2H), 7.40 – 7.33 (m, 3H), 7.23 – 7.15 (m, 2H), 7.10 – 7.06 (m, 1H), 7.01 – 6.96 (m, 2H), 6.90 – 6.86 (m, 1H), 6.76 (d, *J* = 8.8 Hz, 2H), 4.92 (brs, 1H), 3.77 (s, 3H); ^{13}C NMR (126 MHz, $CDCl_3$) δ 159.5, 155.7, 136.6, 130.7, 129.6, 129.0, 125.8 (q, *J* = 284.3 Hz), 125.7, 124.7, 123.6, 123.1 (q, *J* = 2.7 Hz), 122.7, 121.3, 120.1, 116.9, 115.5, 115.2, 112.6, 111.4, 86.4, 86.1 (q, *J* = 1.7 Hz), 55.5, 51.3 (q, *J* = 29.3 Hz); ^{19}F NMR (471 MHz, $CDCl_3$) δ -70.6; HRMS (ESI) m/z calculated for $C_{25}H_{17}F_3NO_2$ $[M - H]^-$ 420.1217, found 420.1222; HPLC: the ee value was determined by HPLC analysis (Chiralpak AS-H, *i*-PrOH/hexane = 15/85, 1.0 mL/min, 225nm), retention time: t_{minor} = 16.943 min, t_{major} = 19.467 min, ee = 92.3%; $[\alpha]_D^{30}$ = -113.6 (c = 0.99, $CHCl_3$).

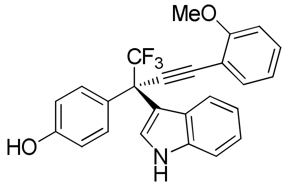

(*S*)-4-(1,1,1-Trifluoro-2-(1*H*-indol-3-yl)-4-(2-methoxyphenyl)but-3-yn-2-yl)phenol (4g)

It was purified by silica gel chromatography using EtOAc/petroleum ether (1:4). Yield: 81% (34.0 mg). ^{1}H NMR (600 MHz, $CDCl_3$) δ 8.16 (brs, 1H), 7.60 (d, *J* = 8.6 Hz, 2H), 7.45 – 7.38 (m, 3H), 7.36 – 7.33 (m, 1H), 7.30 – 7.26 (m, 1H), 7.17 – 7.13 (m, 1H), 6.98 – 6.95 (m, 1H), 6.89 – 6.85 (m, 2H), 6.76 (d, *J* = 8.8 Hz, 2H), 3.85 (s, 3H); ^{13}C NMR (151 MHz, $CDCl_3$) δ 160.9, 155.6, 136.6, 133.7, 130.8, 130.2, 129.3, 125.8 (q, *J* = 284.3 Hz), 125.7, 123.1 (q, *J* = 2.4 Hz), 122.5, 121.7, 120.5, 119.9, 115.1, 112.7, 112.1, 111.2, 111.0, 90.1 (q, *J* = 1.4 Hz), 83.1, 56.0, 51.4 (q, *J* = 29.2 Hz); ^{19}F NMR (471 MHz, $CDCl_3$) δ -70.5; HRMS (ESI) m/z calculated for $C_{25}H_{17}F_3NO_2$ $[M - H]^-$ 420.1217, found 420.1219; HPLC: the ee value was determined by HPLC analysis (Chiralpak AS-H, *i*-PrOH/hexane = 15/85, 1.0 mL/min, 291 nm), retention time: t_{minor} = 23.080 min, t_{major} = 26.093 min, ee = 96.2%; $[\alpha]_D^{30}$ = -79.4 (c = 0.99, $CHCl_3$).

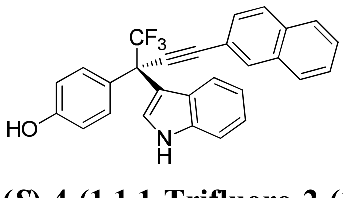

(*S*)-4-(1,1,1-Trifluoro-2-(1*H*-indol-3-yl)-4-(naphthalen-2-yl)but-3-yn-2-yl)phenol (4h)

It was purified by silica gel chromatography using EtOAc/petroleum ether (1:4). Yield: 67% (29.5 mg). ^{1}H NMR (500 MHz, $CDCl_3$) δ 8.17 (brs, 1H), 8.00 (s, 1H), 7.81 – 7.74 (m, 3H), 7.58 (d, *J* = 8.6 Hz, 2H), 7.53 – 7.46 (m, 3H), 7.44 – 7.35 (m, 3H), 7.22 – 7.16 (m, 1H), 7.04 – 6.99 (m, 1H), 6.78 (d, *J* = 8.8 Hz, 2H), 4.99 (brs, 1H); ^{13}C NMR (126 MHz, $CDCl_3$) δ 155.8, 136.6, 133.2, 133.1, 132.1, 130.7, 129.1, 128.7, 128.1, 128.0, 128.0, 127.0, 126.8, 125.8 (q, *J* = 284.3 Hz), 125.7, 123.1 (q, *J* = 2.2 Hz), 122.7, 121.3, 120.1, 119.9, 115.2, 112.6, 111.4, 86.9, 86.5 (q, *J* = 1.6 Hz), 51.3 (q, *J* = 29.4 Hz); ^{19}F NMR (471 MHz, $CDCl_3$) δ -70.5; HRMS (ESI) m/z calculated for $C_{28}H_{17}F_3NO$ $[M - H]^-$ 440.1268, found 440.1257; HPLC: the ee value was determined by HPLC analysis (Chiralpak AS-H, *i*-PrOH/hexane = 20/80, 1.0 mL/min, 290 nm), retention time: t_{minor} =10.457 min, t_{major} = 12.797min, ee = 92.1%; $[\alpha]_D^{30}$ = -63.7 (c = 1.03, $CHCl_3$).

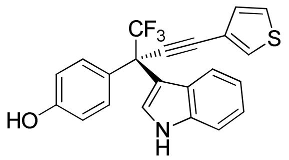

(*S*)-4-(1,1,1-Trifluoro-2-(1*H*-indol-3-yl)-4-(thiophen-3-yl)but-3-yn-2-yl)phenol (4i)

It was purified by silica gel chromatography using EtOAc/petroleum ether (1:4). Yield: 83% (32.8 mg). ^{1}H NMR (500 MHz, $CDCl_3$) δ 8.18 (brs, 1H), 7.51 (d, *J* = 8.6 Hz, 2H), 7.49 – 7.45 (m, 1H), 7.40 – 7.35 (m, 2H), 7.34 – 7.30 (m, 1H), 7.26 – 7.23 (m, 1H), 7.19 – 7.15 (m, 1H), 7.14 – 7.11 (m, 1H), 7.00 – 6.95 (m, 1H), 6.76 (d, *J* = 8.8 Hz, 2H); ^{13}C NMR (126 MHz, $CDCl_3$) δ 155.8, 136.6, 130.7, 130.2, 129.6, 129.0, 125.7 (q, *J* = 284.2 Hz), 125.7, 125.5, 123.1 (q, *J* = 2.7 Hz), 122.6, 121.6, 121.3, 120.1, 115.2, 112.5, 111.4, 85.8 (q, *J* = 1.6 Hz), 81.7, 51.3 (q, *J* = 29.4 Hz); ^{19}F NMR (471 MHz, $CDCl_3$) δ -70.6; HRMS (ESI) m/z calculated for $C_{22}H_{13}F_3NOS$ $[M - H]^-$ 396.0675, found 396.0683; HPLC: the ee value was determined by HPLC analysis (Chiralpak AS-H, *i*-PrOH/hexane = 15/85, 1.0 mL/min, 215 nm), retention time: t_{minor} =20.300 min, t_{major} = 22.877min, ee = 96.2%; $[\alpha]_D^{30}$ = -93.8 (c = 0.98, $CHCl_3$).

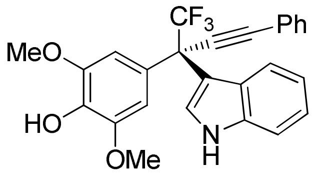

(*S*)-2,6-Dimethoxy-4-(1,1,1-trifluoro-2-(1*H*-indol-3-yl)-4-phenylbut-3-yn-2-yl)phenol (4j)

It was purified by silica gel chromatography using EtOAc/petroleum ether (1:4). Yield: 63% (28.5 mg). ^{1}H NMR (500 MHz, $CDCl_3$) δ 8.27 (brs, 1H), 7.50 – 7.45 (m, 2H), 7.43 – 7.35 (m, 3H), 7.35 – 7.27 (m, 3H), 7.19 – 7.15 (m, 1H), 7.03 – 6.98 (m, 1H), 6.96 (s, 2H), 5.59 (brs, 1H), 3.78 (s, 6H); ^{13}C NMR (126 MHz, $CDCl_3$) δ 146.7, 136.6, 135.1, 132.0, 128.9, 128.5, 127.8, 125.8 (q, *J* = 284.4 Hz), 125.8, 123.2 (q, *J* = 2.5 Hz), 122.6, 122.6, 121.3, 120.1, 112.4, 111.4, 106.7, 86.7, 86.2, 56.6, 51.9 (q, *J* = 29.4 Hz); ^{19}F NMR (471 MHz, $CDCl_3$) δ -70.2; HRMS (ESI) m/z calculated for $C_{26}H_{19}F_3NO_3$ $[M - H]^-$ 450.1323, found 450.1327; HPLC: the the ee value was determined by HPLC analysis (Chiralpak IG, *i*-PrOH/hexane = 20/80, 1.0 mL/min, 220 nm), retention time: t_{minor} = 7.267 min, t_{minor} = 10.190 min, ee = 83.0%; $[\alpha]_D^{30}$ = -9.6 (c = 1.04, $CHCl_3$).

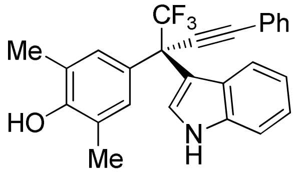

(*S*)-2,6-Dimethyl-4-(1,1,1-trifluoro-2-(1*H*-indol-3-yl)-4-phenylbut-3-yn-2-yl)phenol (4k)

It was purified by silica gel chromatography using EtOAc/petroleum ether (1:4). Yield: 76% (32.0 mg). ^{1}H NMR (500 MHz, $CDCl_3$) δ 8.14 (brs, 1H), 7.52 – 7.47 (m, 2H), 7.46 – 7.43 (m, 1H), 7.39 – 7.35 (m, 2H), 7.34 – 7.27 (m, 5H), 7.20 – 7.15 (m, 1H), 7.03 – 6.98 (m, 1H), 4.65 (brs, 1H), 2.20 (s, 6H); ^{13}C NMR (126 MHz, $CDCl_3$) δ 152.4, 136.6, 132.1, 129.3, 128.7, 128.4, 128.0, 125.9 (q, *J* = 284.2 Hz), 125.8, 123.0 (q, *J* = 2.3 Hz),122.8, 122.7, 122.5, 121.4, 120.0, 112.8, 111.3, 86.5 (q, *J* = 1.1 Hz), 86.4, 51.3 (q, *J* = 28.1 Hz), 16.3; ^{19}F NMR (471 MHz, $CDCl_3$) δ -70.4; HRMS (ESI) m/z calculated for $C_{26}H_{19}F_3NO$ $[M - H]^-$ 418.1424, found 418.1432; HPLC: the the ee value was determined by HPLC analysis (Chiralpak IG, *i*-PrOH/hexane = 5/95, 1.0 mL/min, 241 nm), retention time: t_{minor} = 10.850 min, t_{major} = 16.043 min, ee = 90.5%; $[\alpha]_D^{30}$ = -67.4 (c = 0.99, $CHCl_3$).

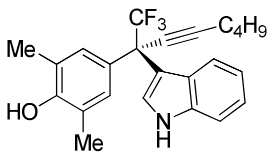

(*S*)-2,6-Dimethyl-4-(1,1,1-trifluoro-2-(1*H*-indol-3-yl)oct-3-yn-2-yl)phenol (4l)

It was prepared using **3b** with 1.1 equiv of **2a** without K_2CO_3 additive and asymmetric nucleophilic addition was performed at 0 °C. Then it was purified by silica gel chromatography using EtOAc/petroleum ether (1:4). Yield: 73% (29.3 mg). ^{1}H NMR (500 MHz, $CDCl_3$) δ 8.08 (brs, 1H), 7.37 – 7.28 (m, 3H), 7.22 (s, 2H), 7.19 – 7.13 (m, 1H), 7.00 – 6.94 (m, 1H), 2.31 (t, *J* = 7.0 Hz, 2H), 2.19 (s, 6H), 1.61 – 1.52 (m, 2H), 1.51 – 1.41 (m, 2H), 0.92 (t, *J* = 7.3 Hz, 3H); ^{13}C NMR (126 MHz, $CDCl_3$) δ 152.2, 136.6, 129.4, 128.5, 126.0 (q, *J* = 283.9 Hz), 125.8, 122.8 (q, *J* = 2.5 Hz), 122.5, 122.4, 121.6, 119.7, 113.4, 111.2, 77.32 – 77.28 (m), 87.2, 50.6 (q, *J* = 28.7 Hz), 30.7, 22.1, 18.7, 16.3, 13.8; ^{19}F NMR (471 MHz, $CDCl_3$) δ -70.8; HRMS (ESI) m/z calculated for $C_{24}H_{23}F_3NO$ [M - H]$^-$ 398.1737, found 398.1746; HPLC: the ee value was determined by HPLC analysis (Chiralpak IG, *i*-PrOH/hexane = 5/95, 1.0 mL/min, 254 nm), retention time: t_{major} =7.550 min, t_{minor} = 8.260min, ee = 92.7%; $[\alpha]_D^{30}$ = +32.2 (c = 0.96, $CHCl_3$).

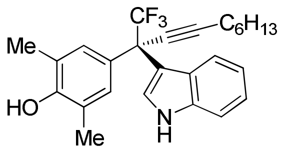

(*S*)-2,6-Dimethyl-4-(1,1,1-trifluoro-2-(1*H*-indol-3-yl)dec-3-yn-2-yl)phenol (4m)

It was prepared using **3b** with 1.1 equiv of **2a** without K_2CO_3 additive and asymmetric nucleophilic addition was performed at 0 °C. Then it was purified by silica gel chromatography using EtOAc/petroleum ether (1:4). Yield: 71% (30.2 mg). ^{1}H NMR (500 MHz, $CDCl_3$) δ 8.07 (brs, 1H), 7.37 – 7.29 (m, 3H), 7.22 (s, 2H), 7.19 – 7.14 (m, 1H), 7.00 – 6.94 (m, 1H), 4.64 (brs, 1H), 2.32 – 2.28 (m, 2H), 2.19 (s, 6H), 1.61 – 1.54 (m, 2H), 1.47 – 1.40 (m, 2H), 1.33 – 1.27 (m, 4H), 0.92 – 0.87 (m, 3H); ^{13}C NMR (126 MHz, $CDCl_3$) δ 152.2, 136.6, 129.4, 128.5, 126.0 (q, *J* = 283.9 Hz), 125.8, 122.8 (q, *J* = 2.4 Hz), 122.5, 122.4, 121.5, 119.7, 113.4, 111.2, 87.3, 77.35 – 77.30 (m), 50.6 (q, *J* = 29.0 Hz), 31.5, 28.7, 28.6, 22.8, 19.1, 16.3, 14.2; ^{19}F NMR (471 MHz, $CDCl_3$) δ -70.8; HRMS (ESI) m/z calculated for $C_{26}H_{27}F_3NO$ [M - H]$^-$ 426.2050, found 426.2062; HPLC: the ee

value was determined by HPLC analysis (Chiralpak IG, *i*-PrOH/hexane = 3/97, 1.0 mL/min, 215 nm), retention time: t_{major} =11.460 min, t_{minor} = 13.030min, ee = 91.2%; $[\alpha]_D^{30}$ = +27.1 (c = 1.03, $CHCl_3$).

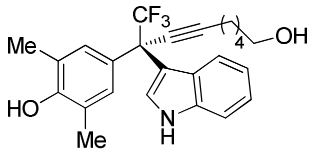

(*S*)-2,6-Dimethyl-4-(1,1,1-trifluoro-6-hydroxy-2-(1*H*-indol-3-yl)hex-3-yn-2-yl)phenol (4n)

It was prepared using **3b** with 1.1 equiv of **2a** without K_2CO_3 additive and asymmetric nucleophilic addition was performed at 0 °C. Then it was purified by silica gel chromatography using EtOAc/petroleum ether (1:4). Yield: 70% (30.1 mg). ^{1}H NMR (500 MHz, $CDCl_3$) δ 8.18 (brs, 1H), 7.35 – 7.28 (m, 3H), 7.20 (s, 2H), 7.17 – 7.12 (m, *J* = 7.5 Hz, 1H), 6.98 – 6.93 (m, *J* = 7.6 Hz, 1H), 3.55 (t, *J* = 6.5 Hz, 2H), 2.32 (t, *J* = 6.8 Hz, 2H), 2.18 (s, 6H), 1.62 – 1.49 (m, 4H), 1.48 – 1.40 (m, 2H); ^{13}C NMR (126 MHz, $CDCl_3$) δ 152.3, 136.6, 129.3, 128.4, 126.0 (q, *J* = 284.1 Hz), 125.8, 122.8 (q, *J* = 2.3 Hz), 122.6, 122.4, 121.5, 119.6, 113.3, 111.3, 86.8, 77.7 (q, *J* = 1.8 Hz), 63.0, 50.6 (q, *J* = 28.4 Hz), 32.4, 28.3, 25.0, 19.0, 16.3; ^{19}F NMR (471 MHz, $CDCl_3$) δ -70.8; HRMS (ESI) m/z calculated for $C_{25}H_{25}F_3NO_2$ $[M - H]^-$ 428.1843, found 428.1840; HPLC: the ee value was determined by HPLC analysis (Chiralpak AD-H, *i*-PrOH/hexane = 20/80, 1.0 mL/min, 220 nm), retention time: t_{minor} =7.697 min, t_{major} = 9.317min, ee = 91.2%; $[\alpha]_D^{30}$ = +50.8 (c = 1.01, $CHCl_3$).

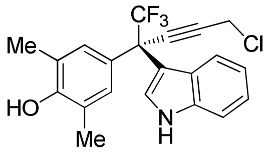

(*S*)-4-(5-Chloro-1,1,1-trifluoro-2-(1*H*-indol-3-yl)pent-3-yn-2-yl)-2,6-dimethylphenol (4o)

It was prepared using **3b** with 1.1 equiv of **2a** without K_2CO_3 additive. Then it was purified by silica gel chromatography using EtOAc/petroleum ether (1:4). Yield: 66% (25.7 mg). ^{1}H NMR (500 MHz, $CDCl_3$) δ 8.12 (brs, 1H), 7.37 – 7.32 (m, 2H), 7.29 (d, *J* = 8.1 Hz, 1H), 7.22 – 7.15 (m, 3H), 7.03 – 6.97 (m, 1H), 4.22 (d, *J* = 2.7 Hz, 2H), 2.19 (s, 6H); ^{13}C NMR (126 MHz, $CDCl_3$) δ 152.5, 136.5, 129.2, 127.3, 125.6 (q, *J* = 284.3 Hz), 125.6, 123.2 (q, *J* = 2.6 Hz), 122.8, 122.6, 121.3, 120.1, 112.1, 111.4, 83.8 (q, *J* = 1.7 Hz), 81.2, 50.8 (q, *J* = 29.2 Hz), 30.7, 16.3; ^{19}F NMR (471 MHz, $CDCl_3$) δ -70.3; HRMS (ESI) m/z calculated for $C_{21}H_{16}ClF_3NO$ $[M - H]^-$ 390.0878, found 390.0887; HPLC:

the ee value was determined by HPLC analysis (Chiralpak AS-H, *i*-PrOH/hexane = 15/85, 1.0 mL/min, 225 nm), retention time: t_{major} =15.707 min, t_{minor} = 19.847min, ee = 86.2%; $[\alpha]_D^{30}$ = +42.8 (c = 1.00, $CHCl_3$).

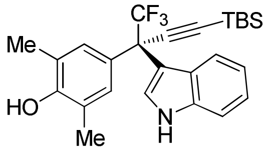

(*R*)-4-(4-(*tert*-Butyldimethylsilyl)-1,1,1-trifluoro-2-(1*H*-indol-3-yl)but-3-yn-2-yl)-2,6-dimethylphenol (4p)

It was prepared using **3b** with 1.1 equiv of **2a** without K_2CO_3 additive. Then it was purified by silica gel chromatography using EtOAc/petroleum ether (1:4). Yield: 70% (31.8 mg). ^{1}H NMR (500 MHz, $CDCl_3$) δ 7.94 (brs, 1H), 7.42 (d, *J* = 8.1 Hz, 1H), 7.30 – 7.21 (m, 4H), 7.20 – 7.14 (m, 1H), 7.04 – 6.96 (m, 1H), 2.19 (s, 6H), 1.01 (s, 9H), 0.15 (s, 3H), 0.13 (s, 3H); ^{13}C NMR (126 MHz, $CDCl_3$) δ 152.3, 136.5, 129.4, 127.9, 125.7 (q, *J* = 284.2 Hz), 125.6, 123.1 (q, *J* = 2.5 Hz), 122.6, 122.5, 121.6, 119.7, 112.2, 111.3, 102.9, 90.2, 51.5 (q, *J* = 28.8 Hz), 26.3, 17.0, 16.3, -4.7, -4.7; ^{19}F NMR (471 MHz, $CDCl_3$) δ -70.5; HRMS (ESI) m/z calculated for $C_{26}H_{29}F_3NOSi$ $[M - H]^-$ 456.1976, found 456.1968; HPLC: the ee value was determined by HPLC analysis (ChiralcelOD-H, *i*-PrOH/hexane = 5/95, 1.0 mL/min, 254 nm), retention time: t_{major} =12.580 min, t_{minor} = 14.453min, ee = 93.1%; $[\alpha]_D^{30}$ = +60.8 (c = 1.02, $CHCl_3$).

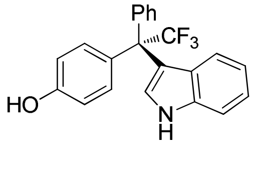

(*S*)-4-(2,2,2-Trifluoro-1-(1*H*-indol-3-yl)-1-phenylethyl)phenol (6a)

It was prepared using **3d** with 1.1 equiv of **2a** without K_2CO_3 additive. Then it was purified by silica gel chromatography using EtOAc/petroleum ether (1:4). Yield: 77% (28.2 mg). ^{1}H NMR (500 MHz, CD_3OD) δ 7.36 (d, *J* = 8.2 Hz, 1H), 7.32 – 7.23 (m, 5H), 7.10 (d, *J* = 8.7 Hz, 2H), 7.07 – 7.03 (m, 1H), 6.89 (d, *J* = 8.2 Hz, 1H), 6.82 – 6.78 (m, 1H), 6.72 (d, *J* = 8.9 Hz, 2H), 6.68 (s, 1H); ^{13}C NMR (126 MHz, CD_3OD) δ 157.8, 141.7, 138.6, 132.1, 131.9, 130.8, 129.7 (q, *J* = 285.8 Hz), 128.8, 128.4, 128.0, 127.6, 122.9, 122.5, 119.9, 116.3, 115.5, 112.4, 61.1 (q, *J* = 25.0 Hz); ^{19}F NMR (471 MHz,

CD_3OD) δ -61.9; HRMS (ESI) m/z calculated for $C_{22}H_{15}F_3NO$ $[M - H]^-$ 366.1111, found 366.1123; HPLC:the the ee value was determined by HPLC analysis (Chiralpak AD-H, *i*-PrOH/hexane = 10/90, 1.0 mL/min, 227 nm), retention time: t_{major} = 20.460 min, t_{minor} = 24.720 min, ee = 93.2%; $[\alpha]_D^{30}$ = -1.8 (c = 0.97, $CHCl_3$).

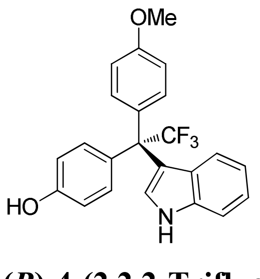

(*R*)-4-(2,2,2-Trifluoro-1-(1*H*-indol-3-yl)-1-(4-methoxyphenyl)ethyl)phenol (6b)

It was prepared using **3d** with 1.1 equiv of **2a** without K_2CO_3 additive. Then it was purified by silica gel chromatography using EtOAc/petroleum ether (1:4). Yield: 70% (27.8 mg). ^{1}H NMR (500 MHz, DMSO-d_6) δ 11.20 (brs, 1H), 9.57 (brs, 1H), 7.40 (d, *J* = 8.2 Hz, 1H), 7.12 (d, *J* = 8.8 Hz, 2H), 7.07 – 7.02 (m, 1H), 7.00 (d, *J* = 8.7 Hz, 2H), 6.91 (d, *J* = 9.1 Hz, 2H), 6.83 – 6.79 (m, 1H), 6.77 – 6.71 (m, 4H), 3.75 (s, 3H); ^{13}C NMR (126 MHz, DMSO-d_6) δ 158.3, 156.6, 136.9, 131.5, 130.3, 129.6, 129.6, 128.3 (q, *J* = 286.3 Hz), 126.8, 125.8, 121.1, 121.1, 118.8, 114.8, 114.2, 113.4, 111.9, 58.5 (q, *J* = 24.3 Hz), 55.0; ^{19}F NMR (471 MHz, DMSO-d_6) δ -60.5; HRMS (ESI) m/z calculated for $C_{23}H_{17}F_3NO_2$ $[M - H]^-$ 396.1217, found 396.1211; HPLC: the the ee value was determined by HPLC analysis (Chiralpak AD-H, *i*-PrOH/hexane = 20/80, 1.0 mL/min, 230 nm), retention time: t_{major} = 8.960 min, t_{minor} = 10.687 min, ee = 91.2%; $[\alpha]_D^{30}$ = -1.6 (c = 0.11, $CHCl_3$).

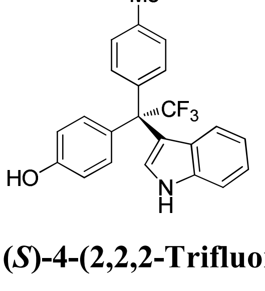

(*S*)-4-(2,2,2-Trifluoro-1-(1*H*-indol-3-yl)-1-(*p*-tolyl)ethyl)phenol (6c)

It was prepared using **3d** with 1.1 equiv of **2a** without K_2CO_3 additive. Then it was purified by silica gel chromatography using EtOAc/petroleum ether (1:4). Yield: 67% (25.7 mg). ^{1}H NMR (500 MHz, CD_3OD) δ 7.35 (d, *J* = 8.2 Hz, 1H), 7.19 – 7.15 (m, 2H), 7.11 – 7.02 (m, 5H), 6.91 – 6.87 (m, 1H), 6.81 – 6.77 (m, 1H), 6.72 – 6.66 (m, 3H), 2.32 – 2.28 (m, 3H); ^{13}C NMR (126 MHz, CD_3OD) δ

157.7, 138.7, 138.6, 138.2, 132.2, 132.1, 130.7, 129.8 (q, J = 285.5 Hz), 129.5, 127.9, 127.7, 123.0, 122.4, 119.9, 116.5, 115.5, 112.4, 60.8 (q, J = 24.6 Hz), 20.9; ^{19}F NMR (471 MHz, CD_3OD) δ -61.9; HRMS (ESI) m/z calculated for $C_{23}H_{17}F_3NO$ [M - H]$^-$ 380.1268, found 380.1263; HPLC:the the ee value was determined by HPLC analysis (Chiralpak AD-H, *i*-PrOH/hexane = 15/85, 0.3 mL/min, 215 nm), retention time: t_{major} =36.220 min, t_{minor} = 38.910min, ee = 90.3%; $[\alpha]_D^{30}$ = -2.3 (c = 1.04, $CHCl_3$).

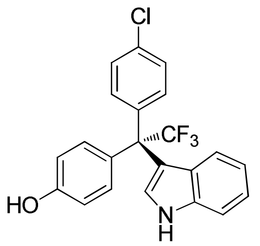

(*R*)-4-(1-(4-Chlorophenyl)-2,2,2-trifluoro-1-(1*H*-indol-3-yl)ethyl)phenol (6d)

It was prepared using **3d** with 1.1 equiv of **2a** without K_2CO_3 additive. Then it was purified by silica gel chromatography using EtOAc/petroleum ether (1:4). Yield: 62% (24.7 mg). ^{1}H NMR (500 MHz, CD_3OD) δ 7.36 (d, J = 8.2 Hz, 1H), 7.30 – 7.27 (m, 4H), 7.11 – 7.04 (m, 3H), 6.88 – 6.80 (m, 2H), 6.74 – 6.69 (m, 3H); ^{13}C NMR (126 MHz, CD_3OD) δ 158.1, 140.5, 138.6, 134.6, 132.5, 132.0, 131.4, 129.53 (q, J = 285.6 Hz), 129.0, 128.0, 127.4, 122.7, 122.6, 120.1, 115.8, 115.7, 112.5, 60.8 (q, J = 25.1 Hz); ^{19}F NMR (471 MHz, CD_3OD) δ -61.8; HRMS (ESI) m/z calculated for $C_{22}H_{14}ClF_3NO$ [M - H]$^-$ 400.0721, found 400.0713; HPLC: the the ee value was determined by HPLC analysis (Chiralpak AD-H, *i*-PrOH/hexane = 15/85, 0.8 mL/min, 215 nm), retention time: t_{major} =10.847 min, t_{minor} = 12.213min, ee = 91.0%; $[\alpha]_D^{30}$ = -4.1 (c = 1.00, $CHCl_3$).

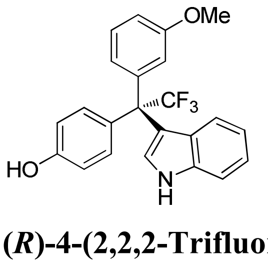

(*R*)-4-(2,2,2-Trifluoro-1-(1*H*-indol-3-yl)-1-(3-methoxyphenyl)ethyl)phenol (6e)

It was prepared using **3d** with 1.1 equiv of **2a** without K_2CO_3 additive. Then it was purified by silica gel chromatography using EtOAc/petroleum ether (1:4). Yield: 58% (23.0 mg). ^{1}H NMR (500 MHz, CD_3OD) δ 7.36 (d, J = 8.2 Hz, 1H), 7.23 – 7.16 (m, 1H), 7.12 – 7.03 (m, 3H), 6.94 – 6.88 (m, 2H), 6.88 – 6.79 (m, 3H), 6.73 – 6.69 (m, 3H), 3.63 – 3.60 (m, 3H); ^{13}C NMR (126 MHz, CD_3OD) δ

160.7, 157.8, 143.2, 138.6, 132.1, 131.9, 129.7, 129.7 (q, *J* = 285.6 Hz), 128.0, 127.6, 123.2, 122.9, 122.5, 119.9, 117.4, 116.2, 115.5, 113.4, 112.4, 61.1 (q, *J* = 25.3 Hz), 55.5; ^{19}F NMR (471 MHz, CD_3OD) δ -61.8; HRMS (ESI) m/z calculated for $C_{23}H_{17}F_3NO_2$ [M - H]$^-$ 396.1217, found 396.1219; HPLC: the the ee value was determined by HPLC analysis (Chiralpak AD-H, *i*-PrOH/hexane = 15/85, 1.0 mL/min, 232 nm), retention time: t_{major} =11.793 min, t_{minor} = 13.543min, ee = 91.0%; $[\alpha]_D^{30}$ = -1.2 (c = 0.92, $CHCl_3$).

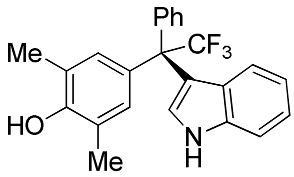

(*S*)-2,6-Dimethyl-4-(2,2,2-trifluoro-1-(1*H*-indol-3-yl)-1-phenylethyl)phenol (6f)

It was prepared using **3d** with 1.1 equiv of **2a** without K_2CO_3 additive. Then it was purified by silica gel chromatography using EtOAc/petroleum ether (1:4). Yield: 77% (30.3 mg). ^{1}H NMR (500 MHz, $CDCl_3$) δ 8.11 (brs, 1H), 7.38 – 7.30 (m, 6H), 7.19 – 7.15 (m, 1H), 7.06 – 7.02 (m, 1H), 6.97 – 6.91 (m, 3H), 6.70 – 6.67 (m, 1H), 2.16 (s, 6H); ^{13}C NMR (126 MHz, $CDCl_3$) δ 151.7, 140.3, 136.9, 131.4, 130.2 (q, *J* = 2.0 Hz), 129.9 (q, *J* = 2.0 Hz), 128.4 (q, *J* = 286.6 Hz), 128.1, 127.6, 126.9, 126.6, 122.7 (q, *J* = 3.0 Hz), 122.5, 122.3, 120.0, 116.7, 111.3, 60.1 (q, *J* = 25.0 Hz), 16.4; ^{19}F NMR (471 MHz, $CDCl_3$) δ -60.3; HRMS (ESI) m/z calculated for $C_{24}H_{19}F_3NO$ [M - H]$^-$ 394.1424, found 394.1432; HPLC: the ee value was determined by HPLC analysis (Chiralpak AD-H, *i*-PrOH/hexane = 10/90, 1.0 mL/min, 215 nm), retention time: t_{major} = 13.397 min, t_{minor} = 14.537 min, ee = 96.5%; $[\alpha]_D^{30}$ = -3.6 (c = 1.01, $CHCl_3$).

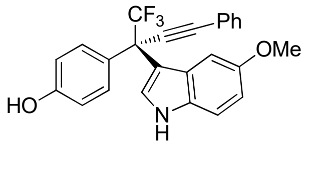

(*S*)-4-(1,1,1-Trifluoro-2-(5-methoxy-1*H*-indol-3-yl)-4-phenylbut-3-yn-2-yl)phenol (7a)

It was purified by silica gel chromatography using EtOAc/petroleum ether (1:4). Yield: 88% (37.1mg). ^{1}H NMR (500 MHz, $CDCl_3$) δ 8.1 (brs, 1H), 7.55 – 7.51 (m, 2H), 7.50 – 7.46 (m, 2H), 7.38 – 7.35 (m, 1H), 7.33 – 7.28 (m, 3H), 7.26 – 7.24 (m, 1H), 6.85 – 6.81 (m, 1H), 6.79 – 6.75 (m, 3H), 3.61 (s, 3H); ^{13}C NMR (126 MHz, $CDCl_3$) δ 155.5, 153.8, 131.9, 131.5, 130.5, 128.8, 128.6, 128.3, 126.1, 125.6 (q, *J* = 284.2 Hz), 123.5 (q, *J* = 2.7 Hz), 122.5, 115.0, 112.7, 112.1, 111.8, 102.9,

86.3, 86.0 (q, J = 1.9 Hz), 55.7, 51.0 (q, J = 29.2 Hz); ^{19}F NMR (471 MHz, $CDCl_3$) δ -70.6; HRMS (ESI) m/z calculated for $C_{25}H_{17}F_3NO_2$ [M - H]$^-$ 420.1217, found 420.1215; HPLC: the ee value was determined by HPLC analysis (Chiralpak AD-H, *i*-PrOH/hexane = 20/80, 1.0 mL/min, 215nm), retention time: t_{minor} = 8.703 min, t_{major} = 10.577 min, ee = 95.3%; $[\alpha]_D^{30}$ = -112.5 (c = 1.08, $CHCl_3$).

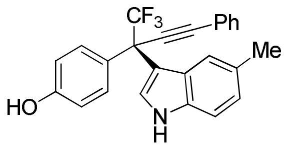

(*S*)-4-(1,1,1-Trifluoro-2-(5-methyl-1*H*-indol-3-yl)-4-phenylbut-3-yn-2-yl)phenol (7b)

It was purified by silica gel chromatography using EtOAc/petroleum ether (1:4). Yield: 85% (34.3 mg). ^{1}H NMR (500 MHz, $CDCl_3$) δ 8.04 (brs, 1H), 7.54 (d, J = 8.6 Hz, 2H), 7.51 – 7.47 (m, 2H), 7.35 – 7.29 (m, 4H), 7.25 (d, J = 8.3 Hz, 1H), 7.19 (s, 1H), 7.01 (dd, J = 8.3, 1.3 Hz, 1H), 6.76 (d, J = 8.8 Hz, 2H), 2.32 (s, 3H); ^{13}C NMR (126 MHz, $CDCl_3$) δ 155.6, 134.9, 132.0, 130.6, 129.2, 129.1, 128.8, 128.5, 125.9, 125.8 (q, J = 284.2 Hz), 124.3, 123.3 (q, J = 2.6 Hz), 122.7, 120.9, 115.2, 111.9, 111.0, 86.5– 86.4 (m), 51.3 (q, J = 29.2 Hz), 21.8; ^{19}F NMR (471 MHz, $CDCl_3$) δ -70.5; HRMS (ESI) m/z calculated for $C_{25}H_{17}F_3NO$ [M - H]$^-$ 404.1268, found 404.1263; HPLC: the ee value was determined by HPLC analysis (Chiralpak AD-H, *i*-PrOH/hexane = 10/90, 1.0 mL/min, 239 nm), retention time: t_{minor} = 25.420 min, t_{major} = 27.323 min, ee = 90.3%; $[\alpha]_D^{30}$ = -146.5 (c = 1.03, $CHCl_3$).

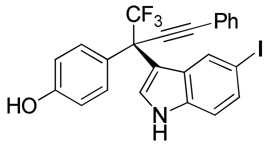

(*S*)-4-(1,1,1-Trifluoro-2-(5-iodo-1*H*-indol-3-yl)-4-phenylbut-3-yn-2-yl)phenol (7c)

It was purified by silica gel chromatography using EtOAc/petroleum ether (1:4). Yield: 82% (42.2 mg). ^{1}H NMR (500 MHz, $CDCl_3$) δ 8.20 (brs, 1H), 7.83 – 7.79 (m, 1H), 7.54 – 7.48 (m, 4H), 7.44 – 7.41 (m, 1H), 7.36 – 7.30 (m, 4H), 7.16 – 7.11 (m, 1H), 6.79 (d, J = 8.7 Hz, 2H), 4.97 (brs, 1H); ^{13}C NMR (126 MHz, $CDCl_3$) δ 155.7, 135.5, 131.9, 131.0, 130.4, 129.9, 128.8, 128.4, 128.0, 125.4 (q, J = 284.1 Hz), 123.6 (q, J = 2.5 Hz), 122.2, 115.2, 113.3, 111.8, 86.8, 85.7 (q, J = 1.7 Hz), 83.6, 50.9 (q, J = 29.6 Hz); ^{19}F NMR (471 MHz, $CDCl_3$) δ -70.7; HRMS (ESI) m/z calculated for $C_{24}H_{14}F_3INO$ [M - H]$^-$ 516.0078, found 516.0086; HPLC: the ee value was determined by HPLC analysis

(Chiralpak AD-H, *i*-PrOH/hexane = 20/80, 1.0 mL/min, 254 nm), retention time: t_{major} = 5.477 min, t_{minor} = 8.657 min, ee = 95.1%; $[\alpha]_D^{30}$ = -122.2 (c = 1.01, $CHCl_3$).

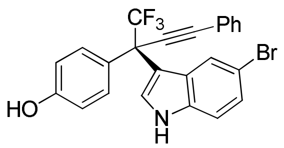

(*S*)-4-(2-(5-Bromo-1*H*-indol-3-yl)-1,1,1-trifluoro-4-phenylbut-3-yn-2-yl)phenol (7d)

It was purified by silica gel chromatography using EtOAc/petroleum ether (1:4). Yield: 80% (37.5 mg). ^{1}H NMR (500 MHz, $CDCl_3$) δ 8.24 (brs, 1H), 7.55 (s, 1H), 7.53 – 7.46 (m, 4H), 7.41 – 7.39 (m, 1H), 7.35 – 7.30 (m, 3H), 7.26 – 7.25 (m, 2H), 6.79 (d, *J* = 8.8 Hz, 2H); ^{13}C NMR (126 MHz, $CDCl_3$) δ 155.7, 135.1, 131.9, 130.4, 128.8, 128.4, 128.4, 127.3, 125.5, 125.4 (q, *J* = 284.2 Hz), 124.0 (q, *J* = 2.8 Hz), 123.6, 122.2, 115.2, 113.3, 112.7, 112.2, 86.7, 85.6 (q, *J* = 1.9 Hz), 50.9 (q, *J* = 29.5 Hz); ^{19}F NMR (471 MHz, $CDCl_3$) δ -70.7; HRMS (ESI) m/z calculated for $C_{24}H_{14}BrF_3NO$ [M - H]$^-$ 468.0216, found 468.0220; HPLC: the ee value was determined by HPLC analysis (Chiralpak AD-H, *i*-PrOH/hexane = 20/80, 1.0 mL/min, 236 nm), retention time: t_{major} = 5.623 min, t_{minor} = 6.840 min, ee = 95.4%; $[\alpha]_D^{30}$ = -125.0 (c = 1.04, $CHCl_3$).

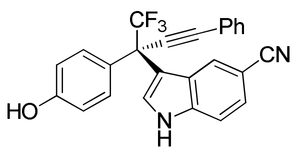

(*S*)-3-(1,1,1-Trifluoro-2-(4-hydroxyphenyl)-4-phenylbut-3-yn-2-yl)-1*H*-indole-5-carbonitrile (7e)

It was purified by silica gel chromatography using EtOAc/petroleum ether (1:4). Yield: 76% (31.8 mg). ^{1}H NMR (600 MHz, CD_3OD) δ 7.60 – 7.56 (m, 2H), 7.56 – 7.53 (m, 1H), 7.47 – 7.41 (m, 4H), 7.39 – 7.33 (m, 4H), 6.79 (d, *J* = 8.9 Hz, 2H); ^{13}C NMR (151 MHz, CD_3OD) δ 159.1, 140.1, 132.6, 131.2, 130.1, 129.7, 128.1, 127.1, 126.9 (q, *J* = 283.3 Hz), 126.7, 125.6, 123.3, 121.5, 116.1, 114.0, 113.9, 103.0, 87.8, 86.9 (q, *J* = 1.4 Hz), 52.2 (q, *J* = 29.4 Hz); ^{19}F NMR (471 MHz, $CDCl_3$) δ -71.0; HRMS (ESI) m/z calculated for $C_{25}H_{14}F_3N_2O$ [M - H]$^-$ 415.1064, found 415.1069; HPLC: the ee value was determined by HPLC analysis (Chiralpak AD-H, *i*-PrOH/hexane = 20/80, 1.0 mL/min, 247 nm), retention time: t_{major} = 4.800 min, t_{minor} = 5.500 min, ee = 92.8%; $[\alpha]_D^{30}$ = -124.9 (c = 1.01, THF).

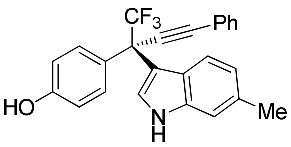

(*S*)-4-(1,1,1-Trifluoro-2-(6-methyl-1*H*-indol-3-yl)-4-phenylbut-3-yn-2-yl)phenol (7f)

It was purified by silica gel chromatography using EtOAc/petroleum ether (1:4). Yield: 81% (32.8 mg). ^{1}H NMR (500 MHz, $CDCl_3$) δ 8.04 (brs, 1H), 7.53 (d, J = 8.6 Hz, 2H), 7.49 – 7.46 (m, 2H), 7.34 – 7.28 (m, 4H), 7.21 (d, J = 8.2 Hz, 1H), 7.16 (s, 1H), 6.82 (dd, J = 8.2, 0.8 Hz, 1H), 6.76 (d, J = 8.8 Hz, 2H), 4.84 (brs, 1H), 2.41 (s, 3H); ^{13}C NMR (126 MHz, $CDCl_3$) δ 155.7, 137.1, 132.5, 132.1, 130.7, 129.2, 128.8, 128.4, 125.8 (q, J = 284.3 Hz), 123.6, 122.7, 122.5 (q, J = 2.6 Hz), 121.9, 120.9, 115.2, 112.5, 111.3, 86.4, 86.4, 51.2 (q, J = 29.2 Hz), 21.8; ^{19}F NMR (471 MHz, $CDCl_3$) δ -70.6; HRMS (ESI) m/z calculated for $C_{25}H_{17}F_3NO$ [M - H]$^-$ 404.1268, found 404.1259; HPLC: the ee value was determined by HPLC analysis (Chiralpak AD-H, *i*-PrOH/hexane = 20/80, 1.0 mL/min, 215 nm), retention time: t_{minor} = 8.347 min, t_{major} = 11.900 min, ee = 95.8%; $[\alpha]_D^{30}$ = -113.3 (c = 0.97, $CHCl_3$).

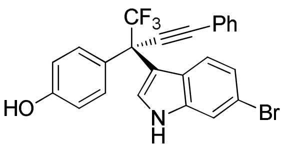

(*S*)-4-(2-(6-Bromo-1*H*-indol-3-yl)-1,1,1-trifluoro-4-phenylbut-3-yn-2-yl)phenol (7g)

It was purified by silica gel chromatography using EtOAc/petroleum ether (1:4). Yield: 78% (36.7 mg). ^{1}H NMR (600 MHz, CD_3OD) δ 7.55 (d, J = 1.5 Hz, 1H), 7.44 – 7.38 (m, 5H), 7.36 – 7.31 (m, 3H), 7.11 (d, J = 8.6 Hz, 1H), 6.98 (dd, J = 8.6, 1.8 Hz, 1H), 6.75 (d, J = 8.9 Hz, 2H); ^{13}C NMR (151 MHz, CD_3OD) δ 158.8, 139.1, 132.7, 131.3, 129.9, 129.6, 128.5, 127.1 (q, J = 283.1 Hz), 125.8, 125.2 (q, J = 2.2 Hz), 123.6, 123.2, 123.0, 116.3, 115.8, 115.4, 113.0, 87.3, 87.3, 52.2 (q, J = 29.2 Hz); ^{19}F NMR (471 MHz, $CDCl_3$) δ -67.1; HRMS (ESI) m/z calculated for $C_{24}H_{14}BrF_3NO$ [M - H]$^-$ 468.0216, found 468.0214; HPLC: the ee value was determined by HPLC analysis (Chiralpak AD-H, *i*-PrOH/hexane = 20/80, 1.0 mL/min, 215 nm), retention time: t_{minor} = 6.293 min, t_{major} = 8.167 min, ee = 95.0%; $[\alpha]_D^{30}$ = -136.9 (c = 1.01, THF).

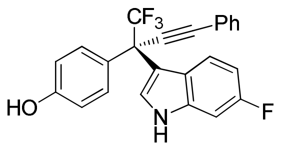

(*S*)-4-(1,1,1-Trifluoro-2-(6-fluoro-1*H*-indol-3-yl)-4-phenylbut-3-yn-2-yl)phenol (7h)

It was purified by silica gel chromatography using EtOAc/petroleum ether (1:4). Yield: 75% (30.7 mg). ^{1}H NMR (500 MHz, $CDCl_3$) δ 8.18 (brs, 1H), 7.53 – 7.49 (m, 2H), 7.48 – 7.45 (m, 2H), 7.39 – 7.37 (m, 1H), 7.34 – 7.28 (m, 3H), 7.25 – 7.22 (m, 1H), 7.07 – 7.04 (m, 1H), 6.80 – 6.72 (m, 3H); ^{13}C NMR (126 MHz, $CDCl_3$) δ 160.3 (d, *J* = 238.9 Hz), 155.9, 136.6 (d, *J* = 12.3 Hz), 132.1, 130.7, 128.9, 128.9, 128.5, 125.7 (q, *J* = 284.7 Hz), 123.4 (q, *J* = 2.5 Hz), 122.5, 122.3, 122.1 (d, *J* = 10.1 Hz), 115.2, 112.9, 109.1 (d, *J* = 24.4 Hz), 97.7 (d, *J* = 26.2 Hz), 86.7, 86.1 (q, *J* = 1.7 Hz), 51.2 (q, *J* = 29.7 Hz); ^{19}F NMR (471 MHz, $CDCl_3$) δ -70.8, -120.5; HRMS (ESI) m/z calculated for $C_{24}H_{14}F_4NO$ $[M - H]^-$ 408.1017, found 408.1007; HPLC: the ee value was determined by HPLC analysis (Chiralpak IB, *i*-PrOH/hexane = 10/90, 1.0 mL/min, 218 nm), retention time: t_{minor} = 12.190 min, t_{major} = 13.153 min, ee = 93.1%; $[\alpha]_D^{30}$ = -168.1 (c = 1.05, $CHCl_3$).

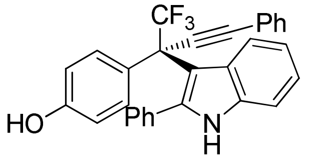

(*S*)-4-(1,1,1-Trifluoro-4-phenyl-2-(2-phenyl-1*H*-indol-3-yl)but-3-yn-2-yl)phenol (7i)

It was purified by silica gel chromatography using EtOAc/petroleum ether (1:4). Yield: 70% (32.6 mg).^{1}H NMR (500 MHz, $CDCl_3$) δ 8.07 (brs, 1H), 7.54 (d, *J* = 8.3 Hz, 1H), 7.43 (d, *J* = 8.5 Hz, 2H), 7.36 – 7.31 (m, 3H), 7.28 – 7.17 (m, 7H), 7.13 – 7.10 (m, 2H), 7.07 – 7.03 (m, 1H), 6.64 (d, *J* = 8.8 Hz, 2H), 5.03 (brs, 1H); ^{13}C NMR (126 MHz, $CDCl_3$) δ 154.9, 138.7, 135.1, 133.9, 131.9, 131.6, 130.6, 130.0, 128.5, 128.3, 128.0, 127.7, 127.1, 126.2 (q, *J* = 284.5 Hz), 122.6, 122.4, 122.3 (q, *J* = 2.2 Hz), 120.3, 115.1, 110.8, 109.2, 87.8 (q, *J* = 2.3 Hz), 87.6, 51.6 (q, *J* = 29.4 Hz); ^{19}F NMR (471 MHz, $CDCl_3$) δ -67.0; HRMS (ESI) m/z calculated for $C_{30}H_{19}F_3NO$ $[M - H]^-$ 466.1424, found 466.1431; HPLC: the ee value was determined by HPLC analysis (Chiralpak AD-H, *i*-PrOH/hexane = 20/80, 1.0 mL/min, 237 nm), retention time: t_{major} = 6.660 min, t_{minor} = 7.757 min, ee = 92.3%; $[\alpha]_D^{30}$ = -16.2 (c = 1.04, $CHCl_3$).

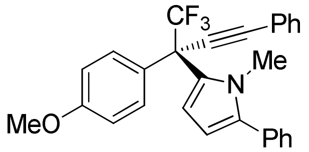

1-Methyl-2-phenyl-5-(1,1,1-trifluoro-2-(4-methoxyphenyl)-4-phenylbut-3-yn-2-yl)-1*H*-pyrrole (9')

9 was prepared using **3d** with 1.0 equiv of **8** without K_2CO_3 additive. The mixture was treated with NaH and MeI for purification due to the product instability issue. Yield: 65% (29.0 mg). ^{1}H NMR (500 MHz, $CDCl_3$) δ 7.58 – 7.54 (m, 2H), 7.51 (d, *J* = 8.6 Hz, 2H), 7.41 – 7.34 (m, 7H), 7.33 – 7.29 (m, 1H), 6.93 (d, *J* = 8.9 Hz, 2H), 6.56 – 6.53 (m, 1H), 6.28 (d, *J* = 3.8 Hz, 1H), 3.84 (s, 3H), 3.28 (s, 3H); ^{13}C NMR (126 MHz, $CDCl_3$) δ 160.1, 136.9, 133.4, 132.1, 130.4, 129.5, 129.1, 128.6, 128.5, 127.9, 127.3, 127.2, 125.1 (q, *J* = 284.1 Hz), 122.3, 114.0, 109.0 (q, *J* = 2.9 Hz), 107.8, 87.3, 84.7, 55.5, 52.4 (q, *J* = 28.9 Hz), 33.7; ^{19}F NMR (471 MHz, $CDCl_3$) δ -70.4; HRMS (ESI) m/z calculated for $C_{28}H_{23}F_3NO$ $[M + H]^+$ 446.1726, found 446.1739; HPLC: the ee value was determined by HPLC analysis (Chiralcel OD-H, *i*-PrOH/hexane = 0.5/99.5, 1.0 mL/min, 279 nm), retention time: t_{minor} =7.360 min, t_{major} = 8.460 min, ee = 93.1%; $[\alpha]_D^{30}$ = -13.3 (c = 0.92, $CHCl_3$).

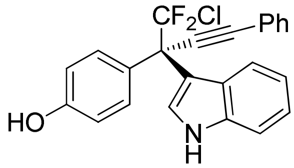

(*S*)-4-(1-Chloro-1,1-difluoro-2-(1*H*-indol-3-yl)-4-phenylbut-3-yn-2-yl)phenol (11a)

It was purified by silica gel chromatography using EtOAc/petroleum ether (1:4). Yield: 63% (25.8mg). ^{1}H NMR (500 MHz, $CDCl_3$) δ 8.16 (brs, 1H), 7.56 (d, *J* = 8.6 Hz, 2H), 7.51 – 7.44 (m, 3H), 7.41 – 7.28 (m, 5H), 7.20 – 7.14 (m, 1H), 7.02 – 6.96 (m, 1H), 6.75 (d, *J* = 8.8 Hz, 2H), 4.95 (brs, 1H); ^{13}C NMR (126 MHz, $CDCl_3$) δ 155.7, 136.4, 132.0, 131.2, 130.9 (t, *J* = 300.6 Hz),129.7, 128.8, 128.5, 126.0, 123.4 (t, *J* = 3.3 Hz), 122.7, 122.6, 121.4, 120.0, 115.0, 113.2, 111.3, 87.4 (t, *J* = 3.1 Hz), 87.2, 56.7 (t, *J* = 25.4 Hz); ^{19}F NMR (471 MHz, $CDCl_3$) δ -54.50 – -56.79 (m, 2F); HRMS (ESI) m/z calculated for $C_{24}H_{15}ClF_2NO$ $[M - H]^-$ 406.0816, found 406.0809; HPLC: the ee value was determined by HPLC analysis (Chiralpak IG, *i*-PrOH/hexane = 10/90, 1.0 mL/min, 254 nm), retention time: t_{minor} = 6.623 min, t_{major} = 15.933min, ee = 84.1%; $[\alpha]_D^{30}$ = -21.5 (c = 1.04, $CHCl_3$).

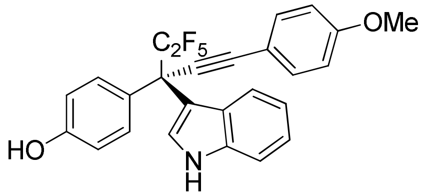

(*S*)-4-(4,4,5,5,5-Pentafluoro-3-(1*H*-indol-3-yl)-1-phenylpent-1-yn-3-yl)phenol (11b)

It was purified by silica gel chromatography using EtOAc/petroleum ether (1:4). Yield: 68% (32.1mg). ^{1}H NMR (500 MHz, $CDCl_3$) δ 8.13 (brs, 1H), 7.59 (d, *J* = 7.6 Hz, 2H), 7.45 – 7.31 (m, 5H), 7.18 – 7.12 (m, 1H), 7.00 – 6.94 (m, 1H), 6.83 (d, *J* = 8.9 Hz, 2H), 6.75 (d, *J* = 8.9 Hz, 2H), 5.12 (brs, 1H), 3.80 (s, 3H); ^{13}C NMR (126 MHz, $CDCl_3$) δ 160.1, 155.7, 136.5, 133.4, 130.8, 128.7, 125.9, 123.2 (t, *J* = 4.6 Hz), 122.5, 121.5, 119.9, 124.1 – 112.7 (m), 115.1, 114.8, 114.1, 113.1, 111.3, 88.1, 84.4 – 84.2 (m), 55.5, 50.2 – 49.7 (m); ^{19}F NMR (471 MHz, $CDCl_3$) δ -76.5 – -76.6 (m, 3F), -110.0 – -115.7 (m, 2F); HRMS (ESI) m/z calculated for $C_{26}H_{17}F_5NO_2$ $[M - H]^-$ 470.1185, found 470.1188; HPLC: the ee value was determined by HPLC analysis (Chiralpak IG, *i*-PrOH/hexane = 10/90, 1.0 mL/min, 256 nm), retention time: t_{minor} =7.460 min, t_{major} = 13.857min, ee = 91.1%; $[\alpha]_D^{30}$ = -88.3 (c = 1.04, $CHCl_3$).

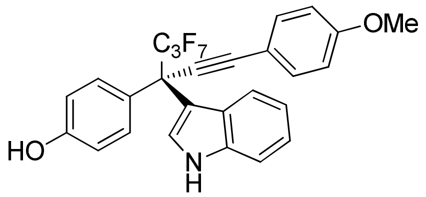

(*S*)-4-(4,4,5,5,6,6,6-Heptafluoro-3-(1*H*-indol-3-yl)-1-(4-methoxyphenyl)hex-1-yn-3-yl)phenol (11c)

It was purified by silica gel chromatography using EtOAc/petroleum ether (1:4). Yield: 66% (34.3 mg). ^{1}H NMR (500 MHz, $CDCl_3$) δ 8.07 (brs, 1H), 7.62 (d, *J* = 8.4 Hz, 2H), 7.49 (d, *J* = 8.1 Hz, 1H), 7.40 (d, *J* = 8.7 Hz, 2H), 7.33 (s, 1H), 7.30 (d, *J* = 8.2 Hz, 1H), 7.18 – 7.14 (m, 1H), 7.03 – 6.98 (m, 1H), 6.84 (d, *J* = 8.7 Hz, 2H), 6.74 (d, *J* = 8.8 Hz, 2H), 5.21 (brs, 1H), 3.80 (s, 3H); ^{13}C NMR (126 MHz, $CDCl_3$) δ 160.0, 155.6, 136.5, 133.4, 131.0, 128.5, 125.8, 123.3, 122.5, 121.5, 119.9, 122.9 – 107.5 (m), 115.1, 114.8, 114.2, 113.1, 111.4, 88.2, 84.3 – 84.2 (m), 55.5, 50.9 (t, *J* = 23.7 Hz); ^{19}F NMR (471 MHz, $CDCl_3$) δ -80.9 – -81.0 (m, 3F), -106.2 – -109.8 (m, 2F), -117.8 – -121.3 (m, 2F); HRMS (ESI) m/z calculated for $C_{27}H_{17}F_7NO_2$ $[M - H]^-$ 520.1153 found 520.1149; HPLC: the ee

value was determined by HPLC analysis (Chiralpak IG, *i*-PrOH/hexane = 10/90, 1.0 mL/min, 253 nm), retention time: t_{minor} = 5.647 min, t_{major} = 8.487 min, ee = 93.0%; $[\alpha]_D^{30}$ = -56.6 (c = 1.01, $CHCl_3$).

Absolute configuration determination

Absolute configuration determination of 4a

The absolute configuration of **4a** was determined by preparing the same product **B** from the reported optical pure substrate (*S*)-**A** and **4a** respectively.[5-6]

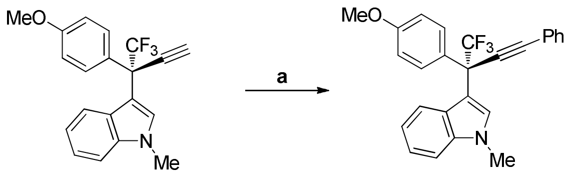

(*S*)-A (reported) (*R*)-B

Regent and condition: (a) iodobenzene (1.1 equiv), $PdCl_2(PPh_3)_2$ (0.02 equiv), CuI (0.015mml, 0.05 equiv), THF/Et_3N (1 mL/ 1 mL), N_2, 60 °C, 12 h.

(*R*)-1-Methyl-3-(1,1,1-trifluoro-2-(4-methoxyphenyl)-4-phenylbut-3-yn-2-yl)-1*H*-indole (B)

Prepared starting from (*S*)-**A** according to procedure **a**. ^{1}H NMR (500 MHz, $CDCl_3$) δ 7.59 (d, *J* = 8.8 Hz, 2H), 7.50 – 7.45 (m, 2H), 7.35 – 7.27 (m, 5H), 7.26 – 7.24 (m, 1H), 7.22 – 7.18 (m, 1H), 6.99 – 6.95 (m, 1H), 6.85 (d, *J* = 8.9 Hz, 2H), 3.84 (s, 3H), 3.79 (s, 3H); ^{13}C NMR (126 MHz, $CDCl_3$) δ 159.7, 137.5, 132.1, 130.4, 129.0, 128.7, 128.4, 127.7 (q, *J* = 2.6 Hz), 126.3, 125.9 (q, *J* = 284.4 Hz), 122.8, 122.1, 121.4, 119.6, 113.6, 110.9, 109.5, 86.5 (q, *J* = 1.2 Hz), 86.4, 55.4, 51.2 (q, *J* = 29.1 Hz), 33.2; ^{19}F NMR (471 MHz, $CDCl_3$) δ -70.6; HRMS (ESI) m/z calculated for $C_{26}H_{21}F_3NO$ $[M+H]^+$ 420.1570, found 420.1566; HPLC: the ee value was determined by HPLC analysis (Chiralpak AD-H, i-PrOH/hexane = 2/98, 1.0 mL/min, 215nm), retention time: t_{major} = 6.753 min, t_{minor} = 10.740 min, ee = 88.8%; $[\alpha]_D^{30}$ = +98.3 (c = 0.50, $CHCl_3$).

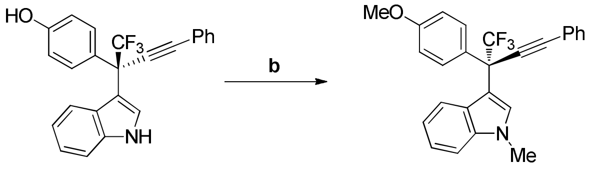

(*S*)-4a (*S*)-B

Regent and condition: (**b**) MeI (5.0 equiv), NaH (2.5 equiv), THF.

Prepared starting from **4a** according to procedure **b.** ^{1}H NMR, ^{13}C NMR and ^{19}F NMR were the same as the (*R*)-**B** above. HPLC: the ee value was determined by HPLC analysis (Chiralpak AD-H, *i*-PrOH/hexane = 2/98, 1.0 mL/min, 215 nm), retention time: t_{minor} = 6.353 min, t_{major} = 9.693 min, ee = 94.4%; $[\alpha]_D^{30}$ = -120.2 (c = 0.22, $CHCl_3$). The results of HPLC and $[\alpha]_D$ were opposite to (*R*)-**B**, which allowed the assignment of the absolute configuration of **4a** as *S* according to the known literature.

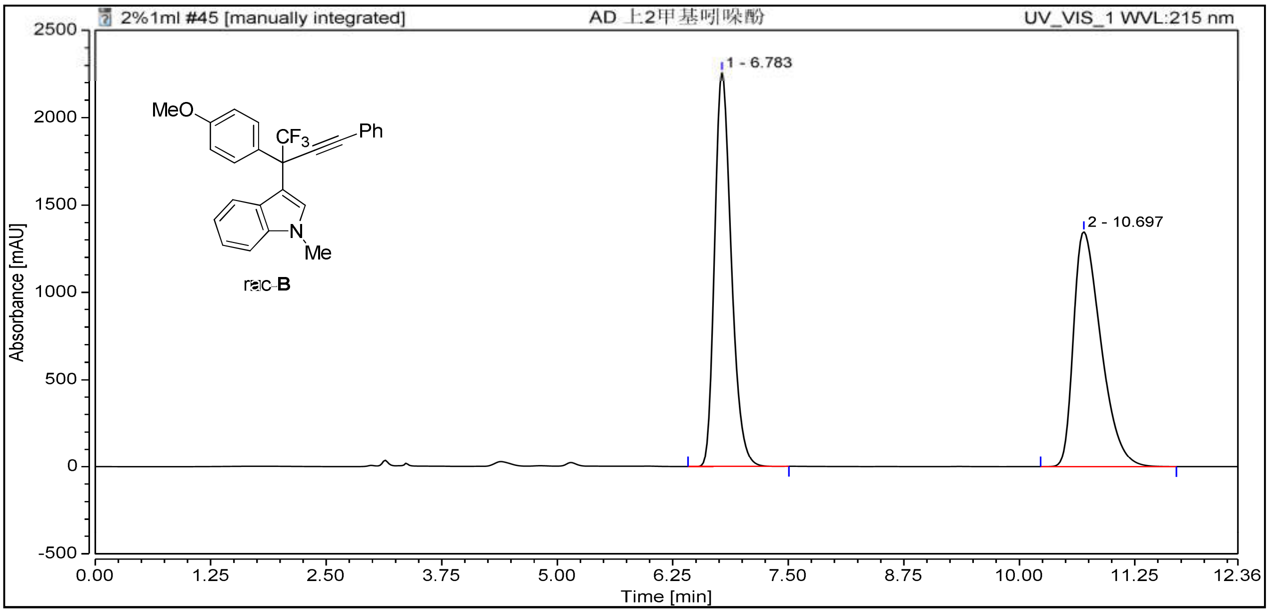

Integration Results					
No.	Peak Name	Retention Time min	Area mAU*min	Relative Area %	Amount n.a.
1		6.783	472.765	50.48	n.a.
2		10.697	463.867	49.52	n.a.
**Total:**			**936.632**	**100.00**	

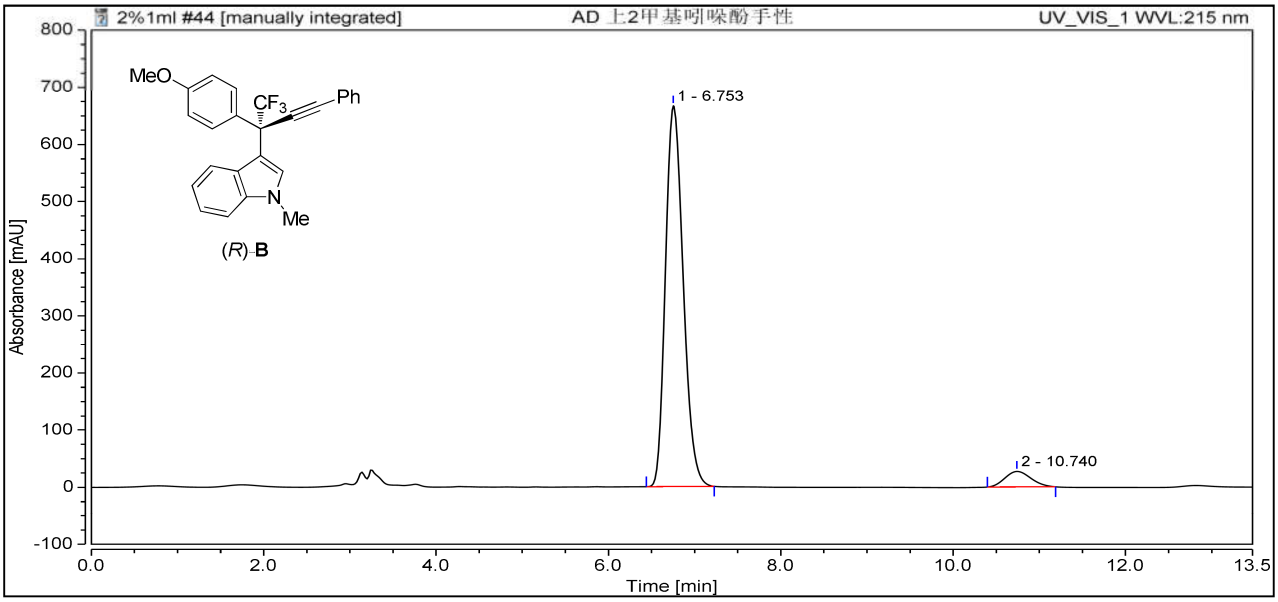

Integration Results					
No.	Peak Name	Retention Time<br>min	Area<br>mAU*min	Relative Area<br>%	Amount<br>n.a.
1		6.753	158.969	94.41	n.a.
2		10.740	9.409	5.59	n.a.
**Total:**			**168.378**	**100.00**	

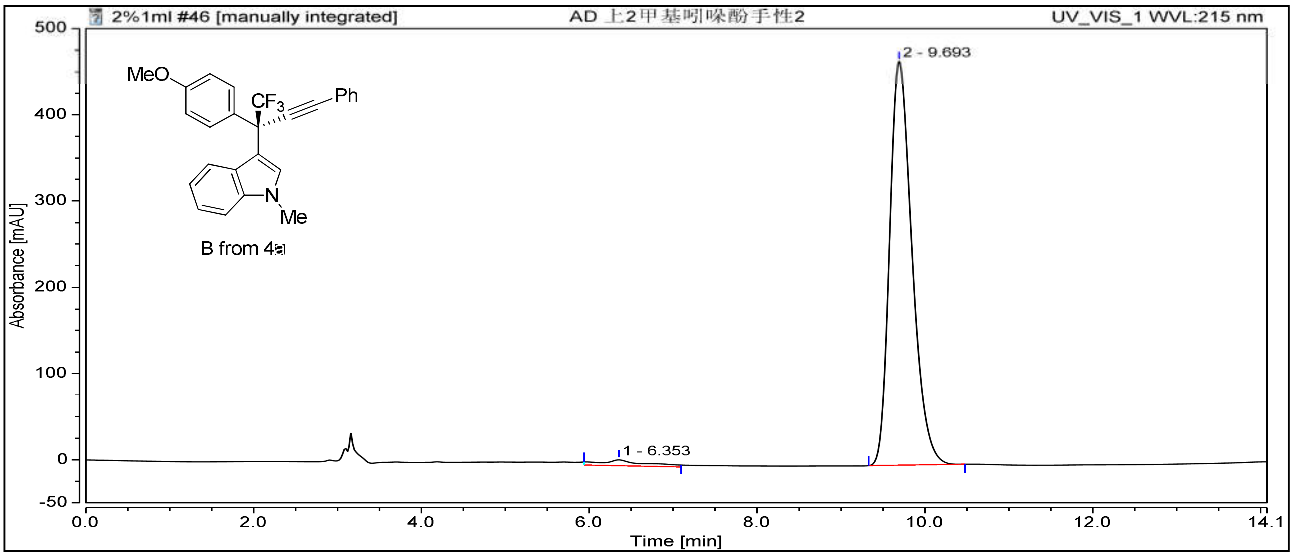

Integration Results					
No.	Peak Name	Retention Time<br>min	Area<br>mAU*min	Relative Area<br>%	Amount<br>n.a.
1		6.353	4.201	2.82	n.a.
2		9.693	144.613	97.18	n.a.
**Total:**			**148.814**	**100.00**	

Absolute configuration determination of 6a

The absolute configuration of **6a** was determined to be *S* on the basis of UV and circular dichroism (CD) analyses. The simulated electrostatic circular dichroism (ECD) curve of (*S*)-**6a** and (*R*)-**6a** through DFT calculation were correspondingly expressed as the red curve **6a$_S$** and the blue curve **6a$_R$**. The experiment ECD curve of **6a** through handling the experimental data from UV and CD spectra was expressed as the black curve **6a**. According to the figure shown below, the trend of the experiment ECD curve of **6a** was consistent with the simulated ECD curve of **6a$_S$**, which allowed the assignment of the absolute configuration of **6a** as *S*.

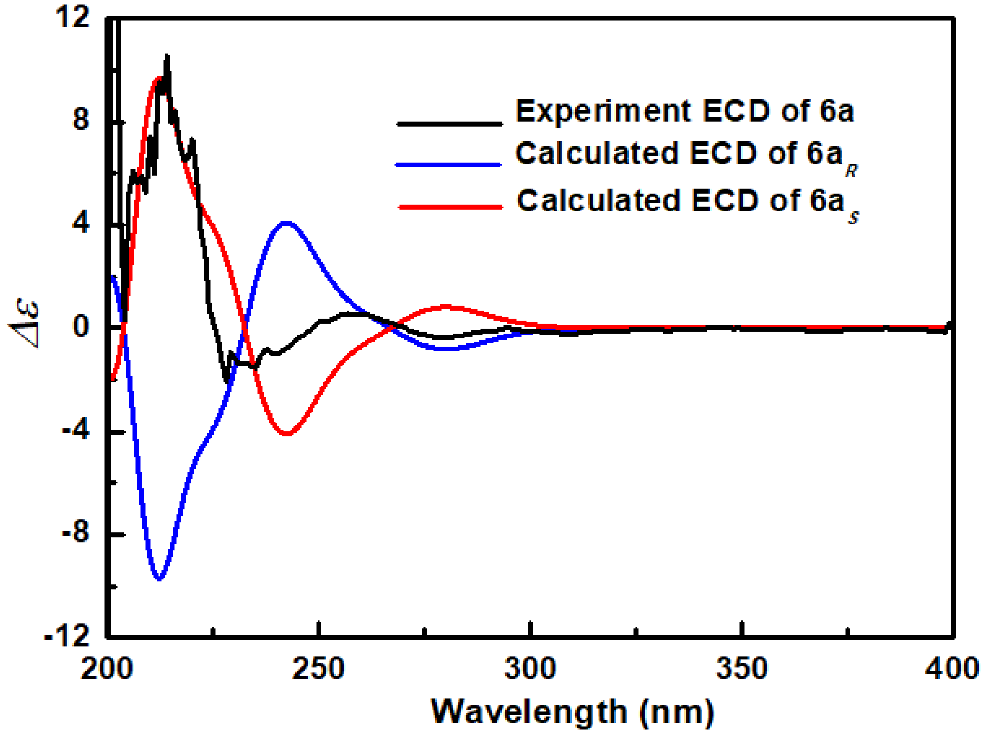

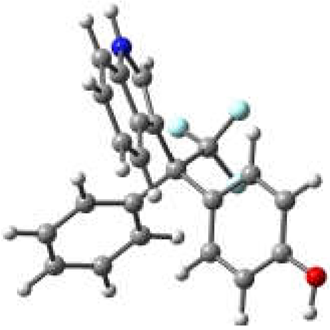

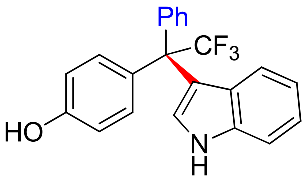

6a

(*S*)-4-(2,2,2-trifluoro-1-(1*H*-indol-3-yl)-1-phenylethyl)phenol

Computational methods:

The calculations were conducted using density functional theory (DFT) as implemented in the gaussian 09.[7] Geometry optimization and frequency calculation were carried out at the B3LYP/6-31G* level. Electronic excitation energies and rotational strengths in methanol were calculated using TDDFT at cam-B3LYP/PCM-6-31+G(d,p) level in velocity formalism for the first 50 states. The CD curves were simulated by using the Gaussian function:[8]

$$\Delta\varepsilon(E) = \frac{1}{2.296\times10^{-39}}\frac{1}{\sigma\sqrt{\pi}}\times\sum_i \Delta E_i R_i e^{-[(E-\Delta E_i)/\sigma]^2}$$

where σ is the width of the band at 1/*e* height and ΔEi and *Ri* are the excitation energies and rotatory strengths for transition *i*, respectively. Here a value of $\sigma = 0.6$ eV.

Synthetic application

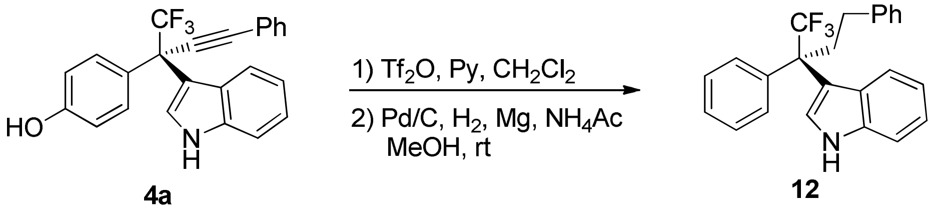

(*R*)-3-(1,1,1-Trifluoro-2,4-diphenylbutan-2-yl)-1*H*-indole (12)

To a solution of **4a** (0.1 mmol, 1.0 equiv) in CH_2Cl_2 (5 mL) were added pyridine (0.2 mmol, 2.0 equiv) and a solution of Tf_2O (0.115 mmol, 1.15 equiv) in CH_2Cl_2 (0.5 mL) sequentially. The reaction mixture was stirred for 2 h. Then water (10 mL) was added. The layers were separated, and the aqueous layer was extracted with ethyl acetate (10 mL×3). The combined organic layers were washed with brine (10 mL), dried over anhydrous Na_2SO_4, filtered, and concentrated. The residue was purified by flash chromatography on silica gel using EtOAc/petroleum ether (1:10) as eluent. Then a mixture of the product, 10% Pd/C (9.3 mg, 10 wt %), NH_4OAc (15.4 mg), and Mg (12.0 mg) in methanol (5.0 mL) was stirred at room temperature under hydrogen balloon for 12 h. The mixture was passed through a short length of silica gel using EtOAc/petroleum ether (1:10) as eluent to afford **12** (32.6 mg, 86% yield). ^1H NMR (500 MHz, $CDCl_3$) δ 8.15 (brs, 1H), 7.44 – 7.41 (m, 2H), 7.39 – 7.35 (m, 2H), 7.30 – 7.26 (m, 3H), 7.24 – 7.20 (m, 2H), 7.17 – 7.13 (m, 2H), 7.03 – 7.01 (m, 2H), 6.91 – 6.87 (m, 1H), 6.81 – 6.78 (m, 1H), 2.88 – 2.76 (m, 2H), 2.64 – 2.55 (m, 1H), 2.38 – 2.29 (m, 1H); ^{13}C NMR (126 MHz, $CDCl_3$) δ 142.4, 139.1, 136.6, 129.3, 128.6, 128.6, 128.3 (q, *J* = 285.1 Hz), 128.2, 127.8, 126.1, 124.3 (q, *J* = 2.2 Hz), 122.4, 121.6, 119.9, 114.0, 111.4, 53.8 (q, *J* = 24.5 Hz), 38.3, 31.8; ^{19}F NMR (471 MHz, $CDCl_3$) δ -68.1; HRMS (EI) m/z calculated for $C_{24}H_{21}F_3N$ $[M + H]^+$: 380.1621, found 380.1623; HPLC: the ee value was determined by HPLC analysis (Chiralpak IG, i-PrOH/hexane = 5/95, 1 mL/min, 215 nm), retention time: t_{major} = 9.737 min, t_{minor}= 11.590 min, ee = 93.4%; $[\alpha]_D^{30}$ = +8.5 (c = 0.96, $CHCl_3$).

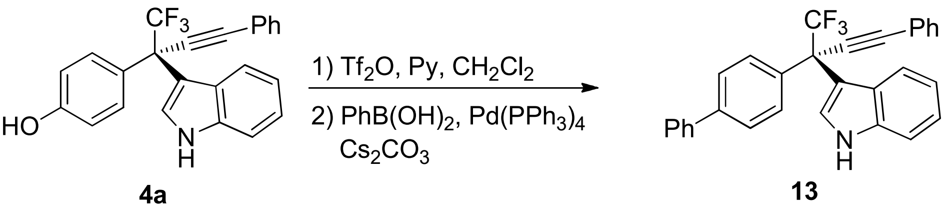

(*S*)-3-(2-([1,1'-Biphenyl]-4-yl)-1,1,1-trifluoro-4-phenylbut-3-yn-2-yl)-1*H*-indole (13)

To a solution of **4a** (0.1 mmol, 1.0 equiv) in CH_2Cl_2 (5 mL) were added pyridine (0.2 mmol, 2.0 equiv) and a solution of Tf_2O (0.115 mmol, 1.15 equiv) in CH_2Cl_2 (0.5 mL) sequentially. The reaction mixture was stirred for 2 h. Then water (10 mL) was added. The layers were separated, and the aqueous layer was extracted with ethyl acetate (10 mL×3). The combined organic layers were washed with brine (10 mL), dried over anhydrous Na_2SO_4, filtered, and concentrated. The residue was purified by flash chromatography on silica gel using EtOAc/petroleum ether (1:10) as eluent. Then to a solution of the product, phenylboronic acid (0.15 mmol, 1.5 equiv), Cs_2CO_3 (97.7 mg, 0.3 mmol, 3 equiv) in dioxane (1 mL) was added $Pd(PPh_3)_4$ (17.3 mg, 0.015 mmol, 0.15 equiv) and degassed with nitrogen. The reaction mixture was stirred at 60 °C for 48 h and then filtered through a short pad of celite, which was washed with CH_2Cl_2 (10 mL). The filtrate was concentrated and the residue was purified by flash chromatography on silica gel using EtOAc/petroleum ether (1:50) as eluent to afford **13** (38.9 mg, 86% yield). ^{1}H NMR (500 MHz, CD_3OD) δ 7.71 (d, *J* = 8.3 Hz, 2H), 7.61 – 7.56 (m, 4H), 7.47 – 7.44 (m, 3H), 7.41 – 7.37 (m, 3H), 7.35 – 7.28 (m, 4H), 7.26 (d, *J* = 8.1 Hz, 1H), 7.10 – 7.06 (m, 1H), 6.88 – 6.84 (m, 1H); ^{13}C NMR (126 MHz, CD_3OD) δ 142.5, 141.6, 138.3, 137.3, 132.7, 130.6, 129.9, 129.9, 129.6, 128.6, 128.1, 127.7, 127.1 (q, *J* = 283.3 Hz), 126.7, 124.5 (q, *J* = 2.3 Hz), 123.6, 122.9, 121.5, 120.1, 112.5, 112.0, 87.5, 87.2 (q, *J* = 1.8 Hz), 52.9 (q, *J* = 28.9 Hz); ^{19}F NMR (471 MHz, CD_3OD) δ -71.4; HRMS (EI) m/z calculated for $C_{30}H_{19}F_3N$ $[M - H]^-$: 450.1475, found 450.1483; HPLC: the ee value was determined by HPLC analysis (Chiralpak IG, i-PrOH/hexane = 5/95, 1 mL/min, 215 nm), retention time: t_{minor} = 8.923 min, t_{major}= 10.400 min, ee = 93.2%; $[\alpha]_D^{30}$ = -54.6 (c = 0.99, $CHCl_3$).

Mechanistic studies

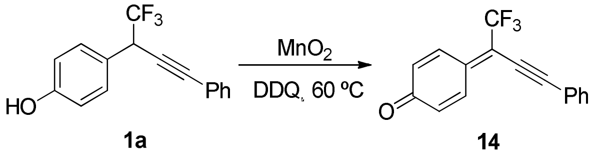

1a **14**

4-(1,1,1-Trifluoro-4-phenylbut-3-yn-2-ylidene)cyclohexa-2,5-dienone (14)

A solution of **1a** (0.1 mmol, 1.0 equiv), MnO_2 (0.5 mmol, 5.0 equiv) and DDQ (0.025 mmol, 0.25 equiv) in CH_2Cl_2 (1.0 mL) was stirred at 60 °C for 8 h. Then it was purified directly by silica gel chromatography using ethyl acetate/petroleum ether 1:9). Yield: 11% (2.9 mg). ^{1}H NMR (500 MHz, C_6D_6) δ 7.37 (dd, J = 10.0, 2.6 Hz, 1H), 7.24 – 7.20 (m, 2H), 7.20 – 7.17 (m, 1H), 7.00 – 6.93 (m, 1H), 6.93 – 6.87 (m, J = 7.6 Hz, 2H), 6.23 (dd, J = 10.0, 1.6 Hz, 1H), 6.08 (dd, J = 10.2, 1.9 Hz, 1H); ^{13}C NMR (126 MHz, C_6D_6) δ 185.6, 139.1, 136.1, 132.2, 131.9 (q, J = 2.7 Hz), 131.5, 130.6, 130.5, 128.9, 121.9 (q, J = 275.7 Hz), 121.5, 121.4 (q, J = 34.1 Hz), 110.1, 84.4 (q, J = 3.7 Hz); ^{19}F NMR (471 MHz, C_6D_6) δ -55.8; HRMS (ESI) m/z calculated for $C_{16}H_{10}F_3O$ $[M + H]^+$ 275.0678, found 275.0673.

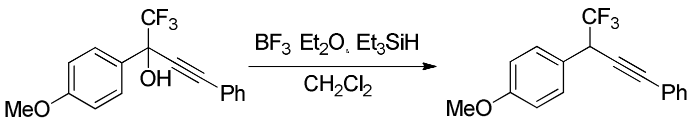

15' **15**

1-Methoxy-4-(1,1,1-trifluoro-4-phenylbut-3-yn-2-yl)benzene (15)

To a solution of **15'** (0.5 mmol, 1.0 equiv) was added Et_3SiH (1.0 mmol, 2.0 equiv) and $BF_3{\cdot}Et_2O$ (0.75 mmol, 1.5 equiv) in CH_2Cl_2 (5.0 mL) at -40 °C. Then it was warmed to room temperature. Upon starting material consumption monitored by TLC, the mixture was quenched by a saturated aqueous $NaHCO_3$ solution. The organic layer was extracted with CH_2Cl_2 and the combined organic layers were washed with saturated aqueous NaCl solution, dried over anhydrous $MgSO_4$, filtered and removed under vacuum. The residue was purified by a column chromatography on silica gel using petroleum ether as eluent to give the desired product **15** (68% yield). ^{1}H NMR (500 MHz, $CDCl_3$) δ 7.56 – 7.44 (m, 4H), 7.40 – 7.32 (m, 3H), 6.95 (d, J = 8.8 Hz, 2H), 4.53 (q, J = 8.0 Hz, 1H), 3.83 (s, 3H); ^{13}C NMR (126 MHz, $CDCl_3$) δ 160.2, 132.1, 130.7, 129.0, 128.5, 124.6 (q, J = 280.4 Hz), 124.1, 122.4, 114.3, 85.7, 82.1 (q, J = 3.3 Hz), 55.5, 43.5 (q, J = 31.6 Hz); ^{19}F NMR (471 MHz, $CDCl_3$) δ -70.8; HRMS (ESI) m/z calculated for $C_{17}H_{14}F_3O$ $[M + H]^+$ 291.0991, found 291.0987.

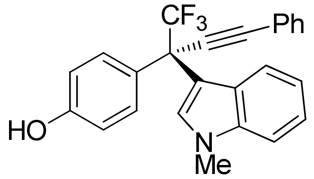

(*S*)-4-(1,1,1-Trifluoro-2-(1-methyl-1*H*-indol-3-yl)-4-phenylbut-3-yn-2-yl)phenol (20)

It was prepared according to the general procedure and purified by a column chromatography on silica gel using CH_2Cl_2. Yield: 60% (24.4 mg) ^1H NMR (500 MHz, $CDCl_3$) δ 7.57 (d, *J* = 8.6 Hz, 2H), 7.53 – 7.47 (m, 2H), 7.38 (d, *J* = 8.1 Hz, 1H), 7.35 – 7.29 (m, 4H), 7.27 (m, 1H), 7.25 – 7.20 (m, 1H), 7.05 – 6.98 (m, 1H), 7.04 – 6.99 (m, 1H), 6.77 (d, *J* = 8.8 Hz, 2H), 3.82 (s, 3H); ^{13}C NMR (126 MHz, $CDCl_3$) δ 155.7, 137.4, 132.1, 130.6, 129.2, 128.8, 128.4, 127.7 (q, *J* = 2.4 Hz), 126.2, 125.8 (q, *J* = 284.3 Hz), 122.7, 122.2, 121.3, 119.6, 115.1, 110.8, 109.5, 86.4, 51.2 (q, *J* = 29.5 Hz), 33.1; ^{19}F NMR (471 MHz, $CDCl_3$) δ -70.5; HRMS (ESI) m/z calculated for $C_{25}H_{17}F_3NO$ $[M - H]^-$ 404.1268, found 404.1275; HPLC: the ee value was determined by HPLC analysis (Chiralpak AD-H, i-PrOH/hexane = 20/80, 1 mL/min, 243 nm), retention time: t_{minor} = 4.707 min, t_{major}= 6.463 min, ee = 5.1%.

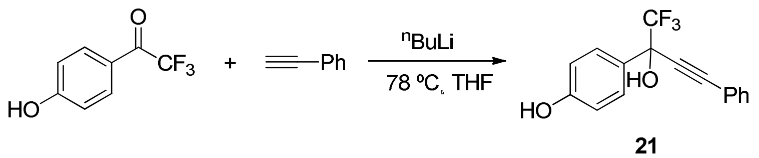

4-(1,1,1-Trifluoro-2-hydroxy-4-phenylbut-3-yn-2-yl)phenol (21)

To a solution of alkyne (2 mmol, 2.0 equiv) in anhydrous THF at -78 °C under N_2 was added ^nBuLi (2 mmol, 0.8 mL, 2.5 M in hexane, 2.0 equiv) dropwise. The reaction was stirred at the same temperature for 1 h. Then a solution of ketone (1 mmol, 1.0 equiv) in anhydrous THF (5 mL) was added to the mixture dropwise. The reaction was kepted at -78°C for 15 min and then warmed up to room temperature slowly overnight. Upon completion, the mixture was quenched by a saturated aqueous NH_4Cl solution. The organic layer was extracted with ethyl acetate and the combined organic layers were washed with saturated aqueous NaCl solution, dried over anhydrous $MgSO_4$, filtered and removed under vacuum. The residue was purified by a column chromatography on silica gel using ethyl acetate/petroleum ether as eluent to give the desired product **21** (70% yield). ^1H NMR (500 MHz, $CDCl_3$) δ 7.69 (d, *J* = 8.6 Hz, 2H), 7.54 – 7.51 (m, 2H), 7.43 – 7.33 (m, 3H), 6.88 (d, *J* =

8.8 Hz, 2H), 5.66 (brs, 1H), 3.50 (brs, 1H); ^{13}C NMR (126 MHz, $CDCl_3$) δ 156.6, 132.2, 129.7, 129.1, 128.7, 127.8, 123.6 (q, J = 285.5 Hz), 121.1, 115.4, 88.3, 84.6, 73.4 (q, J = 32.6 Hz); HRMS (ESI) m/z calculated for $C_{16}H_{10}F_3O_2$ $[M - H]^-$ 291.0638, found 291.0645.

References

1. Chen, M.; Sun, J. W. *Angew. Chem. Int. Ed.* **2017**, *56*, 11966.

2. Chen, D. D.; Chen, W. M.; Feng, S.; Gao, L.; Shen, H.; Tan, X. F.; Wang, L. *PCT Int. Appl.* **2017**, 2017202798.

3. Kelly, C. B.; Mercadante, M. A.; Hamlin, T. A.; Fletcher, M. H.; Leadbeater, N. E. *J. Org. Chem.* **2012**, *77*, 8131.

4. Sumi, T.; Goseki, R.; Otsuka, H. *Chem. Commun*., 2017, **53**, 11885.

5. Tsuchida, K.; Senda, Y.; Nakajima, K.; Nishibayashi, Y. *Angew. Chem. Int. Ed.* **2013**, *52*, 5146.

6. Sun, L.; Liu, P.; Wang, J.; Lu, P.; Wang, Y. G. *J. Org. Chem.* **2017**, *82*, 8407.

7. Frisch, M. J.; Trucks, G. W.; Schlegel, H. B.; Scuseria, G. E.; Robb, M. A.; Cheeseman, J. R.; Montgomery, J. A., Jr.; Vreven, T.; Kudin, K. N.; Burant, J. C.; Millam, J. M.; Iyengar, S. S.; Tomasi, J.; Barone, V.; Mennucci, B.; Cossi, M.; Scalmani, G.; Rega, N.; Petersson, G. A.; Nakatsuji, H.; Hada, M.; Ehara, M.; Toyota, K.; Fukuda, R.; Hasegawa, J.; Ishida, M.; Nakajima, T.; Honda, Y.; Kitao, O.; Nakai, H.; Klene, M.; Li, X.; Knox, J. E.; Hratchian, H. P.; Cross, J. B.; Bakken, V.; Adamo, C.; Jaramillo, J.; Gomperts, R.; Stratmann, R. E.; Yazyev, O.; Austin, A. J.; Cammi, R.; Pomelli, C.; Ochterski, J. W.; Ayala, P. Y.; Morokuma, K.; Voth, G. A.; Salvador, P.; Dannenberg, J. J.; Zakrzewski, V. G.; Dapprich, S.; Daniels, A. D.; Strain, M. C.; Farkas, O.; Malick, D. K.; Rabuck, A. D.; Raghavachari, K.; Foresman, J. B.; Ortiz, J. V.; Cui, Q.; Baboul, A. G.; Clifford, S.; Cioslowski, J.; Stefanov, B. B.; Liu, G.; Liashenko, A.; Piskorz, P.; Komaromi, I.; Martin, R. L.; Fox, D. J.; Keith, T.; Al-Laham, M. A.; Peng, C. Y.; Nanayakkara, A.; Challacombe, M.; Gill, P. M. W.; Johnson, B.; Chen, W.; Wong, M. W.; Gonzalez, C.; Pople, J. A. Gaussian03, revision A.1; Gaussian, Inc.: Pittsburgh, PA, 2004.

8. Philip J. S.; Nobuyuki H. *Chirality*, **2010**, *22*, 229.

NMR spectra

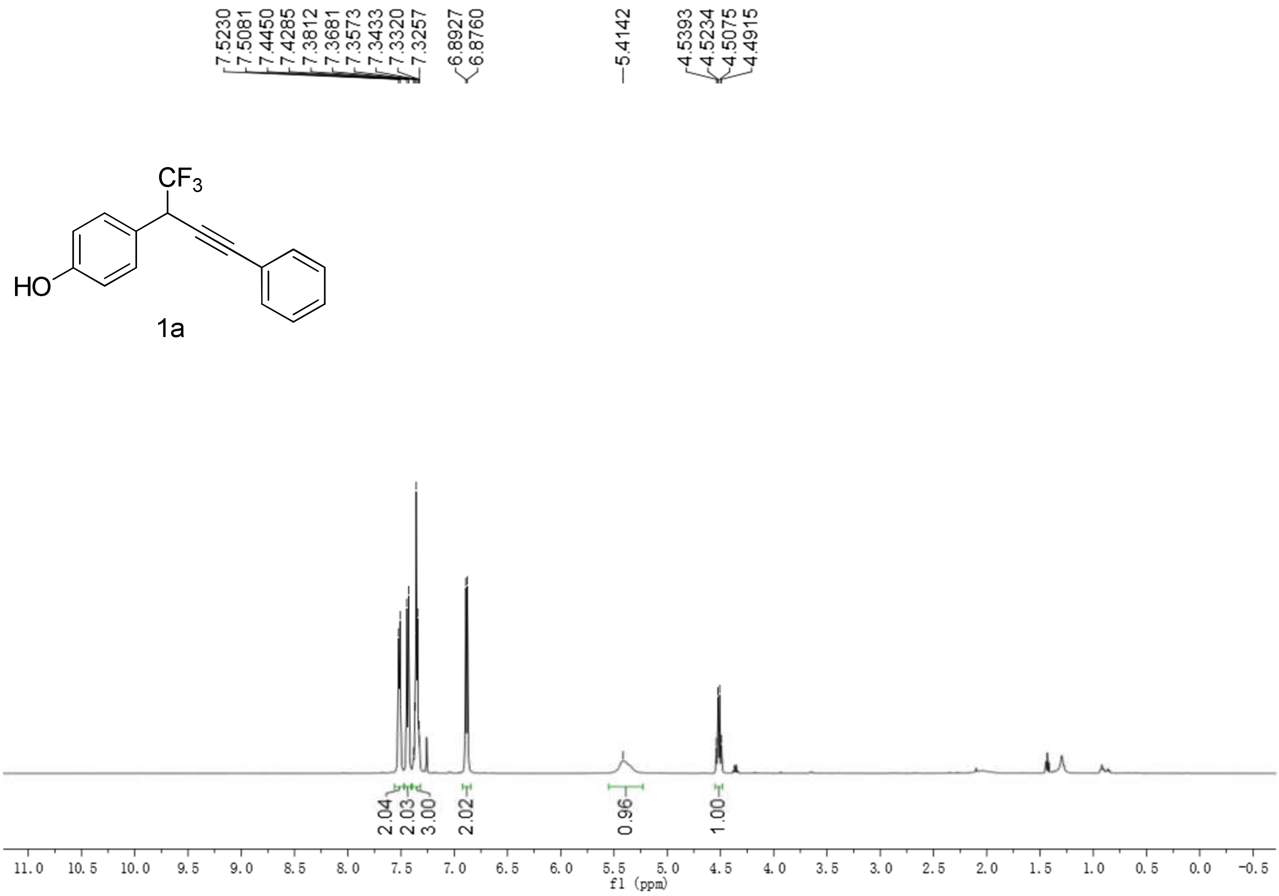
7.5230
7.5081
7.4450
7.4285
7.3812
7.3681
7.3573
7.3433
7.3320
7.3257
6.8927
6.8760
5.4142
4.5393
4.5234
4.5075
4.4915
CF3
HO
1a
2.04
2.03
3.00
2.02
0.96
1.00
f1 (ppm)

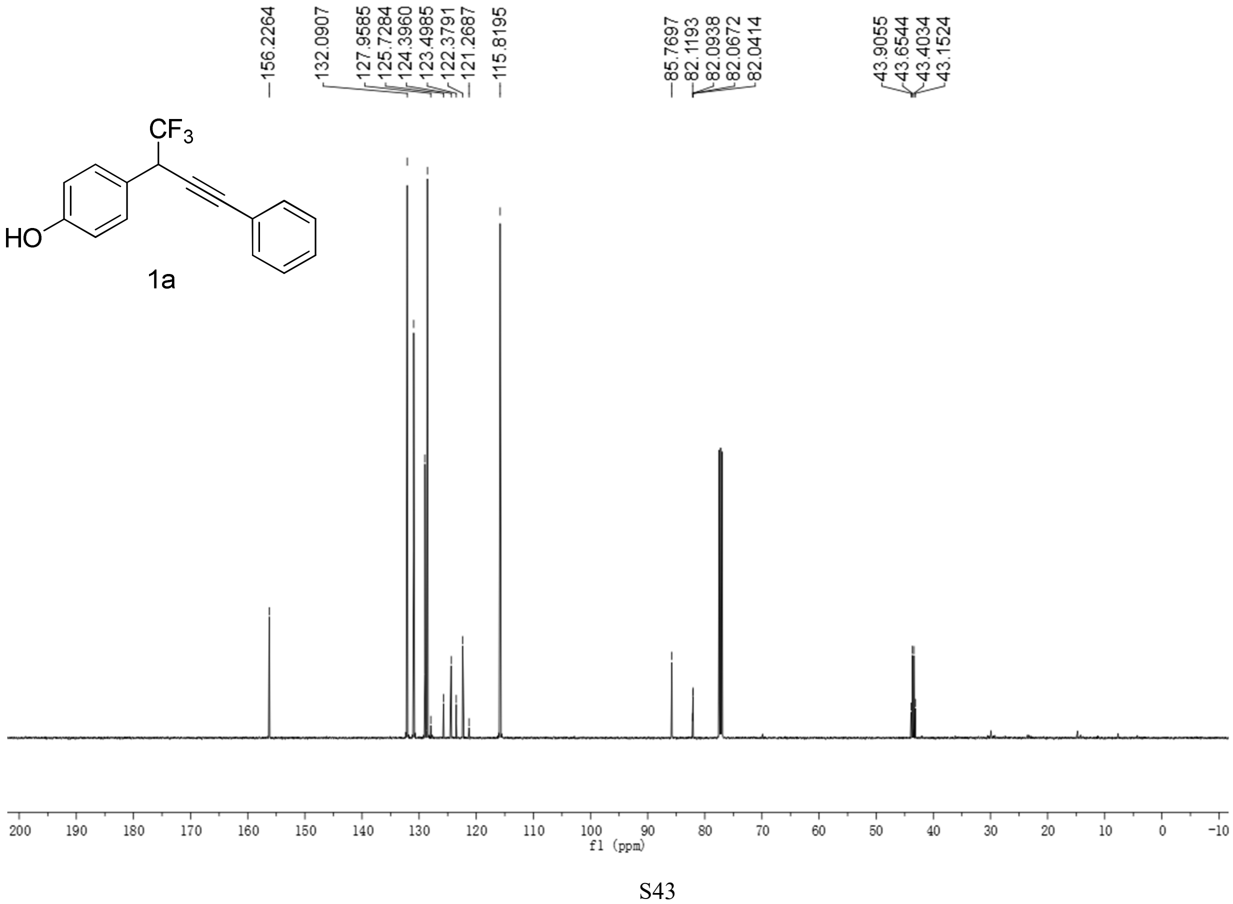
156.2264
132.0907
127.9585
125.7284
124.3960
123.4985
122.3791
121.2687
115.8195
85.7697
82.1193
82.0938
82.0672
82.0414
43.9055
43.6544
43.4034
43.1524
CF3
HO
1a
f1 (ppm)

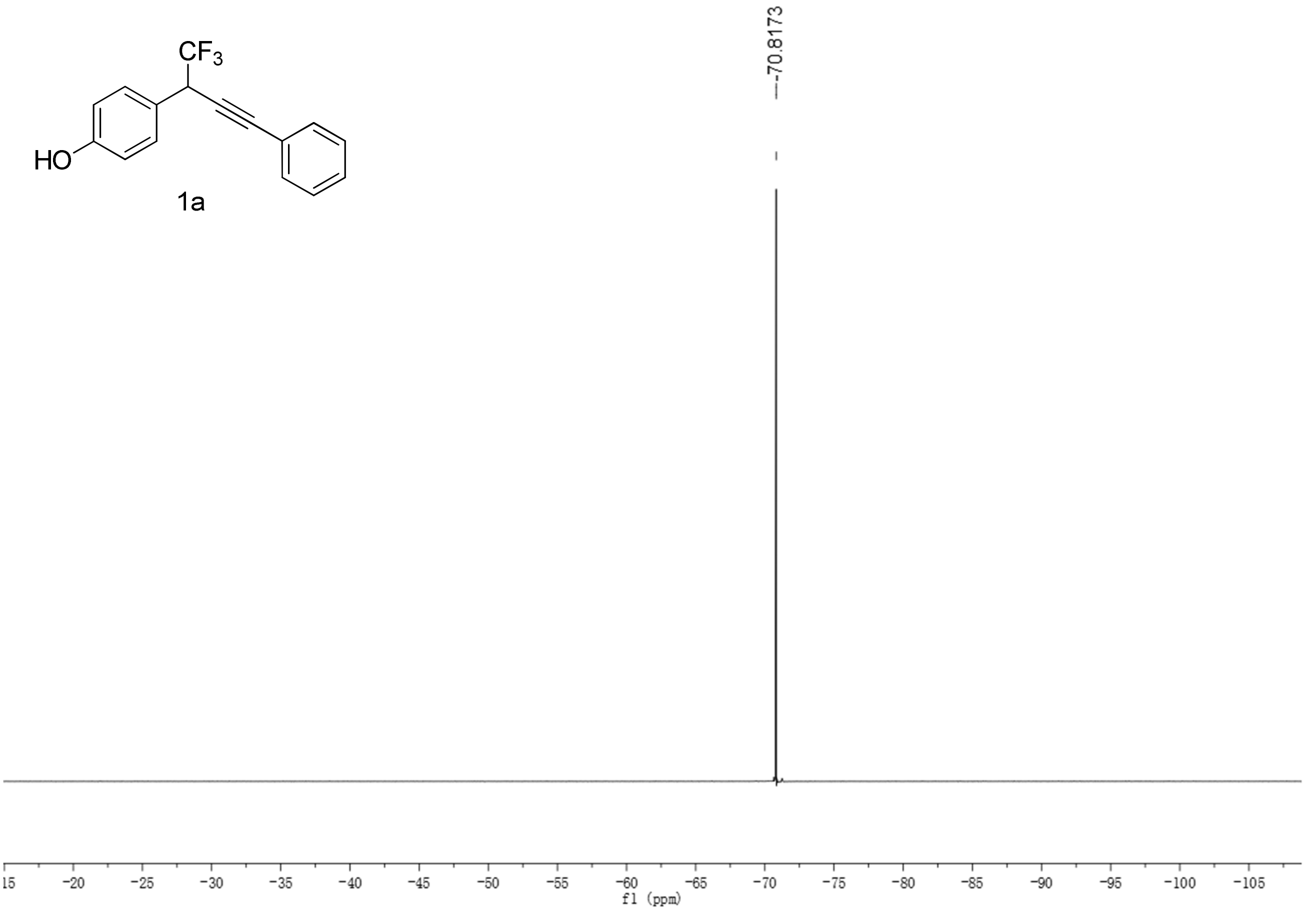
CF3
HO
1a
-70.8173
15 -20 -25 -30 -35 -40 -45 -50 -55 -60 -65 -70 -75 -80 -85 -90 -95 -100 -105
f1 (ppm)

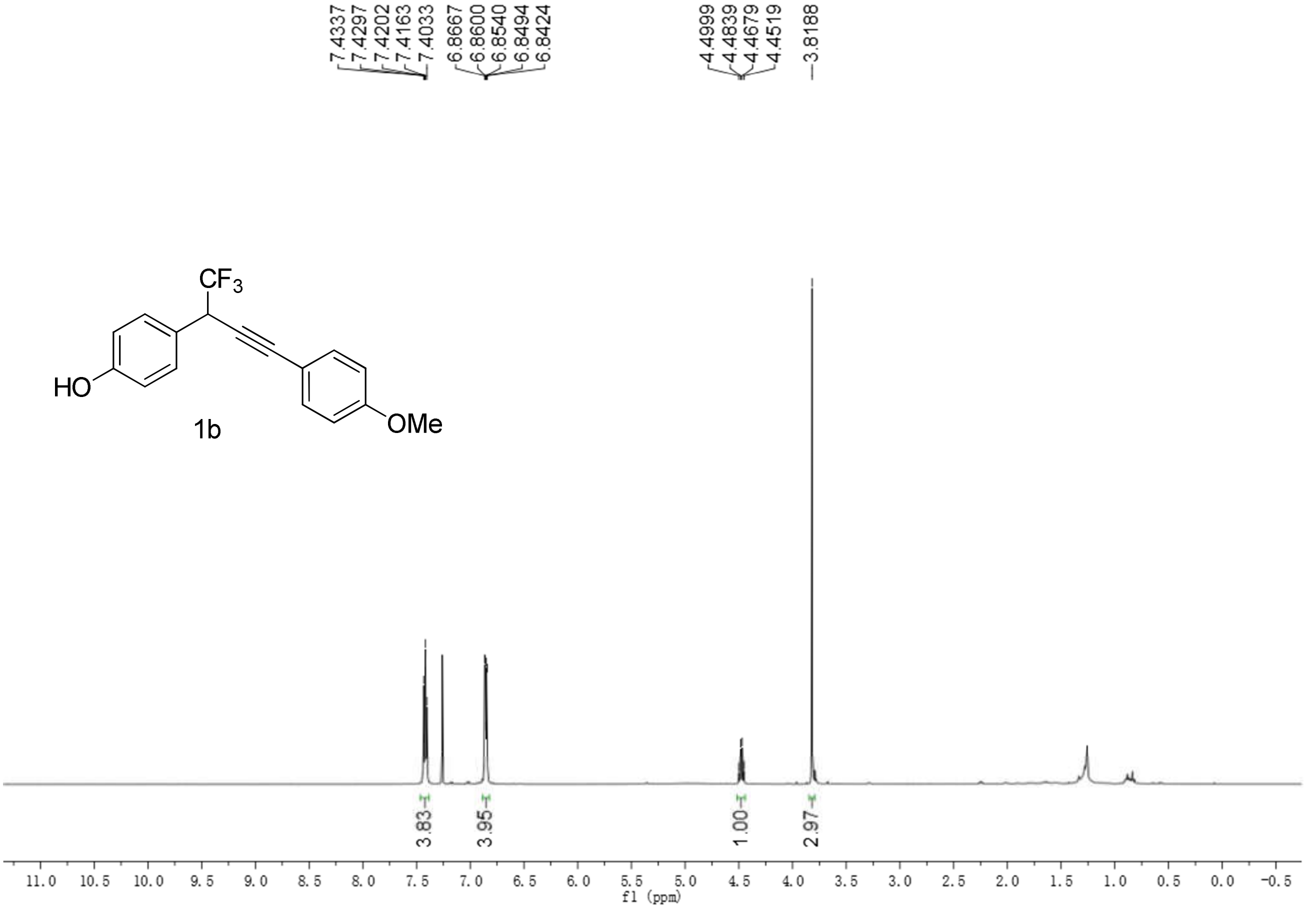
7.4337
7.4297
7.4202
7.4163
7.4033
6.8667
6.8600
6.8540
6.8494
6.8424
4.4999
4.4839
4.4679
4.4519
3.8188
CF3
HO
1b
OMe
3.83
3.95
1.00
2.97
11.0 10.5 10.0 9.5 9.0 8.5 8.0 7.5 7.0 6.5 6.0 5.5 5.0 4.5 4.0 3.5 3.0 2.5 2.0 1.5 1.0 0.5 0.0 -0.5
f1 (ppm)

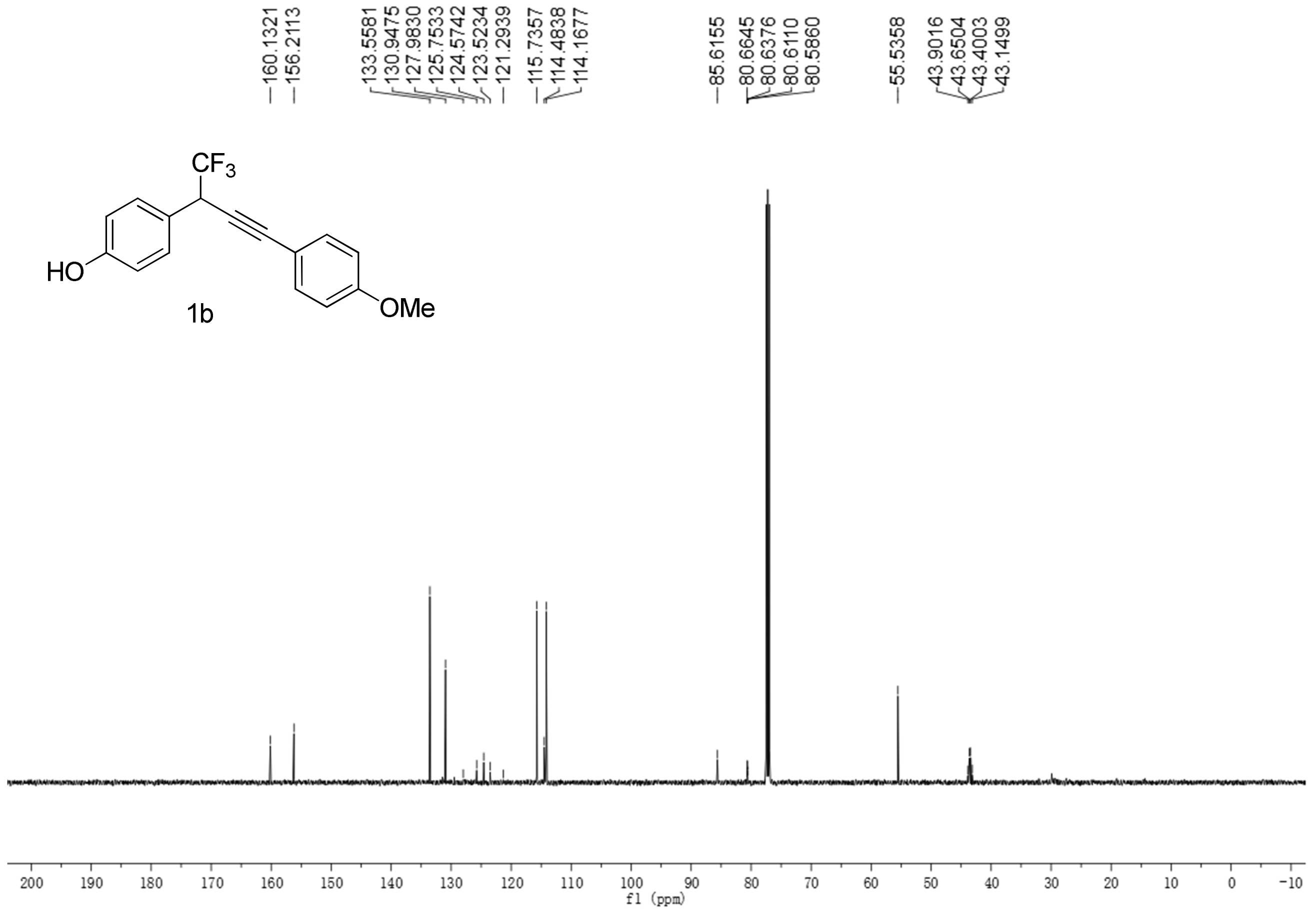
160.1321
156.2113
133.5581
130.9475
127.9830
125.7533
124.5742
123.5234
121.2939
115.7357
114.4838
114.1677
85.6155
80.6645
80.6376
80.6110
80.5860
55.5358
43.9016
43.6504
43.4003
43.1499
CF3
HO
OMe
1b
f1 (ppm)

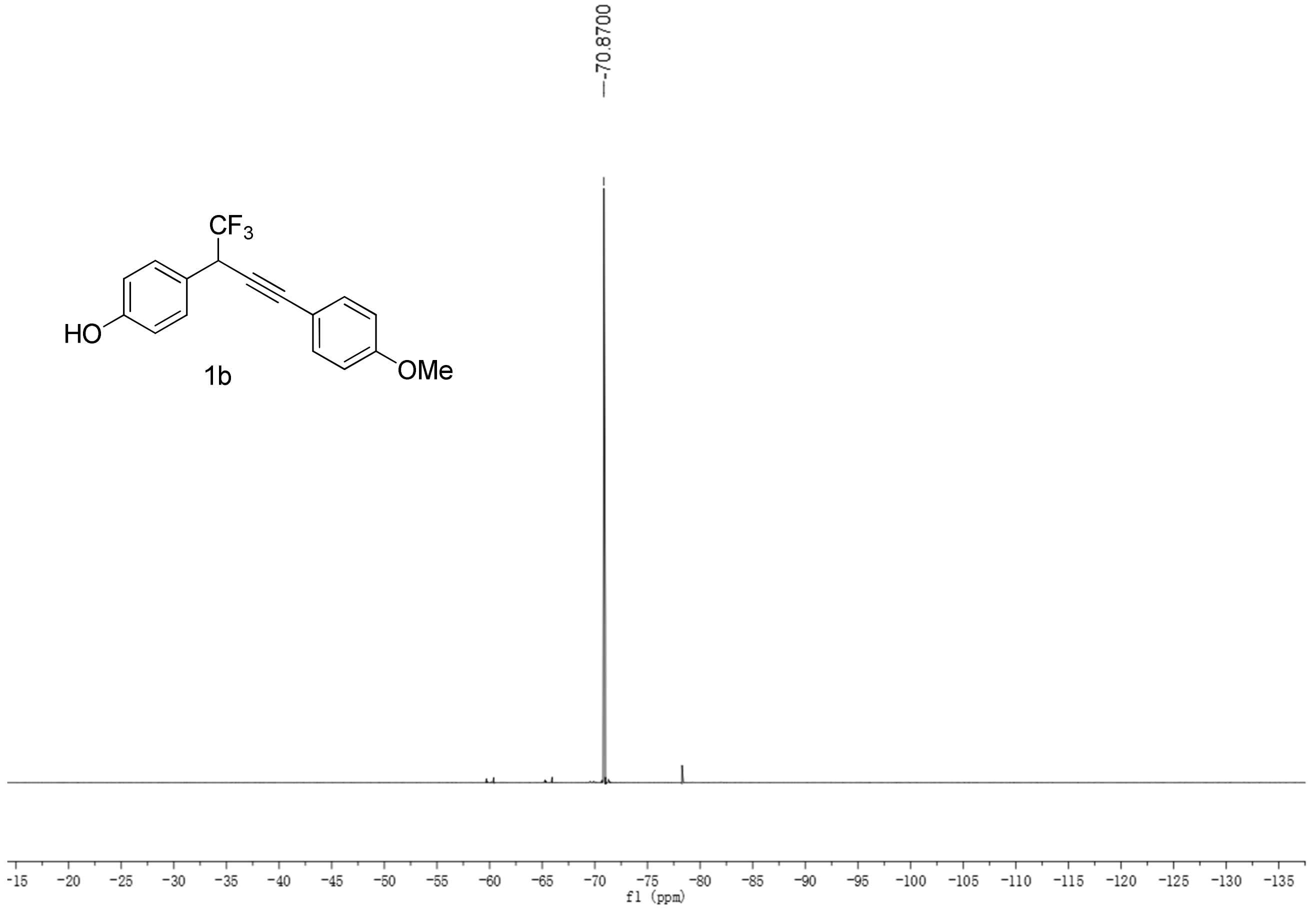
-70.8700
CF3
HO
OMe
1b
f1 (ppm)

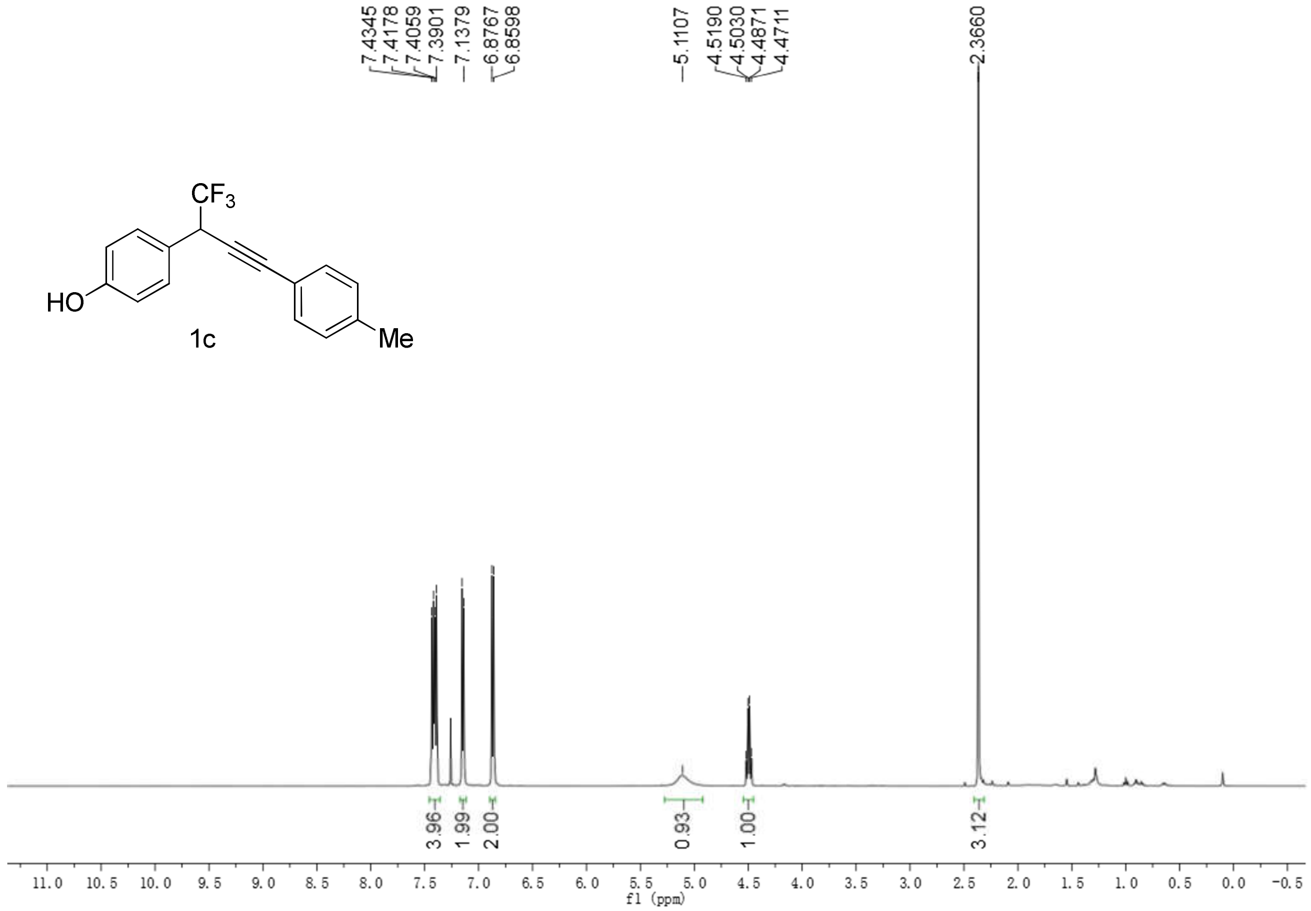
7.4345
7.4178
7.4059
7.3901
7.1379
6.8767
6.8598
5.1107
4.5190
4.5030
4.4871
4.4711
2.3660
CF_3
HO
1c
Me
3.96
1.99
2.00
0.93
1.00
3.12
11.0 10.5 10.0 9.5 9.0 8.5 8.0 7.5 7.0 6.5 6.0 5.5 5.0 4.5 4.0 3.5 3.0 2.5 2.0 1.5 1.0 0.5 0.0 -0.5
f1 (ppm)

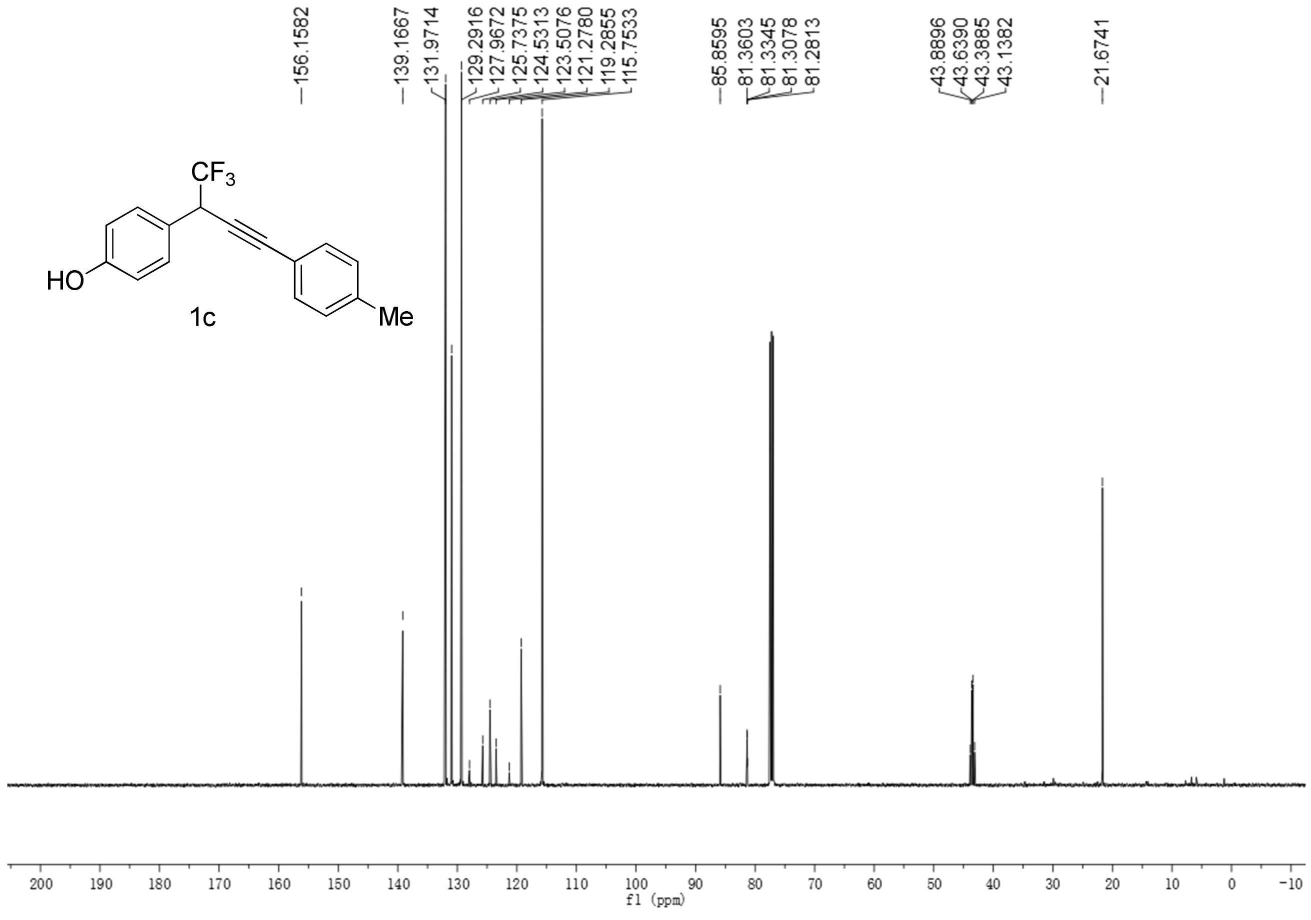
156.1582
139.1667
131.9714
129.2916
127.9672
125.7375
124.5313
123.5076
121.2780
119.2855
115.7533
85.8595
81.3603
81.3345
81.3078
81.2813
43.8896
43.6390
43.3885
43.1382
21.6741
CF_3
HO
1c
Me
200 190 180 170 160 150 140 130 120 110 100 90 80 70 60 50 40 30 20 10 0 -10
f1 (ppm)

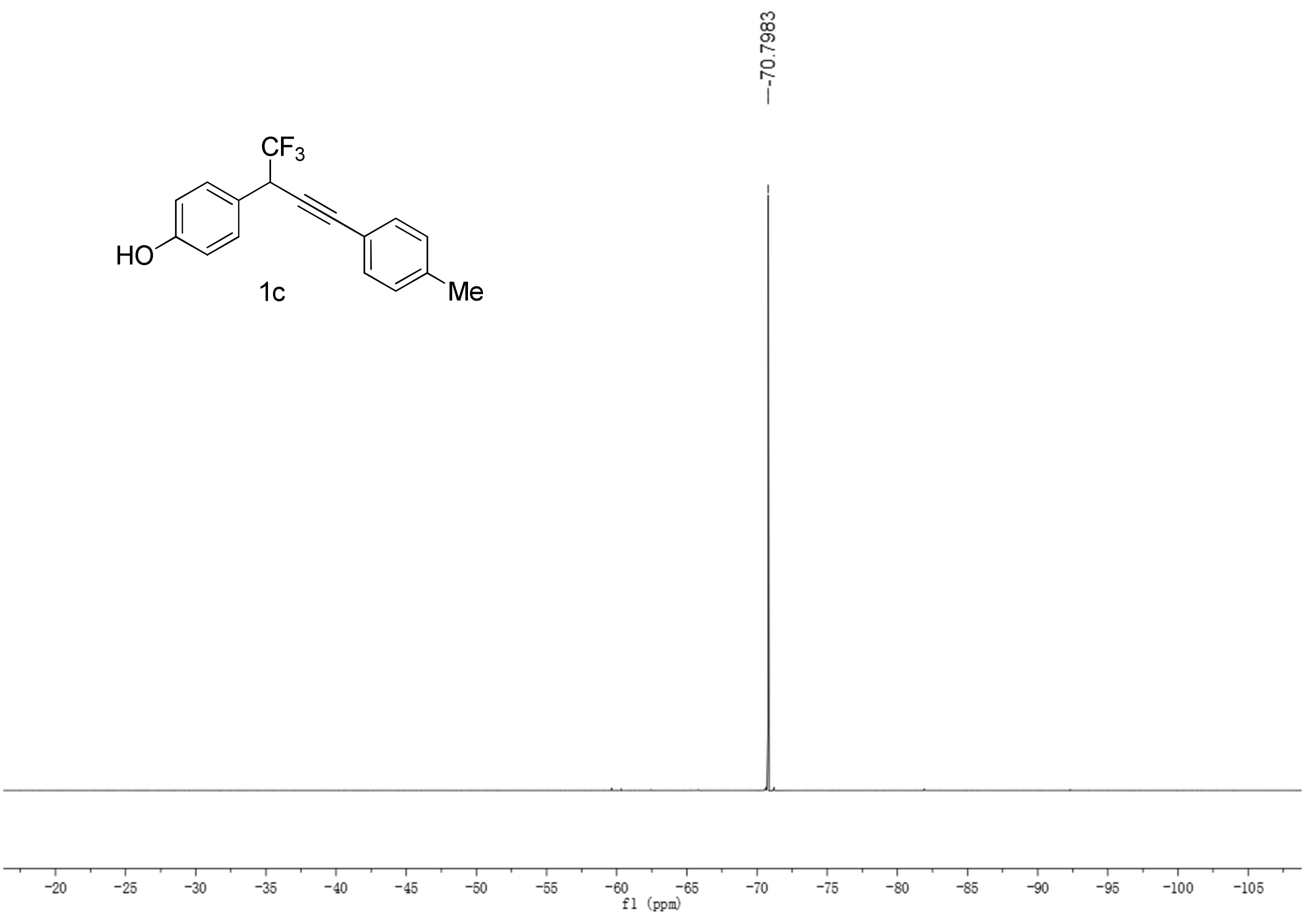

-70.7983
CF3
HO
1c
Me
-20 -25 -30 -35 -40 -45 -50 -55 -60 -65 -70 -75 -80 -85 -90 -95 -100 -105
f1 (ppm)

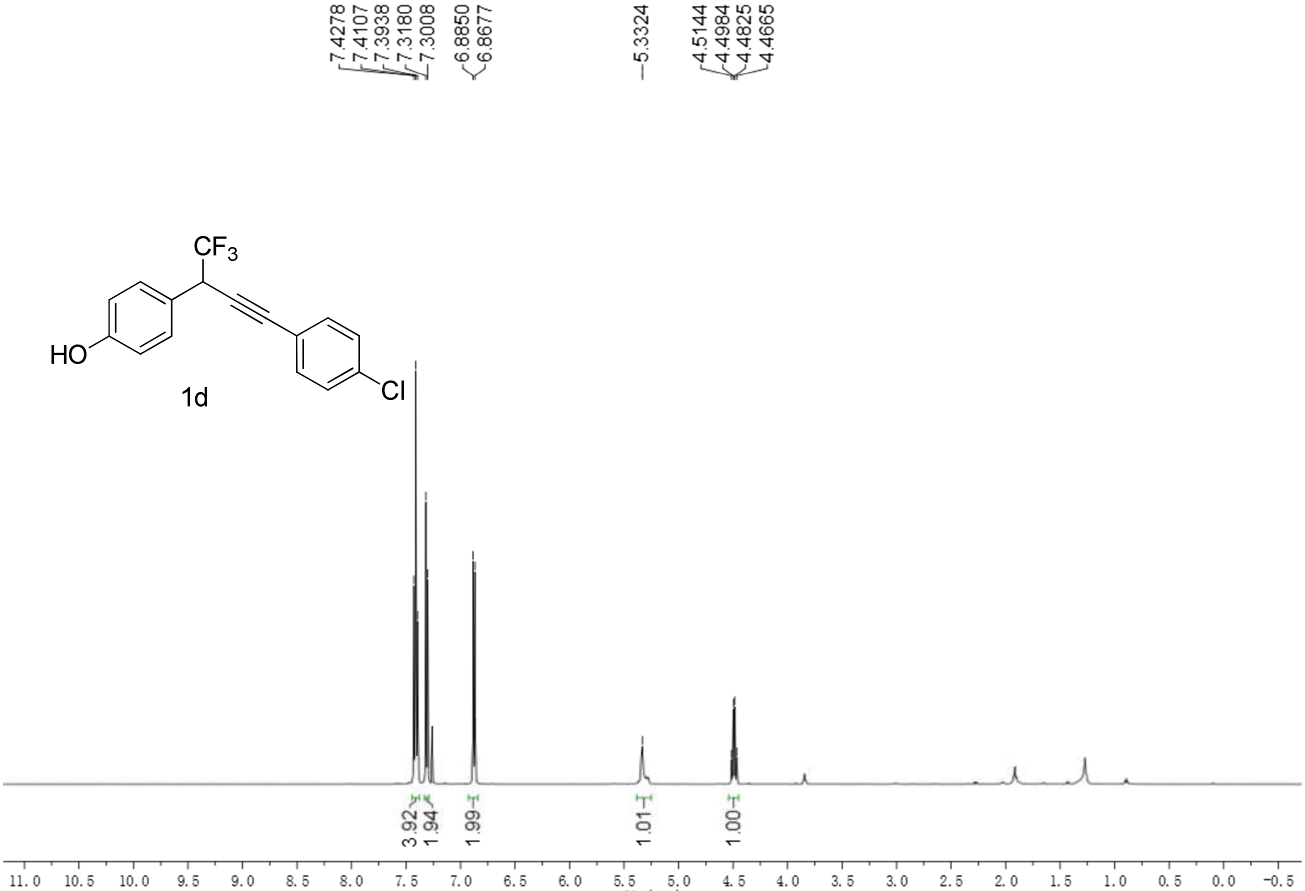

7.4278
7.4107
7.3938
7.3180
7.3008
6.8850
6.8677
5.3324
4.5144
4.4984
4.4825
4.4665
CF3
HO
1d
Cl
3.92
1.94
1.99
1.01
1.00
11.0 10.5 10.0 9.5 9.0 8.5 8.0 7.5 7.0 6.5 6.0 5.5 5.0 4.5 4.0 3.5 3.0 2.5 2.0 1.5 1.0 0.5 0.0 -0.5
f1 (ppm)

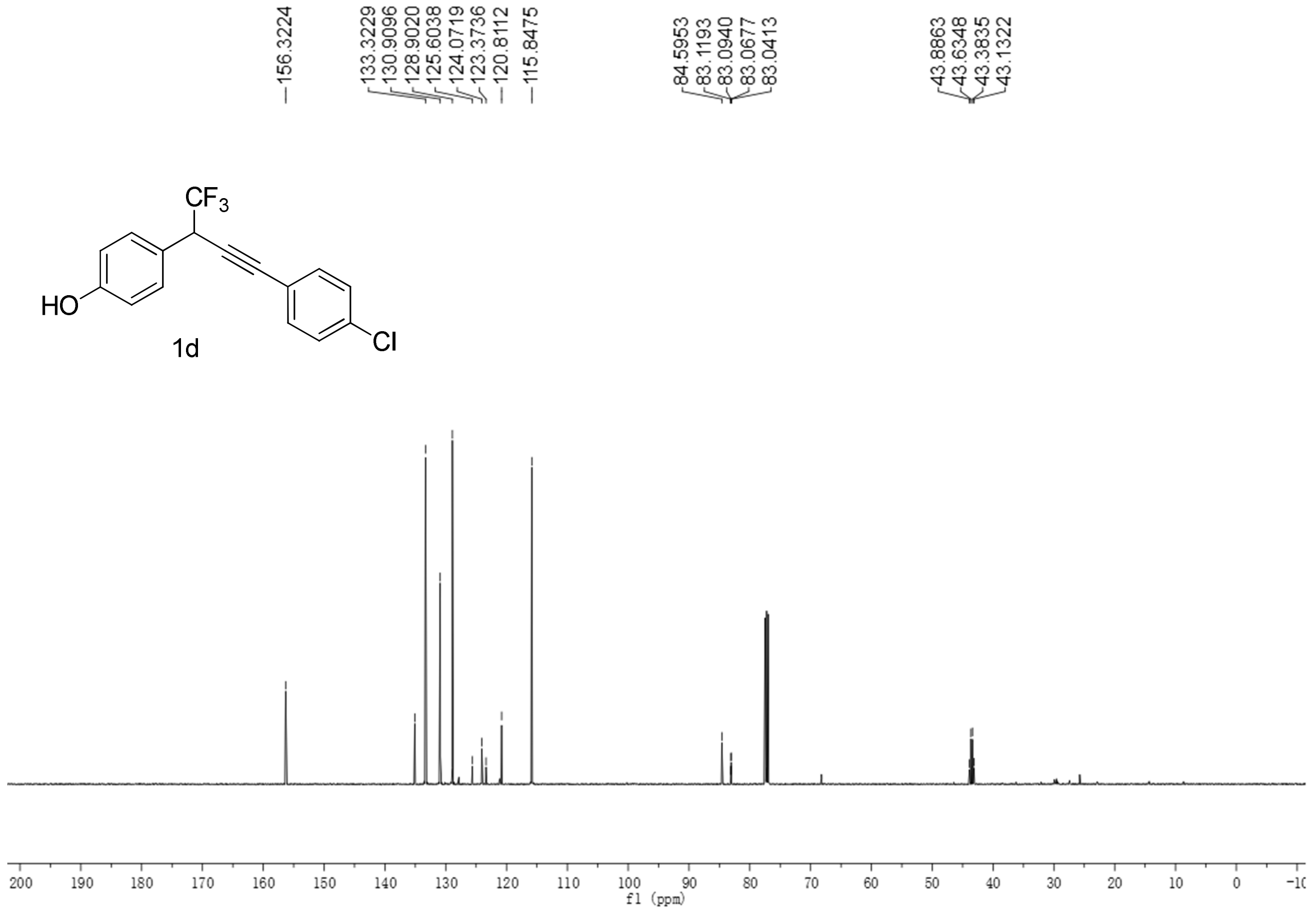

156.3224
133.3229
130.9096
128.9020
125.6038
124.0719
123.3736
120.8112
115.8475
84.5953
83.1193
83.0940
83.0677
83.0413
43.8863
43.6348
43.3835
43.1322
CF3
HO
Cl
1d
f1 (ppm)

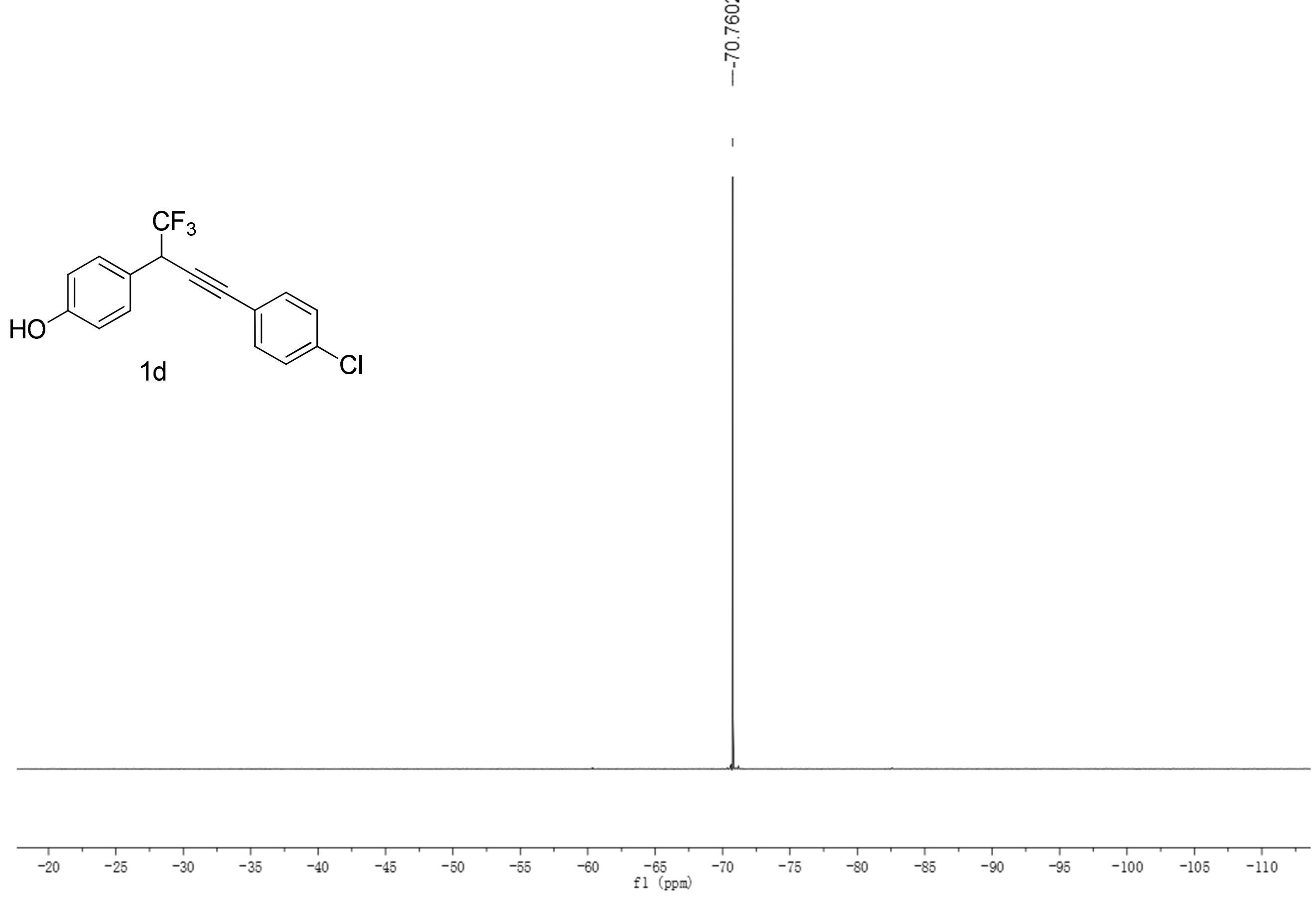

-70.7602
CF3
HO
Cl
1d
f1 (ppm)

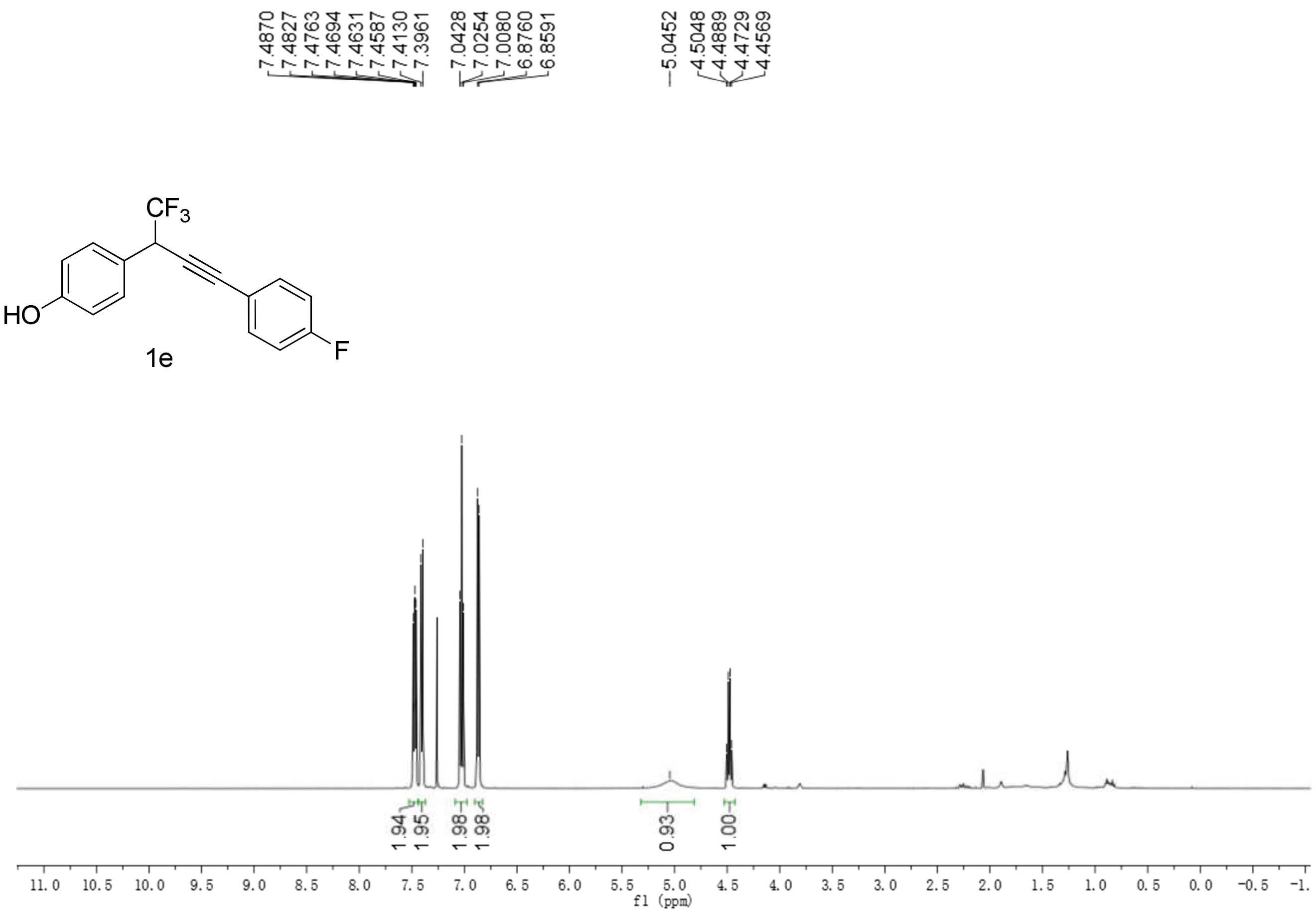
7.4870
7.4827
7.4763
7.4694
7.4631
7.4587
7.4130
7.3961
7.0428
7.0254
7.0080
6.8760
6.8591
5.0452
4.5048
4.4889
4.4729
4.4569
CF3
HO
F
1e
1.94
1.95
1.98
1.98
0.93
1.00
11.0 10.5 10.0 9.5 9.0 8.5 8.0 7.5 7.0 6.5 6.0 5.5 5.0 4.5 4.0 3.5 3.0 2.5 2.0 1.5 1.0 0.5 0.0 -0.5 -1.
f1 (ppm)

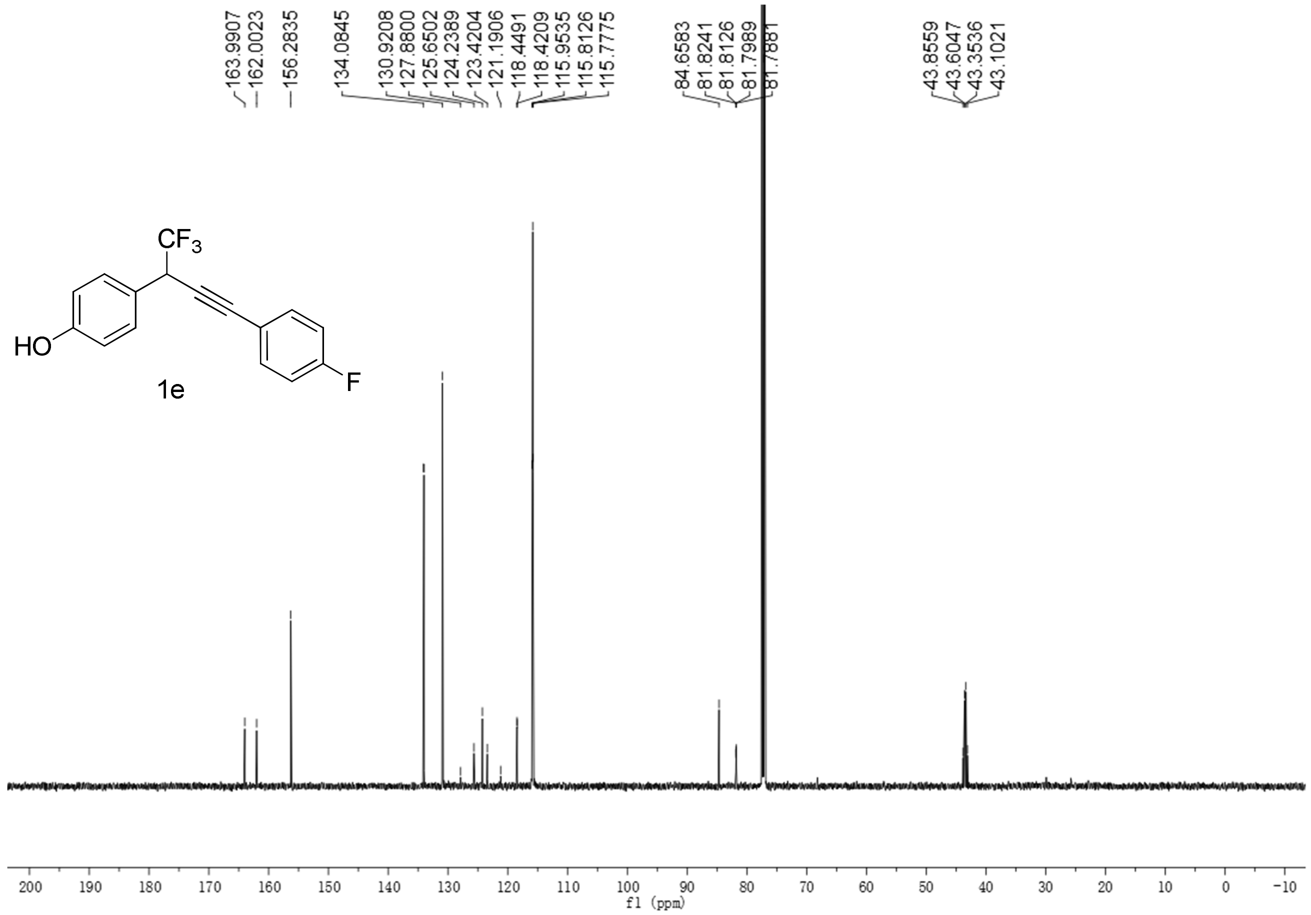
163.9907
162.0023
156.2835
134.0845
130.9208
127.8800
125.6502
124.2389
123.4204
121.1906
118.4491
118.4209
115.9535
115.8126
115.7775
84.6583
81.8241
81.8126
81.7989
81.7881
43.8559
43.6047
43.3536
43.1021
CF3
HO
F
1e
200 190 180 170 160 150 140 130 120 110 100 90 80 70 60 50 40 30 20 10 0 -10
f1 (ppm)

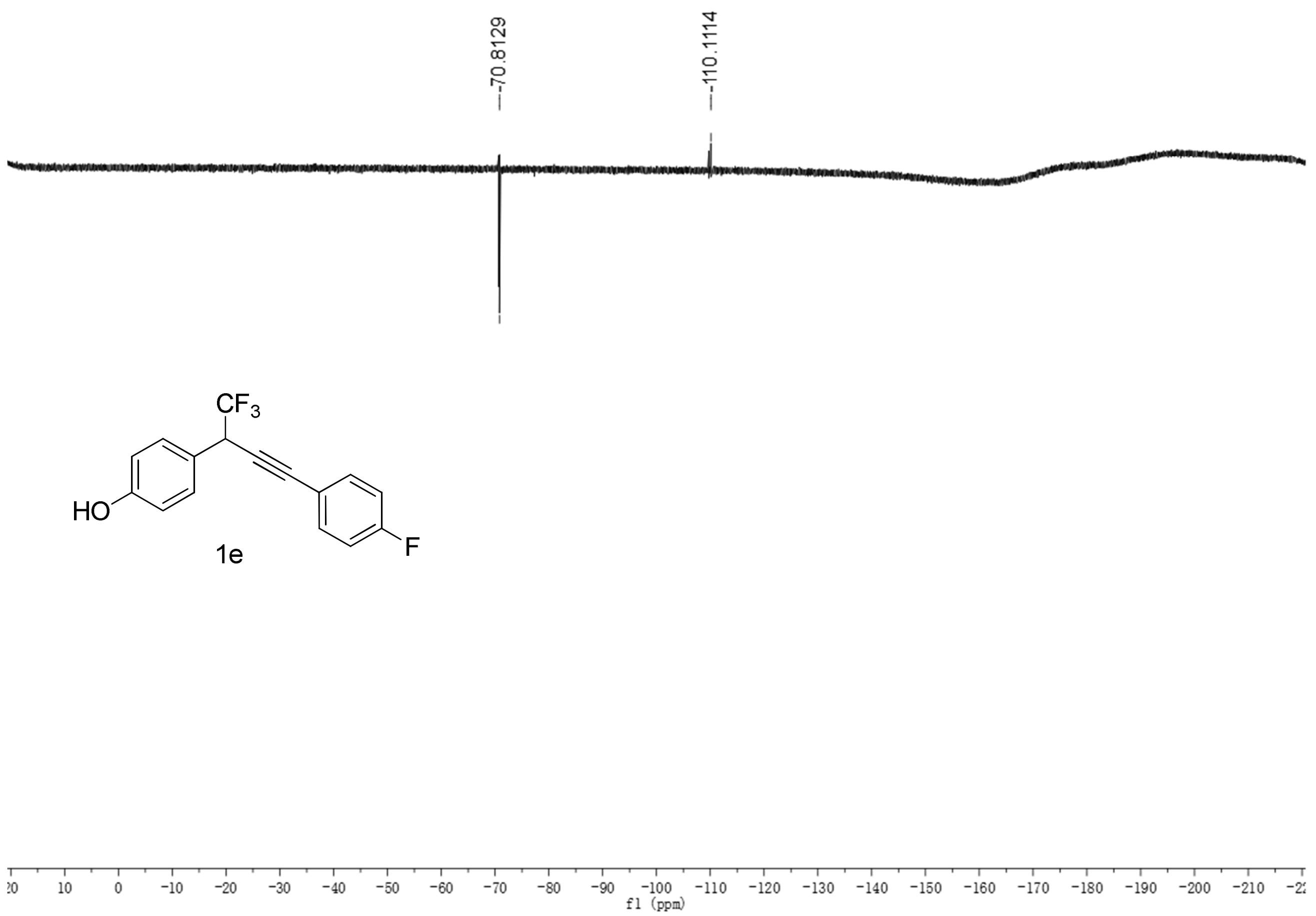

-70.8129
-110.1114
CF3
HO
F
1e
f1 (ppm)

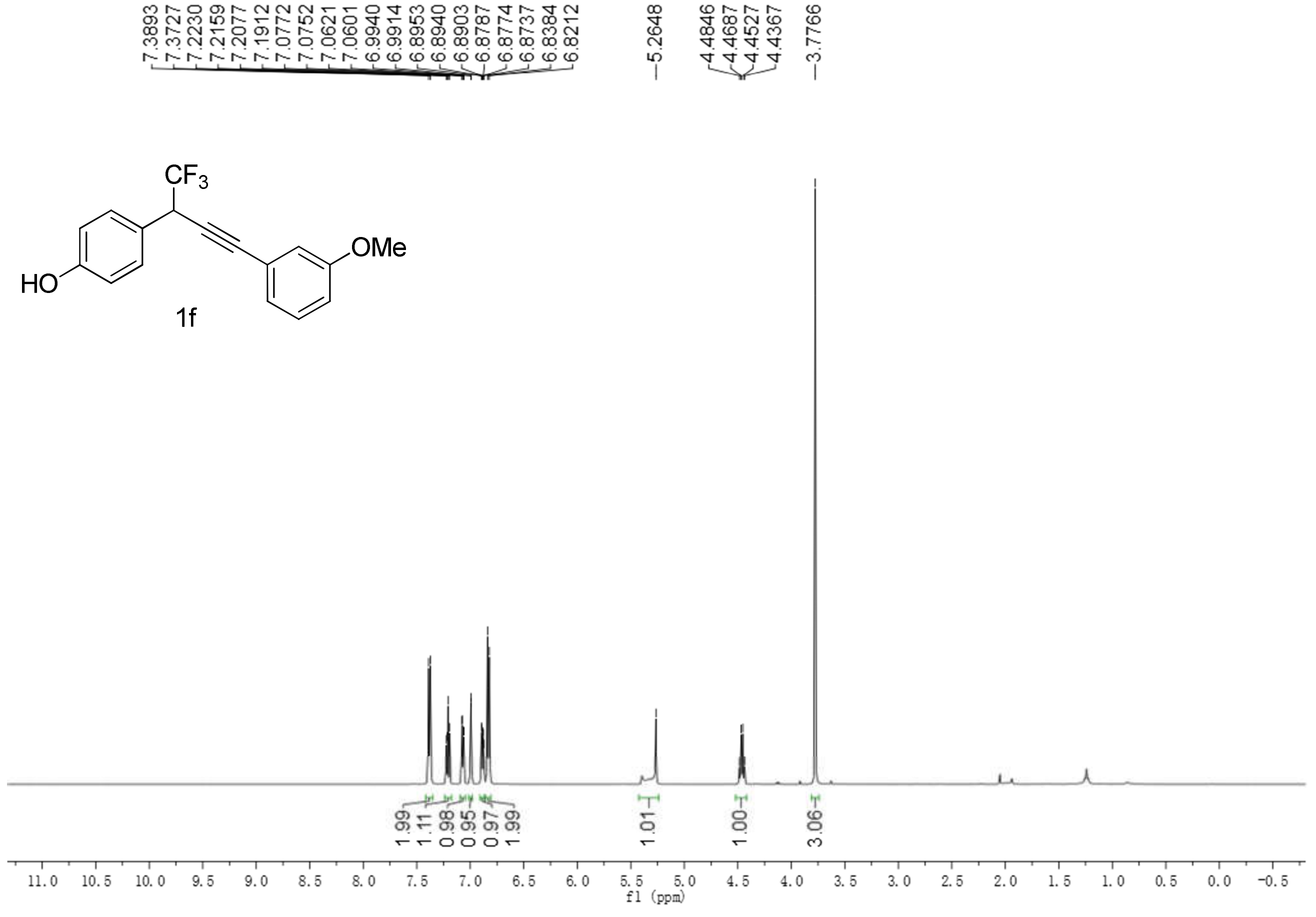

7.3893
7.3727
7.2230
7.2159
7.2077
7.1912
7.0772
7.0752
7.0621
7.0601
6.9940
6.9914
6.8953
6.8940
6.8903
6.8787
6.8774
6.8737
6.8384
6.8212
5.2648
4.4846
4.4687
4.4527
4.4367
3.7766
CF3
OMe
HO
1f
1.99
1.11
0.98
0.95
0.97
1.99
1.01
1.00
3.06
f1 (ppm)

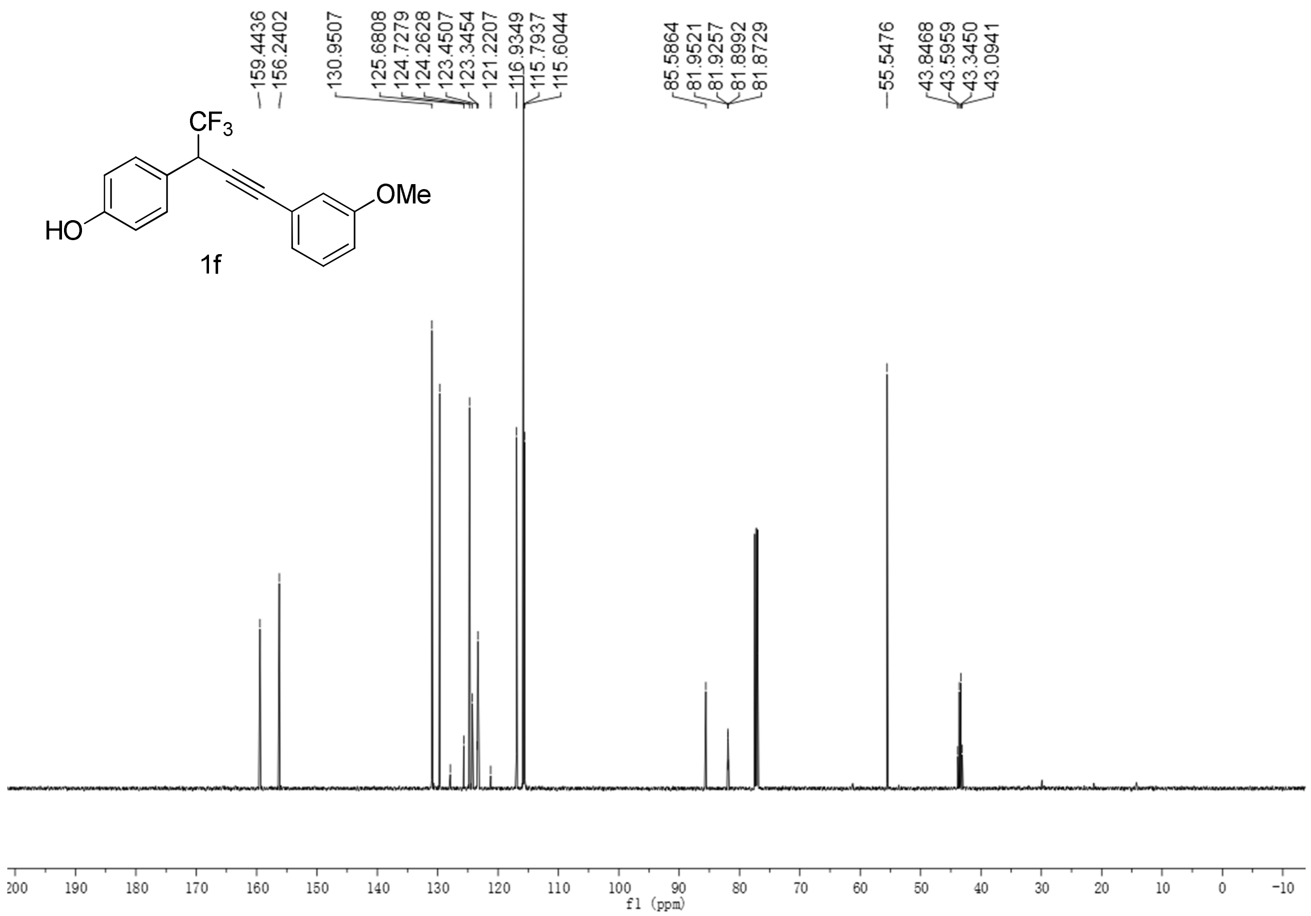
159.4436
156.2402
130.9507
125.6808
124.7279
124.2628
123.4507
123.3454
121.2207
116.9349
115.7937
115.6044
85.5864
81.9521
81.9257
81.8992
81.8729
55.5476
43.8468
43.5959
43.3450
43.0941
CF3
OMe
HO
1f
f1 (ppm)

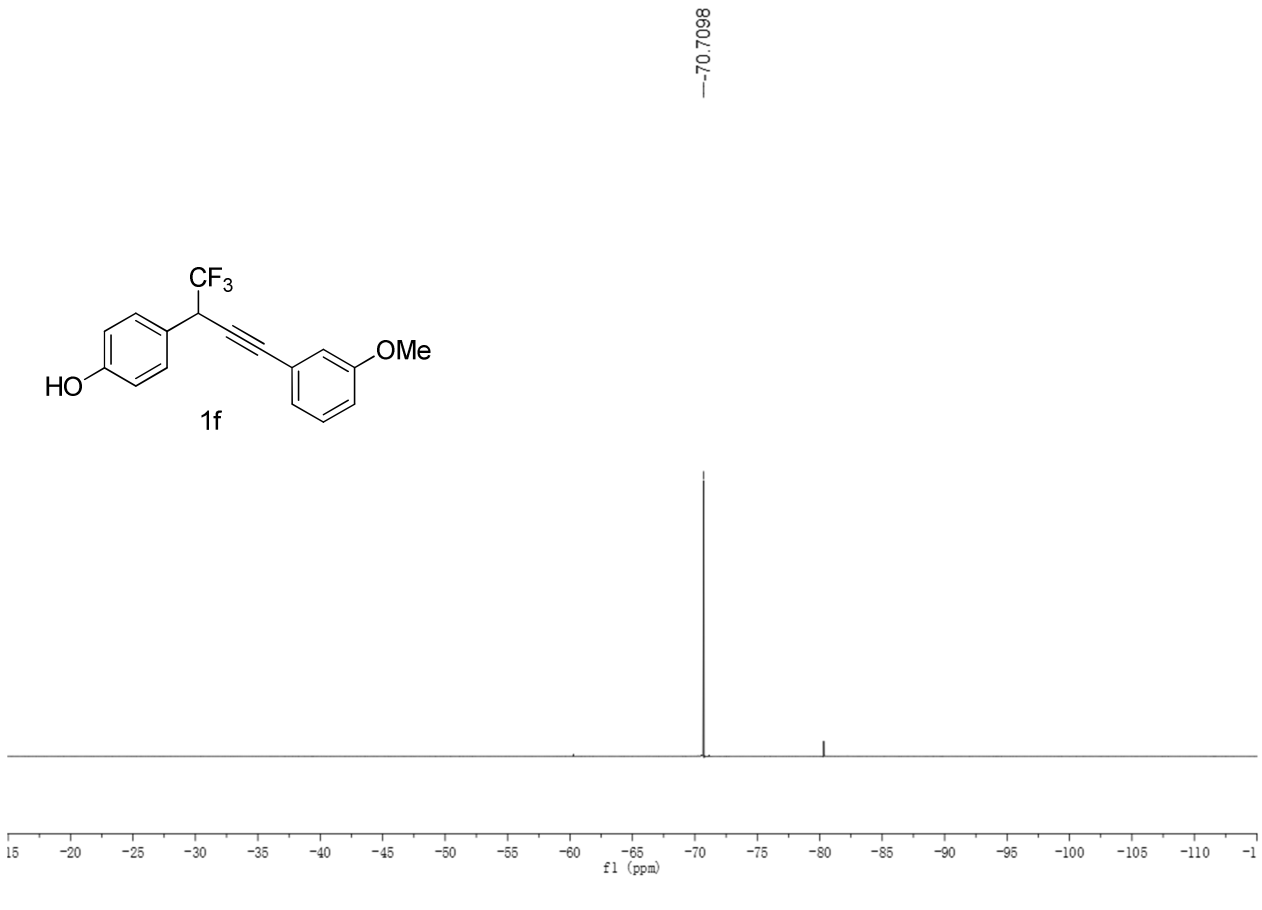
-70.7098
CF3
OMe
HO
1f
f1 (ppm)

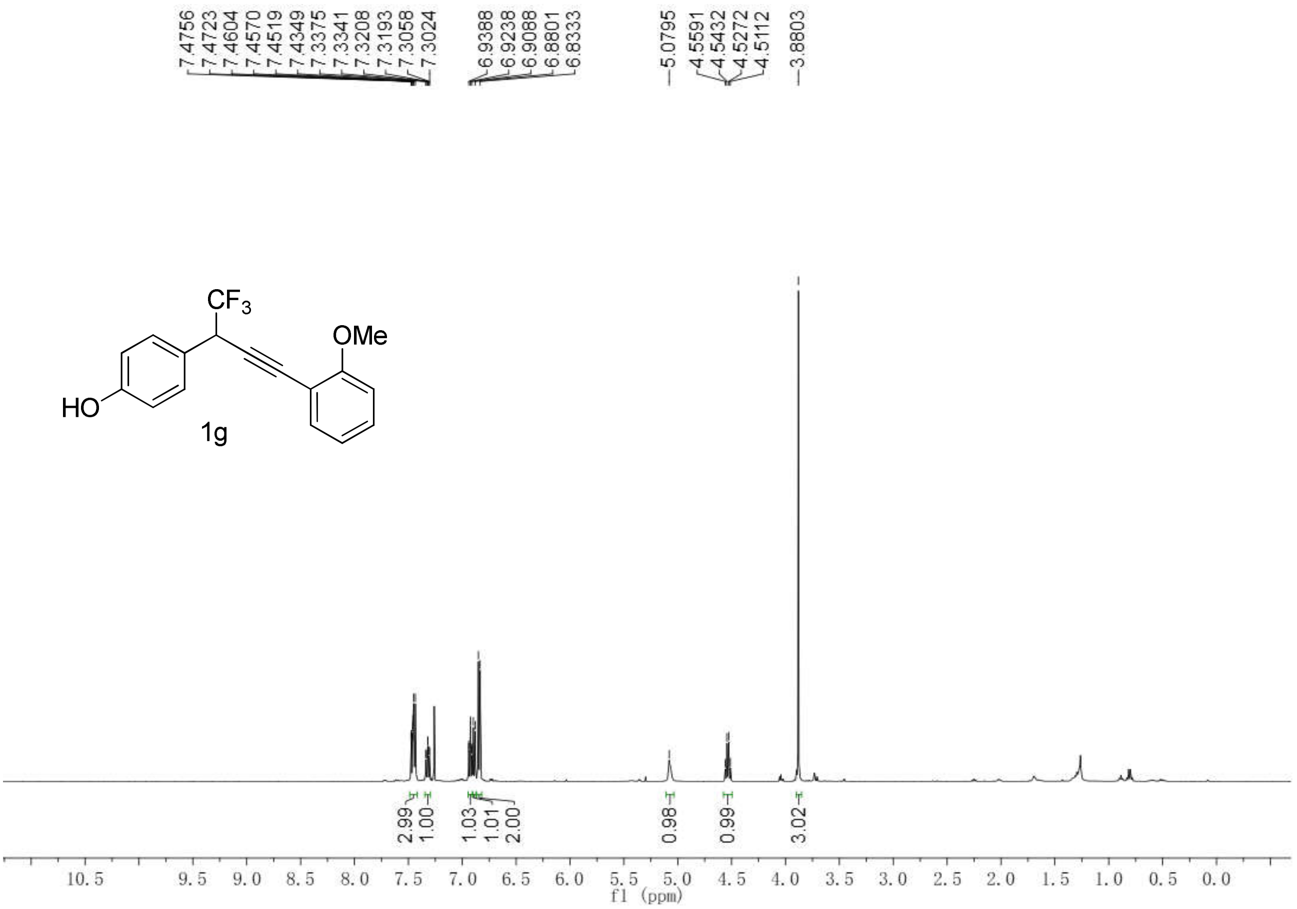

7.4756
7.4723
7.4604
7.4570
7.4519
7.4349
7.3375
7.3341
7.3208
7.3193
7.3058
7.3024
6.9388
6.9238
6.9088
6.8801
6.8333
5.0795
4.5591
4.5432
4.5272
4.5112
3.8803
CF3
OMe
HO
1g
2.99
1.00
1.03
1.01
2.00
0.98
0.99
3.02
10.5 9.5 9.0 8.5 8.0 7.5 7.0 6.5 6.0 5.5 5.0 4.5 4.0 3.5 3.0 2.5 2.0 1.5 1.0 0.5 0.0
f1 (ppm)

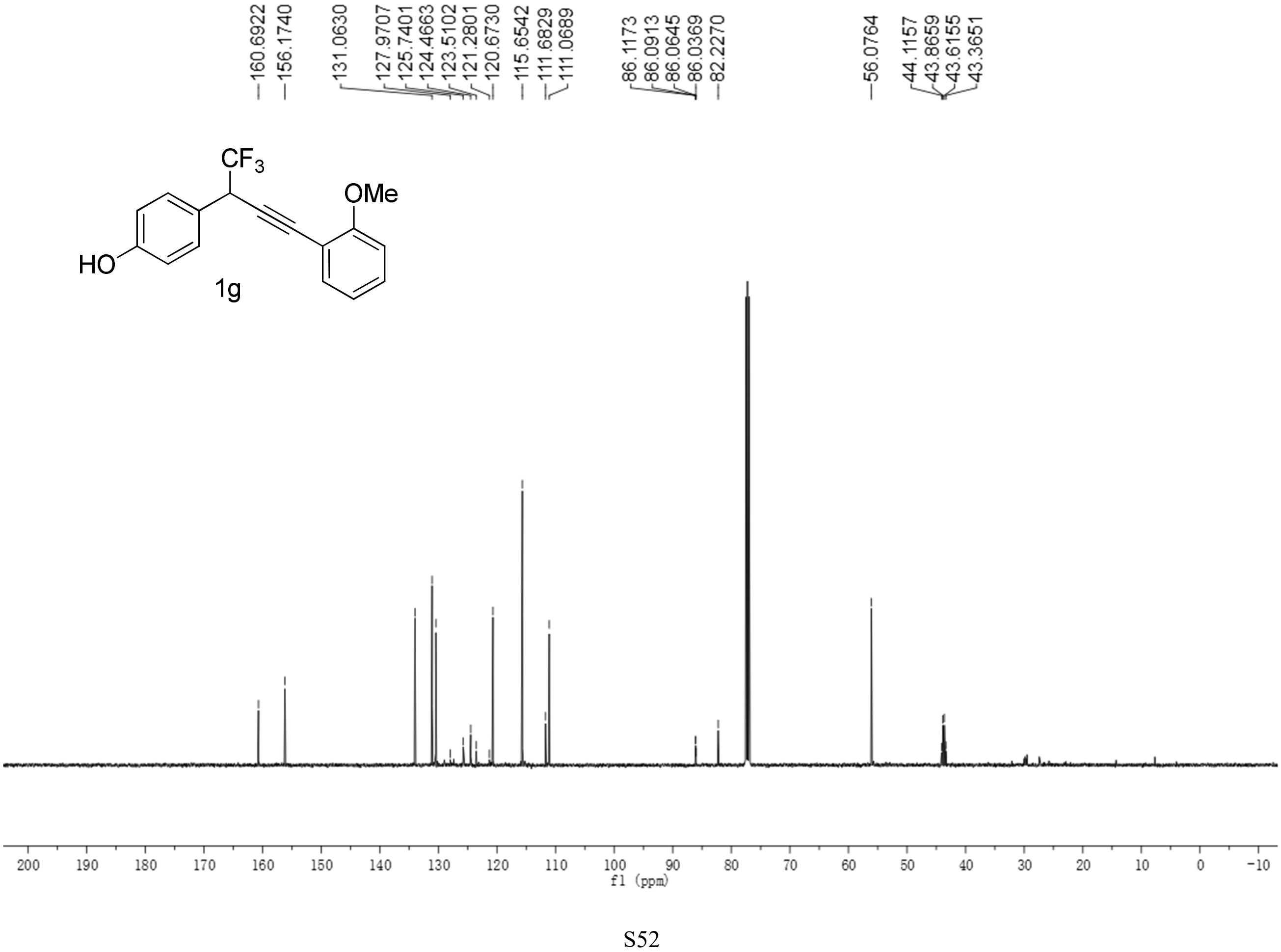

160.6922
156.1740
131.0630
127.9707
125.7401
124.4663
123.5102
121.2801
120.6730
115.6542
111.6829
111.0689
86.1173
86.0913
86.0645
86.0369
82.2270
56.0764
44.1157
43.8659
43.6155
43.3651
CF3
OMe
HO
1g
200 190 180 170 160 150 140 130 120 110 100 90 80 70 60 50 40 30 20 10 0 -10
f1 (ppm)

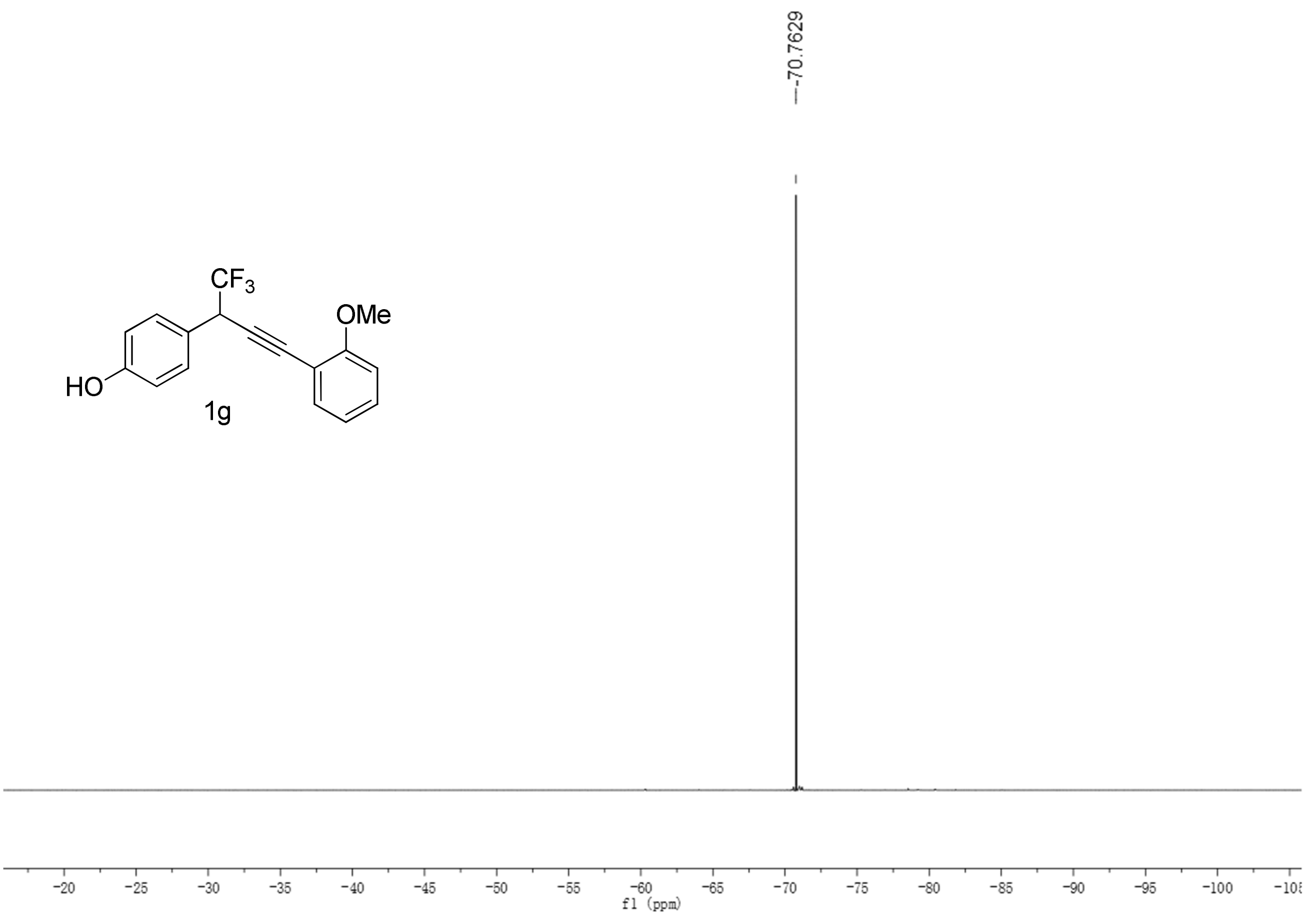

-70.7629
CF3
OMe
HO
-20 -25 -30 -35 -40 -45 -50 -55 -60 -65 -70 -75 -80 -85 -90 -95 -100
f1 (ppm)

1g

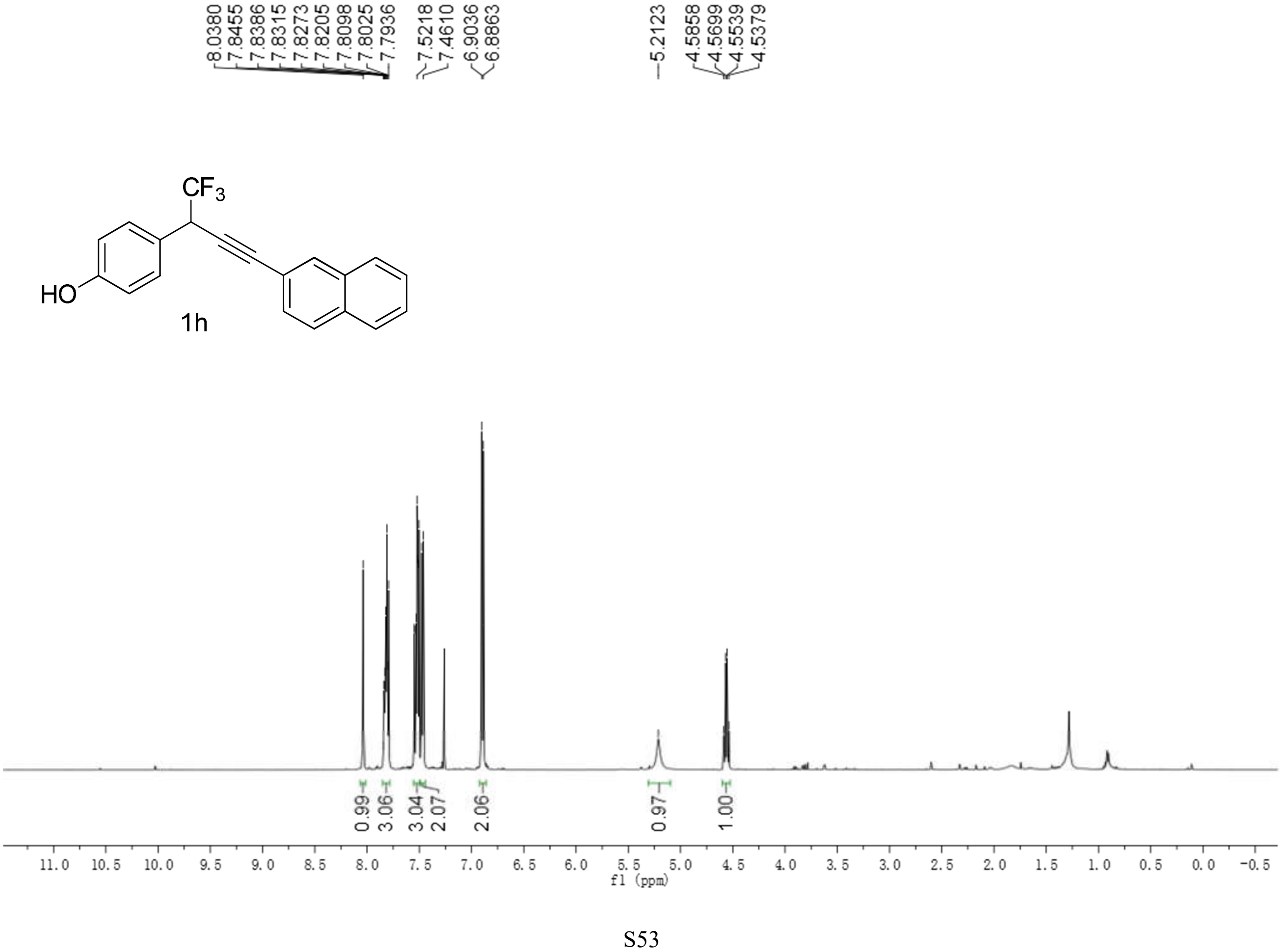

8.0380
7.8455
7.8386
7.8315
7.8273
7.8205
7.8098
7.8025
7.7936
7.5218
7.4610
6.9036
6.8863
5.2123
4.5858
4.5699
4.5539
4.5379
CF3
HO
0.99
3.06
3.04
2.07
2.06
0.97
1.00
11.0 10.5 10.0 9.5 9.0 8.5 8.0 7.5 7.0 6.5 6.0 5.5 5.0 4.5 4.0 3.5 3.0 2.5 2.0 1.5 1.0 0.5 0.0 -0.5
f1 (ppm)

1h

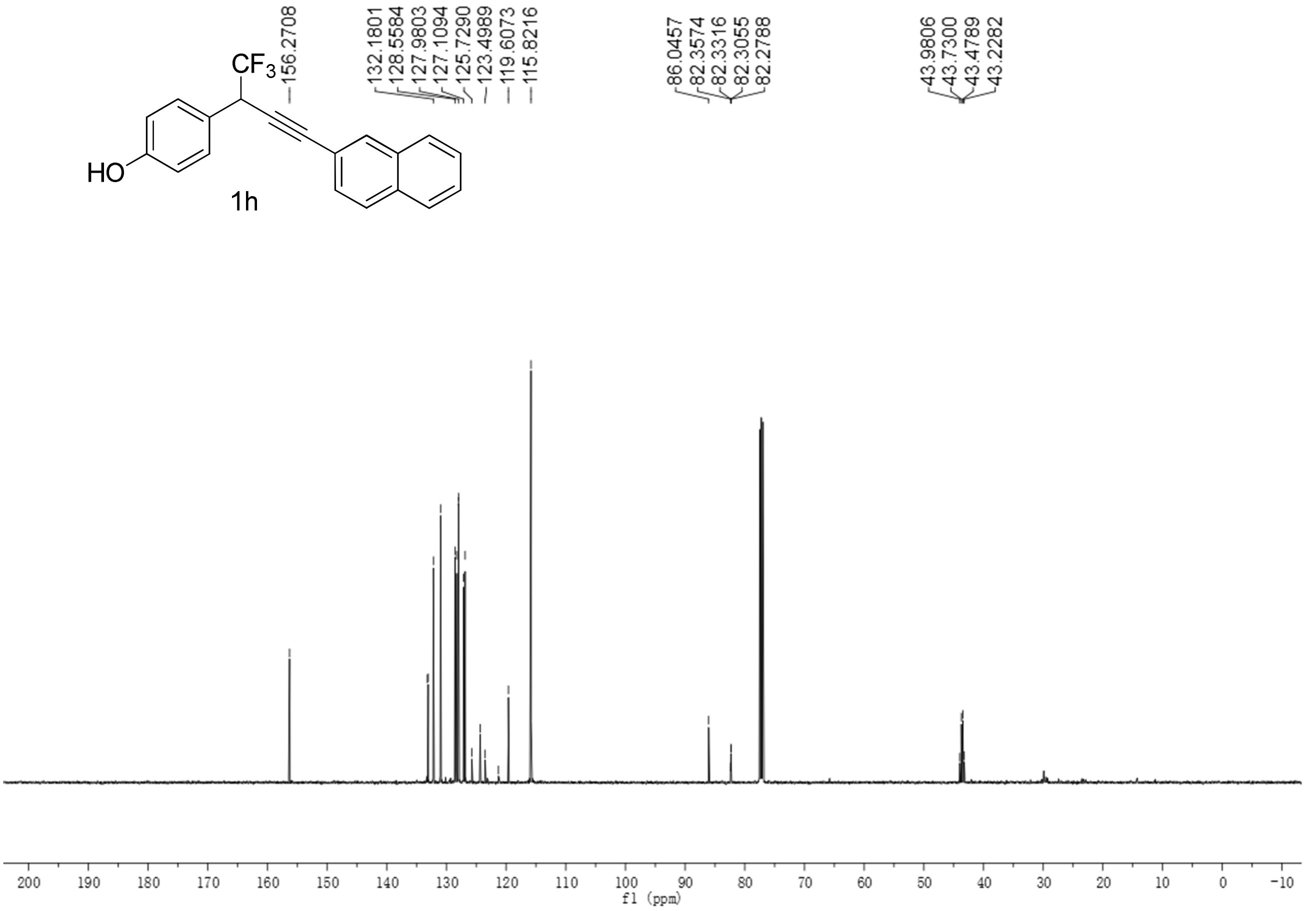
CF3
HO
1h
156.2708
132.1801
128.5584
127.9803
127.1094
125.7290
123.4989
119.6073
115.8216
86.0457
82.3574
82.3316
82.3055
82.2788
43.9806
43.7300
43.4789
43.2282
f1 (ppm)

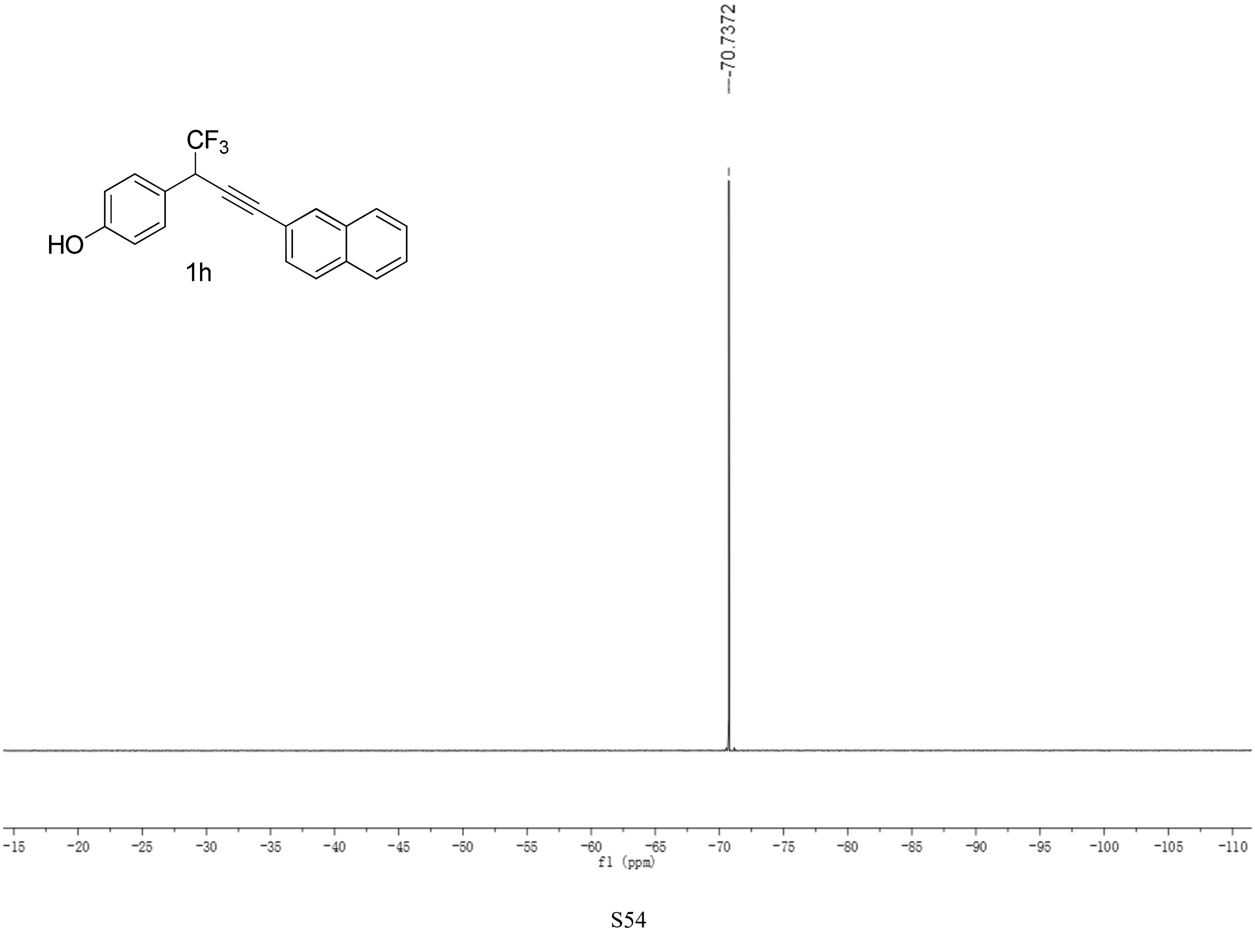
-70.7372
CF3
HO
1h
f1 (ppm)

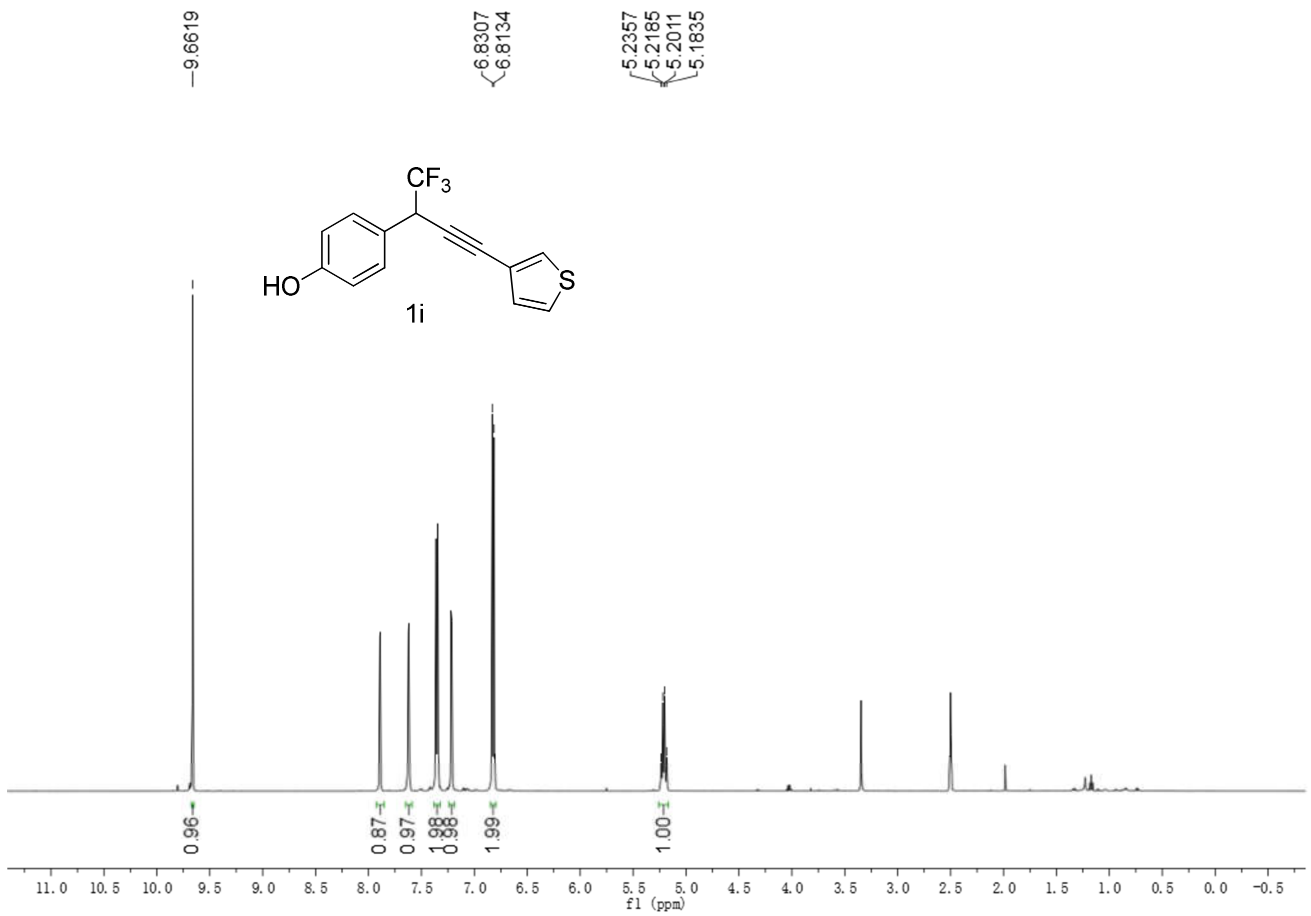
9.6619
6.8307
6.8134
5.2357
5.2185
5.2011
5.1835
CF3
HO
S
1i
0.96
0.87
0.97
1.98
0.98
1.99
1.00
f1 (ppm)

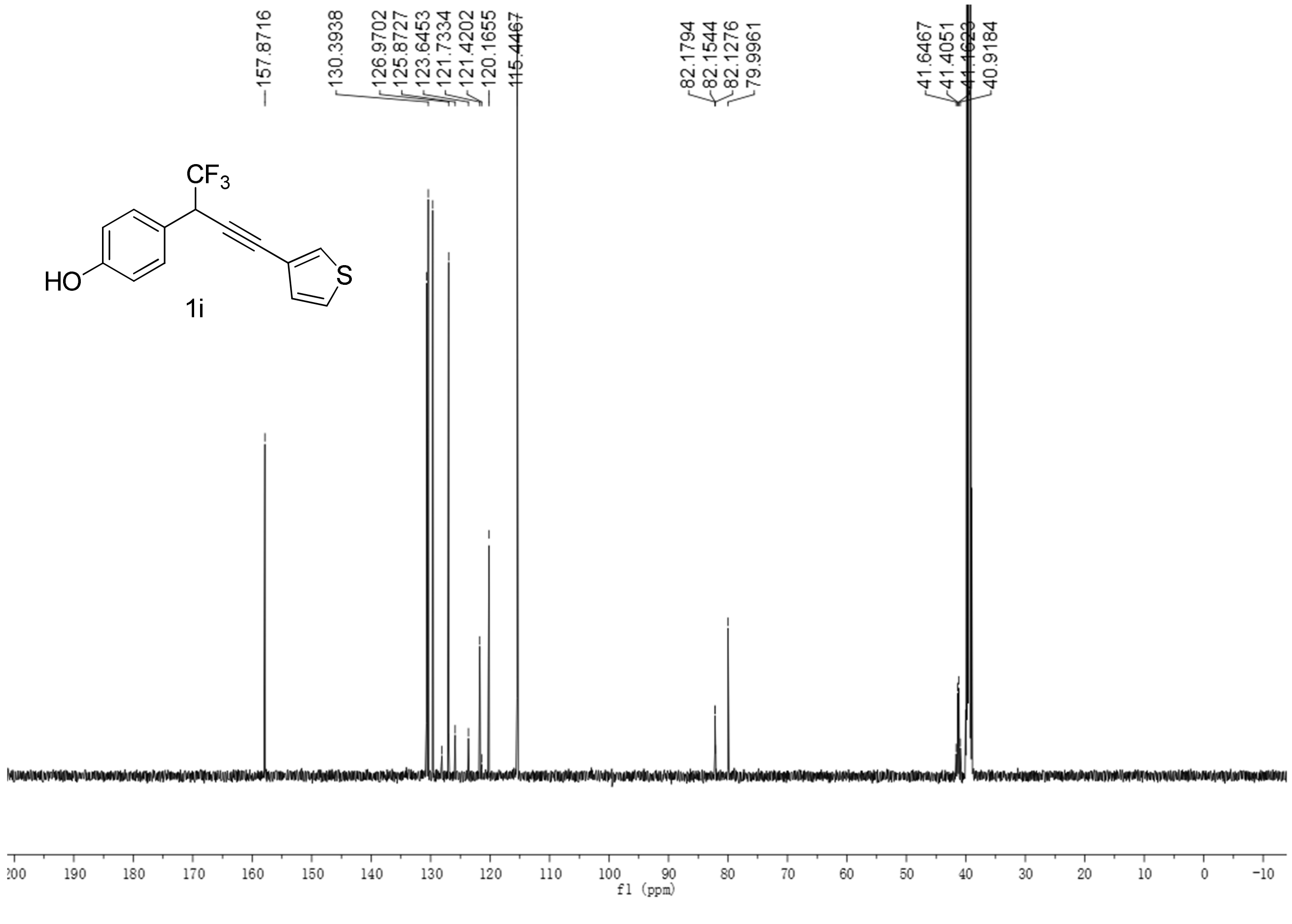
157.8716
130.3938
126.9702
125.8727
123.6453
121.7334
121.4202
120.1655
115.4467
82.1794
82.1544
82.1276
79.9961
41.6467
41.4051
40.9184
CF3
HO
S
1i
f1 (ppm)

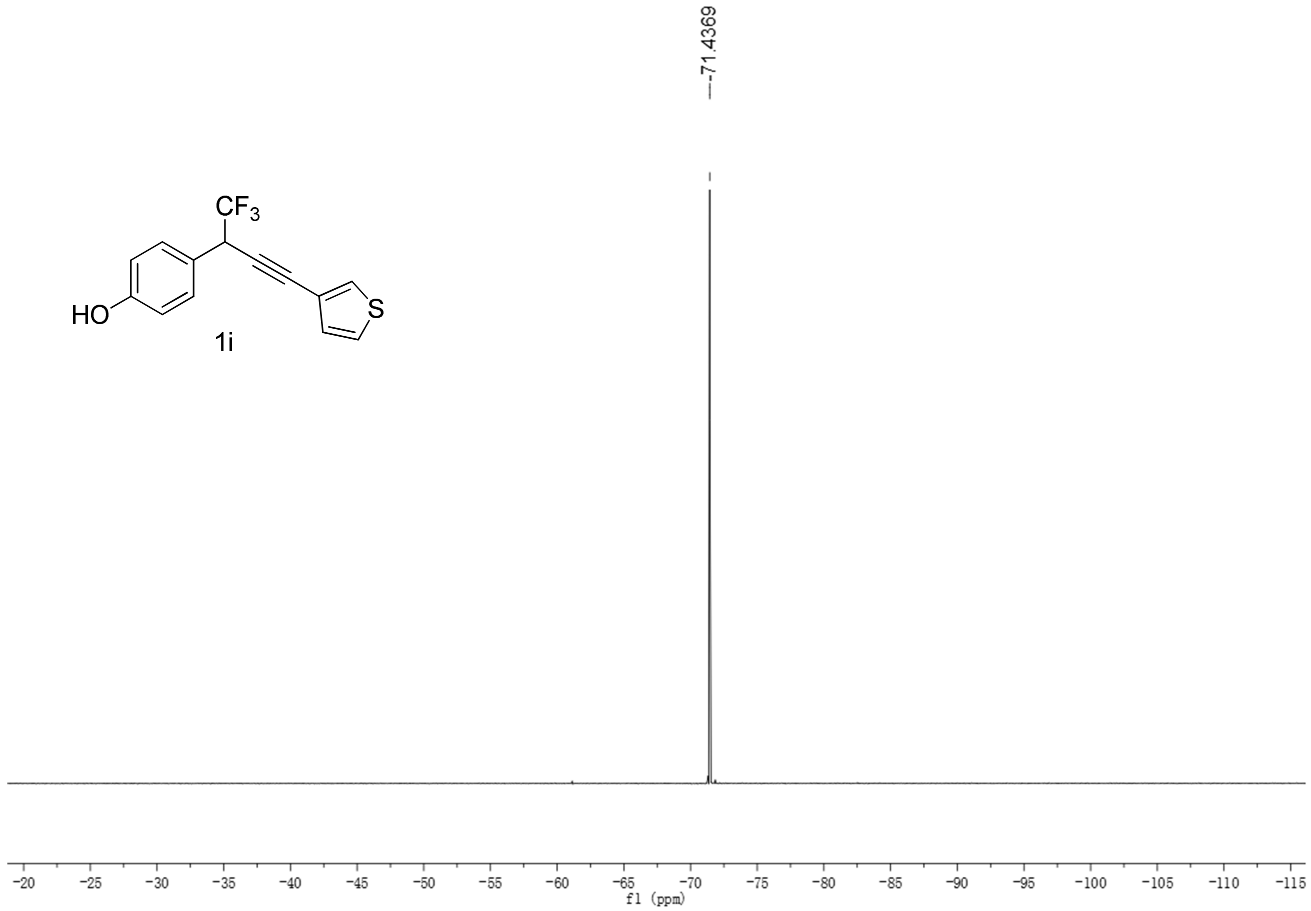
-71.4369
CF3
HO
S
1i
f1 (ppm)

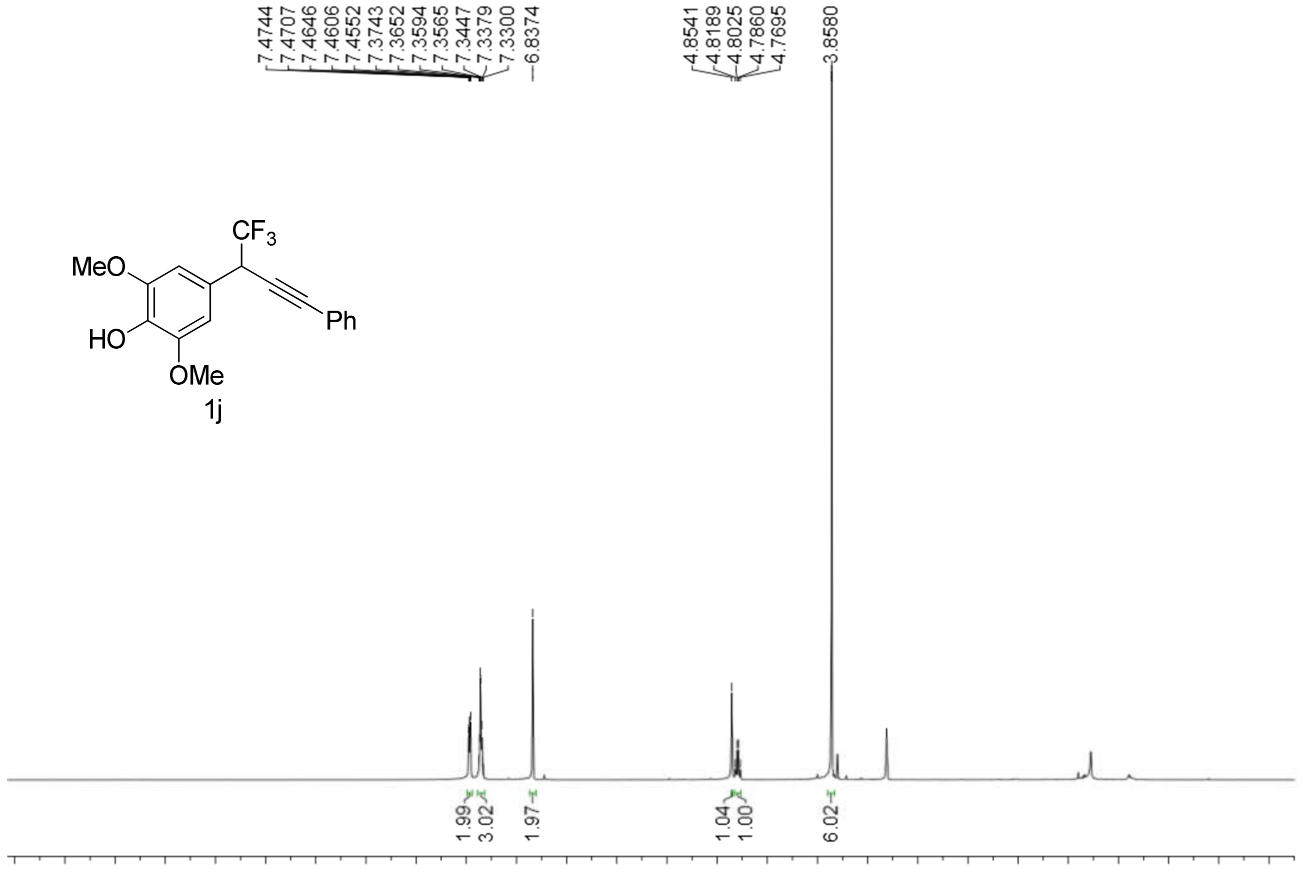
7.4744
7.4707
7.4646
7.4606
7.4552
7.3743
7.3652
7.3594
7.3565
7.3447
7.3379
7.3300
6.8374
4.8541
4.8189
4.8025
4.7860
4.7695
3.8580
CF3
MeO
HO
OMe
Ph
1j
1.99
3.02
1.97
1.04
1.00
6.02
f1 (ppm)

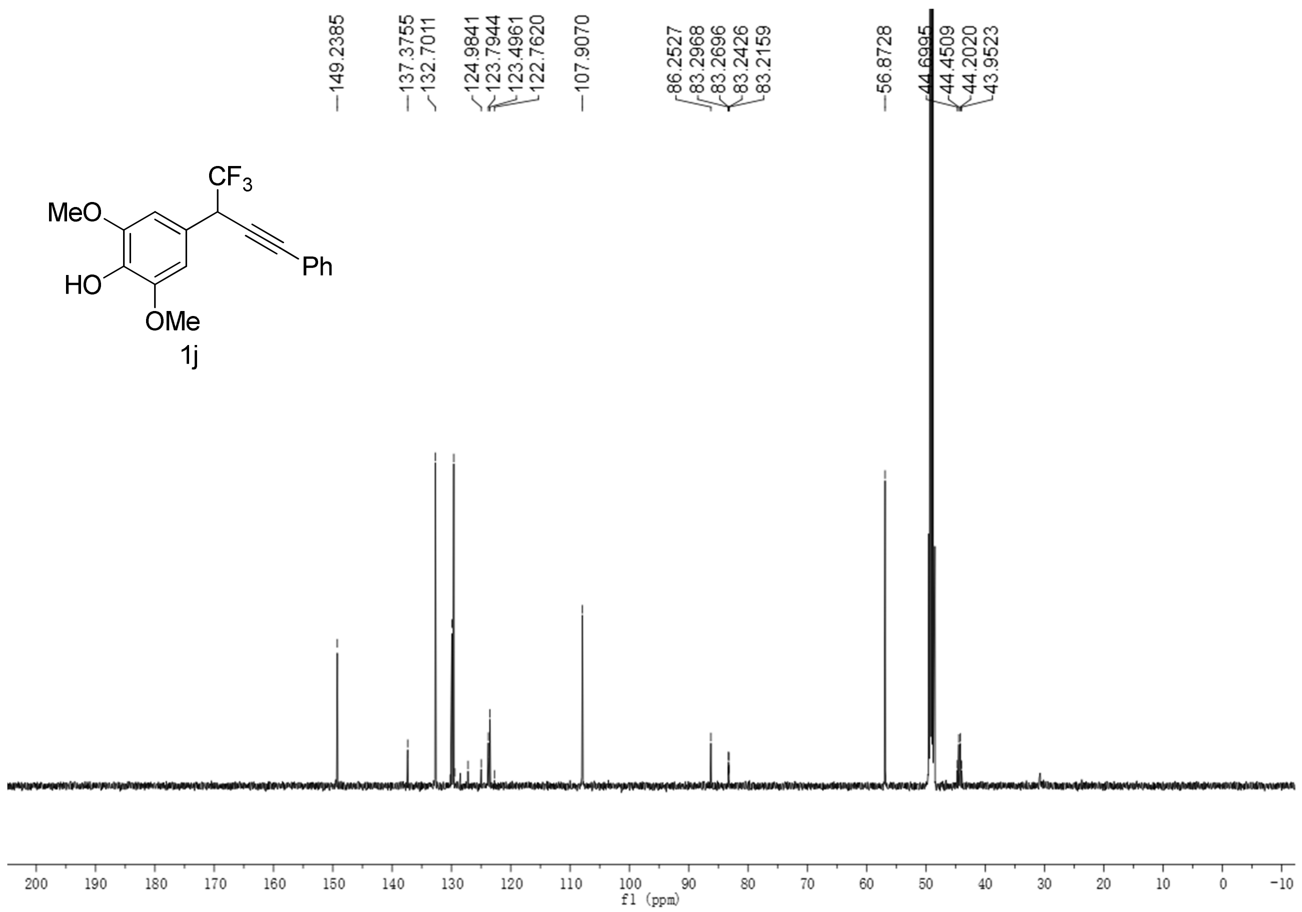
149.2385
137.3755
132.7011
124.9841
123.7944
123.4961
122.7620
107.9070
86.2527
83.2968
83.2696
83.2426
83.2159
56.8728
44.6995
44.4509
44.2020
43.9523
CF3
MeO
HO
OMe
Ph
1j
f1 (ppm)

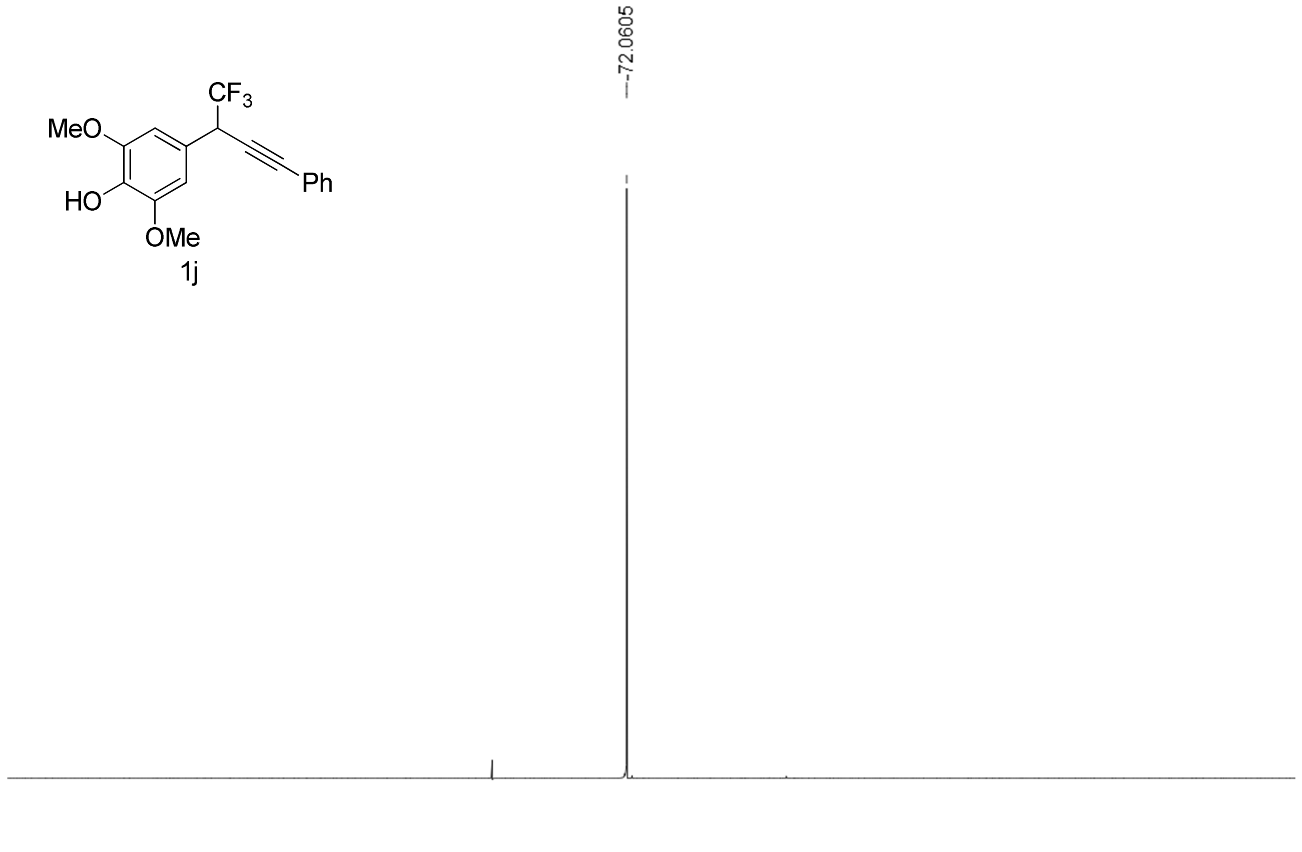
-72.0605
CF3
MeO
HO
OMe
Ph
1j
f1 (ppm)

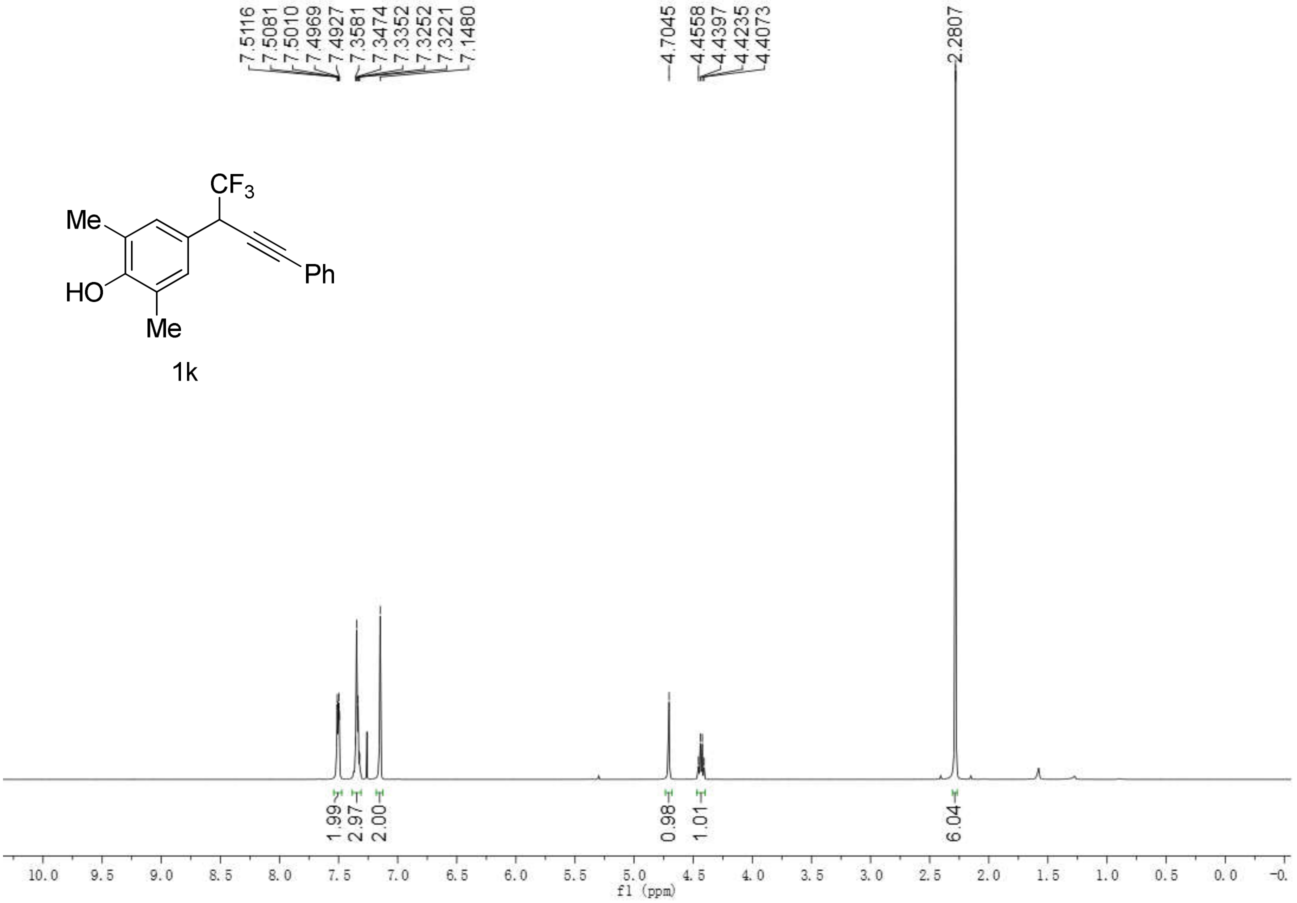
7.5116
7.5081
7.5010
7.4969
7.4927
7.3581
7.3474
7.3352
7.3252
7.3221
7.1480
4.7045
4.4558
4.4397
4.4235
4.4073
2.2807
CF3
Me
HO
Me
Ph
1k
1.99
2.97
2.00
0.98
1.01
6.04
f1 (ppm)

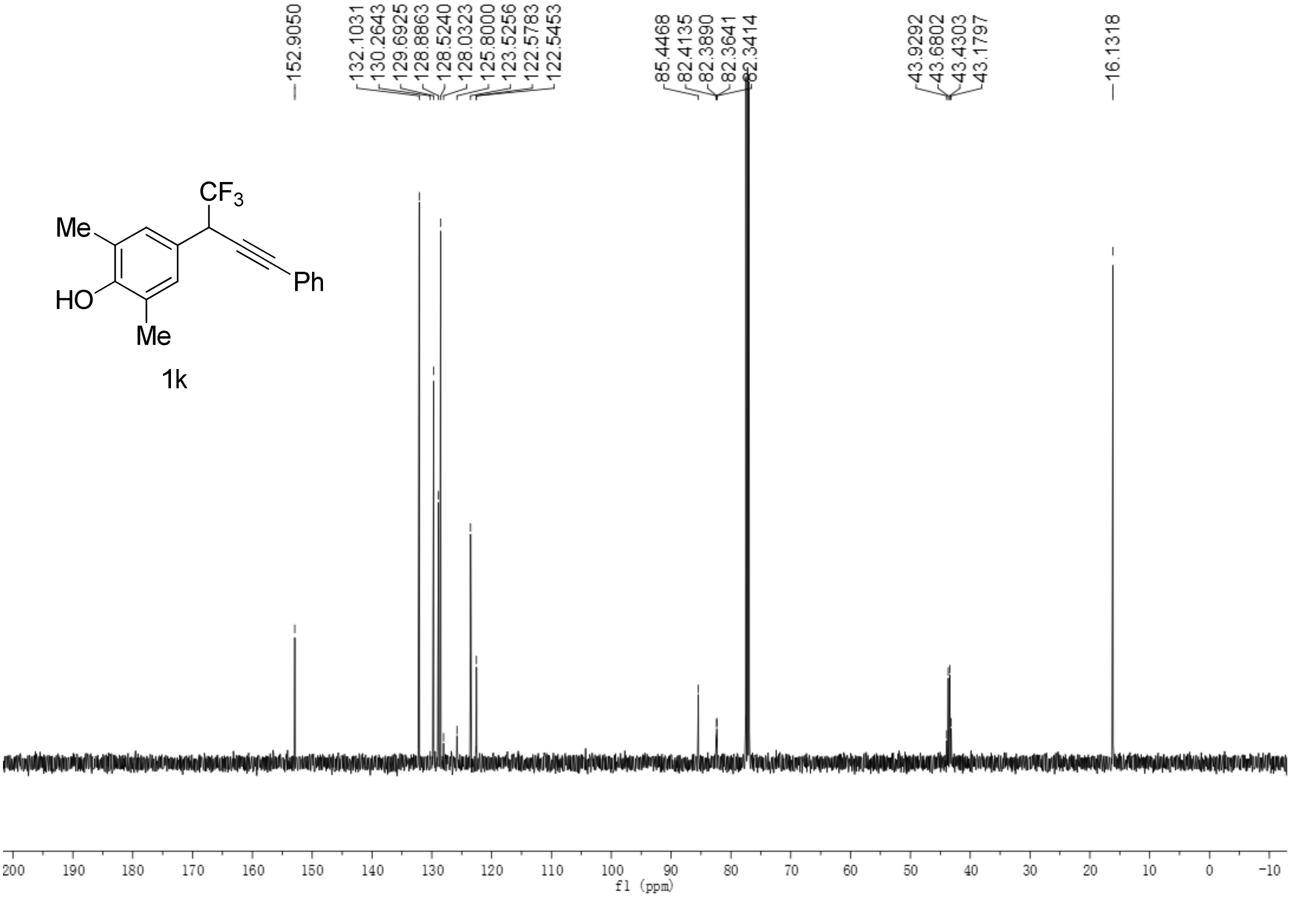
152.9050
132.1031
130.2643
129.6925
128.8863
128.5240
128.0323
125.8000
123.5256
122.5783
122.5453
85.4468
82.4135
82.3890
82.3641
82.3414
43.9292
43.6802
43.4303
43.1797
16.1318
CF3
Me
HO
Me
Ph
1k
f1 (ppm)

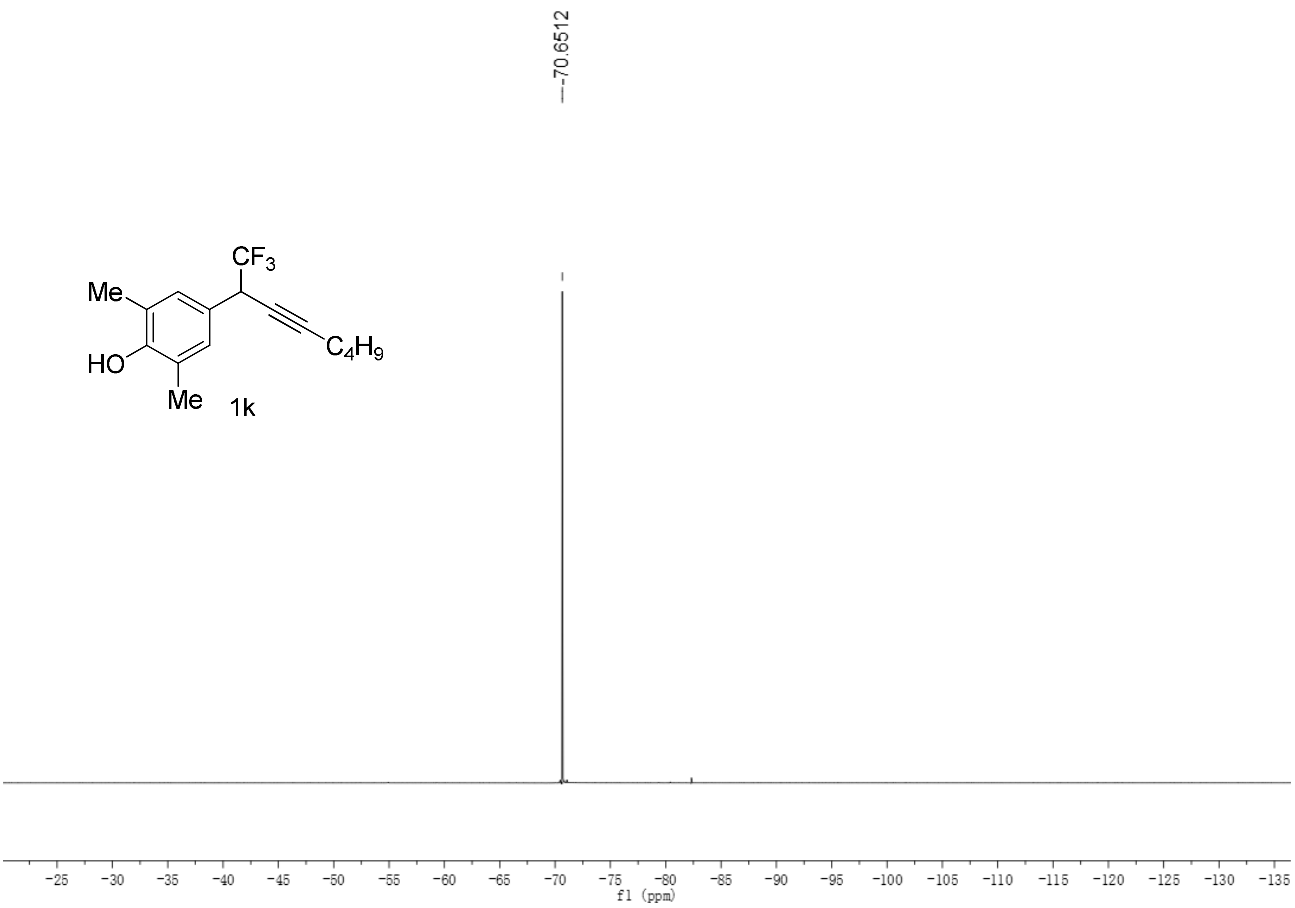

-70.6512
CF3
Me
HO
Me
C4H9
1k
-25 -30 -35 -40 -45 -50 -55 -60 -65 -70 -75 -80 -85 -90 -95 -100 -105 -110 -115 -120 -125 -130 -135
f1 (ppm)

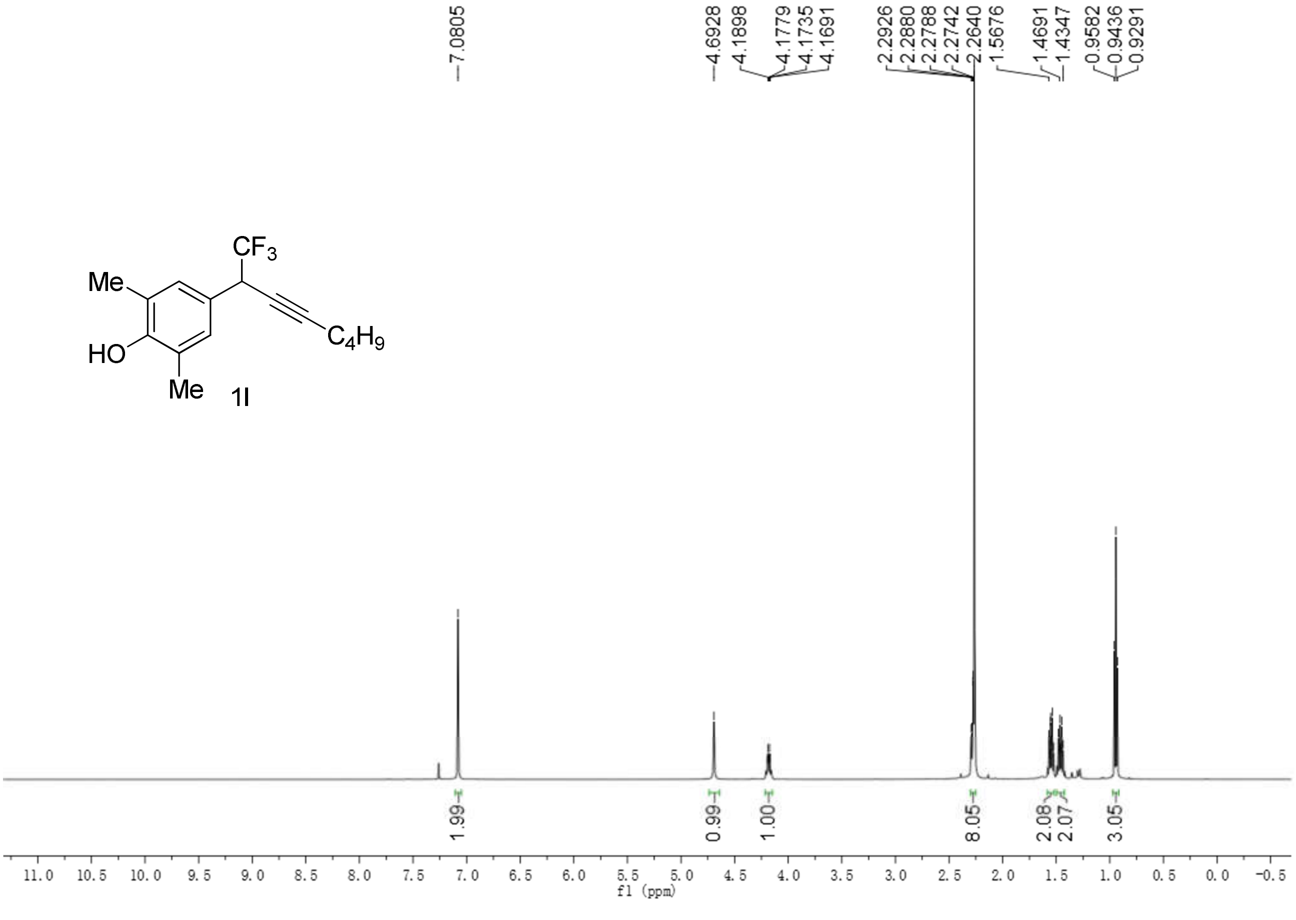

7.0805
4.6928
4.1898
4.1779
4.1735
4.1691
2.2926
2.2880
2.2788
2.2742
2.2640
1.5676
1.4691
1.4347
0.9582
0.9436
0.9291
CF3
Me
HO
Me
C4H9
1l
1.99
0.99
1.00
8.05
2.08
2.07
3.05
11.0 10.5 10.0 9.5 9.0 8.5 8.0 7.5 7.0 6.5 6.0 5.5 5.0 4.5 4.0 3.5 3.0 2.5 2.0 1.5 1.0 0.5 0.0 -0.5
f1 (ppm)

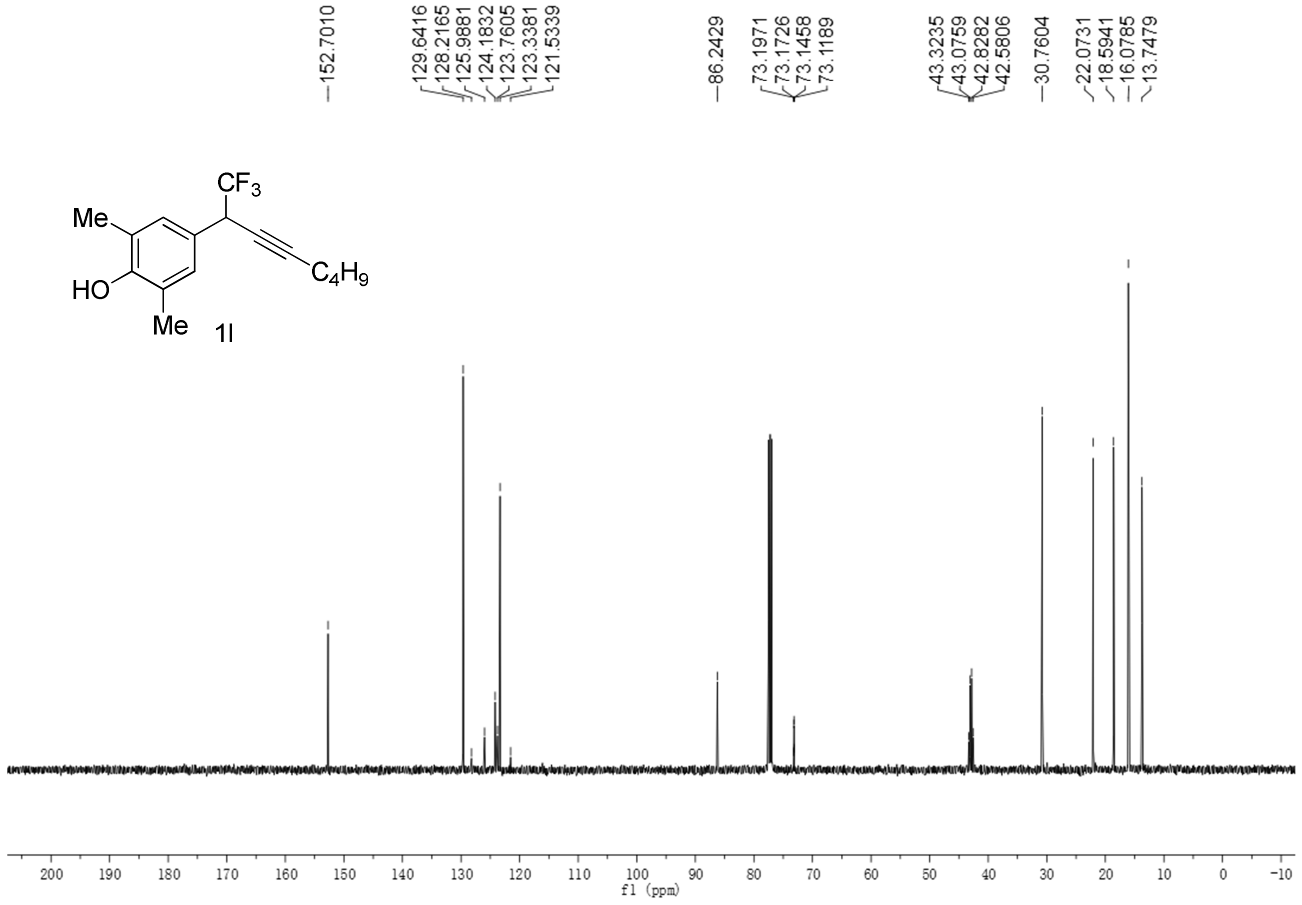
152.7010
129.6416
128.2165
125.9881
124.1832
123.7605
123.3381
121.5339
86.2429
73.1971
73.1726
73.1458
73.1189
43.3235
43.0759
42.8282
42.5806
30.7604
22.0731
18.5941
16.0785
13.7479
Me
CF3
HO
C4H9
Me
1l
f1 (ppm)

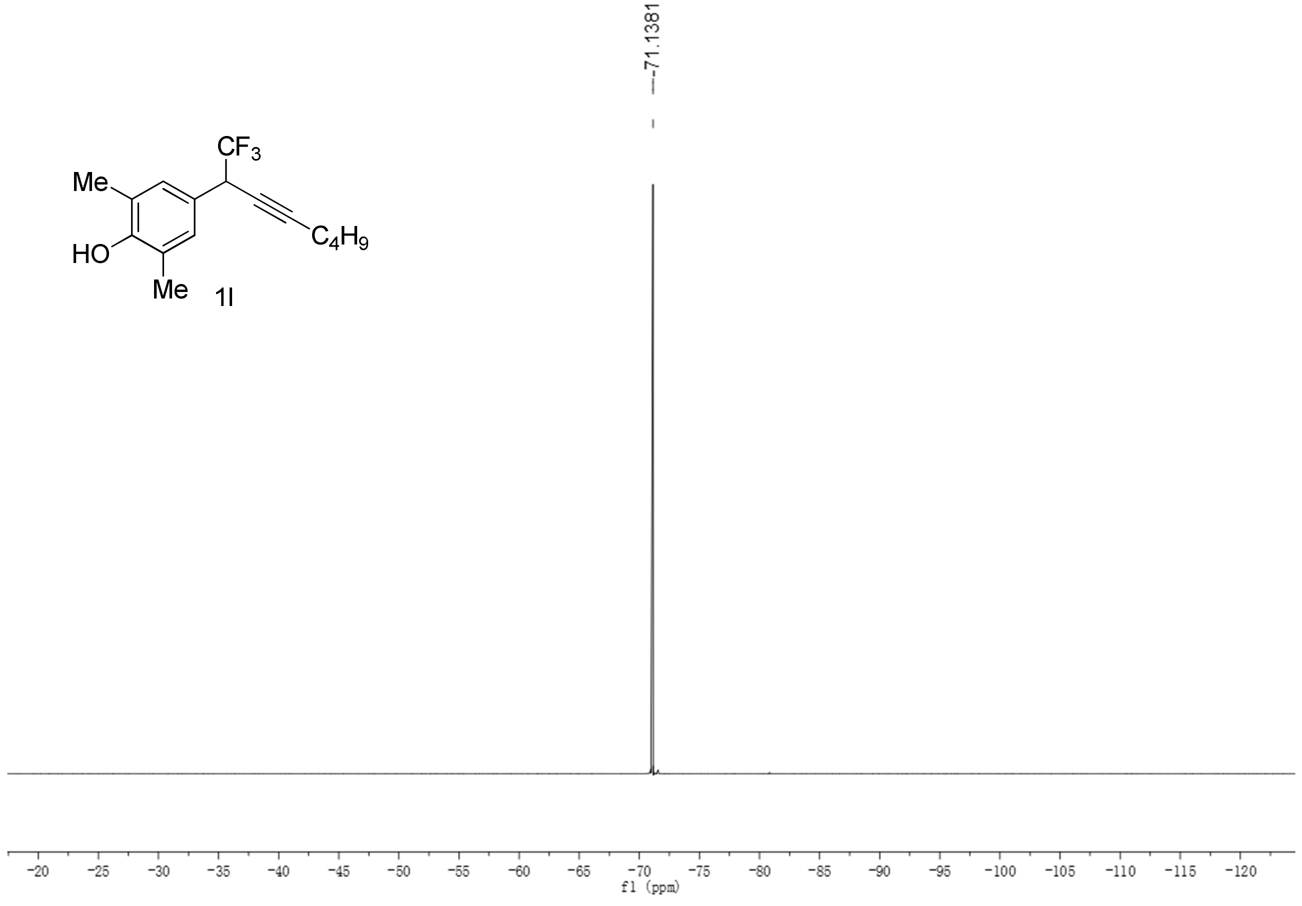
-71.1381
Me
CF3
HO
C4H9
Me
1l
f1 (ppm)

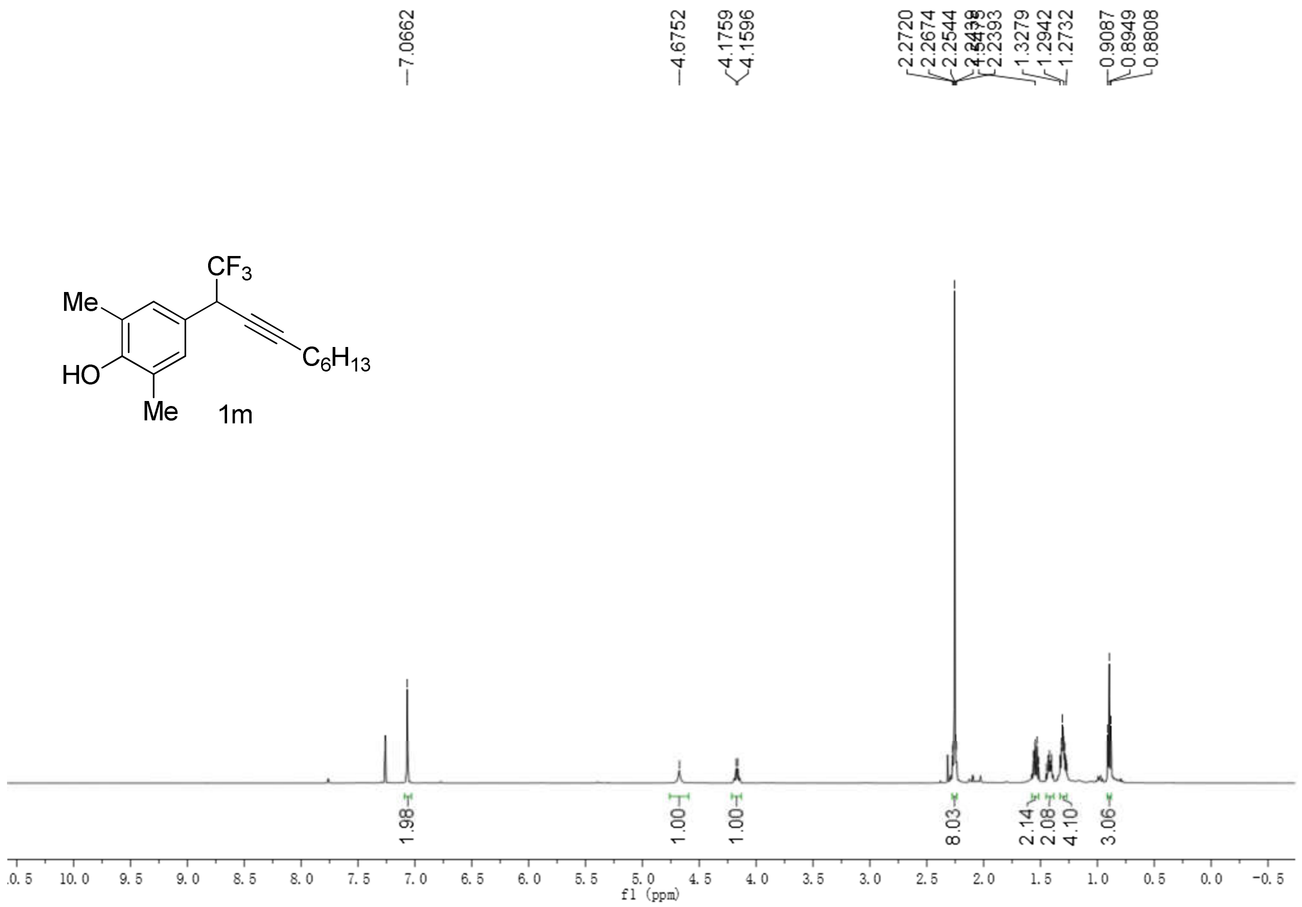

7.0662
4.6752
4.1759
4.1596
2.2720
2.2674
2.2544
2.2393
1.3279
1.2942
1.2732
0.9087
0.8949
0.8808
CF3
Me
HO
Me
C6H13
1m
1.98
1.00
1.00
8.03
2.14
2.08
4.10
3.06
f1 (ppm)

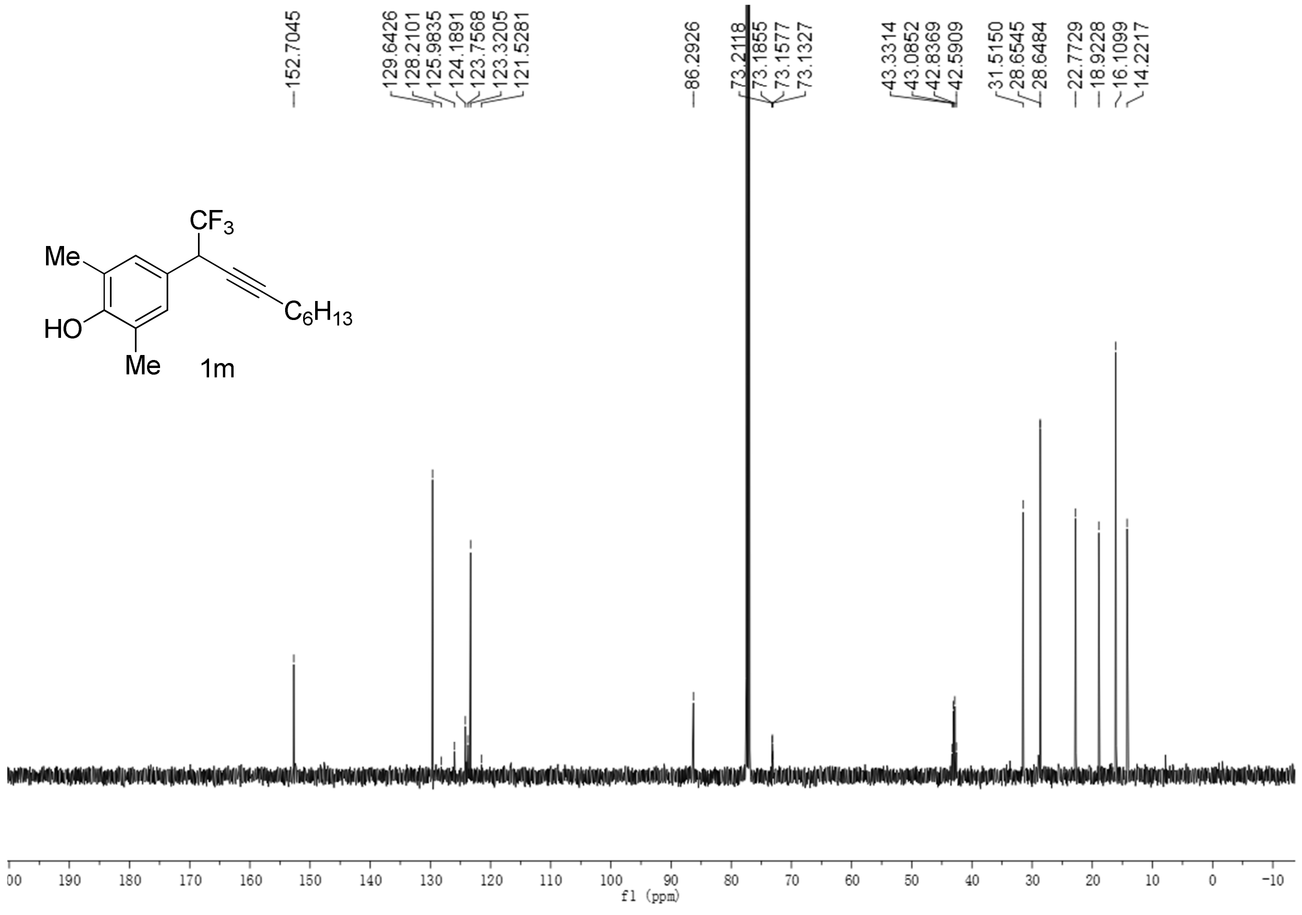

152.7045
129.6426
128.2101
125.9835
124.1891
123.7568
123.3205
121.5281
86.2926
73.2118
73.1855
73.1577
73.1327
43.3314
43.0852
42.8369
42.5909
31.5150
28.6545
28.6484
22.7729
18.9228
16.1099
14.2217
CF3
Me
HO
Me
C6H13
1m
f1 (ppm)

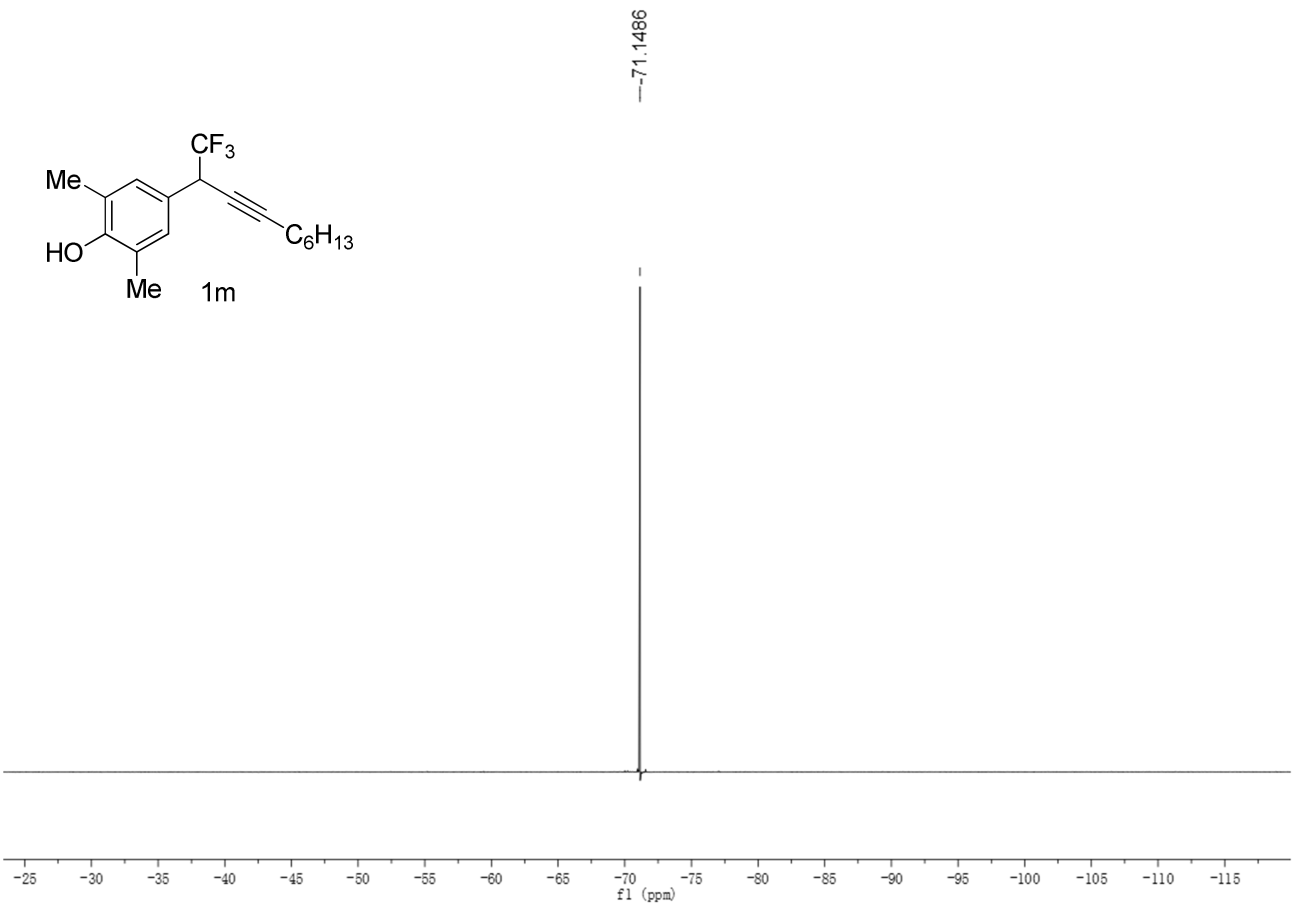

CF3
Me
HO
Me
C6H13
1m
-71.1486
-25 -30 -35 -40 -45 -50 -55 -60 -65 -70 -75 -80 -85 -90 -95 -100 -105 -110 -115
f1 (ppm)

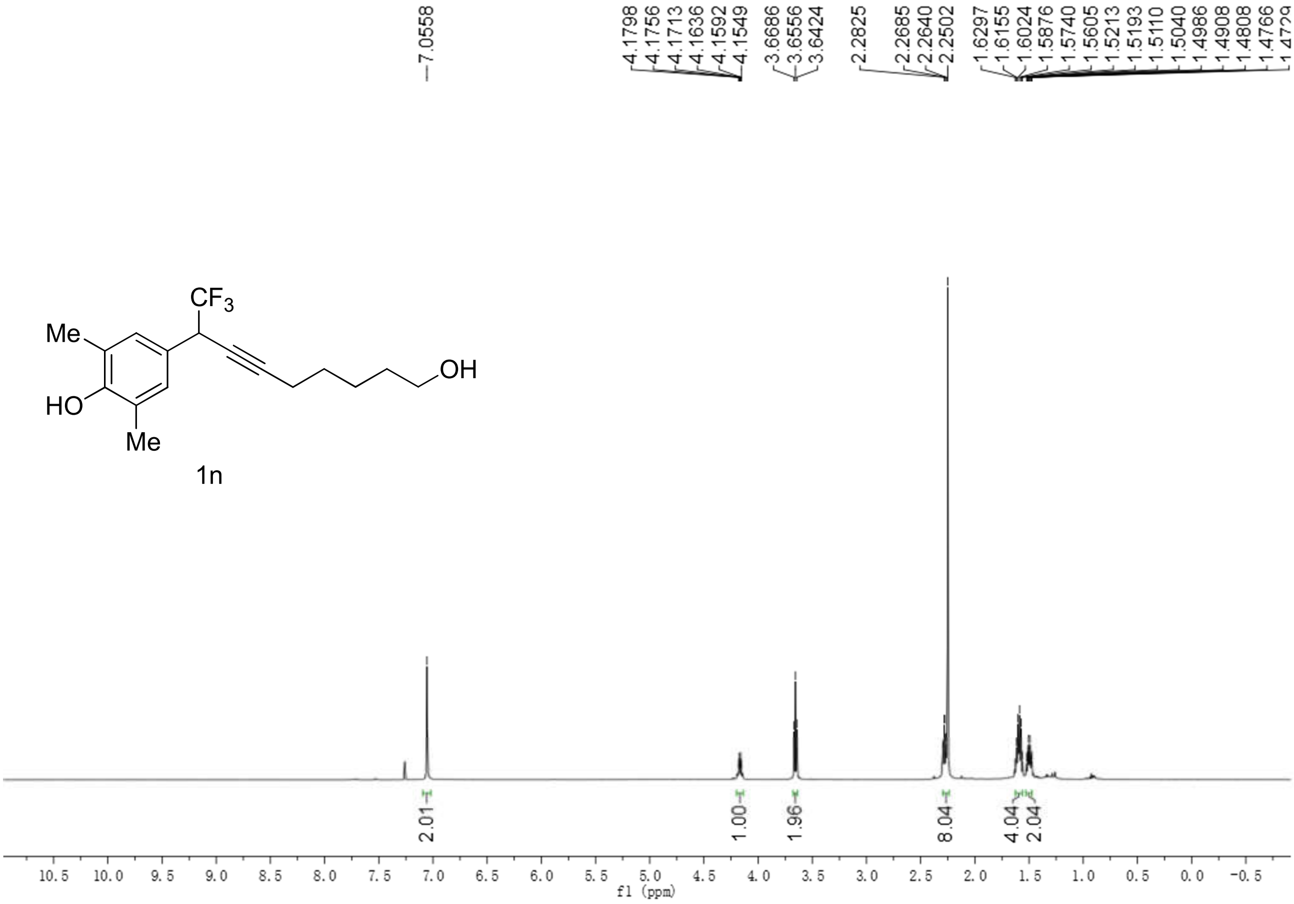

7.0558
4.1798
4.1756
4.1713
4.1636
4.1592
4.1549
3.6686
3.6556
3.6424
2.2825
2.2685
2.2640
2.2502
1.6297
1.6155
1.6024
1.5876
1.5740
1.5605
1.5213
1.5193
1.5110
1.5040
1.4986
1.4908
1.4808
1.4766
CF3
Me
HO
Me
OH
1n
2.01
1.00
1.96
8.04
4.04
2.04
10.5 10.0 9.5 9.0 8.5 8.0 7.5 7.0 6.5 6.0 5.5 5.0 4.5 4.0 3.5 3.0 2.5 2.0 1.5 1.0 0.5 0.0 -0.5
f1 (ppm)

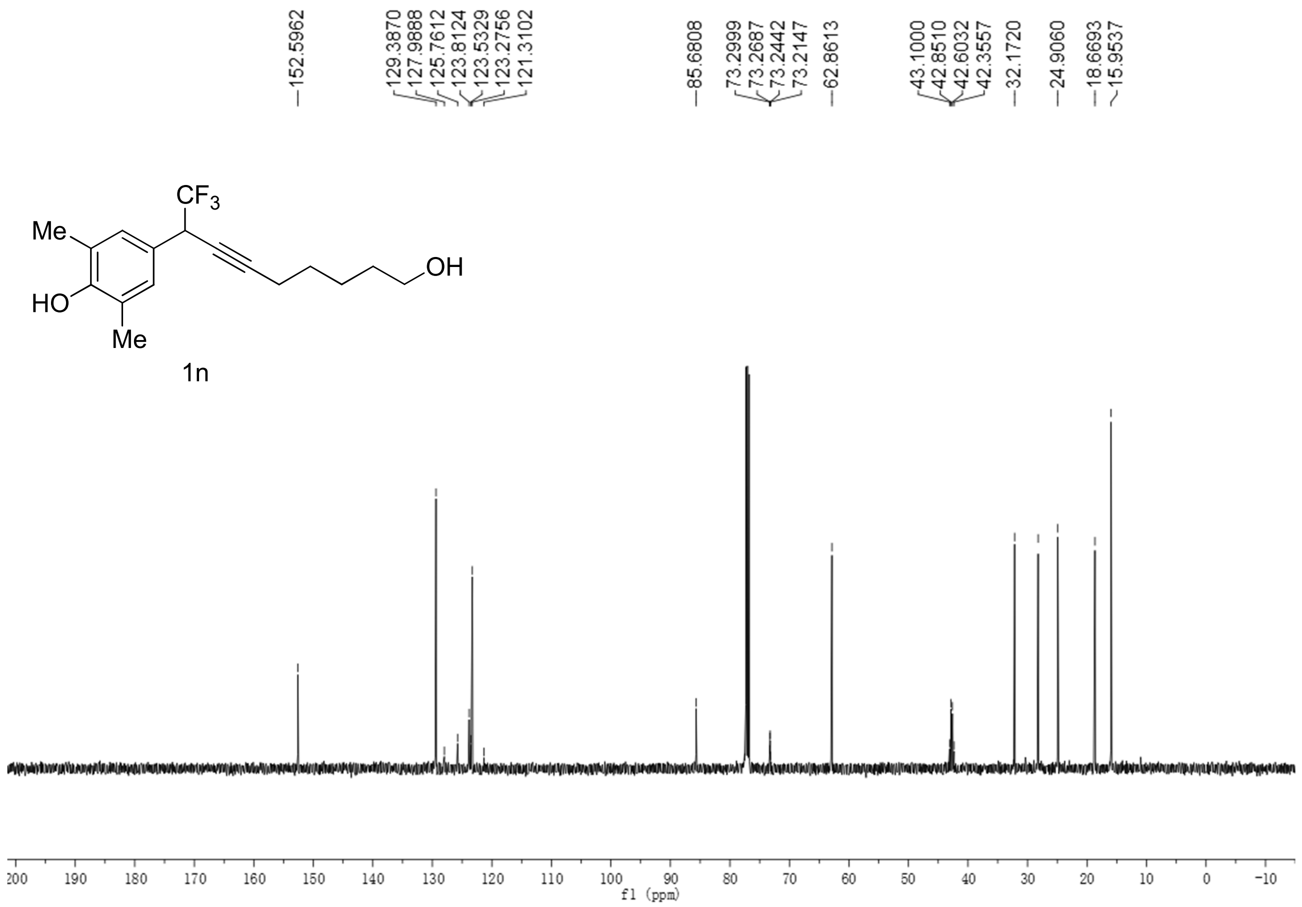
152.5962
129.3870
127.9888
125.7612
123.8124
123.5329
123.2756
121.3102
85.6808
73.2999
73.2687
73.2442
73.2147
62.8613
43.1000
42.8510
42.6032
42.3557
32.1720
24.9060
18.6693
15.9537
CF3
Me
HO
Me
OH
1n
f1 (ppm)

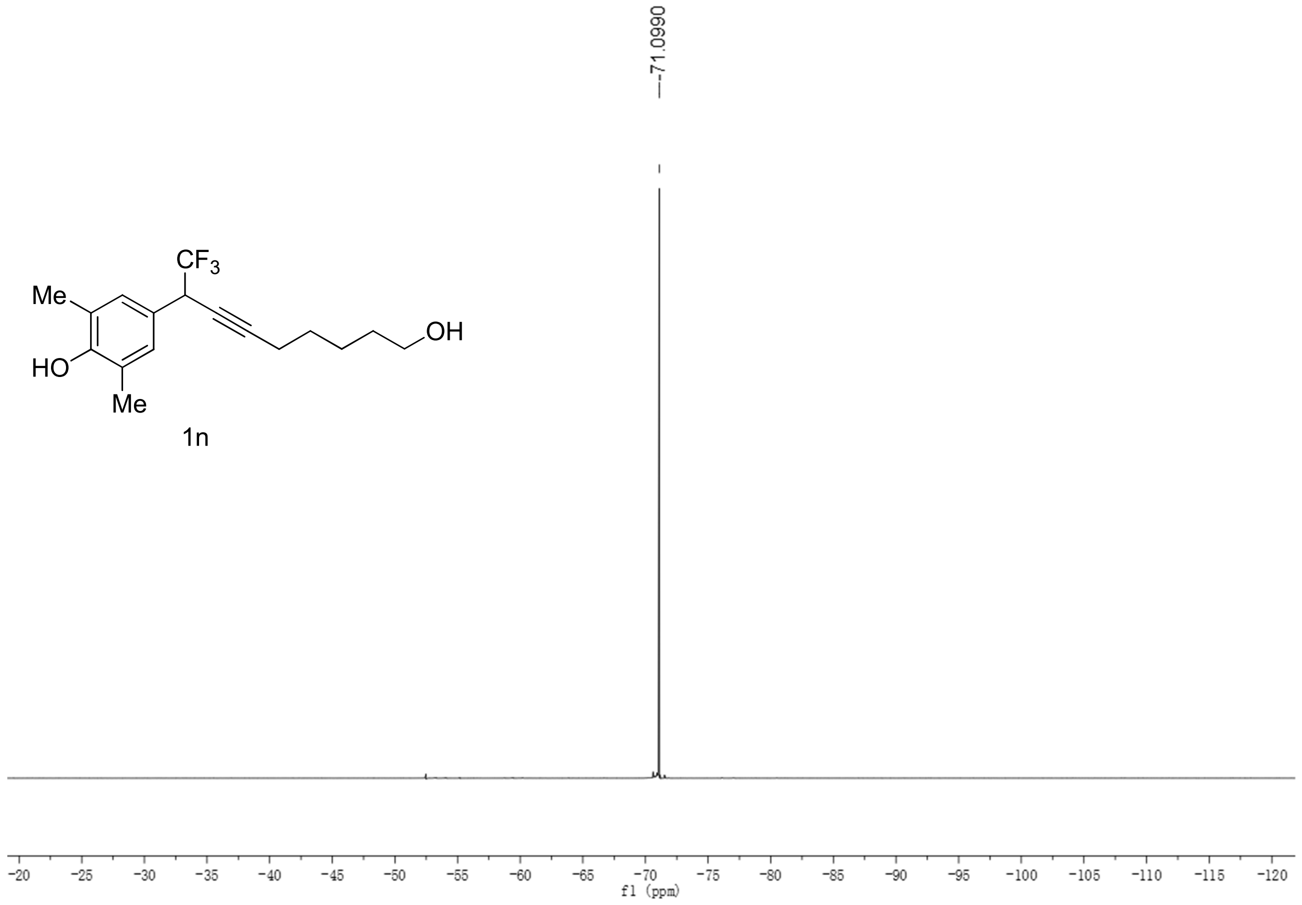
-71.0990
CF3
Me
HO
Me
OH
1n
f1 (ppm)

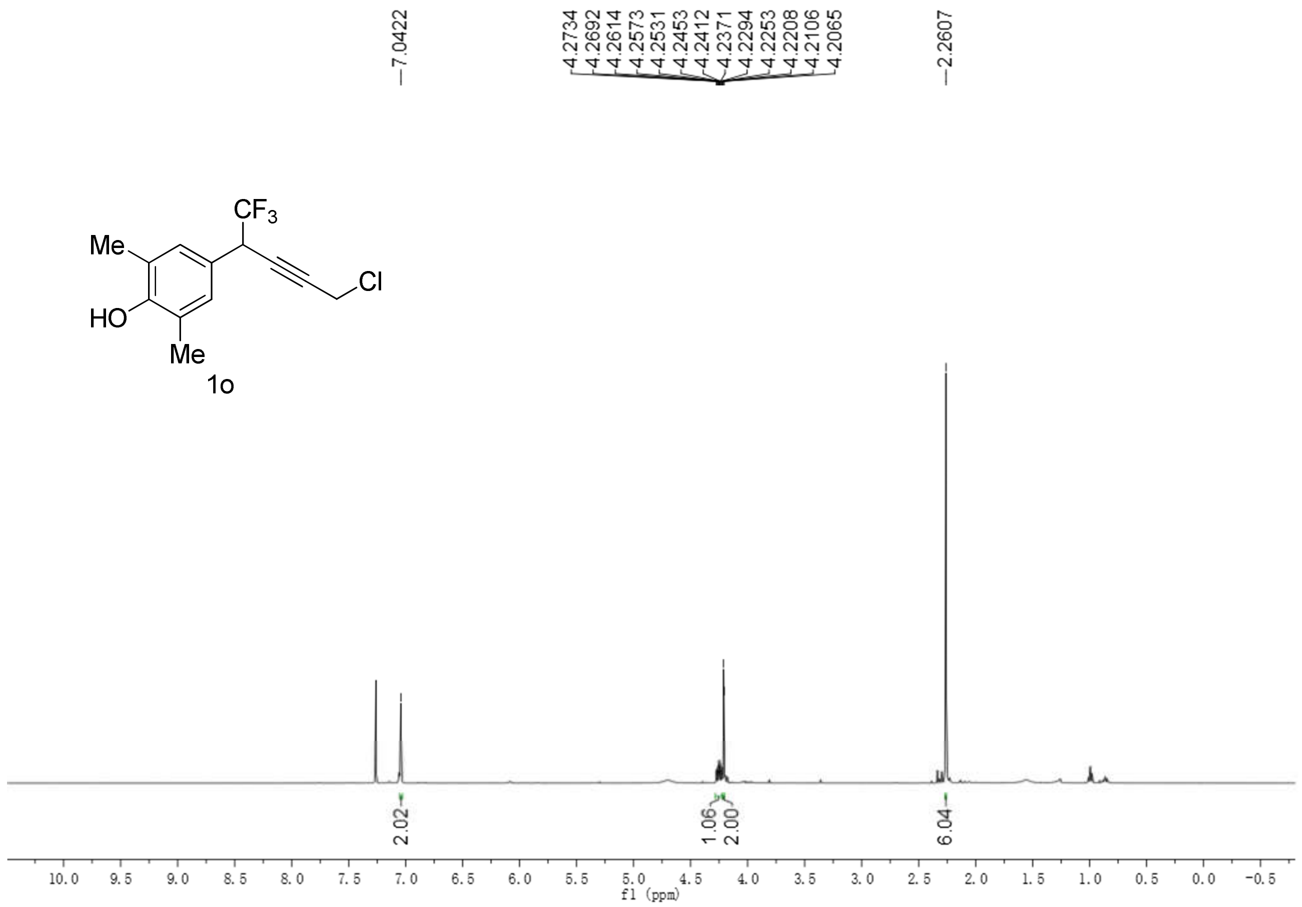
7.0422
4.2734
4.2692
4.2614
4.2573
4.2531
4.2453
4.2412
4.2371
4.2294
4.2253
4.2208
4.2106
4.2065
2.2607
CF_3
Me
HO
Me
Cl
1o
2.02
1.06
2.00
6.04
10.0 9.5 9.0 8.5 8.0 7.5 7.0 6.5 6.0 5.5 5.0 4.5 4.0 3.5 3.0 2.5 2.0 1.5 1.0 0.5 0.0 -0.5
f1 (ppm)

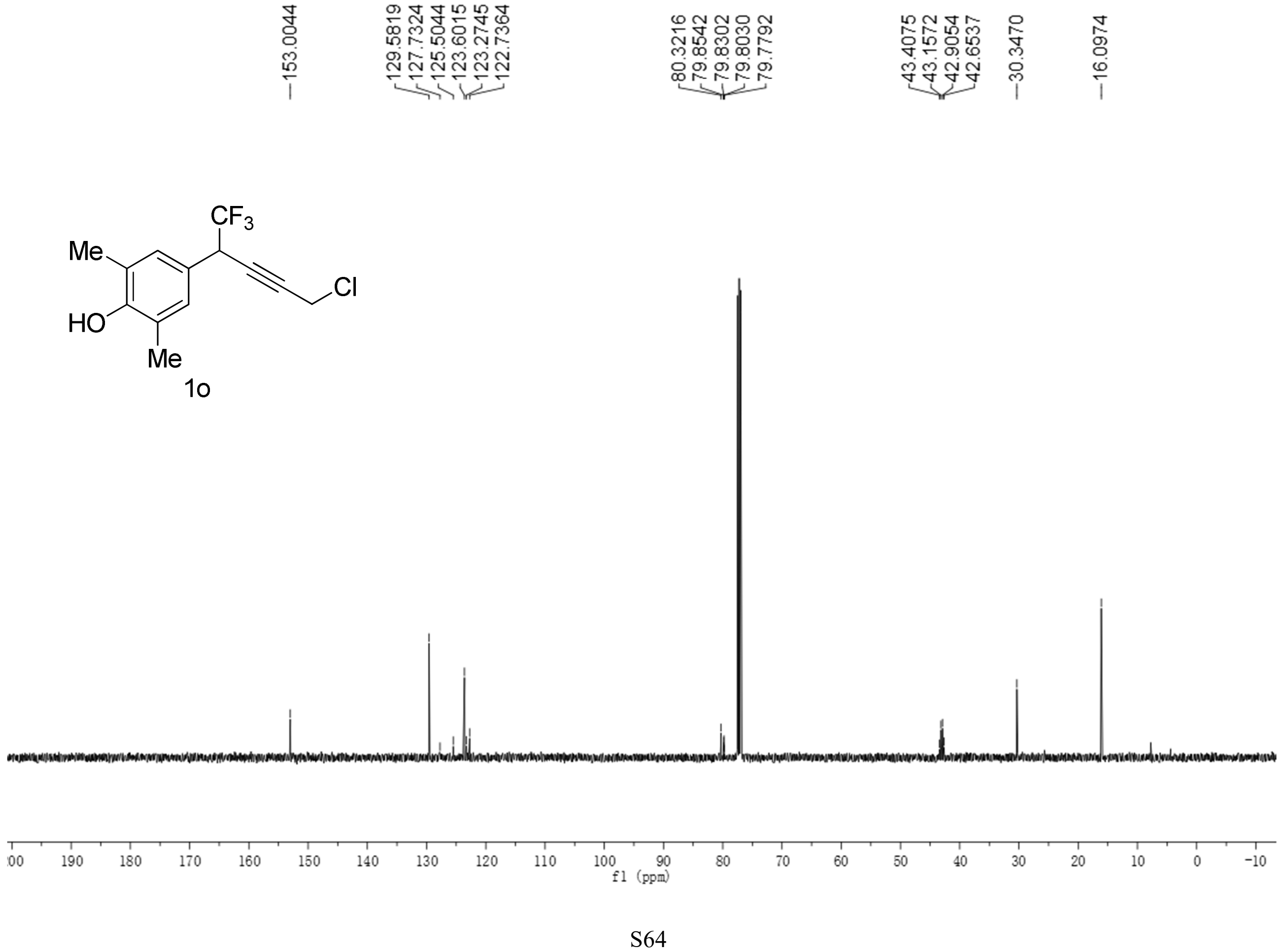
153.0044
129.5819
127.7324
125.5044
123.6015
123.2745
122.7364
80.3216
79.8542
79.8302
79.8030
79.7792
43.4075
43.1572
42.9054
42.6537
30.3470
16.0974
CF_3
Me
HO
Me
Cl
1o
190 180 170 160 150 140 130 120 110 100 90 80 70 60 50 40 30 20 10 0 -10
f1 (ppm)

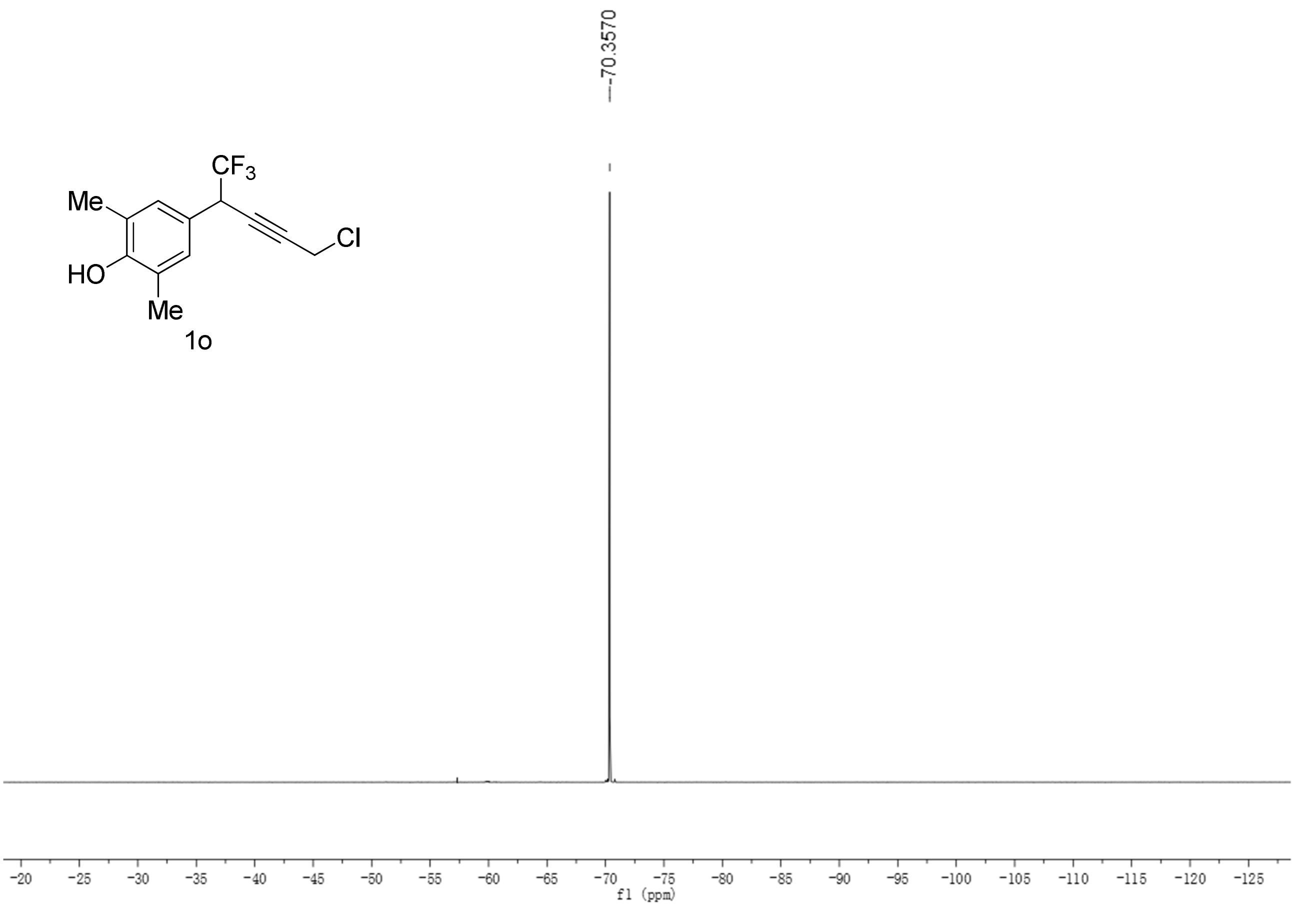
-70.3570
CF3
Me
HO
Me
Cl
1o
-20 -25 -30 -35 -40 -45 -50 -55 -60 -65 -70 -75 -80 -85 -90 -95 -100 -105 -110 -115 -120 -125
f1 (ppm)

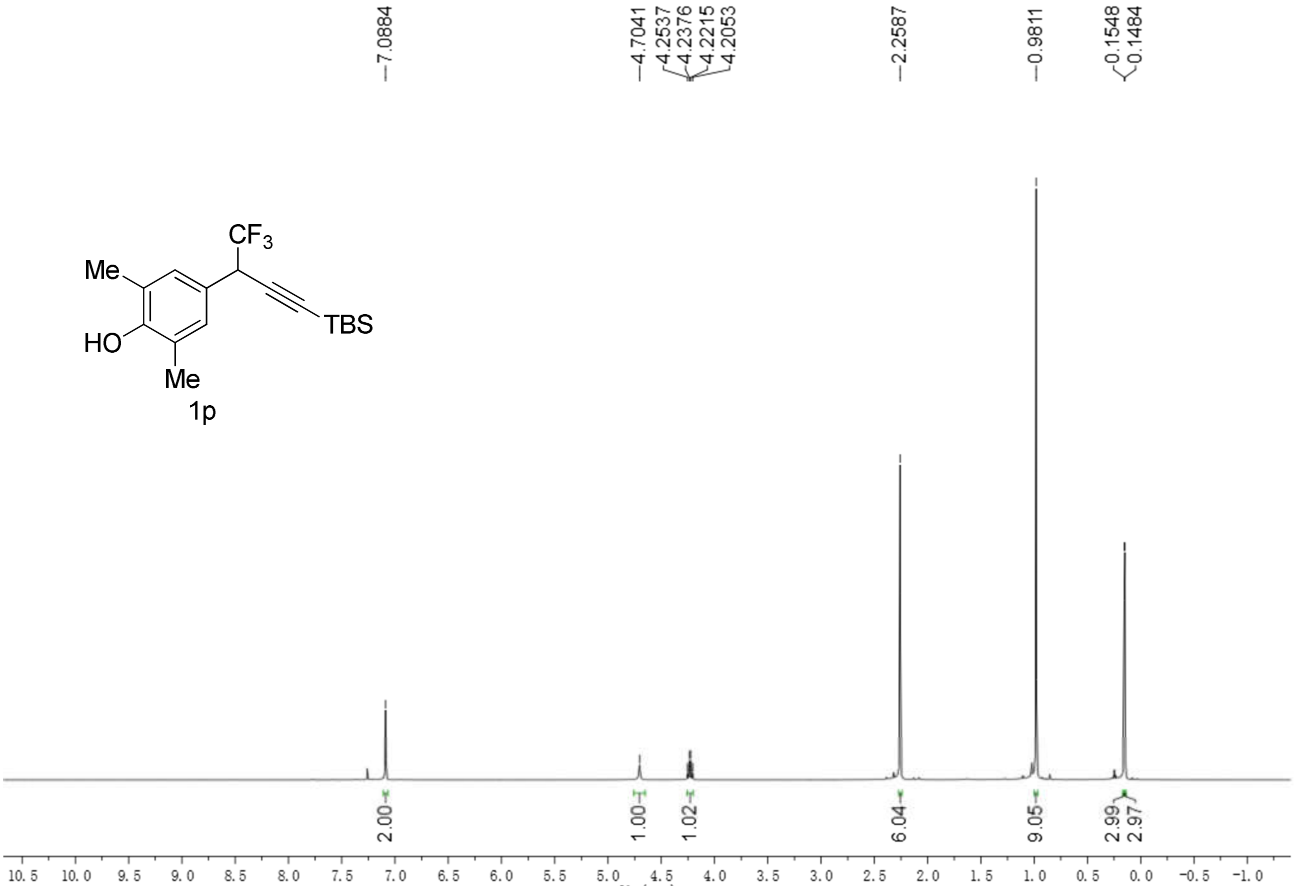
7.0884
4.7041
4.2537
4.2376
4.2215
4.2053
2.2587
0.9811
0.1548
0.1484
CF3
Me
HO
Me
TBS
1p
2.00
1.00
1.02
6.04
9.05
2.99
2.97
10.5 10.0 9.5 9.0 8.5 8.0 7.5 7.0 6.5 6.0 5.5 5.0 4.5 4.0 3.5 3.0 2.5 2.0 1.5 1.0 0.5 0.0 -0.5 -1.0
f1 (ppm)

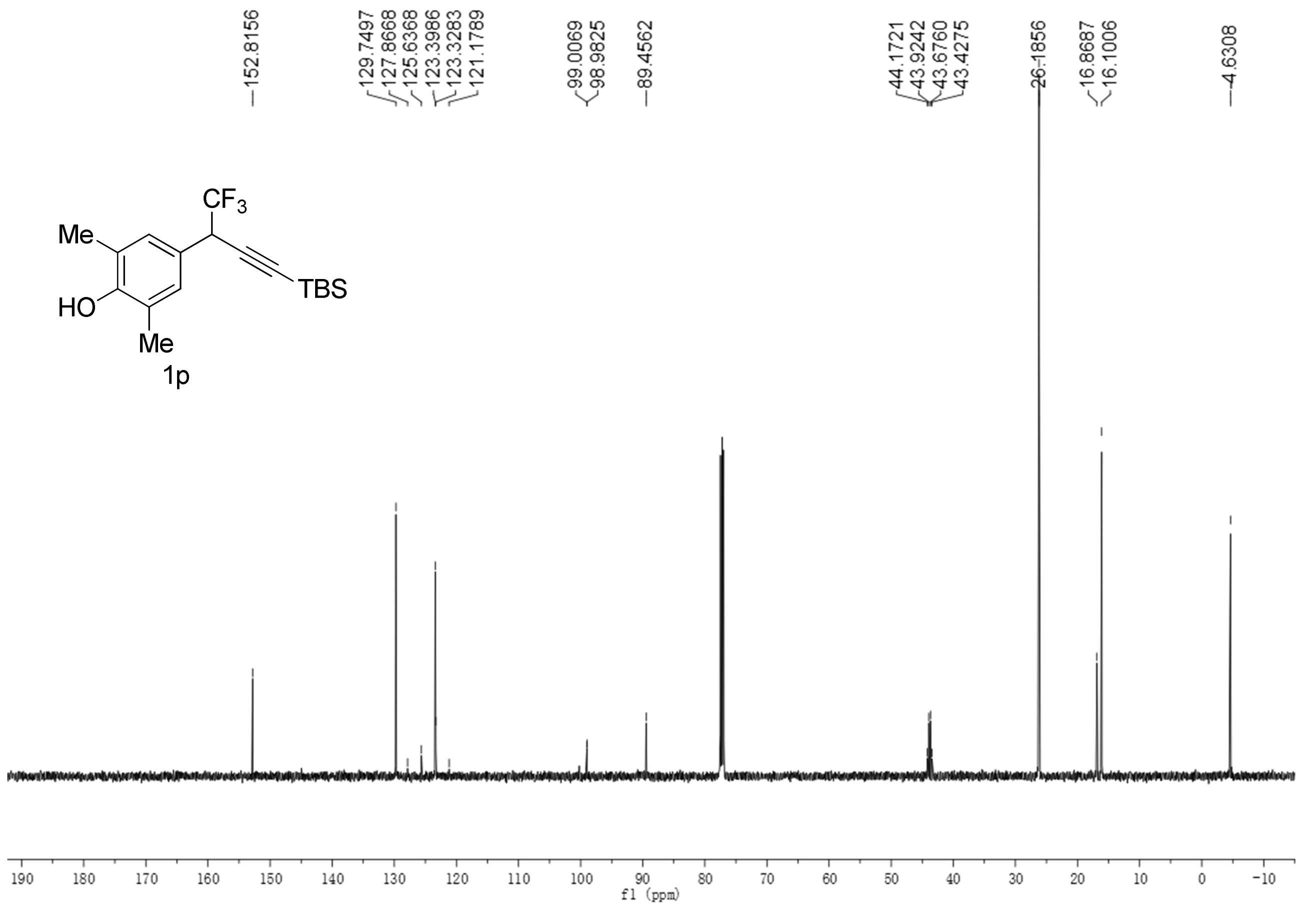

152.8156
129.7497
127.8668
125.6368
123.3986
123.3283
121.1789
99.0069
98.9825
89.4562
44.1721
43.9242
43.6760
43.4275
26.1856
16.8687
16.1006
-4.6308
CF3
Me
HO
Me
TBS
1p
f1 (ppm)

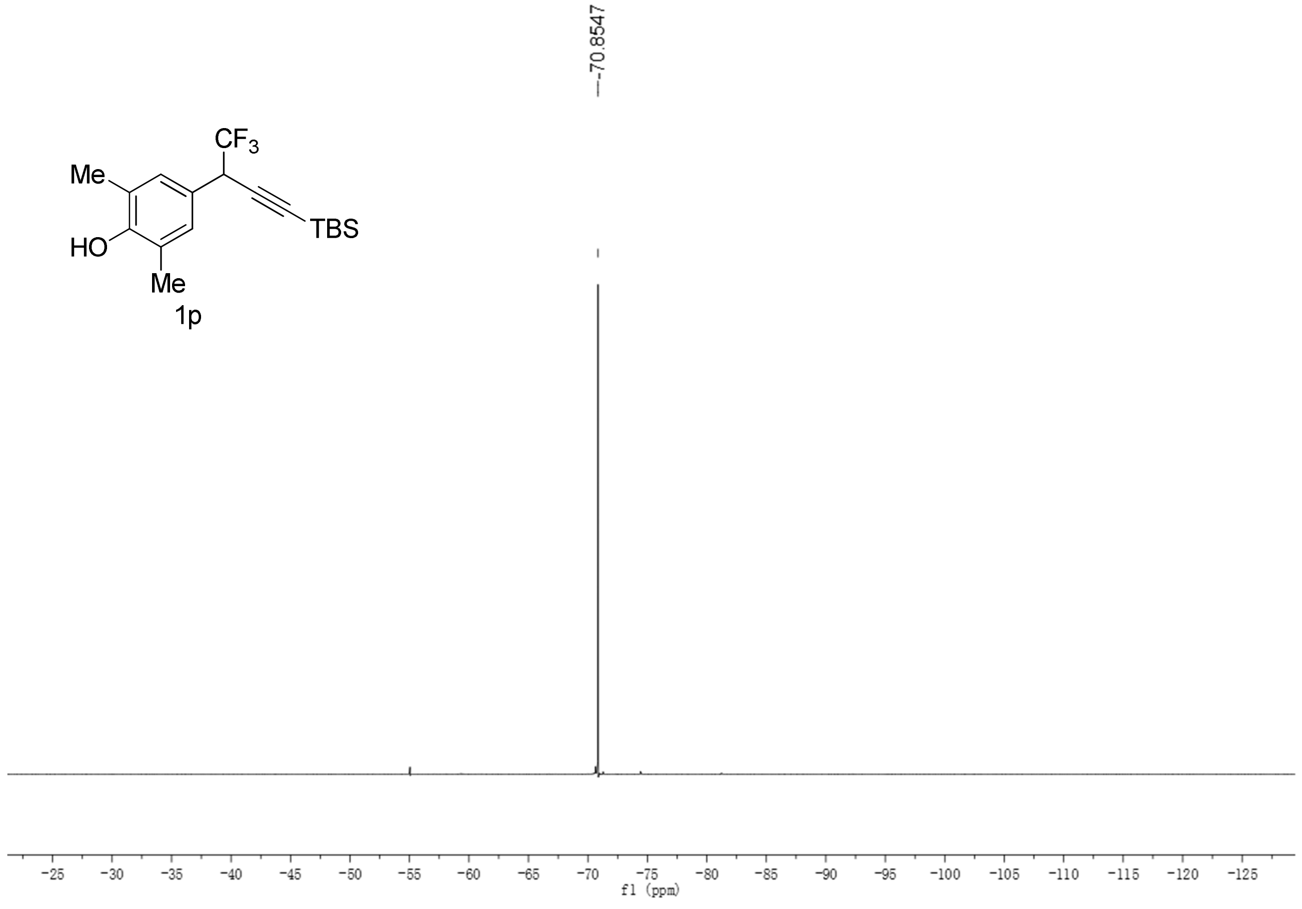

-70.8547
CF3
Me
HO
Me
TBS
1p
f1 (ppm)

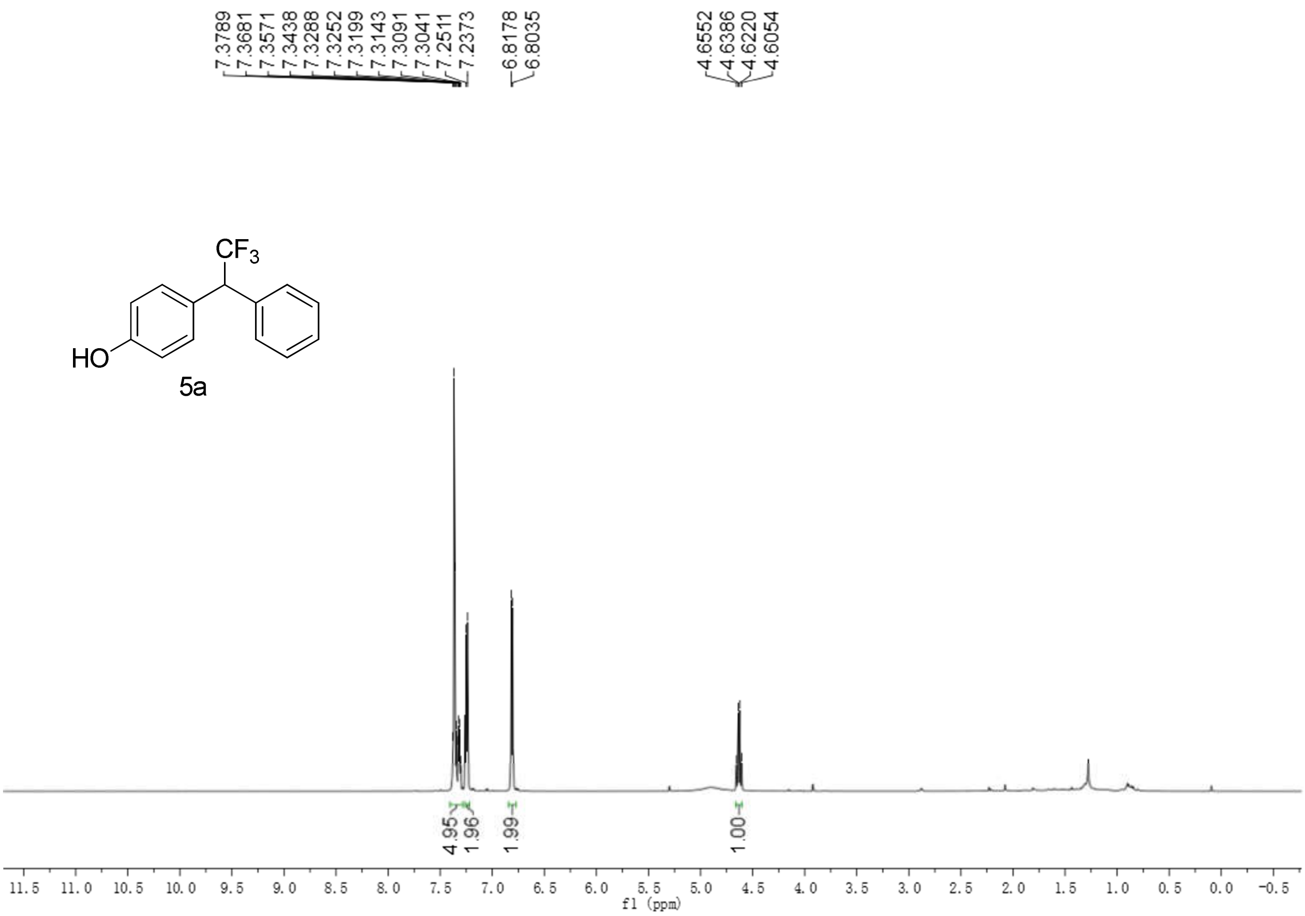

7.3789
7.3681
7.3571
7.3438
7.3288
7.3252
7.3199
7.3143
7.3091
7.3041
7.2511
7.2373
6.8178
6.8035
4.6552
4.6386
4.6220
4.6054
CF3
HO
5a
4.95
1.96
1.99
1.00
11.5 11.0 10.5 10.0 9.5 9.0 8.5 8.0 7.5 7.0 6.5 6.0 5.5 5.0 4.5 4.0 3.5 3.0 2.5 2.0 1.5 1.0 0.5 0.0 -0.5
f1 (ppm)

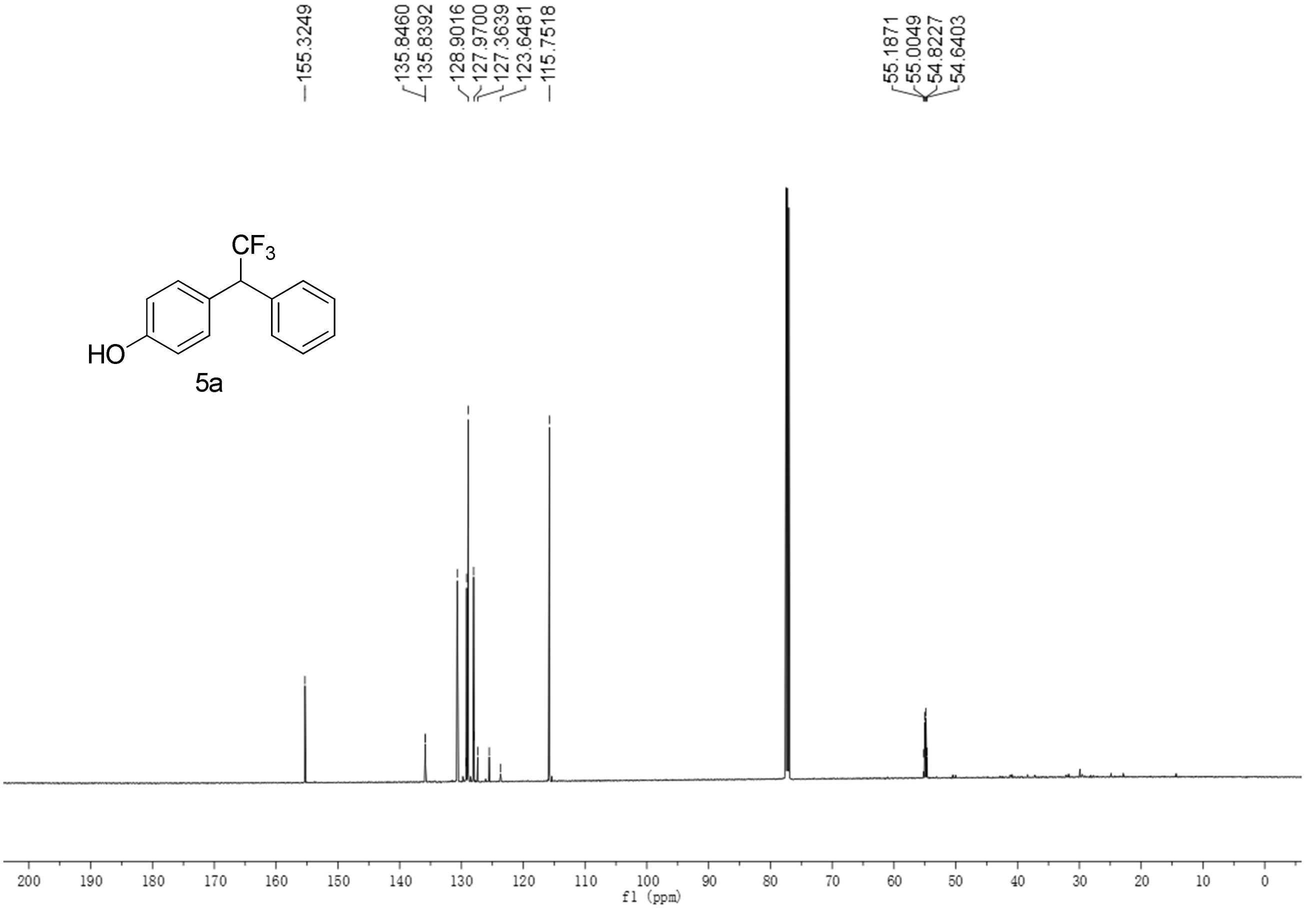

155.3249
135.8460
135.8392
128.9016
127.9700
127.3639
123.6481
115.7518
55.1871
55.0049
54.8227
54.6403
CF3
HO
5a
200 190 180 170 160 150 140 130 120 110 100 90 80 70 60 50 40 30 20 10 0
f1 (ppm)

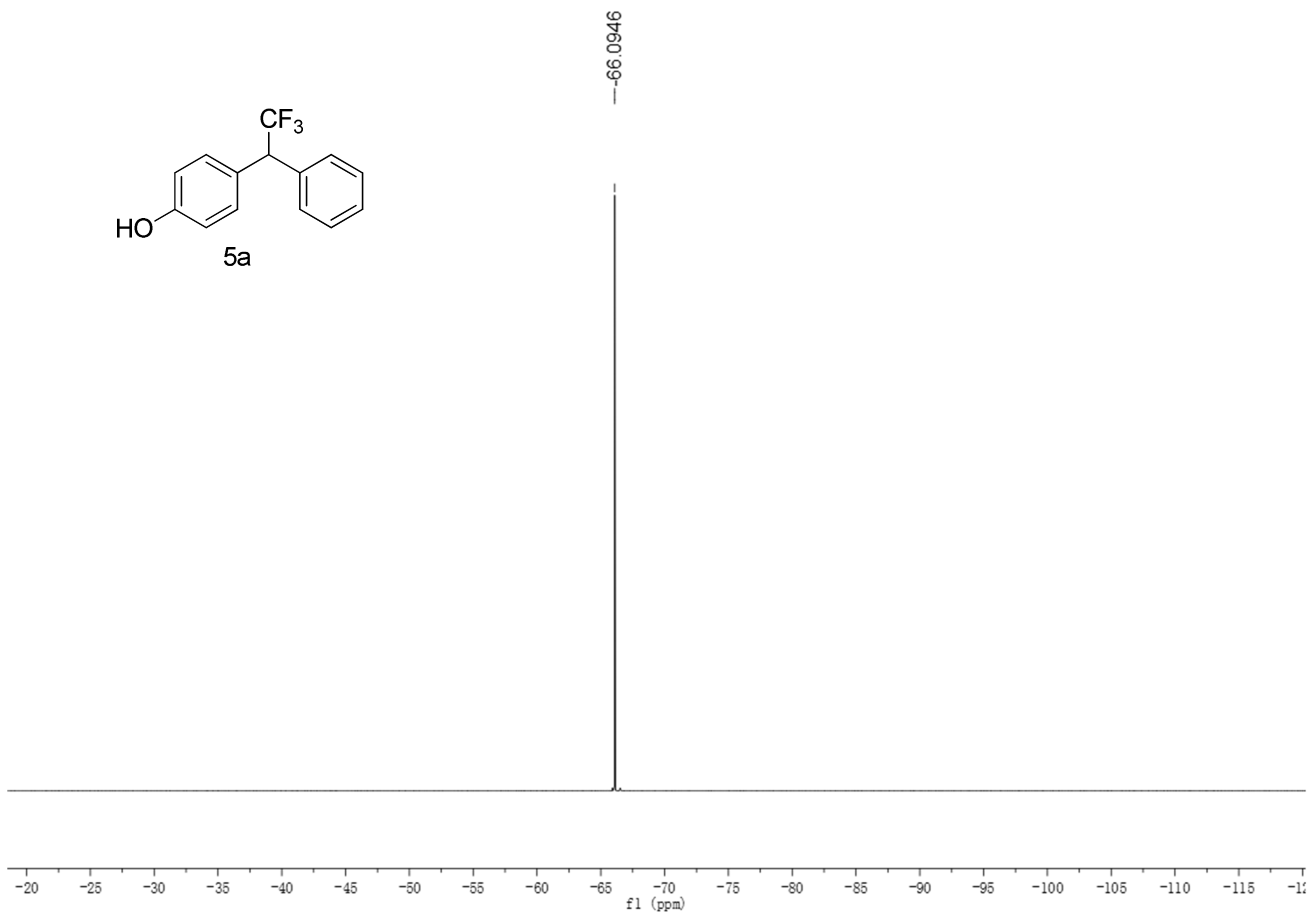
-66.0946
CF3
HO
5a
-20 -25 -30 -35 -40 -45 -50 -55 -60 -65 -70 -75 -80 -85 -90 -95 -100 -105 -110 -115
f1 (ppm)

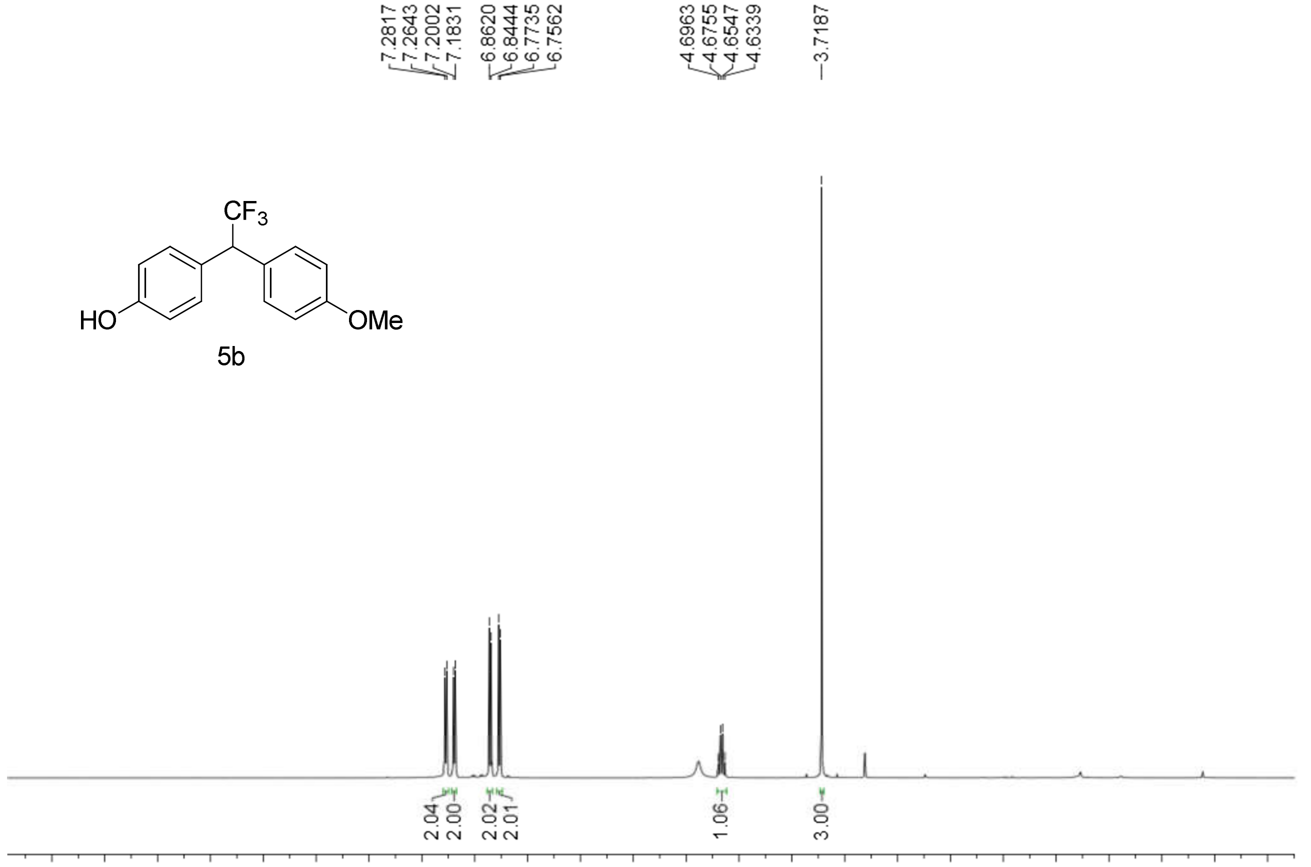
7.2817
7.2643
7.2002
7.1831
6.8620
6.8444
6.7735
6.7562
4.6963
4.6755
4.6547
4.6339
3.7187
CF3
HO
OMe
5b
2.04
2.00
2.02
2.01
1.06
3.00
11.0 10.5 10.0 9.5 9.0 8.5 8.0 7.5 7.0 6.5 6.0 5.5 5.0 4.5 4.0 3.5 3.0 2.5 2.0 1.5 1.0 0.5 0.0 -0.5
f1 (ppm)

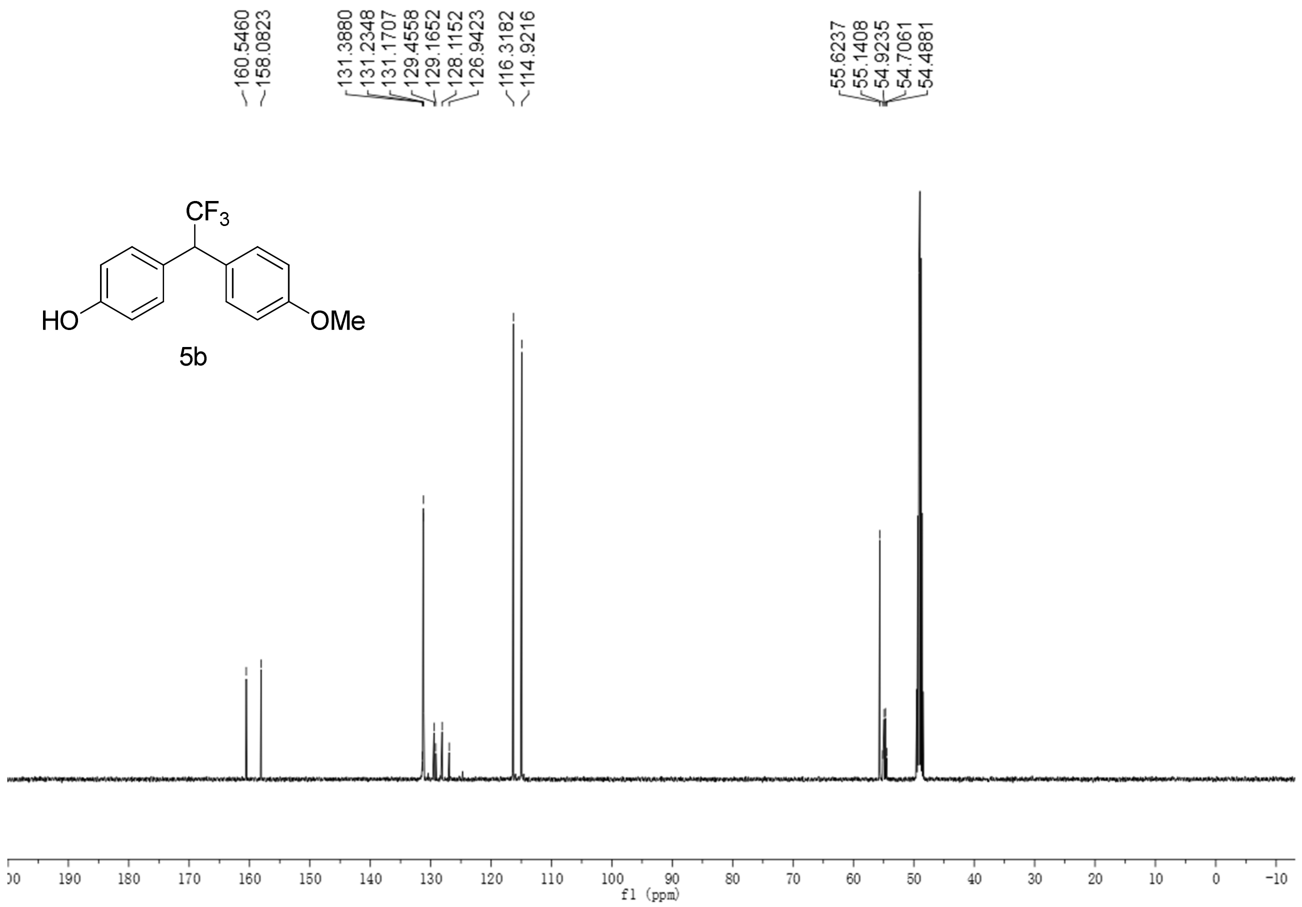
160.5460
158.0823
131.3880
131.2348
131.1707
129.4558
129.1652
128.1152
126.9423
116.3182
114.9216
55.6237
55.1408
54.9235
54.7061
54.4881
CF3
HO
OMe
5b
f1 (ppm)

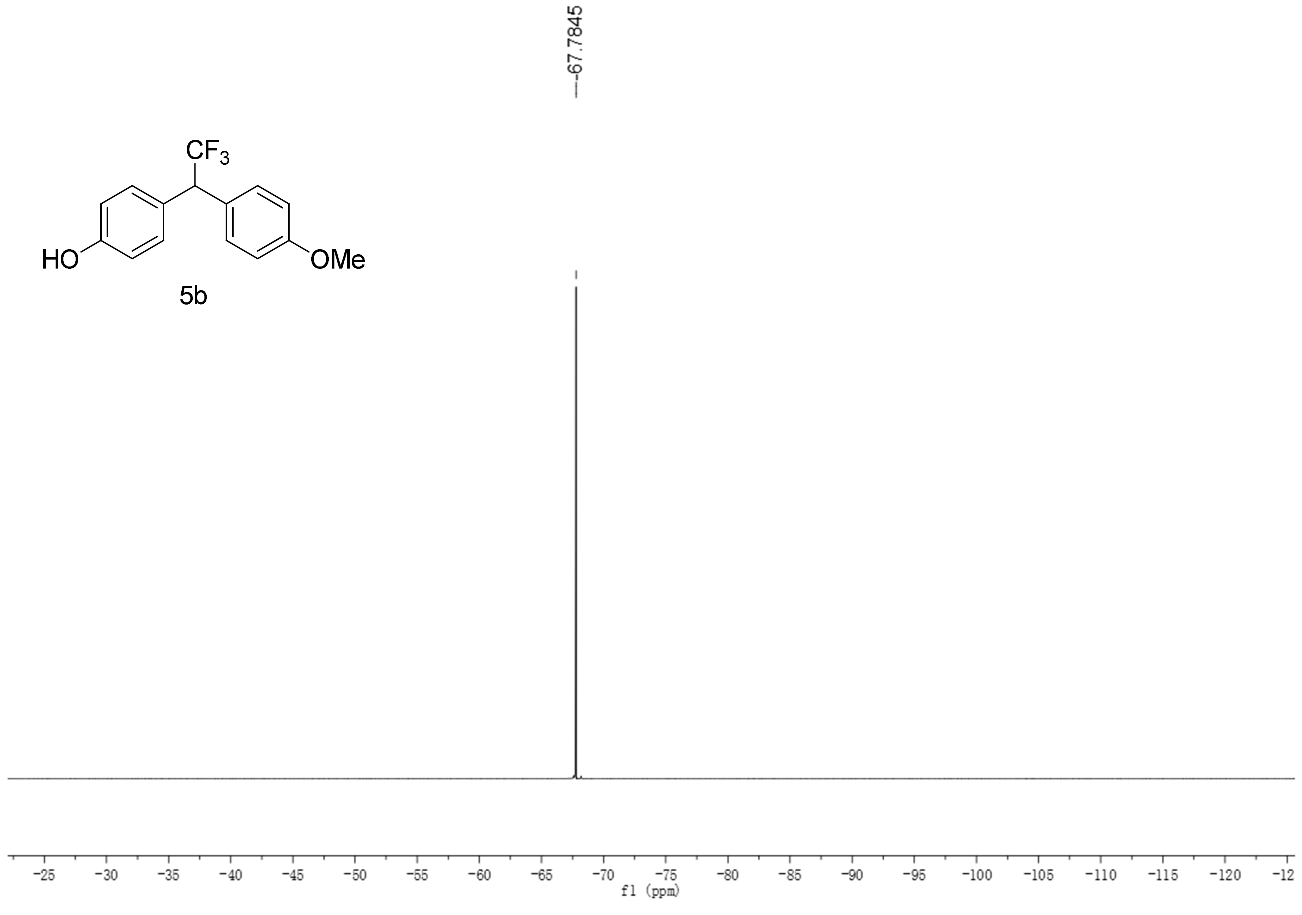
-67.7845
CF3
HO
OMe
5b
f1 (ppm)

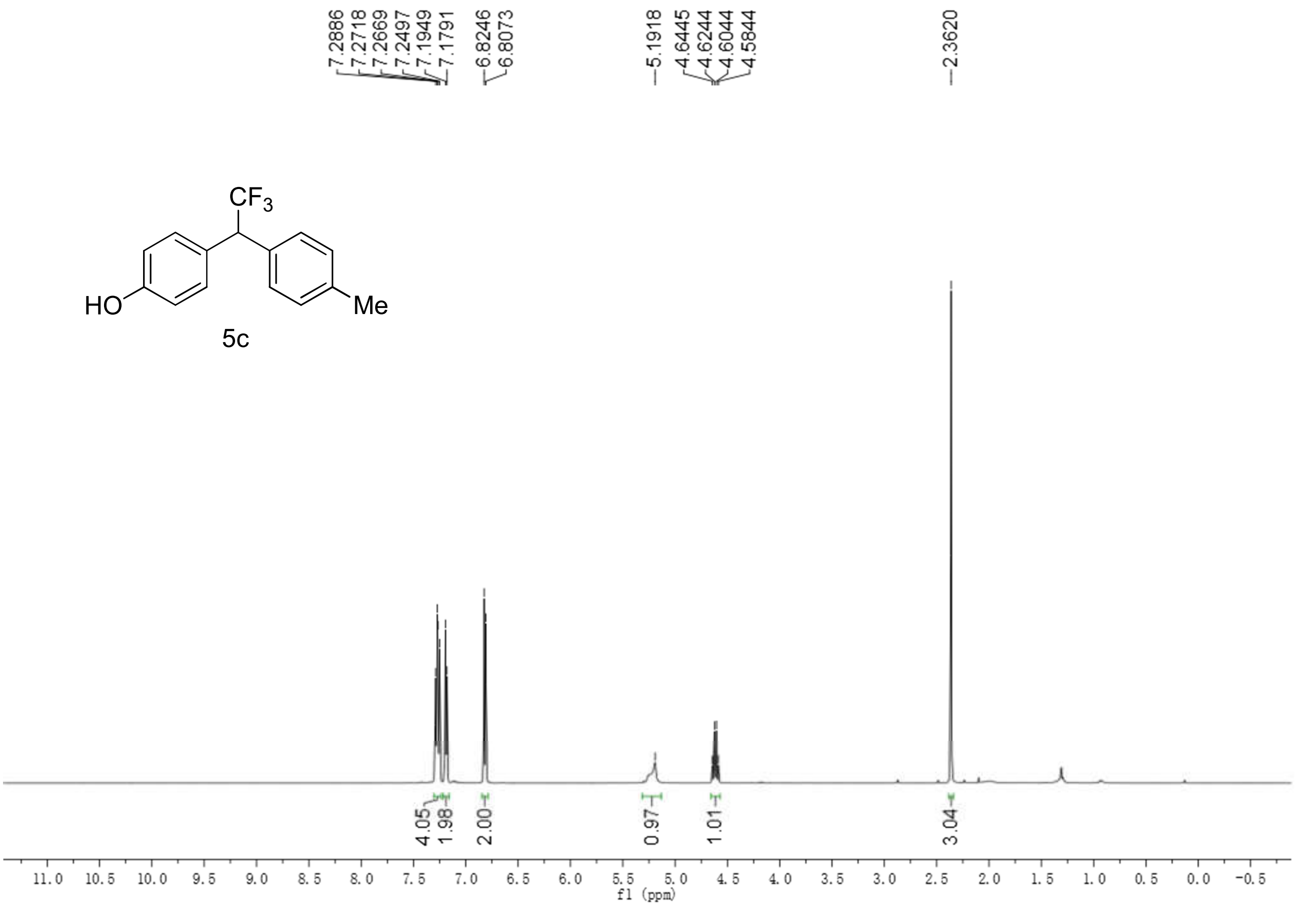
7.2886
7.2718
7.2669
7.2497
7.1949
7.1791
6.8246
6.8073
5.1918
4.6445
4.6244
4.6044
4.5844
2.3620
CF3
HO
Me
5c
4.05
1.98
2.00
0.97
1.01
3.04
11.0 10.5 10.0 9.5 9.0 8.5 8.0 7.5 7.0 6.5 6.0 5.5 5.0 4.5 4.0 3.5 3.0 2.5 2.0 1.5 1.0 0.5 0.0 -0.5
f1 (ppm)

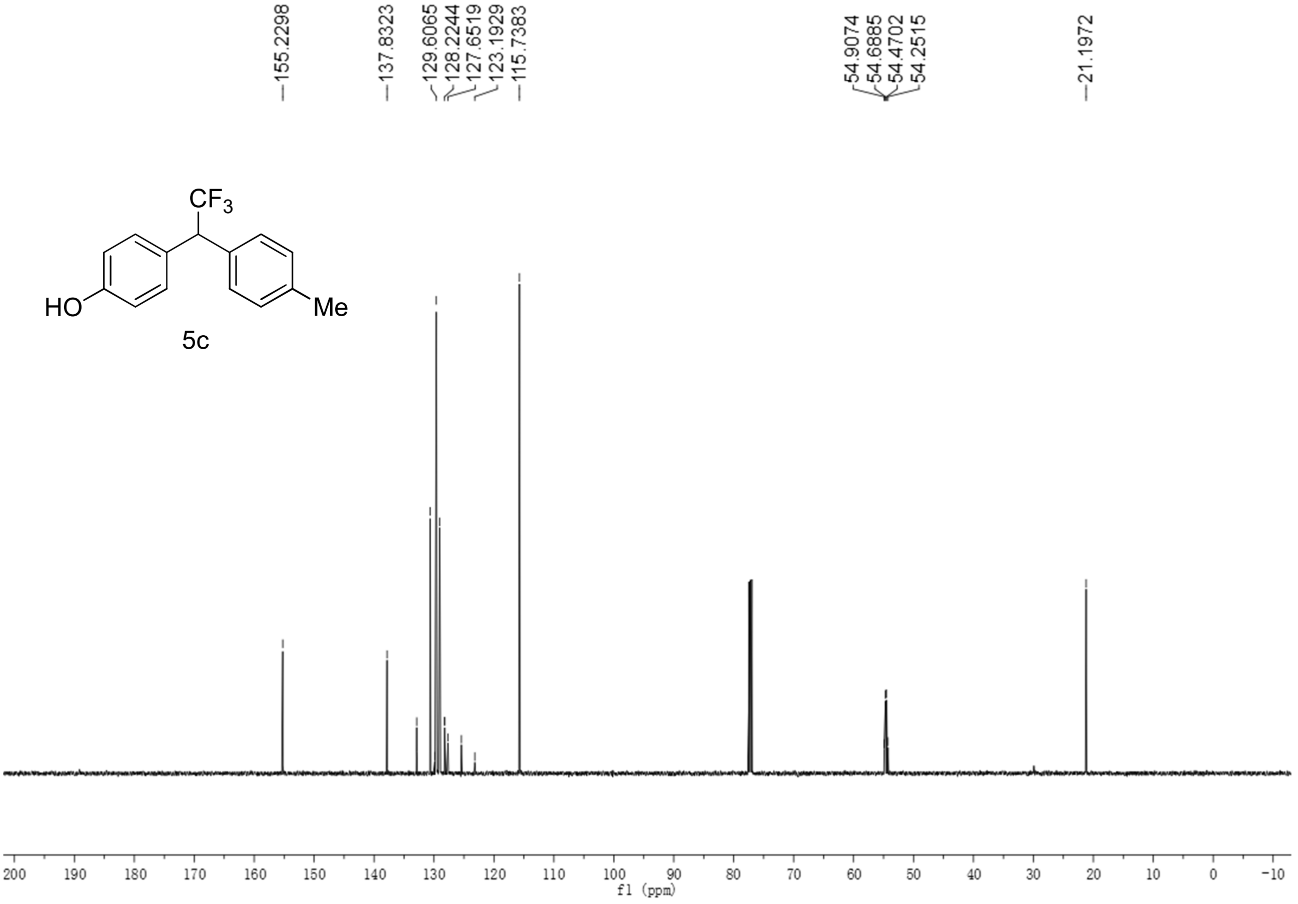
155.2298
137.8323
129.6065
128.2244
127.6519
123.1929
115.7383
54.9074
54.6885
54.4702
54.2515
21.1972
CF3
HO
Me
5c
200 190 180 170 160 150 140 130 120 110 100 90 80 70 60 50 40 30 20 10 0 -10
f1 (ppm)

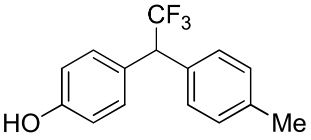

5c

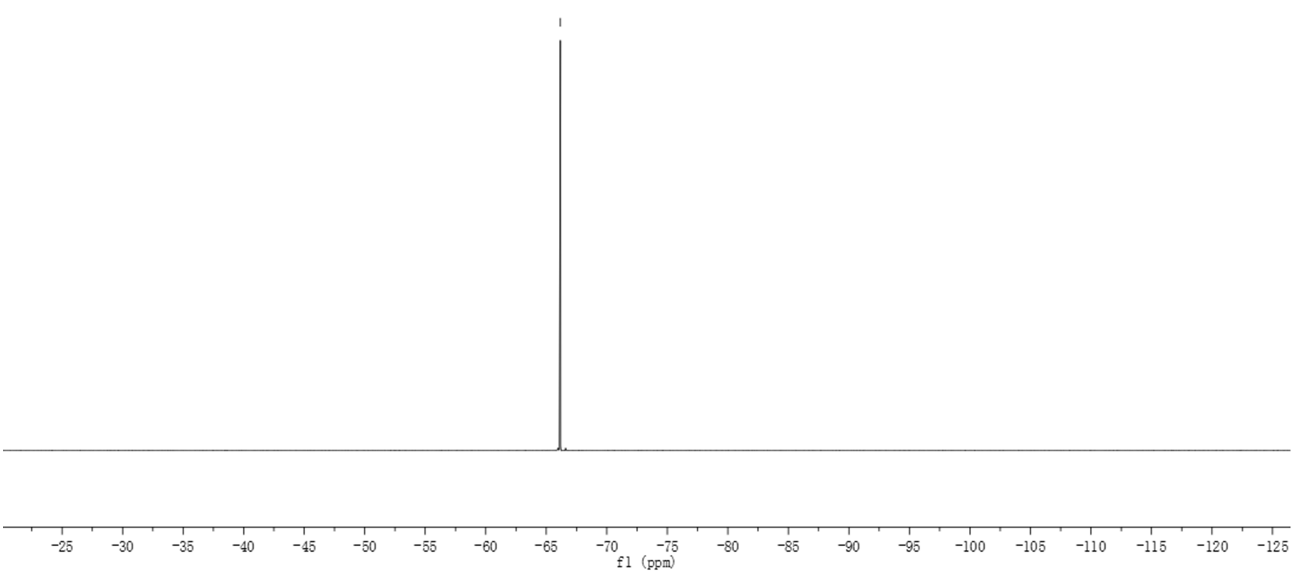

66.1679
f1 (ppm)

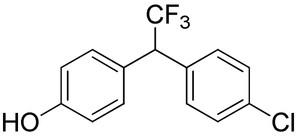

5d

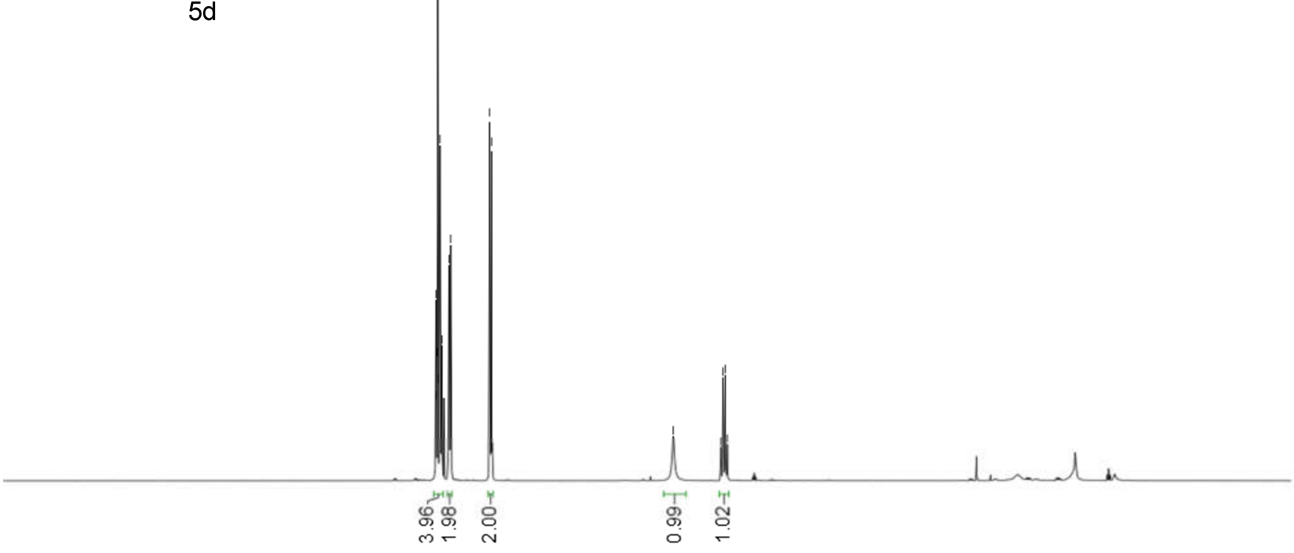

7.3355
7.3312
7.3226
7.3180
7.3139
7.2962
7.2790
7.2118
7.1946
6.8252
6.8078
5.0829
4.6301
4.6105
4.5909
4.5712
3.96
1.98
2.00
0.99
1.02
f1 (ppm)

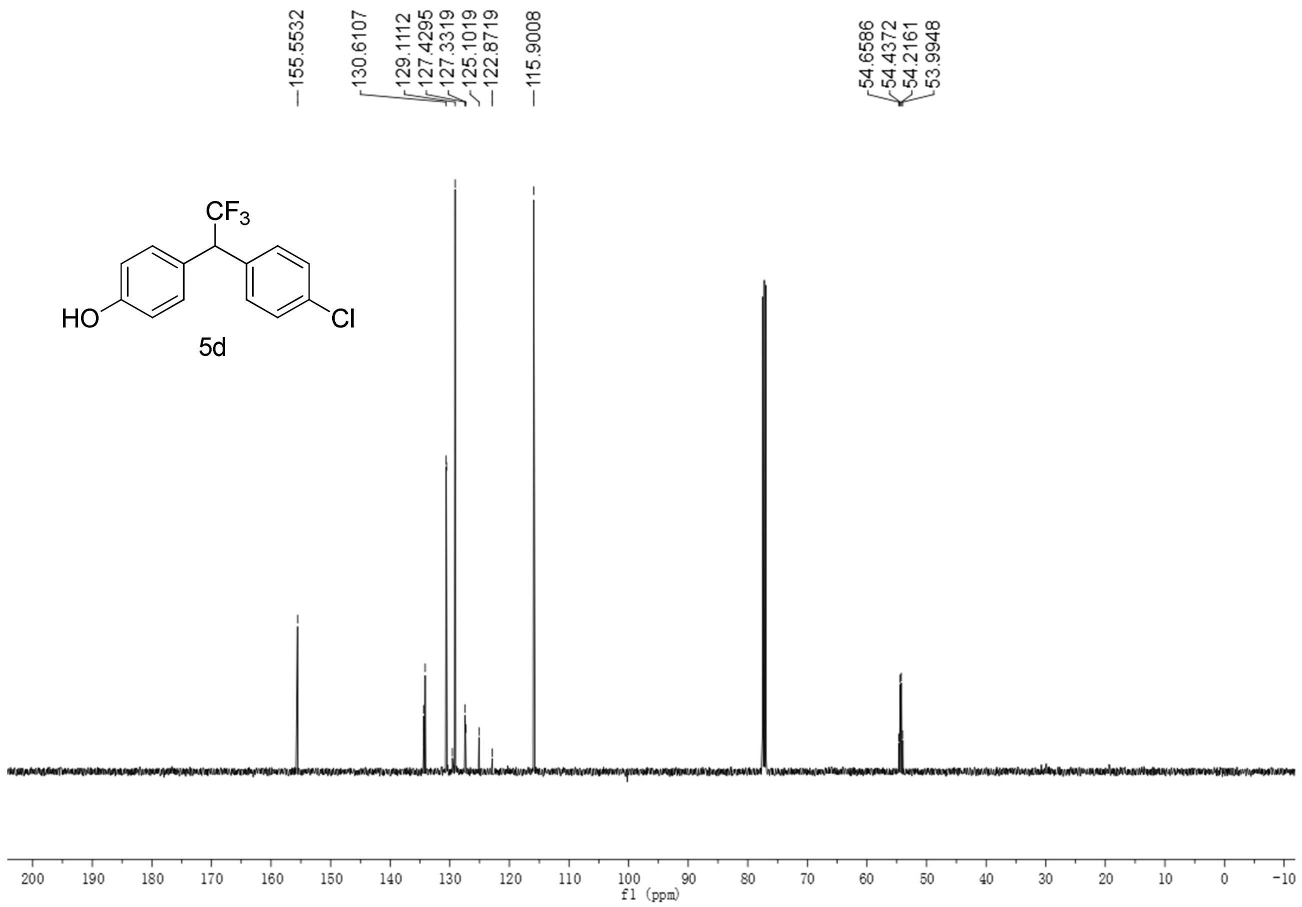
155.5532
130.6107
129.1112
127.4295
127.3319
125.1019
122.8719
115.9008
54.6586
54.4372
54.2161
53.9948
CF3
HO
Cl
5d
f1 (ppm)

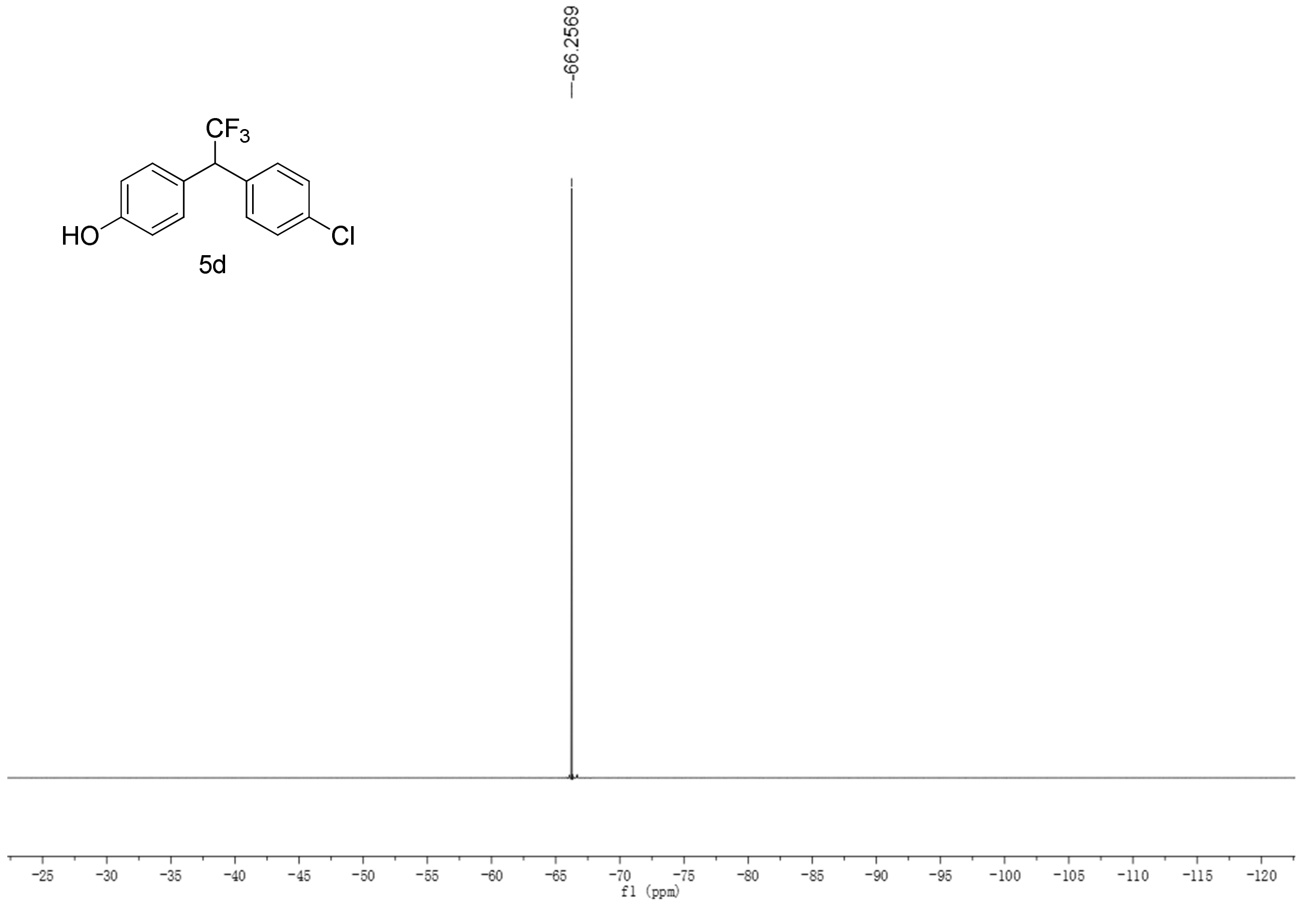
-66.2569
CF3
HO
Cl
5d
f1 (ppm)

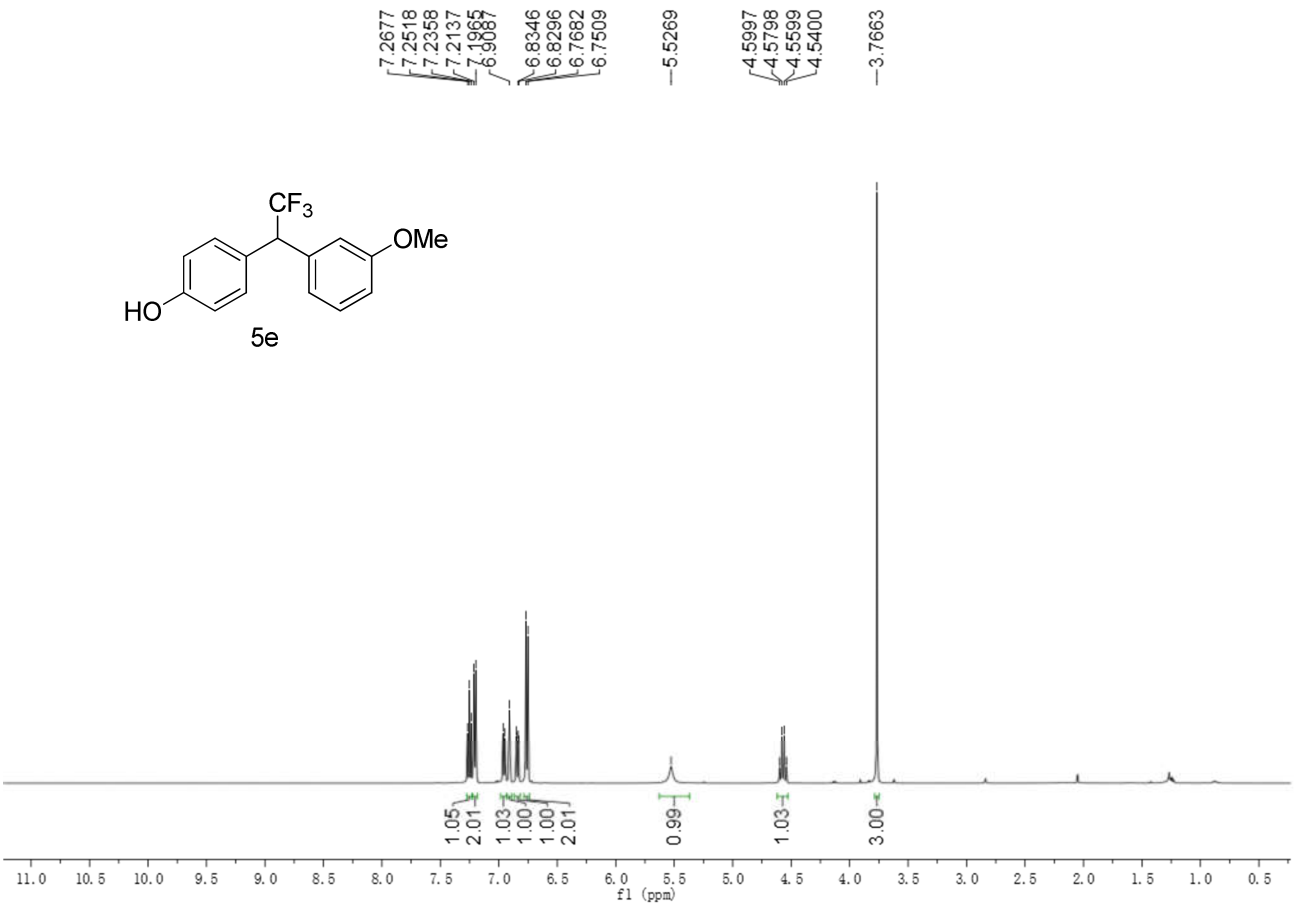
7.2677
7.2518
7.2358
7.2137
7.1965
6.9087
6.8346
6.8296
6.7682
6.7509
5.5269
4.5997
4.5798
4.5599
4.5400
3.7663
CF_3
OMe
HO
5e
1.05
2.01
1.03
1.00
1.00
2.01
0.99
1.03
3.00
f1 (ppm)

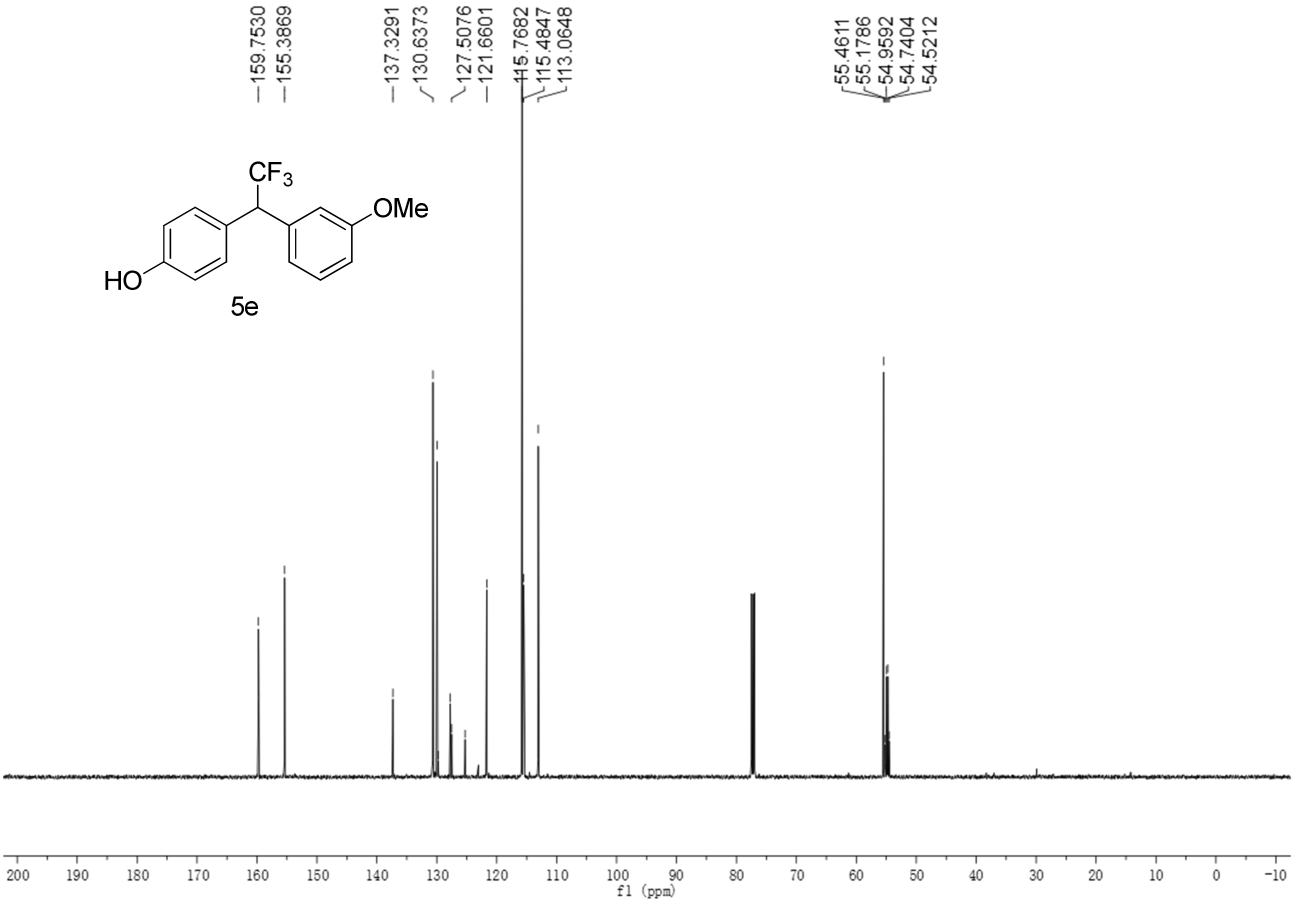
159.7530
155.3869
137.3291
130.6373
127.5076
121.6601
115.7682
115.4847
113.0648
55.4611
55.1786
54.9592
54.7404
54.5212
CF_3
OMe
HO
5e
f1 (ppm)

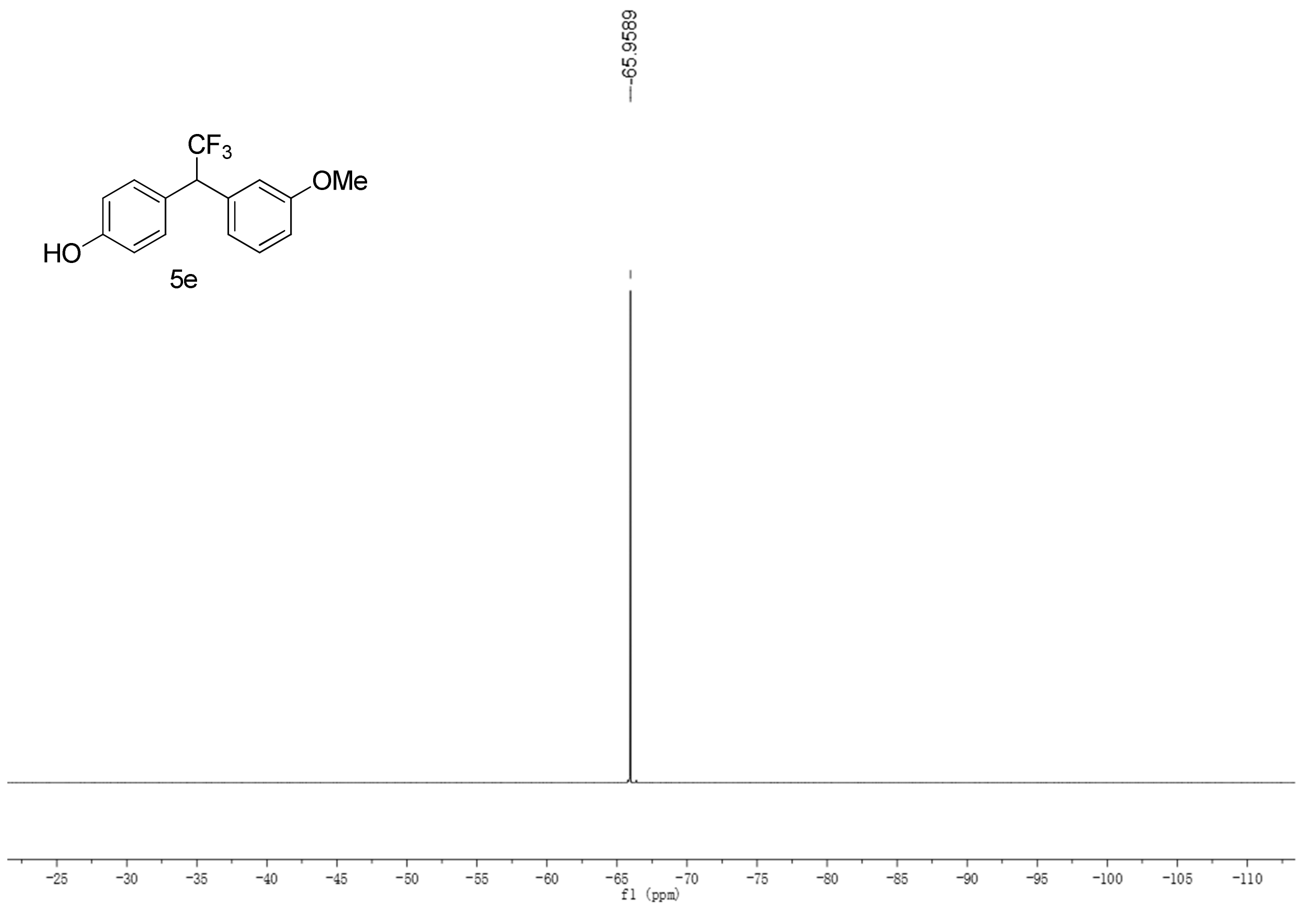

-65.9589
CF3
OMe
HO
5e
-25 -30 -35 -40 -45 -50 -55 -60 -65 -70 -75 -80 -85 -90 -95 -100 -105 -110
f1 (ppm)

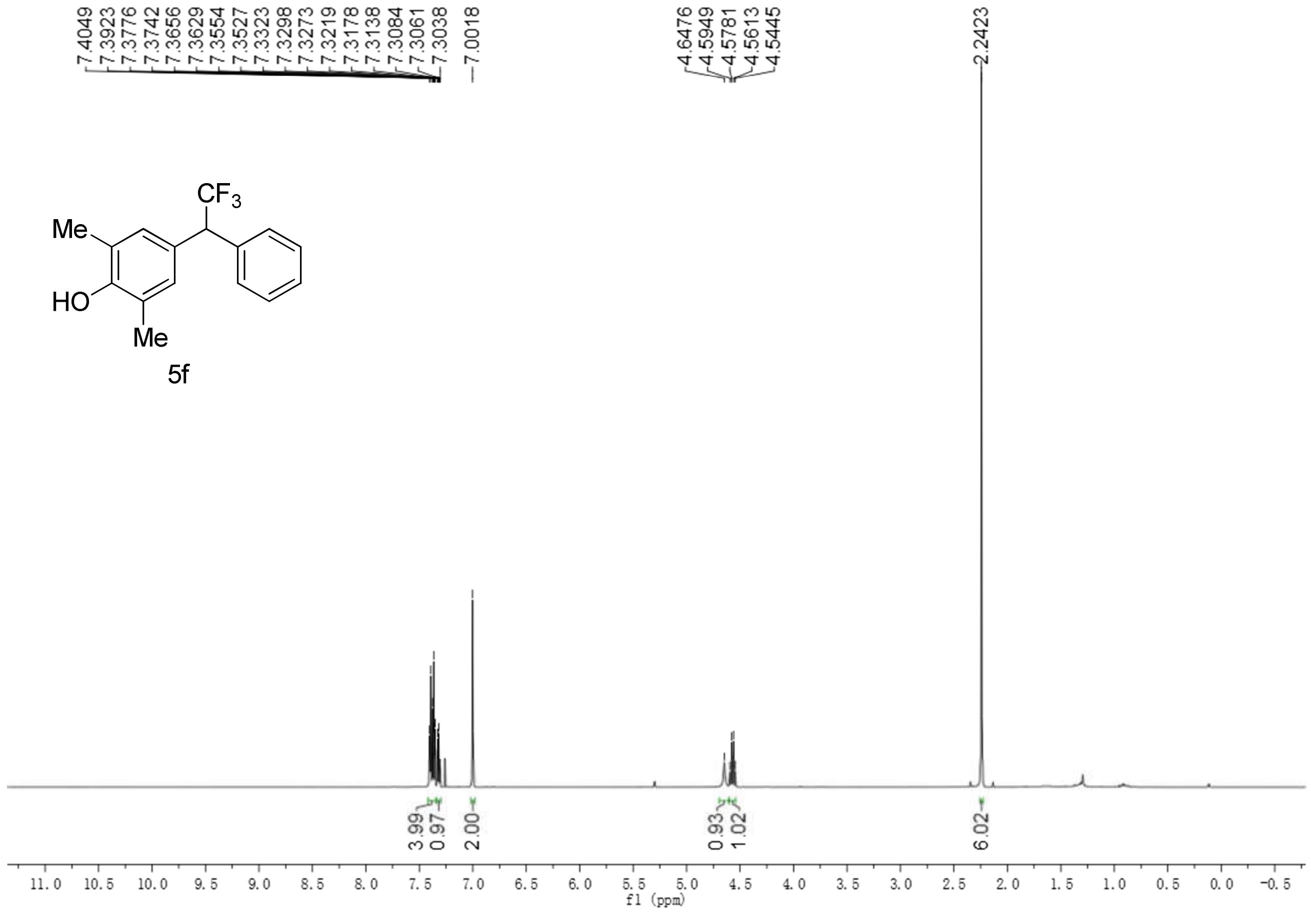

7.4049 7.3923 7.3776 7.3742 7.3656 7.3629 7.3554 7.3527 7.3323 7.3298 7.3273 7.3219 7.3178 7.3138 7.3084 7.3061 7.3038
7.0018
4.6476 4.5949 4.5781 4.5613 4.5445
2.2423
CF3
Me
HO
Me
5f
3.99 0.97 2.00 0.93 1.02 6.02
11.0 10.5 10.0 9.5 9.0 8.5 8.0 7.5 7.0 6.5 6.0 5.5 5.0 4.5 4.0 3.5 3.0 2.5 2.0 1.5 1.0 0.5 0.0 -0.5
f1 (ppm)

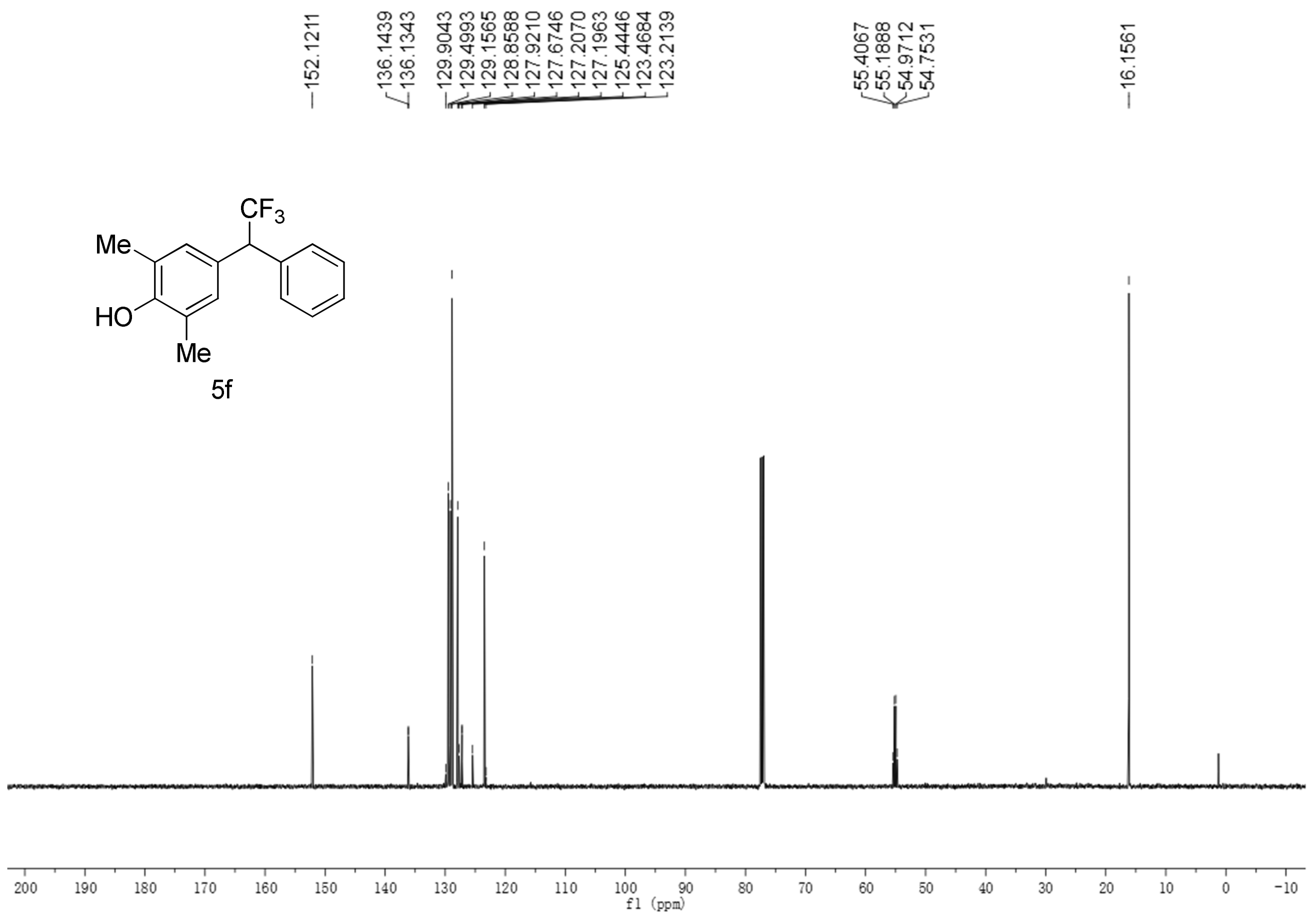
152.1211
136.1439
136.1343
129.9043
129.4993
129.1565
128.8588
127.9210
127.6746
127.2070
127.1963
125.4446
123.4684
123.2139
55.4067
55.1888
54.9712
54.7531
16.1561
CF3
Me
HO
Me
5f
f1 (ppm)

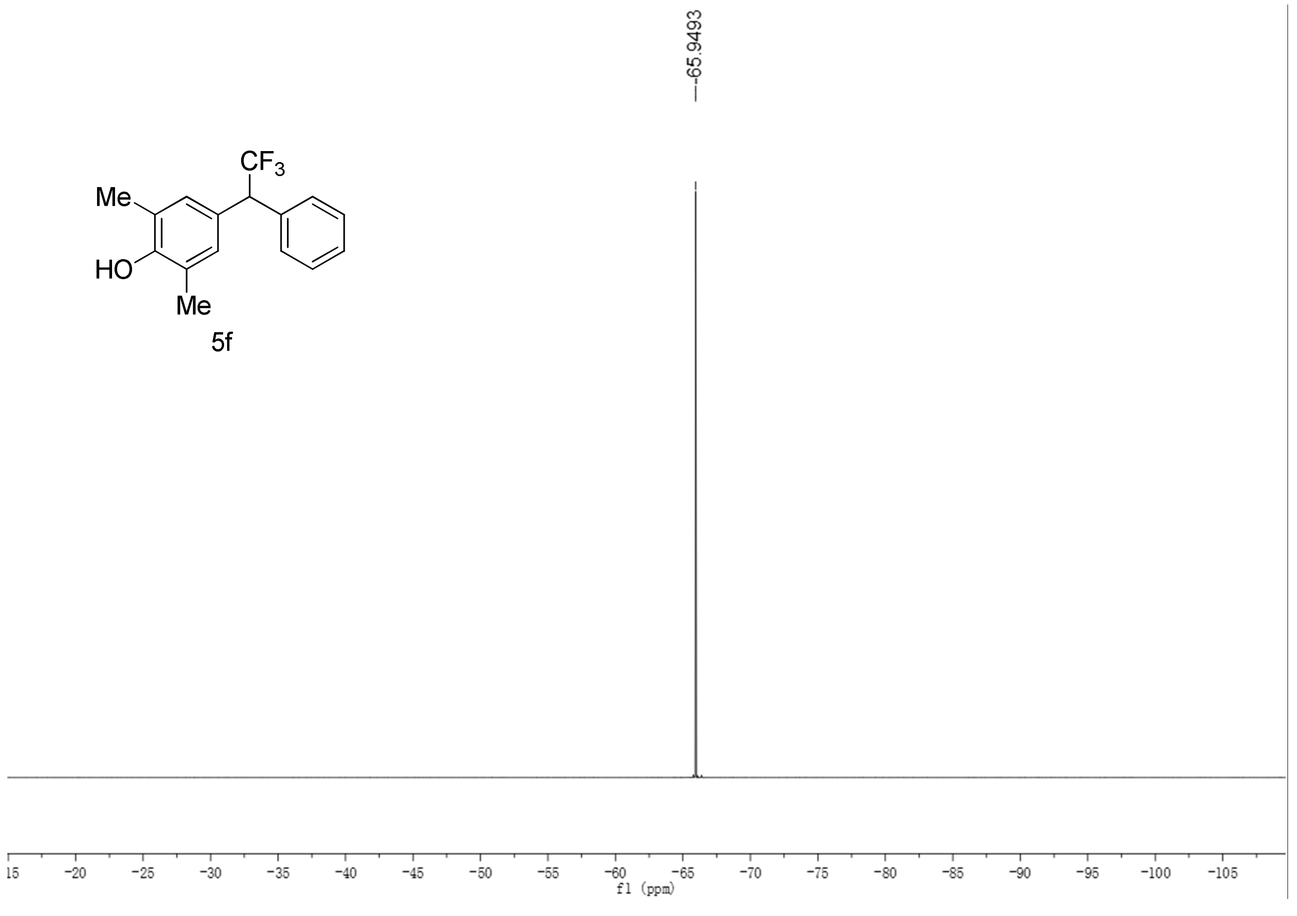
-65.9493
CF3
Me
HO
Me
5f
f1 (ppm)

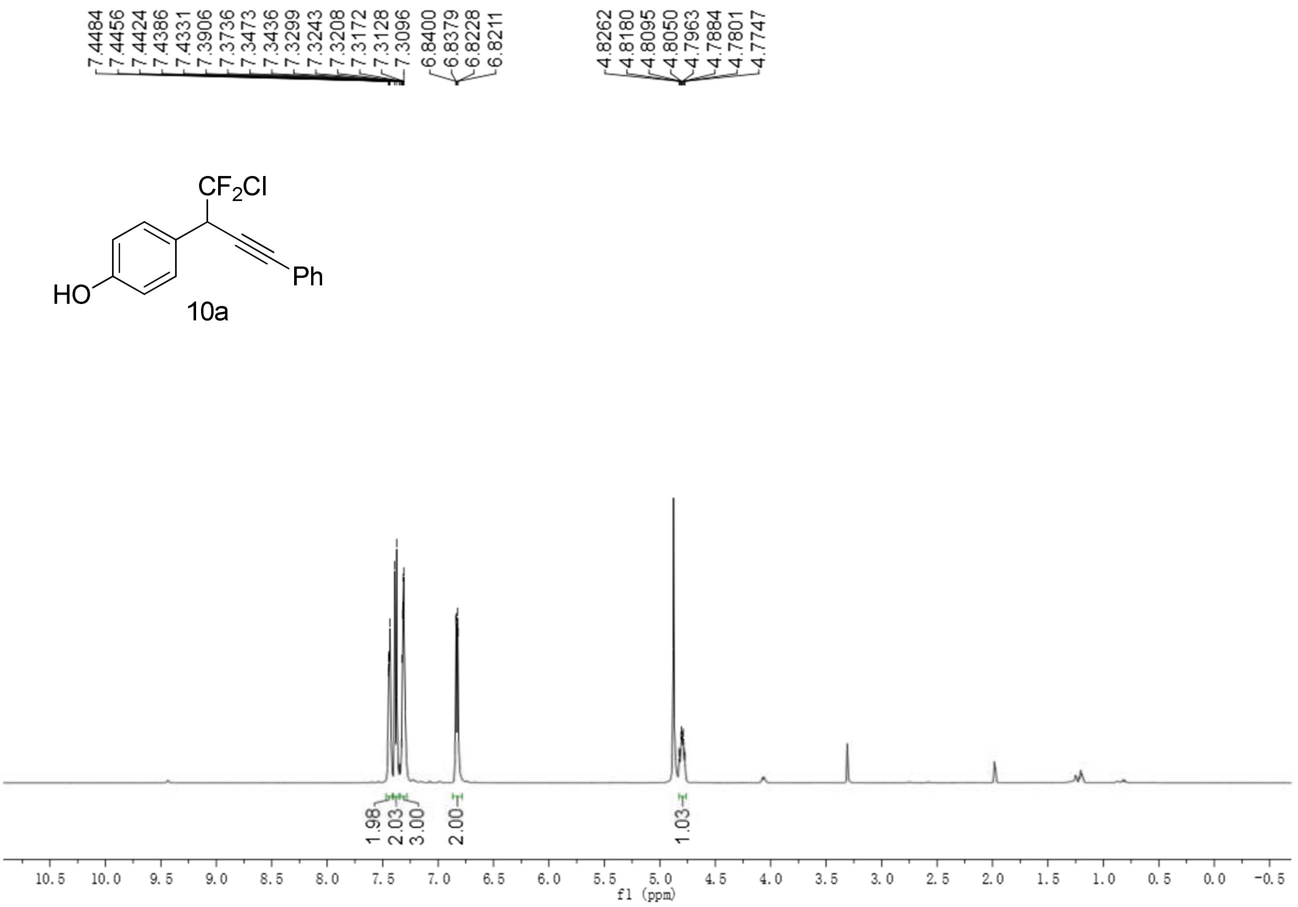
7.4484
7.4456
7.4424
7.4386
7.4331
7.3906
7.3736
7.3473
7.3436
7.3299
7.3243
7.3208
7.3172
7.3128
7.3096
6.8400
6.8379
6.8228
6.8211
4.8262
4.8180
4.8095
4.8050
4.7963
4.7884
4.7801
4.7747
CF2Cl
HO
Ph
10a
1.98
2.03
3.00
2.00
1.03
f1 (ppm)

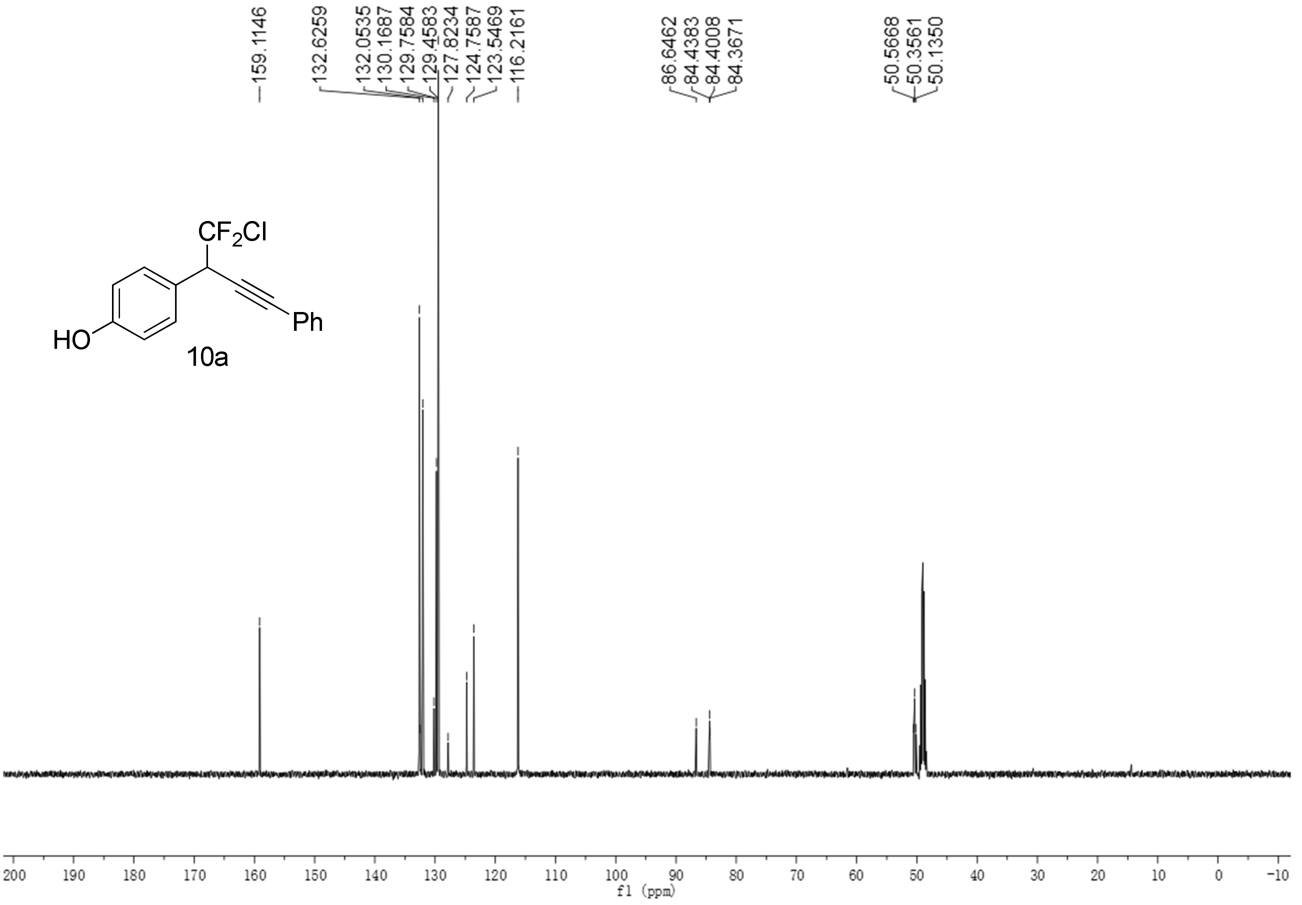
159.1146
132.6259
132.0535
130.1687
129.7584
129.4583
127.8234
124.7587
123.5469
116.2161
86.6462
84.4383
84.4008
84.3671
50.5668
50.3561
50.1350
CF2Cl
HO
Ph
10a
f1 (ppm)

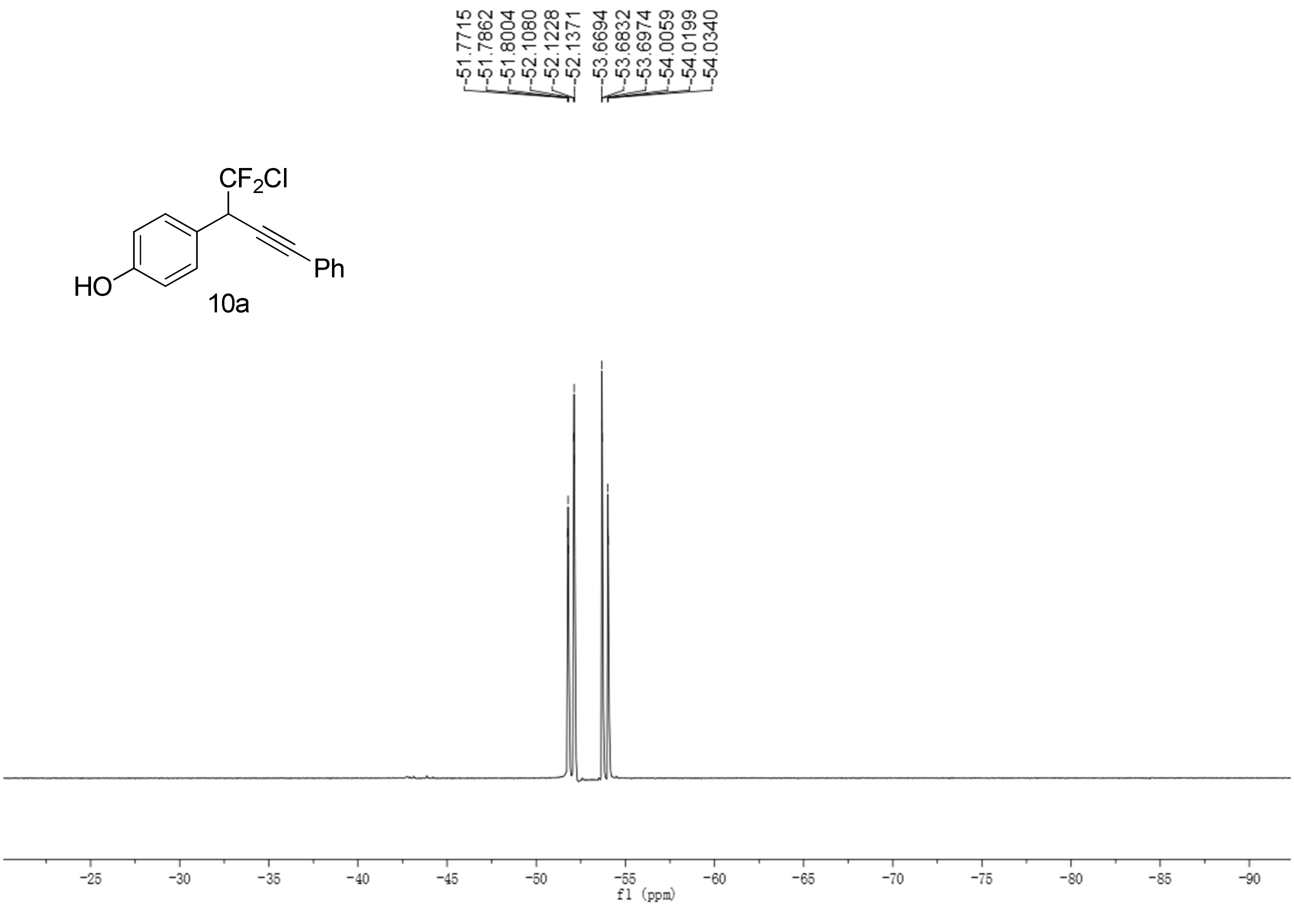

-51.7715
-51.7862
-51.8004
-52.1080
-52.1228
-52.1371
-53.6694
-53.6832
-53.6974
-54.0059
-54.0199
-54.0340
CF2Cl
HO
Ph
10a
f1 (ppm)

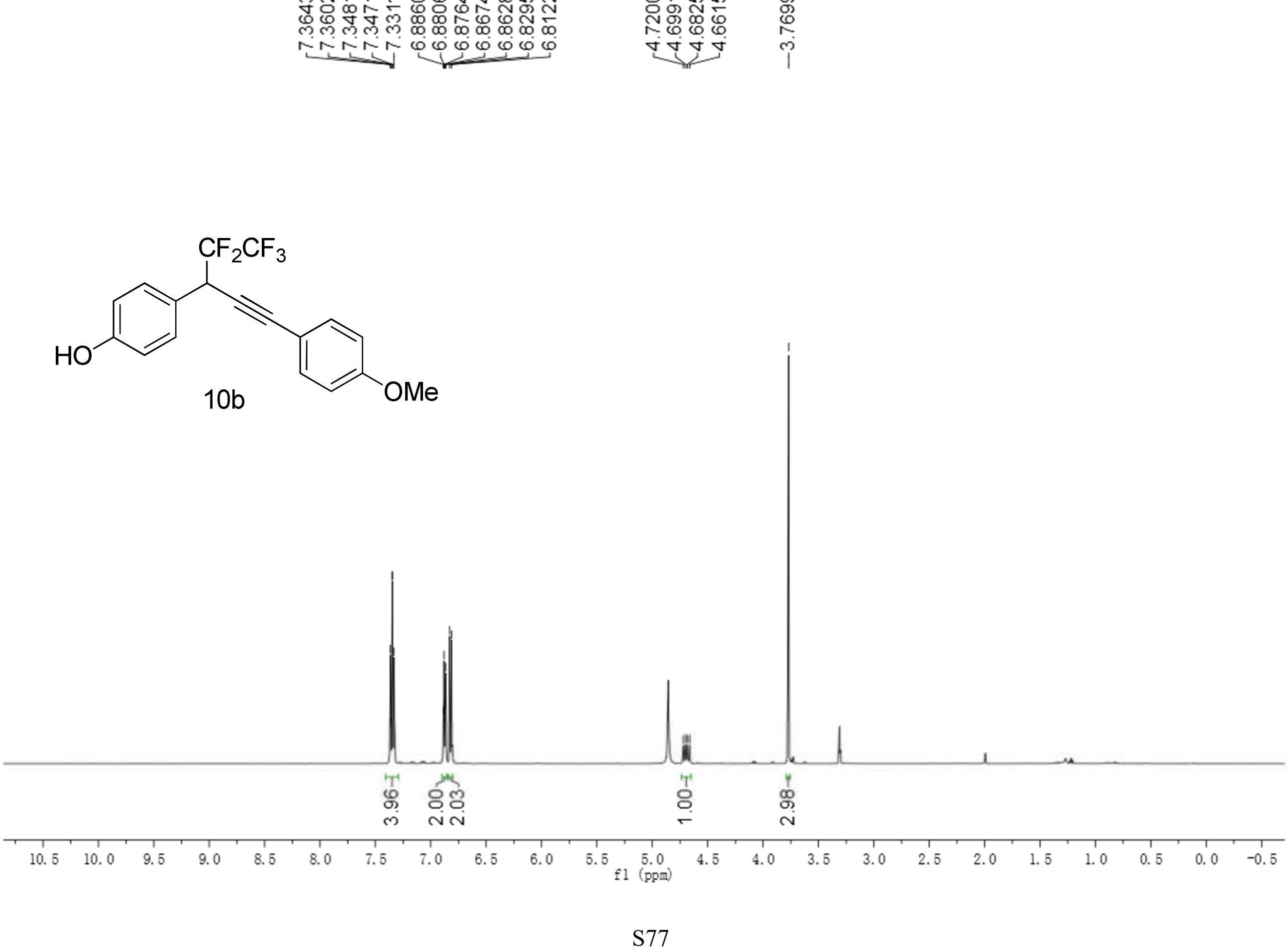

7.3643
7.3602
7.3481
7.3471
7.3311
6.8860
6.8806
6.8764
6.8674
6.8628
6.8295
6.8122
4.7200
4.6991
4.6825
4.6615
3.7699
CF2CF3
HO
OMe
10b
3.96
2.00
2.03
1.00
2.98
f1 (ppm)

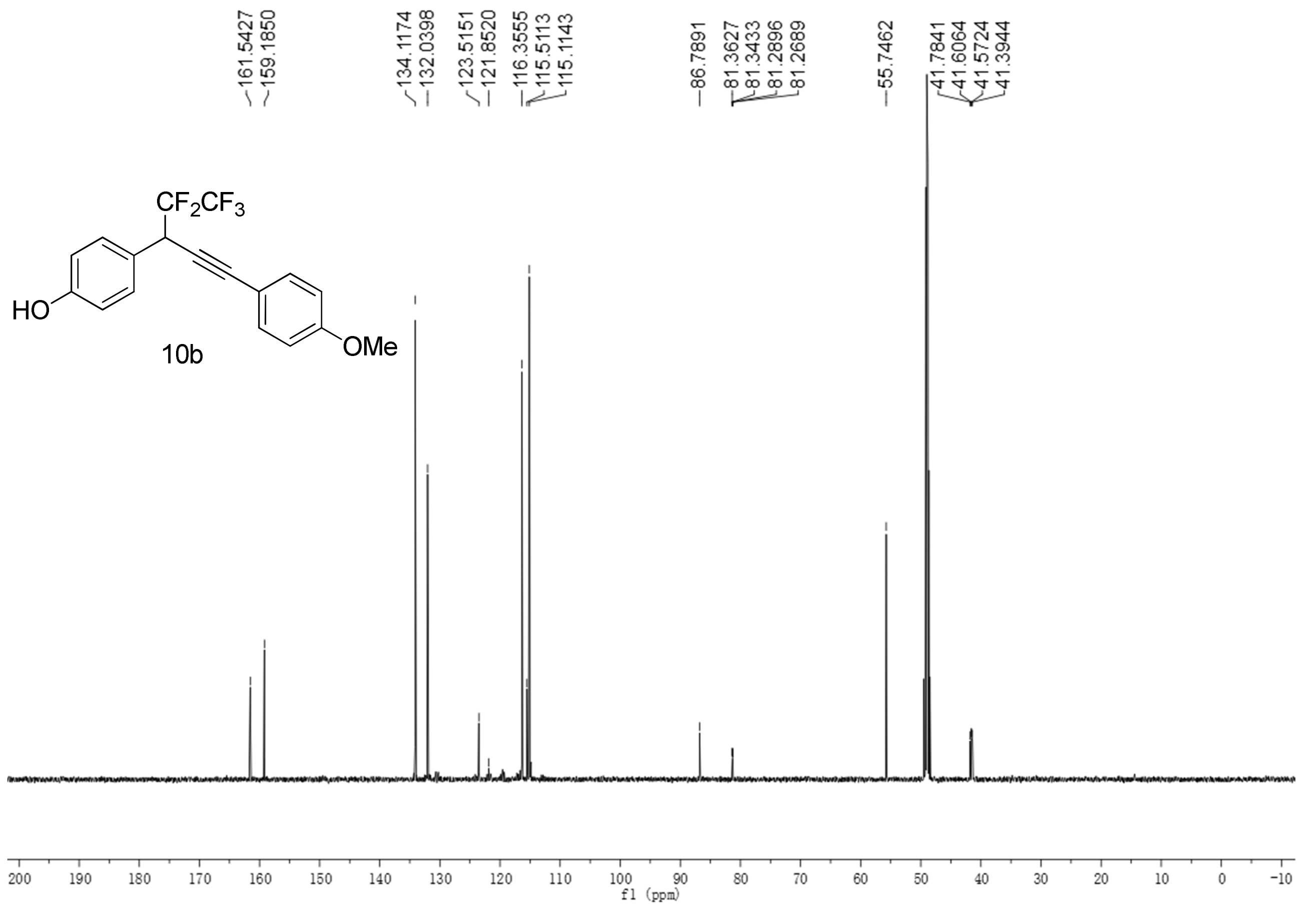
161.5427
159.1850
134.1174
132.0398
123.5151
121.8520
116.3555
115.5113
115.1143
86.7891
81.3627
81.3433
81.2896
81.2689
55.7462
41.7841
41.6064
41.5724
41.3944
CF2CF3
HO
OMe
10b
f1 (ppm)

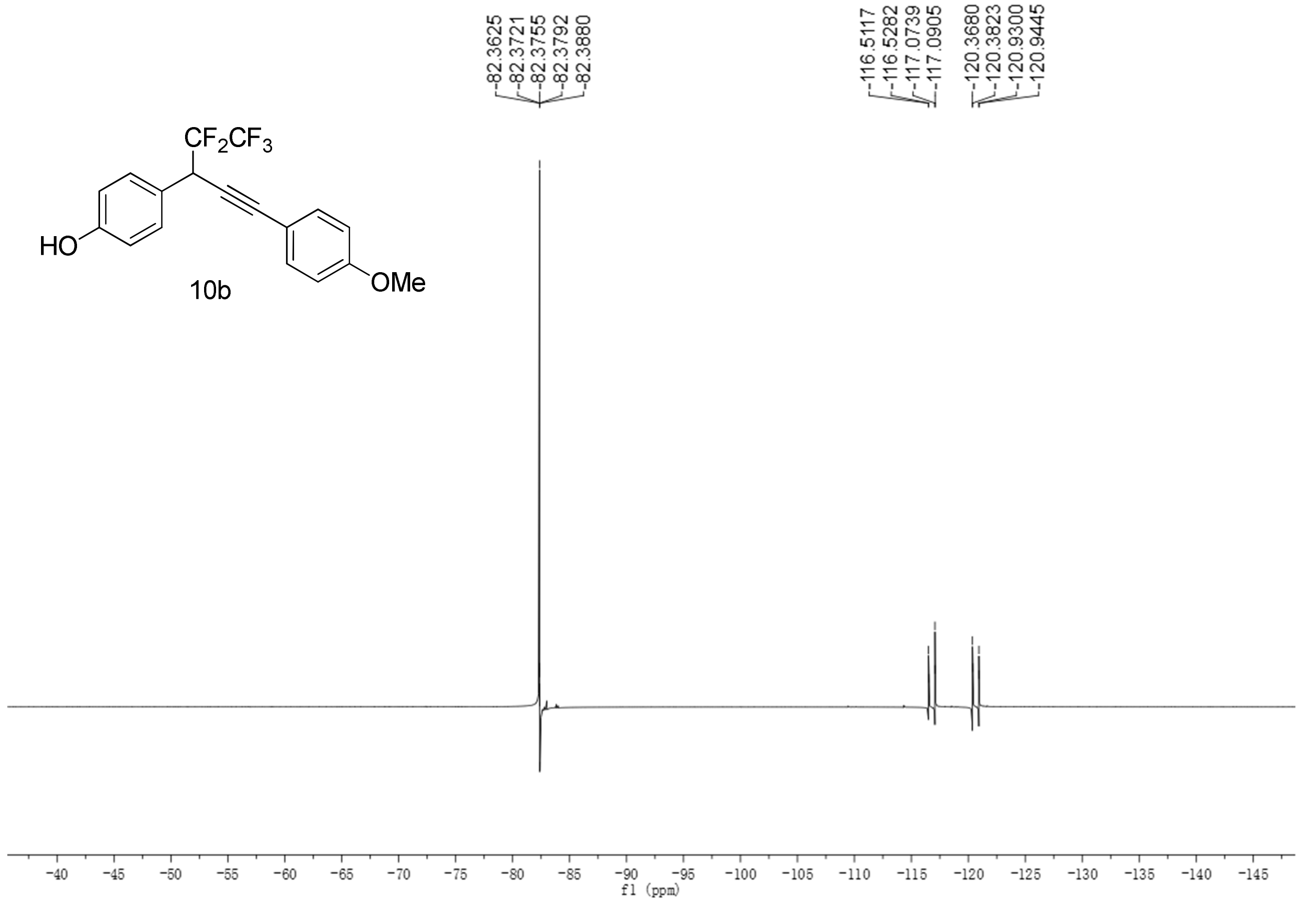
-82.3625
-82.3721
-82.3755
-82.3792
-82.3880
-116.5117
-116.5282
-117.0739
-117.0905
-120.3680
-120.3823
-120.9300
-120.9445
CF2CF3
HO
OMe
10b
f1 (ppm)

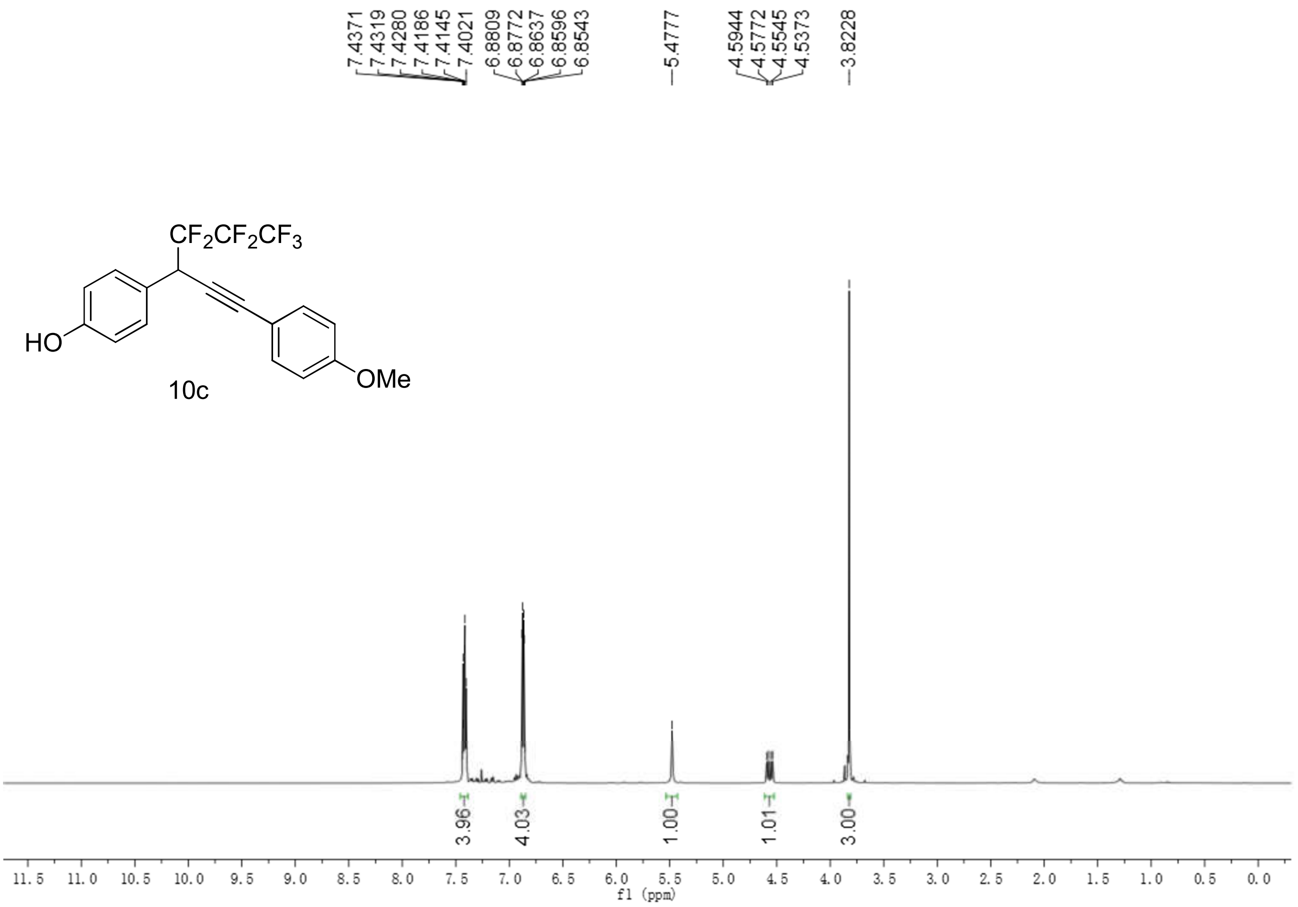
7.4371
7.4319
7.4280
7.4186
7.4145
7.4021
6.8809
6.8772
6.8637
6.8596
6.8543
5.4777
4.5944
4.5772
4.5545
4.5373
3.8228
$CF_2CF_2CF_3$
HO
OMe
10c
3.96
4.03
1.00
1.01
3.00
f1 (ppm)

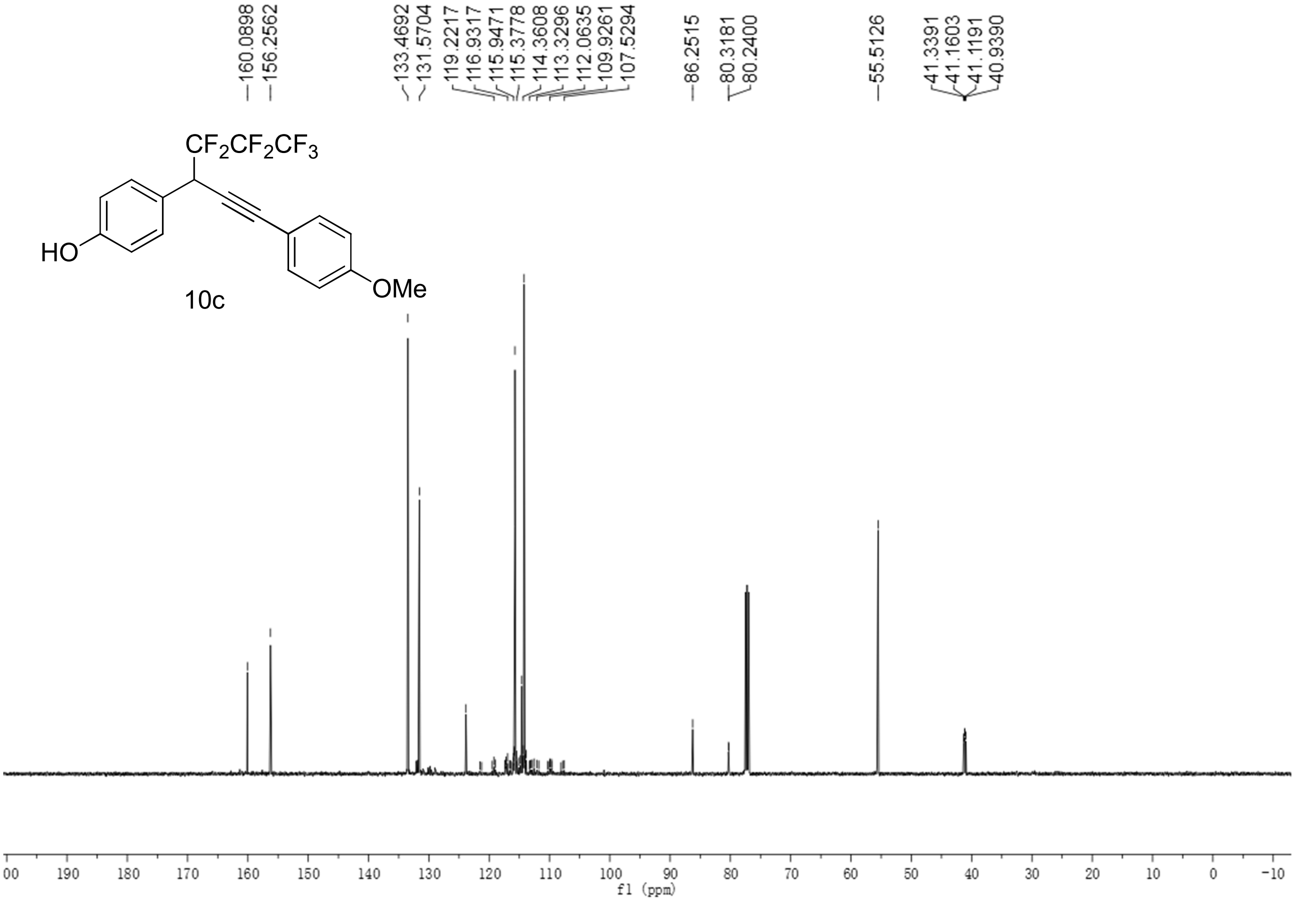
160.0898
156.2562
133.4692
131.5704
119.2217
116.9317
115.9471
115.3778
114.3608
113.3296
112.0635
109.9261
107.5294
86.2515
80.3181
80.2400
55.5126
41.3391
41.1603
41.1191
40.9390
$CF_2CF_2CF_3$
HO
OMe
10c
f1 (ppm)

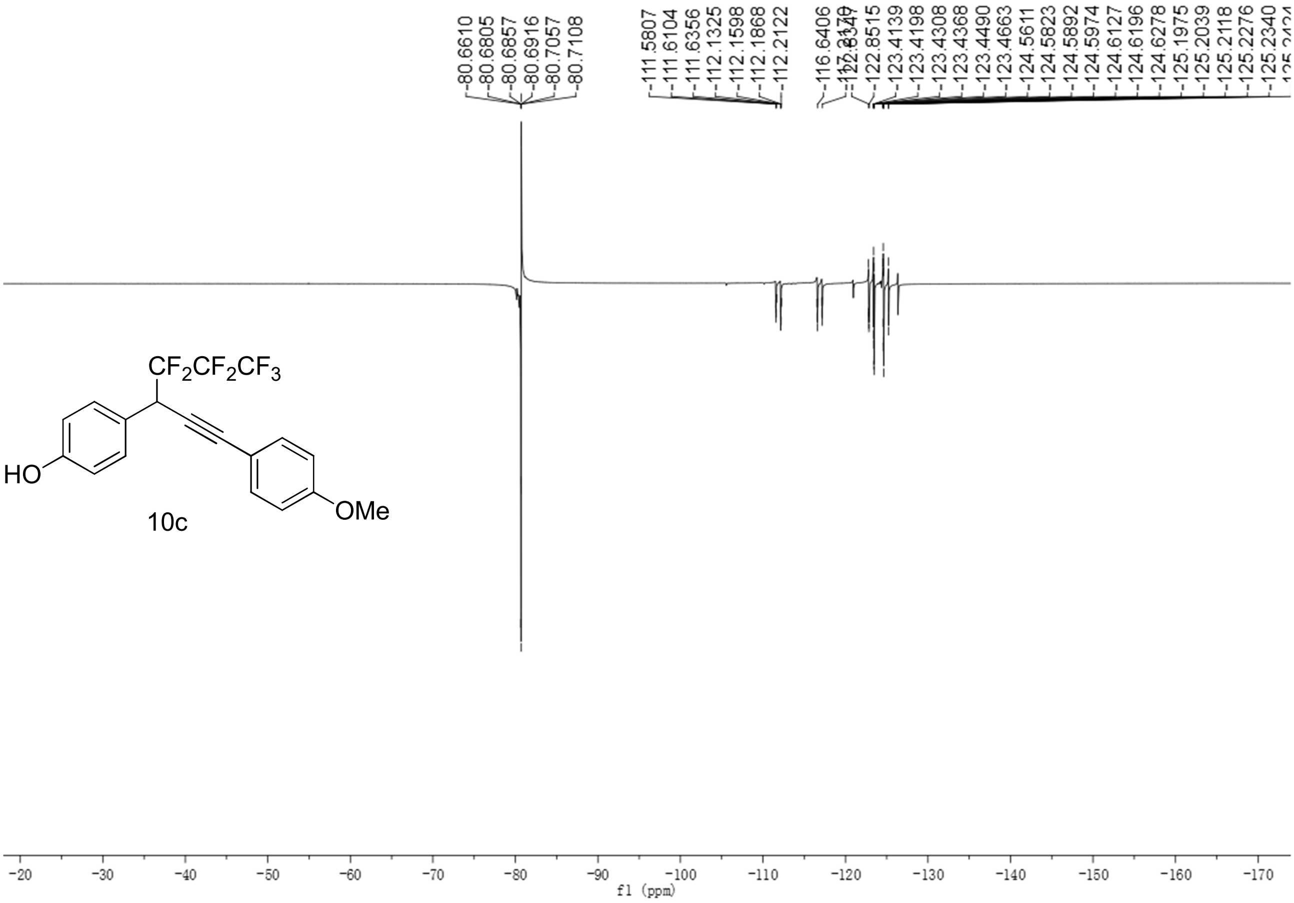

-80.6610
-80.6805
-80.6857
-80.6916
-80.7057
-80.7108
-111.5807
-111.6104
-111.6356
-112.1325
-112.1598
-112.1868
-112.2122
-116.6406
-122.8515
-123.4139
-123.4198
-123.4308
-123.4368
-123.4490
-123.4663
-124.5611
-124.5823
-124.5892
-124.5974
-124.6127
-124.6196
-124.6278
-125.1975
-125.2039
-125.2118
-125.2276
-125.2340
CF2CF2CF3
HO
OMe
10c
-20 -30 -40 -50 -60 -70 -80 -90 -100 -110 -120 -130 -140 -150 -160 -170
f1 (ppm)

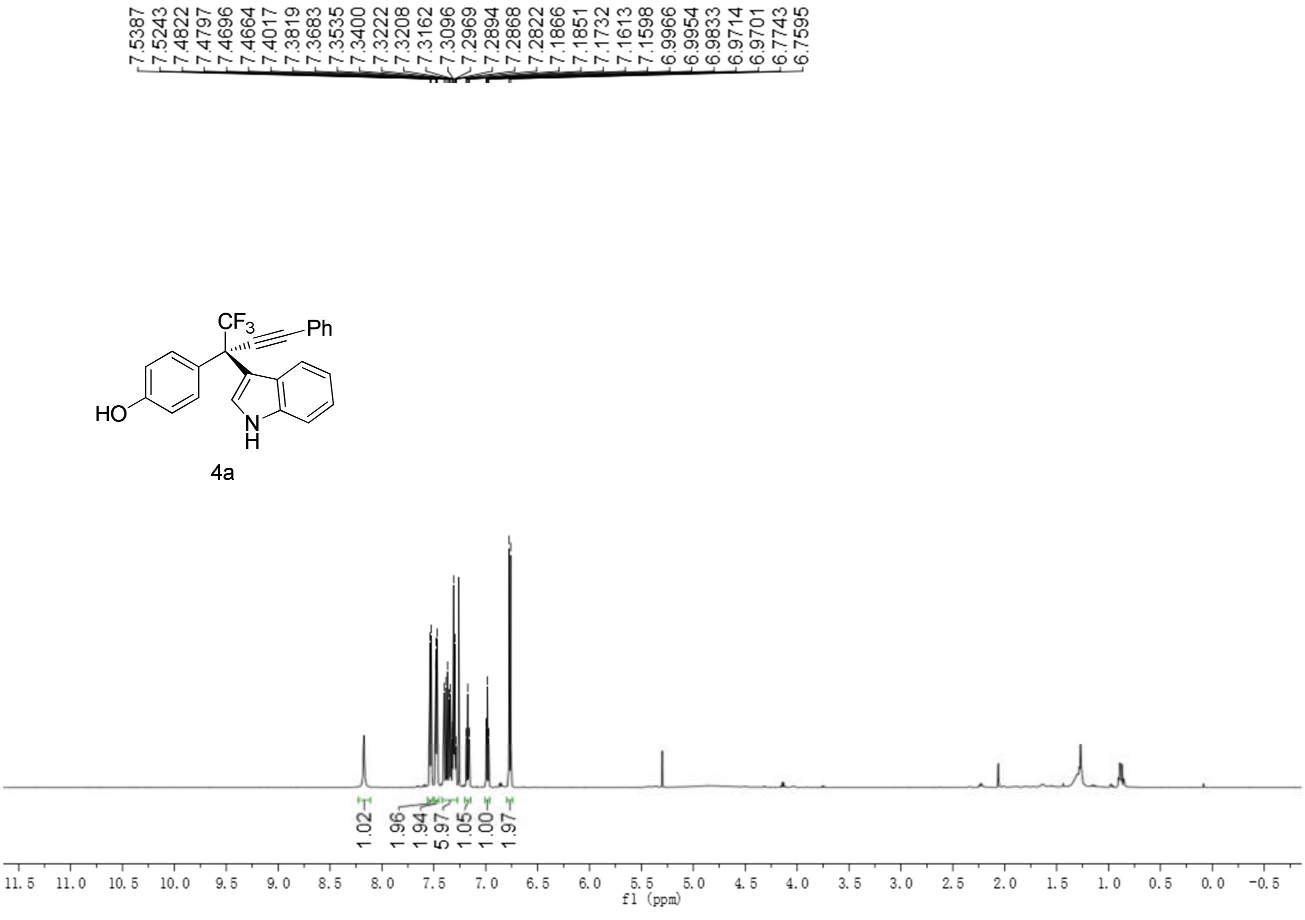

7.5387
7.5243
7.4822
7.4797
7.4696
7.4664
7.4017
7.3819
7.3683
7.3535
7.3400
7.3222
7.3208
7.3162
7.3096
7.2969
7.2894
7.2868
7.2822
7.1866
7.1851
7.1732
7.1613
7.1598
6.9966
6.9954
6.9833
6.9714
6.9701
6.7743
6.7595
CF3
Ph
HO
N
H
4a
1.02
1.96
1.94
5.97
1.05
1.00
1.97
11.5 11.0 10.5 10.0 9.5 9.0 8.5 8.0 7.5 7.0 6.5 6.0 5.5 5.0 4.5 4.0 3.5 3.0 2.5 2.0 1.5 1.0 0.5 0.0 -0.5
f1 (ppm)

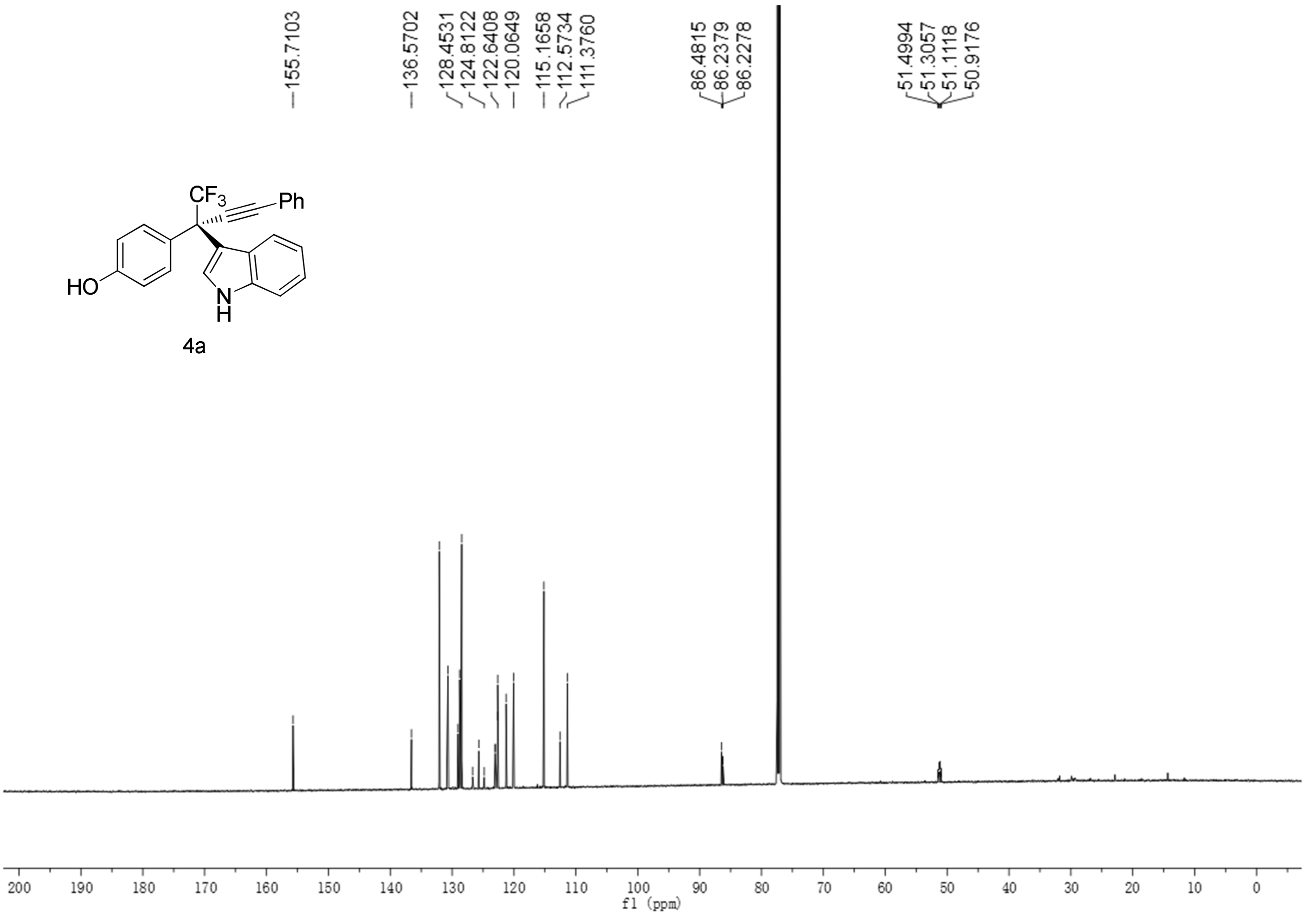
155.7103
136.5702
128.4531
124.8122
122.6408
120.0649
115.1658
112.5734
111.3760
86.4815
86.2379
86.2278
51.4994
51.3057
51.1118
50.9176
CF3
Ph
HO
N
H
4a
200 190 180 170 160 150 140 130 120 110 100 90 80 70 60 50 40 30 20 10 0
f1 (ppm)

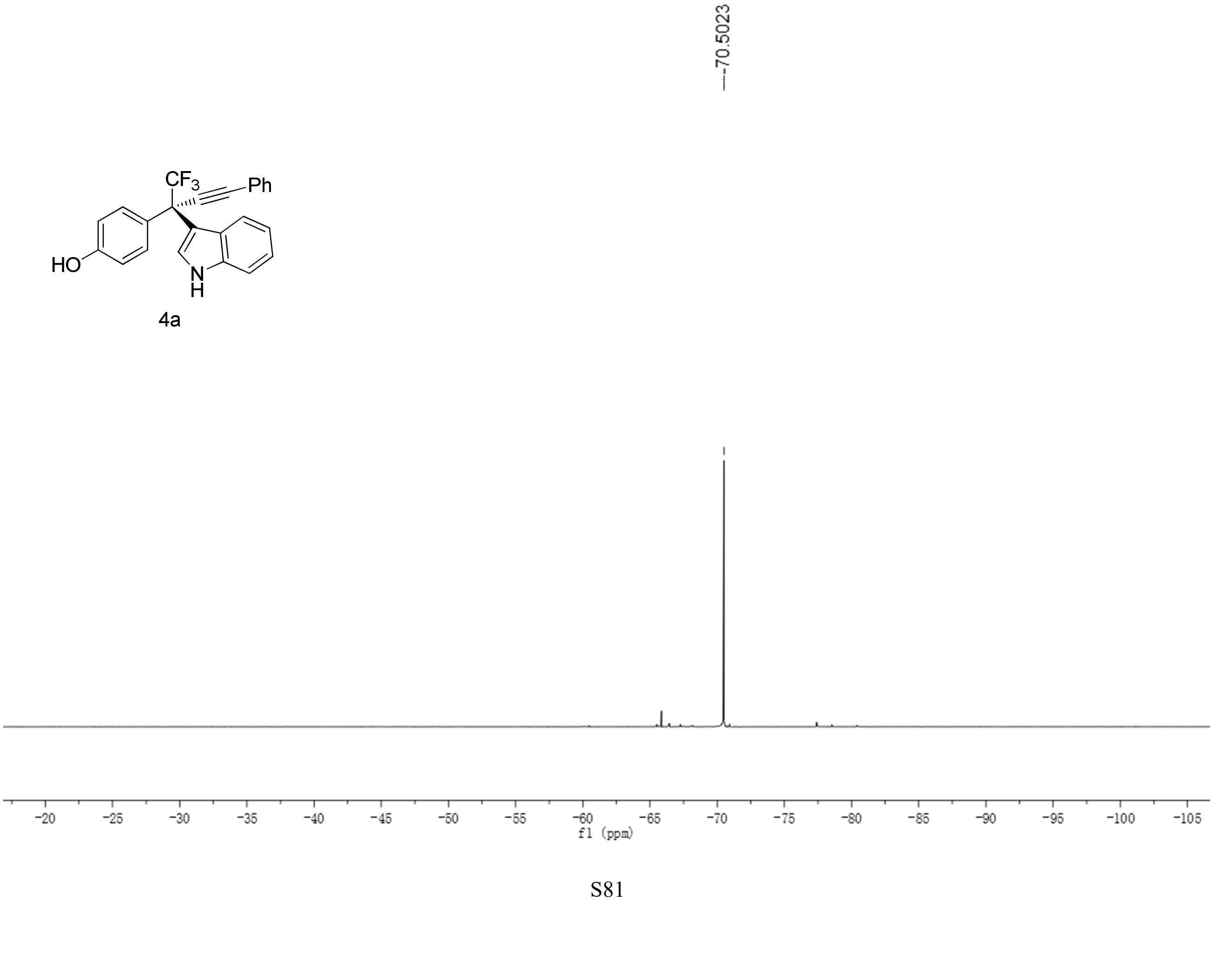
-70.5023
CF3
Ph
HO
N
H
4a
-20 -25 -30 -35 -40 -45 -50 -55 -60 -65 -70 -75 -80 -85 -90 -95 -100 -105
f1 (ppm)

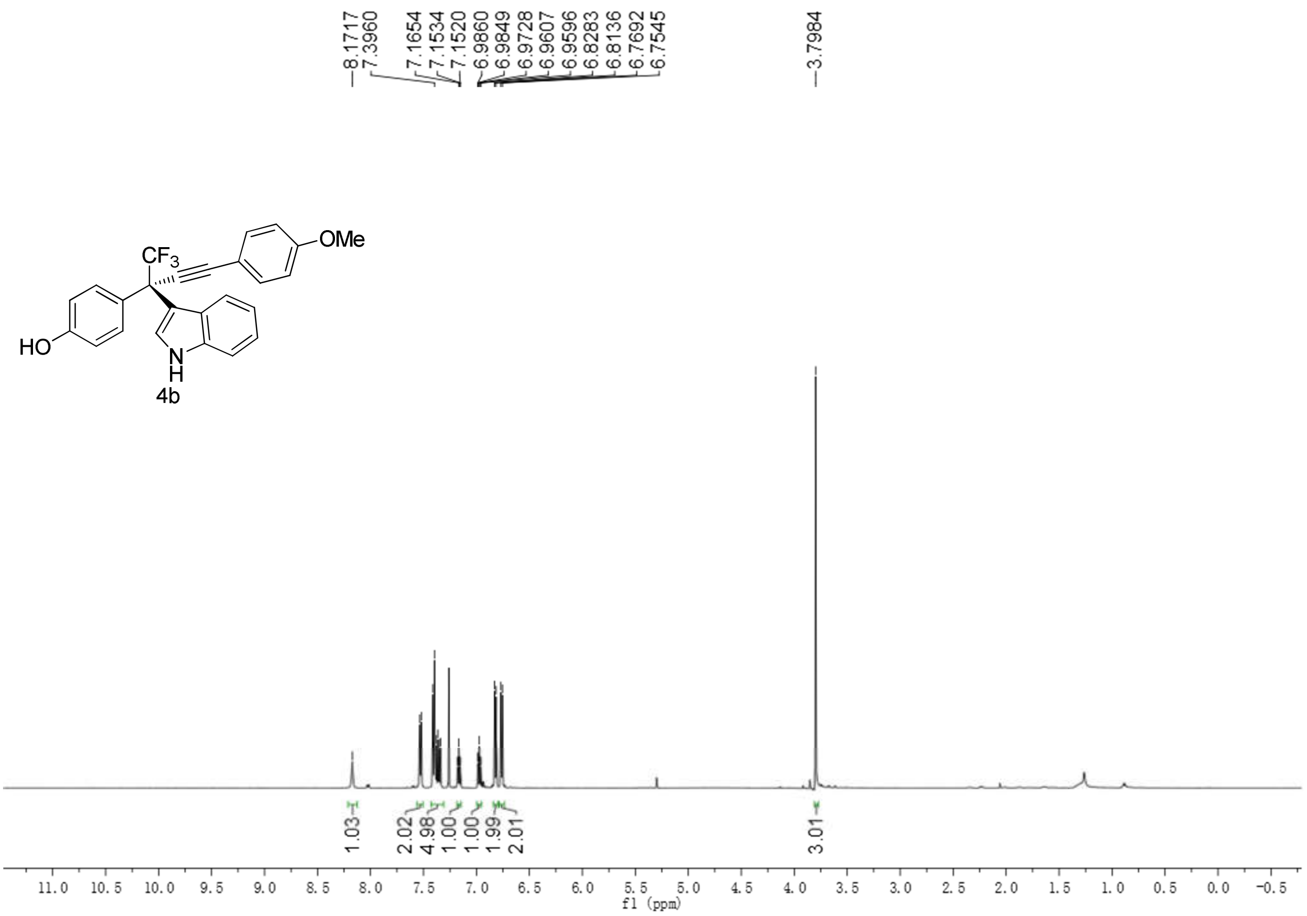
8.1717
7.3960
7.1654
7.1534
7.1520
6.9860
6.9849
6.9728
6.9607
6.9596
6.8283
6.8136
6.7692
6.7545
3.7984
CF3
OMe
HO
N
H
4b
1.03
2.02
4.98
1.00
1.00
1.99
2.01
3.01
f1 (ppm)

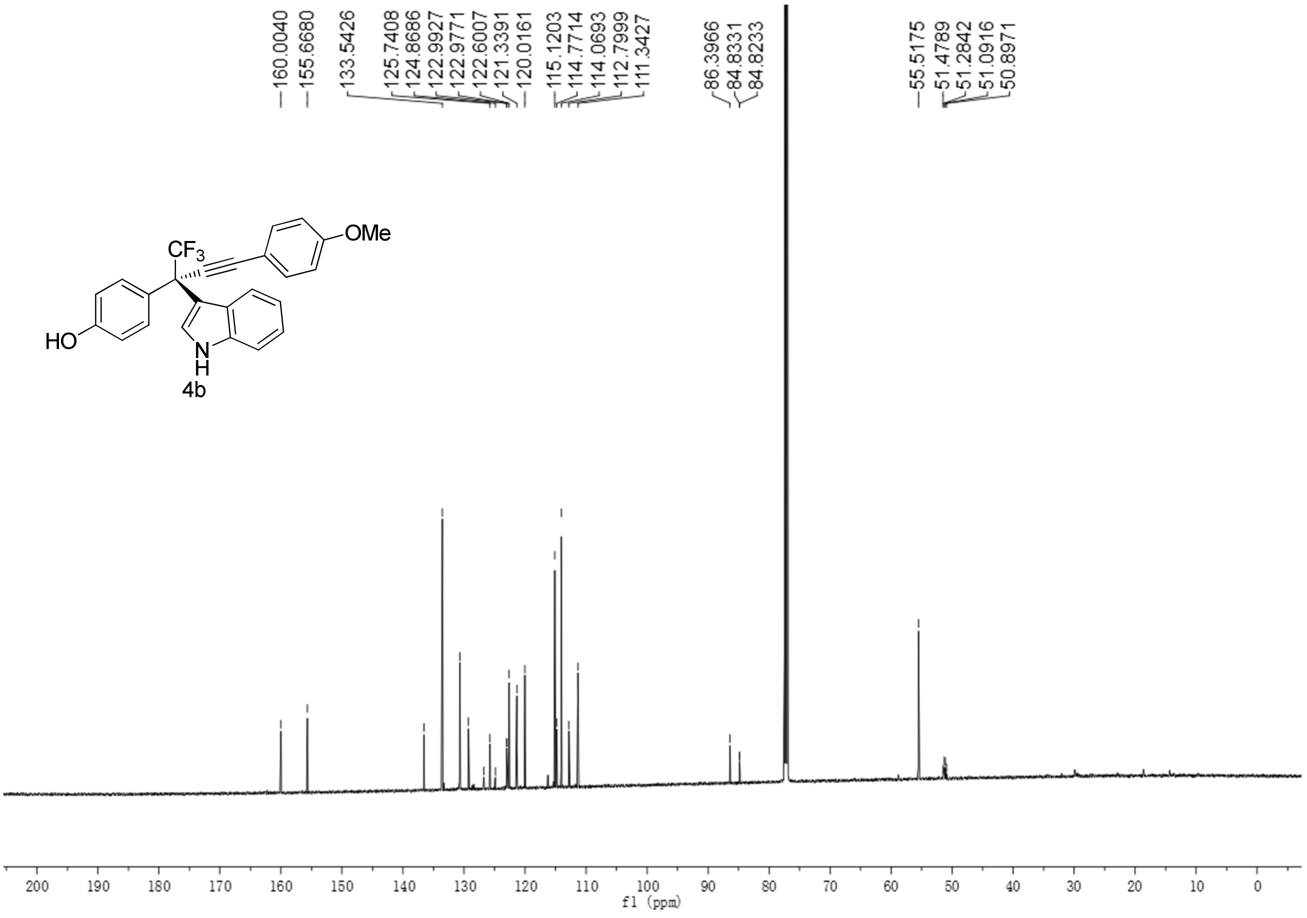
160.0040
155.6680
133.5426
125.7408
124.8686
122.9927
122.9771
122.6007
121.3391
120.0161
115.1203
114.7714
114.0693
112.7999
111.3427
86.3966
84.8331
84.8233
55.5175
51.4789
51.2842
51.0916
50.8971
CF3
OMe
HO
N
H
4b
f1 (ppm)

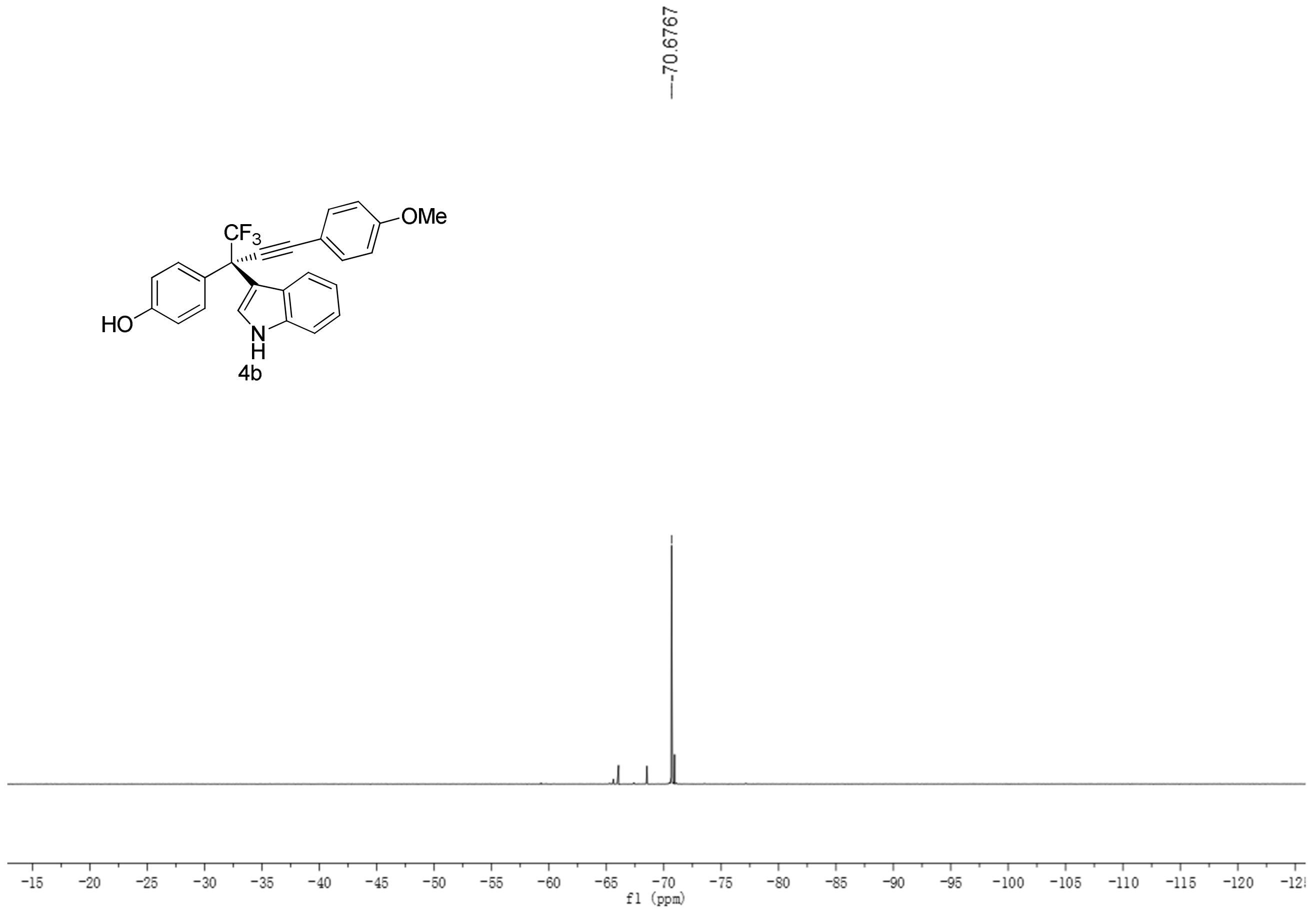

-70.6767
CF3
OMe
HO
N
H
4b
-15 -20 -25 -30 -35 -40 -45 -50 -55 -60 -65 -70 -75 -80 -85 -90 -95 -100 -105 -110 -115 -120
f1 (ppm)

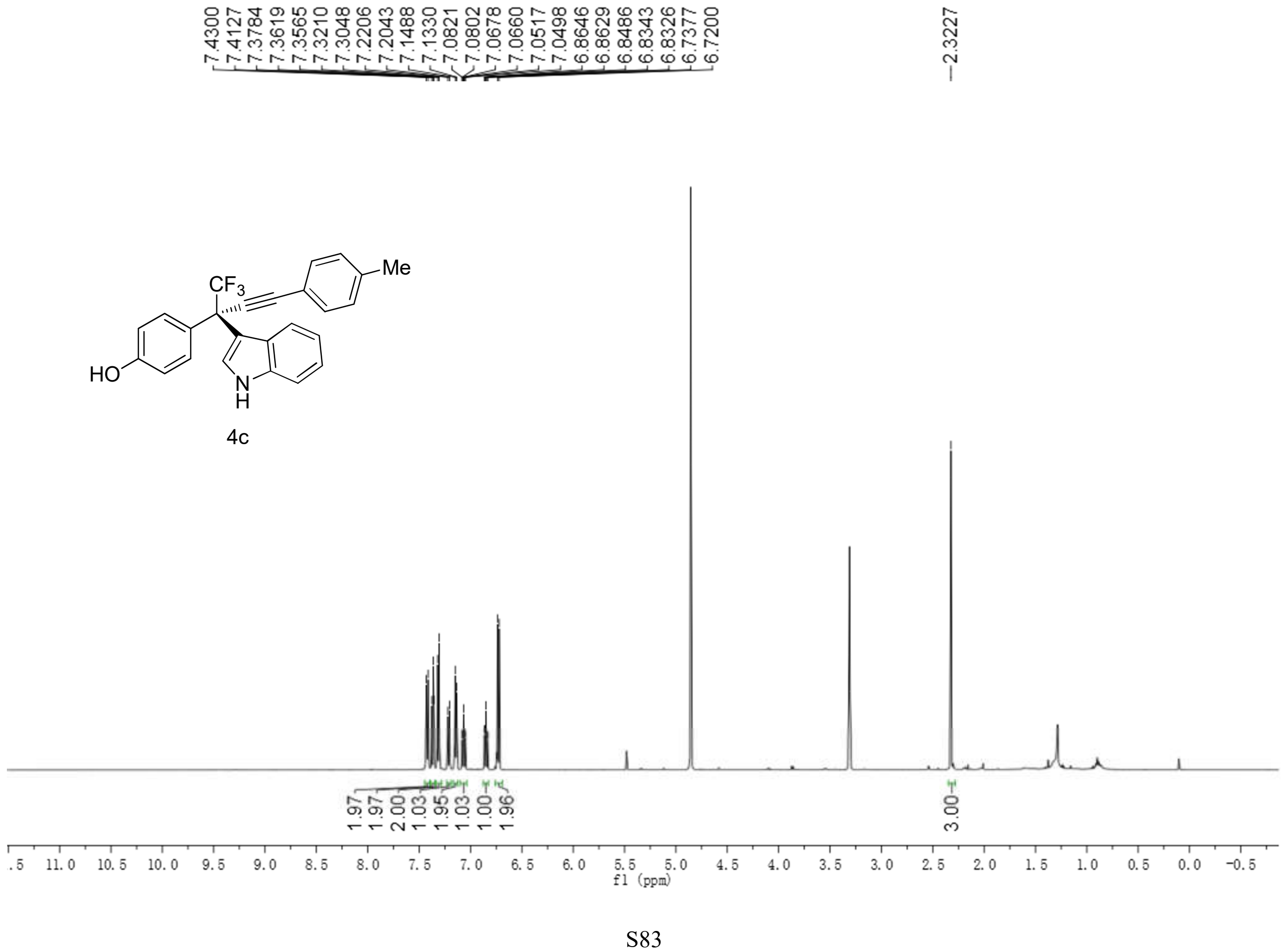

7.4300
7.4127
7.3784
7.3619
7.3565
7.3210
7.3048
7.2206
7.2043
7.1488
7.1330
7.0821
7.0802
7.0678
7.0660
7.0517
7.0498
6.8646
6.8629
6.8486
6.8343
6.8326
6.7377
6.7200
2.3227
CF3
Me
HO
N
H
4c
1.97
1.97
2.00
1.03
1.95
1.03
1.00
1.96
3.00
11.0 10.5 10.0 9.5 9.0 8.5 8.0 7.5 7.0 6.5 6.0 5.5 5.0 4.5 4.0 3.5 3.0 2.5 2.0 1.5 1.0 0.5 0.0 -0.5
f1 (ppm)

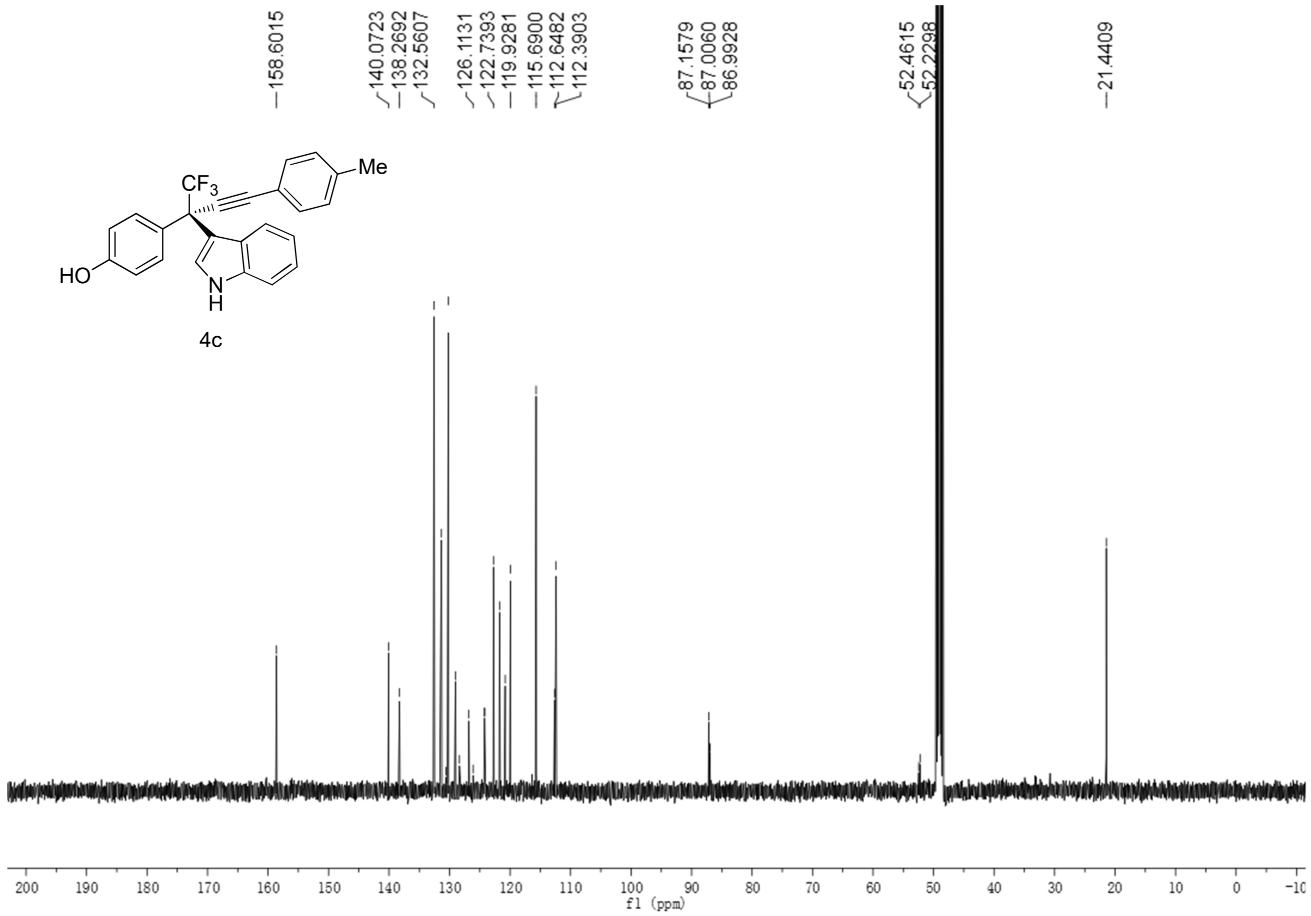
158.6015
140.0723
138.2692
132.5607
126.1131
122.7393
119.9281
115.6900
112.6482
112.3903
87.1579
87.0060
86.9928
52.4615
52.2298
21.4409
CF3
Me
HO
N
H
4c
f1 (ppm)

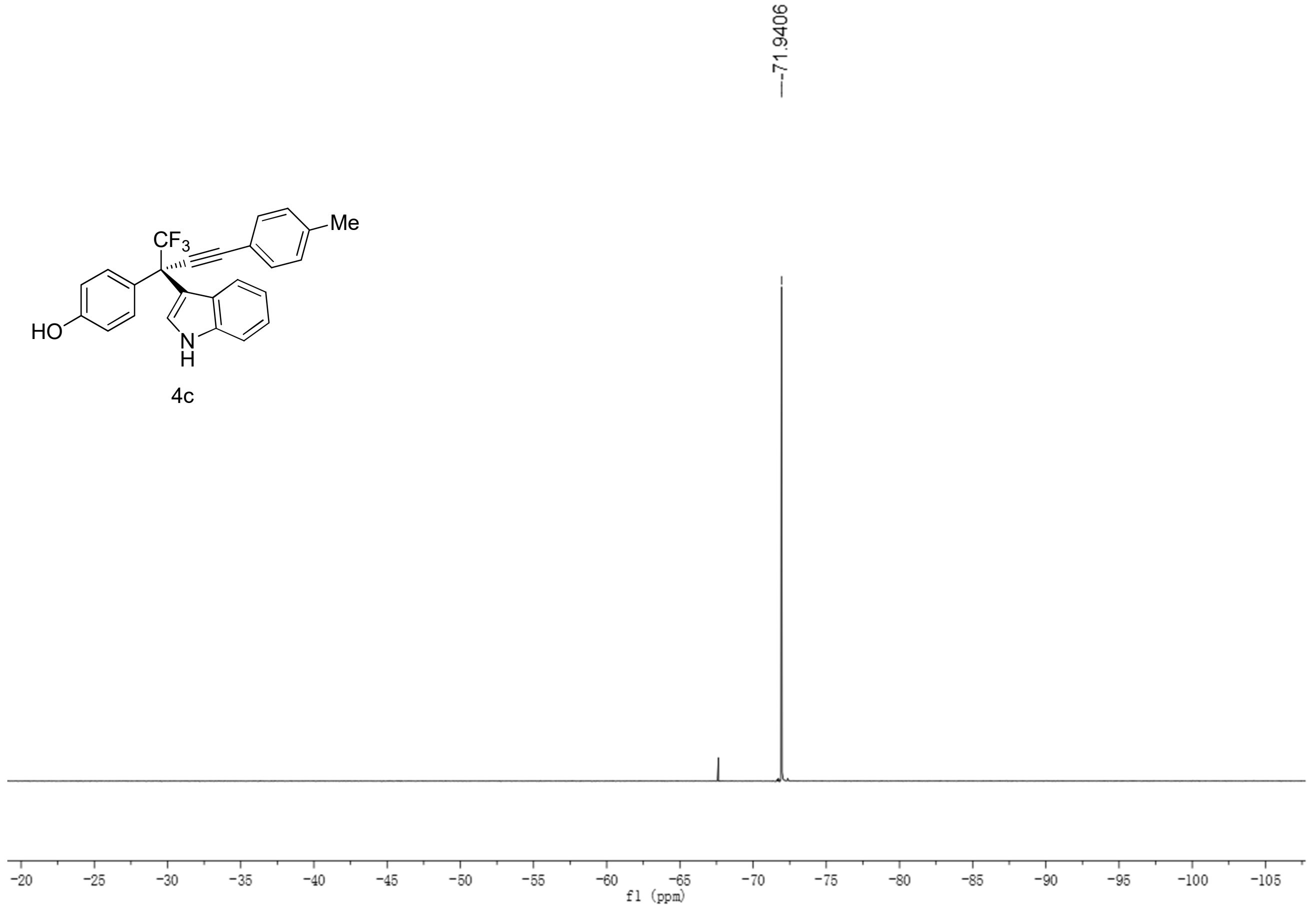
-71.9406
CF3
Me
HO
N
H
4c
f1 (ppm)

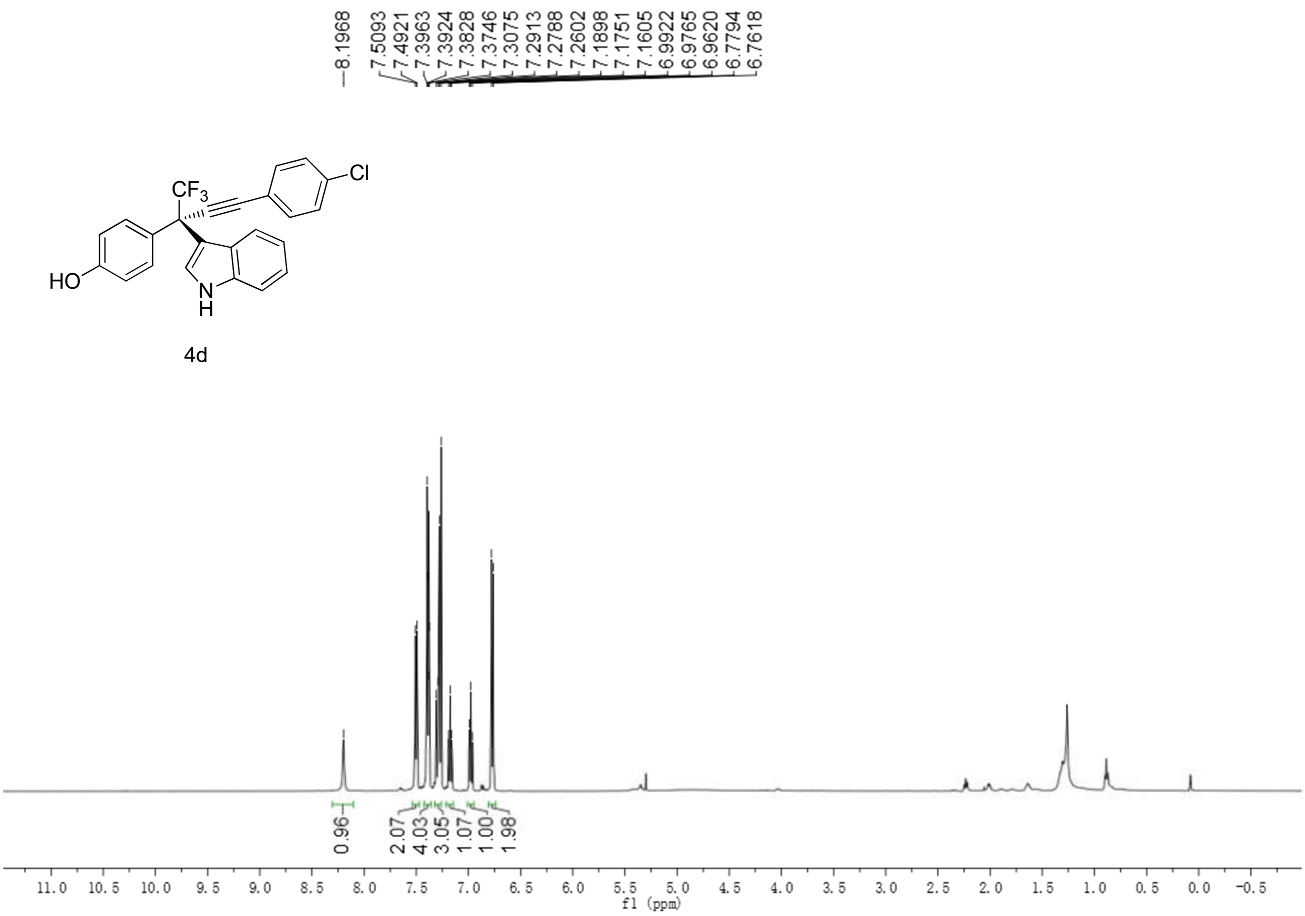
8.1968
7.5093
7.4921
7.3963
7.3924
7.3828
7.3746
7.3075
7.2913
7.2788
7.2602
7.1898
7.1751
7.1605
6.9922
6.9765
6.9620
6.7794
6.7618
CF3
Cl
HO
N
H
4d
0.96
2.07
4.03
3.05
1.07
1.00
1.98
f1 (ppm)

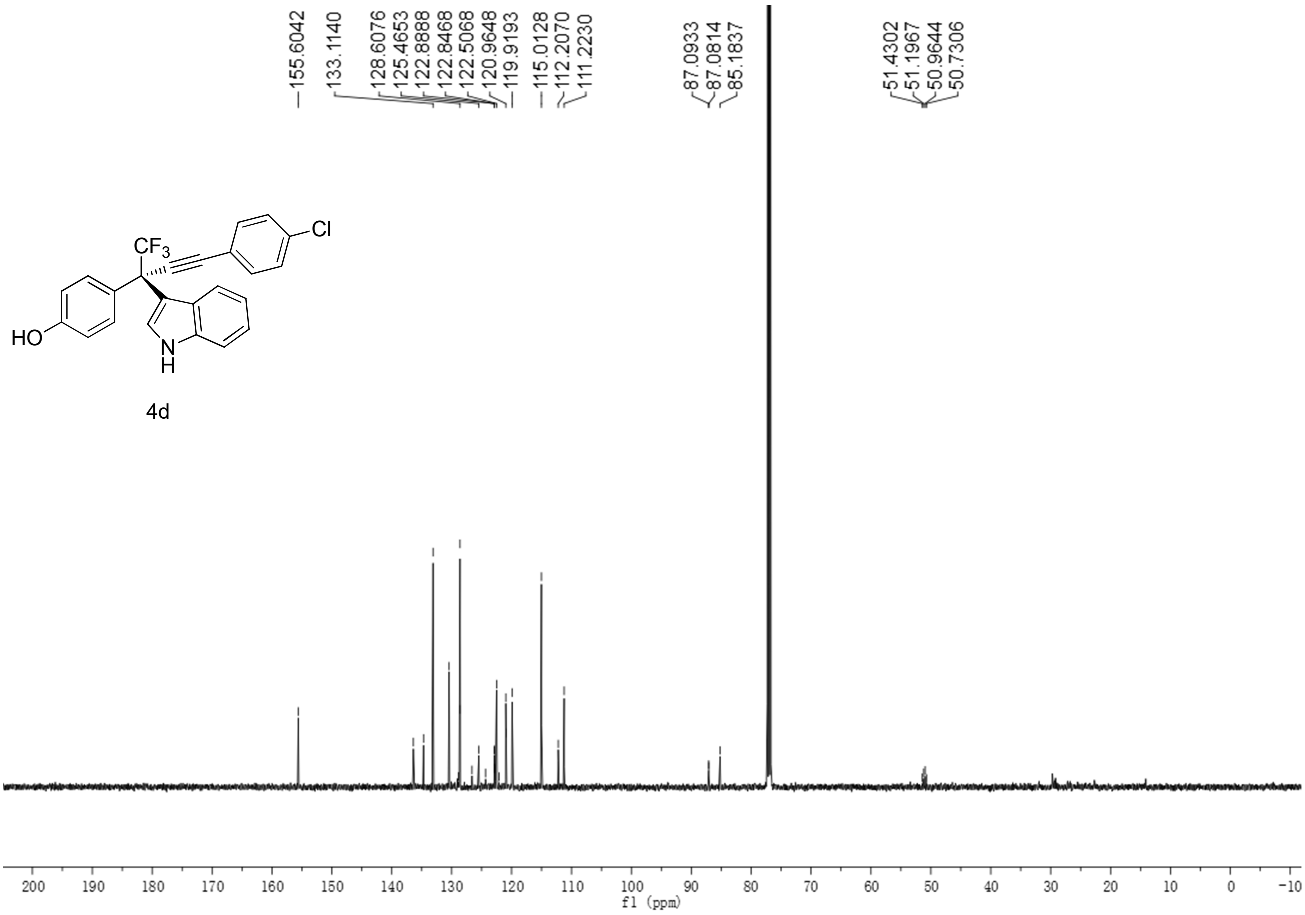
155.6042
133.1140
128.6076
125.4653
122.8888
122.8468
122.5068
120.9648
119.9193
115.0128
112.2070
111.2230
87.0933
87.0814
85.1837
51.4302
51.1967
50.9644
50.7306
CF3
Cl
HO
N
H
4d
f1 (ppm)

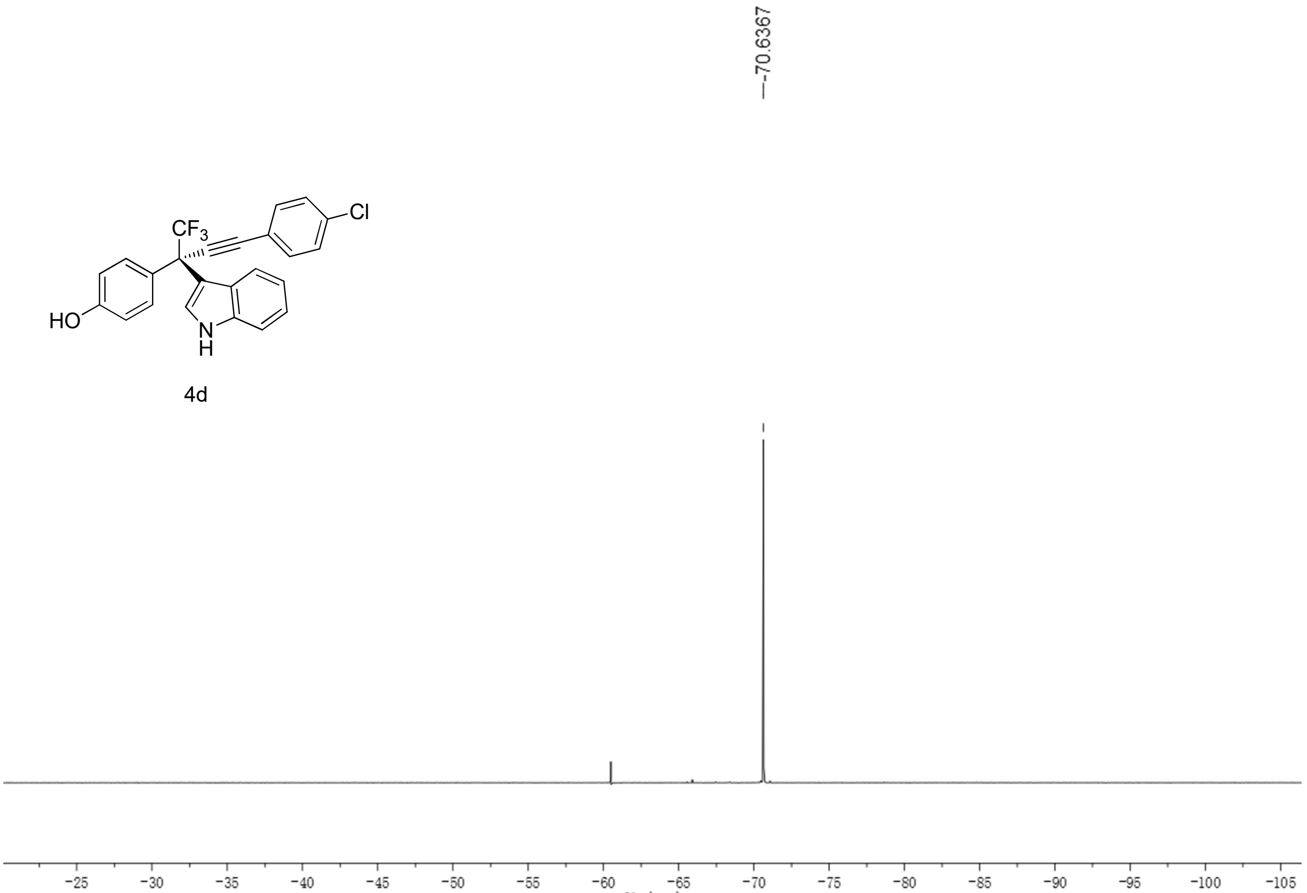

-70.6367
CF3
Cl
HO
N
H
4d
-25 -30 -35 -40 -45 -50 -55 -60 -65 -70 -75 -80 -85 -90 -95 -100 -105
f1 (ppm)

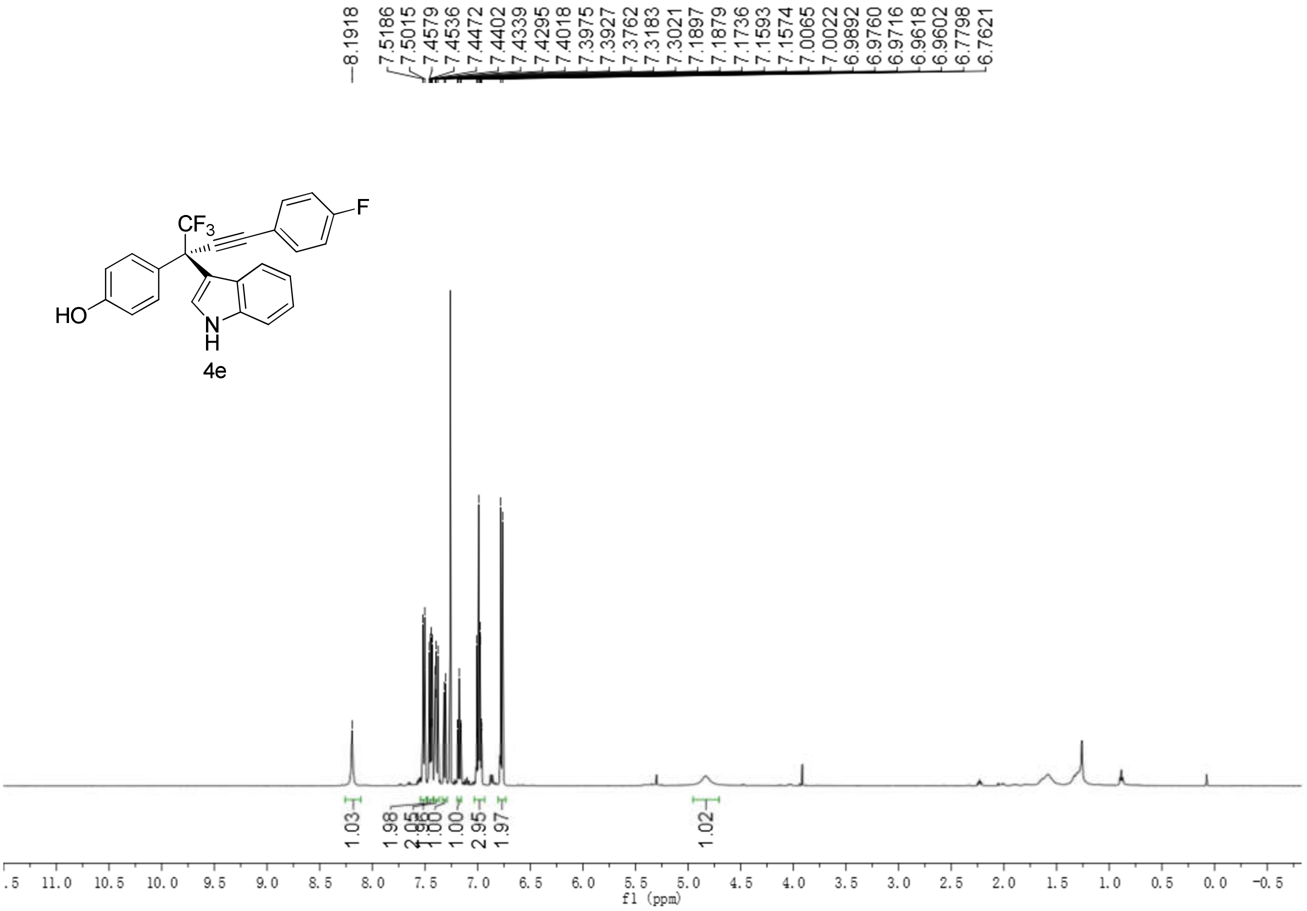

8.1918
7.5186
7.5015
7.4579
7.4536
7.4472
7.4402
7.4339
7.4295
7.4018
7.3975
7.3927
7.3762
7.3183
7.3021
7.1897
7.1879
7.1736
7.1593
7.1574
7.0065
7.0022
6.9892
6.9760
6.9716
6.9618
6.9602
6.7798
6.7621
CF3
F
HO
N
H
4e
1.03
1.98
2.05
1.96
1.00
2.95
1.97
1.02
.5 11.0 10.5 10.0 9.5 9.0 8.5 8.0 7.5 7.0 6.5 6.0 5.5 5.0 4.5 4.0 3.5 3.0 2.5 2.0 1.5 1.0 0.5 0.0 -0.5
f1 (ppm)

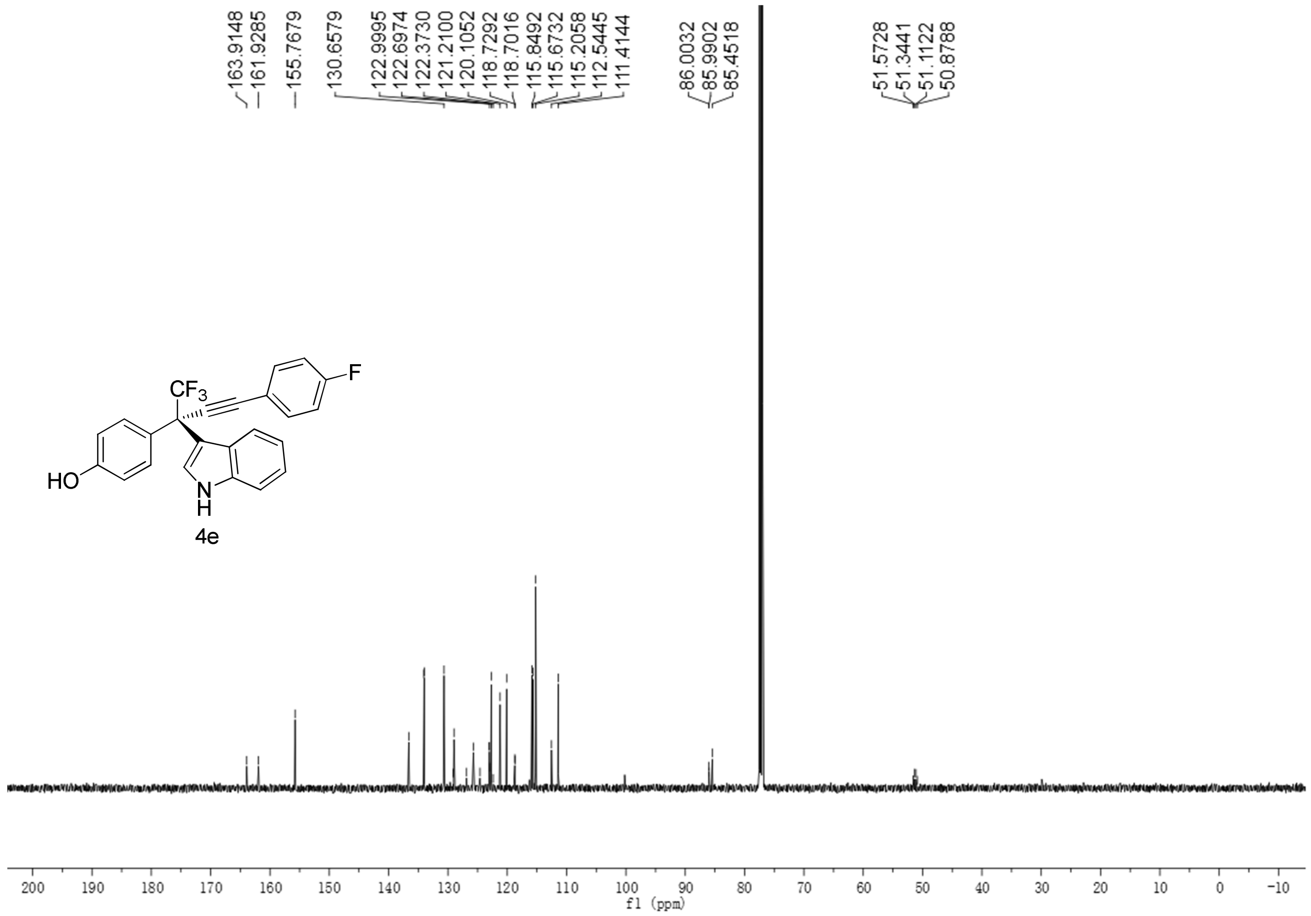
163.9148
161.9285
155.7679
130.6579
122.9995
122.6974
122.3730
121.2100
120.1052
118.7292
118.7016
115.8492
115.6732
115.2058
112.5445
111.4144
86.0032
85.9902
85.4518
51.5728
51.3441
51.1122
50.8788
CF3
F
HO
N
H
4e
f1 (ppm)

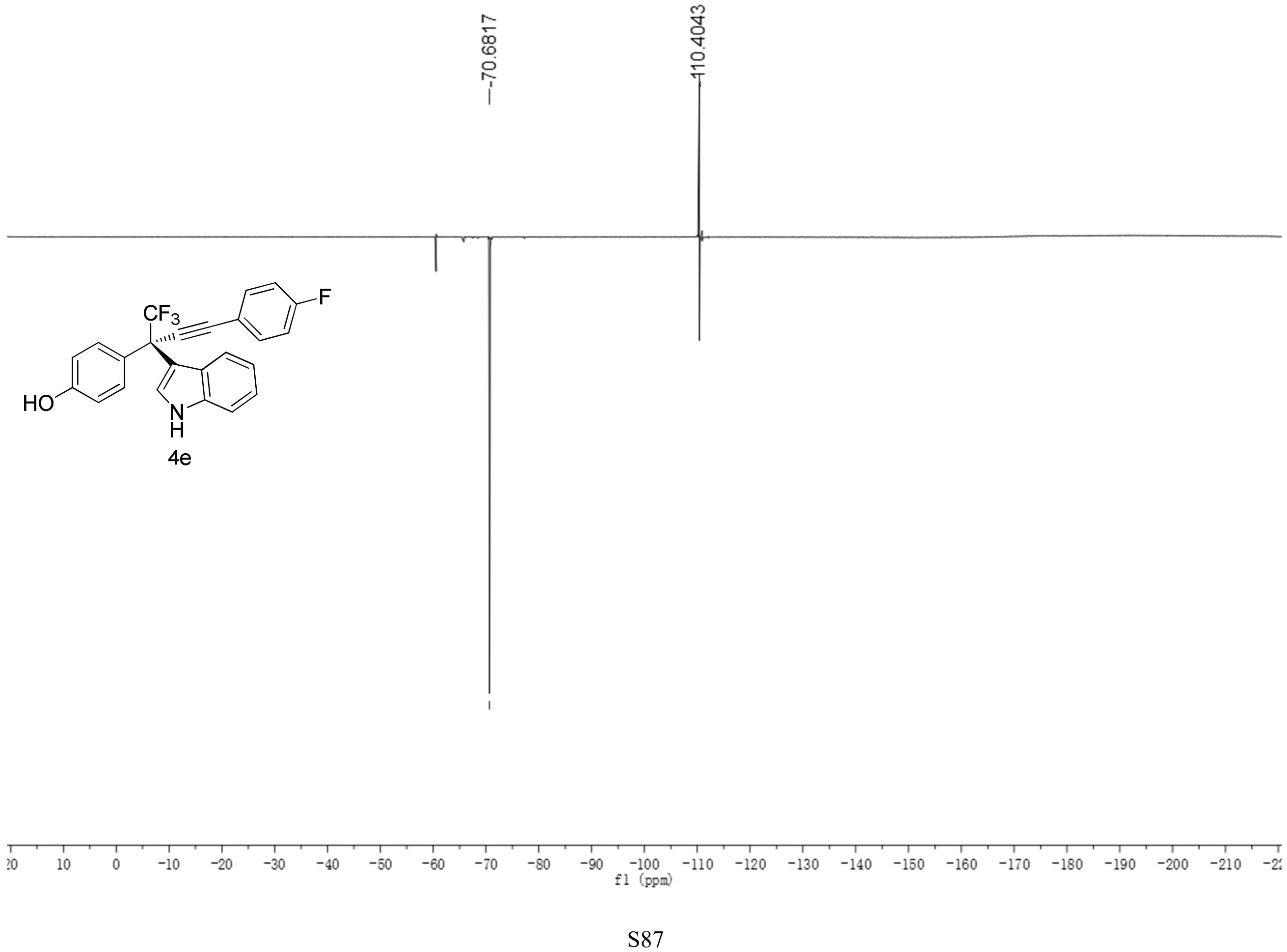
-70.6817
-110.4043
CF3
F
HO
N
H
4e
f1 (ppm)

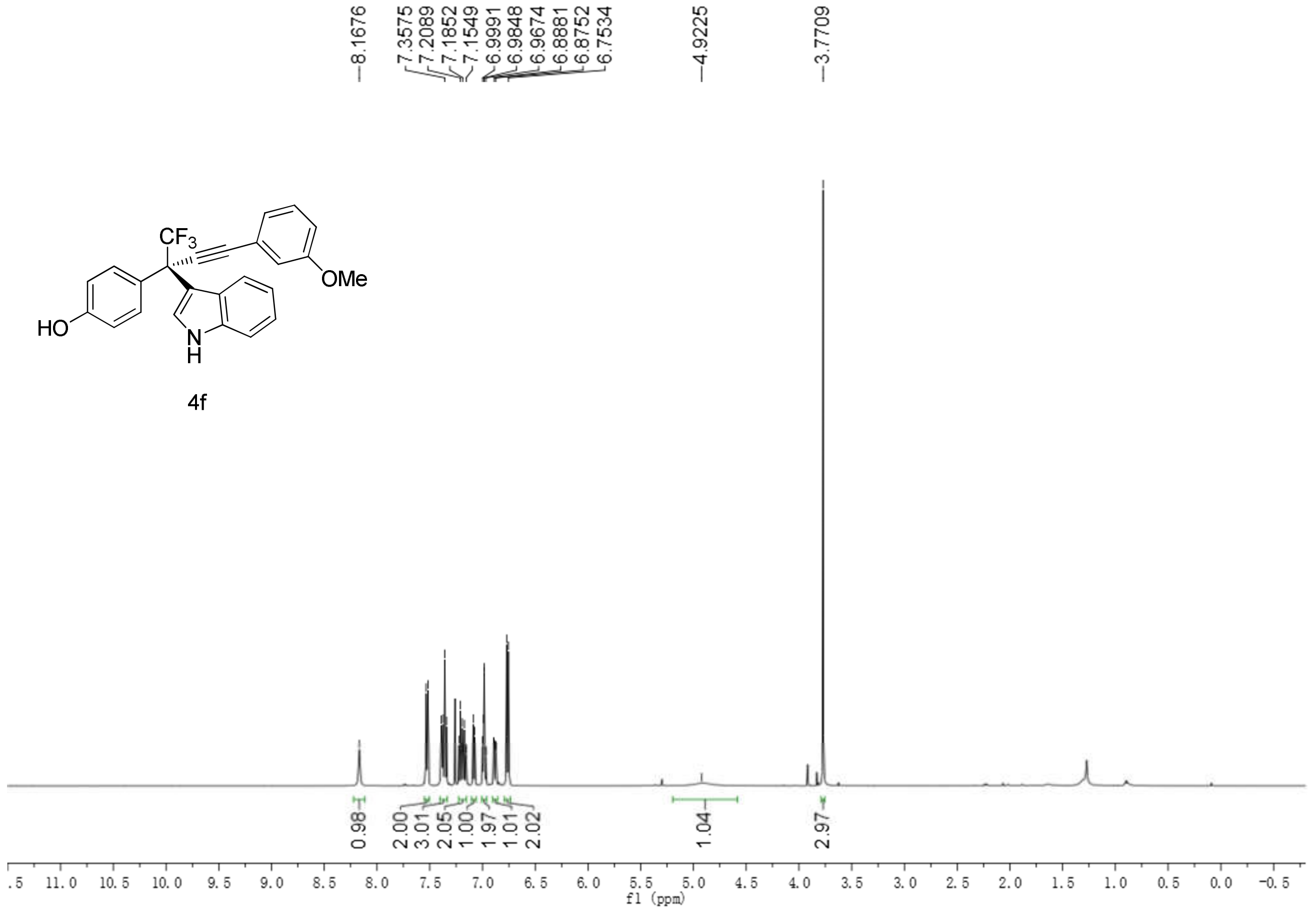
8.1676
7.3575
7.2089
7.1852
7.1549
6.9991
6.9848
6.9674
6.8881
6.8752
6.7534
4.9225
3.7709
CF3
OMe
HO
N
H
4f
0.98
2.00
3.01
2.05
1.00
1.97
1.01
2.02
1.04
2.97
f1 (ppm)

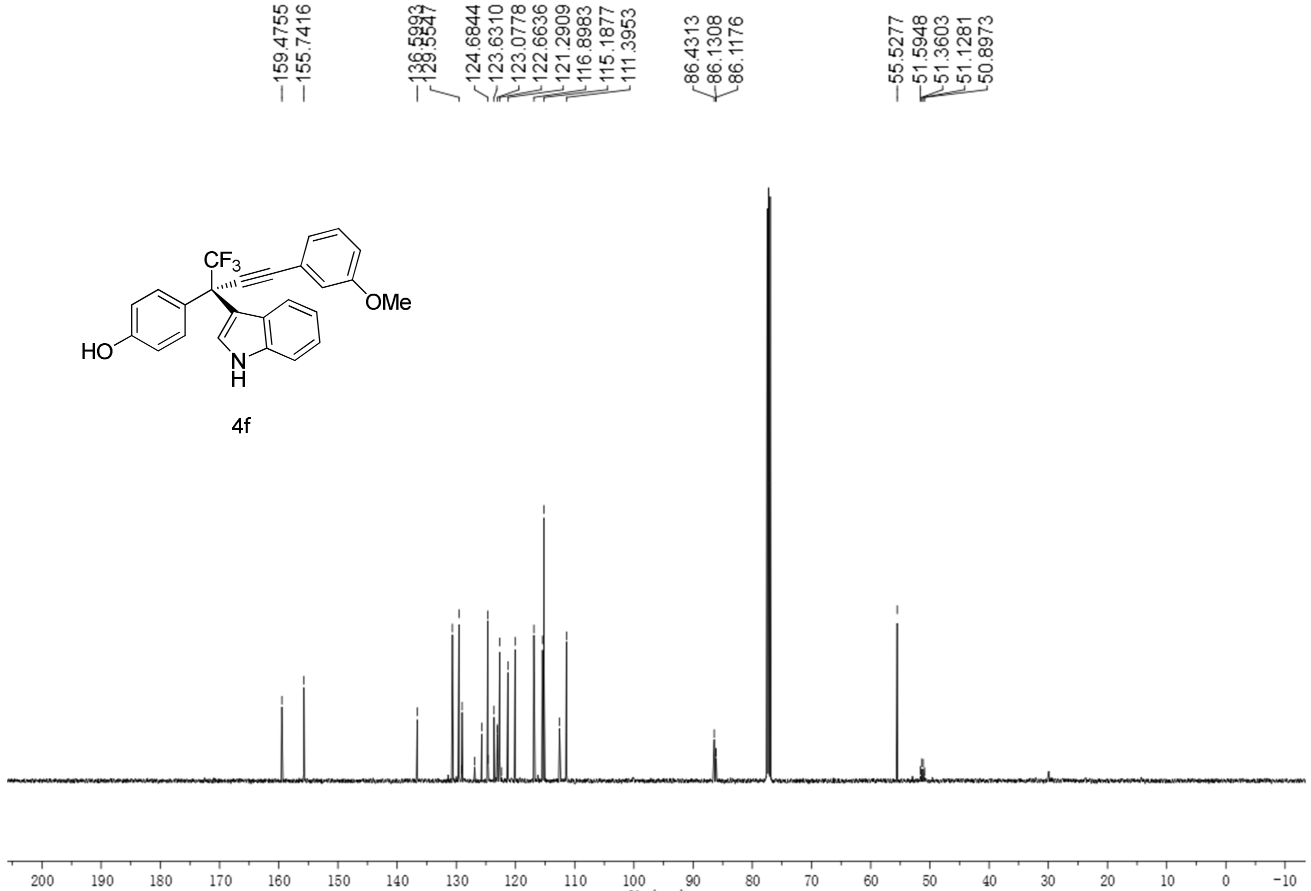
159.4755
155.7416
136.5993
129.5547
124.6844
123.6310
123.0778
122.6636
121.2909
116.8983
115.1877
111.3953
86.4313
86.1308
86.1176
55.5277
51.5948
51.3603
51.1281
50.8973
CF3
OMe
HO
N
H
4f
f1 (ppm)

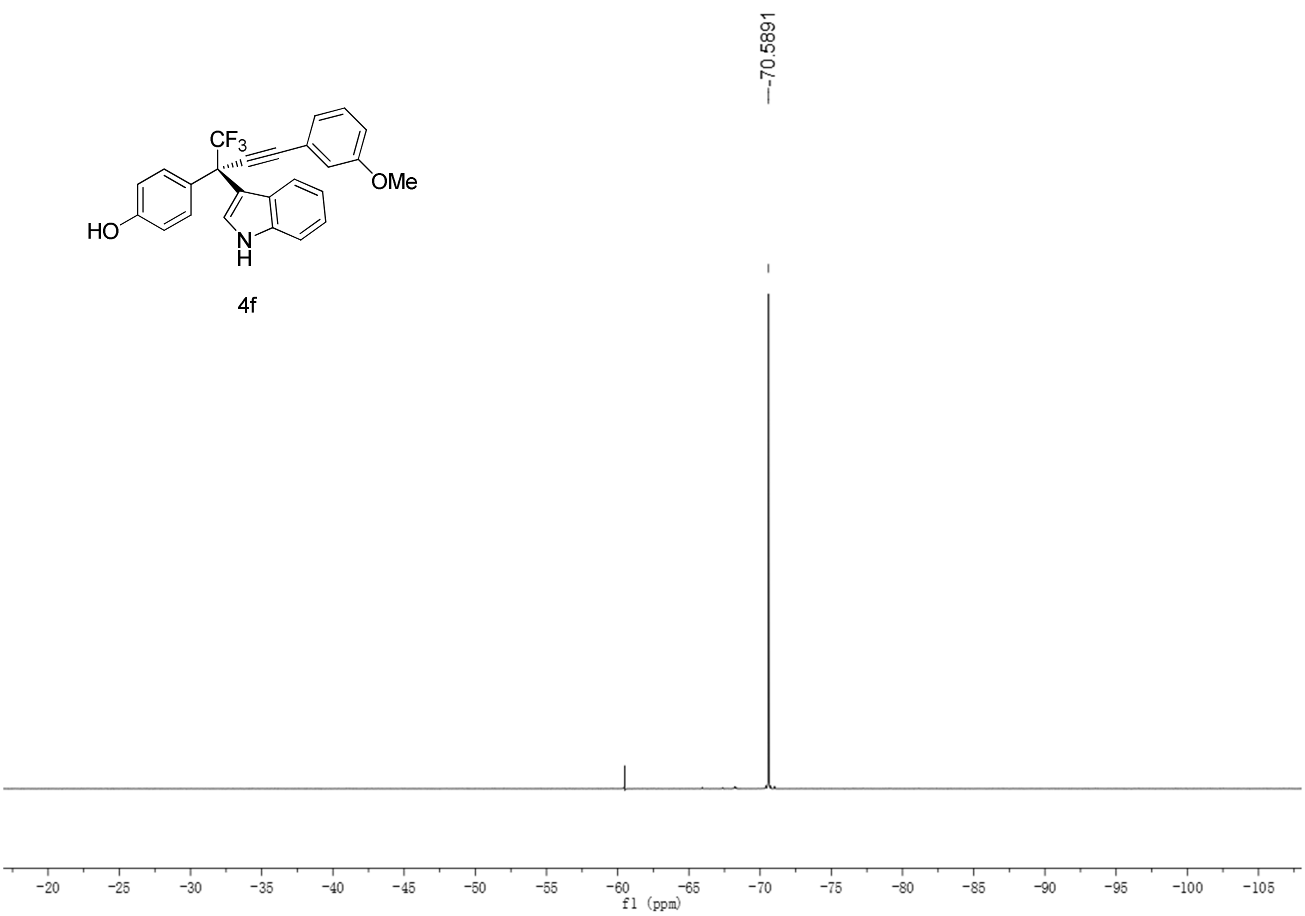
-70.5891
CF3
OMe
HO
N
H
4f
f1 (ppm)

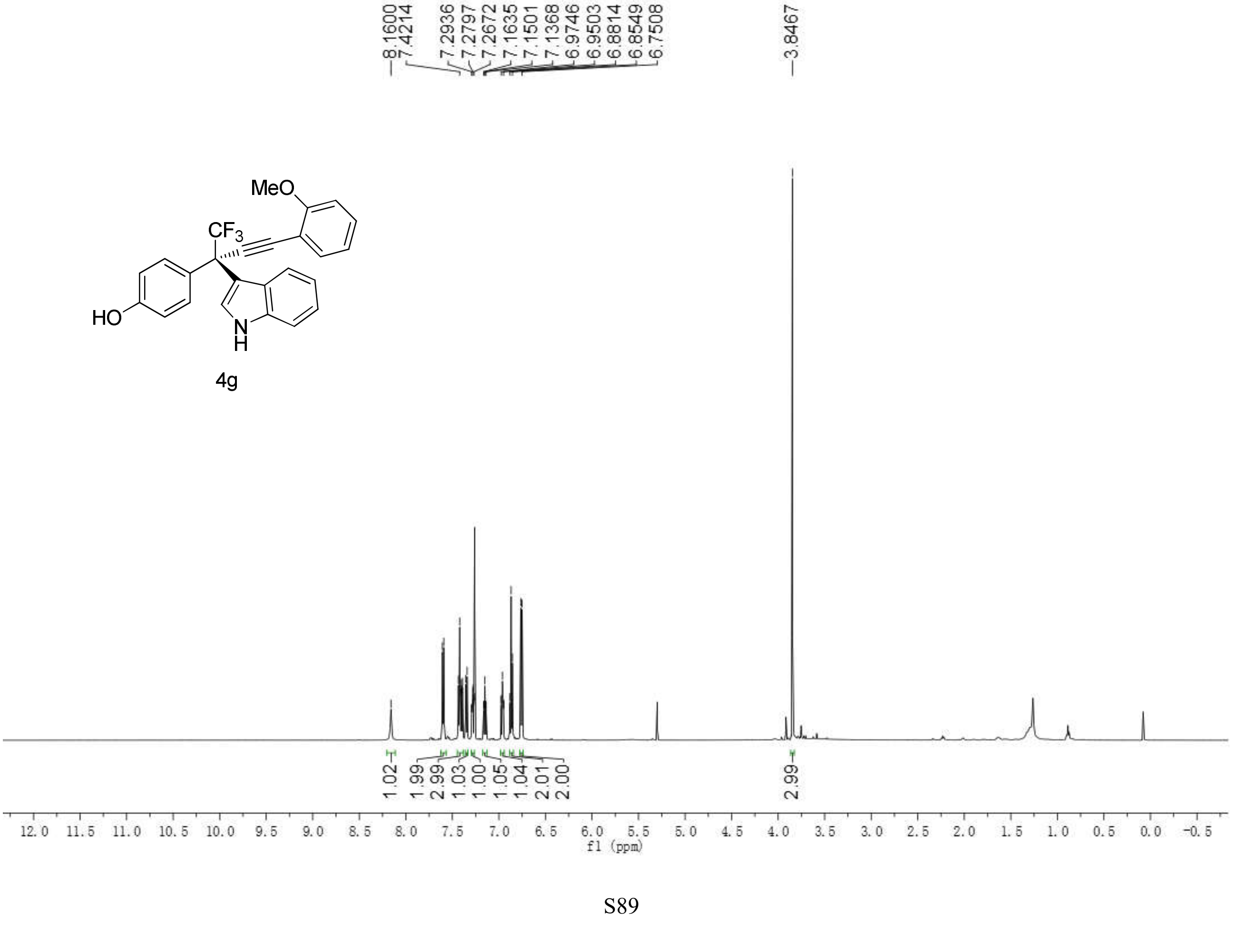
8.1600
7.4214
7.2936
7.2797
7.2672
7.1635
7.1501
7.1368
6.9746
6.9503
6.8814
6.8549
6.7508
3.8467
MeO
CF3
HO
N
H
4g
1.02
1.99
2.99
1.03
1.00
1.05
1.04
2.01
2.00
2.99
f1 (ppm)

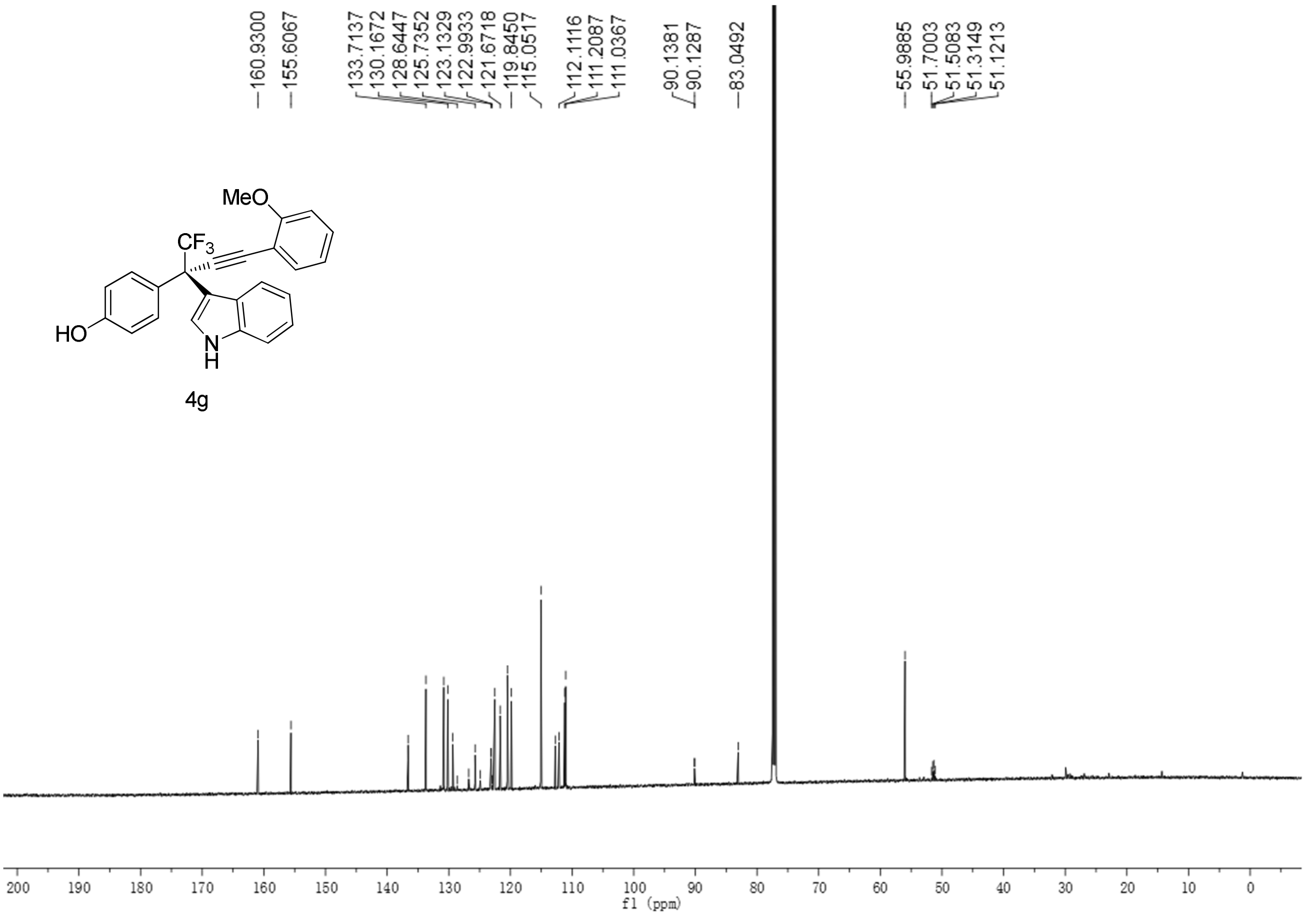
160.9300
155.6067
133.7137
130.1672
128.6447
125.7352
123.1329
122.9933
121.6718
119.8450
115.0517
112.1116
111.2087
111.0367
90.1381
90.1287
83.0492
55.9885
51.7003
51.5083
51.3149
51.1213
MeO
CF3
HO
N
H
4g
200 190 180 170 160 150 140 130 120 110 100 90 80 70 60 50 40 30 20 10 0
f1 (ppm)

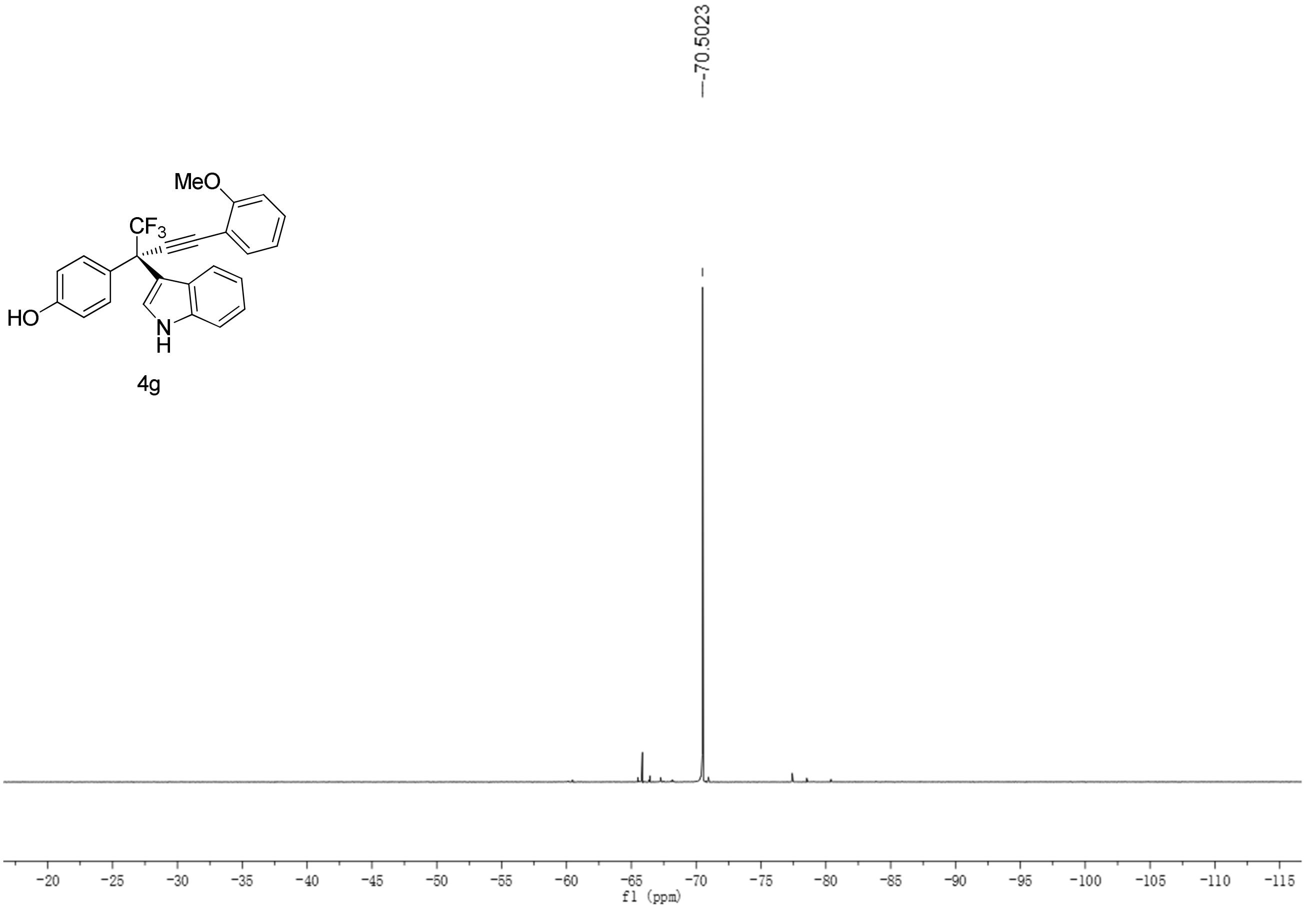
-70.5023
MeO
CF3
HO
N
H
4g
-20 -25 -30 -35 -40 -45 -50 -55 -60 -65 -70 -75 -80 -85 -90 -95 -100 -105 -110 -115
f1 (ppm)

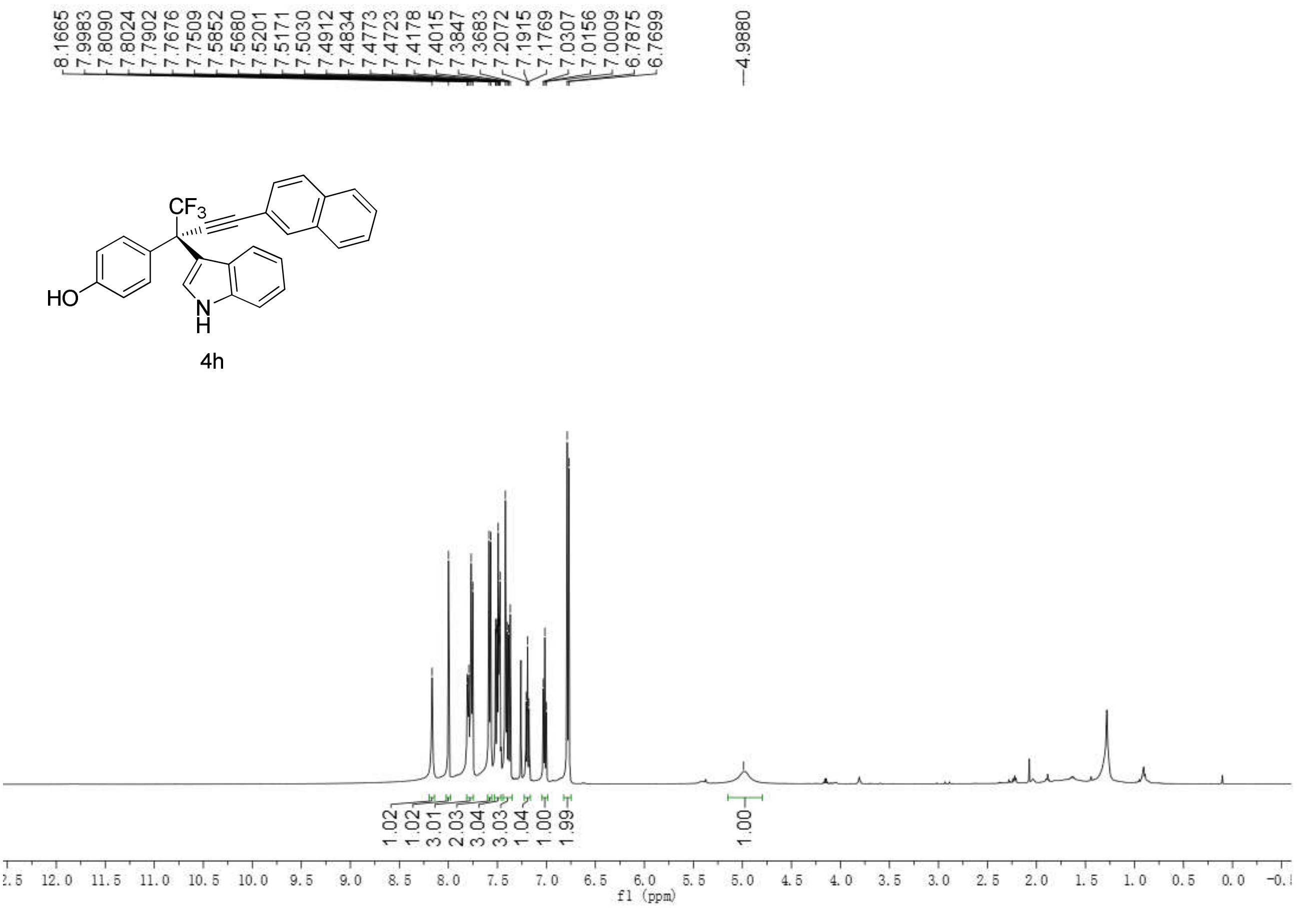
8.1665
7.9983
7.8090
7.8024
7.7902
7.7676
7.7509
7.5852
7.5680
7.5201
7.5171
7.5030
7.4912
7.4834
7.4773
7.4723
7.4178
7.4015
7.3847
7.3683
7.2072
7.1915
7.1769
7.0307
7.0156
7.0009
6.7875
6.7699
4.9880
CF3
HO
N
H
4h
1.02
1.02
3.01
2.03
3.04
3.03
1.04
1.00
1.99
1.00
f1 (ppm)

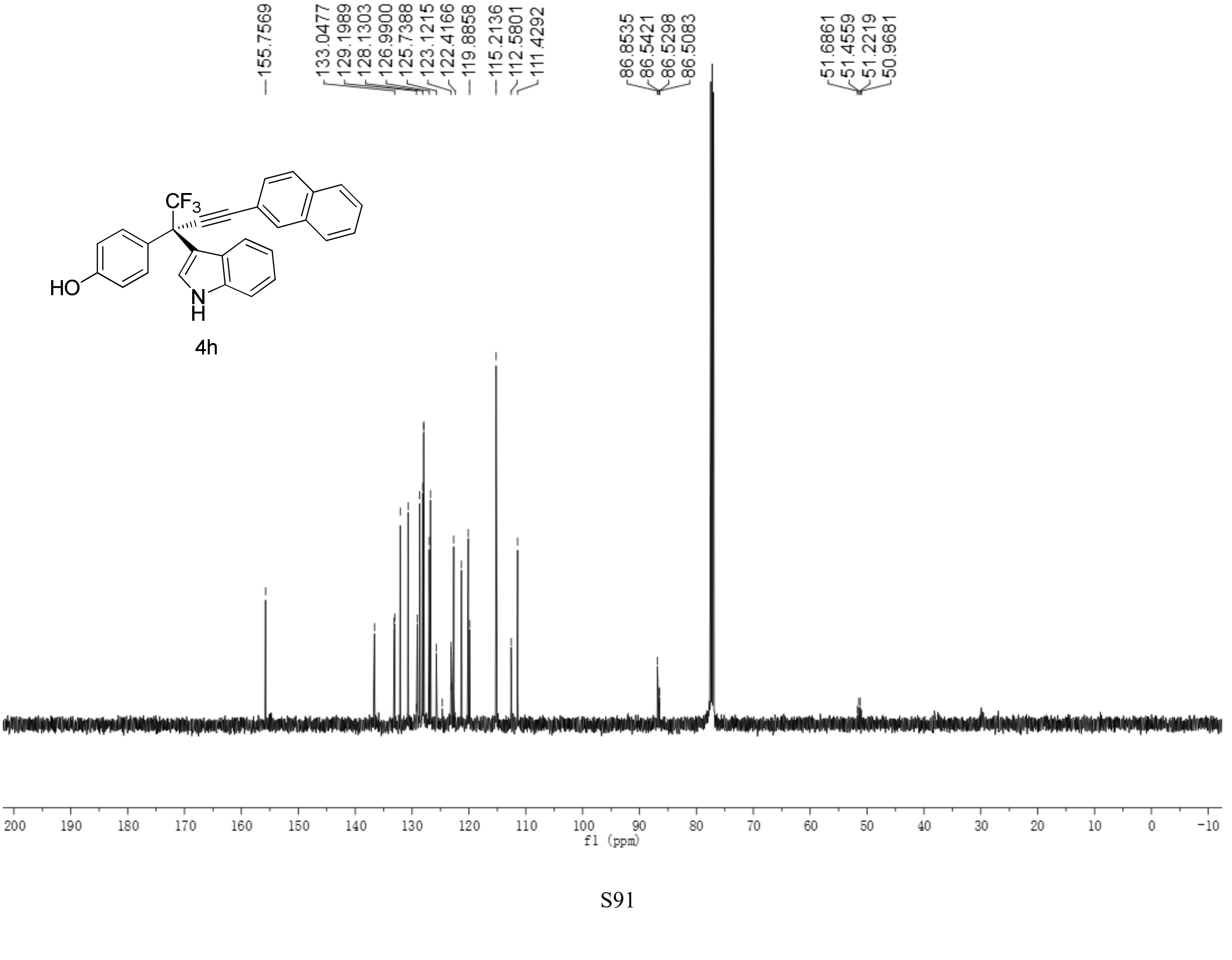
155.7569
133.0477
129.1989
128.1303
126.9900
125.7388
123.1215
122.4166
119.8858
115.2136
112.5801
111.4292
86.8535
86.5421
86.5298
86.5083
51.6861
51.4559
51.2219
50.9681
CF3
HO
N
H
4h
f1 (ppm)

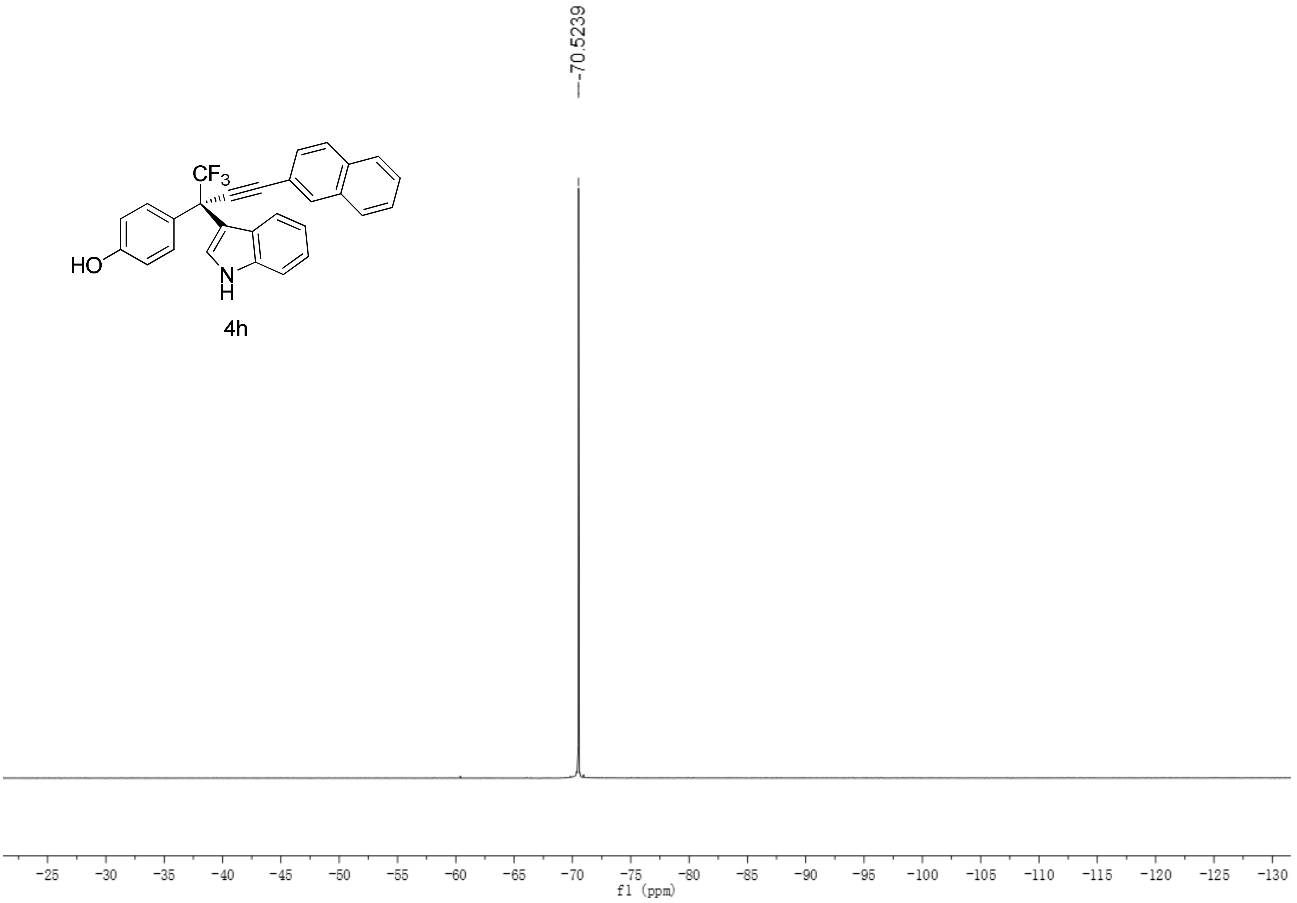
-70.5239
CF3
HO
N
H
4h
f1 (ppm)

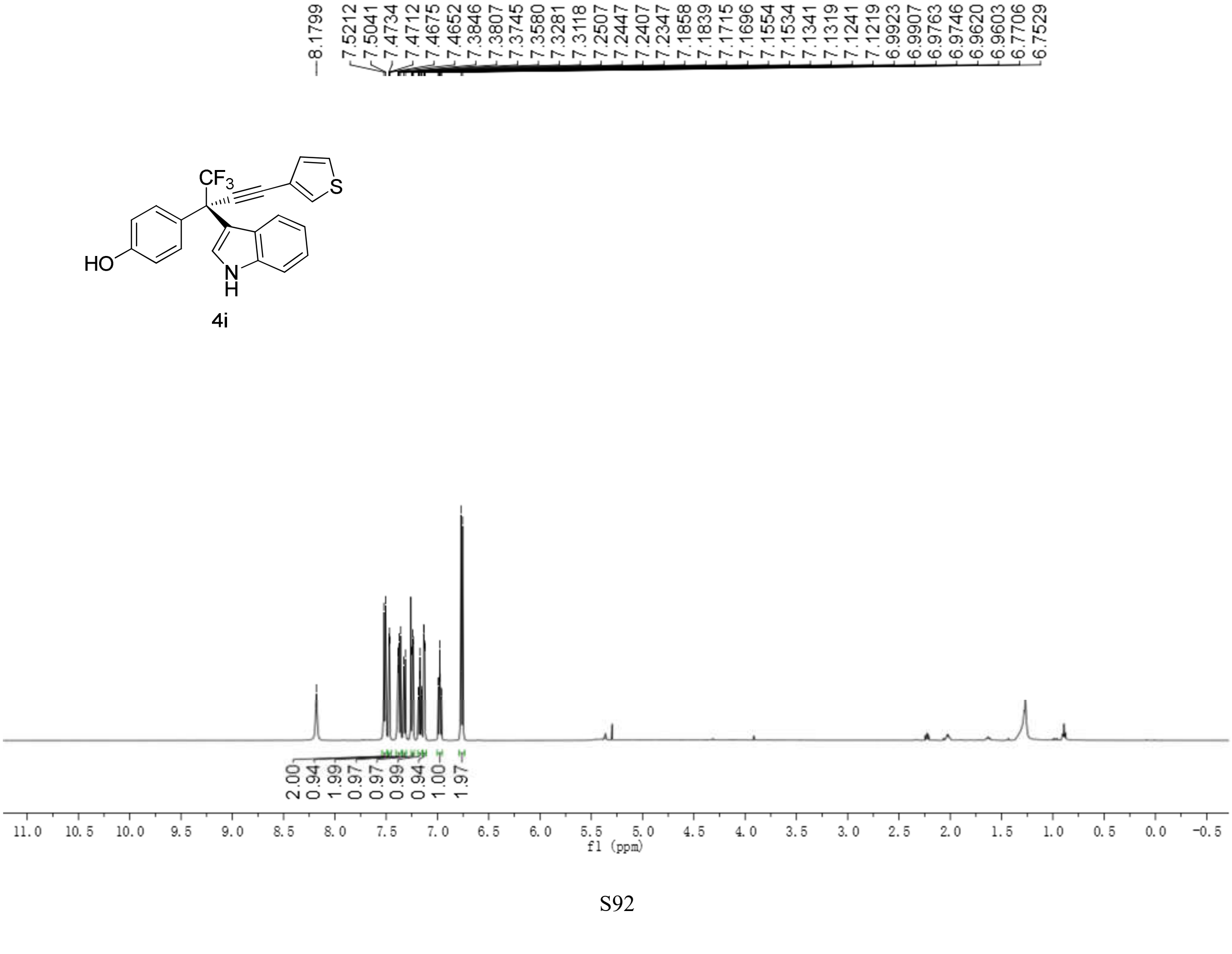
8.1799
7.5212
7.5041
7.4734
7.4712
7.4675
7.4652
7.3846
7.3807
7.3745
7.3580
7.3281
7.3118
7.2507
7.2447
7.2407
7.2347
7.1858
7.1839
7.1715
7.1696
7.1554
7.1534
7.1341
7.1319
7.1241
7.1219
6.9923
6.9907
6.9763
6.9746
6.9620
6.9603
6.7706
6.7529
CF3
S
HO
N
H
4i
2.00
0.94
1.99
0.97
0.97
0.99
0.94
1.00
1.97
f1 (ppm)

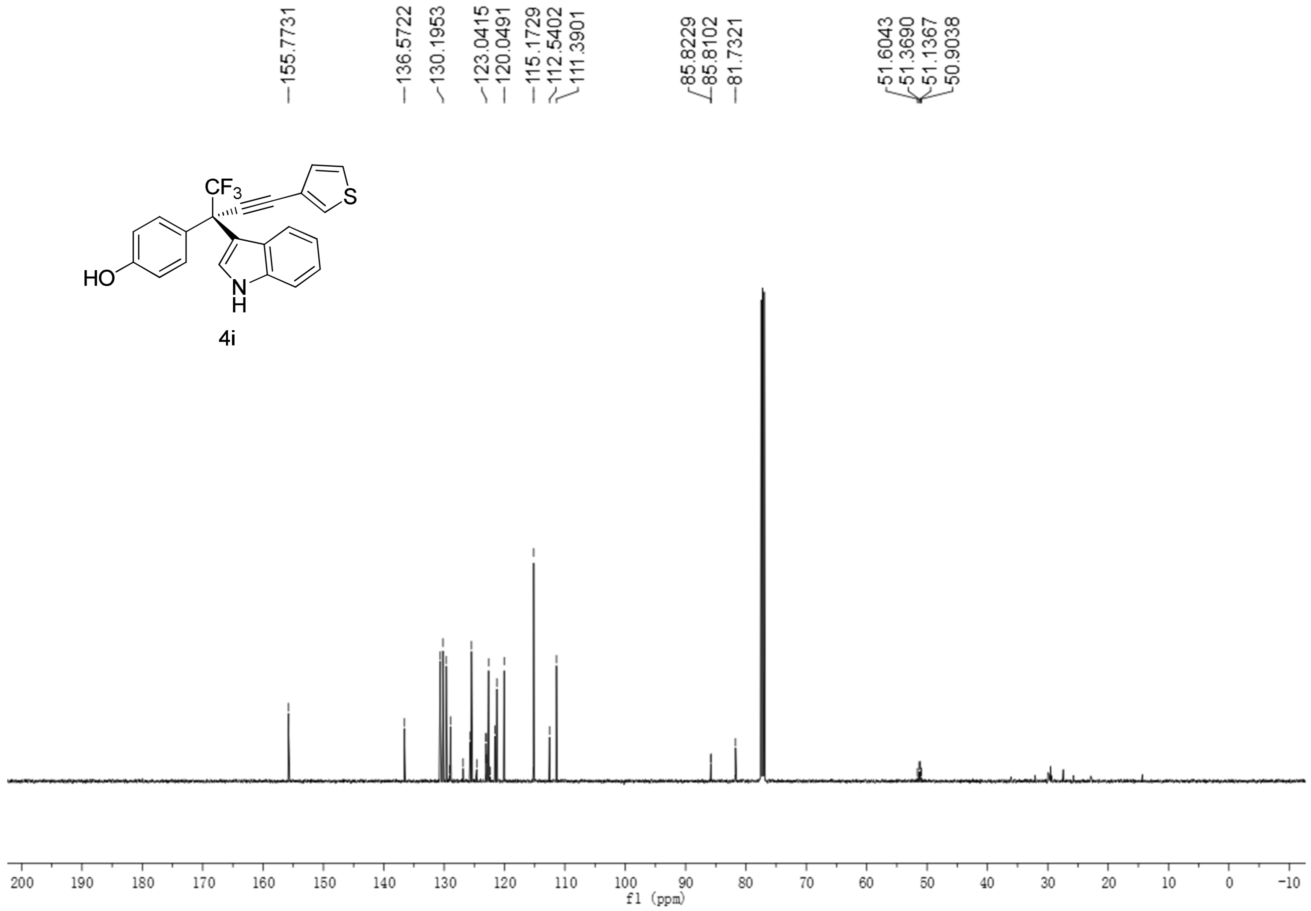
155.7731
136.5722
130.1953
123.0415
120.0491
115.1729
112.5402
111.3901
85.8229
85.8102
81.7321
51.6043
51.3690
51.1367
50.9038
CF3
S
HO
N
H
4i
f1 (ppm)

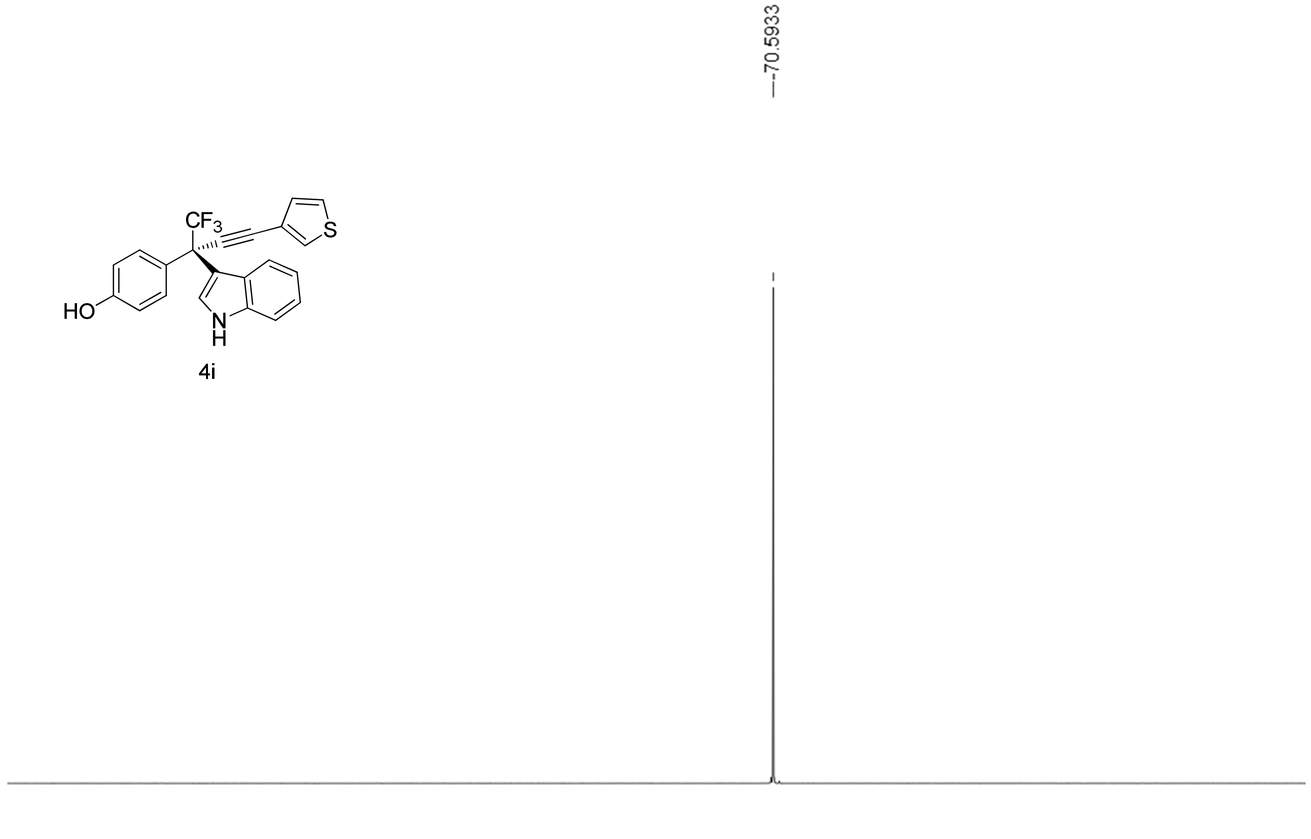
-70.5933
CF3
S
HO
N
H
4i
f1 (ppm)

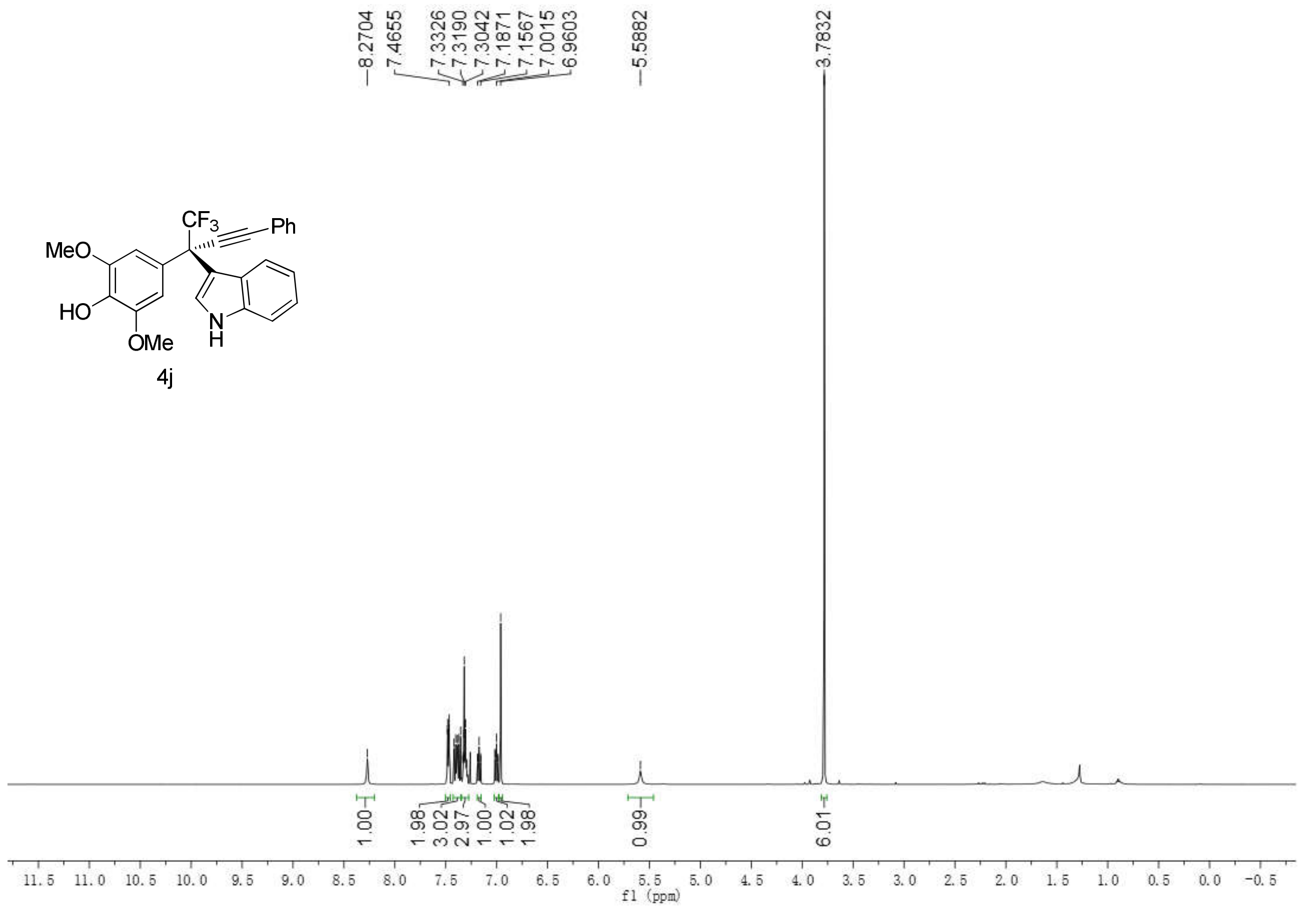

8.2704
7.4655
7.3326
7.3190
7.3042
7.1871
7.1567
7.0015
6.9603
5.5882
3.7832
CF3
Ph
MeO
HO
OMe
N
H
1.00
1.98
3.02
2.97
1.00
1.02
1.98
0.99
6.01
f1 (ppm)

4j

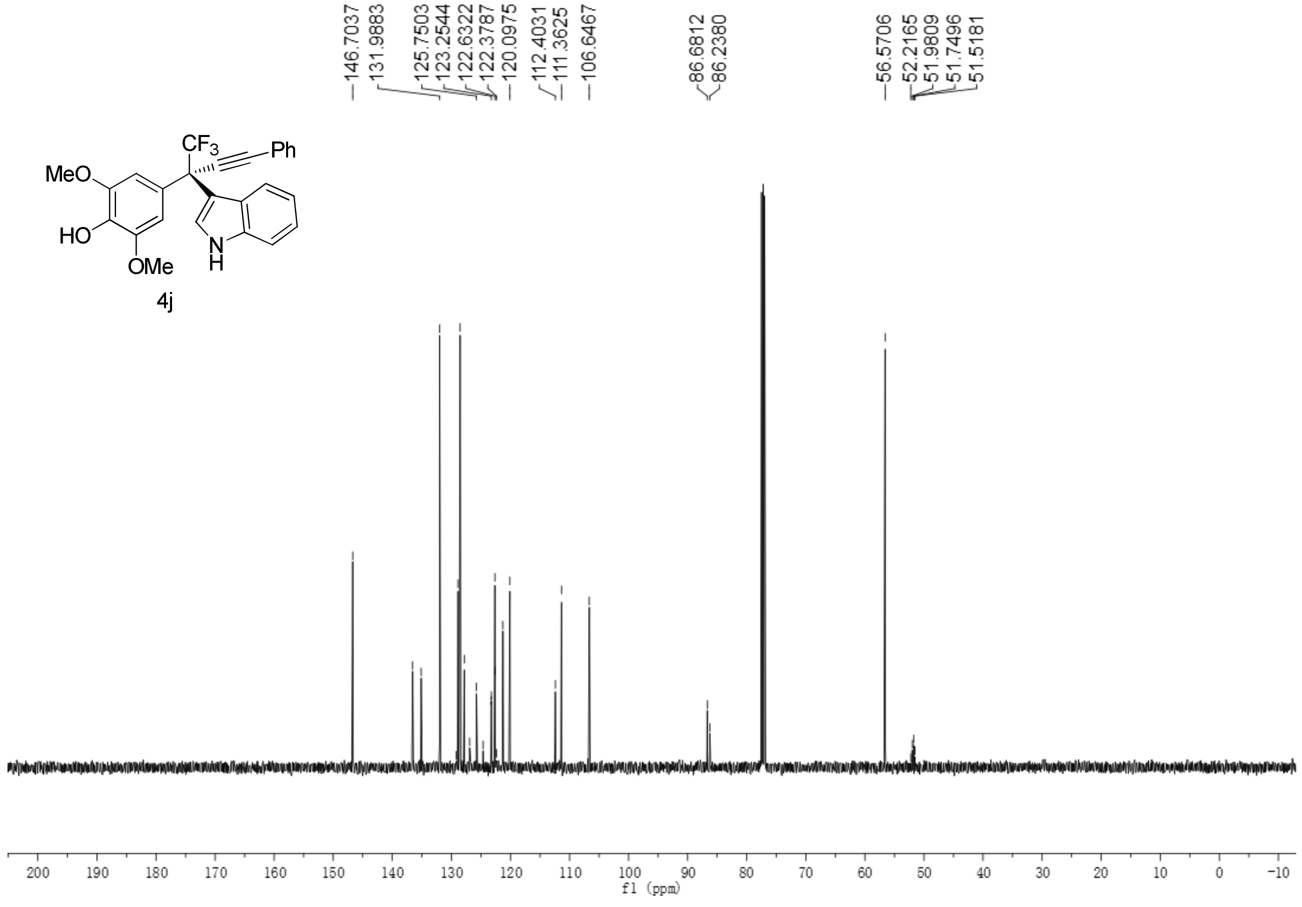

146.7037
131.9883
125.7503
123.2544
122.6322
122.3787
120.0975
112.4031
111.3625
106.6467
86.6812
86.2380
56.5706
52.2165
51.9809
51.7496
51.5181
CF3
Ph
MeO
HO
OMe
N
H
f1 (ppm)

4j

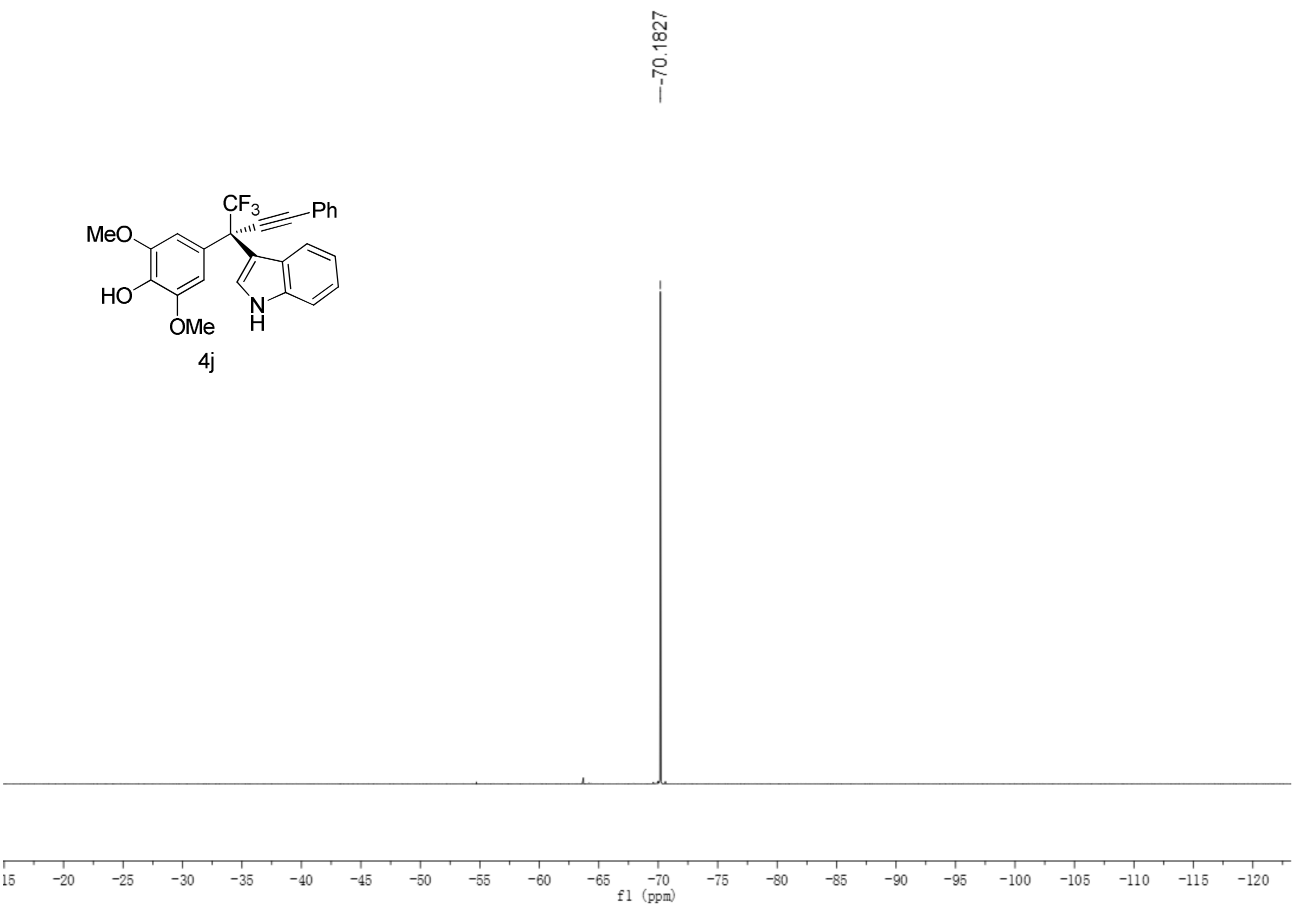

-70.1827
CF3
Ph
MeO
HO
OMe
N
H
4j
15 -20 -25 -30 -35 -40 -45 -50 -55 -60 -65 -70 -75 -80 -85 -90 -95 -100 -105 -110 -115 -120
f1 (ppm)

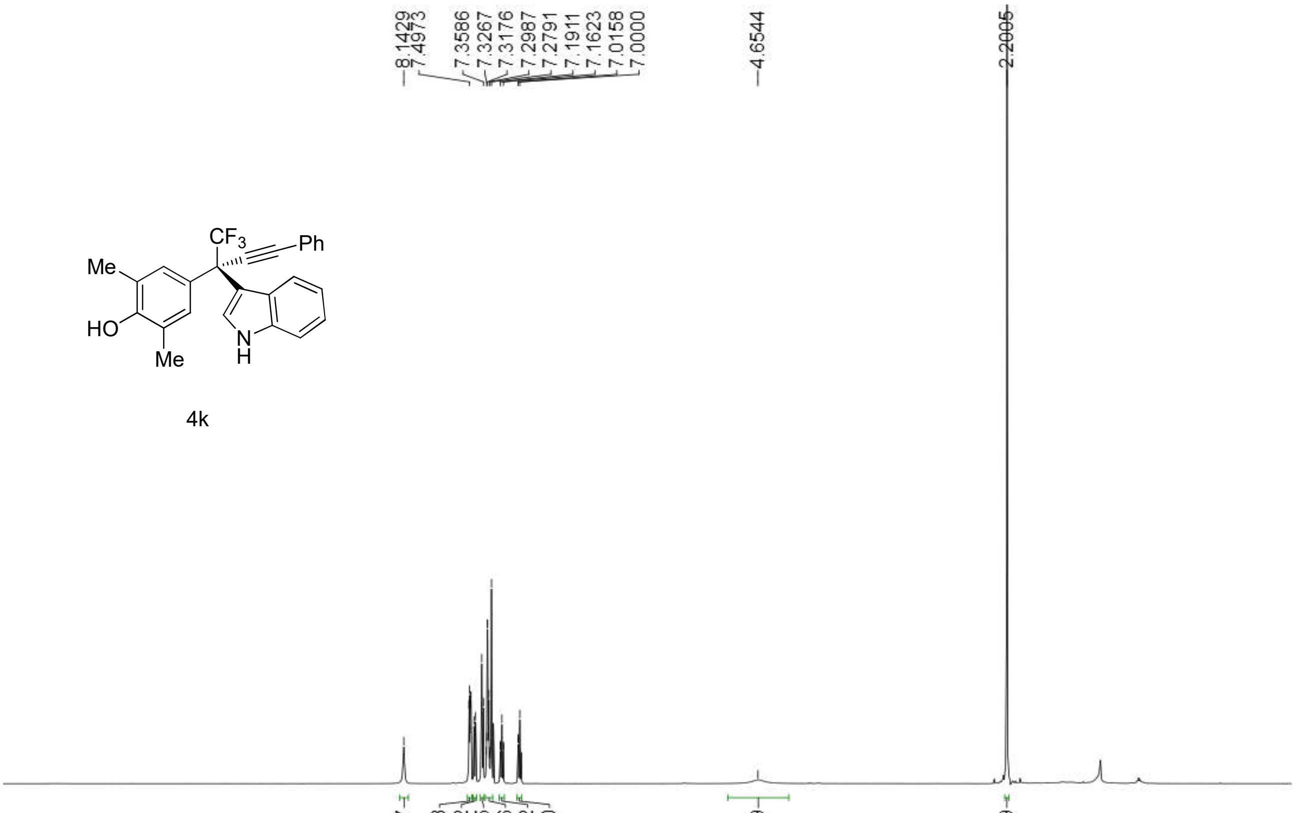

8.1429
7.4973
7.3586
7.3267
7.3176
7.2987
7.2791
7.1911
7.1623
7.0158
7.0000
4.6544
2.2005
CF3
Ph
Me
HO
Me
N
H
4k
0.97
1.98
1.02
2.05
4.96
1.02
1.00
0.99
5.99
11.5 11.0 10.5 10.0 9.5 9.0 8.5 8.0 7.5 7.0 6.5 6.0 5.5 5.0 4.5 4.0 3.5 3.0 2.5 2.0 1.5 1.0 0.5 0.0
f1 (ppm)

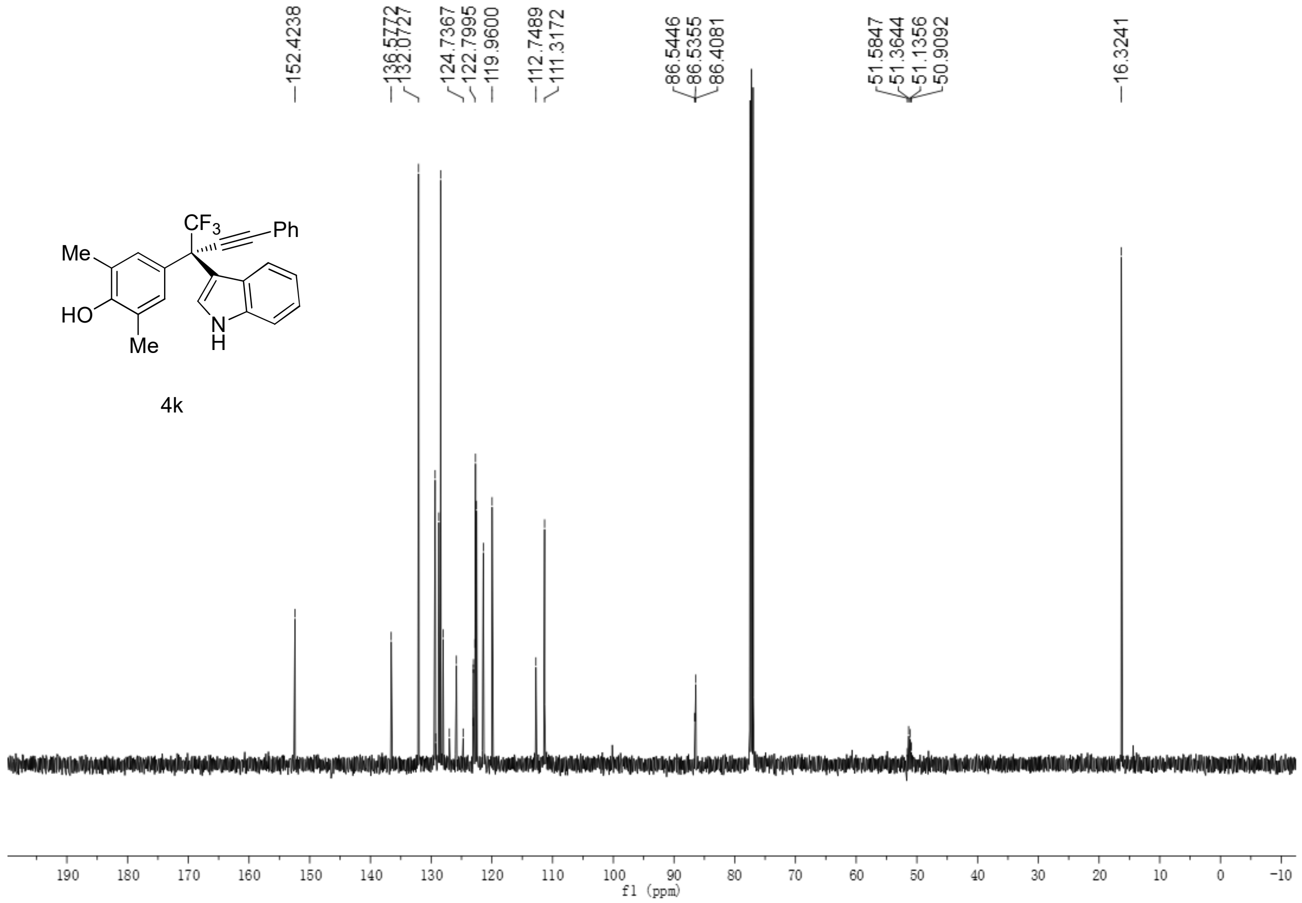
152.4238
136.5772
132.0727
124.7367
122.7995
119.9600
112.7489
111.3172
86.5446
86.5355
86.4081
51.5847
51.3644
51.1356
50.9092
16.3241
CF3
Ph
Me
HO
Me
N
H
4k
f1 (ppm)

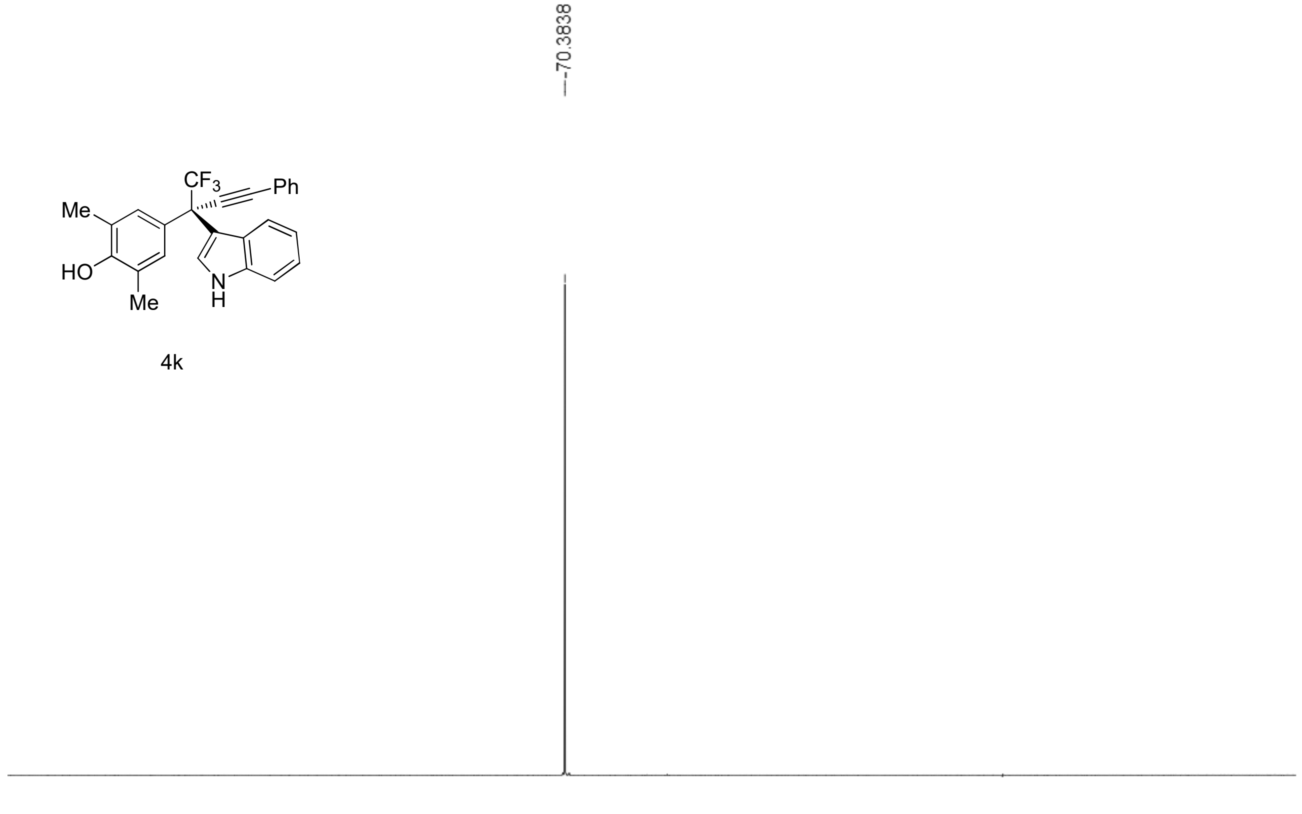
-70.3838
CF3
Ph
Me
HO
Me
N
H
4k
f1 (ppm)

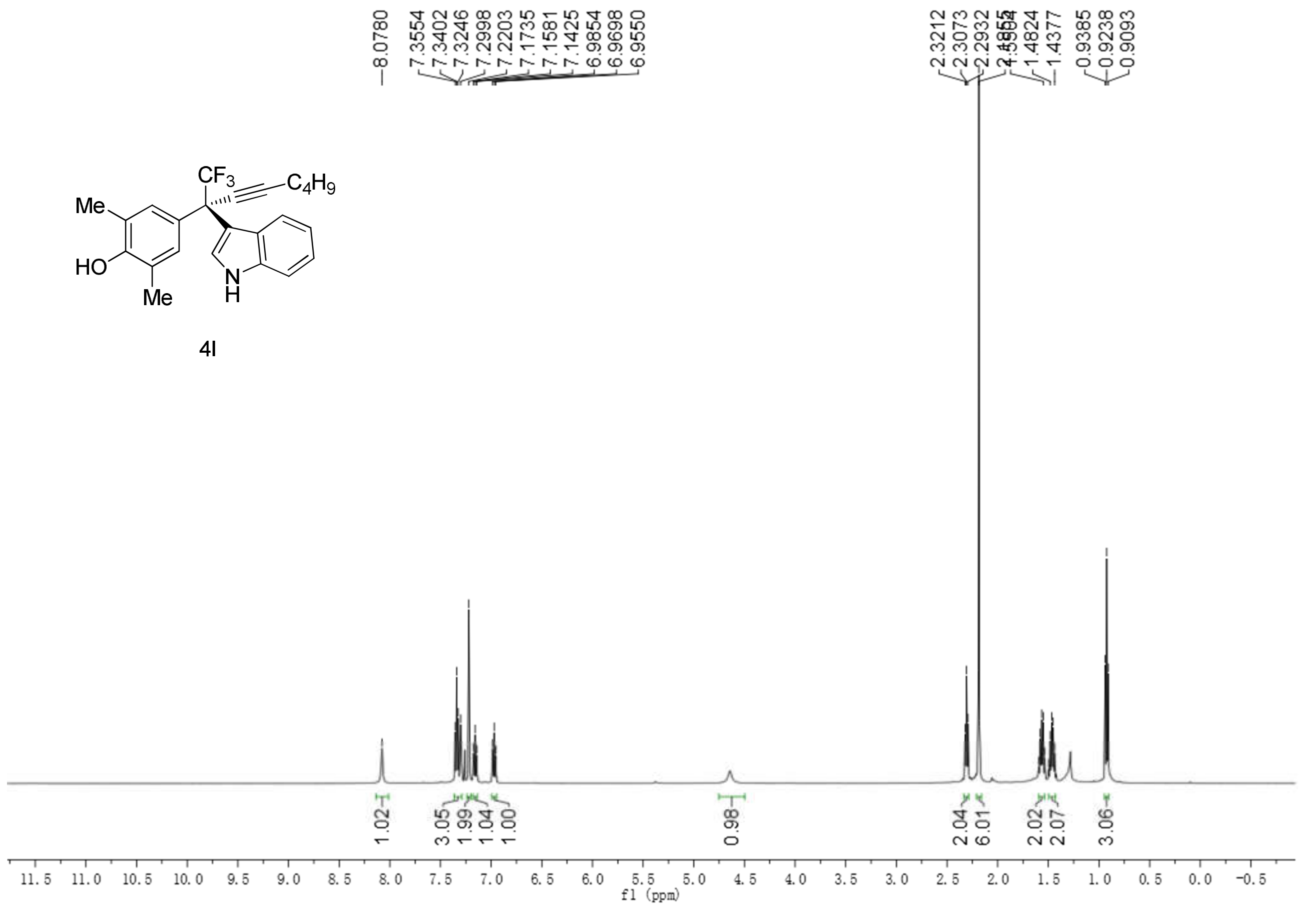
8.0780
7.3554
7.3402
7.3246
7.2998
7.2203
7.1735
7.1581
7.1425
6.9854
6.9698
6.9550
2.3212
2.3073
2.2932
1.4824
1.4377
0.9385
0.9238
0.9093
Me
HO
Me
CF3
C4H9
H
4l
1.02
3.05
1.99
1.04
1.00
0.98
2.04
6.01
2.02
2.07
3.06
f1 (ppm)

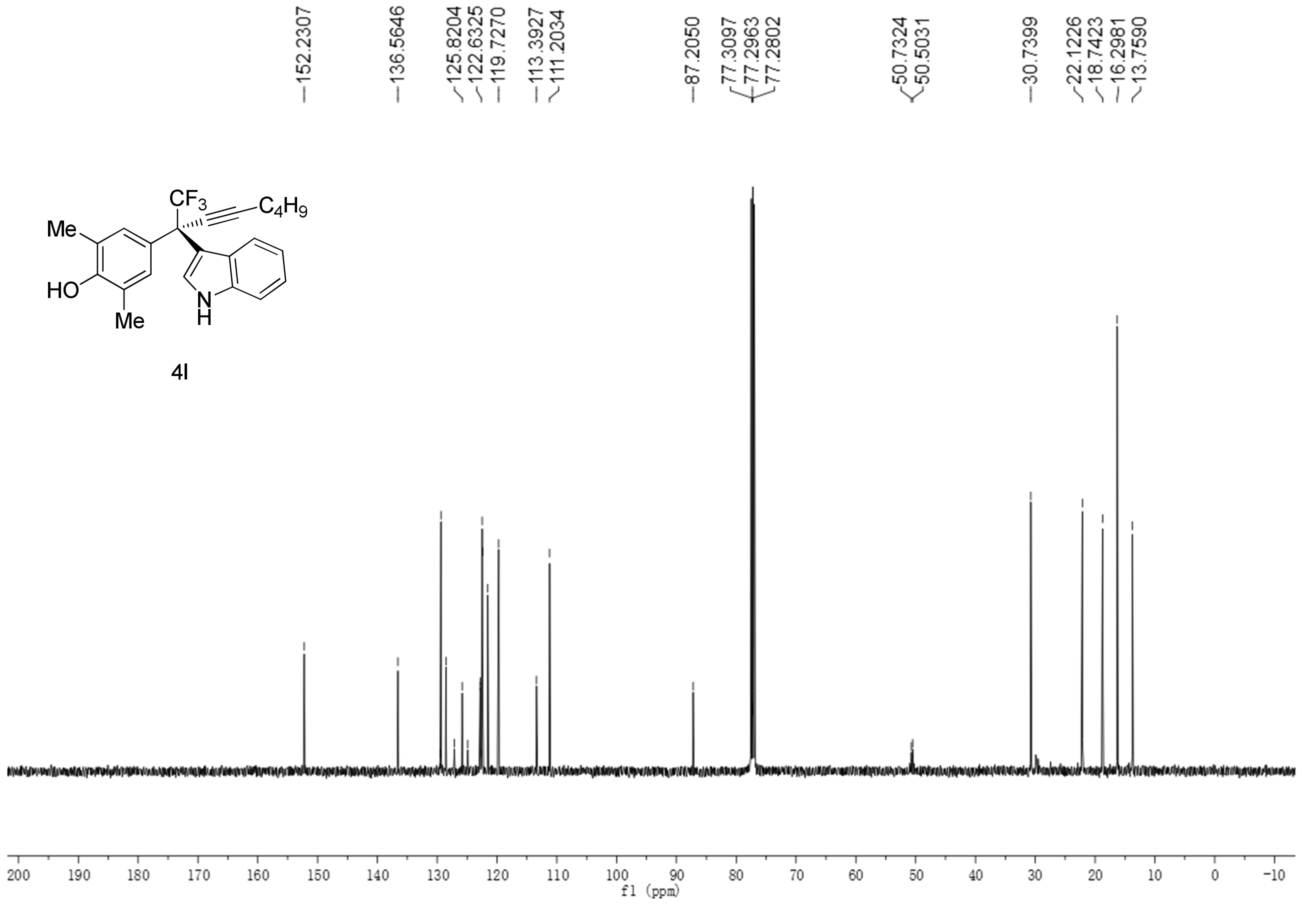
152.2307
136.5646
125.8204
122.6325
119.7270
113.3927
111.2034
87.2050
77.3097
77.2963
77.2802
50.7324
50.5031
30.7399
22.1226
18.7423
16.2981
13.7590
Me
HO
Me
CF3
C4H9
H
4l
f1 (ppm)

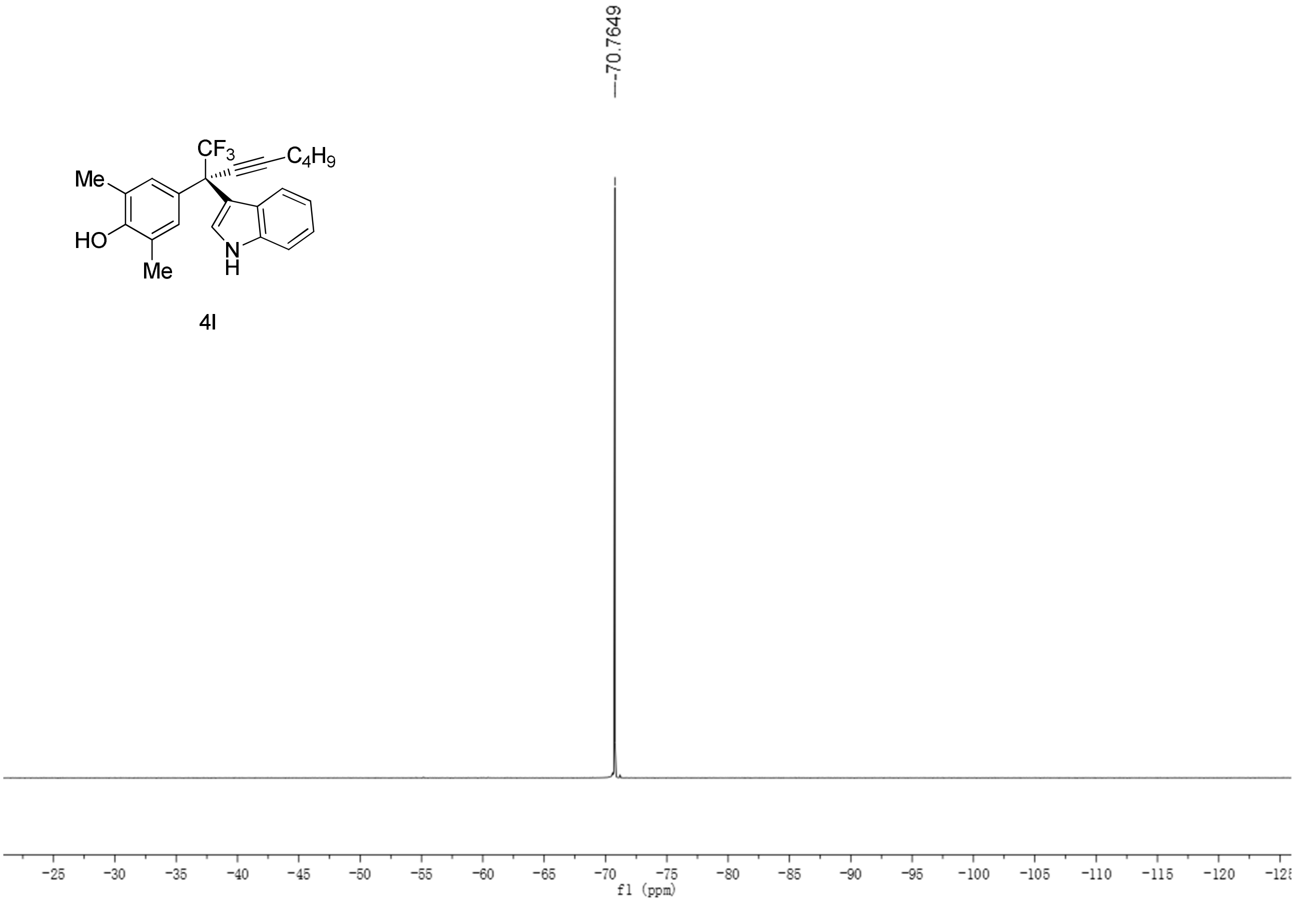

-70.7649
CF_3
C_4H_9
Me
HO
Me
N
H
4l
-25 -30 -35 -40 -45 -50 -55 -60 -65 -70 -75 -80 -85 -90 -95 -100 -105 -110 -115 -120
f1 (ppm)

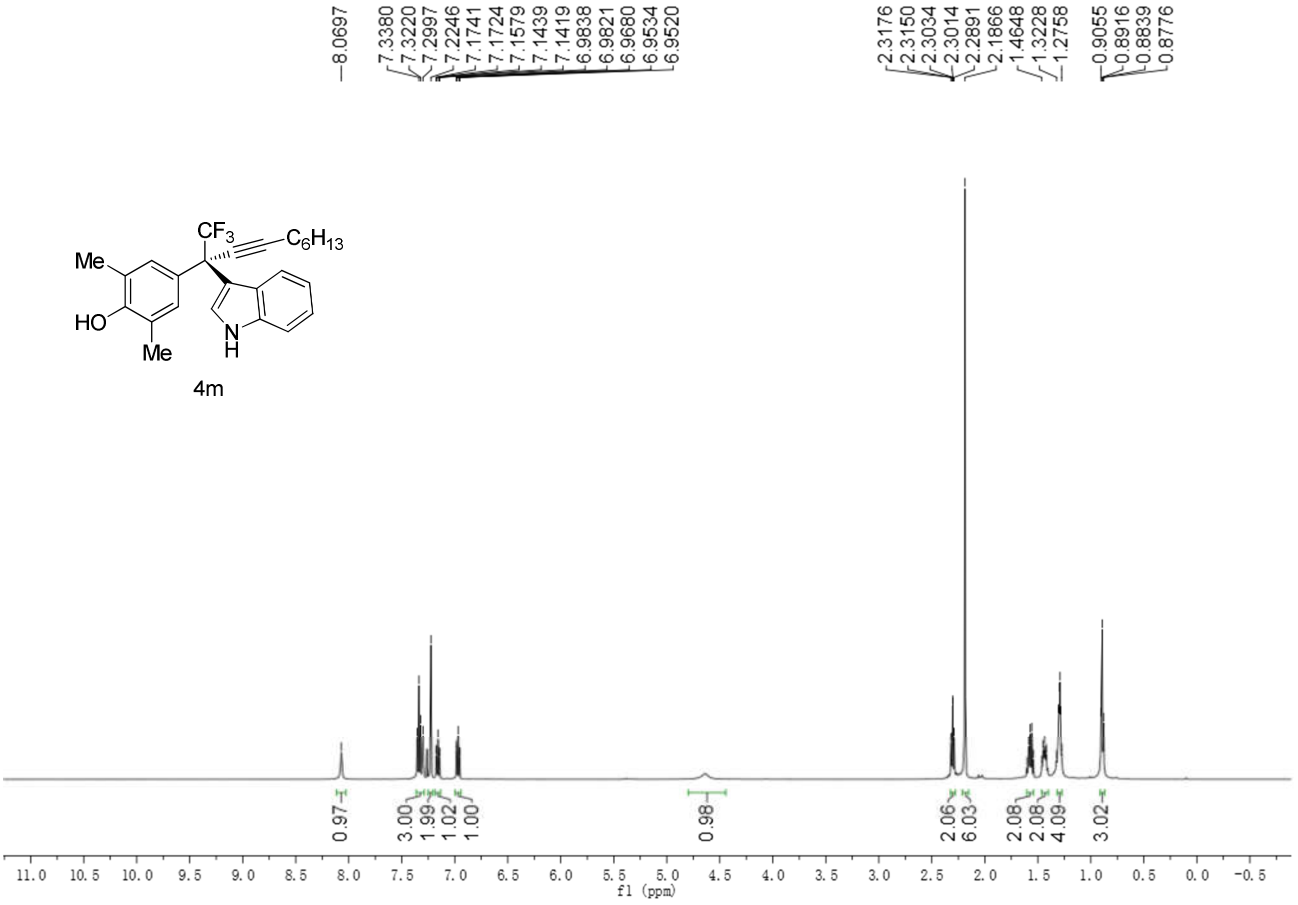

8.0697
7.3380
7.3220
7.2997
7.2246
7.1741
7.1724
7.1579
7.1439
7.1419
6.9838
6.9821
6.9680
6.9534
6.9520
2.3176
2.3150
2.3034
2.3014
2.2891
2.1866
1.4648
1.3228
1.2758
0.9055
0.8916
0.8839
0.8776
CF_3
C_6H_{13}
Me
HO
Me
N
H
4m
0.97
3.00
1.99
1.02
1.00
0.98
2.06
6.03
2.08
2.08
4.09
3.02
11.0 10.5 10.0 9.5 9.0 8.5 8.0 7.5 7.0 6.5 6.0 5.5 5.0 4.5 4.0 3.5 3.0 2.5 2.0 1.5 1.0 0.5 0.0 -0.5
f1 (ppm)

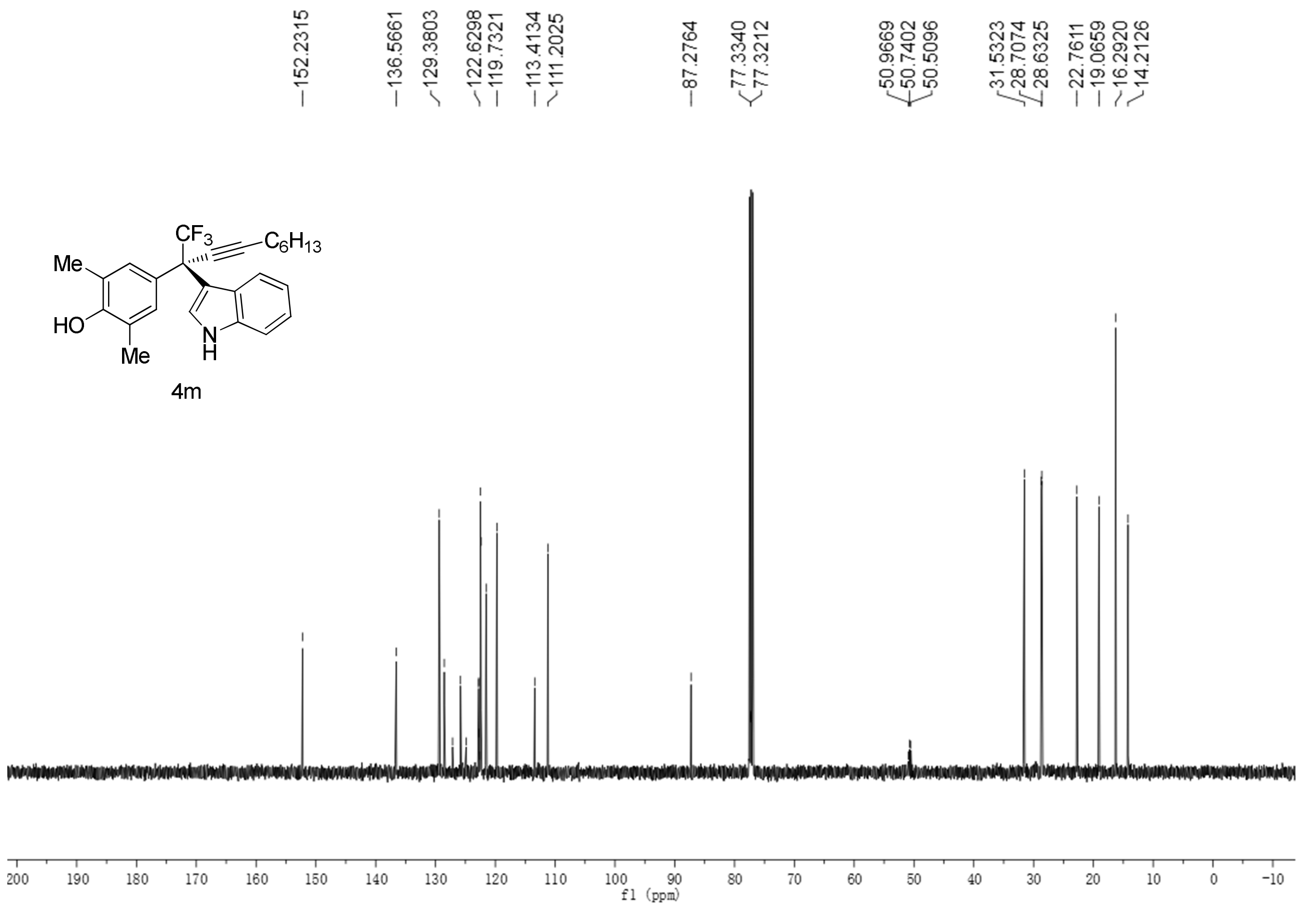

152.2315
136.5661
129.3803
122.6298
119.7321
113.4134
111.2025
87.2764
77.3340
77.3212
50.9669
50.7402
50.5096
31.5323
28.7074
28.6325
22.7611
19.0659
16.2920
14.2126
Me
HO
Me
CF3
C6H13
N
H
4m
f1 (ppm)

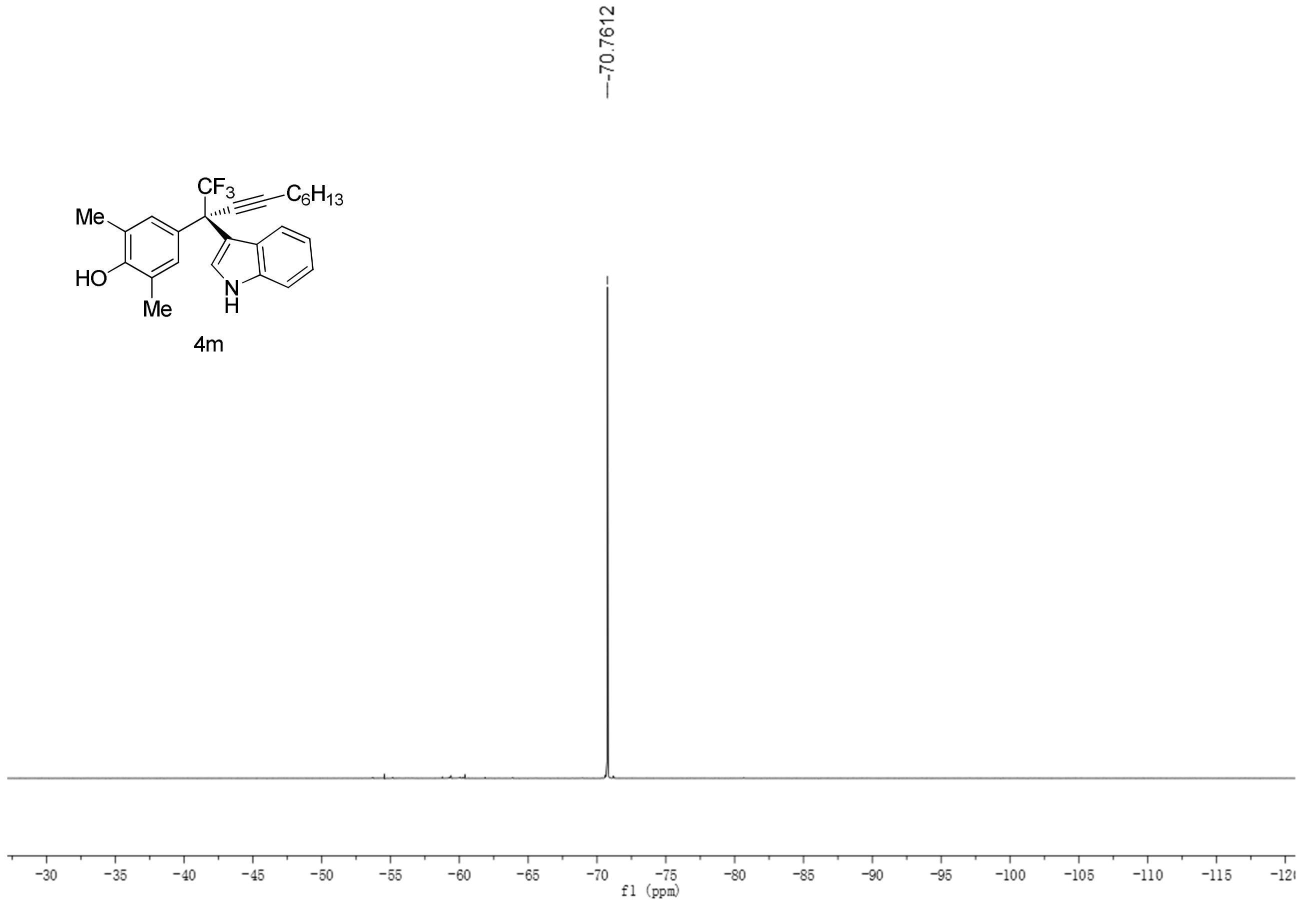

-70.7612
Me
HO
Me
CF3
C6H13
N
H
4m
f1 (ppm)

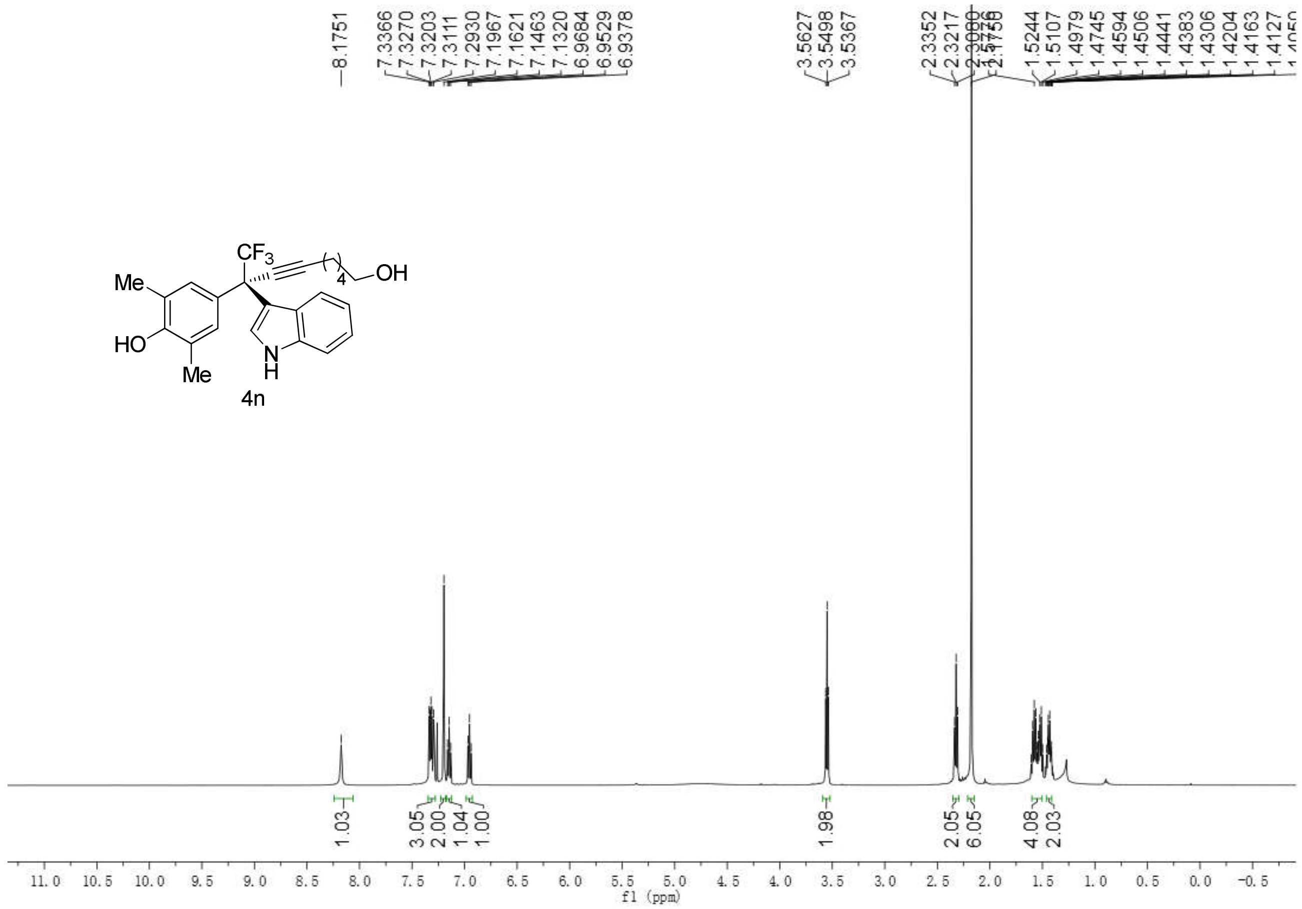
8.1751
7.3366
7.3270
7.3203
7.3111
7.2930
7.1967
7.1621
7.1463
7.1320
6.9684
6.9529
6.9378
3.5627
3.5498
3.5367
2.3352
2.3217
1.5244
1.5107
1.4979
1.4745
1.4594
1.4506
1.4441
1.4383
1.4306
1.4204
1.4163
1.4127
CF3
Me
HO
Me
OH
4
N
H
4n
1.03
3.05
2.00
1.04
1.00
1.98
2.05
6.05
4.08
2.03
f1 (ppm)

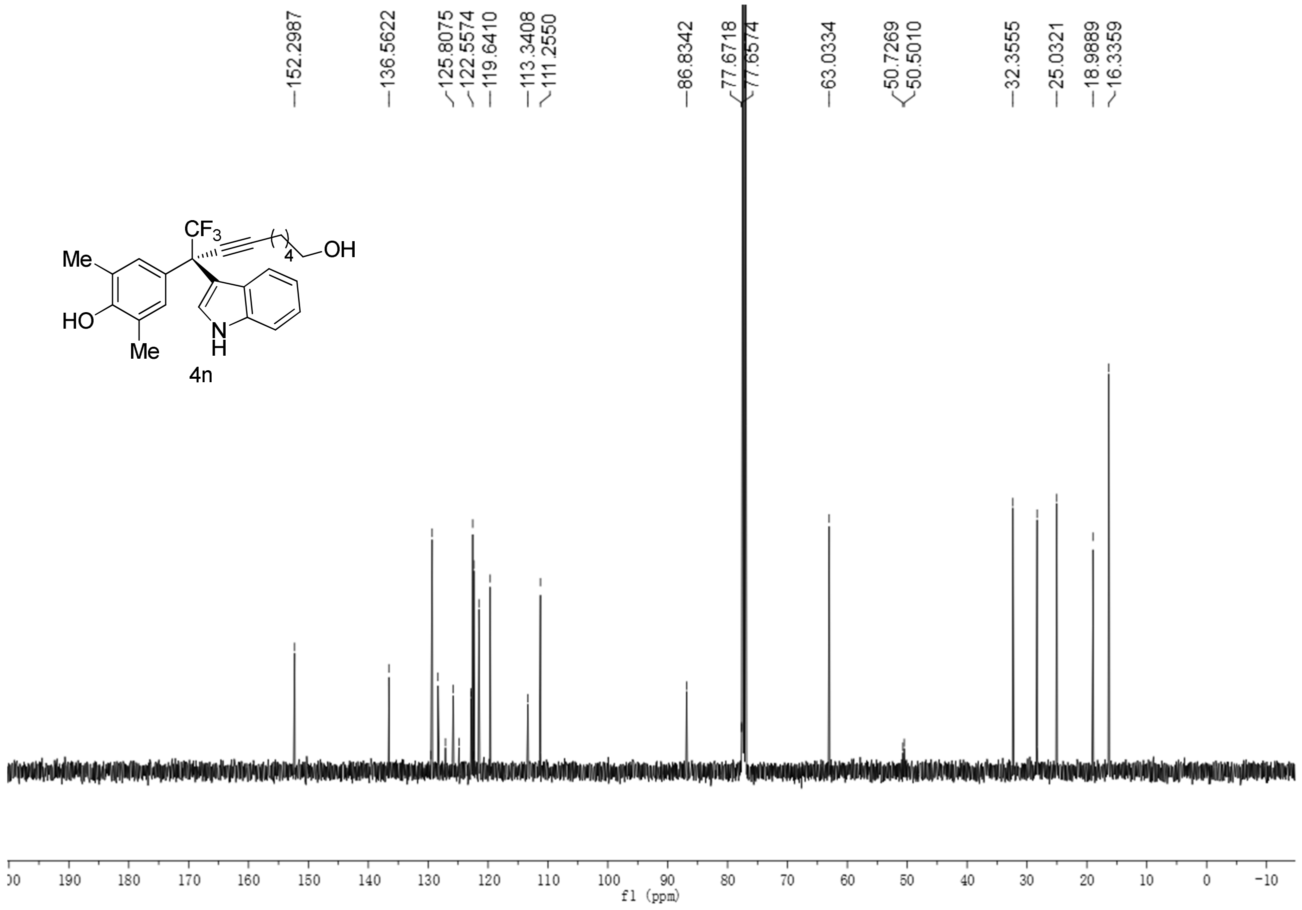
152.2987
136.5622
125.8075
122.5574
119.6410
113.3408
111.2550
86.8342
77.6718
77.6574
63.0334
50.7269
50.5010
32.3555
25.0321
18.9889
16.3359
CF3
Me
HO
Me
OH
4
N
H
4n
f1 (ppm)

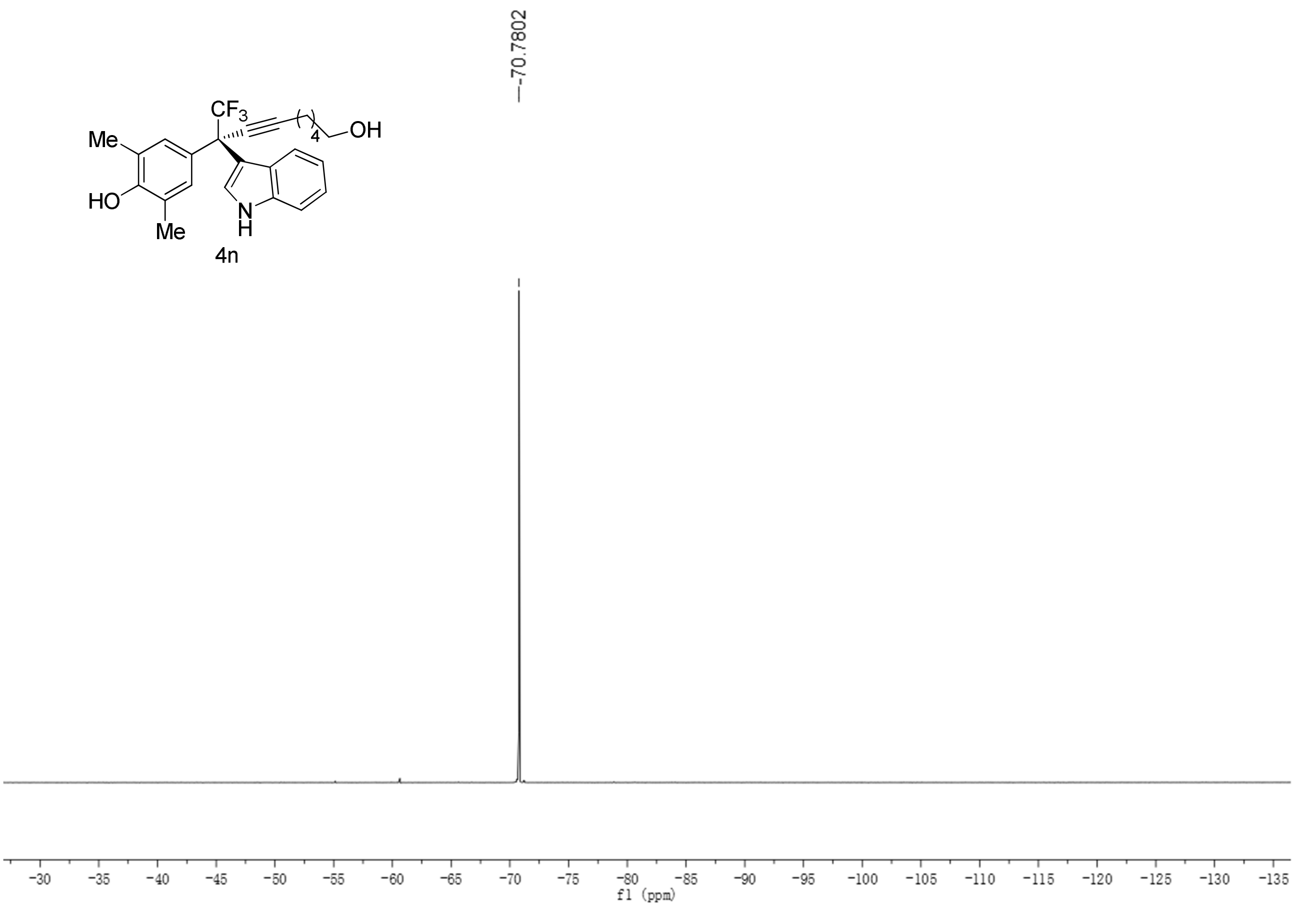

-70.7802
CF3
Me
HO
Me
N
H
OH
4
4n
-30 -35 -40 -45 -50 -55 -60 -65 -70 -75 -80 -85 -90 -95 -100 -105 -110 -115 -120 -125 -130 -135
f1 (ppm)

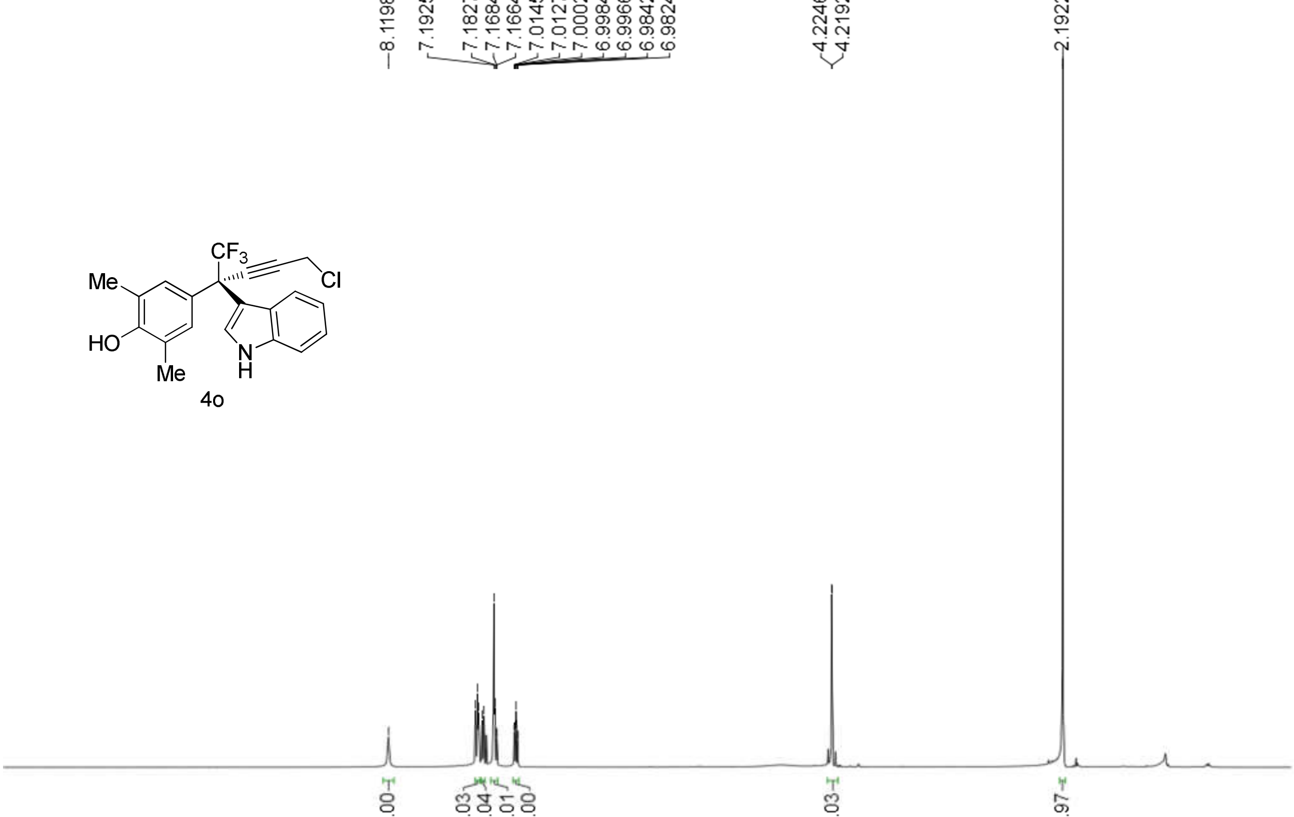

8.1198
7.1925
7.1827
7.1684
7.1664
7.0145
7.0127
7.0002
6.9984
6.9966
6.9842
6.9824
4.2246
4.2192
2.1922
CF3
Cl
Me
HO
Me
N
H
4o
1.00
2.03
1.04
3.01
1.00
2.03
5.97
.5 11.0 10.5 10.0 9.5 9.0 8.5 8.0 7.5 7.0 6.5 6.0 5.5 5.0 4.5 4.0 3.5 3.0 2.5 2.0 1.5 1.0 0.5
f1 (ppm)

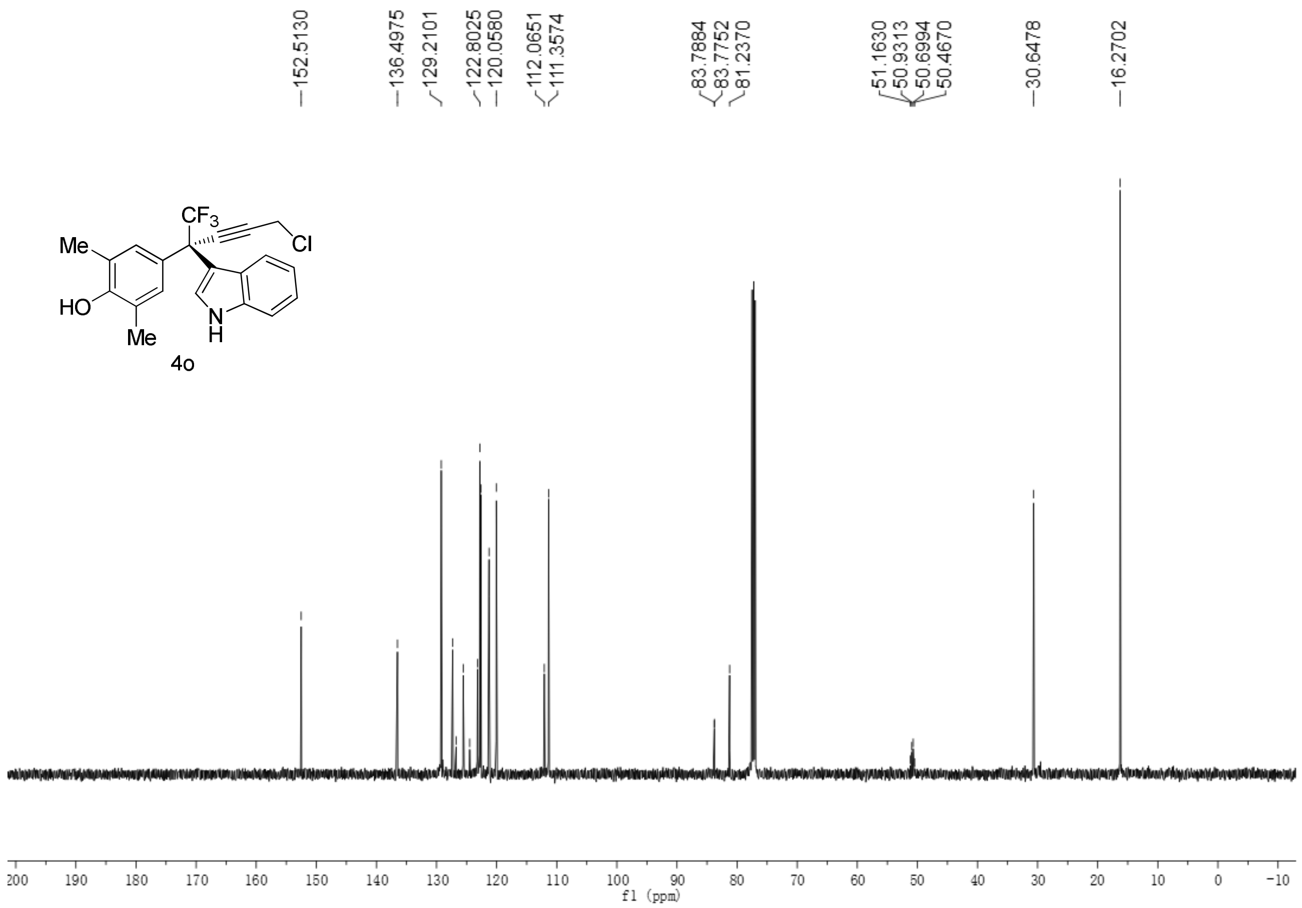
152.5130
136.4975
129.2101
122.8025
120.0580
112.0651
111.3574
83.7884
83.7752
81.2370
51.1630
50.9313
50.6994
50.4670
30.6478
16.2702
Me
HO
Me
CF3
Cl
N
H
4o
f1 (ppm)

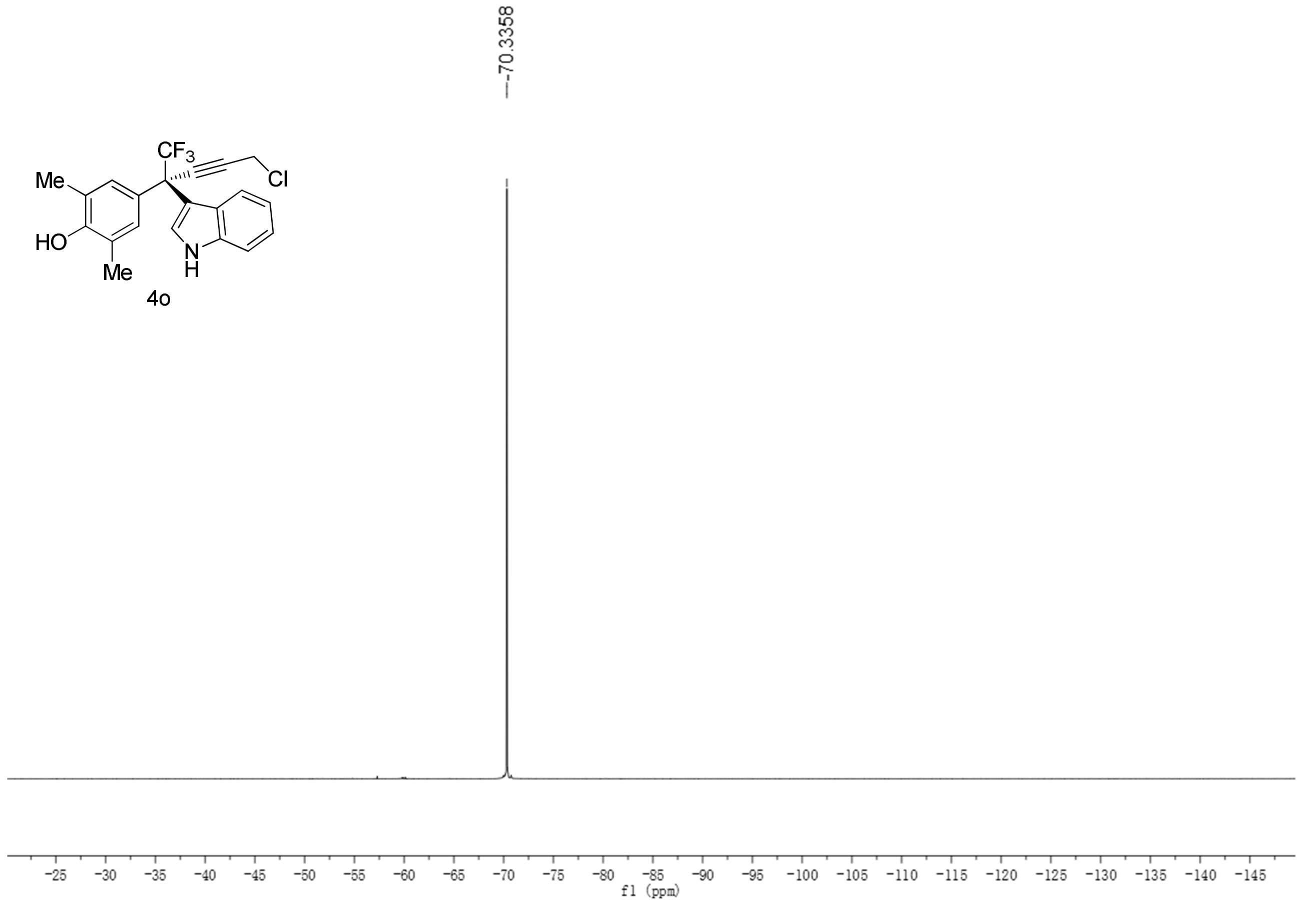
-70.3358
Me
HO
Me
CF3
Cl
N
H
4o
f1 (ppm)

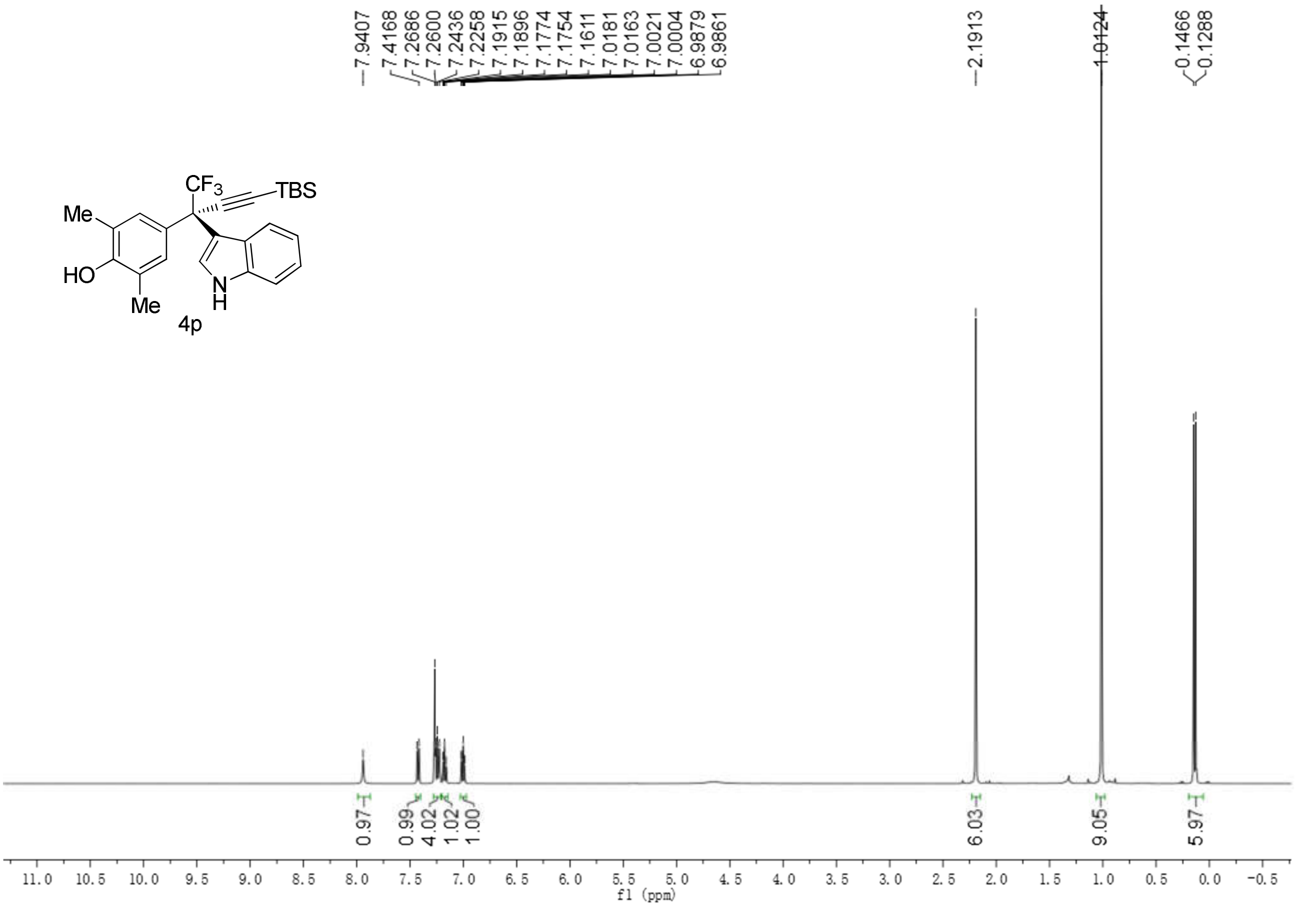

7.9407
7.4168
7.2686
7.2600
7.2436
7.2258
7.1915
7.1896
7.1774
7.1754
7.1611
7.0181
7.0163
7.0021
7.0004
6.9879
6.9861
2.1913
1.0124
0.1466
0.1288
CF3
TBS
Me
HO
Me
N
H
4p
0.97
0.99
4.02
1.02
1.00
6.03
9.05
5.97
f1 (ppm)

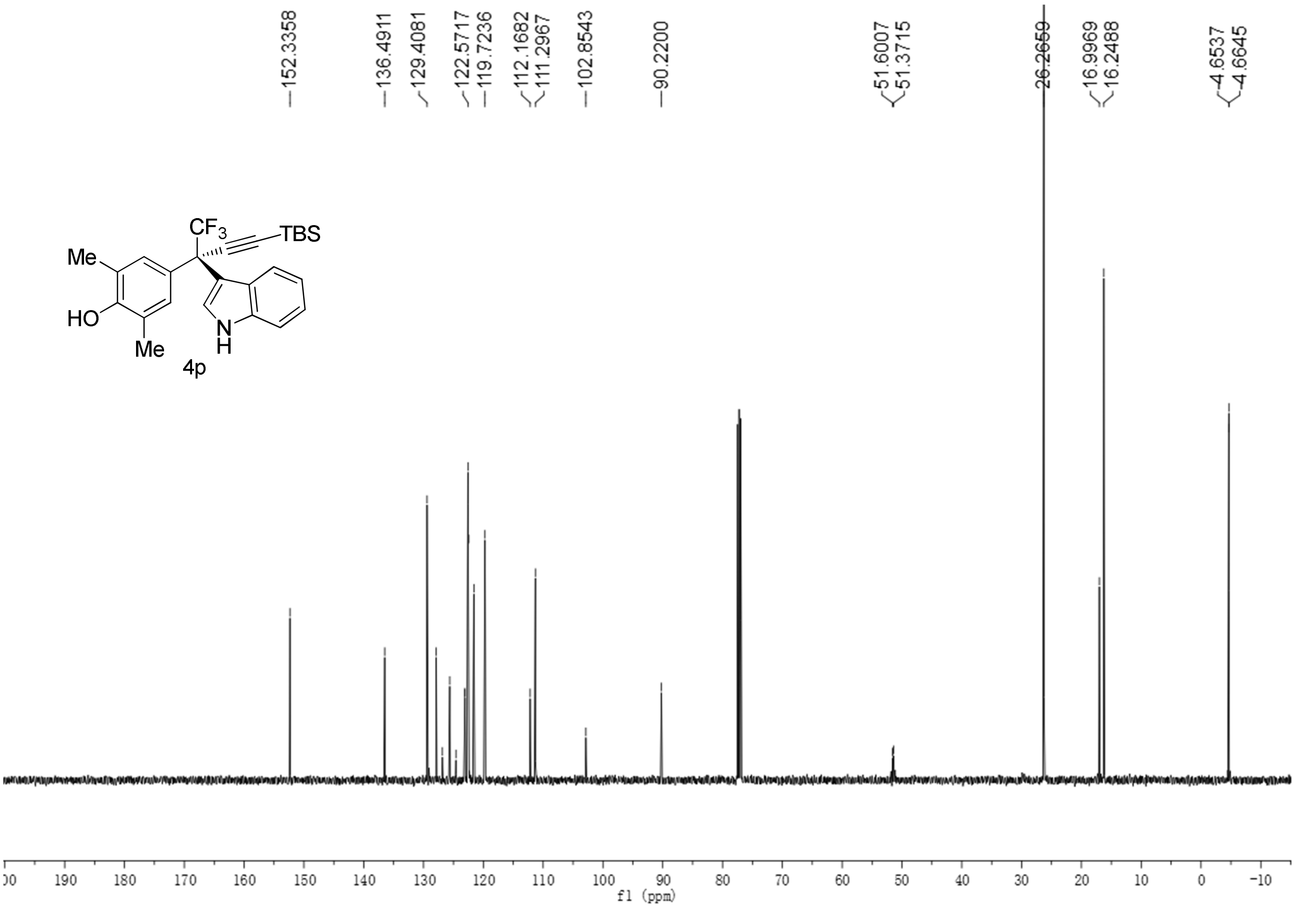

152.3358
136.4911
129.4081
122.5717
119.7236
112.1682
111.2967
102.8543
90.2200
51.6007
51.3715
26.2659
16.9969
16.2488
-4.6537
-4.6645
CF3
TBS
Me
HO
Me
N
H
4p
f1 (ppm)

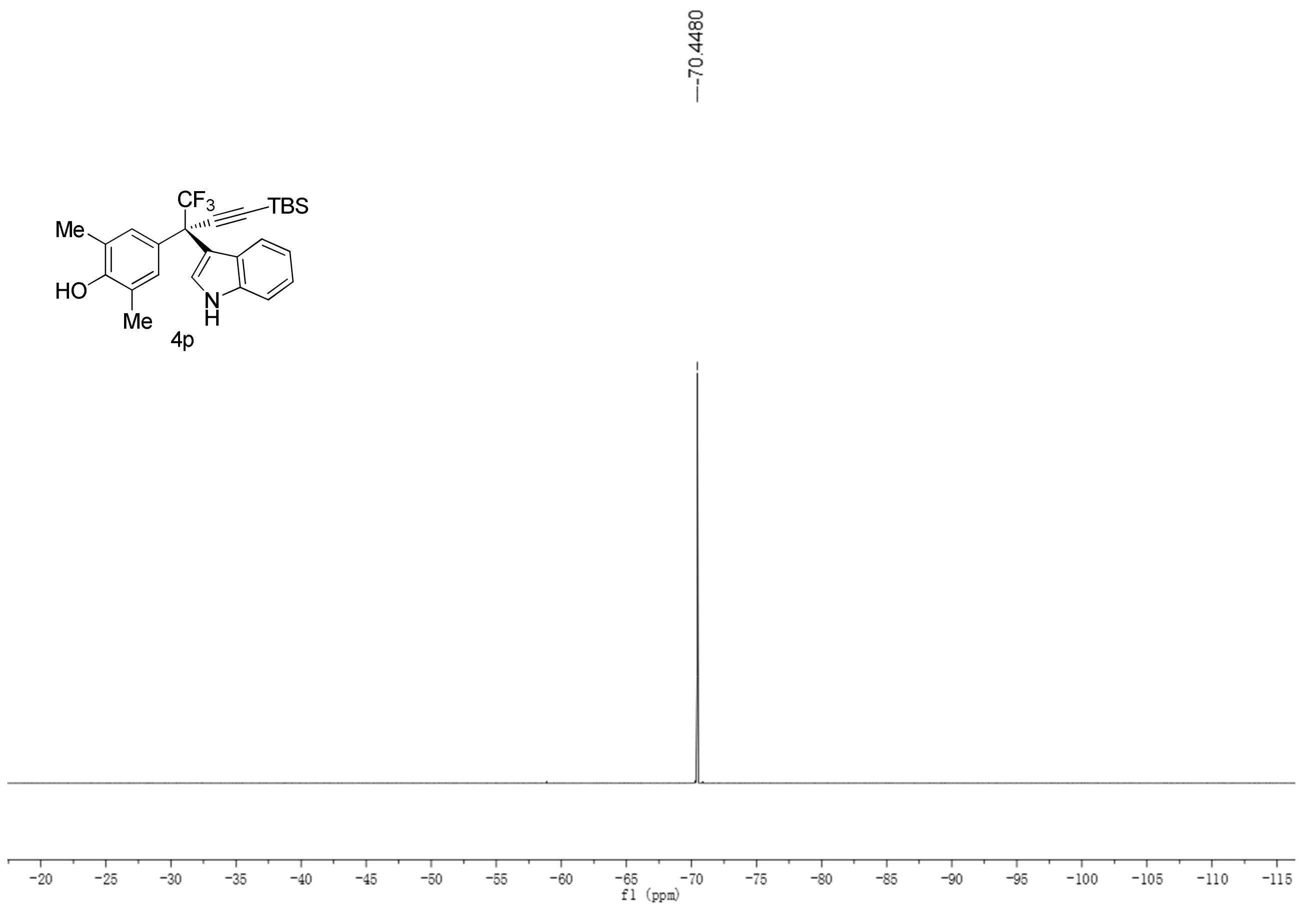

-70.4480
CF3
TBS
Me
HO
Me
N
H
4p
f1 (ppm)

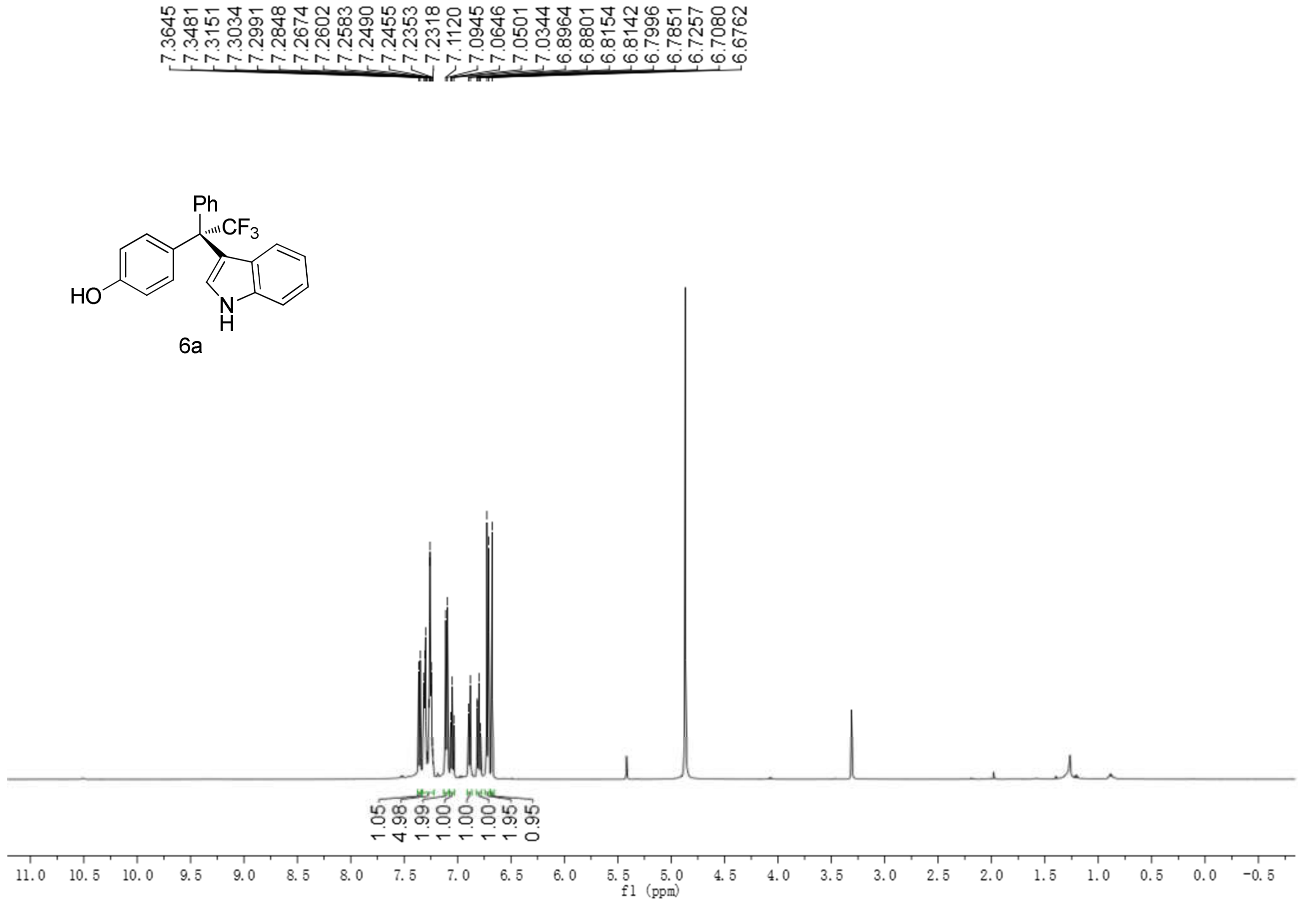

7.3645
7.3481
7.3151
7.3034
7.2991
7.2848
7.2674
7.2602
7.2583
7.2490
7.2455
7.2353
7.2318
7.1120
7.0945
7.0646
7.0501
7.0344
6.8964
6.8801
6.8154
6.8142
6.7996
6.7851
6.7257
6.7080
6.6762
Ph
CF3
HO
N
H
6a
1.05
4.98
1.99
1.00
1.00
1.00
1.95
0.95
f1 (ppm)

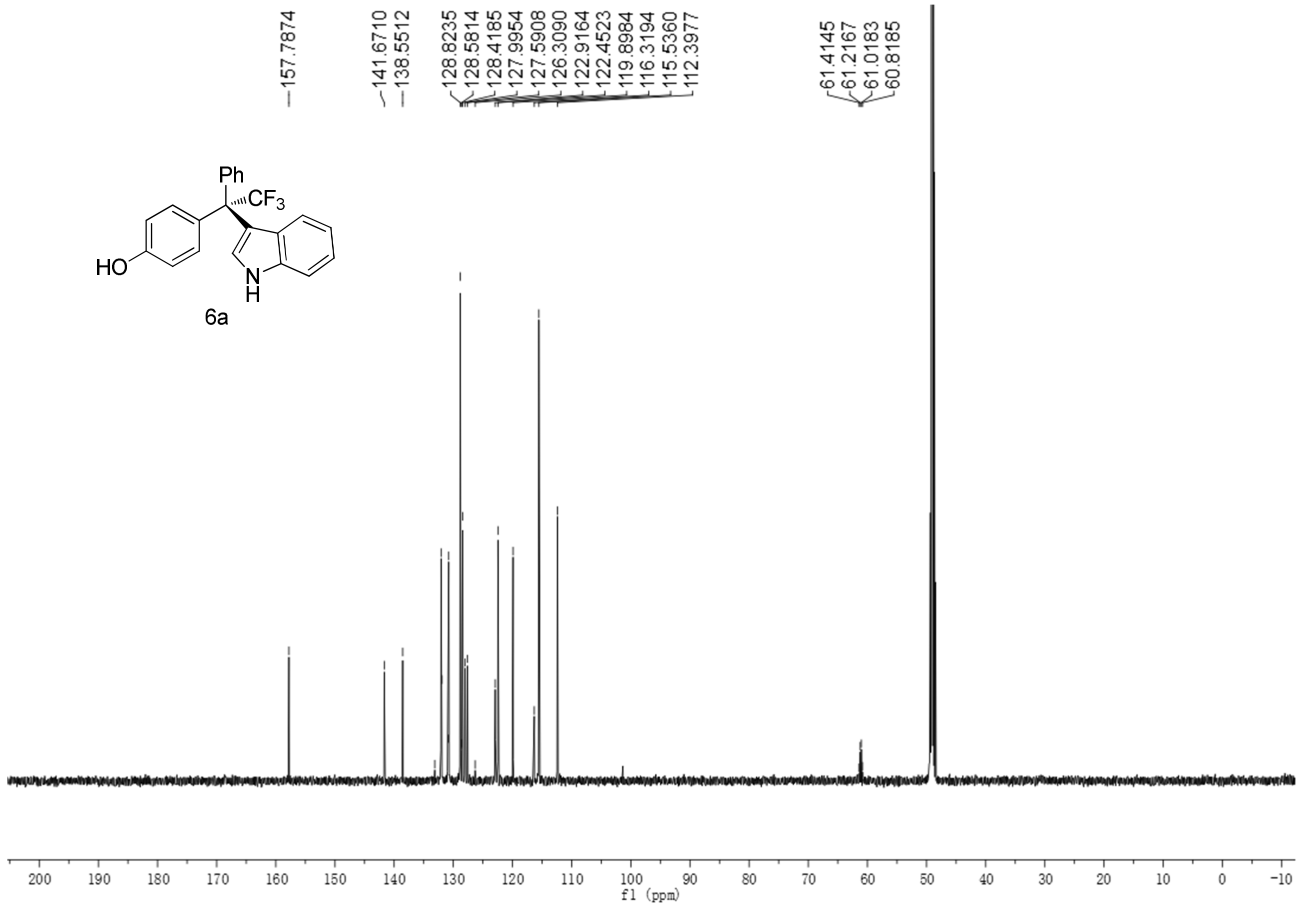
157.7874
141.6710
138.5512
128.8235
128.5814
128.4185
127.9954
127.5908
126.3090
122.9164
122.4523
119.8984
116.3194
115.5360
112.3977
61.4145
61.2167
61.0183
60.8185
Ph
CF3
HO
N
H
6a
200
190
180
170
160
150
140
130
120
110
100
90
80
70
60
50
40
30
20
10
0
-10
f1 (ppm)

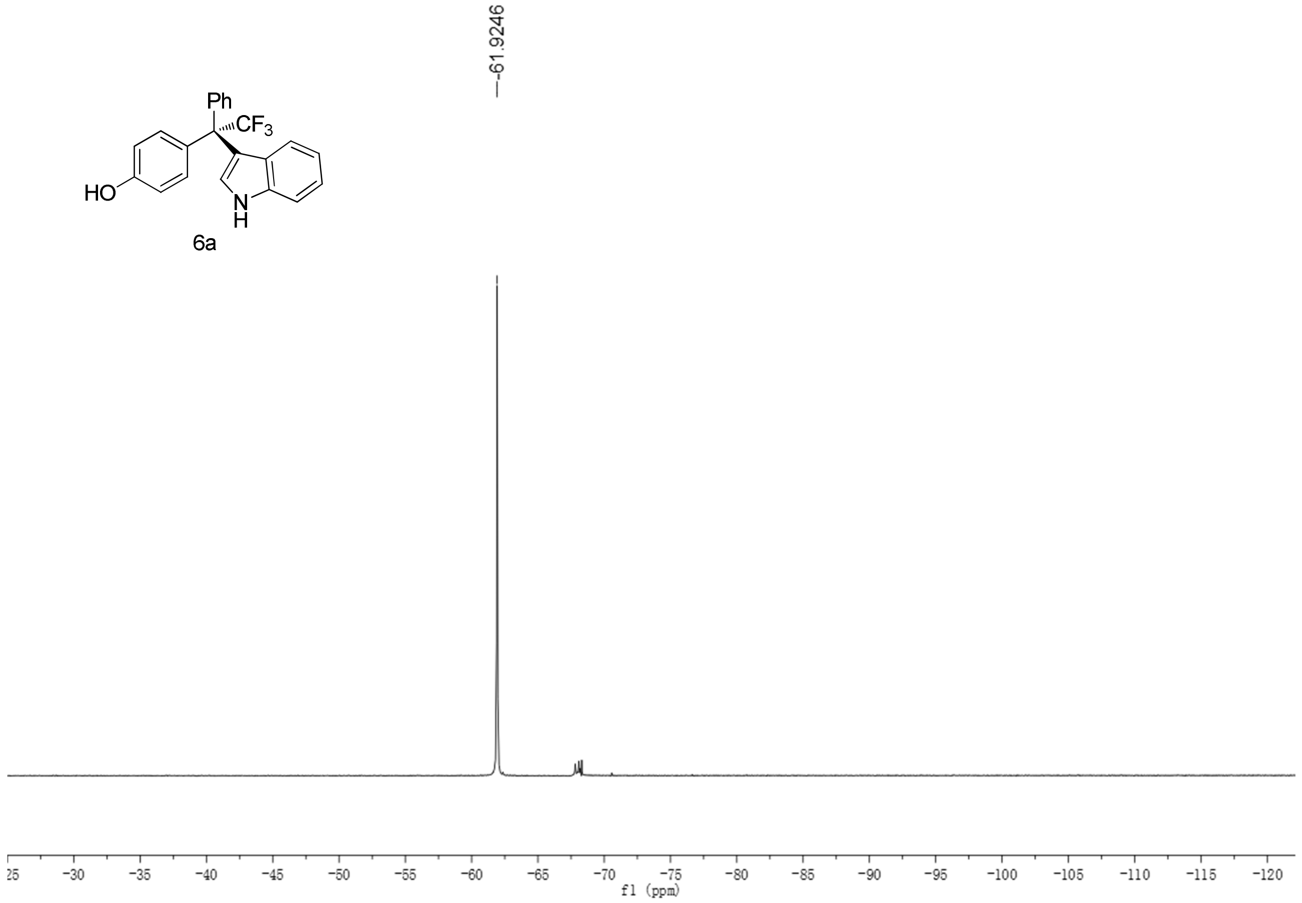
-61.9246
Ph
CF3
HO
N
H
6a
-30
-35
-40
-45
-50
-55
-60
-65
-70
-75
-80
-85
-90
-95
-100
-105
-110
-115
-120
f1 (ppm)

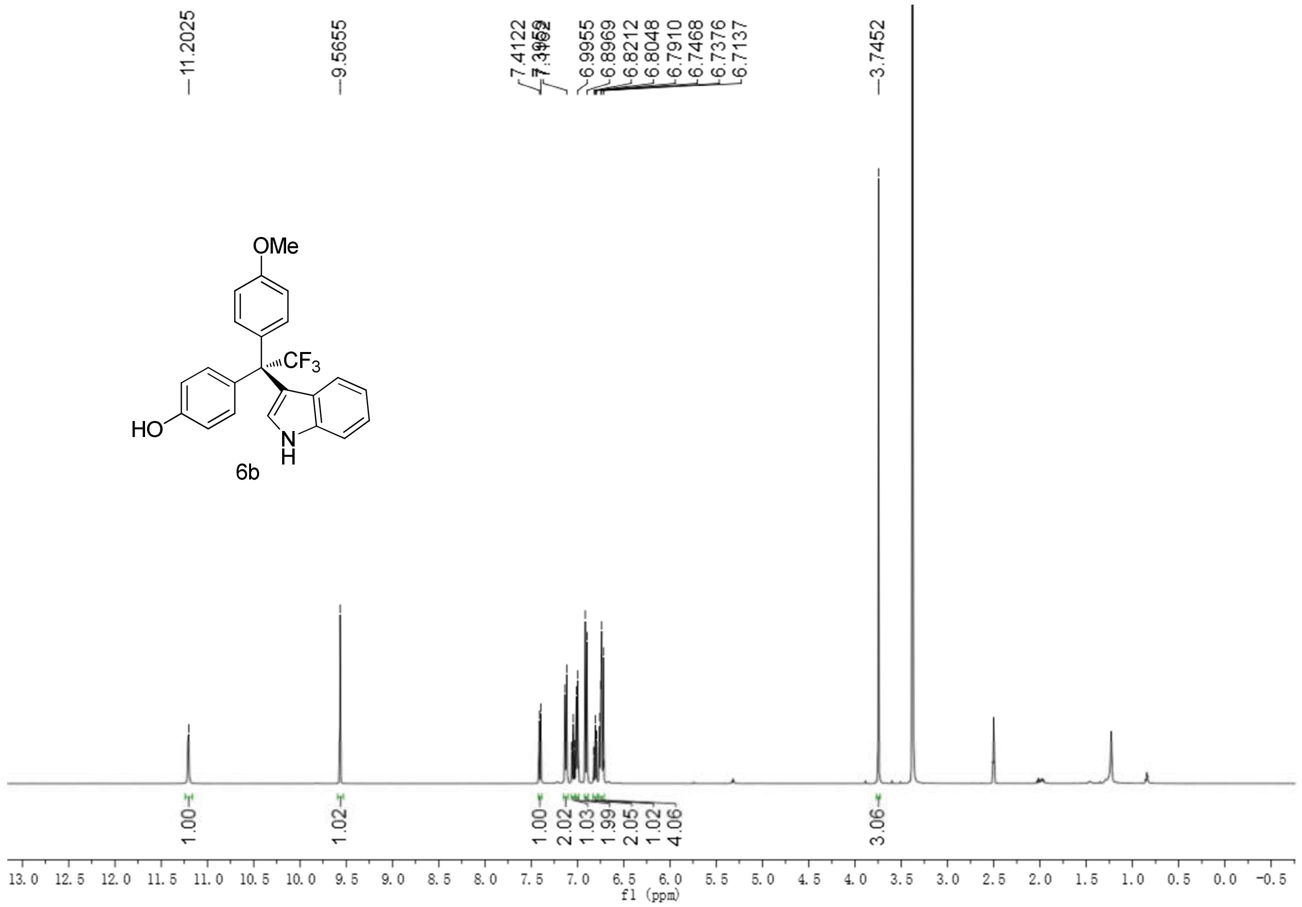
11.2025
9.5655
7.4122
6.9955
6.8969
6.8212
6.8048
6.7910
6.7468
6.7376
6.7137
3.7452
OMe
CF_3
HO
N
H
6b
1.00
1.02
1.00
2.02
1.03
1.99
2.05
1.02
4.06
3.06
f1 (ppm)

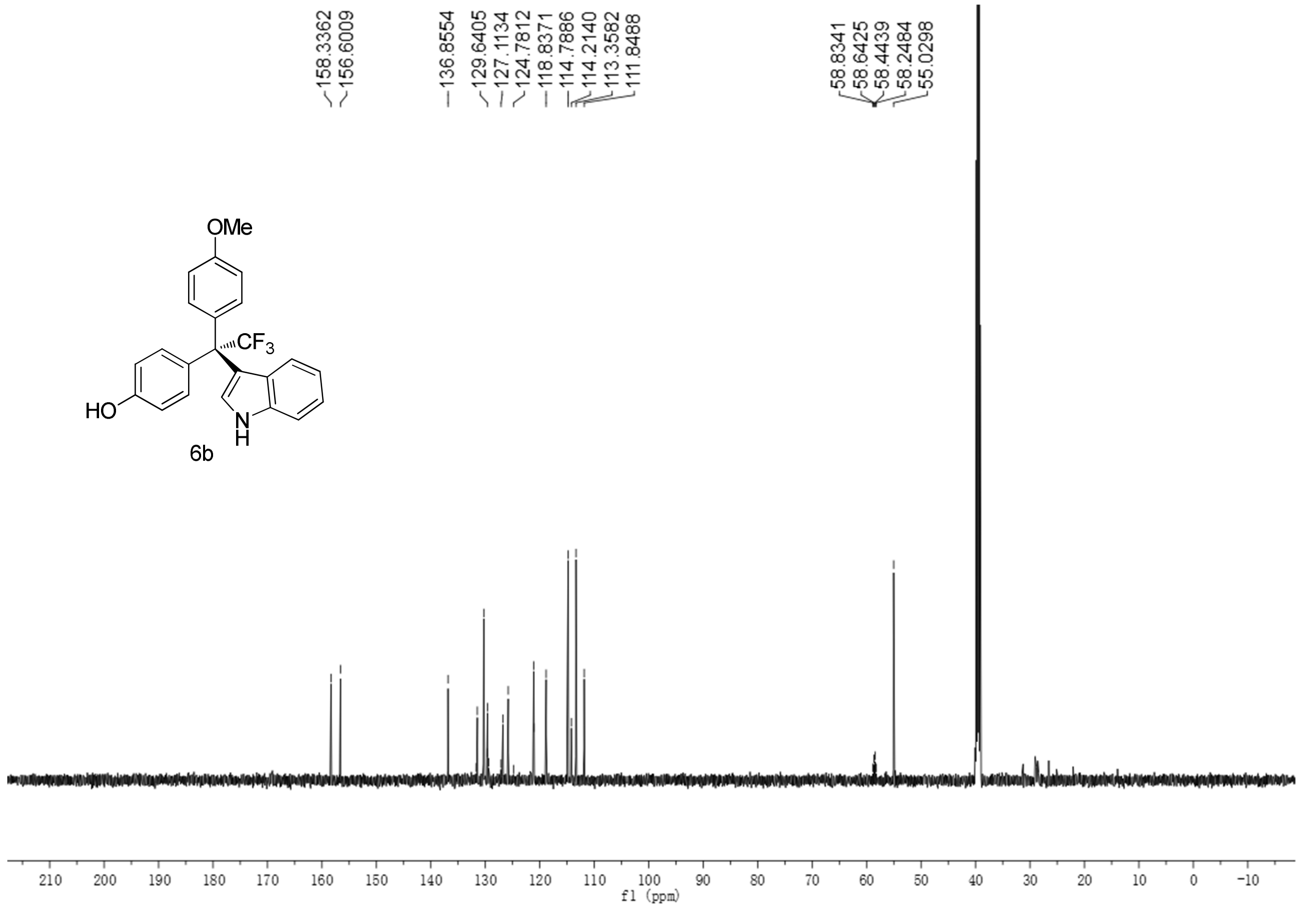
158.3362
156.6009
136.8554
129.6405
127.1134
124.7812
118.8371
114.7886
114.2140
113.3582
111.8488
58.8341
58.6425
58.4439
58.2484
55.0298
OMe
CF_3
HO
N
H
6b
f1 (ppm)

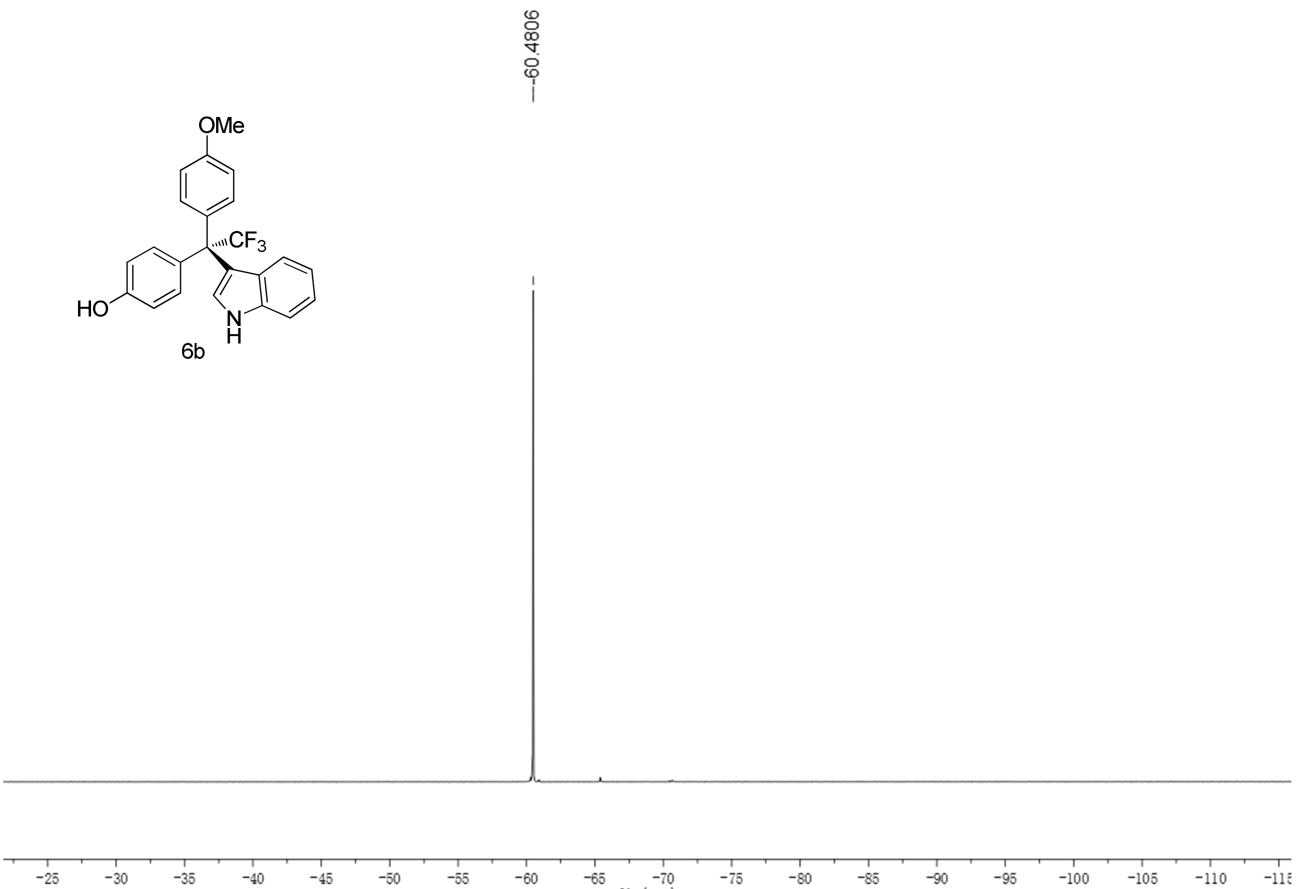
-60.4806
OMe
CF3
HO
N
H
6b
-25 -30 -35 -40 -45 -50 -55 -60 -65 -70 -75 -80 -85 -90 -95 -100 -105 -110
f1 (ppm)

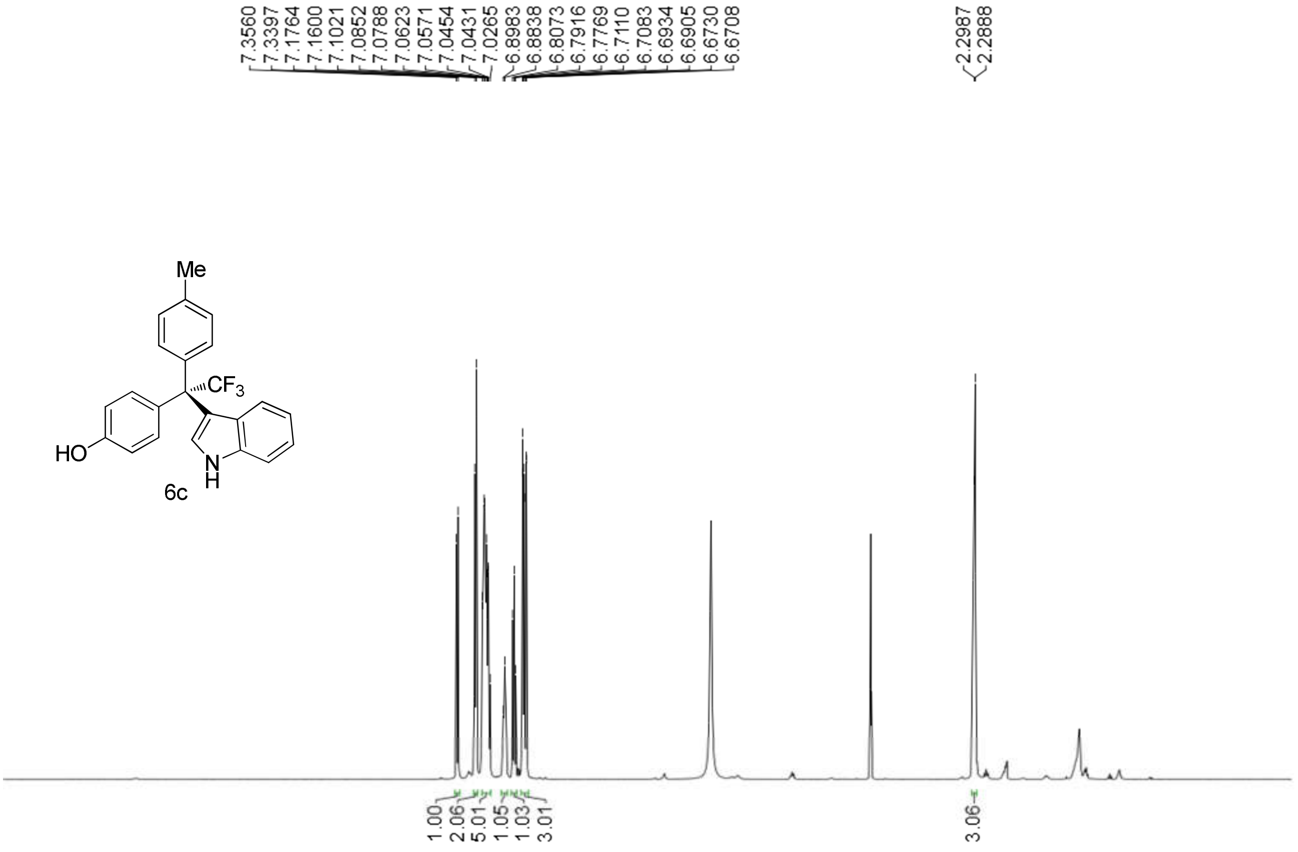
7.3560
7.3397
7.1764
7.1600
7.1021
7.0852
7.0788
7.0623
7.0571
7.0454
7.0431
7.0265
6.8983
6.8838
6.8073
6.7916
6.7769
6.7110
6.7083
6.6934
6.6905
6.6730
6.6708
2.2987
2.2888
Me
CF3
HO
N
H
6c
1.00
2.06
5.01
1.05
1.03
3.01
3.06
11.5 11.0 10.5 10.0 9.5 9.0 8.5 8.0 7.5 7.0 6.5 6.0 5.5 5.0 4.5 4.0 3.5 3.0 2.5 2.0 1.5 1.0 0.5 0.0 -0.5
f1 (ppm)

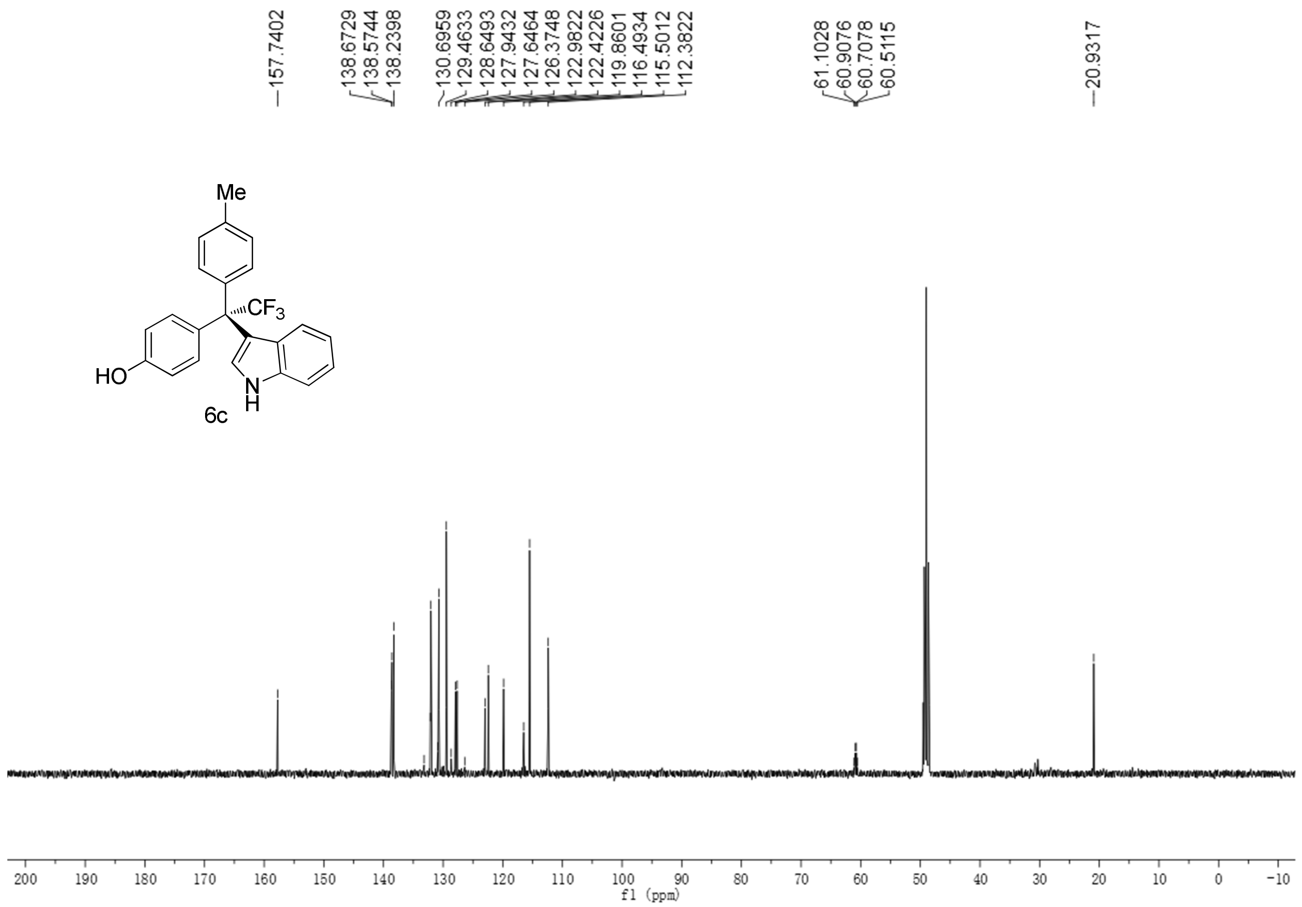
157.7402
138.6729
138.5744
138.2398
130.6959
129.4633
128.6493
127.9432
127.6464
126.3748
122.9822
122.4226
119.8601
116.4934
115.5012
112.3822
61.1028
60.9076
60.7078
60.5115
20.9317
Me
CF3
HO
N
H
6c
f1 (ppm)

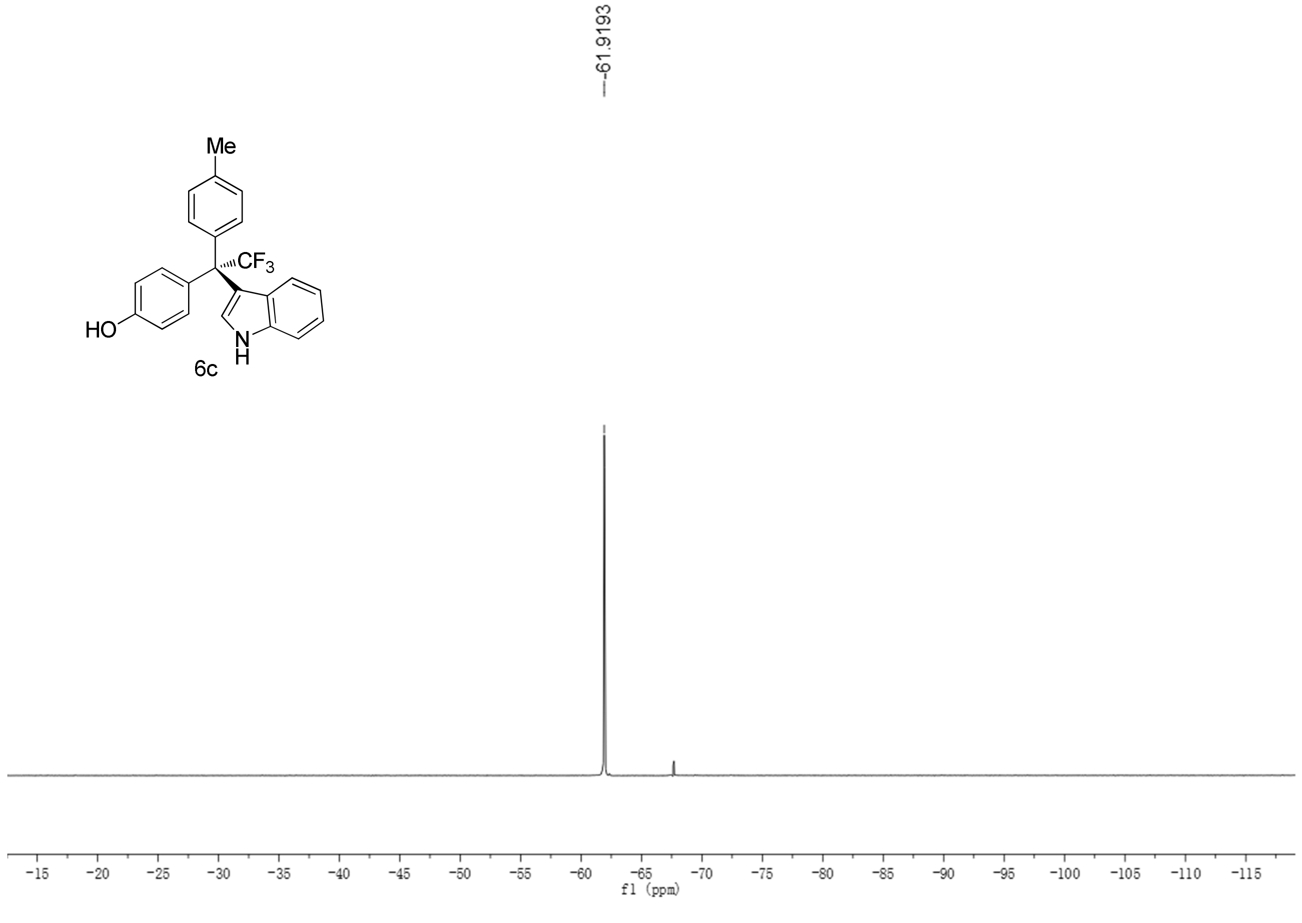
-61.9193
Me
CF3
HO
N
H
6c
f1 (ppm)

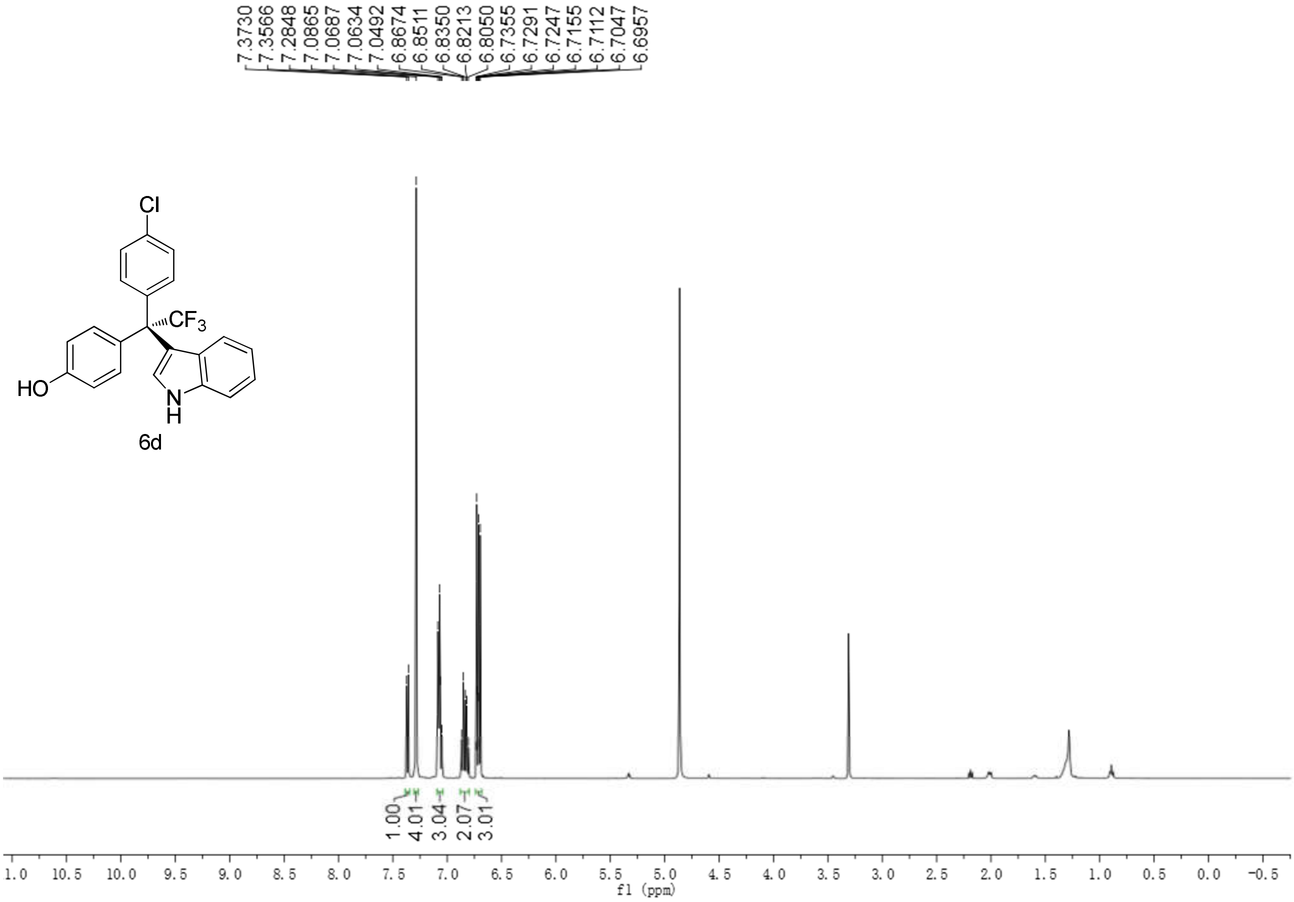
7.3730
7.3566
7.2848
7.0865
7.0687
7.0634
7.0492
6.8674
6.8511
6.8350
6.8213
6.8050
6.7355
6.7291
6.7247
6.7155
6.7112
6.7047
6.6957
Cl
CF3
HO
N
H
1.00
4.01
3.04
2.07
3.01
f1 (ppm)

6d

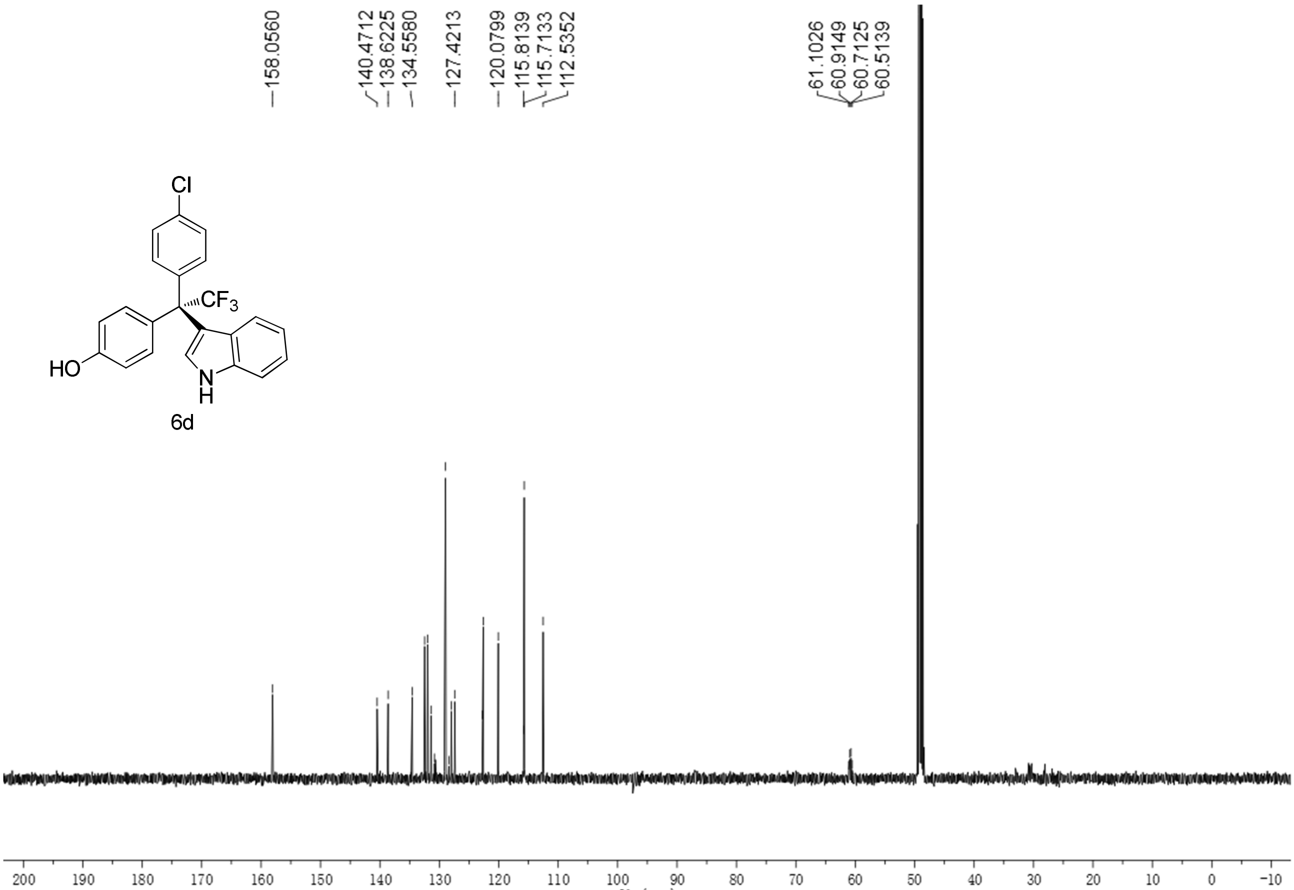
158.0560
140.4712
138.6225
134.5580
127.4213
120.0799
115.8139
115.7133
112.5352
61.1026
60.9149
60.7125
60.5139
Cl
CF3
HO
N
H
f1 (ppm)

6d

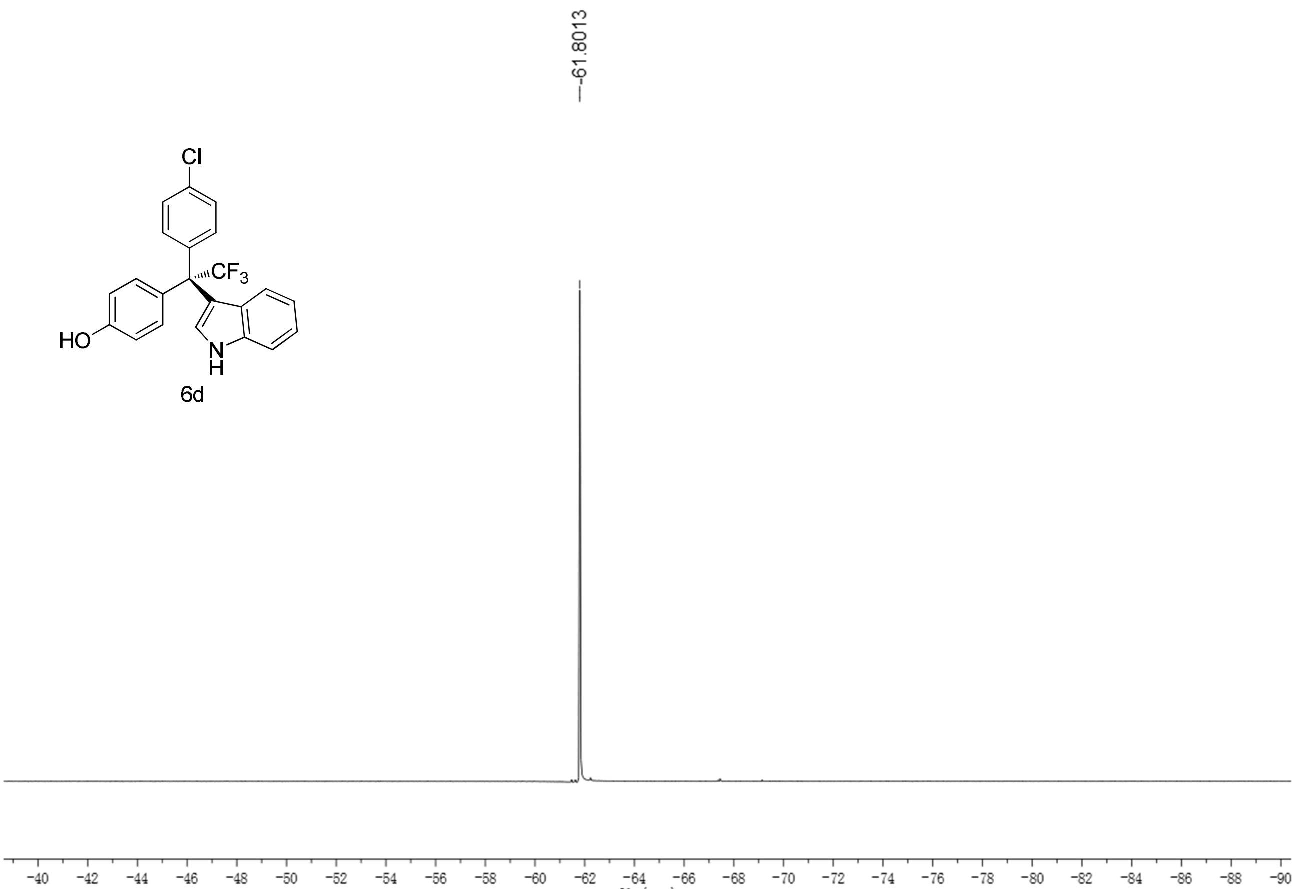

-61.8013
Cl
CF3
HO
N
H
6d
f1 (ppm)

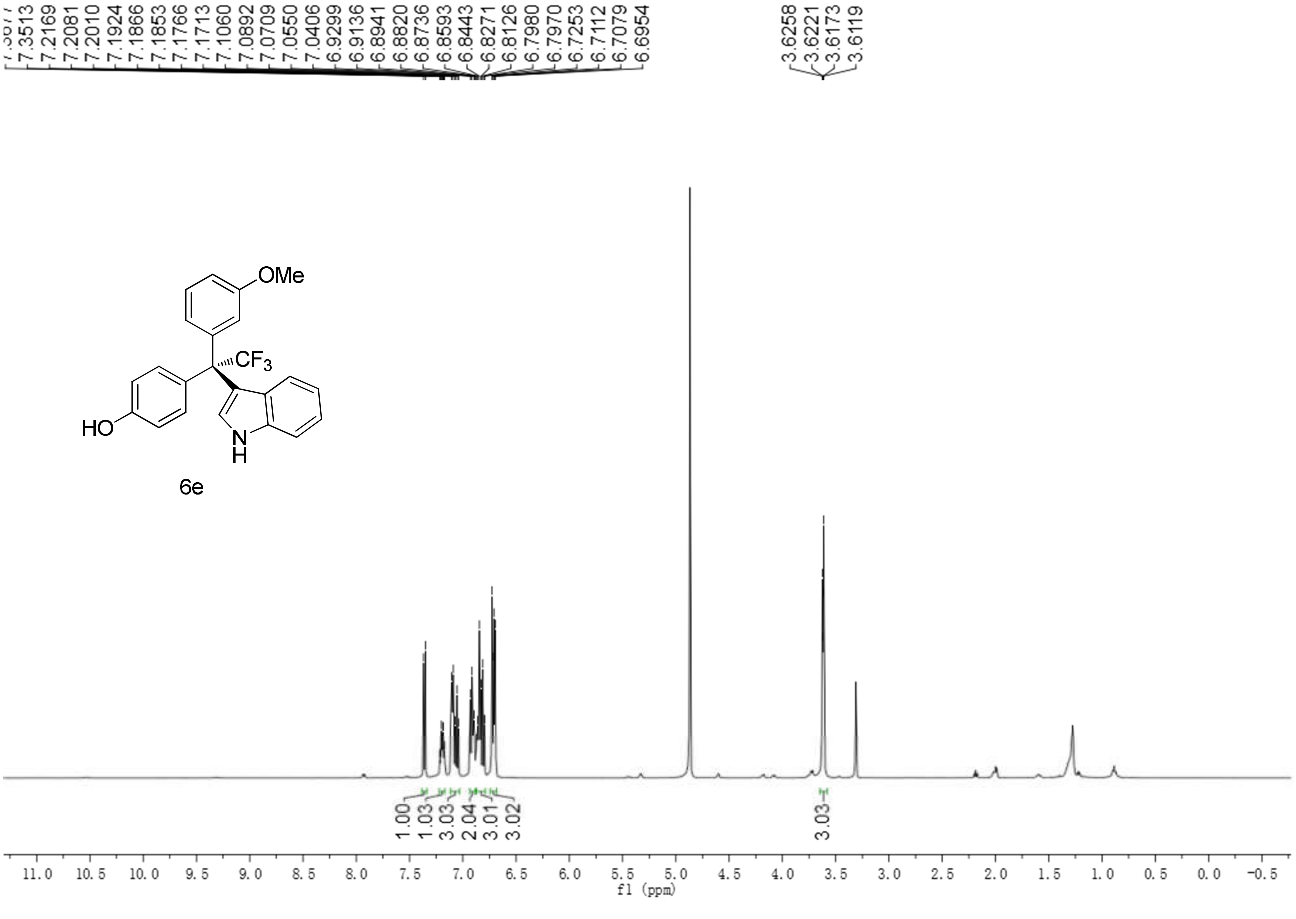

7.3513
7.2169
7.2081
7.2010
7.1924
7.1866
7.1853
7.1766
7.1713
7.1060
7.0892
7.0709
7.0550
7.0406
6.9299
6.9136
6.8941
6.8820
6.8736
6.8593
6.8443
6.8271
6.8126
6.7980
6.7970
6.7253
6.7112
6.7079
6.6954
3.6258
3.6221
3.6173
3.6119
OMe
CF3
HO
N
H
6e
1.00
1.03
3.03
2.04
3.01
3.02
3.03
f1 (ppm)

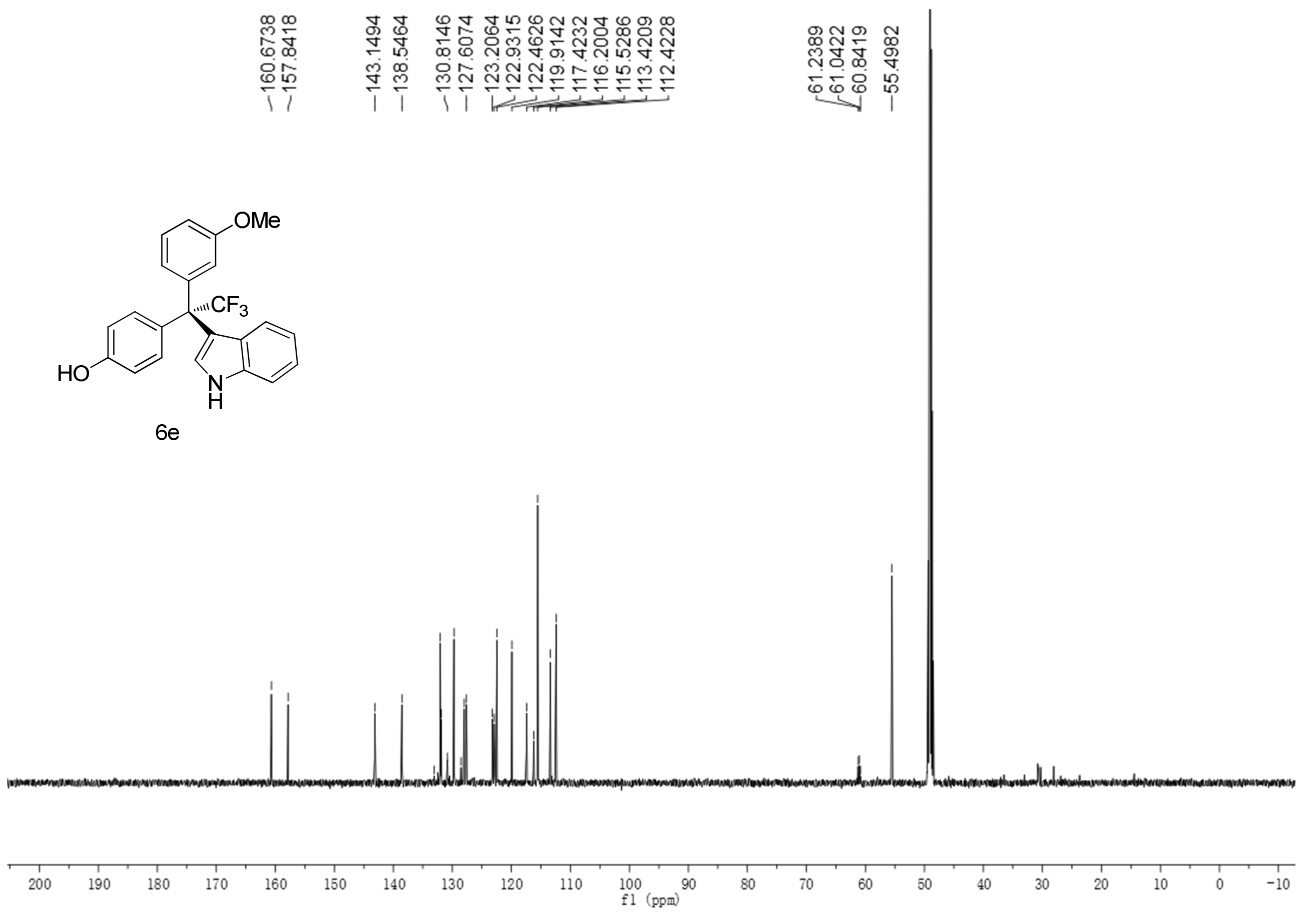
160.6738
157.8418
143.1494
138.5464
130.8146
127.6074
123.2064
122.9315
122.4626
119.9142
117.4232
116.2004
115.5286
113.4209
112.4228
61.2389
61.0422
60.8419
55.4982
OMe
CF3
HO
N
H
6e
f1 (ppm)

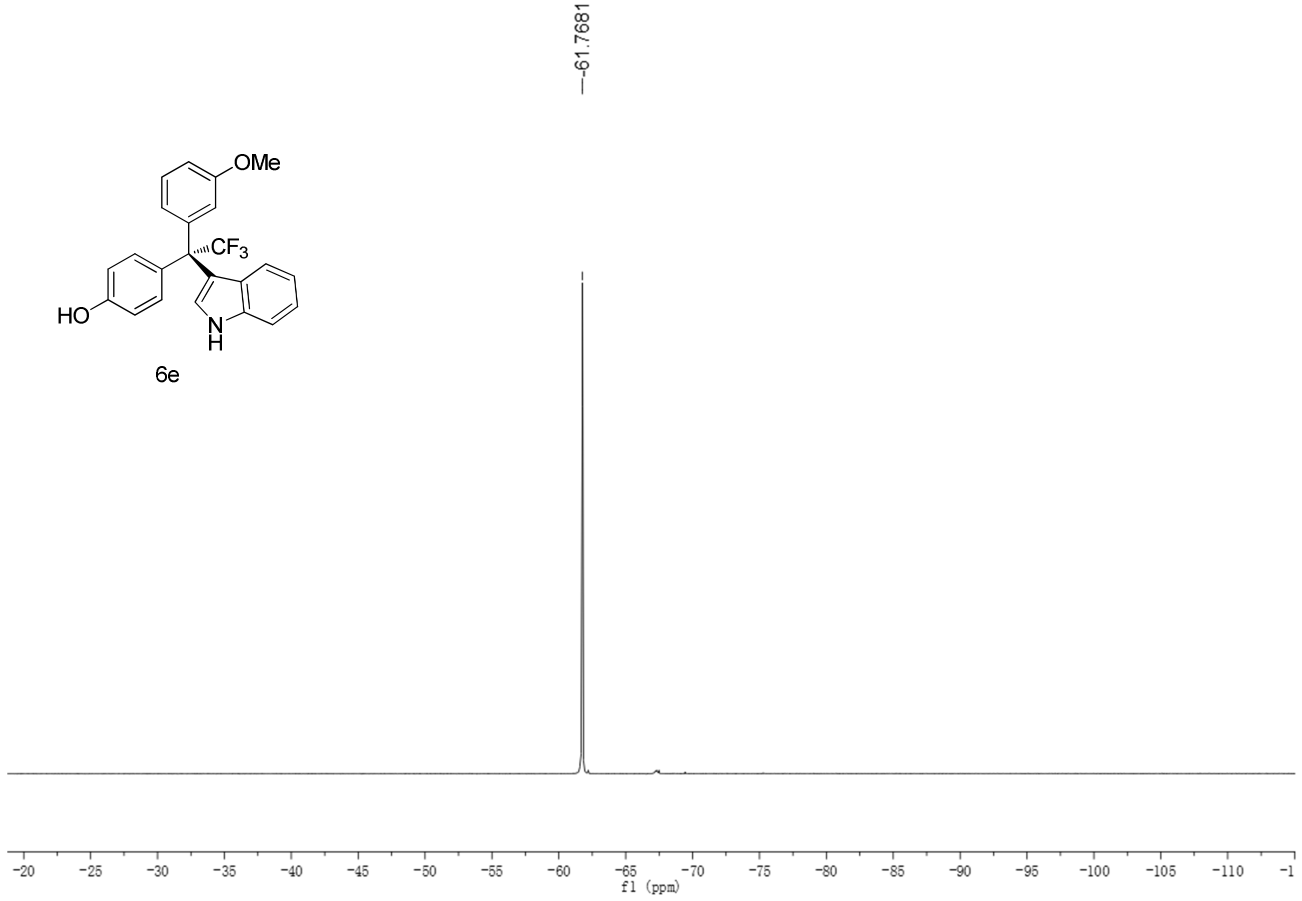
-61.7681
OMe
CF3
HO
N
H
6e
f1 (ppm)

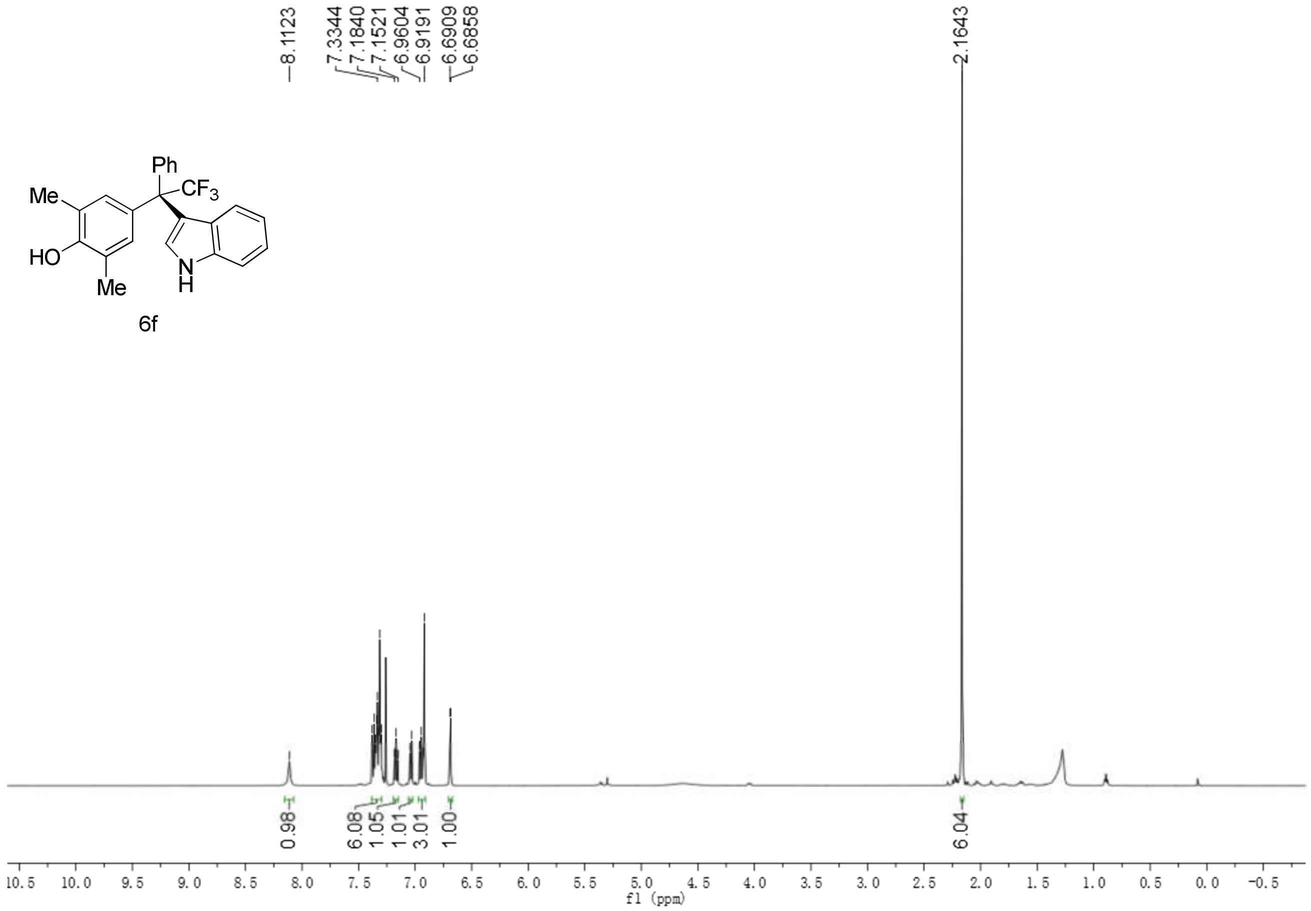

8.1123
7.3344
7.1840
7.1521
6.9604
6.9191
6.6909
6.6858
2.1643
Ph
CF_3
Me
HO
Me
N
H
6f
0.98
6.08
1.05
1.01
3.01
1.00
6.04
f1 (ppm)

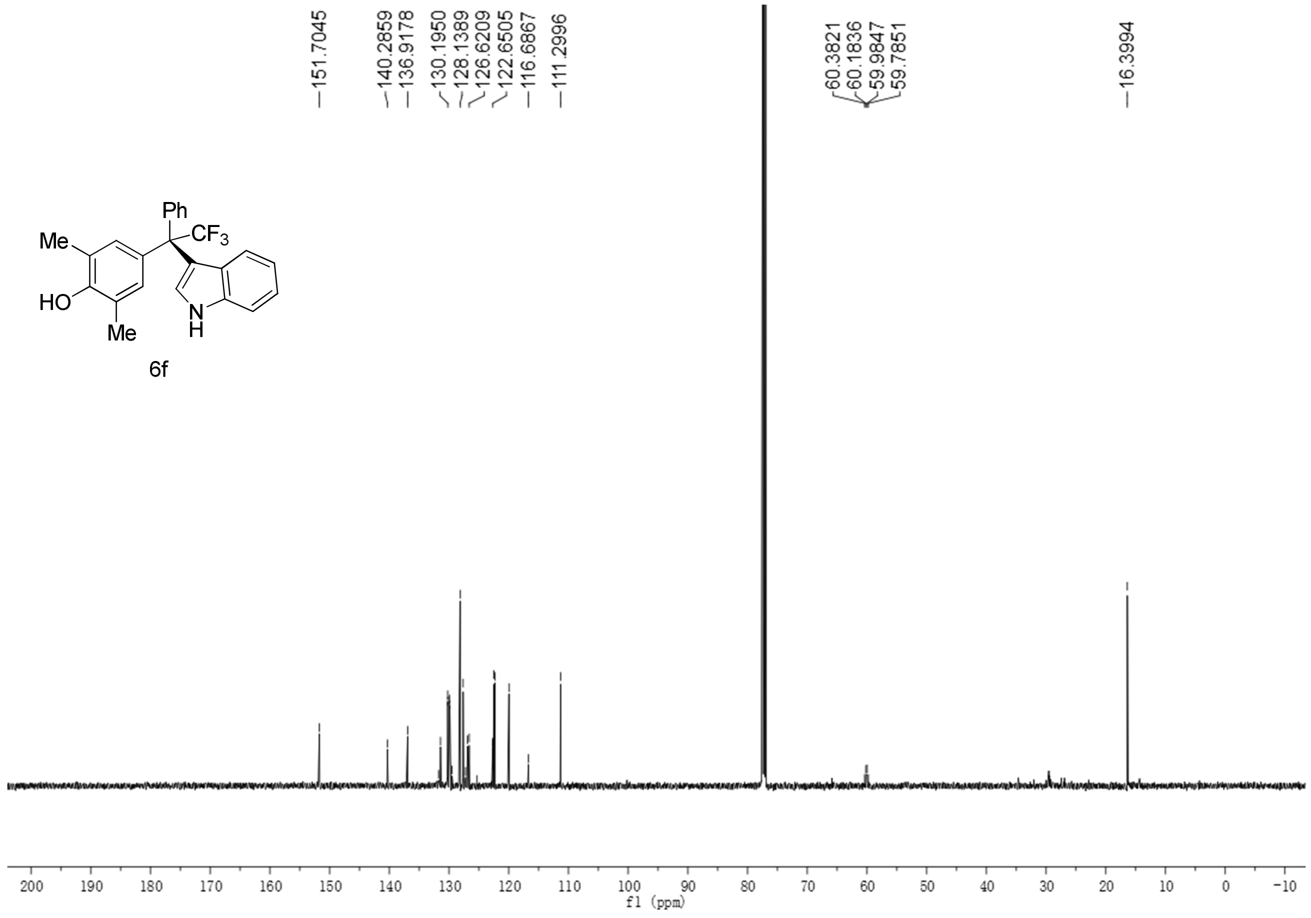

151.7045
140.2859
136.9178
130.1950
128.1389
126.6209
122.6505
116.6867
111.2996
60.3821
60.1836
59.9847
59.7851
16.3994
Ph
CF_3
Me
HO
Me
N
H
6f
f1 (ppm)

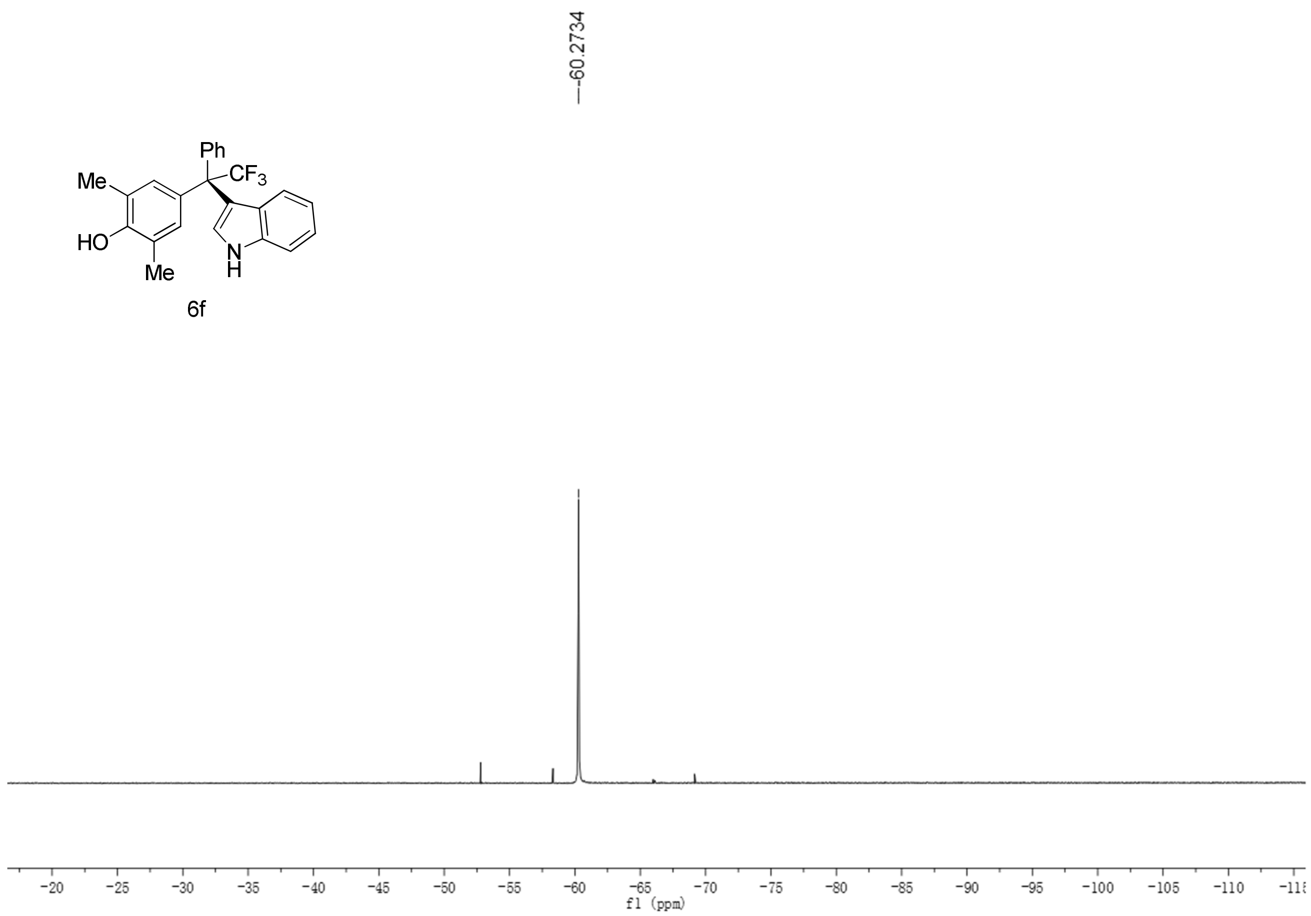

-60.2734
Ph
CF_3
Me
HO
Me
N
H
6f
-20 -25 -30 -35 -40 -45 -50 -55 -60 -65 -70 -75 -80 -85 -90 -95 -100 -105 -110
f1 (ppm)

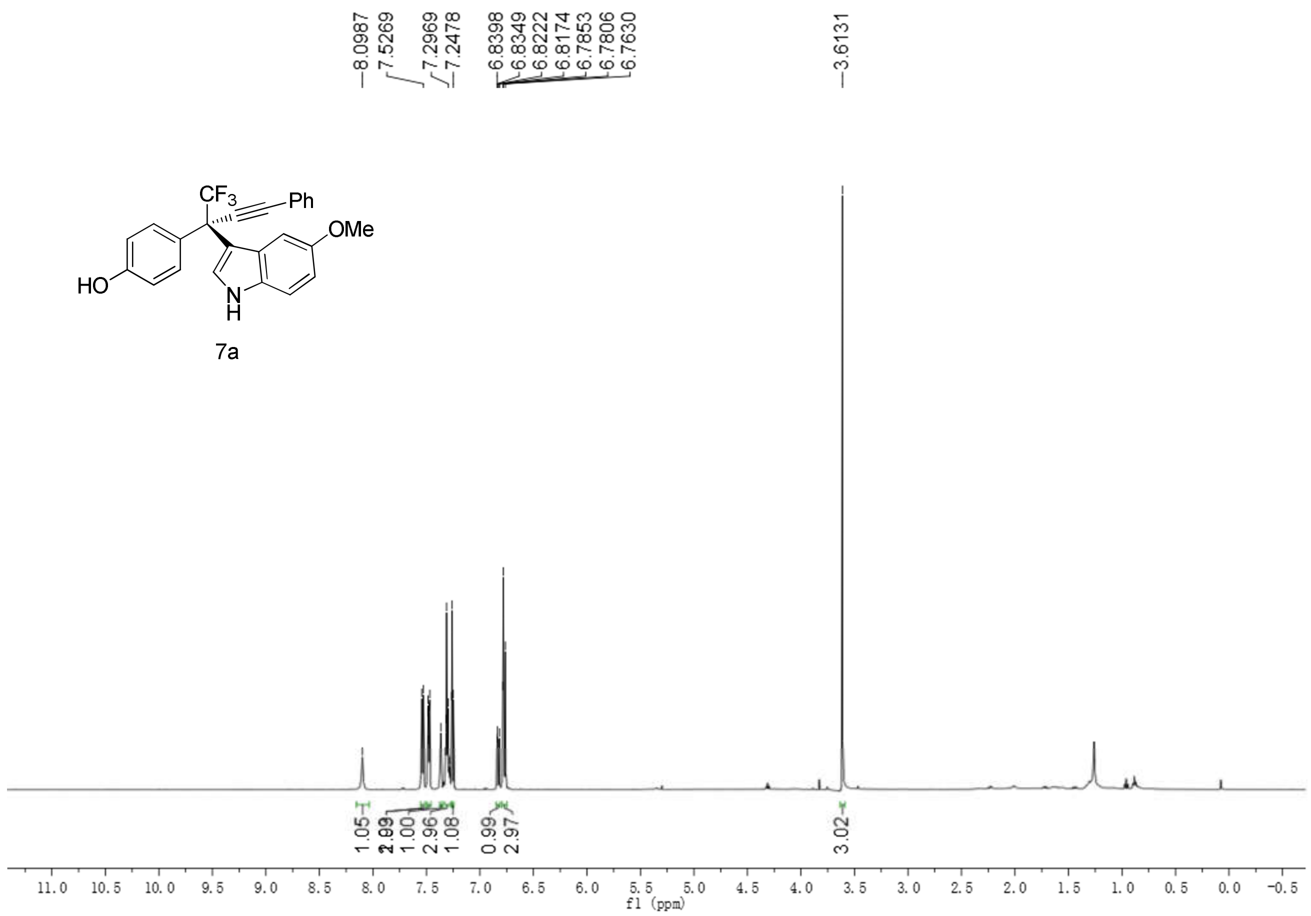

8.0987
7.5269
7.2969
7.2478
6.8398
6.8349
6.8222
6.8174
6.7853
6.7806
6.7630
3.6131
CF_3
Ph
OMe
HO
N
H
7a
1.05
1.00
2.96
1.08
0.99
2.97
3.02
11.0 10.5 10.0 9.5 9.0 8.5 8.0 7.5 7.0 6.5 6.0 5.5 5.0 4.5 4.0 3.5 3.0 2.5 2.0 1.5 1.0 0.5 0.0 -0.5
f1 (ppm)

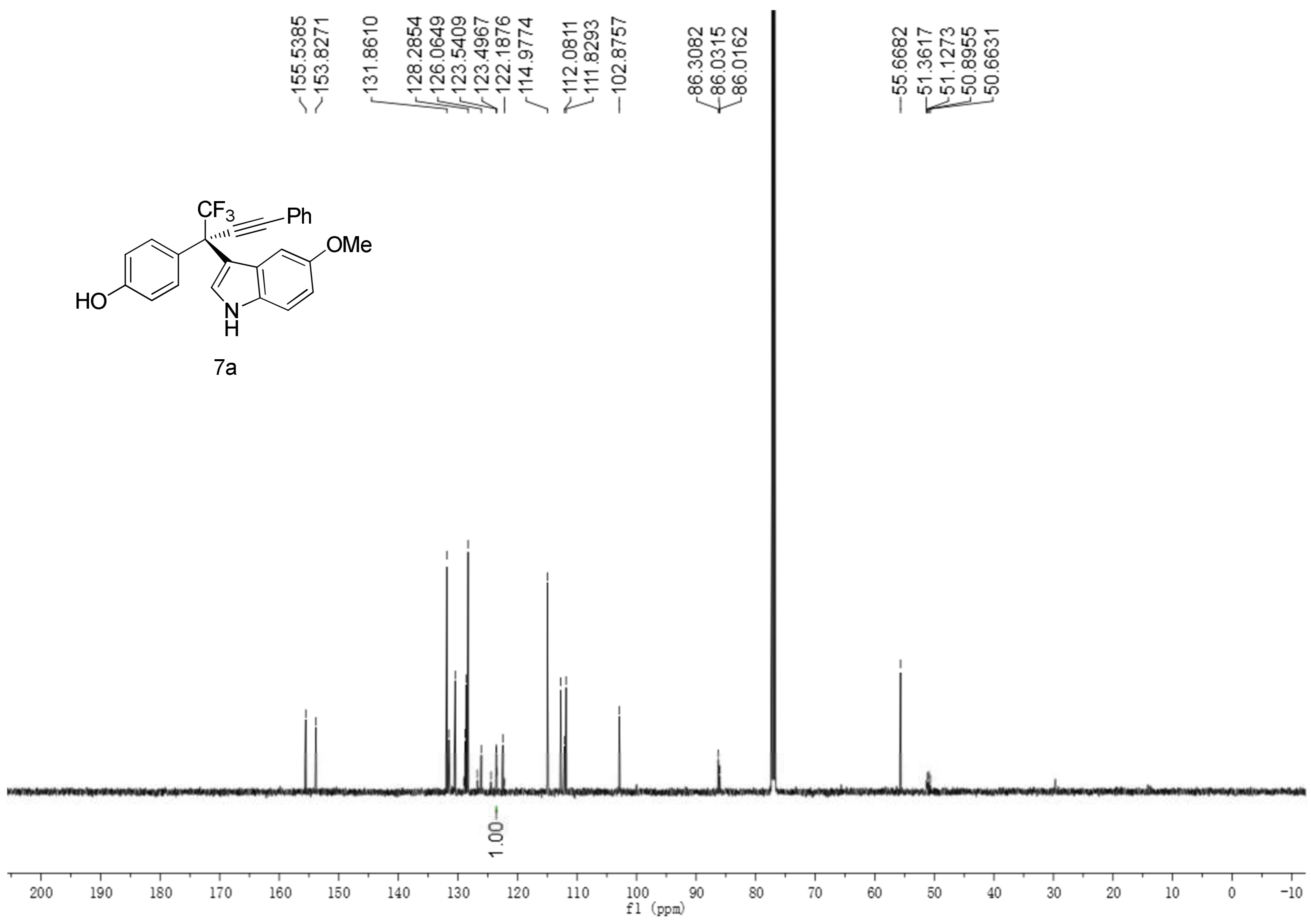
155.5385
153.8271
131.8610
128.2854
126.0649
123.5409
123.4967
122.1876
114.9774
112.0811
111.8293
102.8757
86.3082
86.0315
86.0162
55.6682
51.3617
51.1273
50.8955
50.6631
CF3
Ph
OMe
HO
N
H
7a
1.00
f1 (ppm)

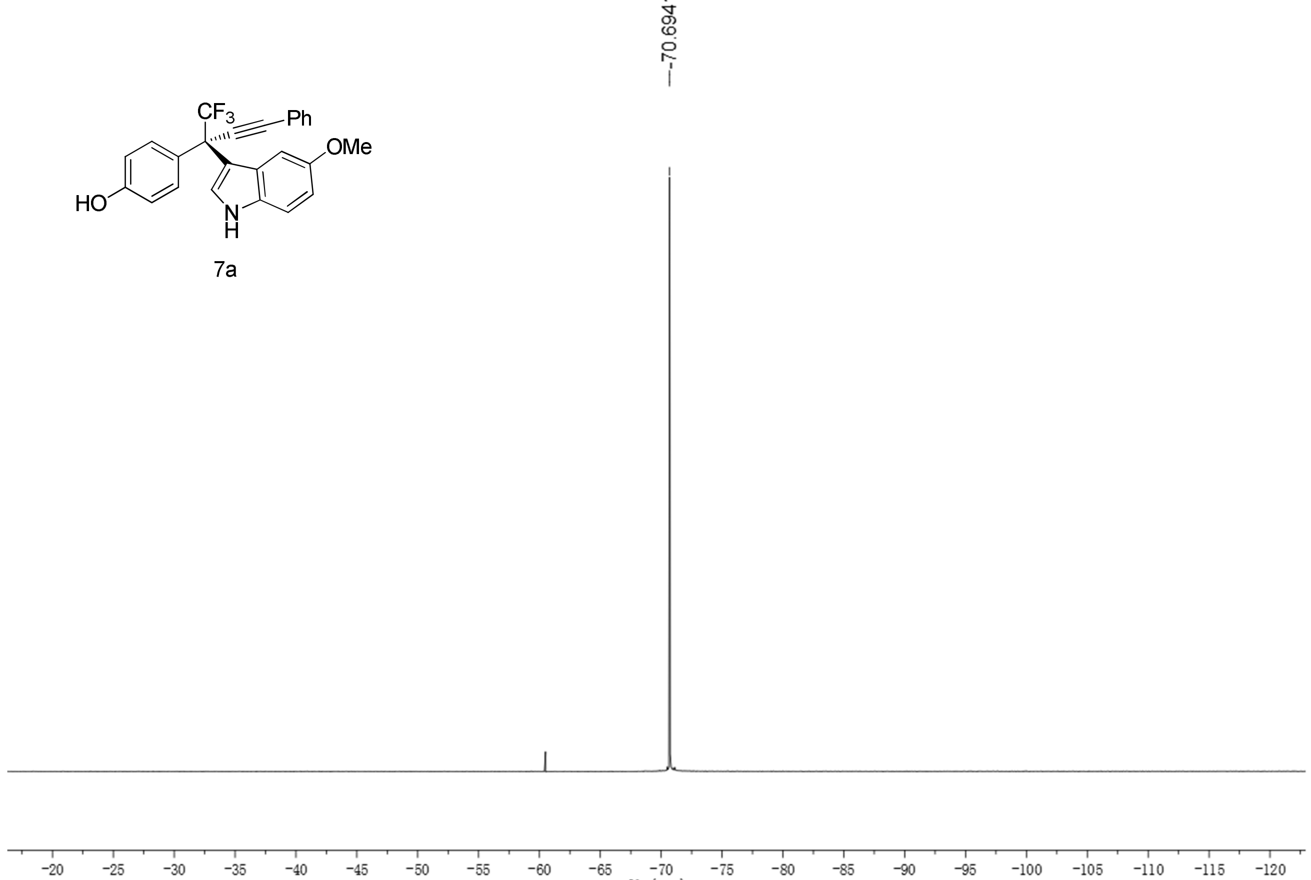
-70.6941
CF3
Ph
OMe
HO
N
H
7a
f1 (ppm)

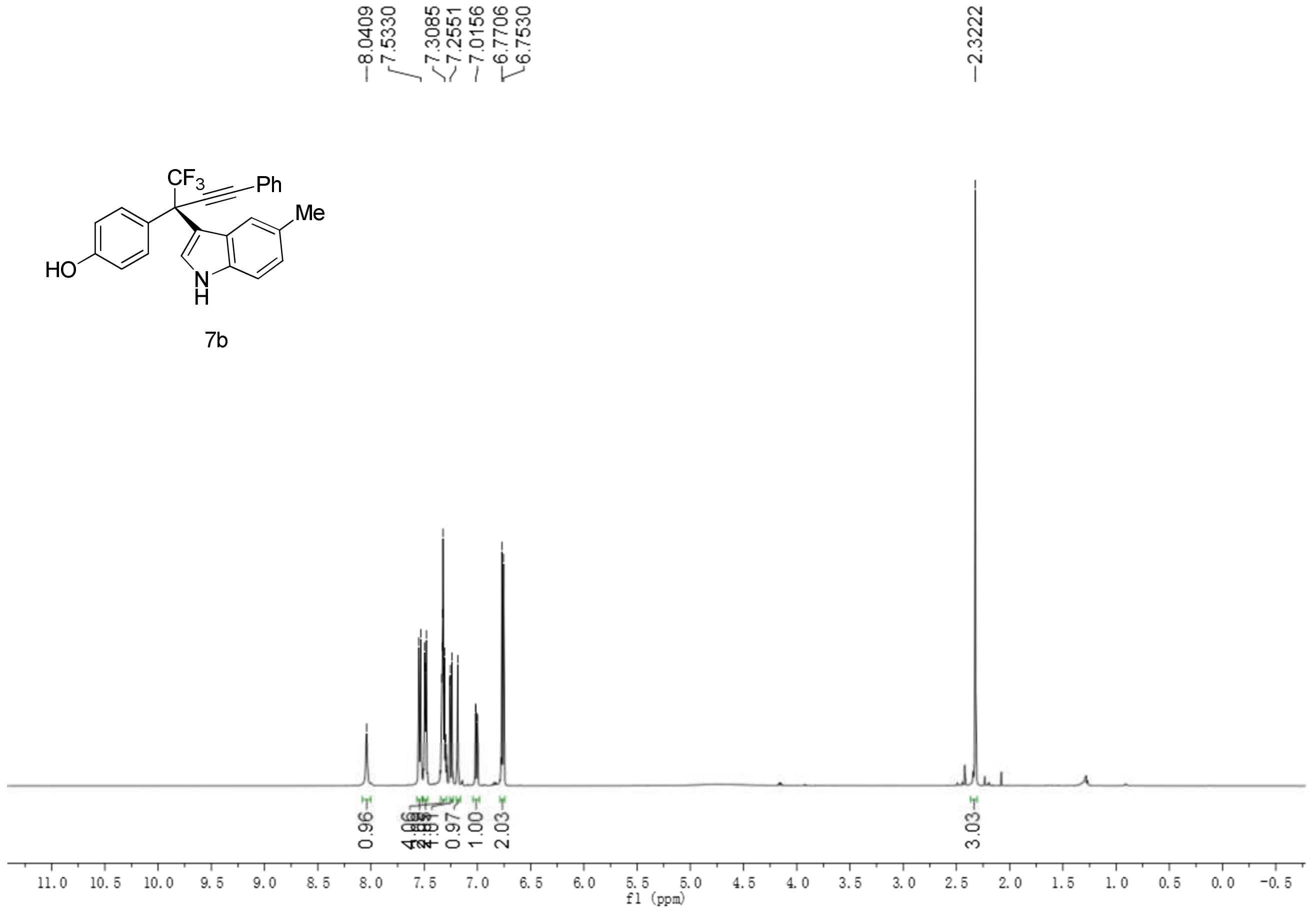
8.0409
7.5330
7.3085
7.2551
7.0156
6.7706
6.7530
2.3222
CF3
Ph
Me
HO
N
H
7b
0.96
4.06
0.97
1.00
2.03
3.03
11.0 10.5 10.0 9.5 9.0 8.5 8.0 7.5 7.0 6.5 6.0 5.5 5.0 4.5 4.0 3.5 3.0 2.5 2.0 1.5 1.0 0.5 0.0 -0.5
f1 (ppm)

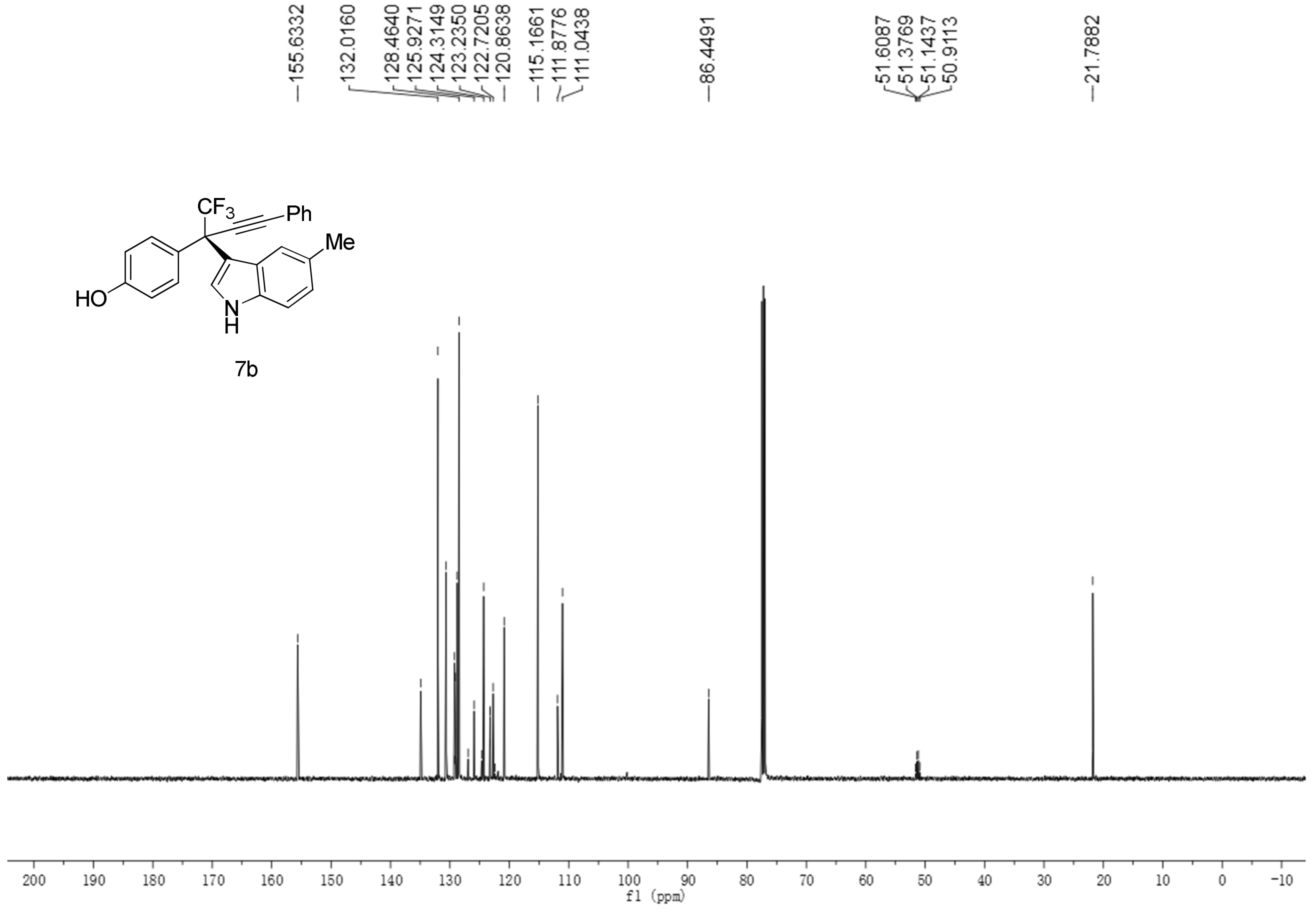
155.6332
132.0160
128.4640
125.9271
124.3149
123.2350
122.7205
120.8638
115.1661
111.8776
111.0438
86.4491
51.6087
51.3769
51.1437
50.9113
21.7882
CF3
Ph
Me
HO
N
H
7b
200 190 180 170 160 150 140 130 120 110 100 90 80 70 60 50 40 30 20 10 0 -10
f1 (ppm)

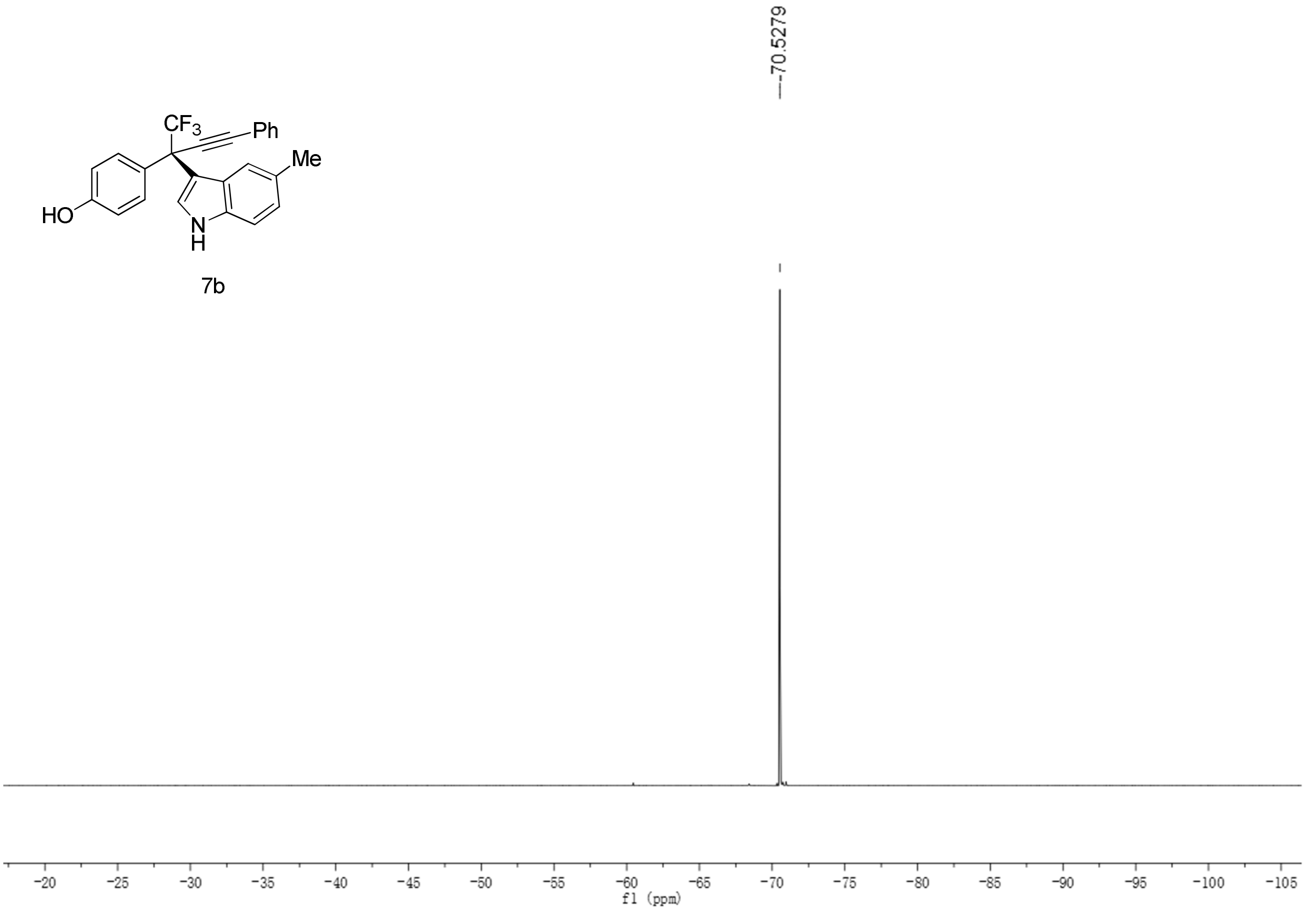
CF3
Ph
Me
HO
N
H
-70.5279
f1 (ppm)

7b

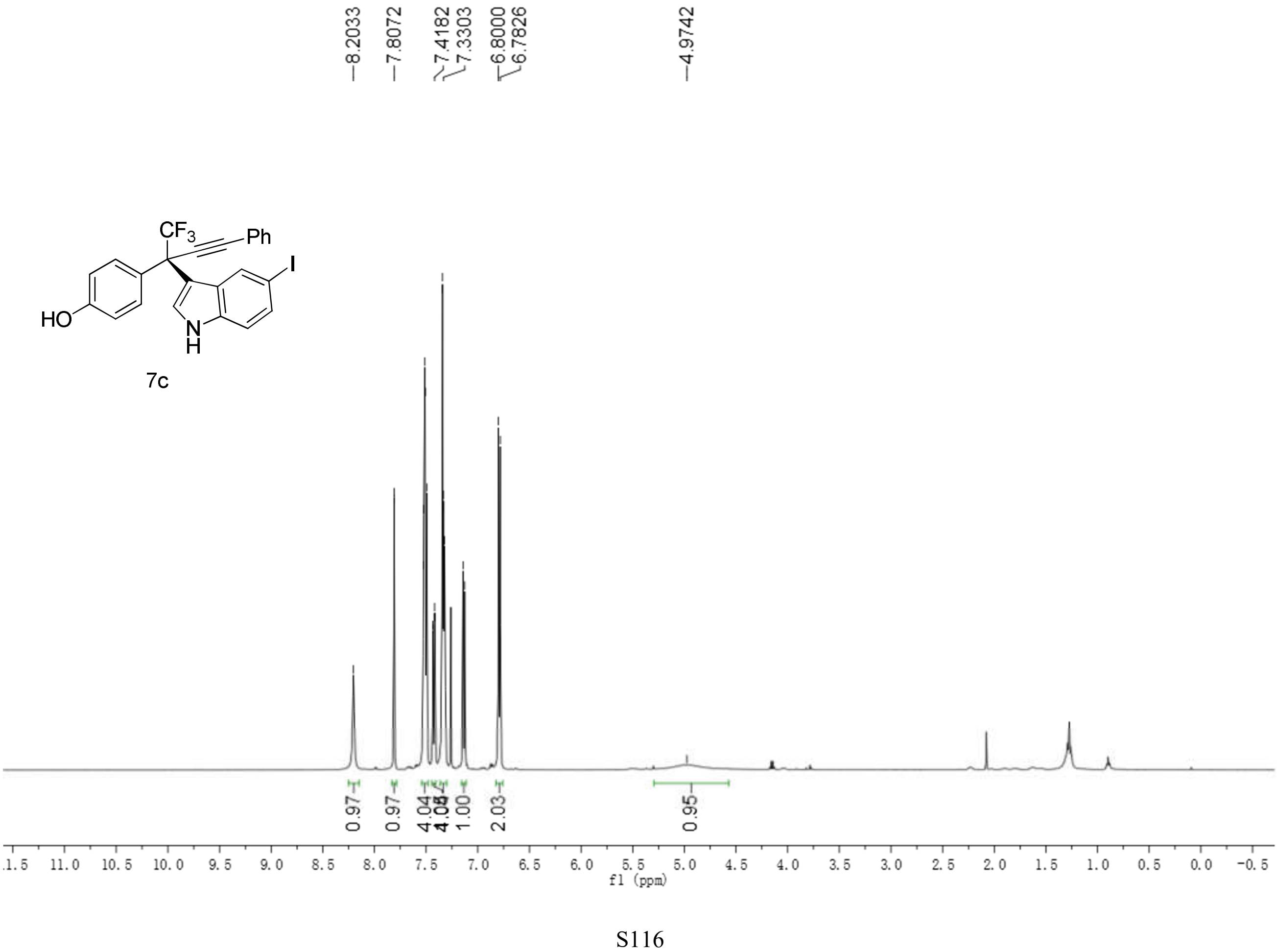
8.2033
7.8072
7.4182
7.3303
6.8000
6.7826
4.9742
CF3
Ph
I
HO
N
H
0.97
0.97
4.04
1.00
2.03
0.95
f1 (ppm)

7c

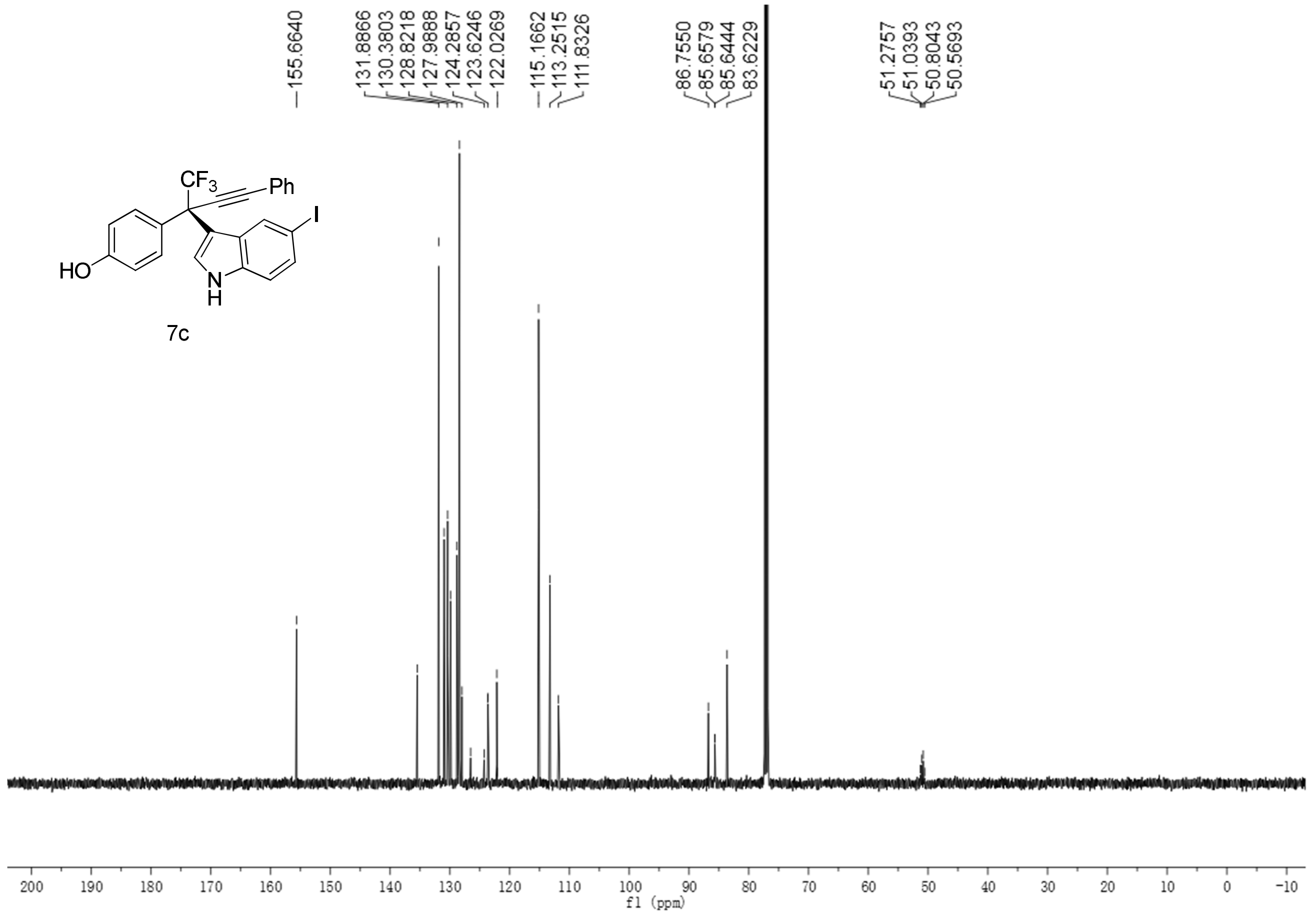
155.6640
131.8866
130.3803
128.8218
127.9888
124.2857
123.6246
122.0269
115.1662
113.2515
111.8326
86.7550
85.6579
85.6444
83.6229
51.2757
51.0393
50.8043
50.5693
CF3
Ph
I
HO
N
H
7c
f1 (ppm)

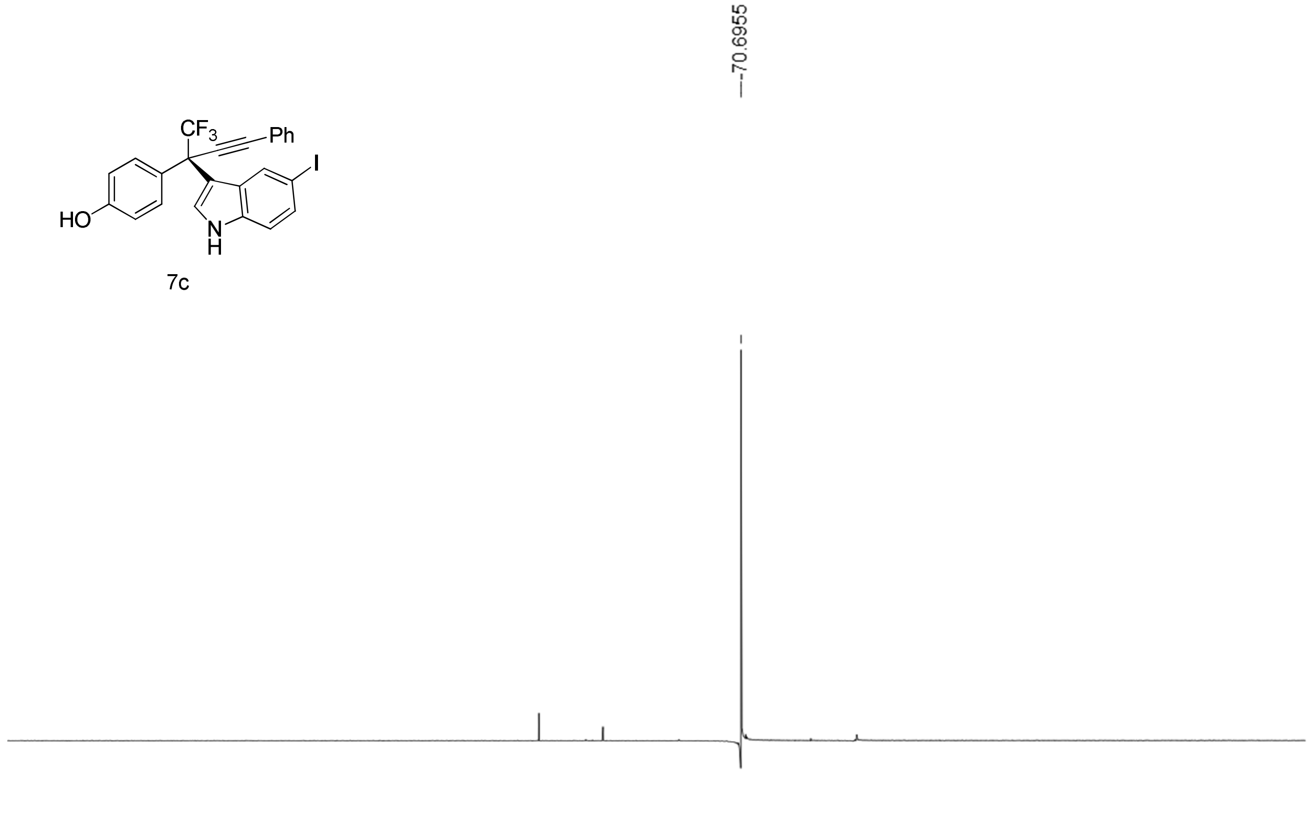
-70.6955
CF3
Ph
I
HO
N
H
7c
f1 (ppm)

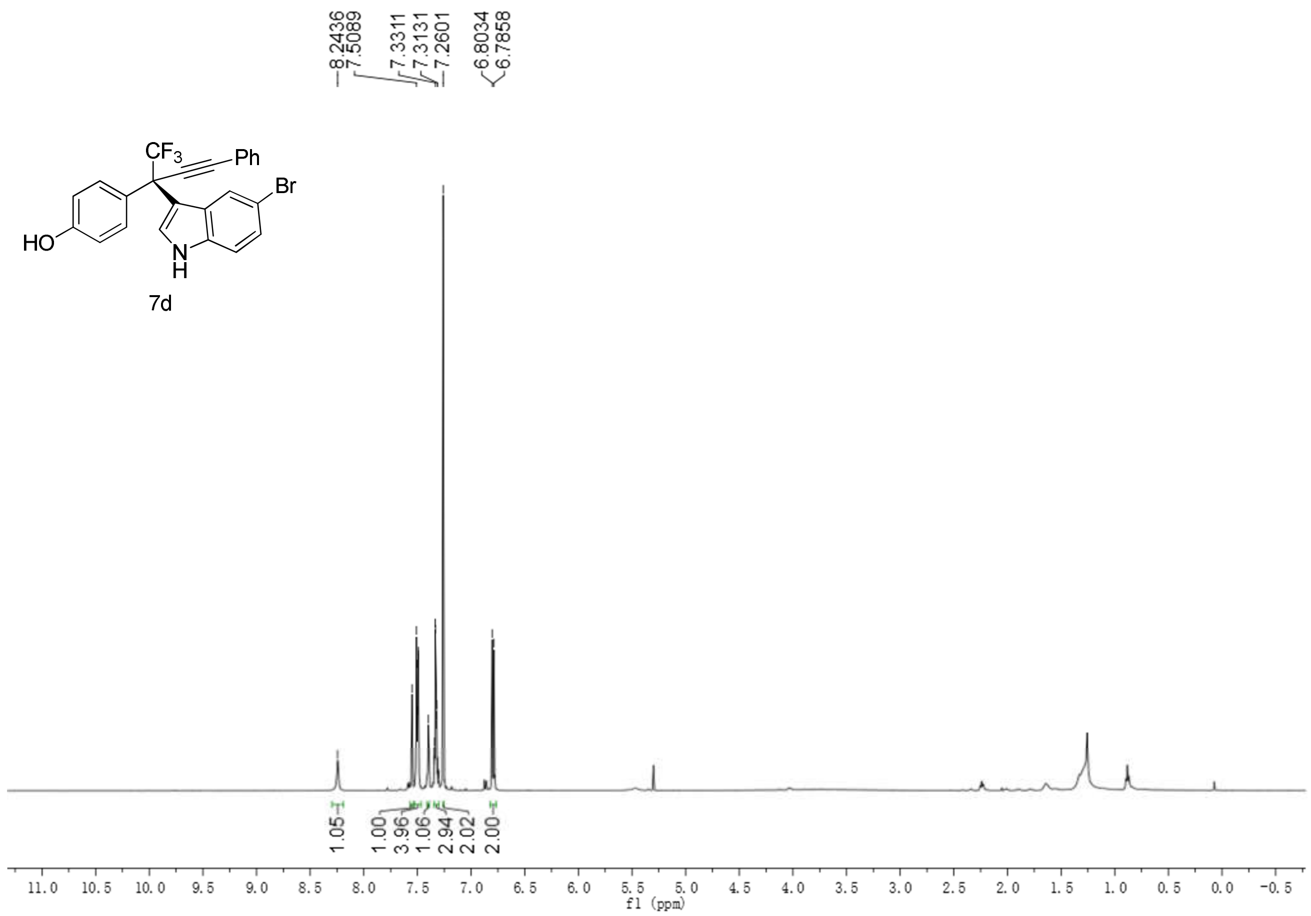

8.2436
7.5089
7.3311
7.3131
7.2601
6.8034
6.7858
CF3
Ph
Br
HO
N
H
7d
1.05
1.00
3.96
1.06
2.94
2.02
2.00
f1 (ppm)

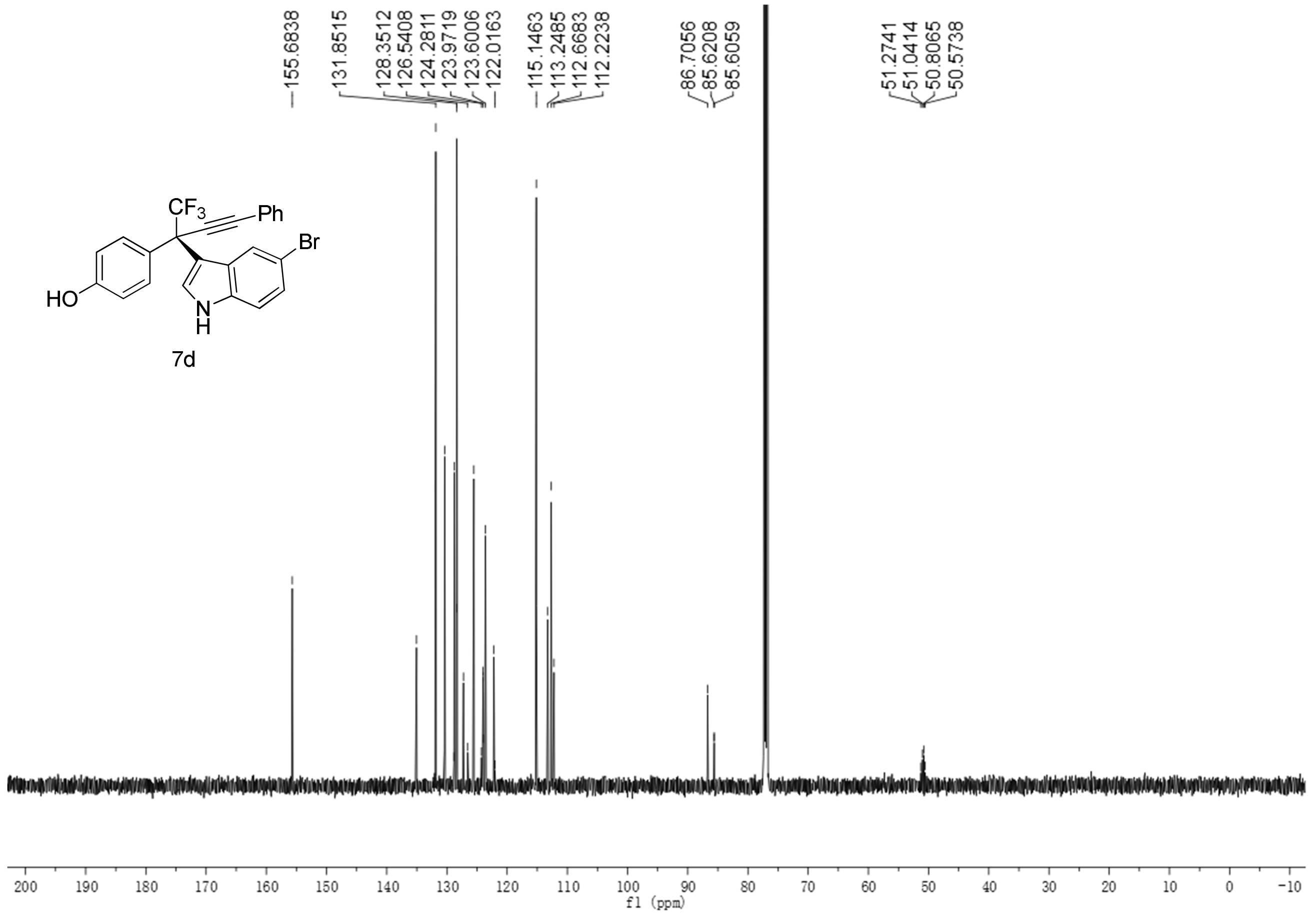

155.6838
131.8515
128.3512
126.5408
124.2811
123.9719
123.6006
122.0163
115.1463
113.2485
112.6683
112.2238
86.7056
85.6208
85.6059
51.2741
51.0414
50.8065
50.5738
CF3
Ph
Br
HO
N
H
7d
f1 (ppm)

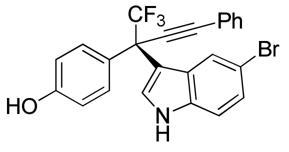

7d

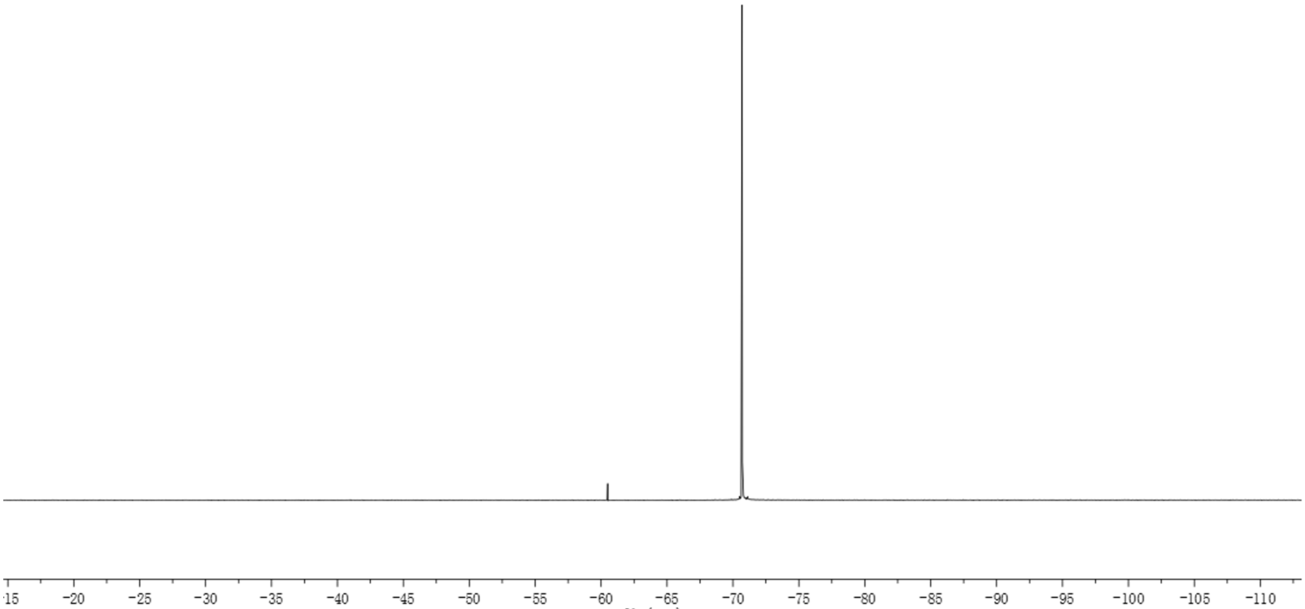
-70.6941
f1 (ppm)

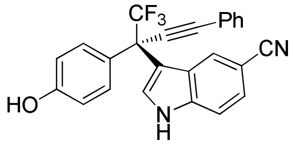

7e

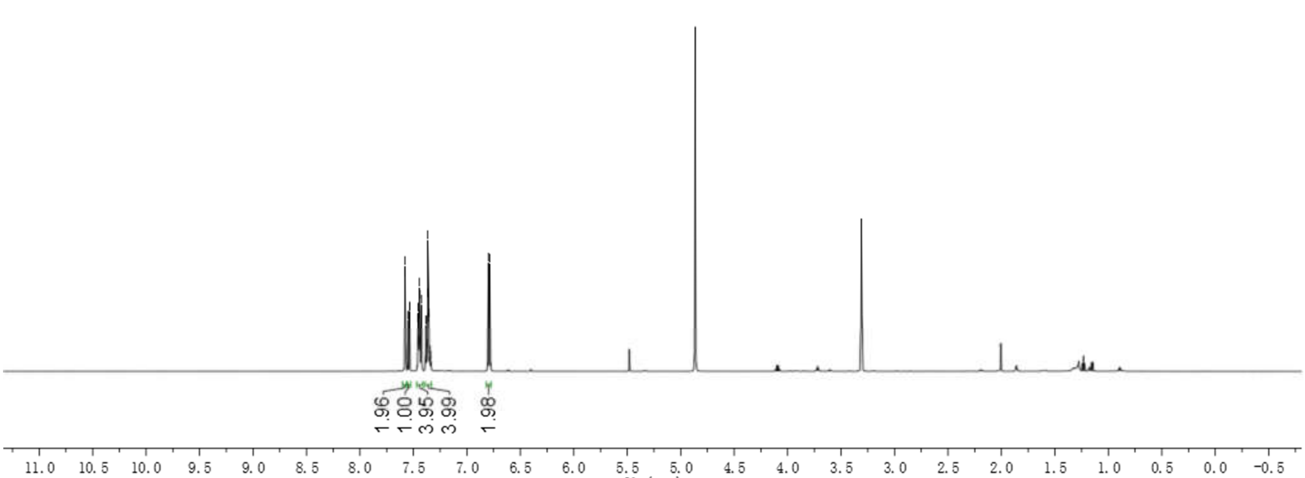
7.5357
7.5348
7.4594
7.4565
7.4522
7.4505
7.4472
7.4433
7.4405
7.4257
7.3826
7.3800
7.3754
7.3729
7.3679
7.3656
7.3602
7.3532
7.3444
7.3383
6.7997
6.7849
1.96
1.00
3.95
3.99
1.98
f1 (ppm)

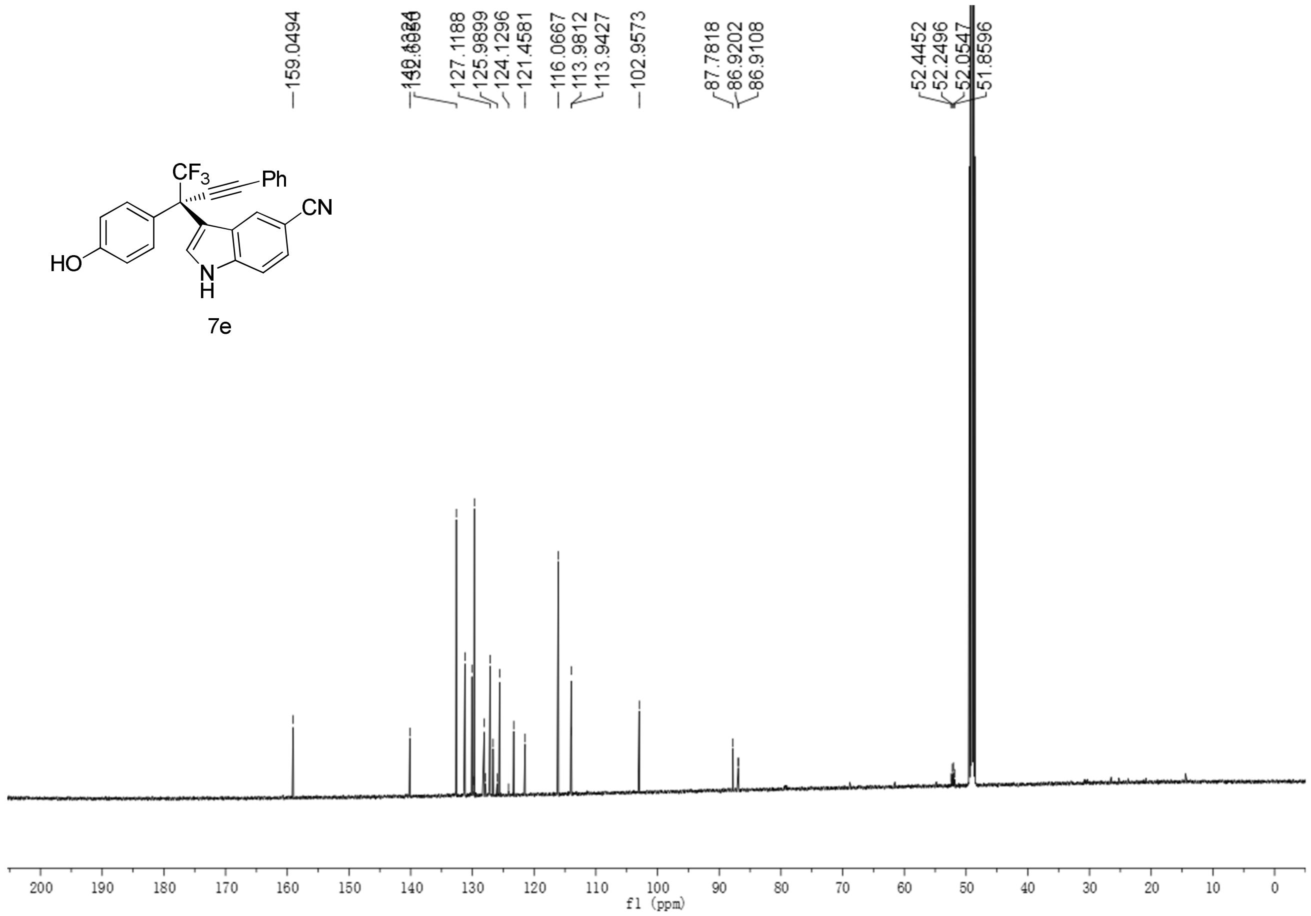
159.0494
127.1188
125.9899
124.1296
121.4581
116.0667
113.9812
113.9427
102.9573
87.7818
86.9202
86.9108
52.4452
52.2496
52.0547
51.8596
CF3
Ph
CN
HO
N
H
7e
f1 (ppm)

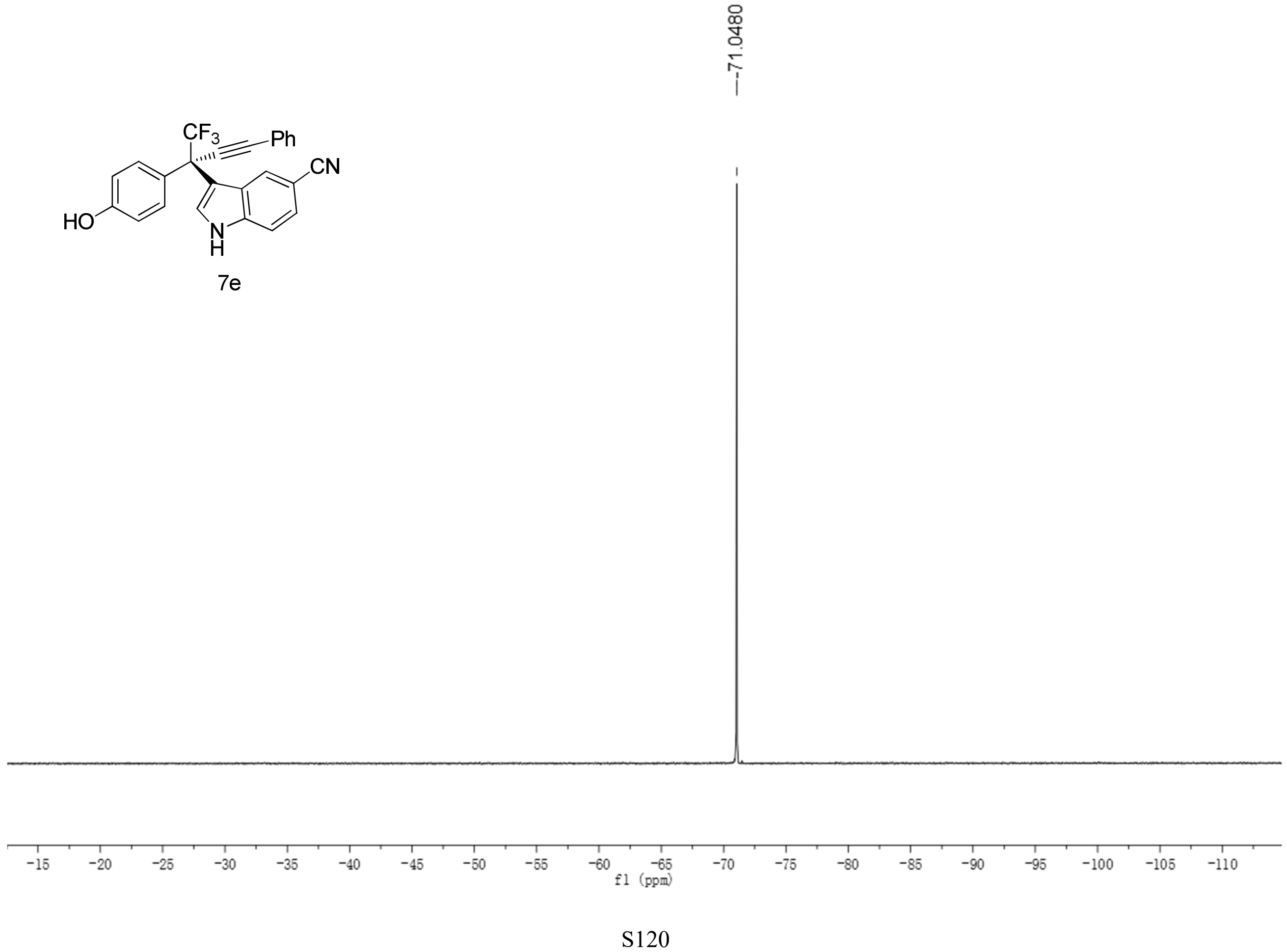
-71.0480
CF3
Ph
CN
HO
N
H
7e
f1 (ppm)

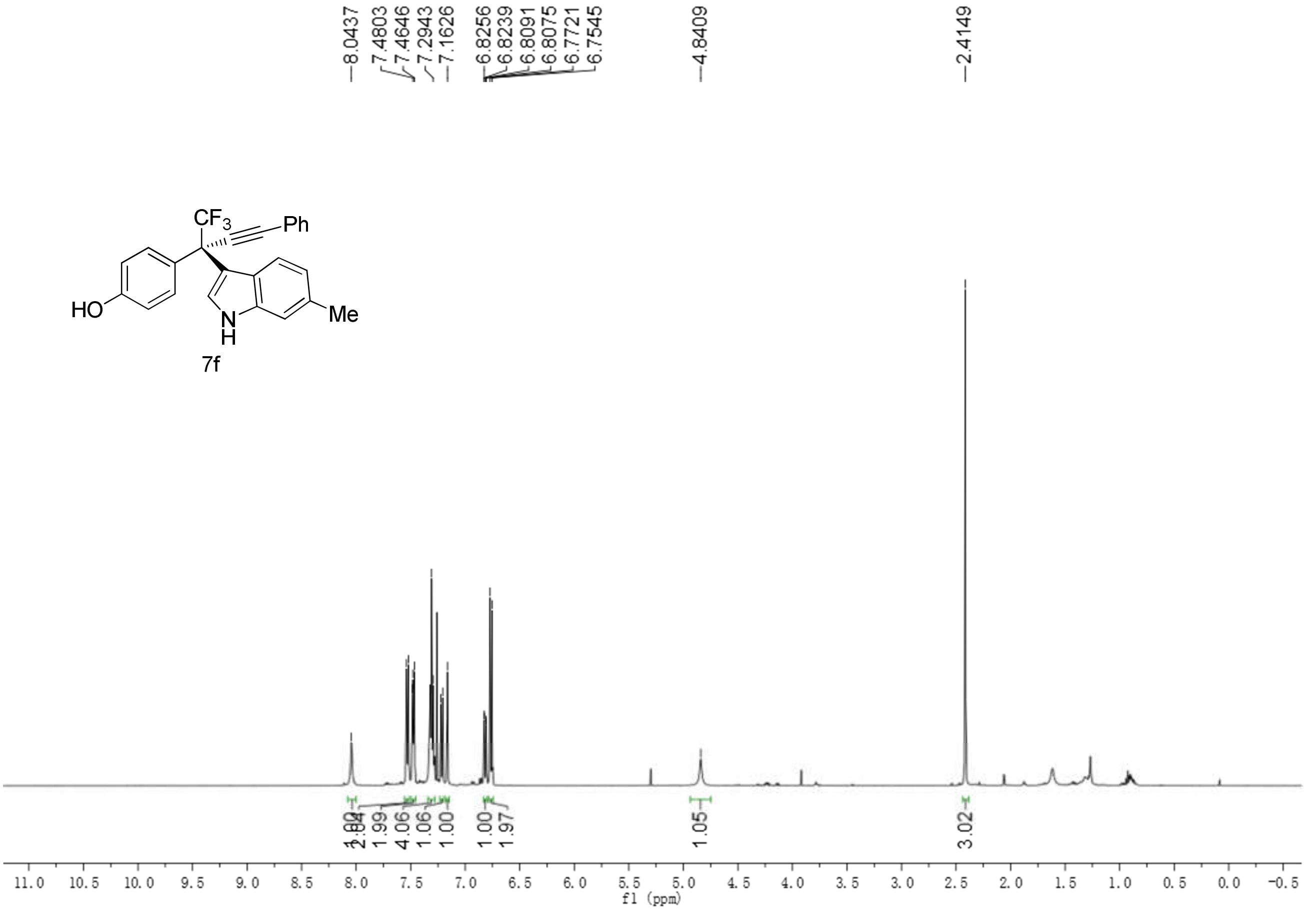
8.0437
7.4803
7.4646
7.2943
7.1626
6.8256
6.8239
6.8091
6.8075
6.7721
6.7545
4.8409
2.4149
CF3
Ph
HO
N
H
Me
7f
1.00
2.04
1.99
4.06
1.06
1.00
1.00
1.97
1.05
3.02
f1 (ppm)

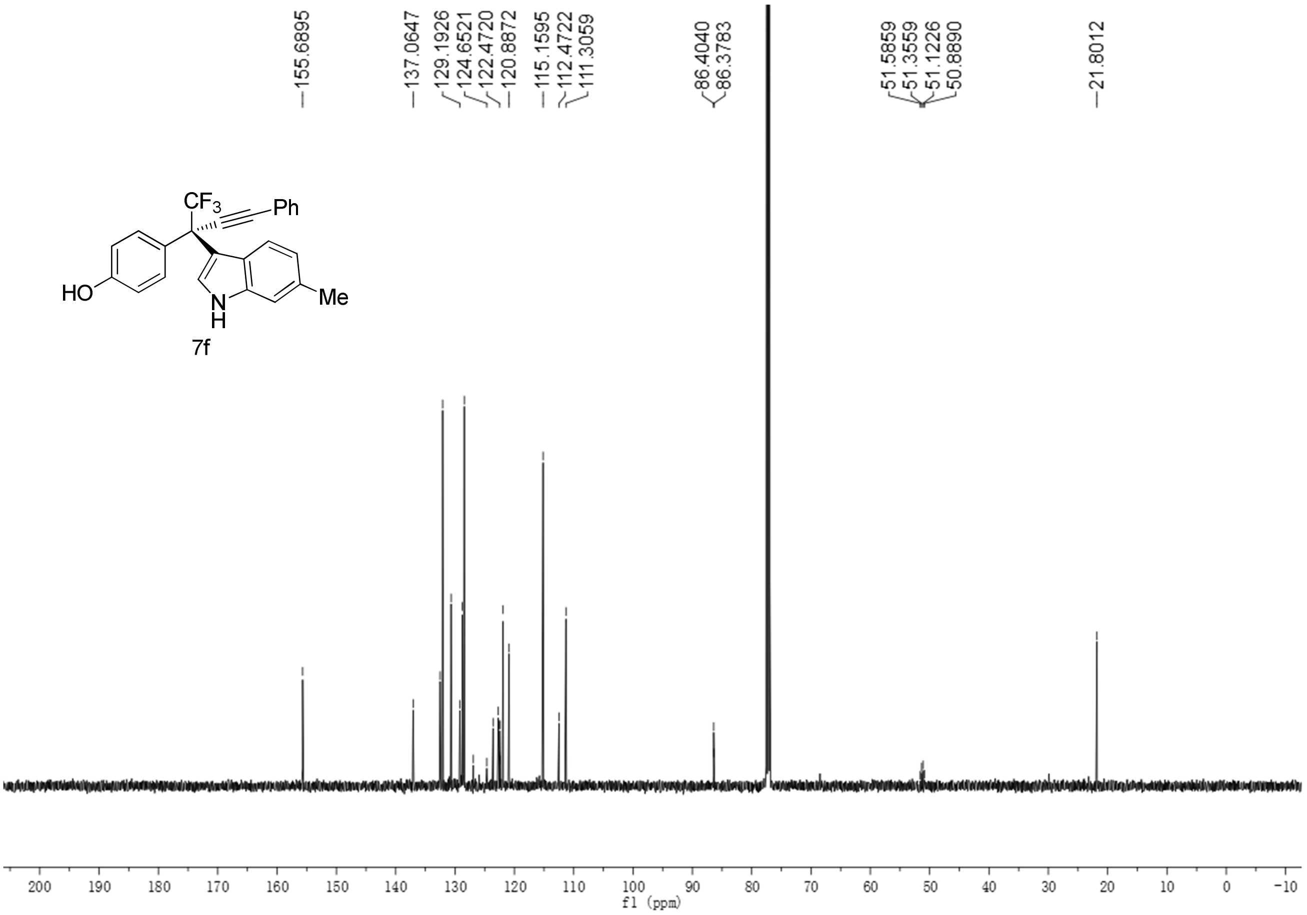
155.6895
137.0647
129.1926
124.6521
122.4720
120.8872
115.1595
112.4722
111.3059
86.4040
86.3783
51.5859
51.3559
51.1226
50.8890
21.8012
CF3
Ph
HO
N
H
Me
7f
f1 (ppm)

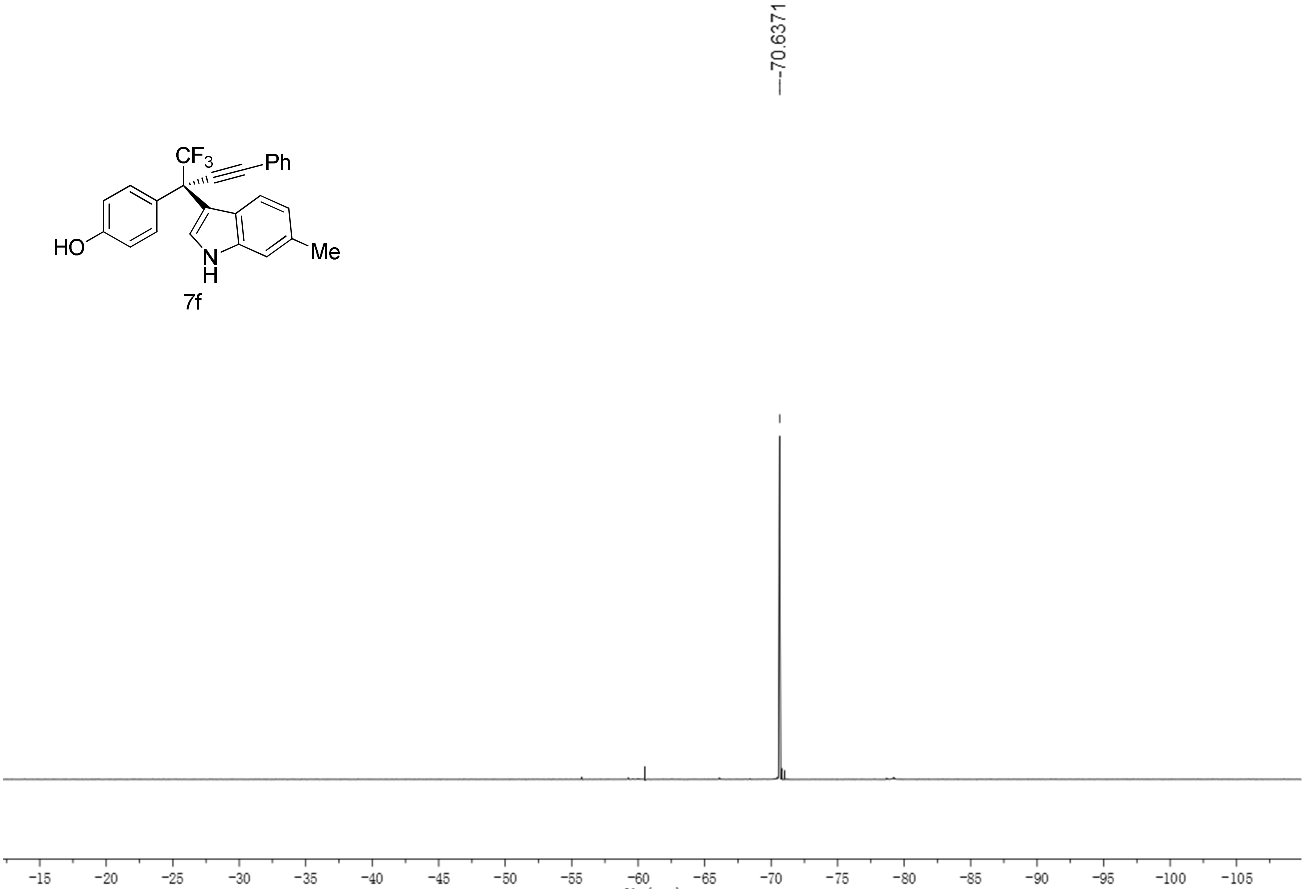

7f

-70.6371
-15 -20 -25 -30 -35 -40 -45 -50 -55 -60 -65 -70 -75 -80 -85 -90 -95 -100 -105
f1 (ppm)

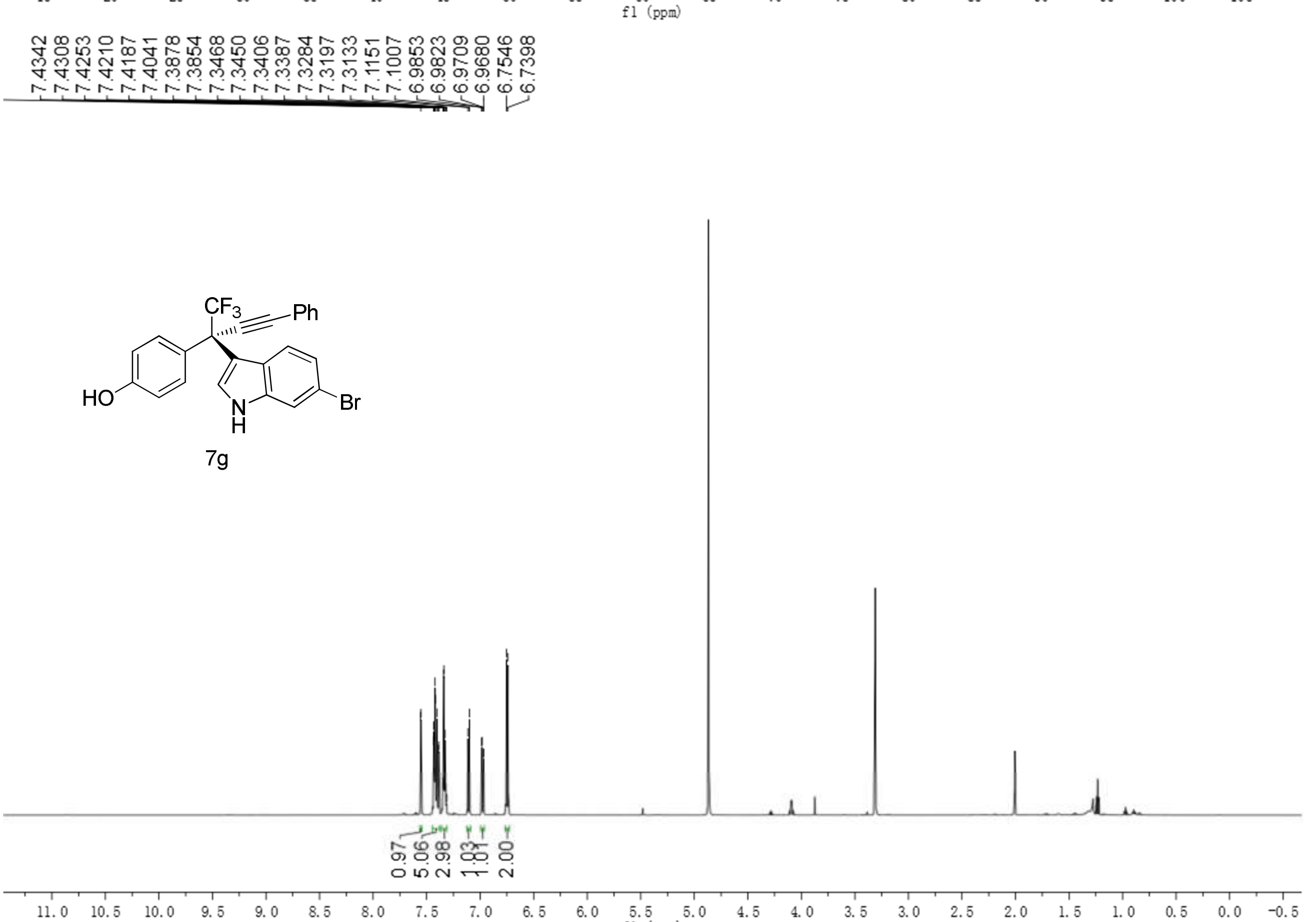

7g

7.4342
7.4308
7.4253
7.4210
7.4187
7.4041
7.3878
7.3854
7.3468
7.3450
7.3406
7.3387
7.3284
7.3197
7.3133
7.1151
7.1007
6.9853
6.9823
6.9709
6.9680
6.7546
6.7398
0.97
5.06
2.98
1.03
1.01
2.00
11.0 10.5 10.0 9.5 9.0 8.5 8.0 7.5 7.0 6.5 6.0 5.5 5.0 4.5 4.0 3.5 3.0 2.5 2.0 1.5 1.0 0.5 0.0 -0.5
f1 (ppm)

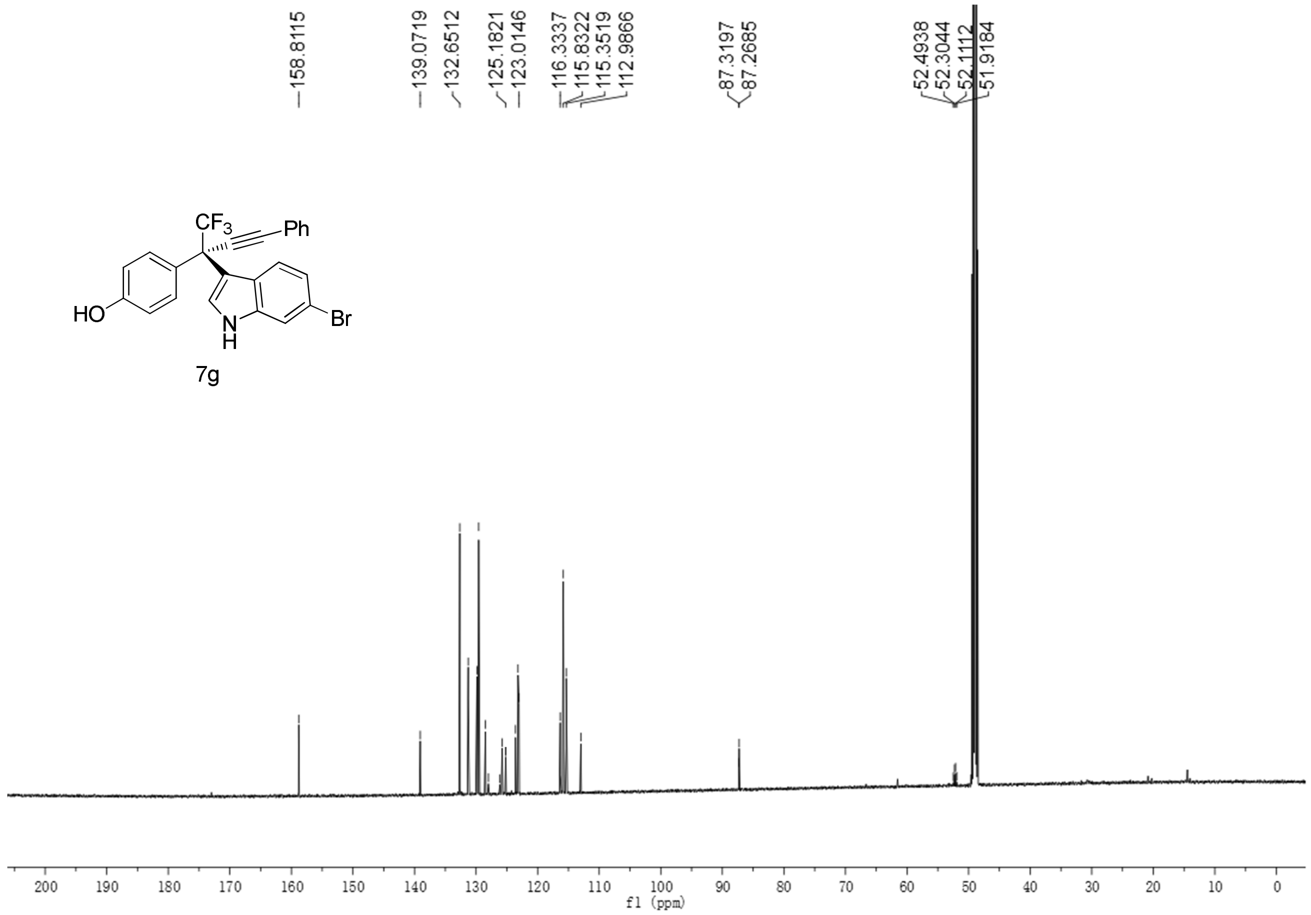
158.8115
139.0719
132.6512
125.1821
123.0146
116.3337
115.8322
115.3519
112.9866
87.3197
87.2685
52.4938
52.3044
52.1112
51.9184
CF3
Ph
HO
Br
N
H
7g
f1 (ppm)

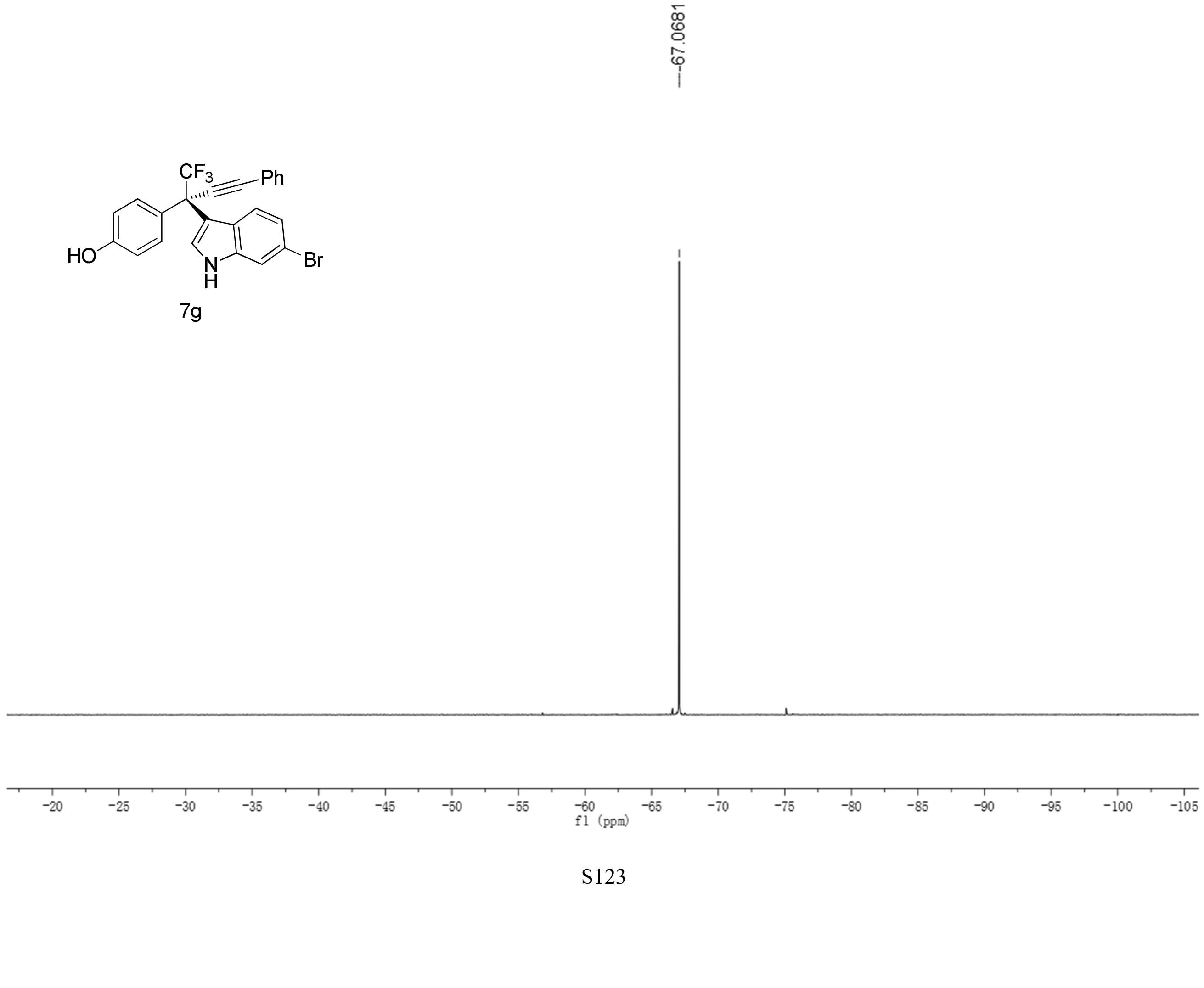
-67.0681
CF3
Ph
HO
Br
N
H
7g
f1 (ppm)

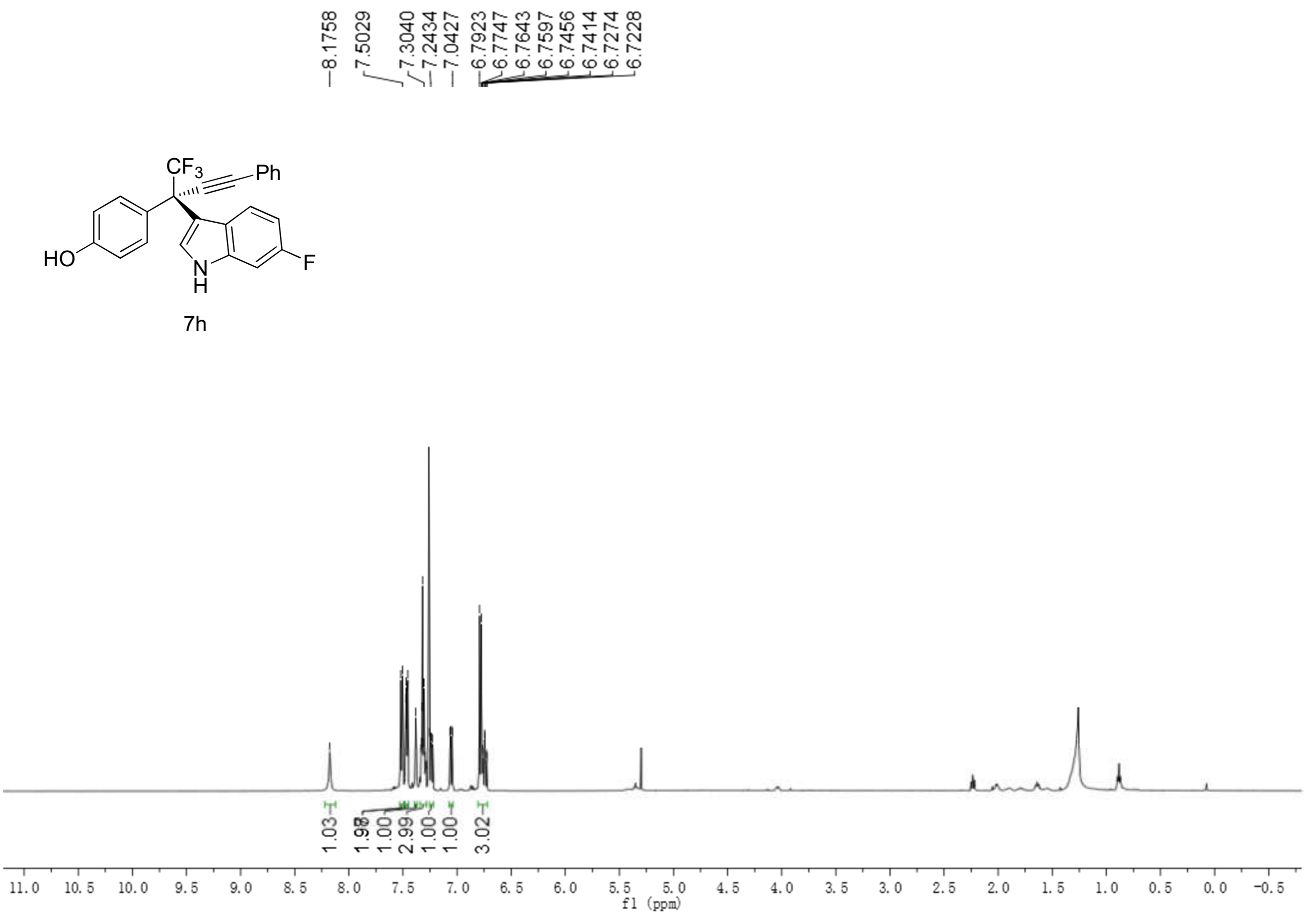

8.1758
7.5029
7.3040
7.2434
7.0427
6.7923
6.7747
6.7643
6.7597
6.7456
6.7414
6.7274
6.7228
CF3
Ph
HO
N
H
F
7h
1.03
1.98
1.00
2.99
1.00
1.00
3.02
11.0 10.5 10.0 9.5 9.0 8.5 8.0 7.5 7.0 6.5 6.0 5.5 5.0 4.5 4.0 3.5 3.0 2.5 2.0 1.5 1.0 0.5 0.0 -0.5
f1 (ppm)

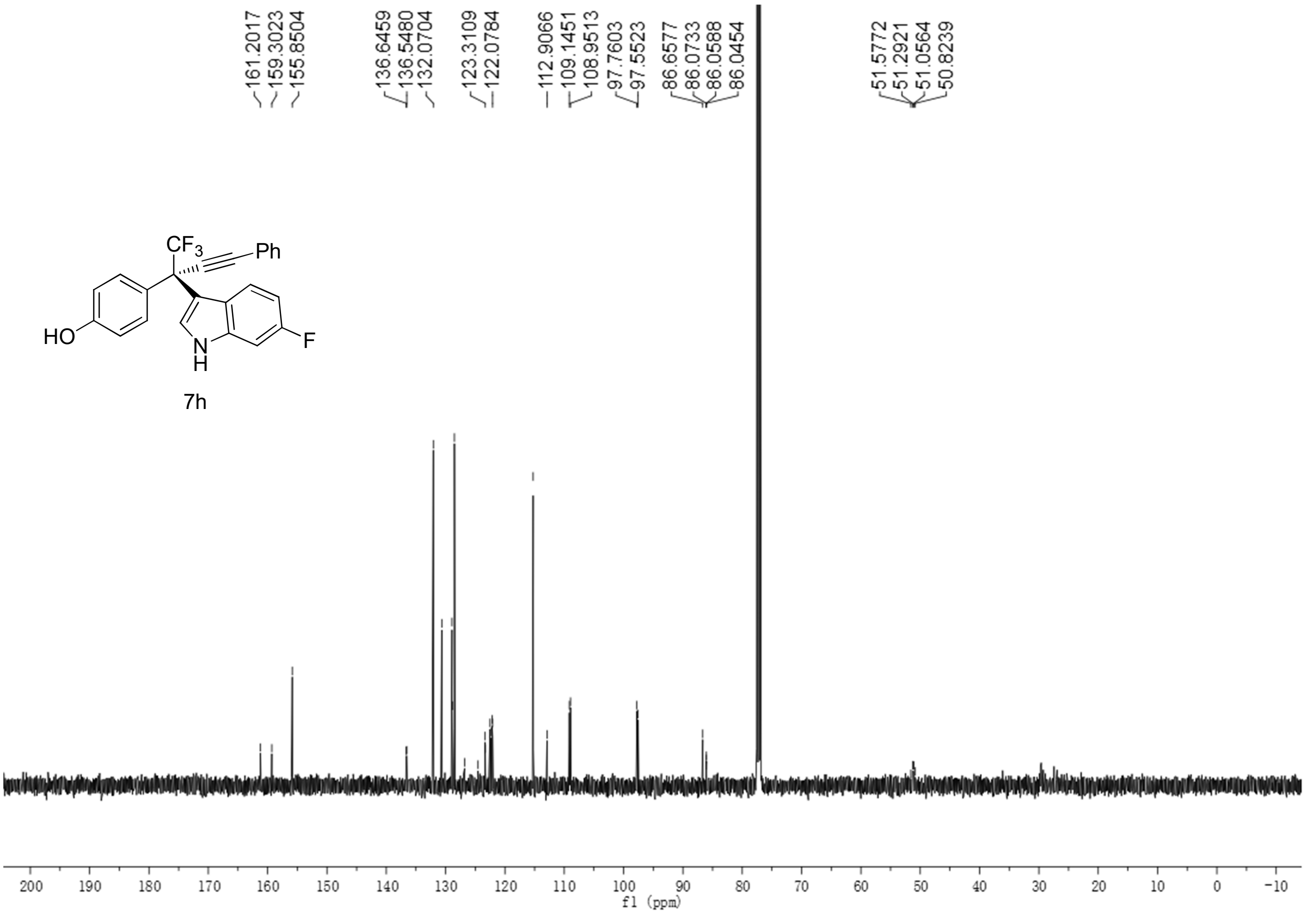

161.2017
159.3023
155.8504
136.6459
136.5480
132.0704
123.3109
122.0784
112.9066
109.1451
108.9513
97.7603
97.5523
86.6577
86.0733
86.0588
86.0454
51.5772
51.2921
51.0564
50.8239
CF3
Ph
HO
N
H
F
7h
200 190 180 170 160 150 140 130 120 110 100 90 80 70 60 50 40 30 20 10 0 -10
f1 (ppm)

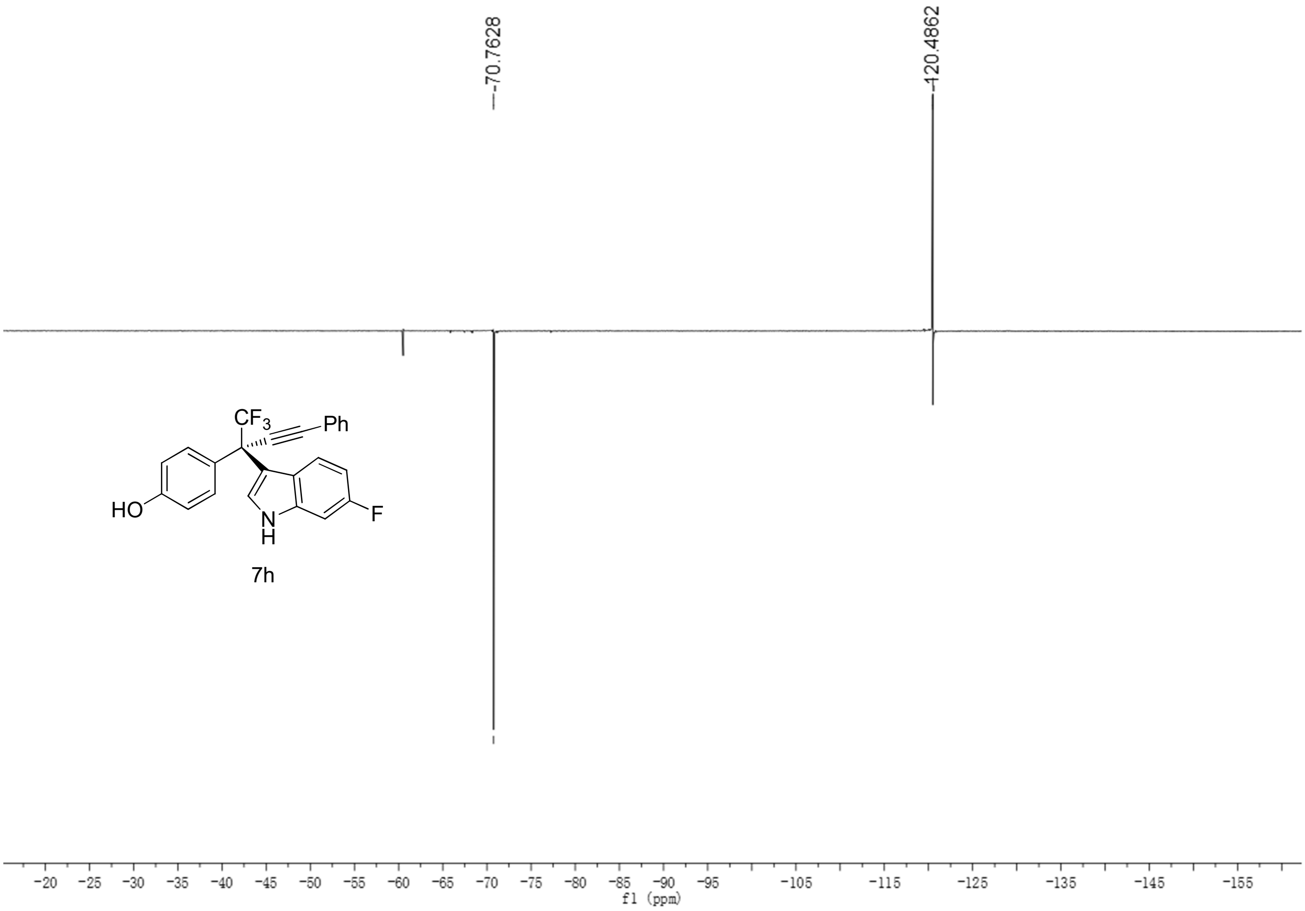

-70.7628
-120.4862
CF3
Ph
HO
N
H
F
7h
-20 -25 -30 -35 -40 -45 -50 -55 -60 -65 -70 -75 -80 -85 -90 -95 -105 -115 -125 -135 -145 -155
f1 (ppm)

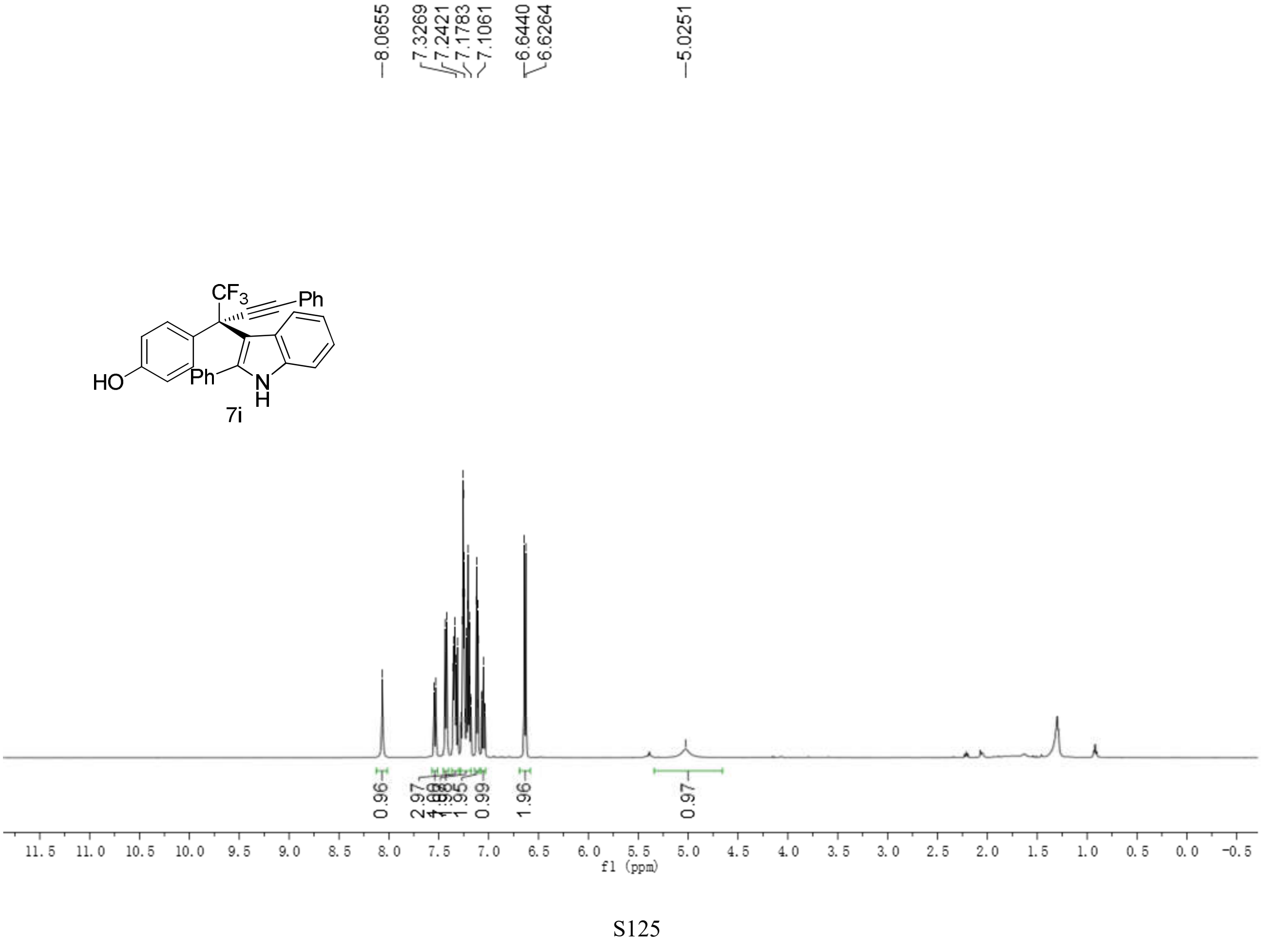

8.0655
7.3269
7.2421
7.1783
7.1061
6.6440
6.6264
5.0251
CF3
Ph
HO
Ph
N
H
7i
0.96
2.97
1.95
0.99
1.96
0.97
11.5 11.0 10.5 10.0 9.5 9.0 8.5 8.0 7.5 7.0 6.5 6.0 5.5 5.0 4.5 4.0 3.5 3.0 2.5 2.0 1.5 1.0 0.5 0.0 -0.5
f1 (ppm)

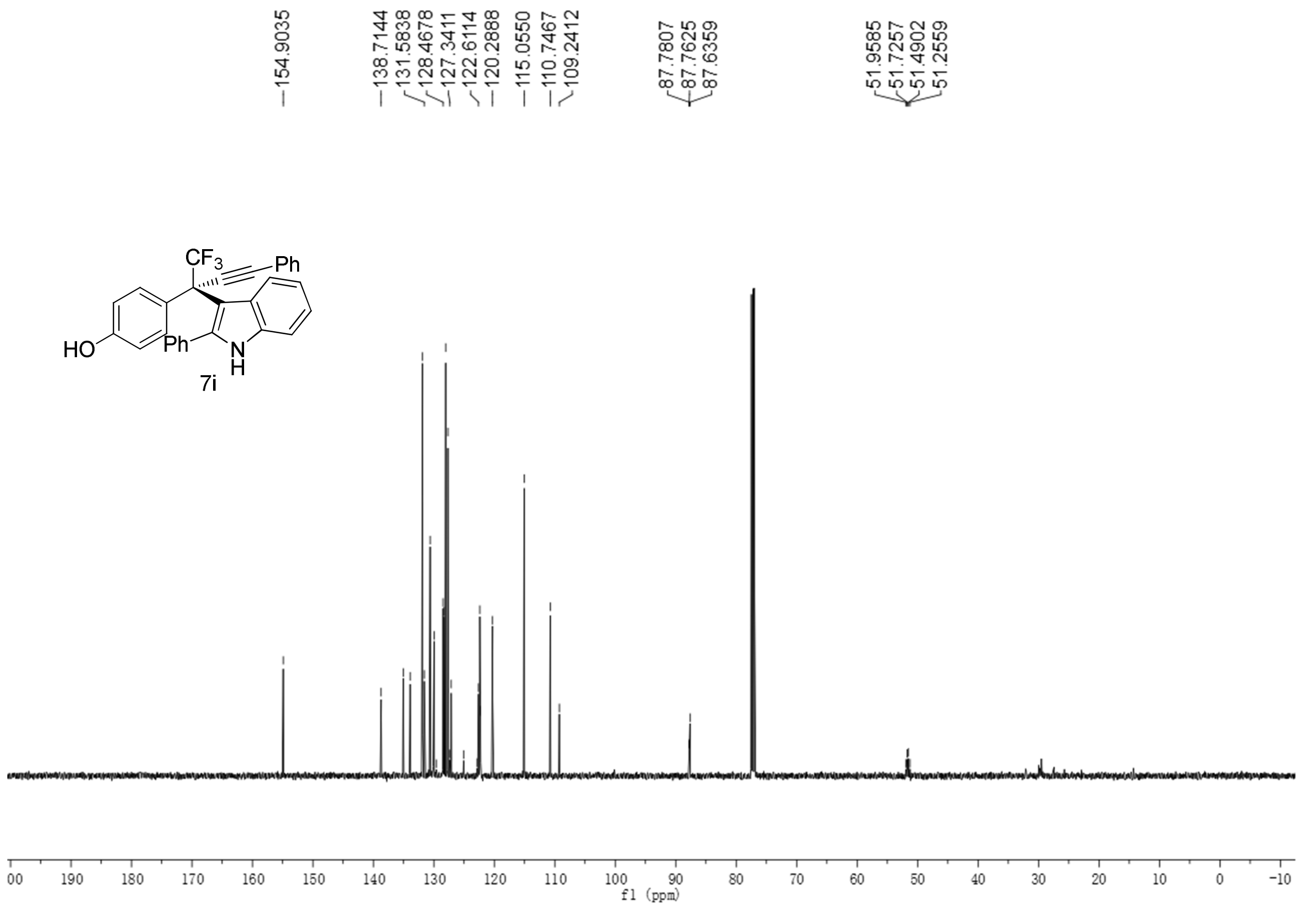
154.9035
138.7144
131.5838
128.4678
127.3411
122.6114
120.2888
115.0550
110.7467
109.2412
87.7807
87.7625
87.6359
51.9585
51.7257
51.4902
51.2559
CF3
Ph
HO
Ph
N
H
7i
f1 (ppm)

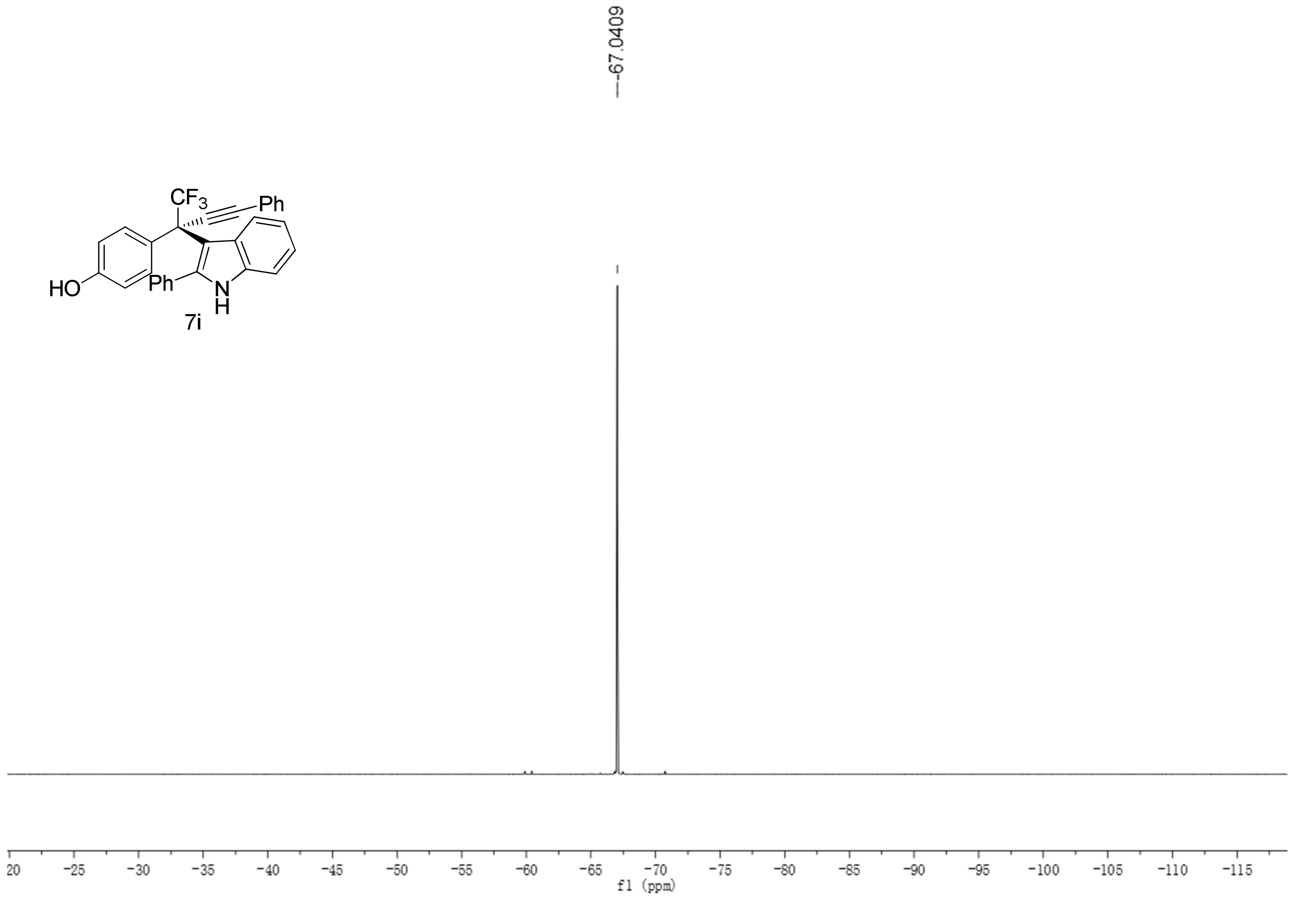
-67.0409
CF3
Ph
HO
Ph
N
H
7i
f1 (ppm)

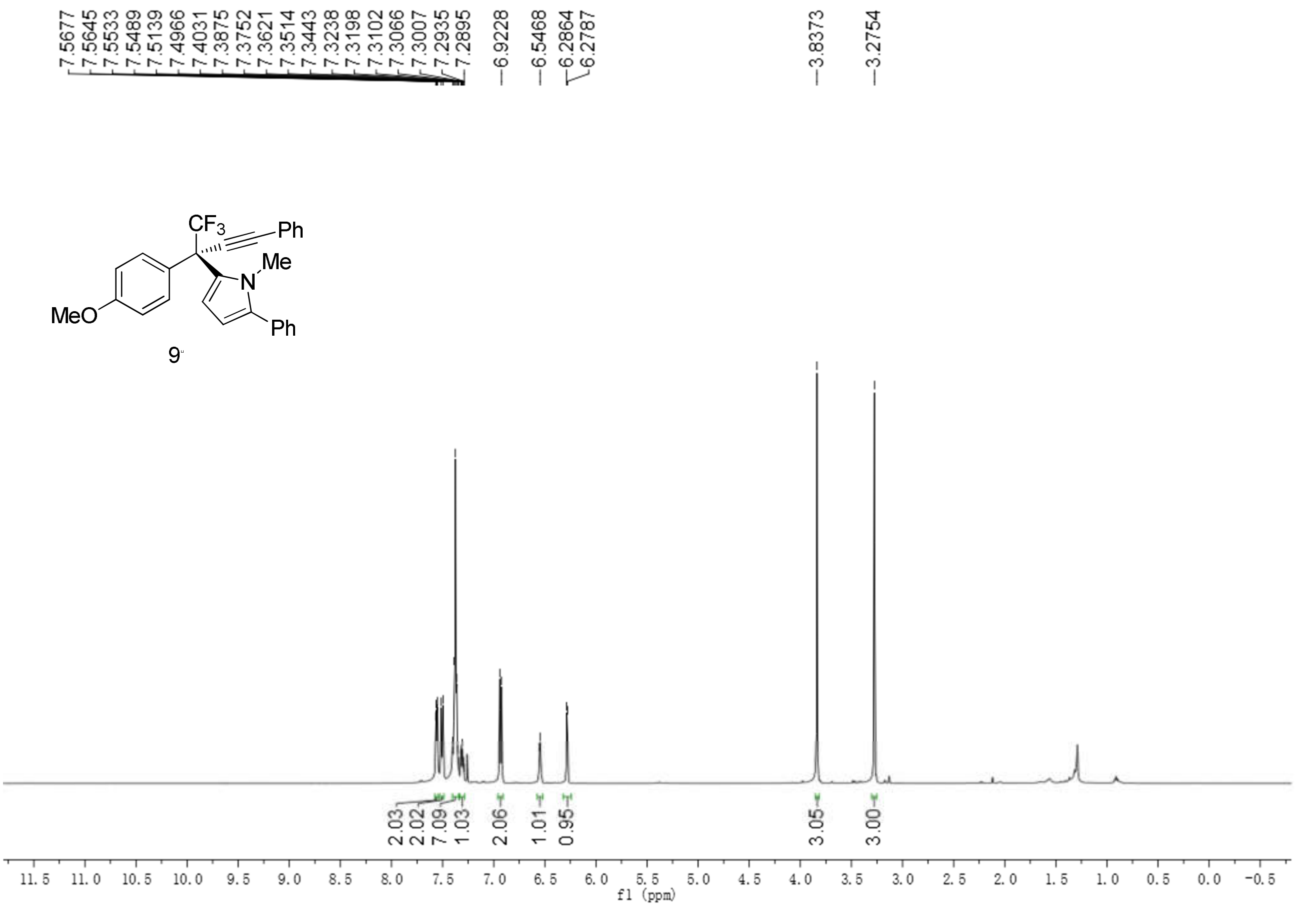
7.5677
7.5645
7.5533
7.5489
7.5139
7.4966
7.4031
7.3875
7.3752
7.3621
7.3514
7.3443
7.3238
7.3198
7.3102
7.3066
7.3007
7.2935
7.2895
6.9228
6.5468
6.2864
6.2787
3.8373
3.2754
CF3
Ph
Me
N
MeO
Ph
9
2.03
2.02
7.09
1.03
2.06
1.01
0.95
3.05
3.00
f1 (ppm)

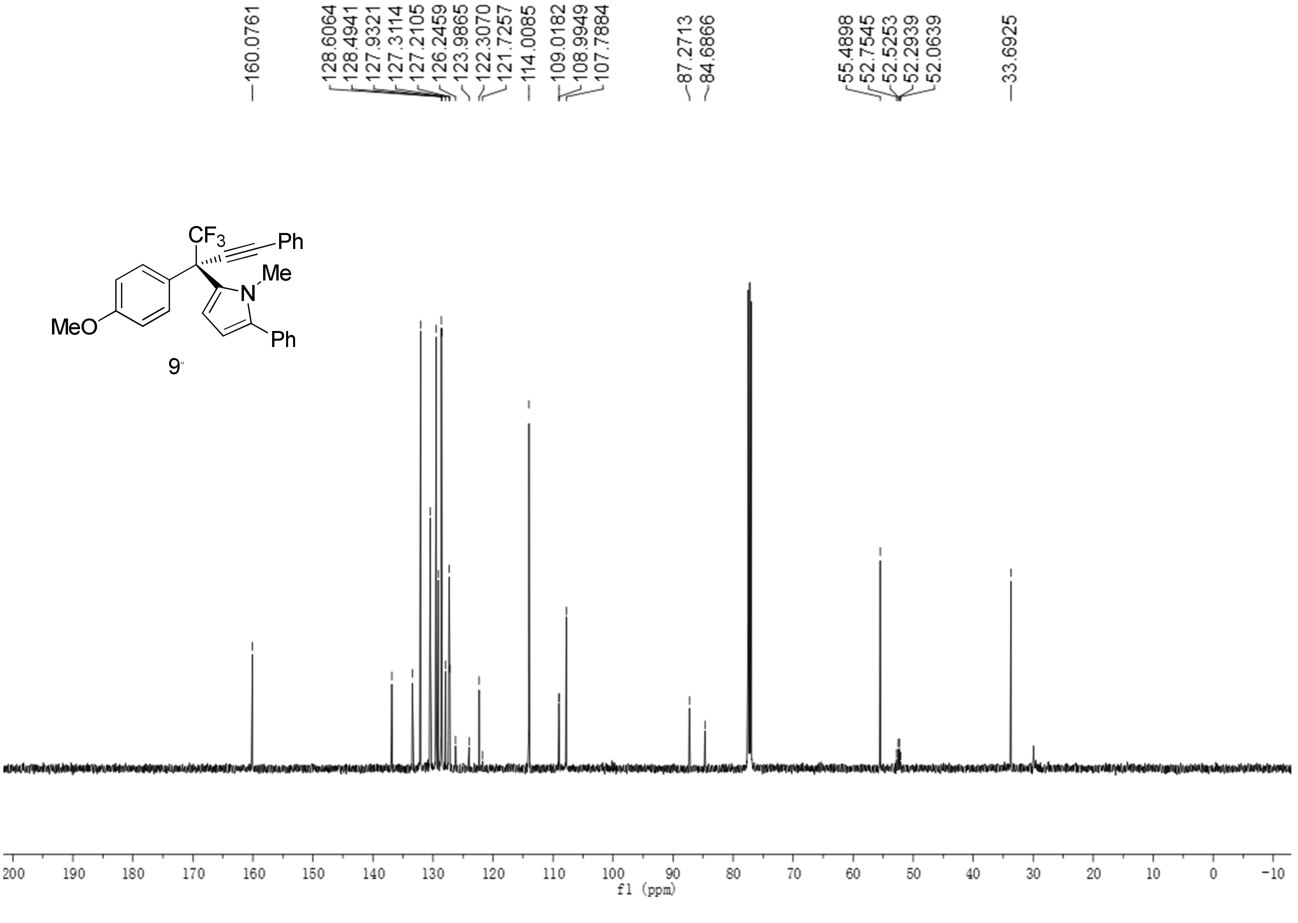
160.0761
128.6064
128.4941
127.9321
127.3114
127.2105
126.2459
123.9865
122.3070
121.7257
114.0085
109.0182
108.9949
107.7884
87.2713
84.6866
55.4898
52.7545
52.5253
52.2939
52.0639
33.6925
CF3
Ph
Me
N
MeO
Ph
9
f1 (ppm)

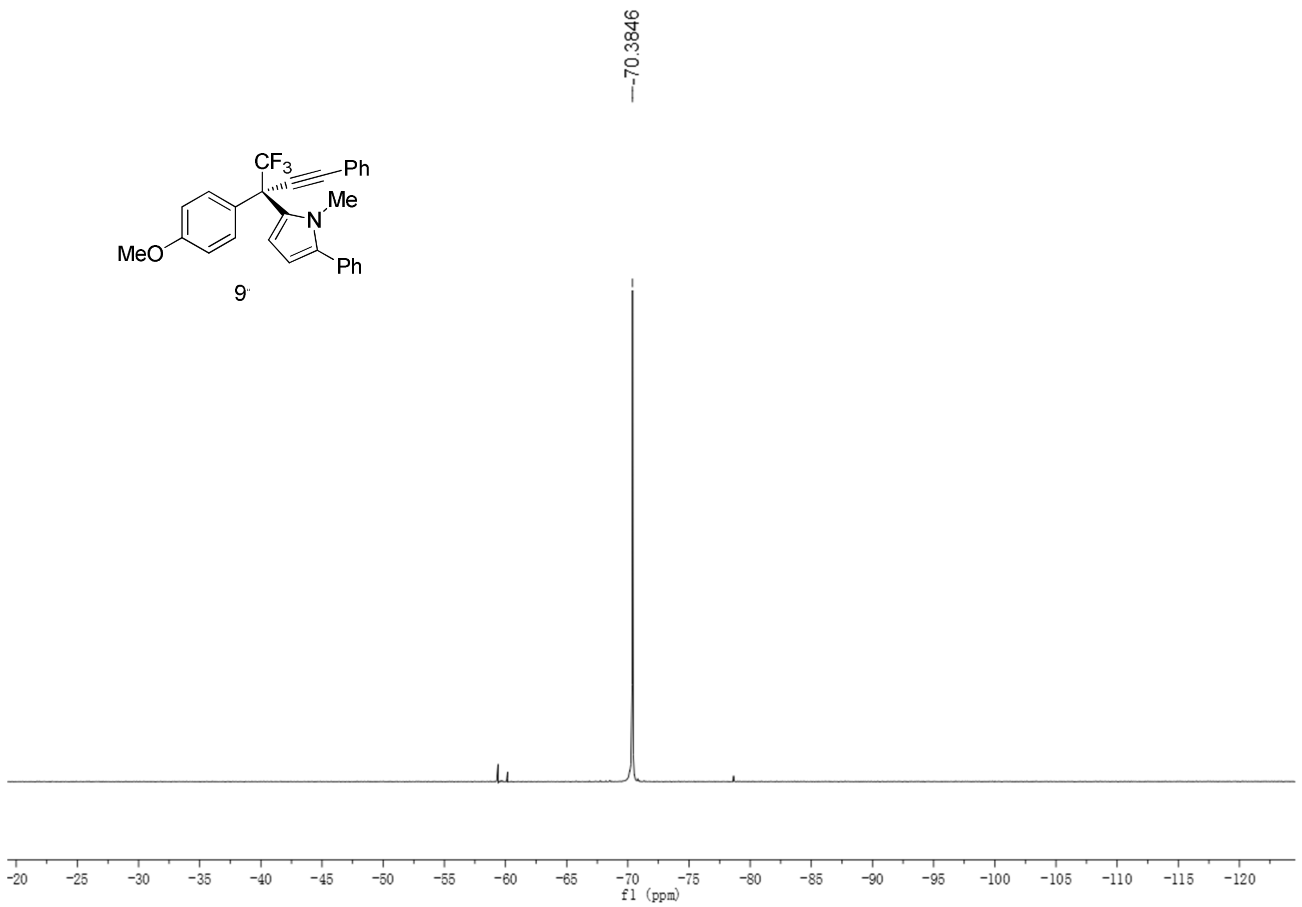

-70.3846
CF3
Ph
N
Me
MeO
Ph
9
-20 -25 -30 -35 -40 -45 -50 -55 -60 -65 -70 -75 -80 -85 -90 -95 -100 -105 -110 -115 -120
f1 (ppm)

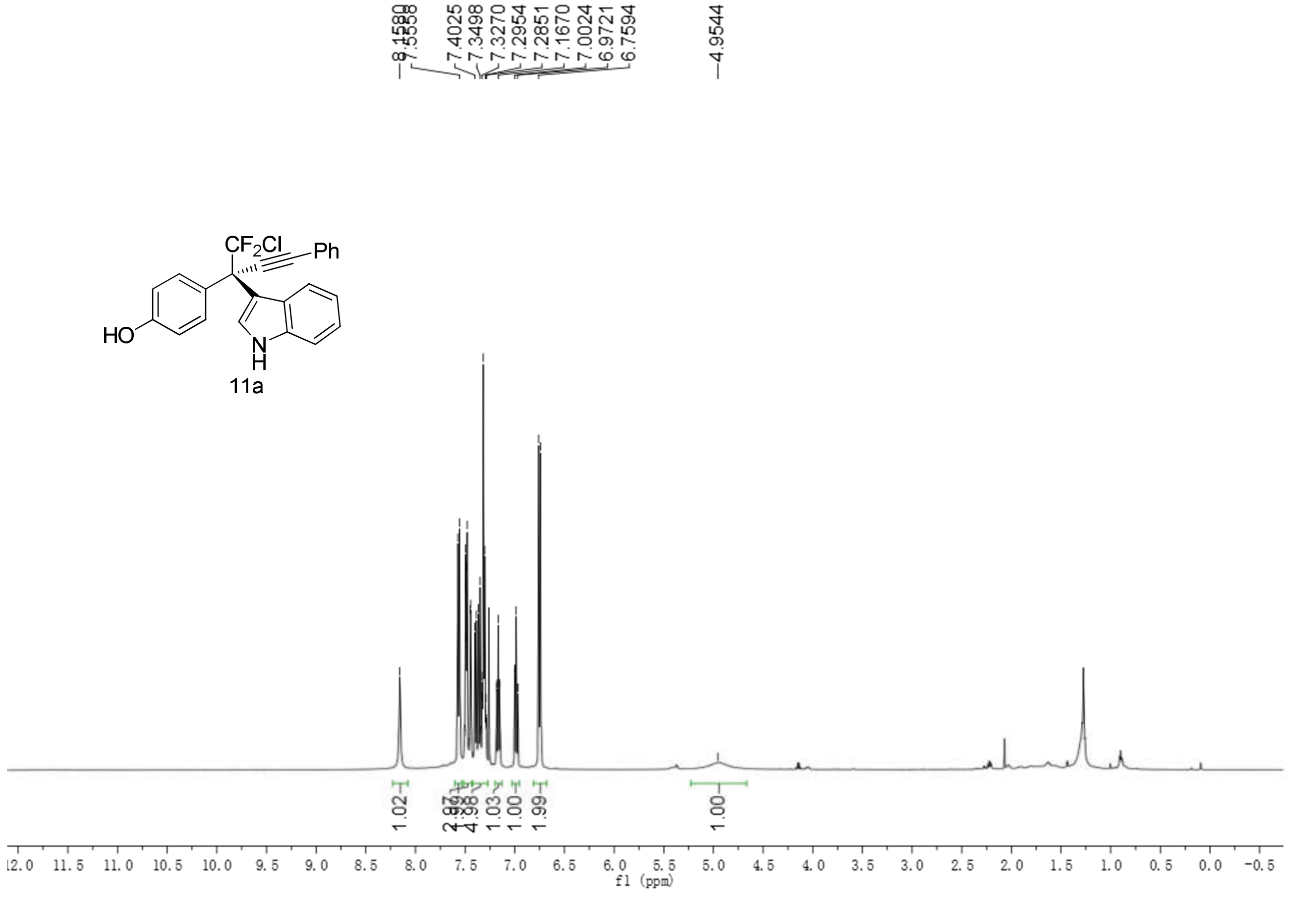

8.1580
7.4025
7.3498
7.3270
7.2954
7.2851
7.1670
7.0024
6.9721
6.7594
4.9544
CF2Cl
Ph
HO
N
H
11a
1.02
1.03
1.00
1.99
1.00
12.0 11.5 11.0 10.5 10.0 9.5 9.0 8.5 8.0 7.5 7.0 6.5 6.0 5.5 5.0 4.5 4.0 3.5 3.0 2.5 2.0 1.5 1.0 0.5 0.0 -0.5
f1 (ppm)

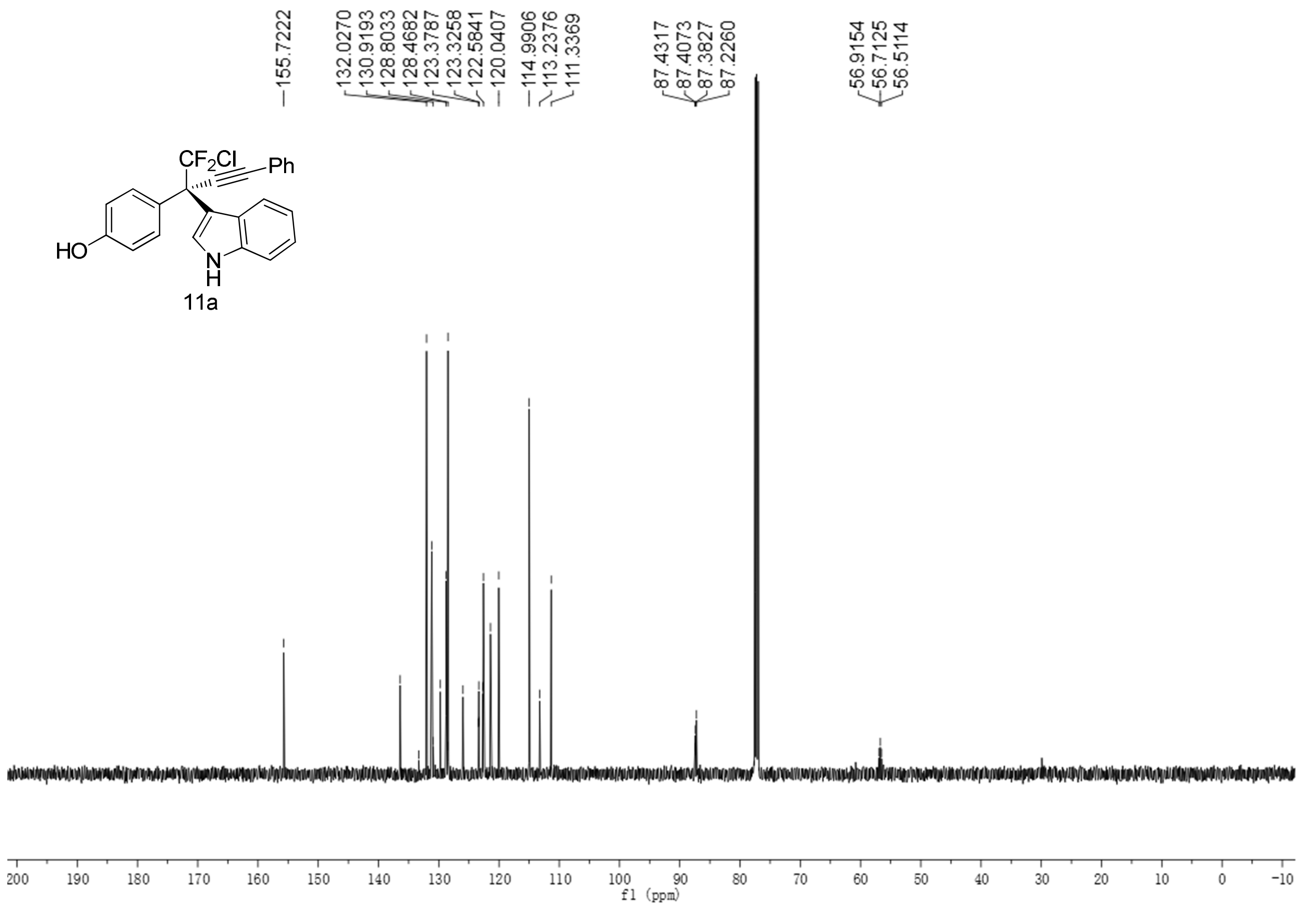
155.7222
132.0270
130.9193
128.8033
128.4682
123.3787
123.3258
122.5841
120.0407
114.9906
113.2376
111.3369
87.4317
87.4073
87.3827
87.2260
56.9154
56.7125
56.5114
CF2Cl
Ph
HO
N
H
11a
200 190 180 170 160 150 140 130 120 110 100 90 80 70 60 50 40 30 20 10 0 -10
f1 (ppm)

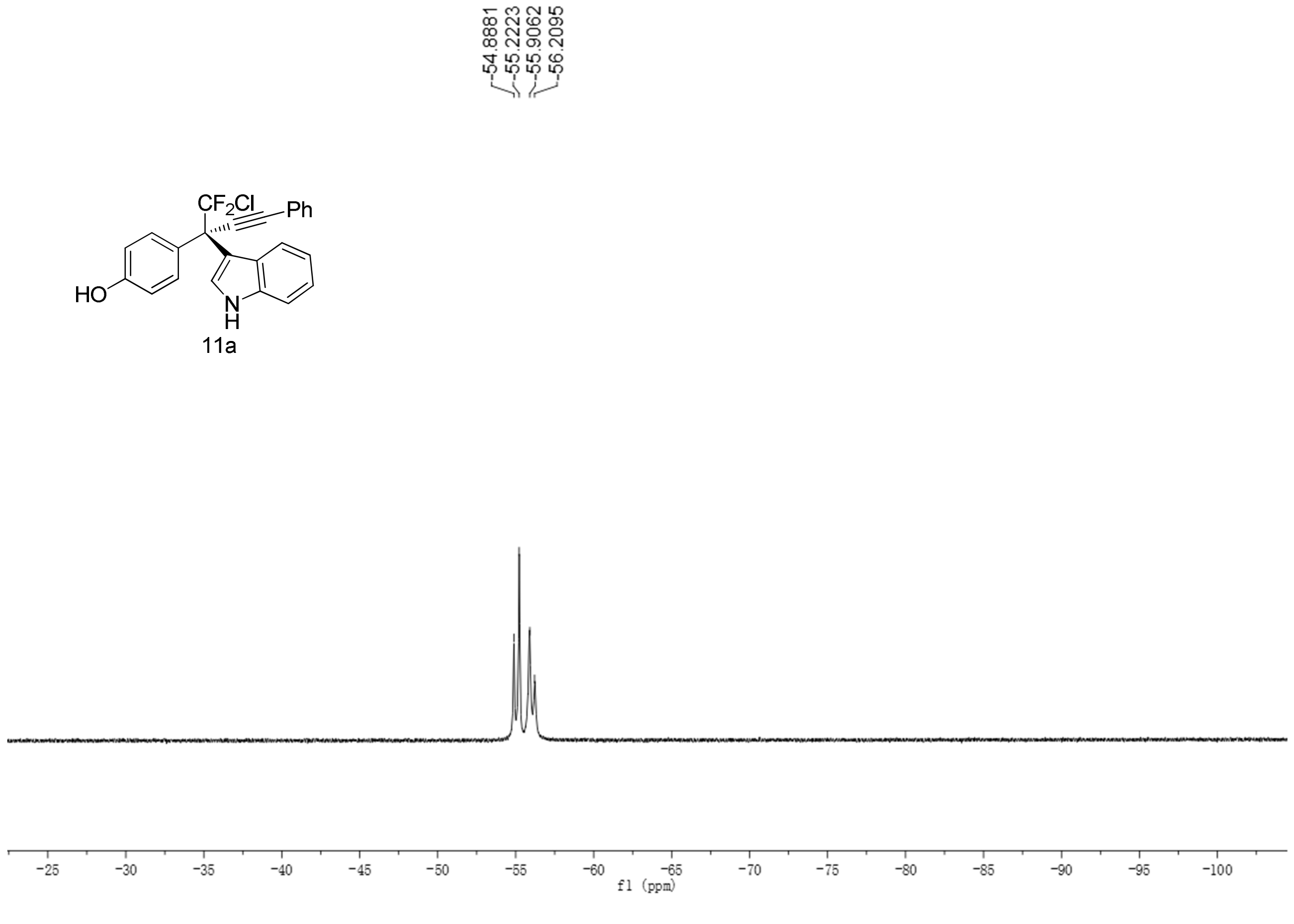
-54.8881
-55.2223
-55.9062
-56.2095
CF2Cl
Ph
HO
N
H
11a
-25 -30 -35 -40 -45 -50 -55 -60 -65 -70 -75 -80 -85 -90 -95 -100
f1 (ppm)

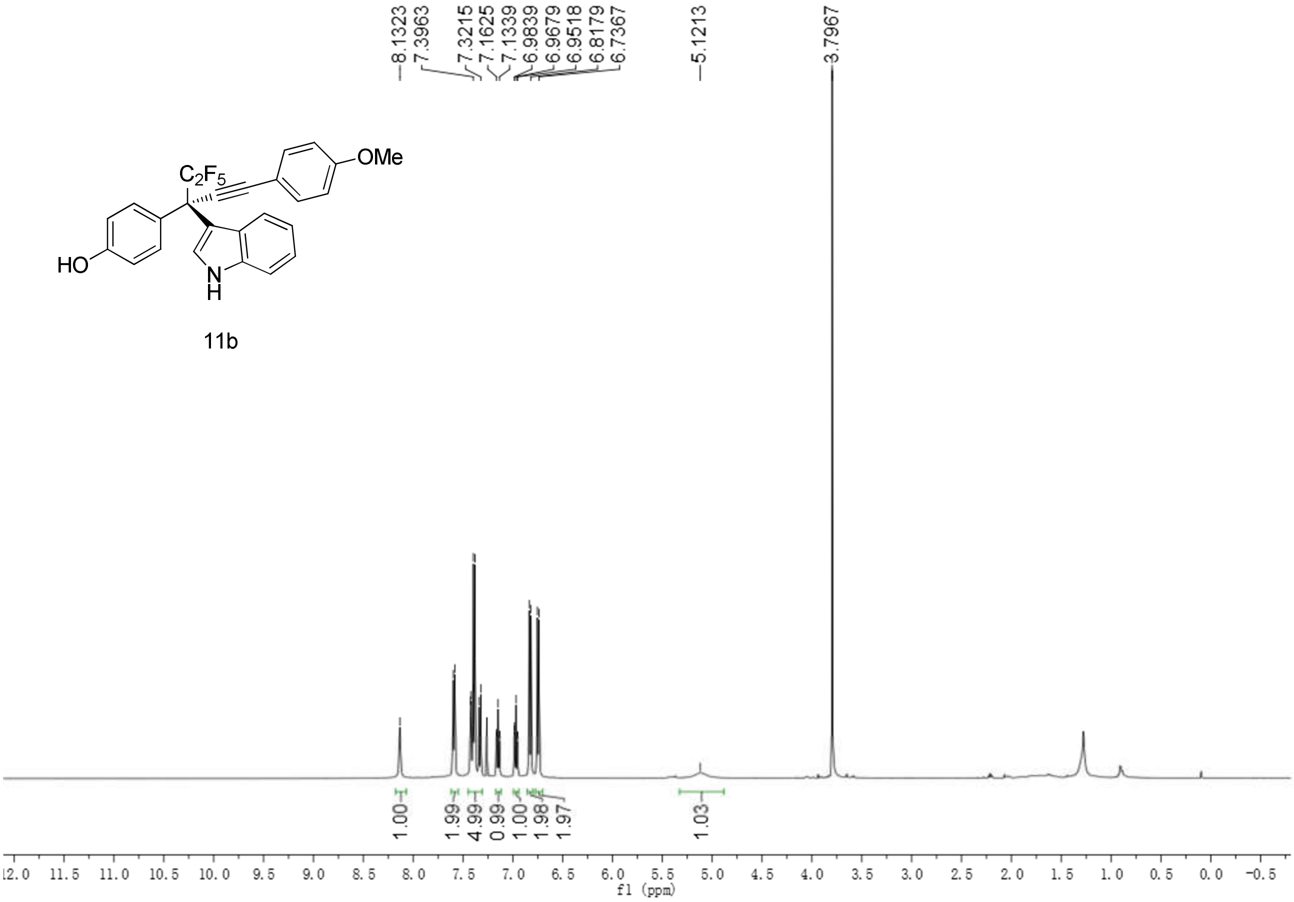

8.1323
7.3963
7.3215
7.1625
7.1339
6.9839
6.9679
6.9518
6.8179
6.7367
5.1213
3.7967
1.00
1.99
4.99
0.99
1.00
1.98
1.97
1.03
f1 (ppm)

11b

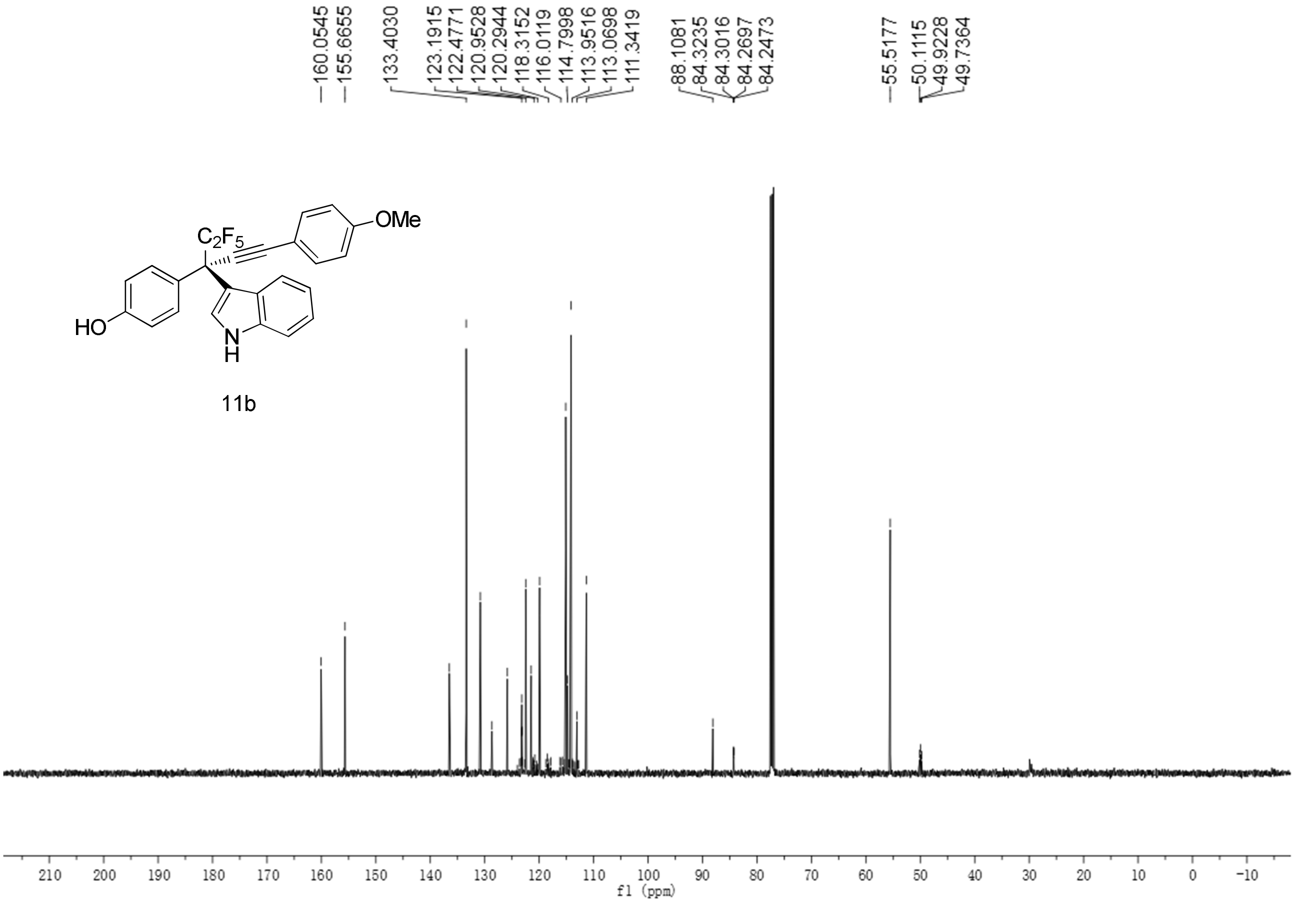

160.0545
155.6655
133.4030
123.1915
122.4771
120.9528
120.2944
118.3152
116.0119
114.7998
113.9516
113.0698
111.3419
88.1081
84.3235
84.3016
84.2697
84.2473
55.5177
50.1115
49.9228
49.7364
f1 (ppm)

11b

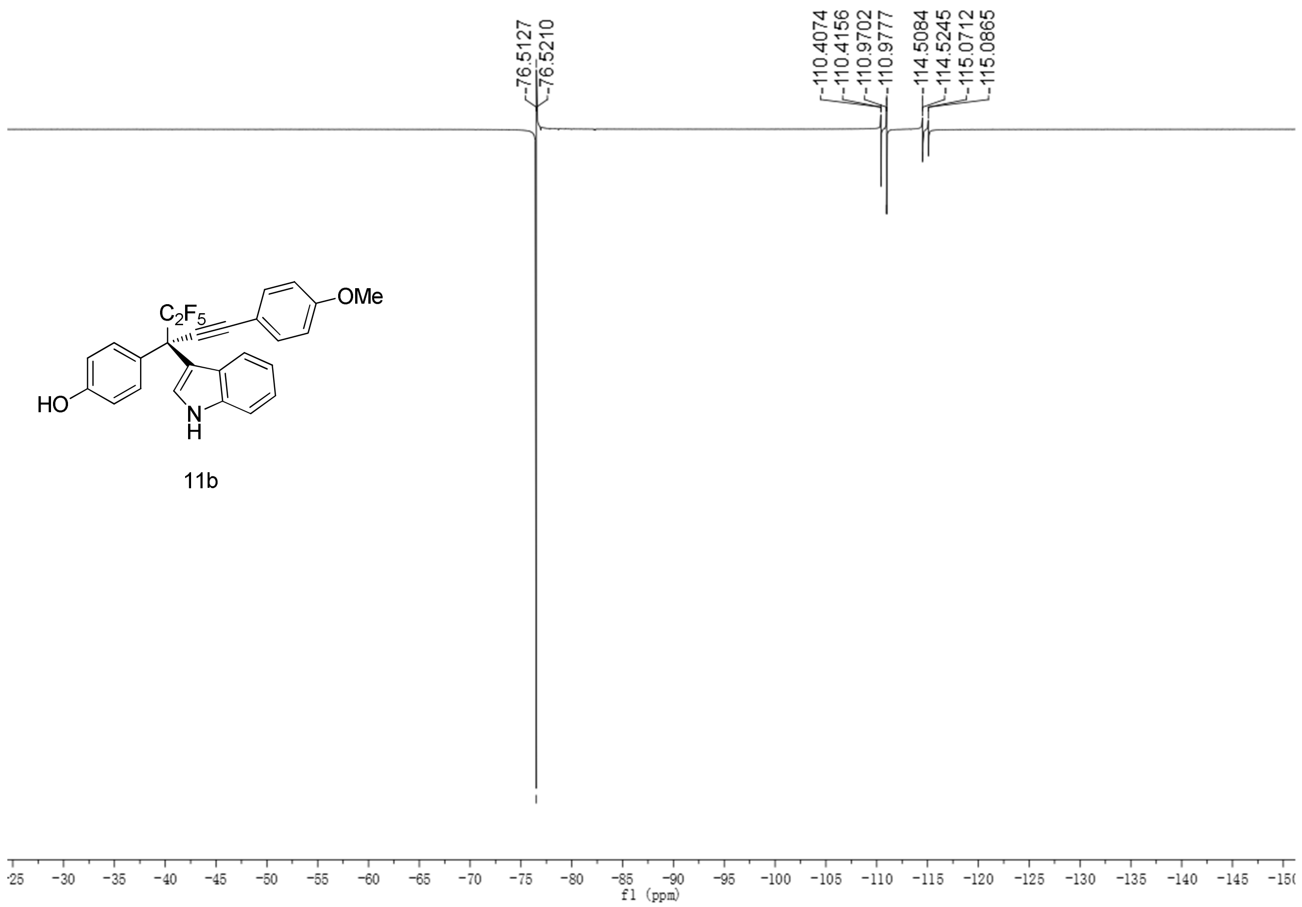

-76.5127
-76.5210
-110.4074
-110.4156
-110.9702
-110.9777
-114.5084
-114.5245
-115.0712
-115.0865
C2F5
OMe
HO
N
H
11b
f1 (ppm)

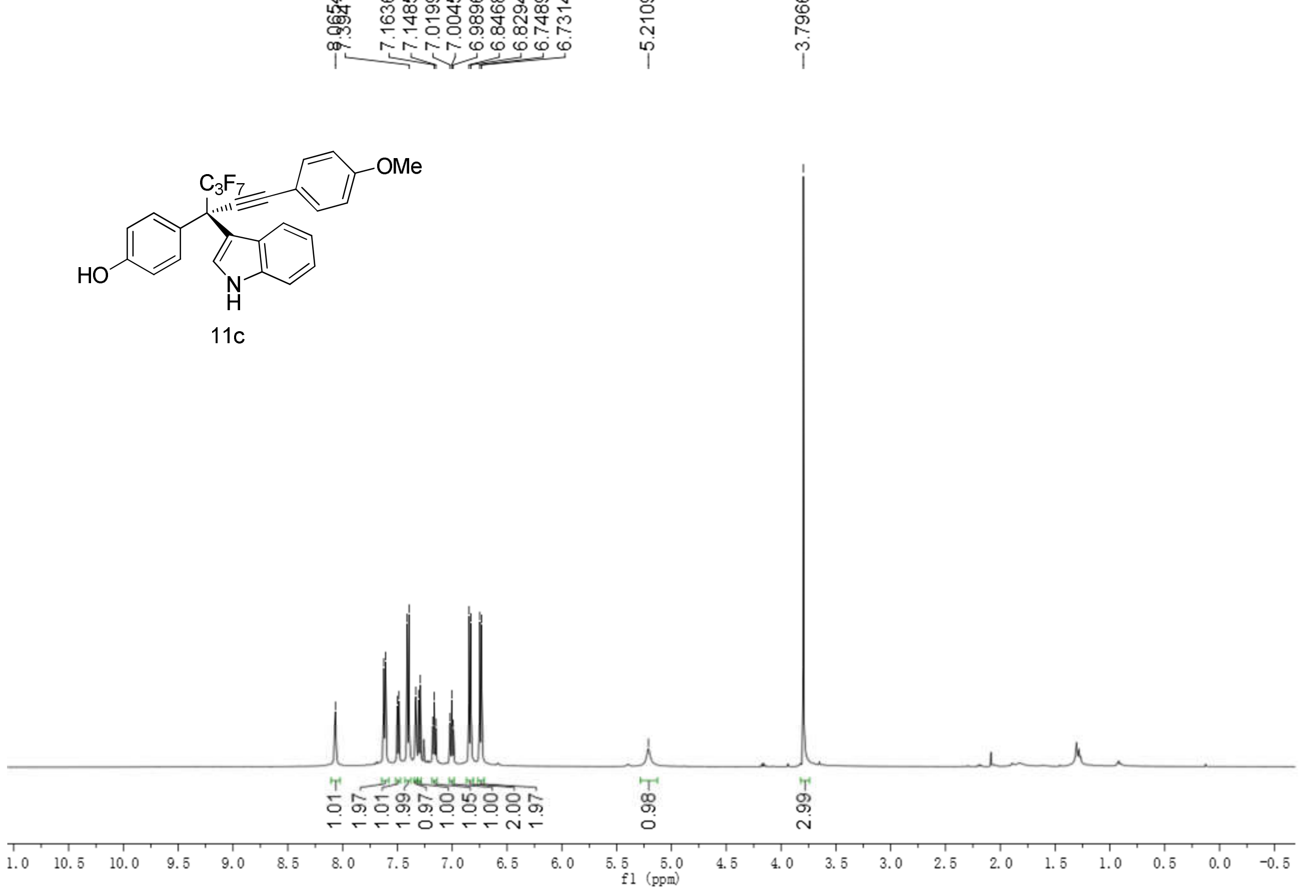

7.1636
7.1485
7.0199
7.0045
6.9896
6.8468
6.8294
6.7489
6.7314
5.2109
3.7966
C3F7
OMe
HO
N
H
11c
1.01
1.97
1.01
1.99
0.97
1.00
1.05
1.00
2.00
1.97
0.98
2.99
f1 (ppm)

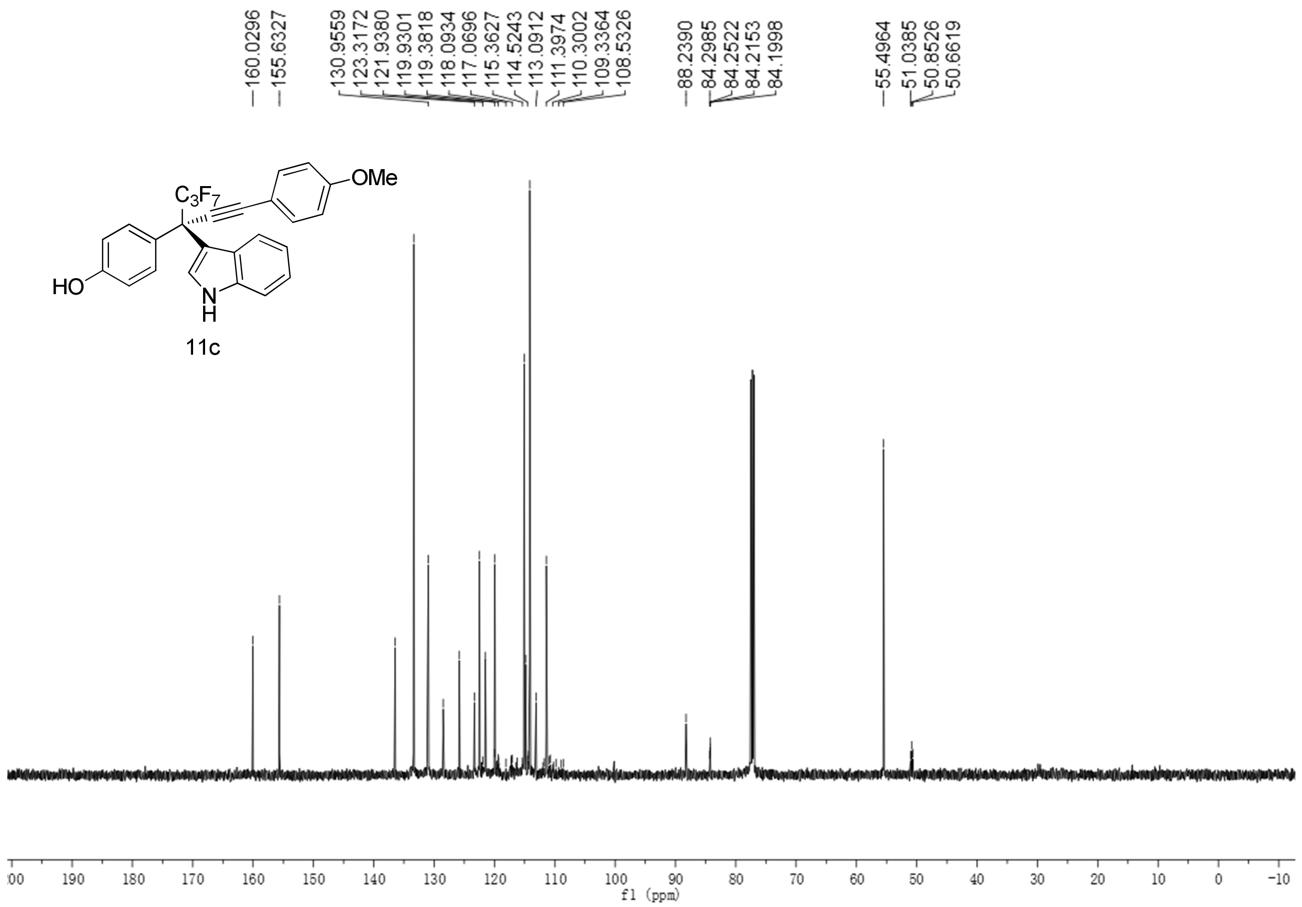
160.0296
155.6327
130.9559
123.3172
121.9380
119.9301
119.3818
118.0934
117.0696
115.3627
114.5243
113.0912
111.3974
110.3002
109.3364
108.5326
88.2390
84.2985
84.2522
84.2153
84.1998
55.4964
51.0385
50.8526
50.6619
C3F7
OMe
HO
N
H
11c
f1 (ppm)

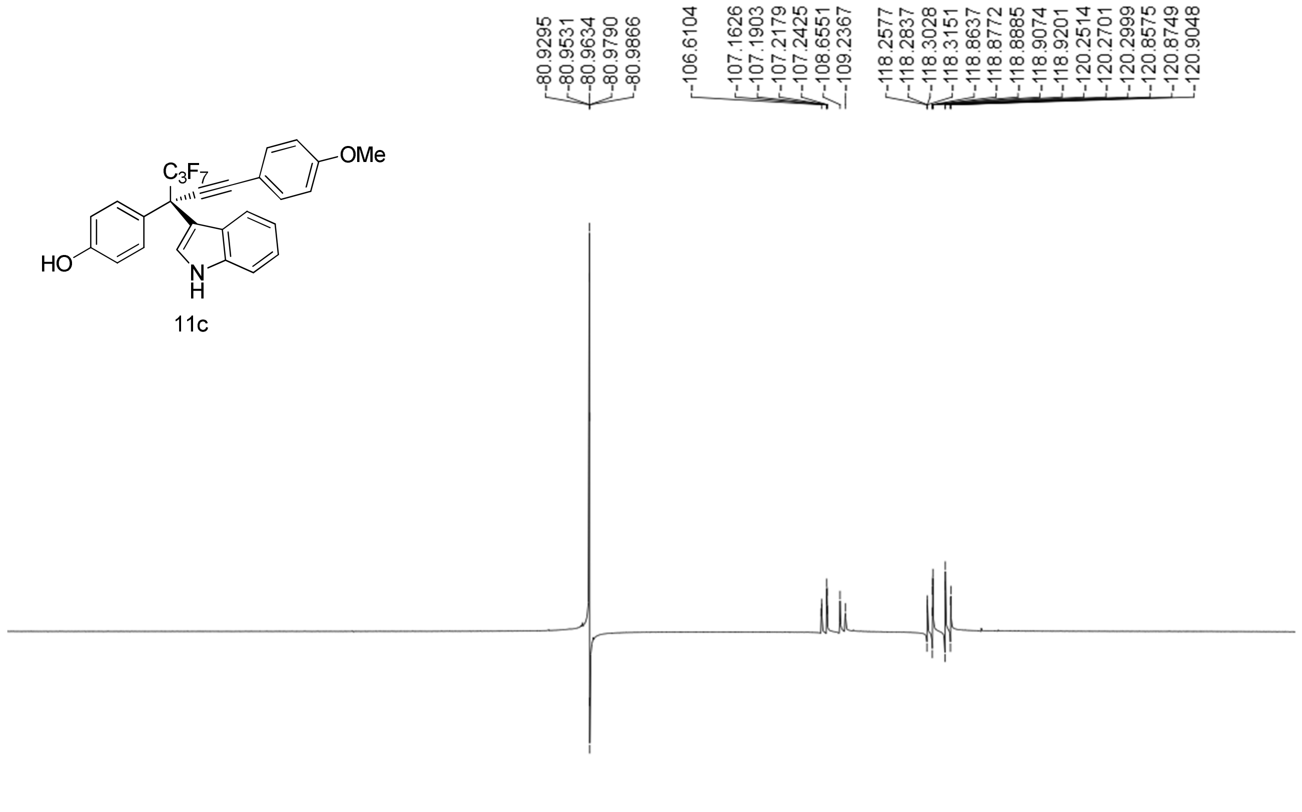
-80.9295
-80.9531
-80.9634
-80.9790
-80.9866
-106.6104
-107.1626
-107.1903
-107.2179
-107.2425
-108.6551
-109.2367
-118.2577
-118.2837
-118.3028
-118.3151
-118.8637
-118.8772
-118.8885
-118.9074
-118.9201
-120.2514
-120.2701
-120.2999
-120.8575
-120.8749
-120.9048
C3F7
OMe
HO
N
H
11c
f1 (ppm)

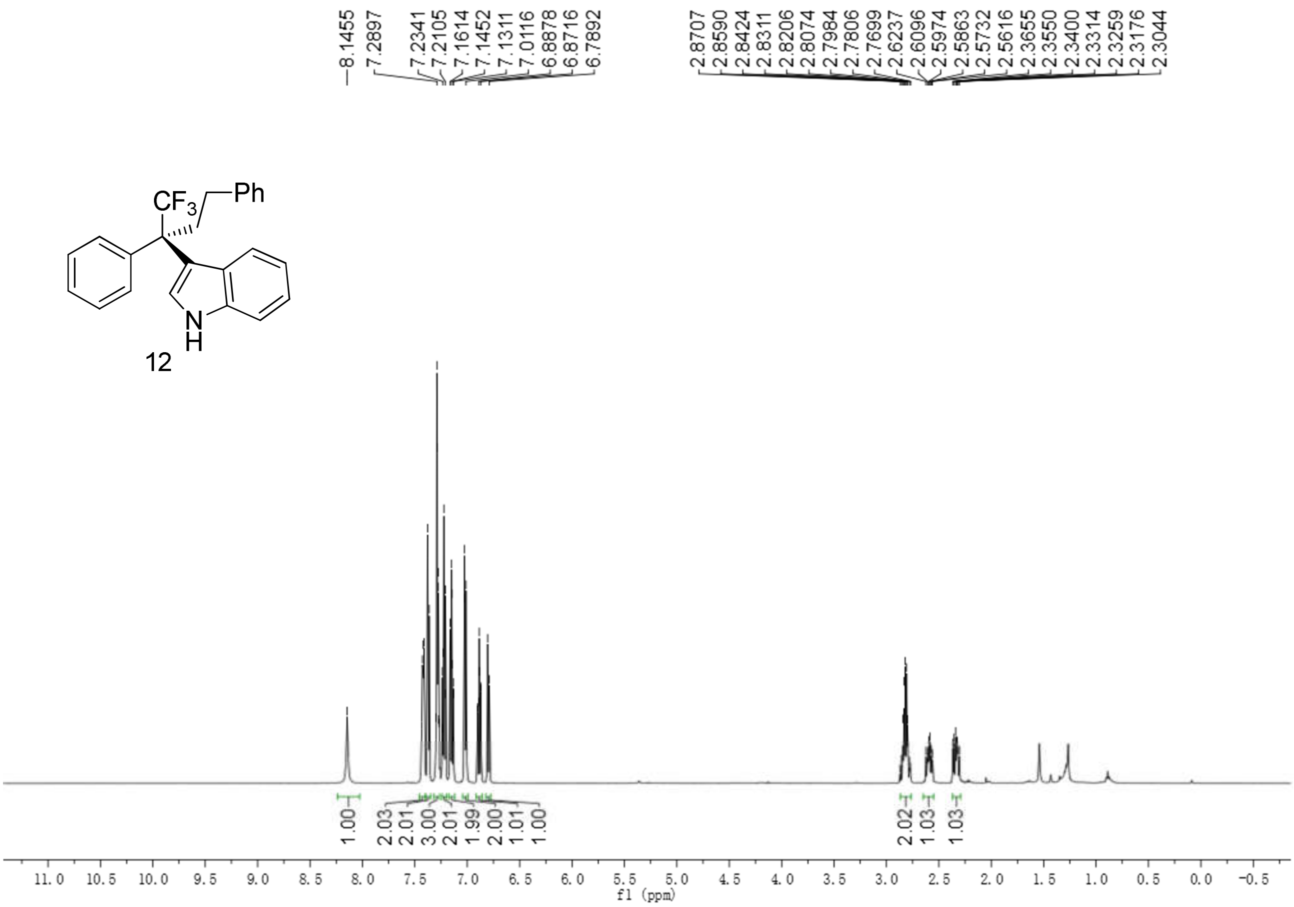
CF3
Ph
N
H
12
8.1455
7.2897
7.2341
7.2105
7.1614
7.1452
7.1311
7.0116
6.8878
6.8716
6.7892
2.8707
2.8590
2.8424
2.8311
2.8206
2.8074
2.7984
2.7806
2.7699
2.6237
2.6096
2.5974
2.5863
2.5732
2.5616
2.3655
2.3550
2.3400
2.3314
2.3259
2.3176
2.3044
1.00
2.03
2.01
3.00
2.01
1.99
2.00
1.01
1.00
2.02
1.03
1.03
11.0 10.5 10.0 9.5 9.0 8.5 8.0 7.5 7.0 6.5 6.0 5.5 5.0 4.5 4.0 3.5 3.0 2.5 2.0 1.5 1.0 0.5 0.0 -0.5
f1 (ppm)

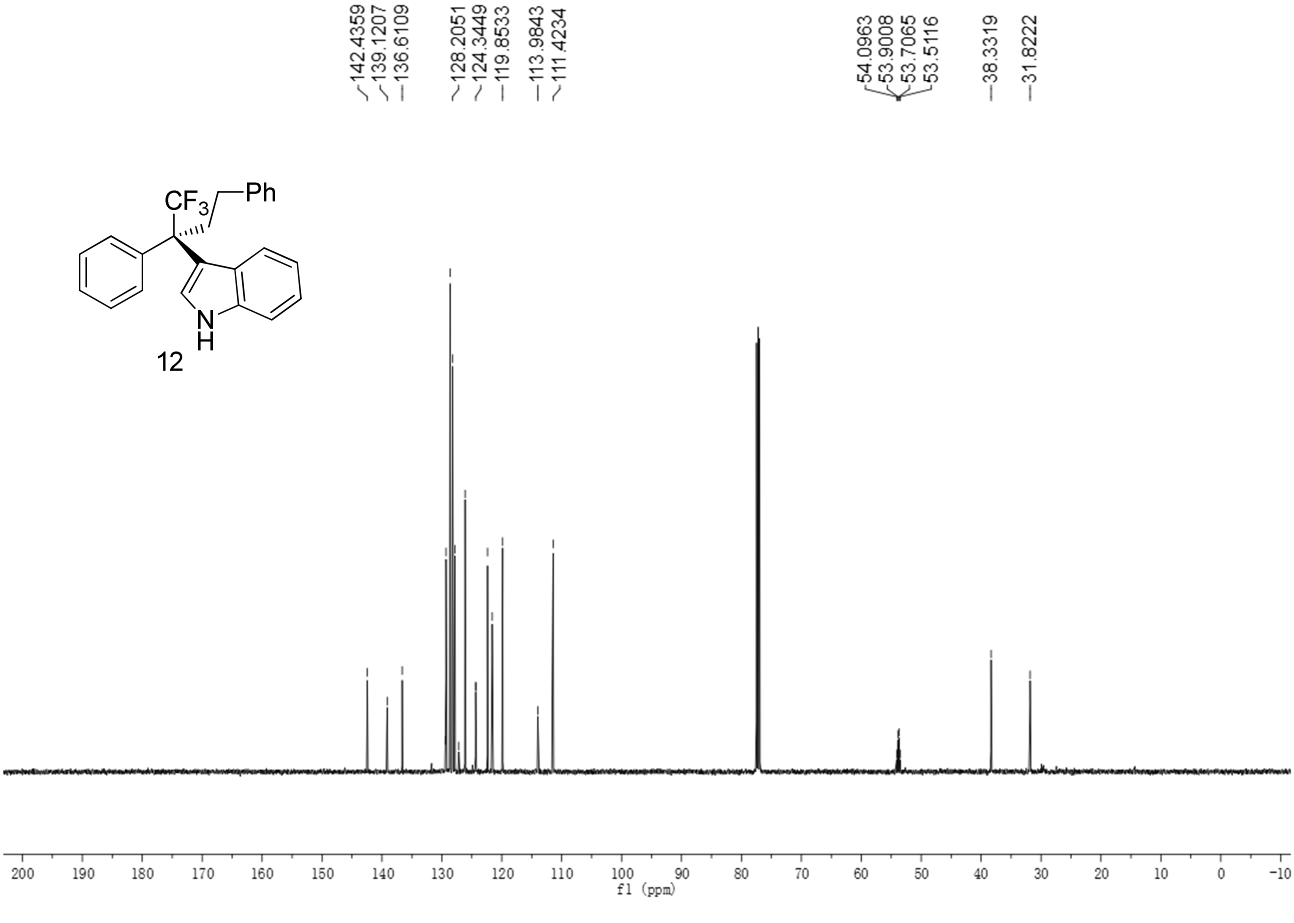
CF3
Ph
N
H
12
142.4359
139.1207
136.6109
128.2051
124.3449
119.8533
113.9843
111.4234
54.0963
53.9008
53.7065
53.5116
38.3319
31.8222
200 190 180 170 160 150 140 130 120 110 100 90 80 70 60 50 40 30 20 10 0 -10
f1 (ppm)

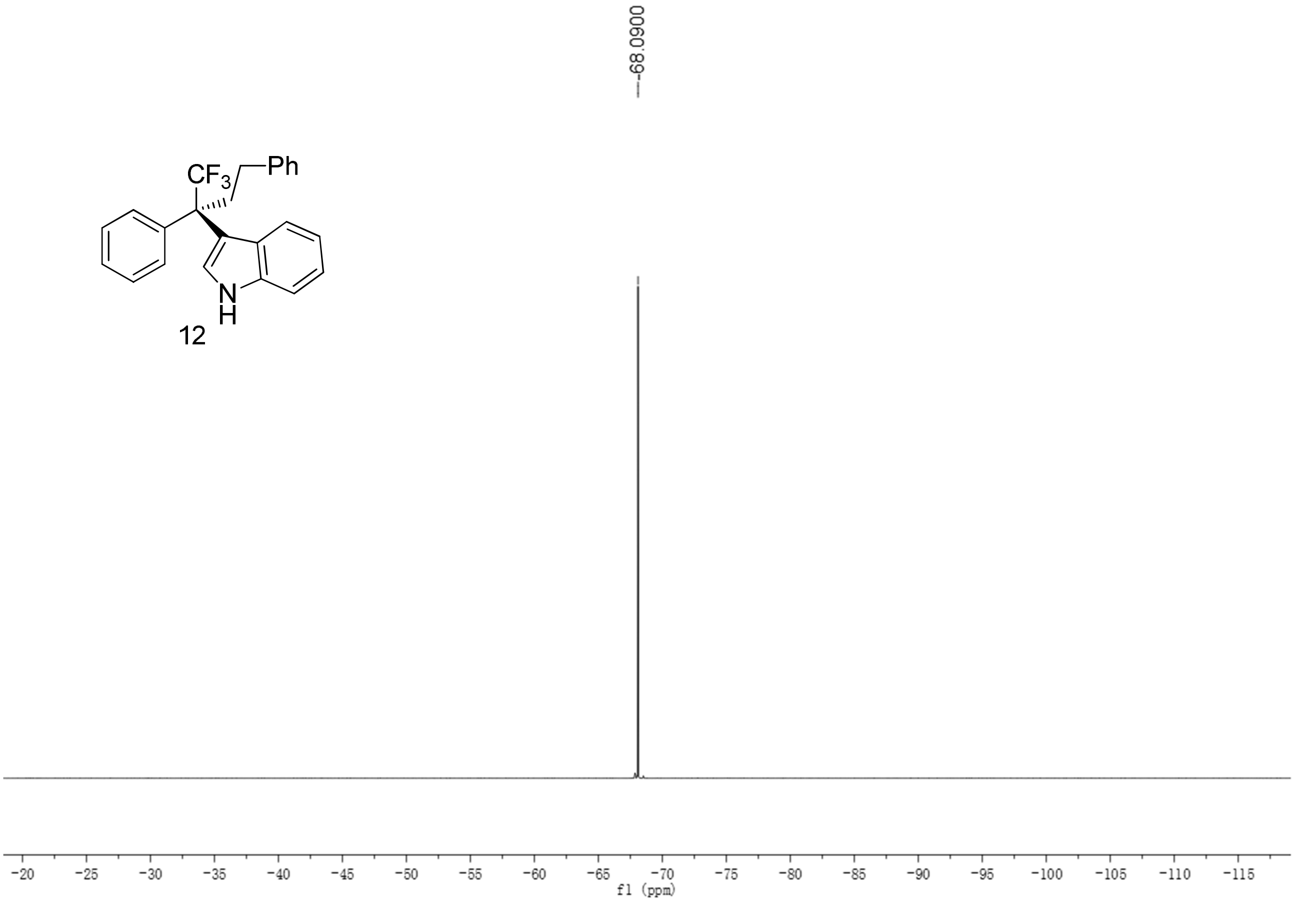

-68.0900
CF3
Ph
N
H
f1 (ppm)

12

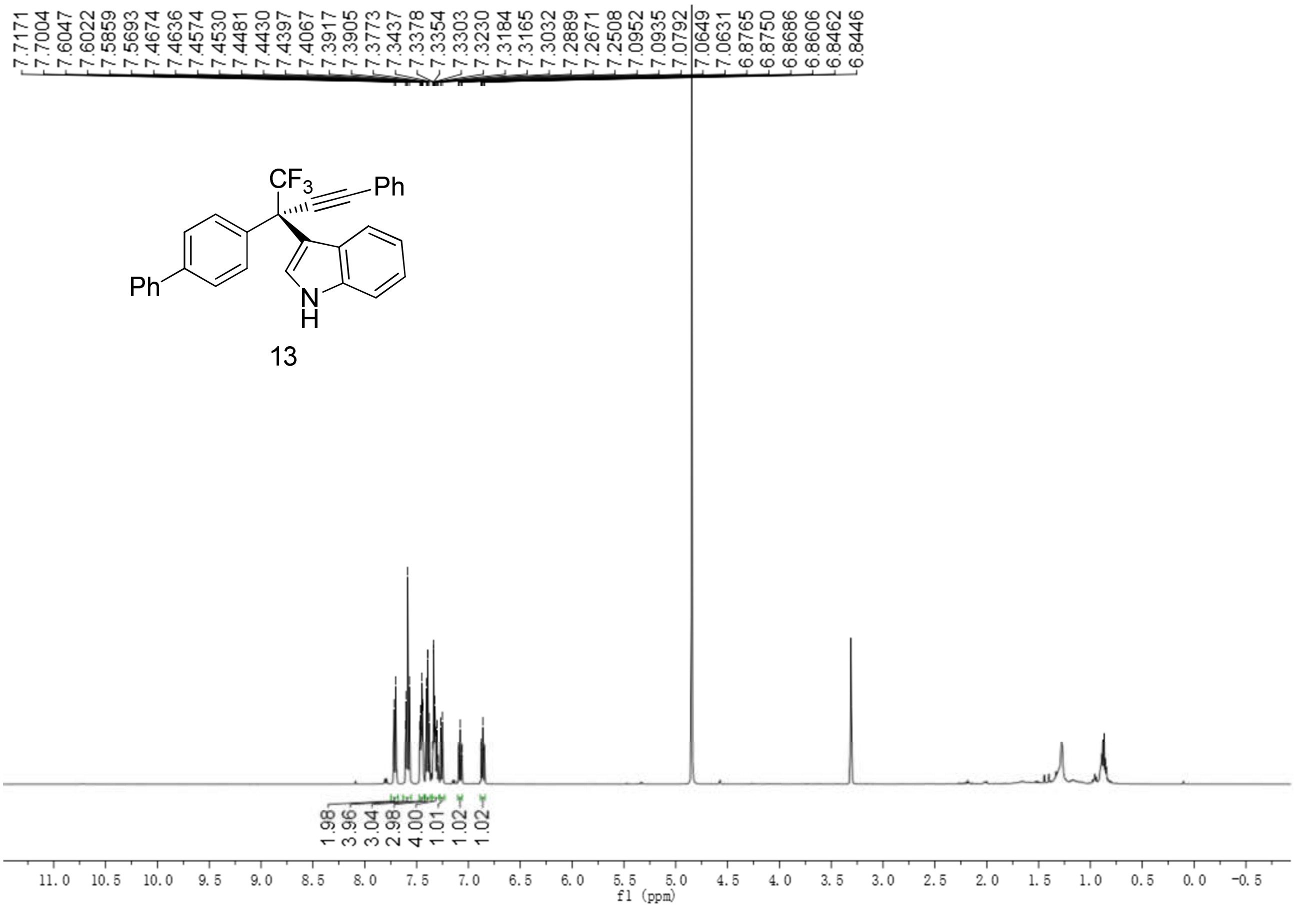

7.7171
7.7004
7.6047
7.6022
7.5859
7.5693
7.4674
7.4636
7.4574
7.4530
7.4481
7.4430
7.4397
7.4067
7.3917
7.3905
7.3773
7.3437
7.3378
7.3354
7.3303
7.3230
7.3184
7.3165
7.3032
7.2889
7.2671
7.2508
7.0952
7.0935
7.0792
7.0649
7.0631
6.8765
6.8750
6.8686
6.8606
6.8462
6.8446
CF3
Ph
Ph
N
H
1.98
3.96
3.04
2.98
4.00
1.01
1.02
1.02
f1 (ppm)

13

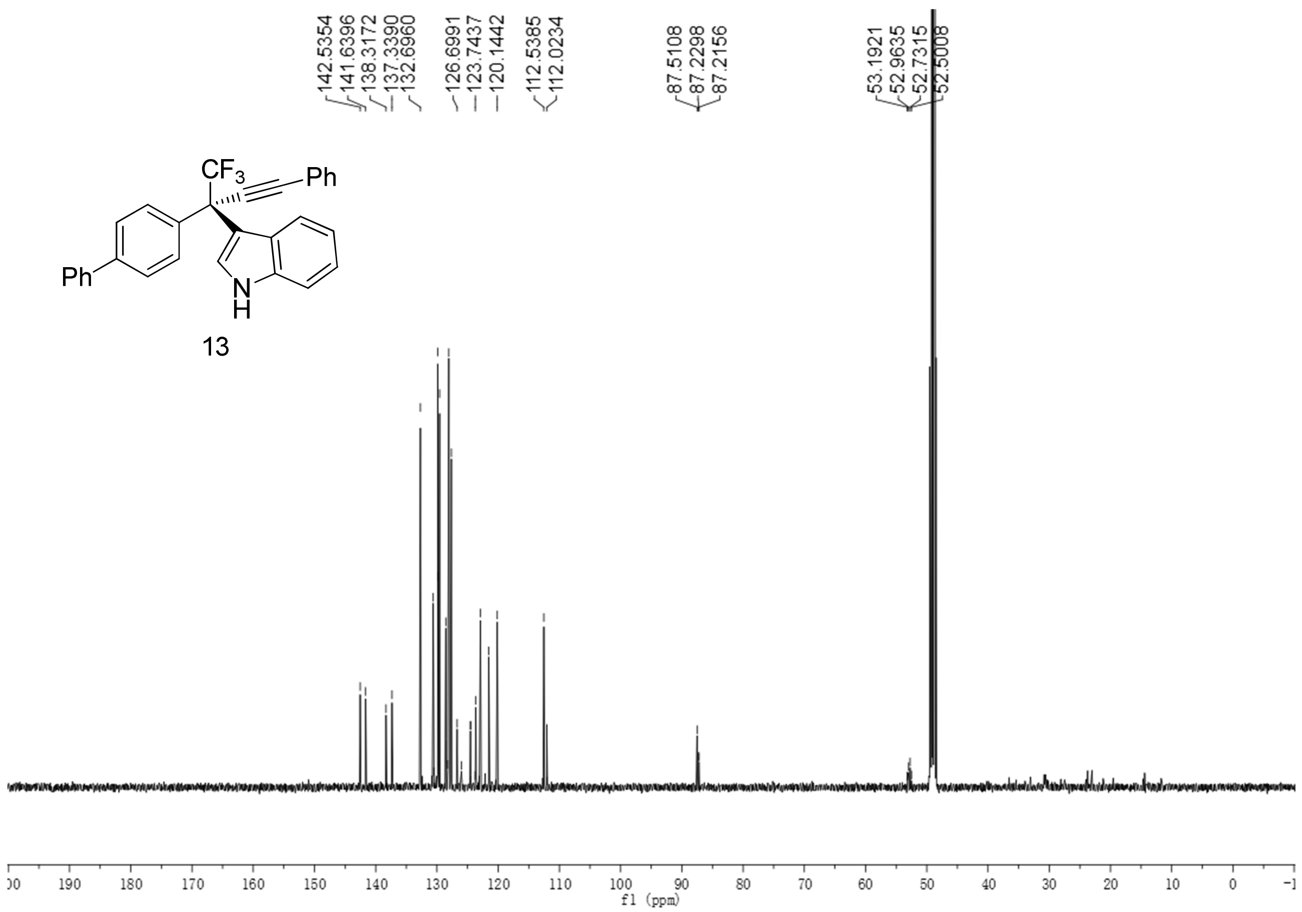
142.5354
141.6396
138.3172
137.3390
132.6960
126.6991
123.7437
120.1442
112.5385
112.0234
87.5108
87.2298
87.2156
53.1921
52.9635
52.7315
52.5008
CF3
Ph
Ph
NH
13
f1 (ppm)

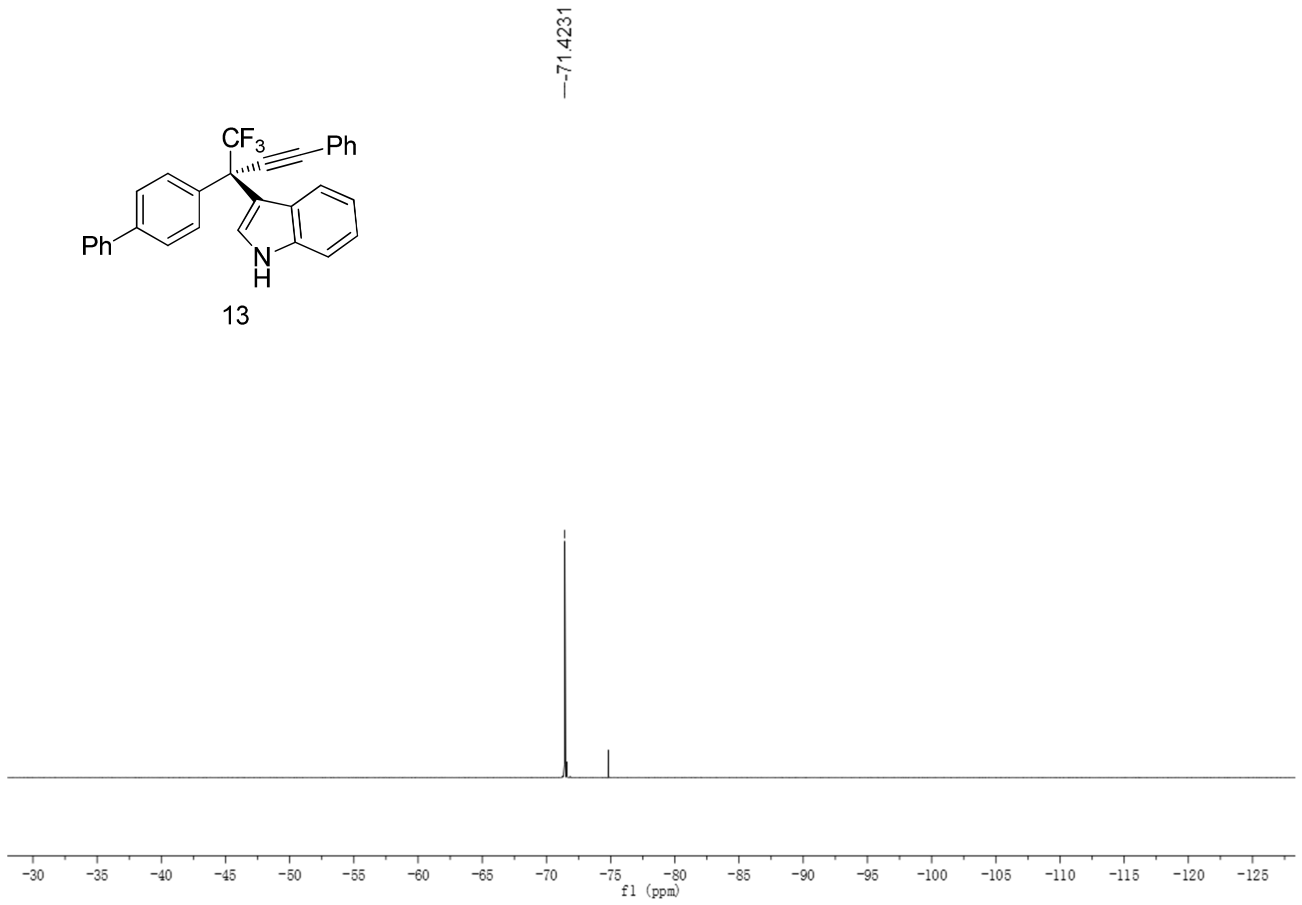
-71.4231
CF3
Ph
Ph
NH
13
f1 (ppm)

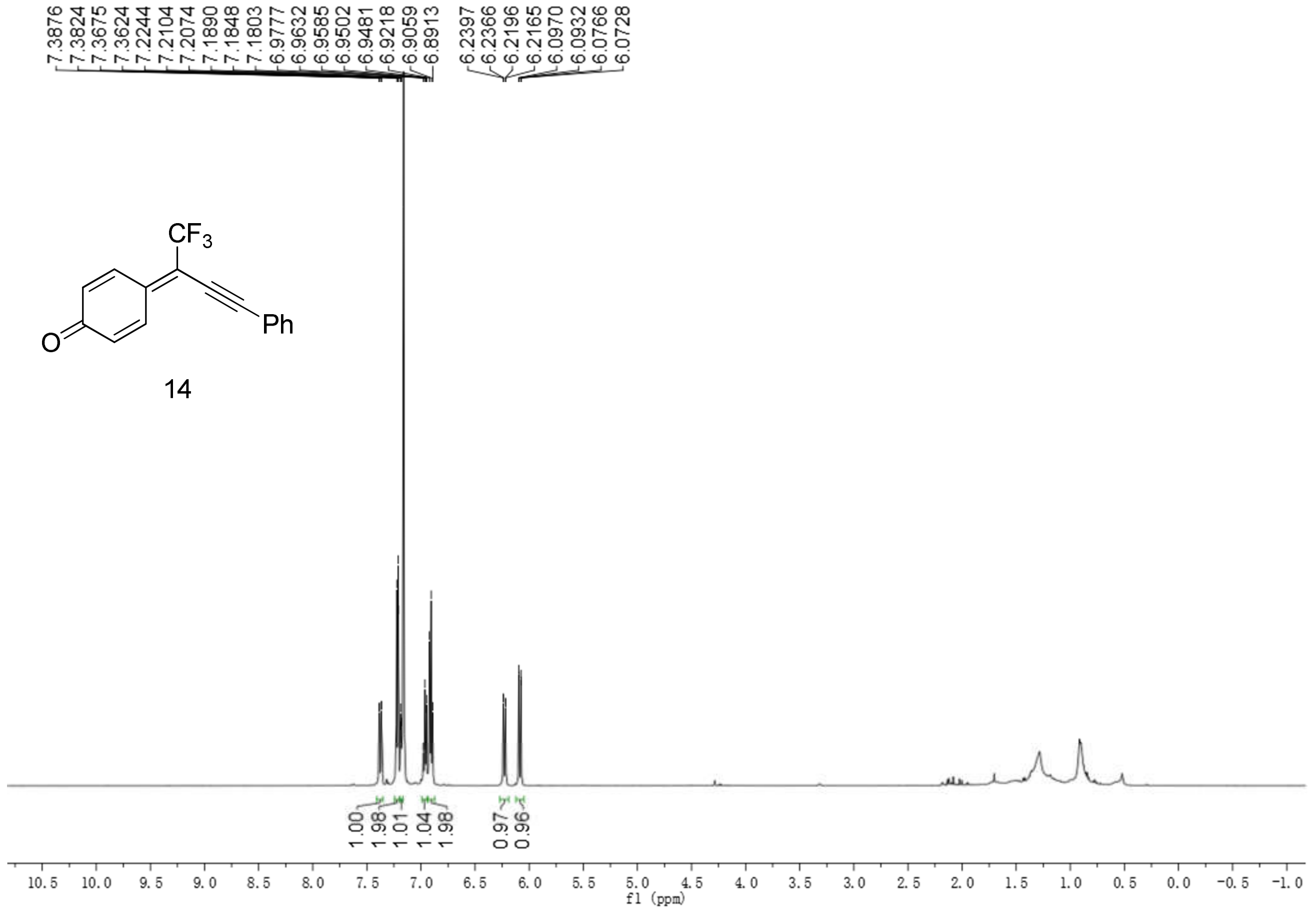
7.3876
7.3824
7.3675
7.3624
7.2244
7.2104
7.2074
7.1890
7.1848
7.1803
6.9777
6.9632
6.9585
6.9502
6.9481
6.9218
6.9059
6.8913
6.2397
6.2366
6.2196
6.2165
6.0970
6.0932
6.0766
6.0728
CF3
O
Ph
14
1.00
1.98
1.01
1.04
1.98
0.97
0.96
f1 (ppm)

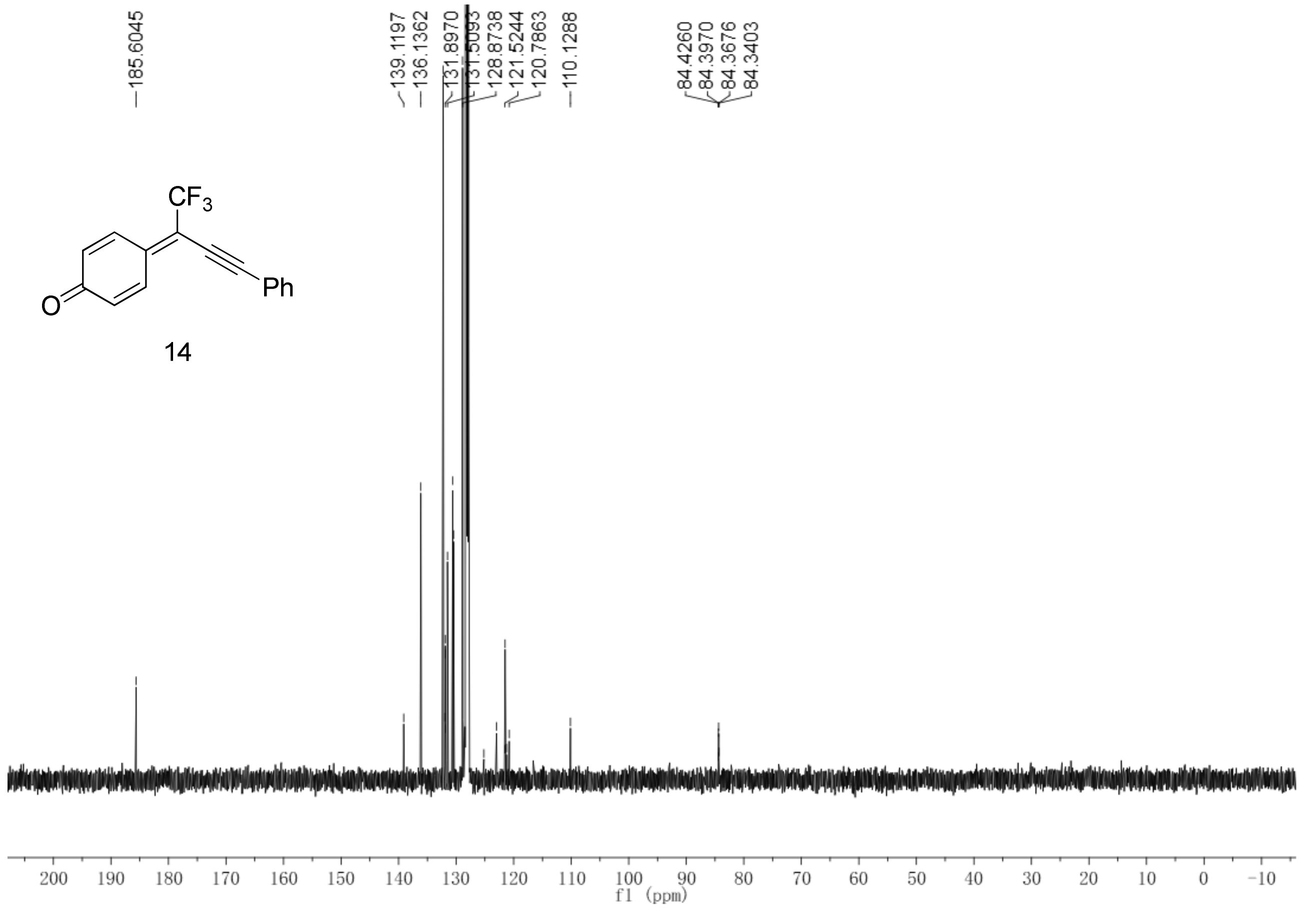
185.6045
139.1197
136.1362
131.8970
128.8738
121.5244
120.7863
110.1288
84.4260
84.3970
84.3676
84.3403
CF3
O
Ph
14
f1 (ppm)

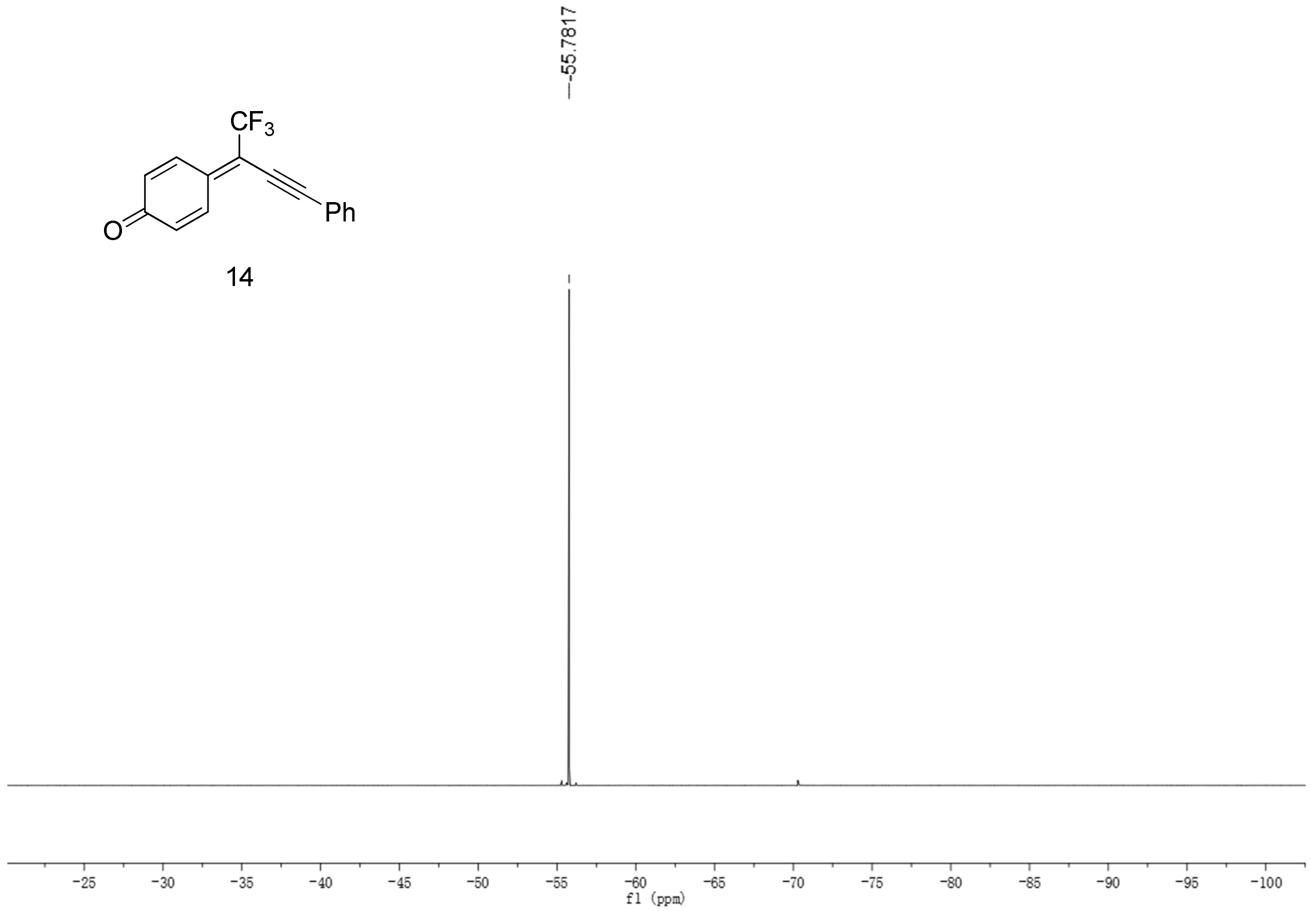

-55.7817
CF3
O
Ph
14
f1 (ppm)

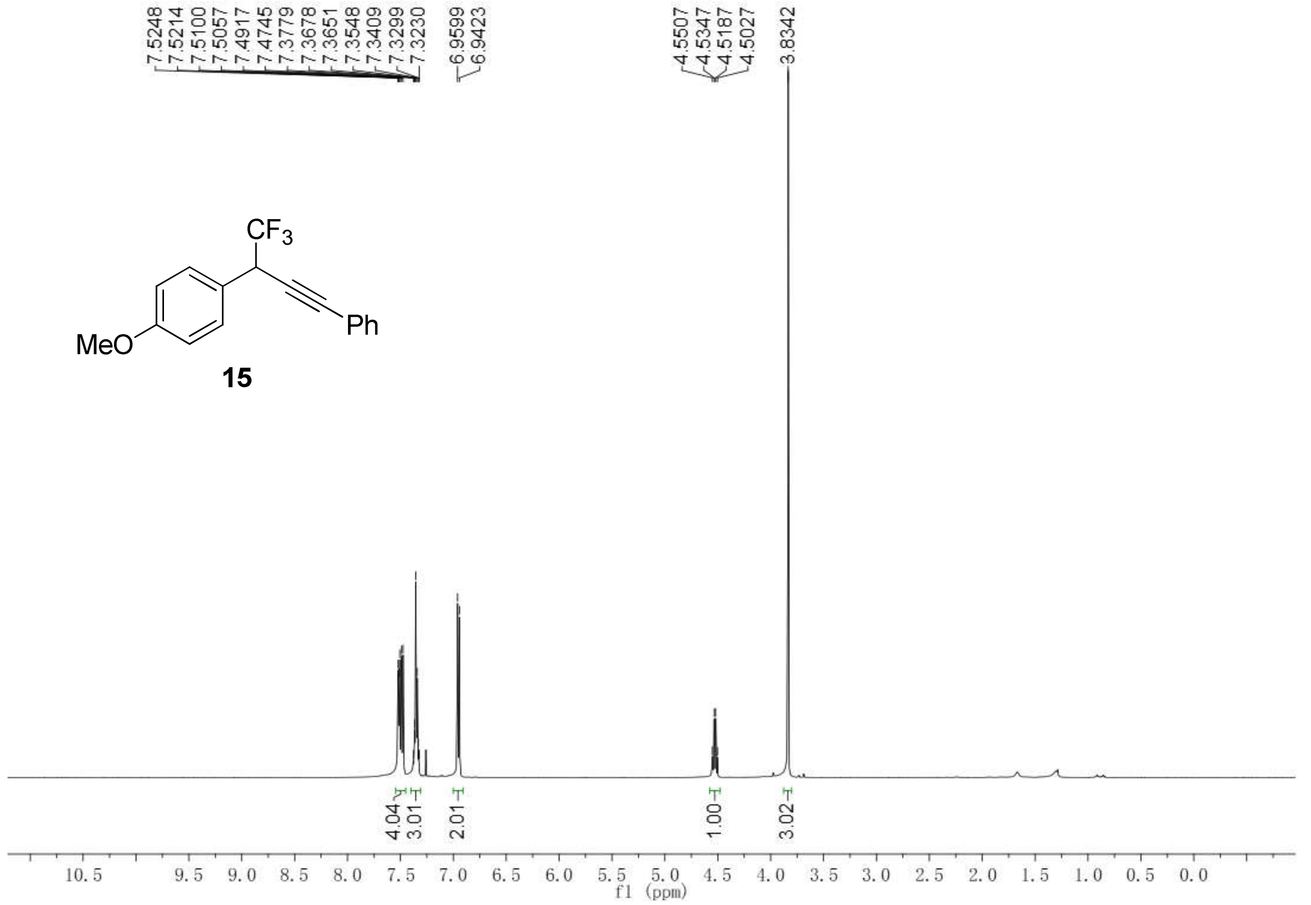

7.5248
7.5214
7.5100
7.5057
7.4917
7.4745
7.3779
7.3678
7.3651
7.3548
7.3409
7.3299
7.3230
6.9599
6.9423
4.5507
4.5347
4.5187
4.5027
3.8342
CF3
MeO
Ph
15
4.04
3.01
2.01
1.00
3.02
f1 (ppm)

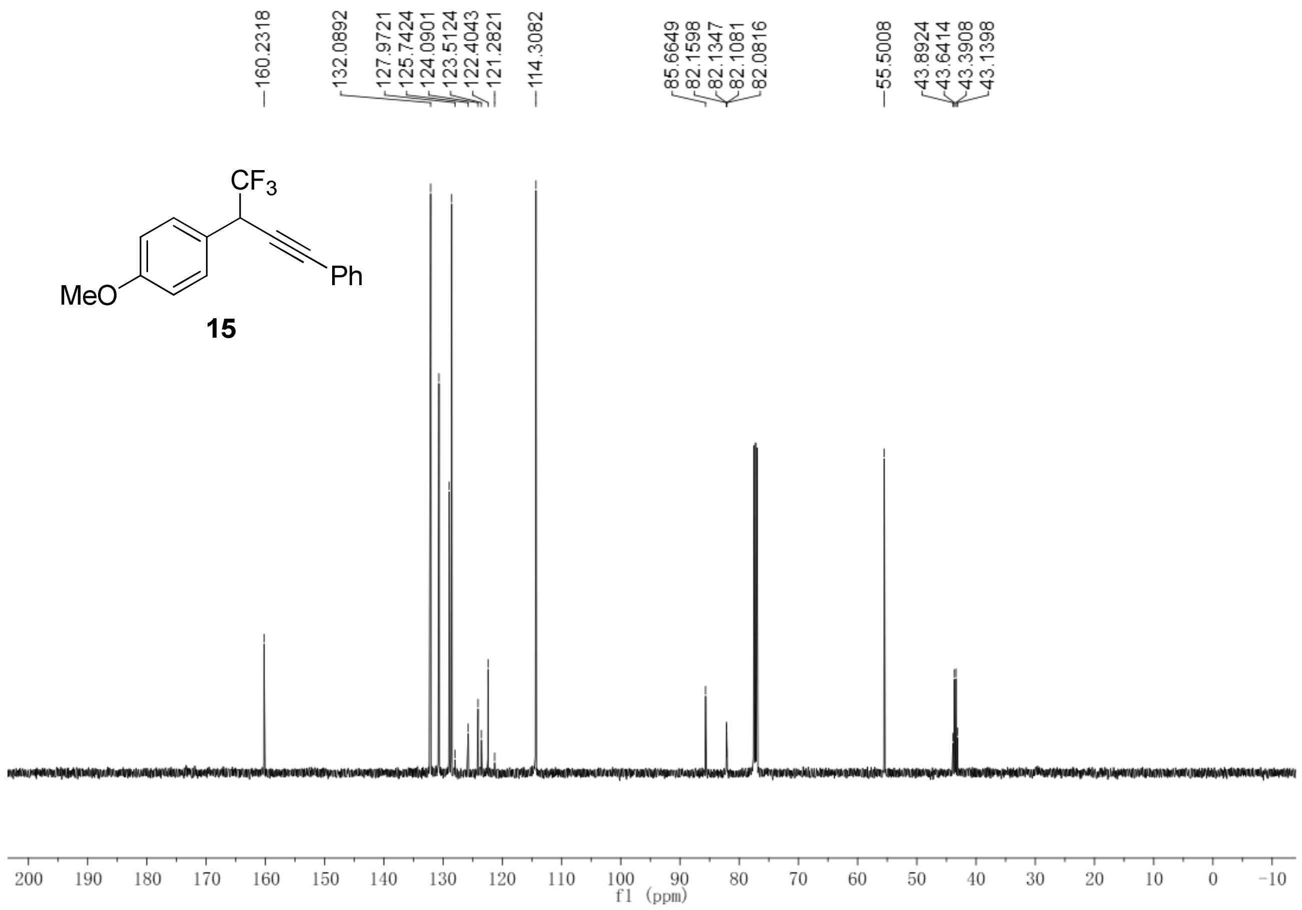
160.2318
132.0892
127.9721
125.7424
124.0901
123.5124
122.4043
121.2821
114.3082
85.6649
82.1598
82.1347
82.1081
82.0816
55.5008
43.8924
43.6414
43.3908
43.1398
CF_3
MeO
Ph
15
200 190 180 170 160 150 140 130 120 110 100 90 80 70 60 50 40 30 20 10 0 −10
f1 (ppm)

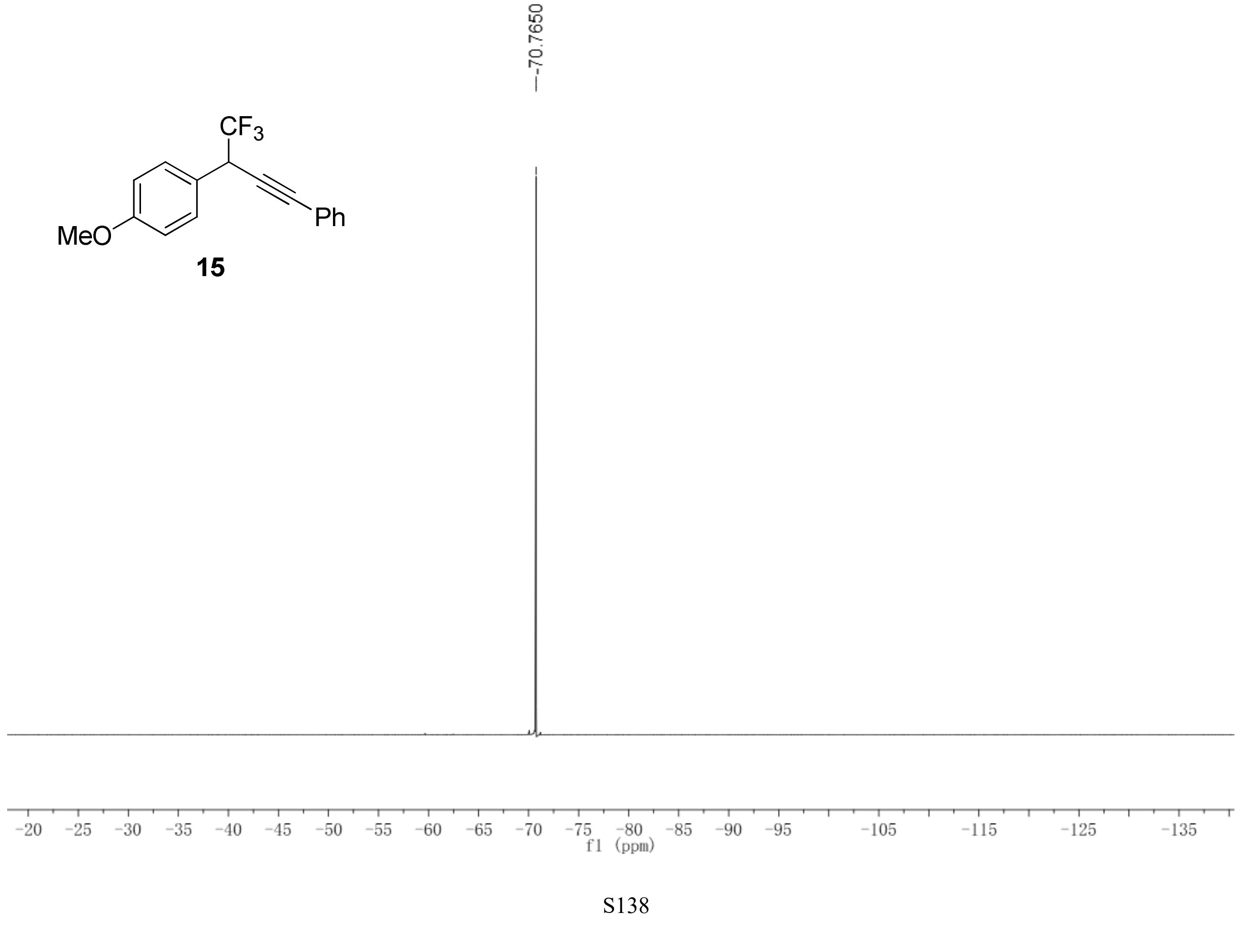
-70.7650
CF_3
MeO
Ph
15
−20 −25 −30 −35 −40 −45 −50 −55 −60 −65 −70 −75 −80 −85 −90 −95 −105 −115 −125 −135
f1 (ppm)

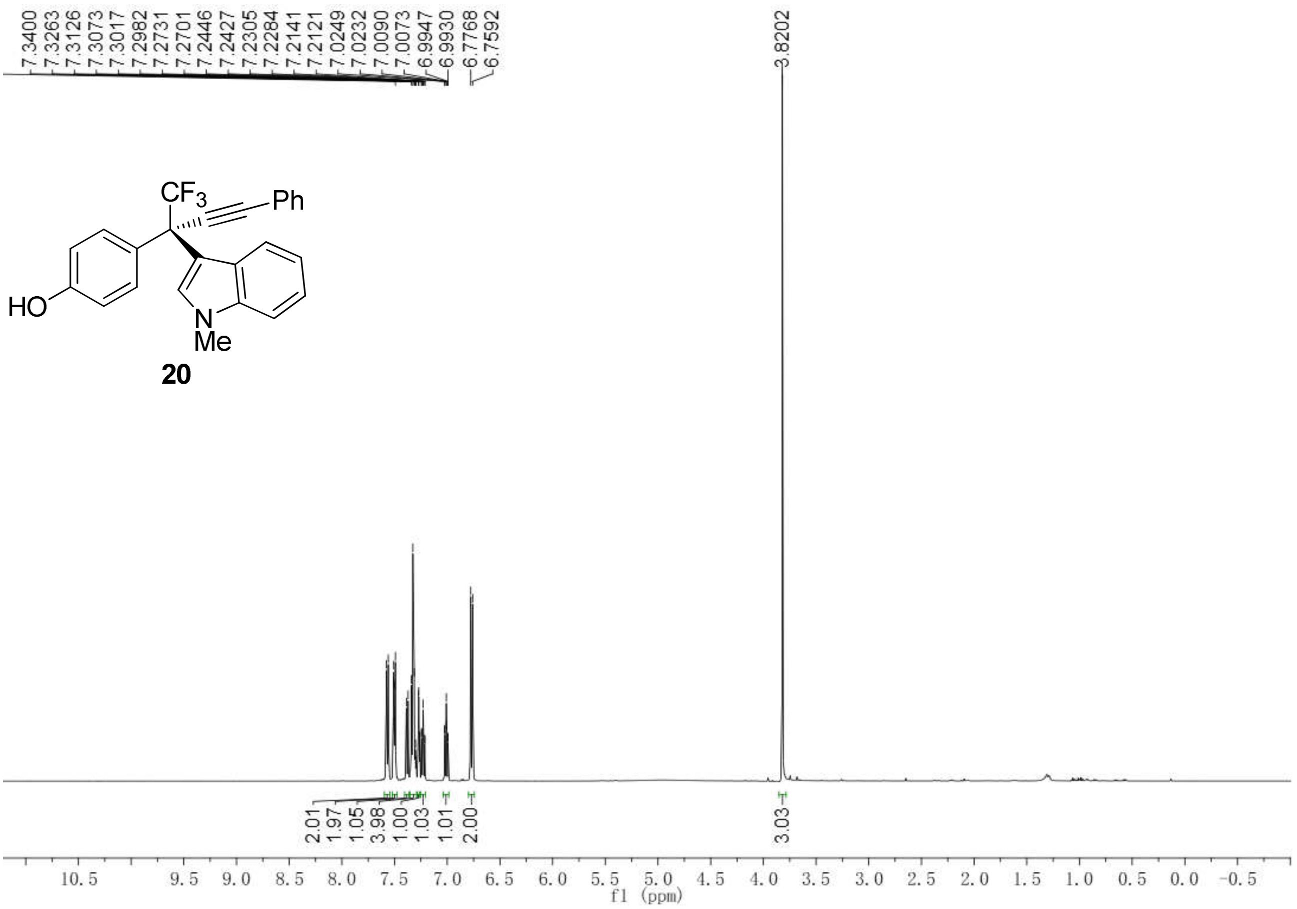
7.3400
7.3263
7.3126
7.3073
7.3017
7.2982
7.2731
7.2701
7.2446
7.2427
7.2305
7.2284
7.2141
7.2121
7.0249
7.0232
7.0090
7.0073
6.9947
6.9930
6.7768
6.7592
3.8202
CF3
Ph
HO
N
Me
20
2.01
1.97
1.05
3.98
1.00
1.03
1.01
2.00
3.03
10.5 9.5 9.0 8.5 8.0 7.5 7.0 6.5 6.0 5.5 5.0 4.5 4.0 3.5 3.0 2.5 2.0 1.5 1.0 0.5 0.0 -0.5
f1 (ppm)

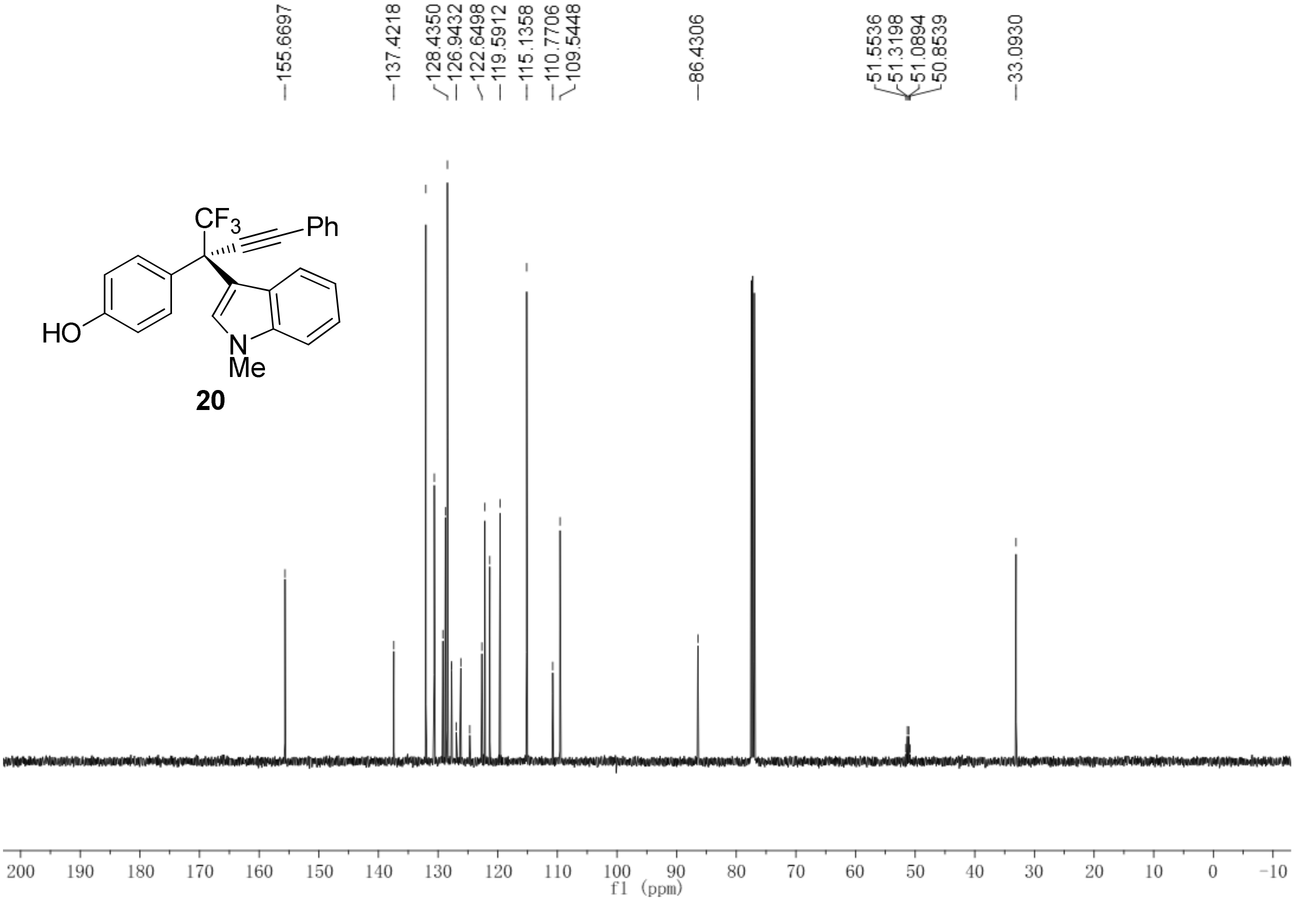
155.6697
137.4218
128.4350
126.9432
122.6498
119.5912
115.1358
110.7706
109.5448
86.4306
51.5536
51.3198
51.0894
50.8539
33.0930
CF3
Ph
HO
N
Me
20
200 190 180 170 160 150 140 130 120 110 100 90 80 70 60 50 40 30 20 10 0 -10
f1 (ppm)

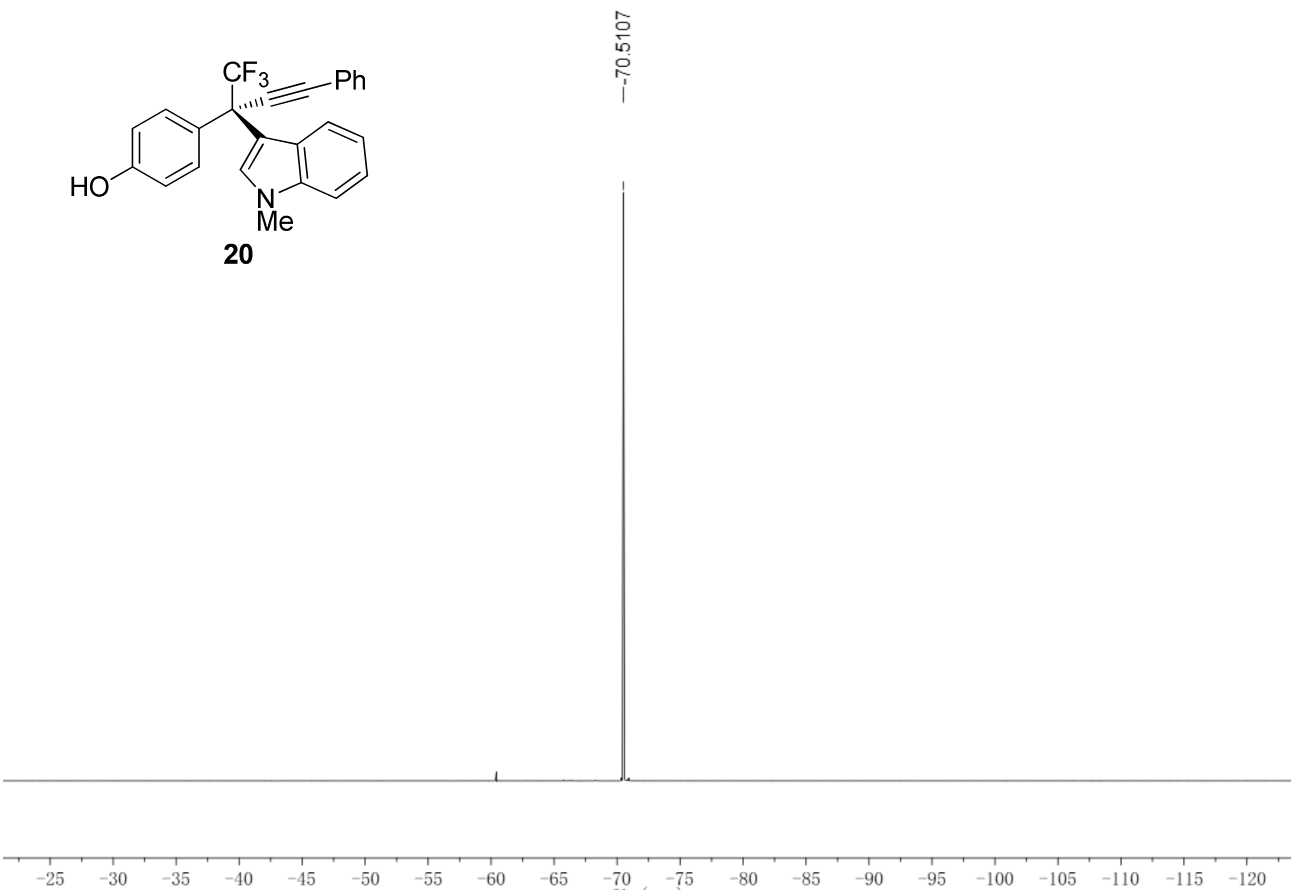

−70.5107
CF_3
Ph
HO
N
Me
20
f1 (ppm)
-25 -30 -35 -40 -45 -50 -55 -60 -65 -70 -75 -80 -85 -90 -95 -100 -105 -110 -115 -120

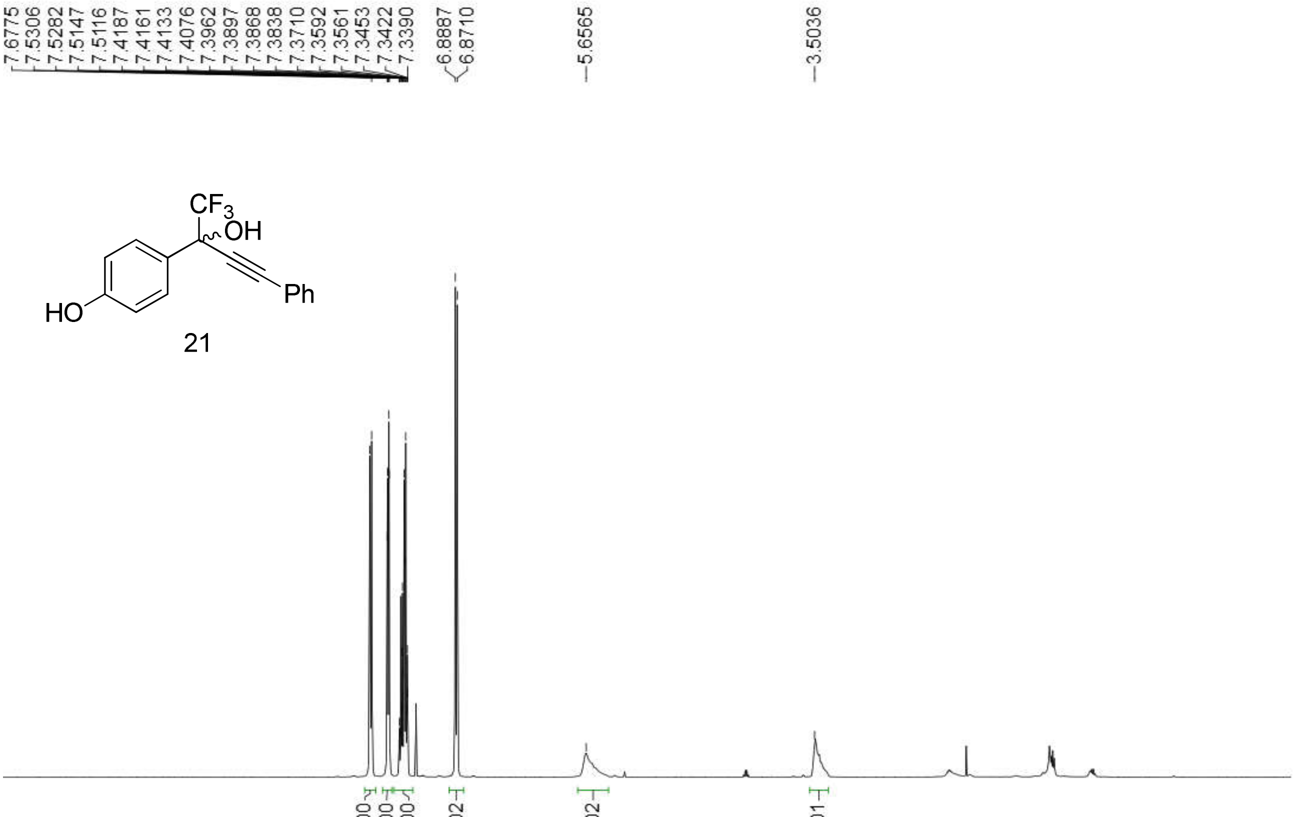

7.6775
7.5306
7.5282
7.5147
7.5116
7.4187
7.4161
7.4133
7.4076
7.3962
7.3897
7.3868
7.3838
7.3710
7.3592
7.3561
7.3453
7.3422
7.3390
6.8887
6.8710
5.6565
3.5036
CF_3
OH
Ph
HO
21
2.00
2.00
3.00
2.02
1.02
1.01
f1 (ppm)
10.5 9.5 9.0 8.5 8.0 7.5 7.0 6.5 6.0 5.5 5.0 4.5 4.0 3.5 3.0 2.5 2.0 1.5 1.0 0.5 0.0

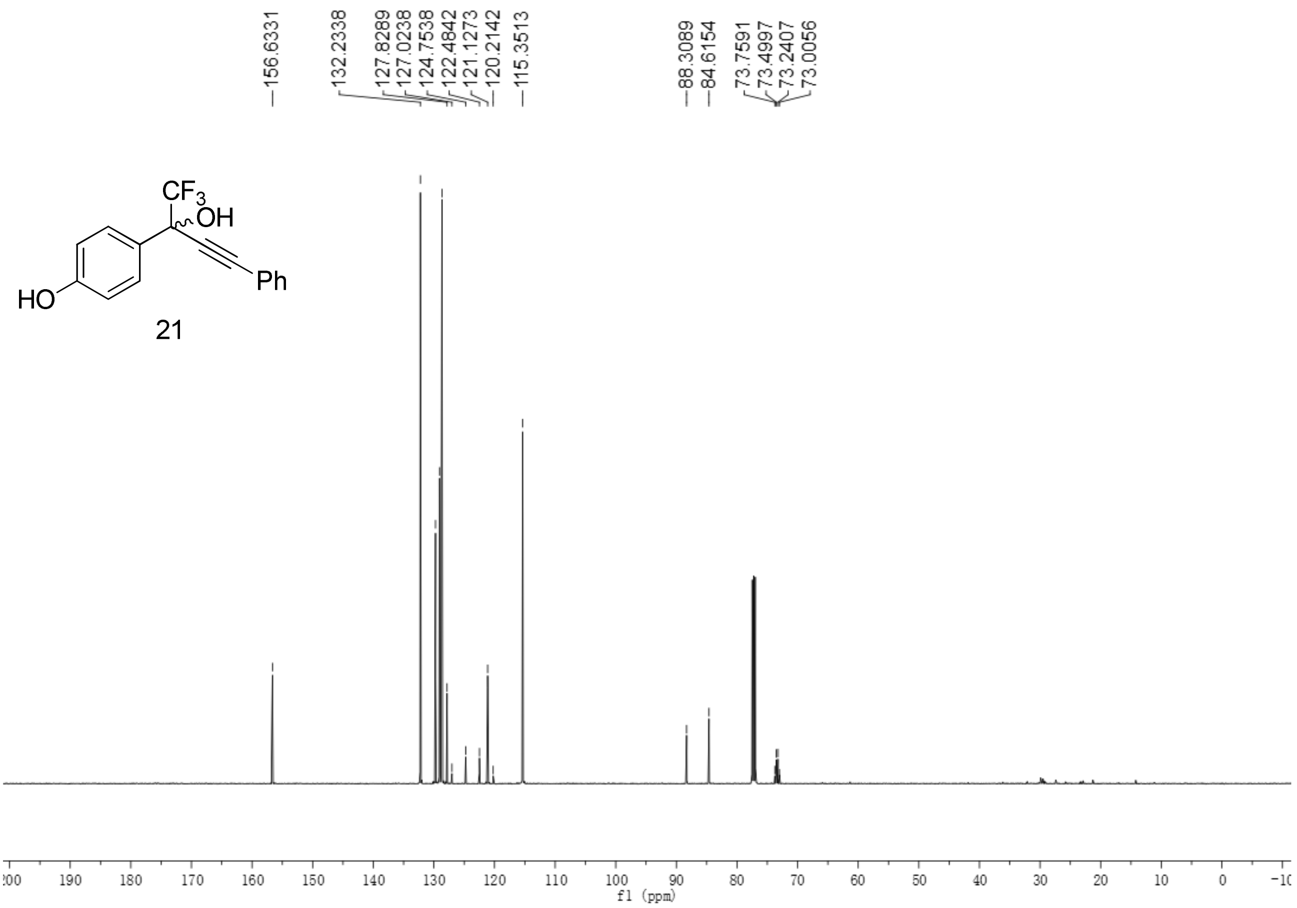
156.6331
132.2338
127.8289
127.0238
124.7538
122.4842
121.1273
120.2142
115.3513
88.3089
84.6154
73.7591
73.4997
73.2407
73.0056
CF3
OH
Ph
HO
fl (ppm)

21

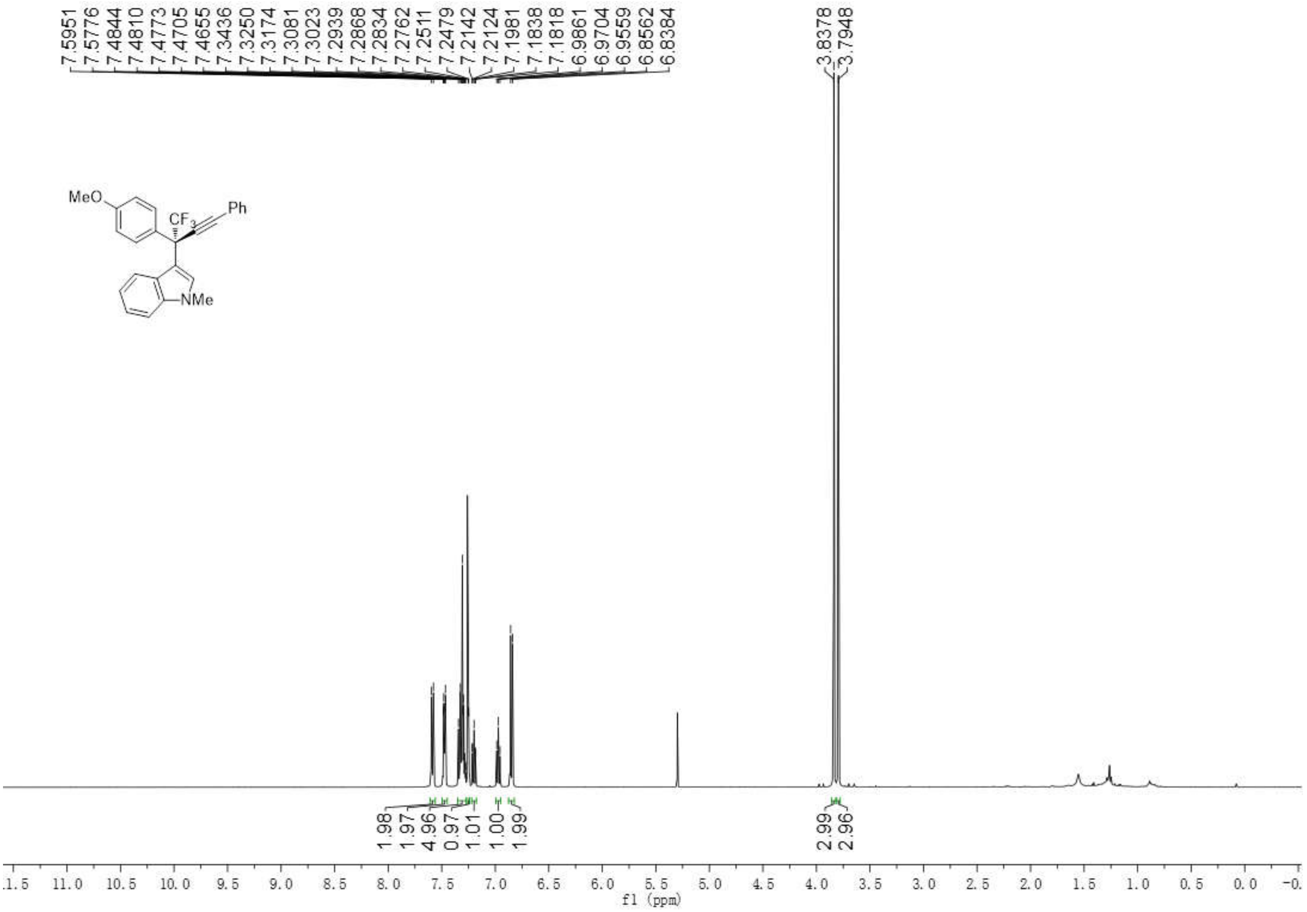
7.5951
7.5776
7.4844
7.4810
7.4773
7.4705
7.4655
7.3436
7.3250
7.3174
7.3081
7.3023
7.2939
7.2868
7.2834
7.2762
7.2511
7.2479
7.2142
7.2124
7.1981
7.1838
7.1818
6.9861
6.9704
6.9559
6.8562
6.8384
3.8378
3.7948
MeO
CF3
Ph
NMe
1.98
1.97
4.96
0.97
1.01
1.00
1.99
2.99
2.96
fl (ppm)

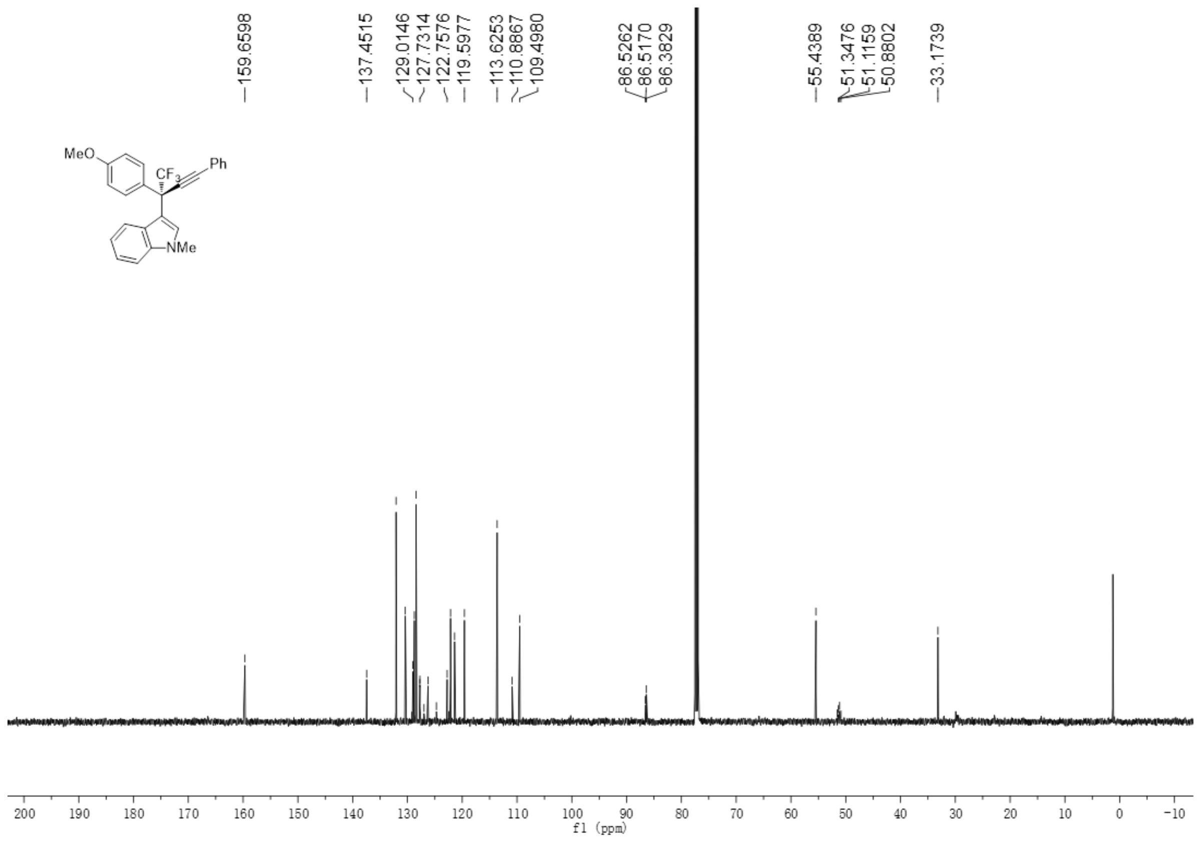
159.6598
137.4515
129.0146
127.7314
122.7576
119.5977
113.6253
110.8867
109.4980
86.5262
86.5170
86.3829
55.4389
51.3476
51.1159
50.8802
33.1739
MeO
CF3
Ph
NMe
f1 (ppm)

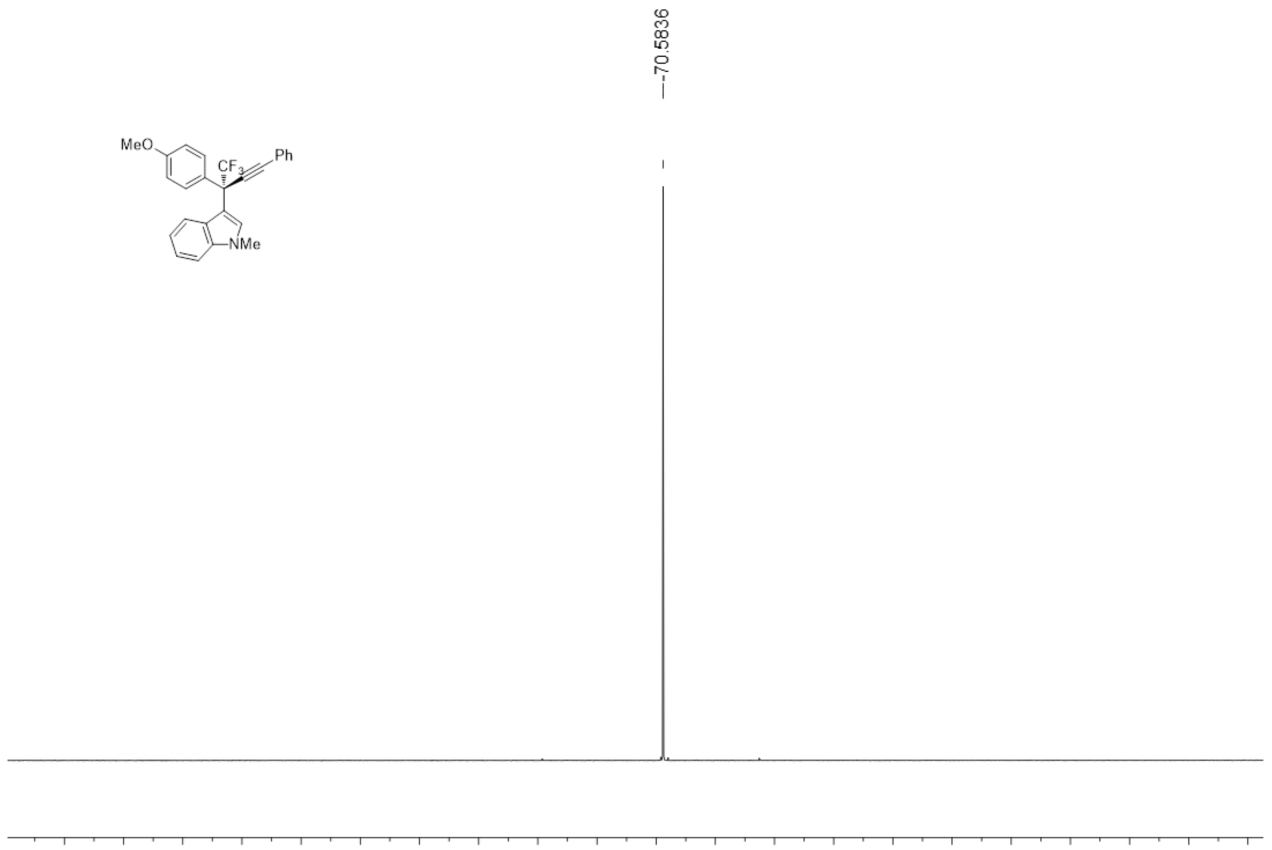
-70.5836
MeO
CF3
Ph
NMe
f1 (ppm)

HPLC spectra for ee determination

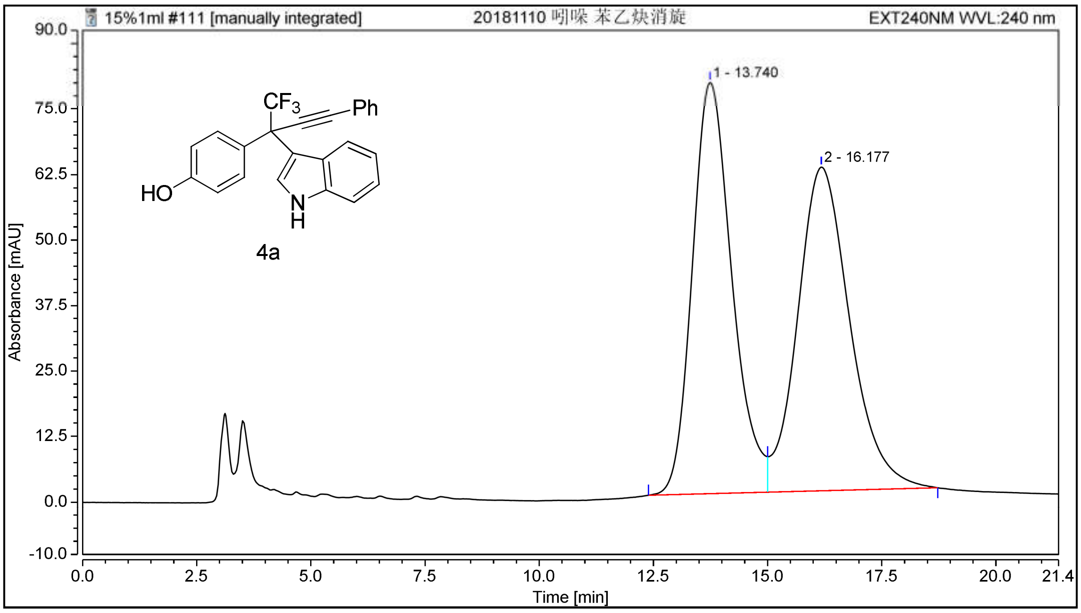

Integration Results					
No.	Peak Name	Retention Time<br>min	Area<br>mAU*min	Relative Area<br>%	Amount<br>n.a.
1		13.740	78.782	49.04	n.a.
2		16.177	81.864	50.96	n.a.
**Total:**			**160.646**	**100.00**	

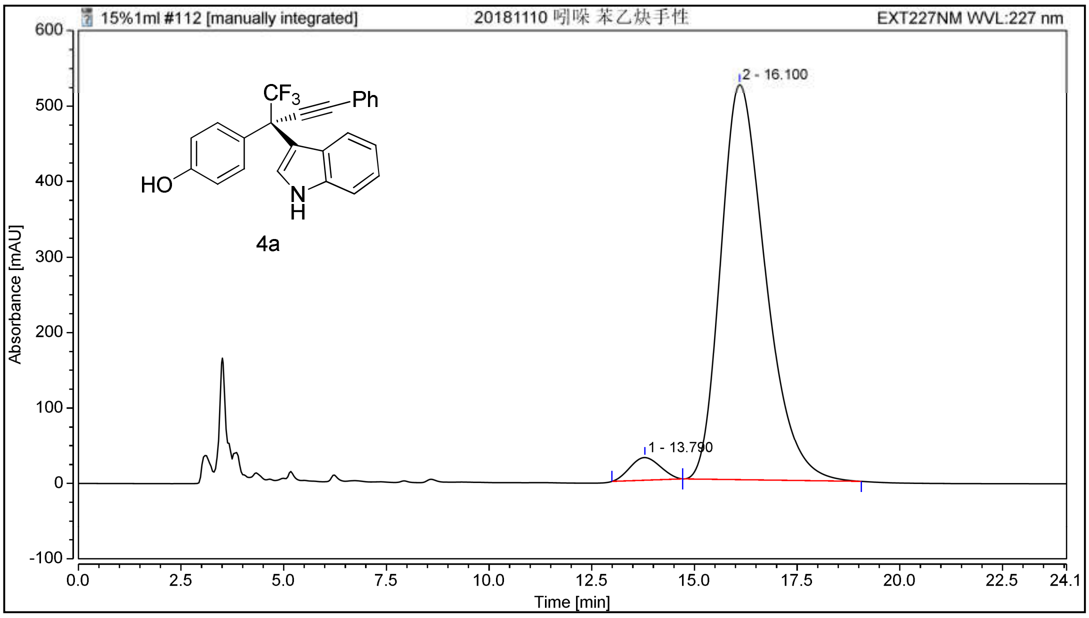

Integration Results					
No.	Peak Name	Retention Time<br>min	Area<br>mAU*min	Relative Area<br>%	Amount<br>n.a.
1		13.790	25.534	3.71	n.a.
2		16.100	662.918	96.29	n.a.
**Total:**			**688.452**	**100.00**	

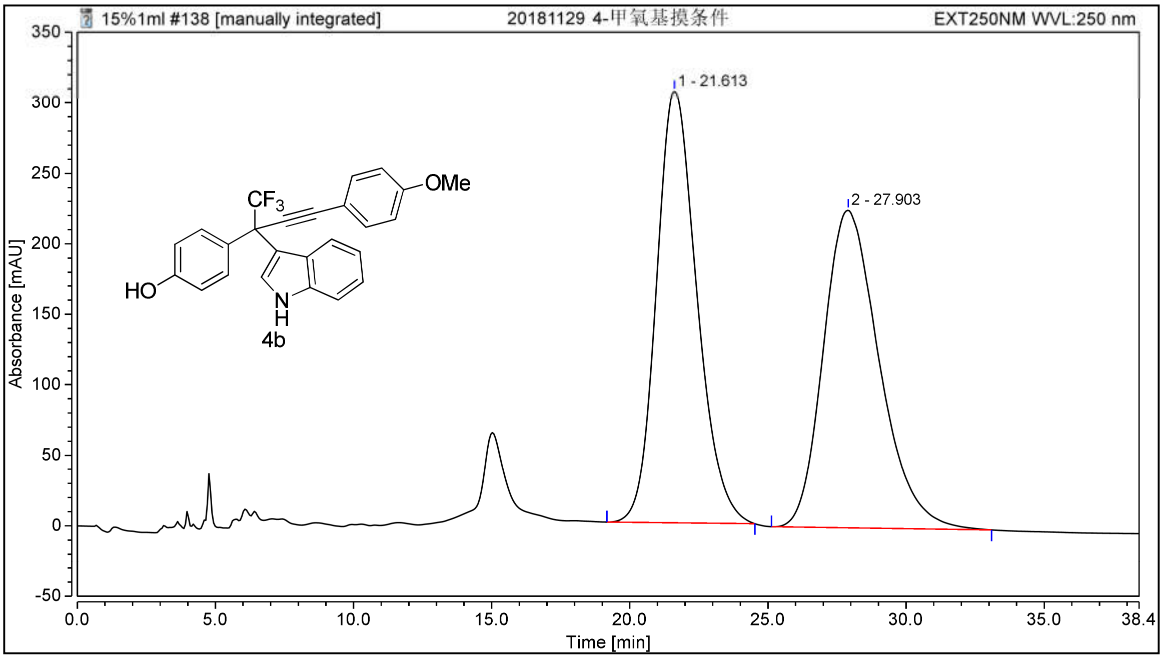

Integration Results

No.	Peak Name	Retention Time min	Area mAU*min	Relative Area %	Amount n.a.
1		21.613	519.701	49.63	n.a.
2		27.903	527.413	50.37	n.a.
**Total:**			**1047.114**	**100.00**	

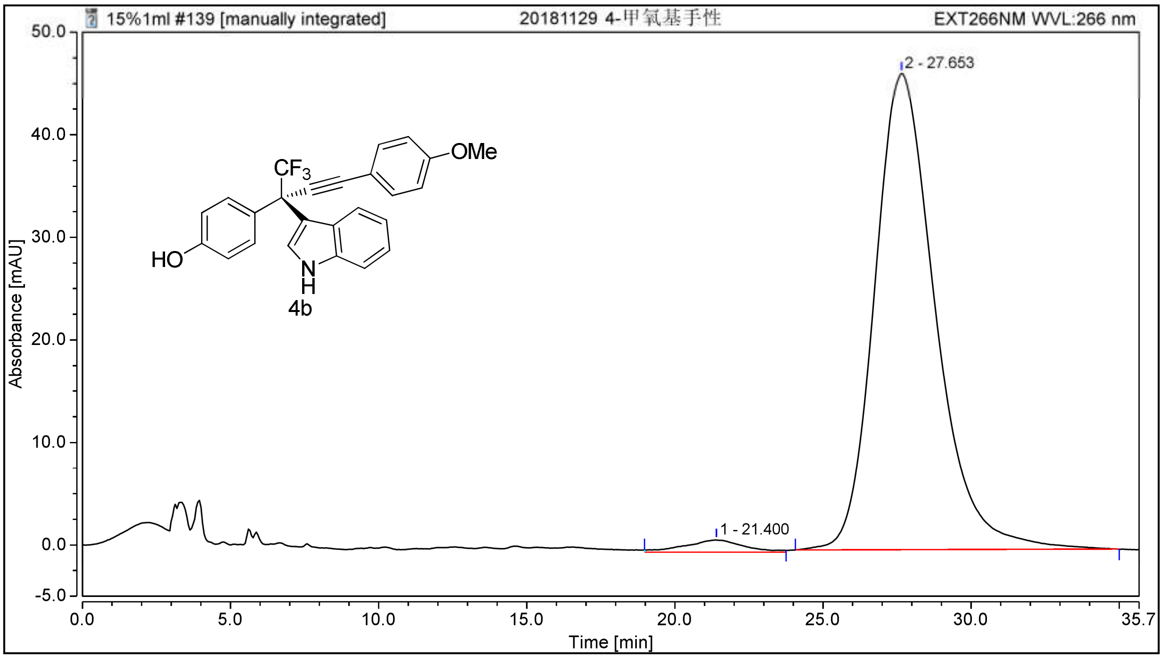

Integration Results

No.	Peak Name	Retention Time min	Area mAU*min	Relative Area %	Amount n.a.
1		21.400	2.749	2.44	n.a.
2		27.653	109.940	97.56	n.a.
**Total:**			**112.689**	**100.00**	

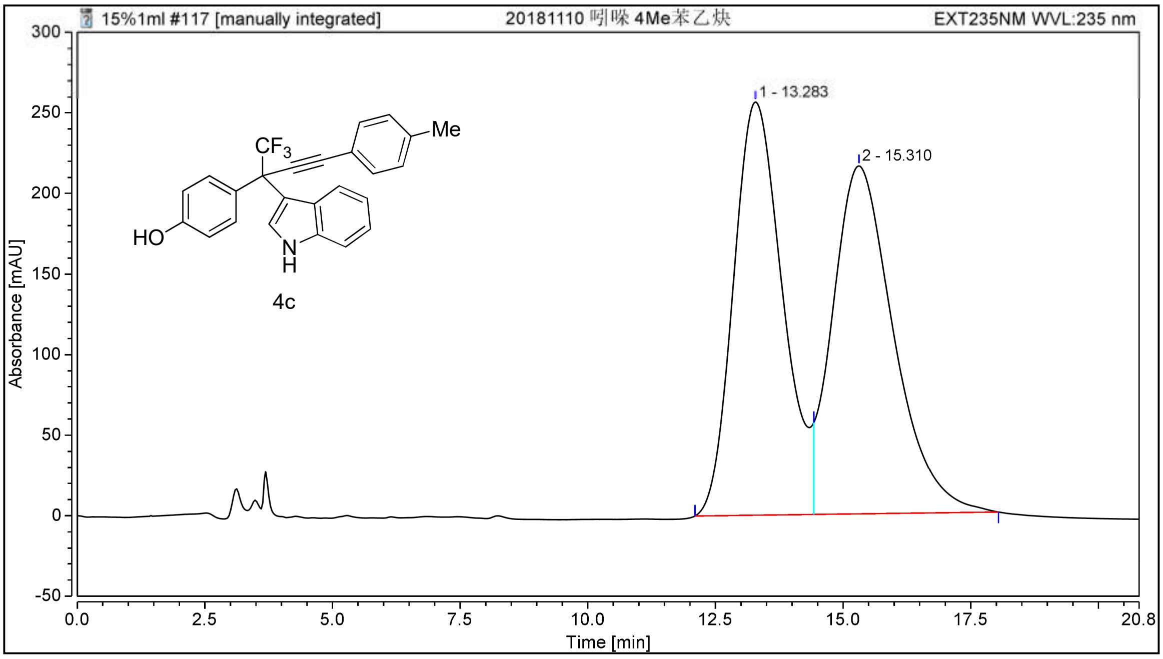

Integration Results					
No.	Peak Name	Retention Time<br>min	Area<br>mAU*min	Relative Area<br>%	Amount<br>n.a.
1		13.283	286.196	48.99	n.a.
2		15.310	298.009	51.01	n.a.
**Total:**			**584.204**	**100.00**	

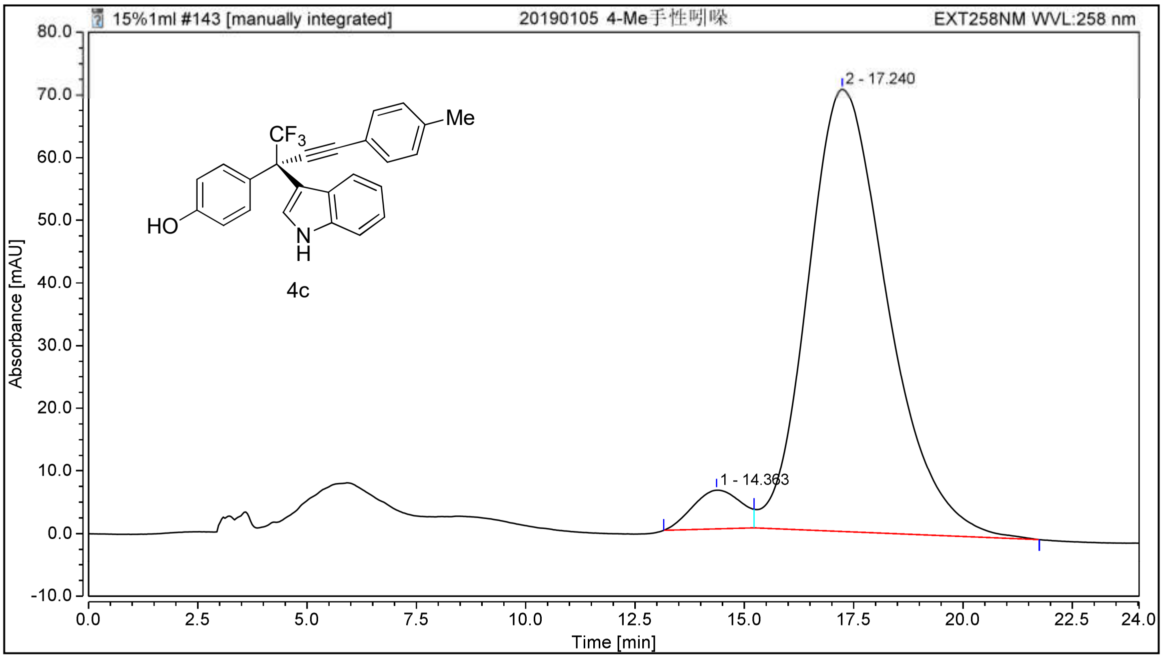

Integration Results					
No.	Peak Name	Retention Time<br>min	Area<br>mAU*min	Relative Area<br>%	Amount<br>n.a.
1		14.363	7.928	5.04	n.a.
2		17.240	149.396	94.96	n.a.
**Total:**			**157.324**	**100.00**	

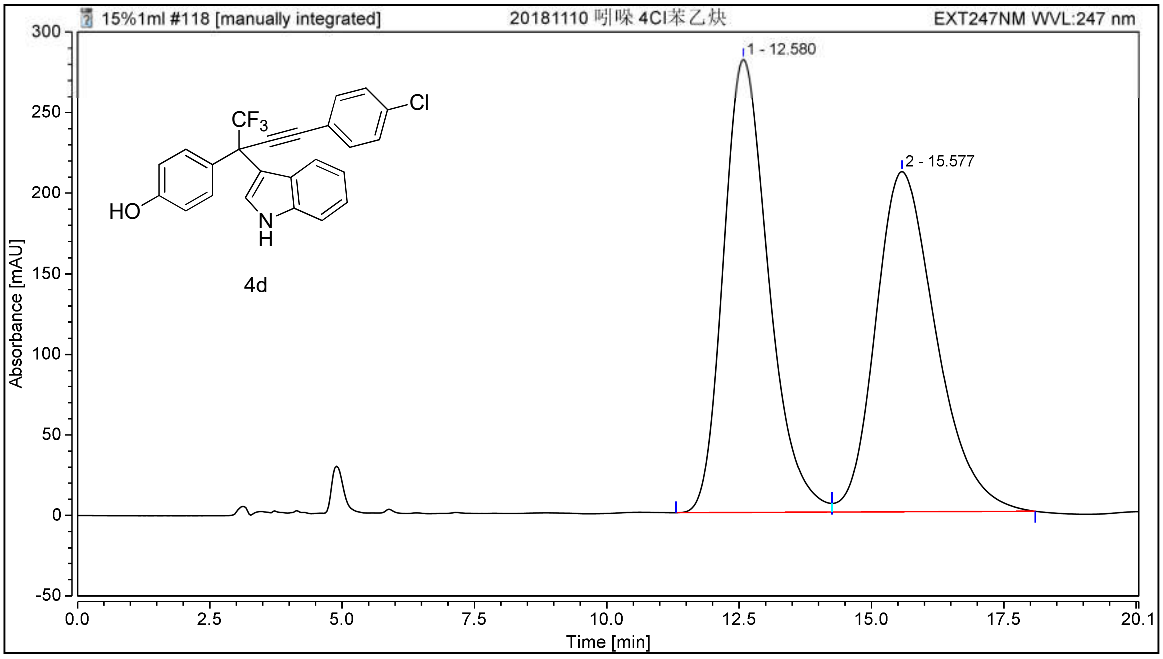

Integration Results

No.	Peak Name	Retention Time min	Area mAU*min	Relative Area %	Amount n.a.
1		12.580	278.634	49.94	n.a.
2		15.577	279.358	50.06	n.a.
**Total:**			**557.993**	**100.00**	

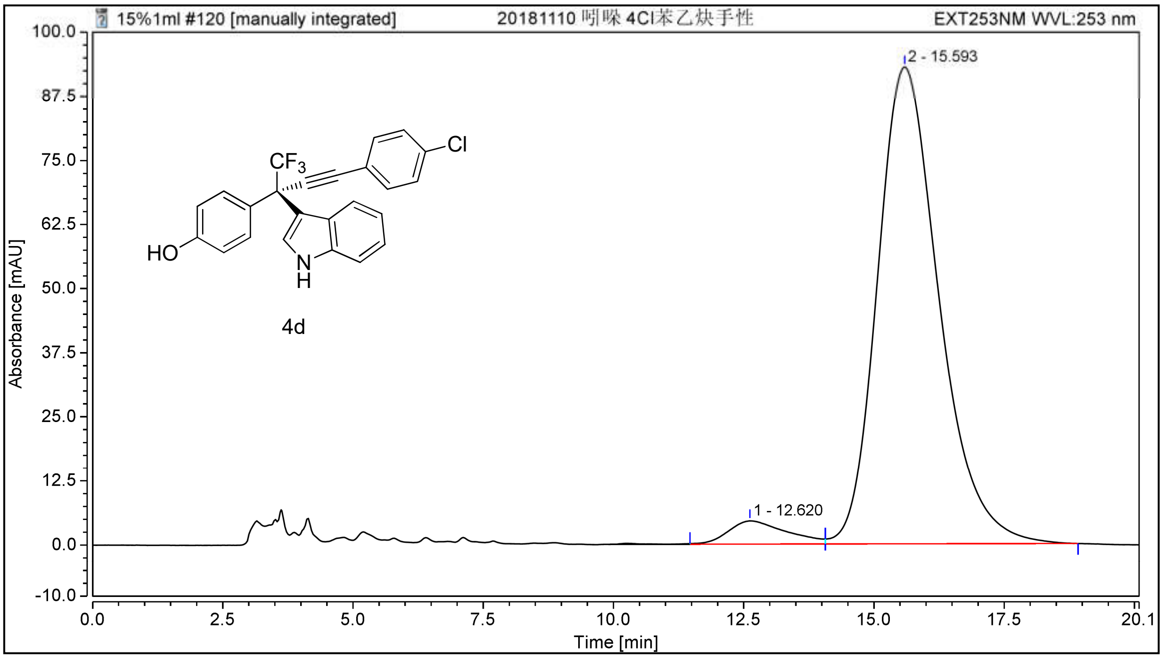

Integration Results

No.	Peak Name	Retention Time min	Area mAU*min	Relative Area %	Amount n.a.
1		12.620	5.829	4.39	n.a.
2		15.593	127.027	95.61	n.a.
**Total:**			**132.856**	**100.00**	

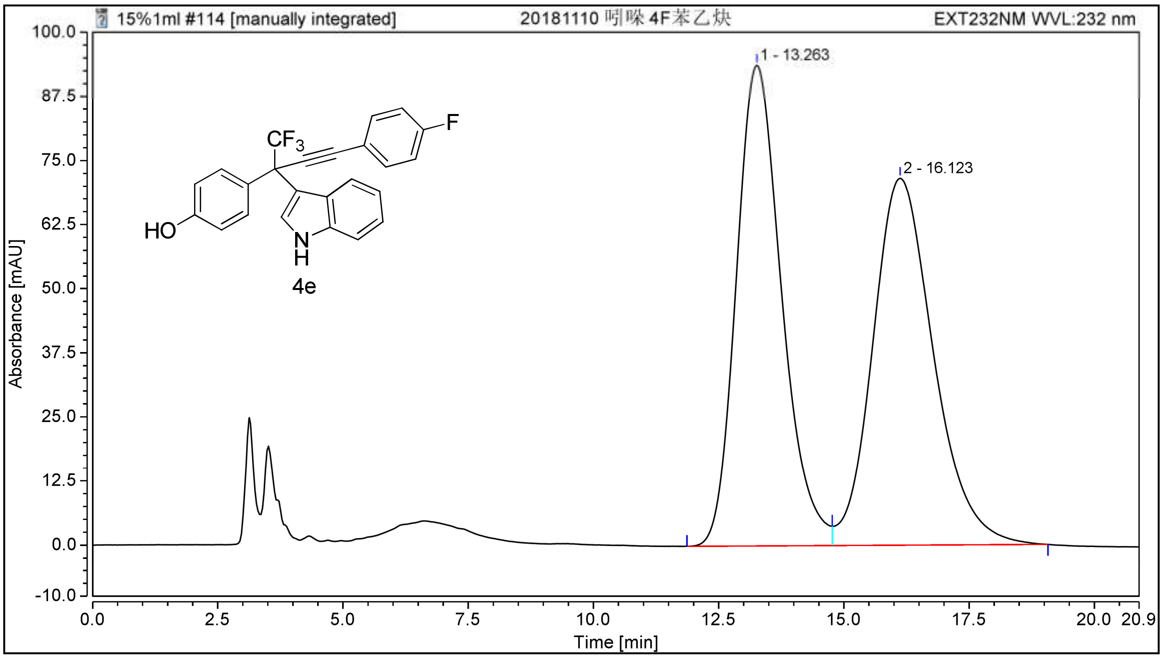

Integration Results

No.	Peak Name	Retention Time<br>min	Area<br>mAU*min	Relative Area<br>%	Amount<br>n.a.
1		13.263	98.168	49.43	n.a.
2		16.123	100.422	50.57	n.a.
**Total:**			**198.589**	**100.00**	

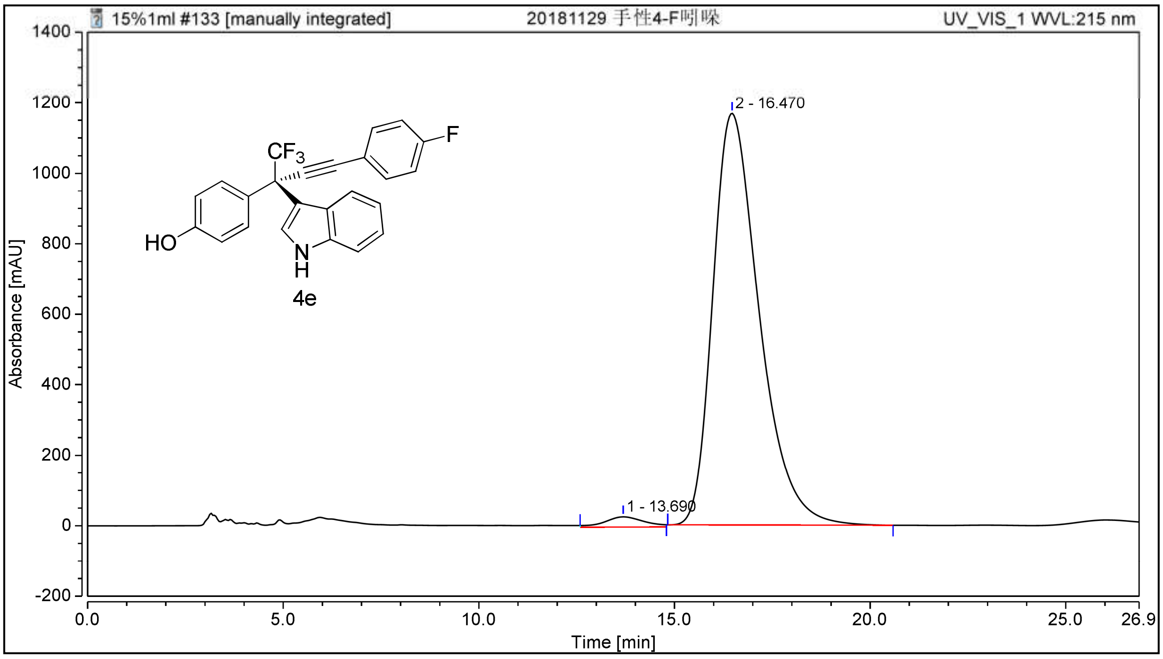

Integration Results

No.	Peak Name	Retention Time<br>min	Area<br>mAU*min	Relative Area<br>%	Amount<br>n.a.
1		13.690	34.242	2.09	n.a.
2		16.470	1604.920	97.91	n.a.
**Total:**			**1639.162**	**100.00**	

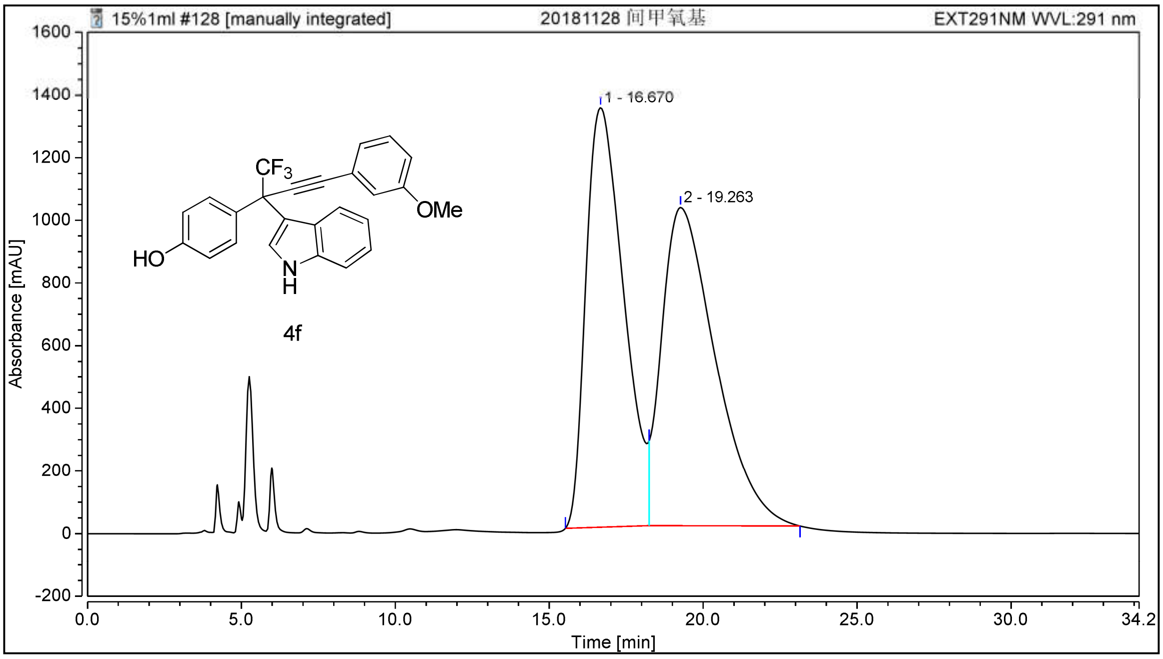

Integration Results					
No.	Peak Name	Retention Time min	Area mAU*min	Relative Area %	Amount n.a.
1		16.670	1901.324	48.13	n.a.
2		19.263	2049.436	51.87	n.a.
**Total:**			**3950.760**	**100.00**	

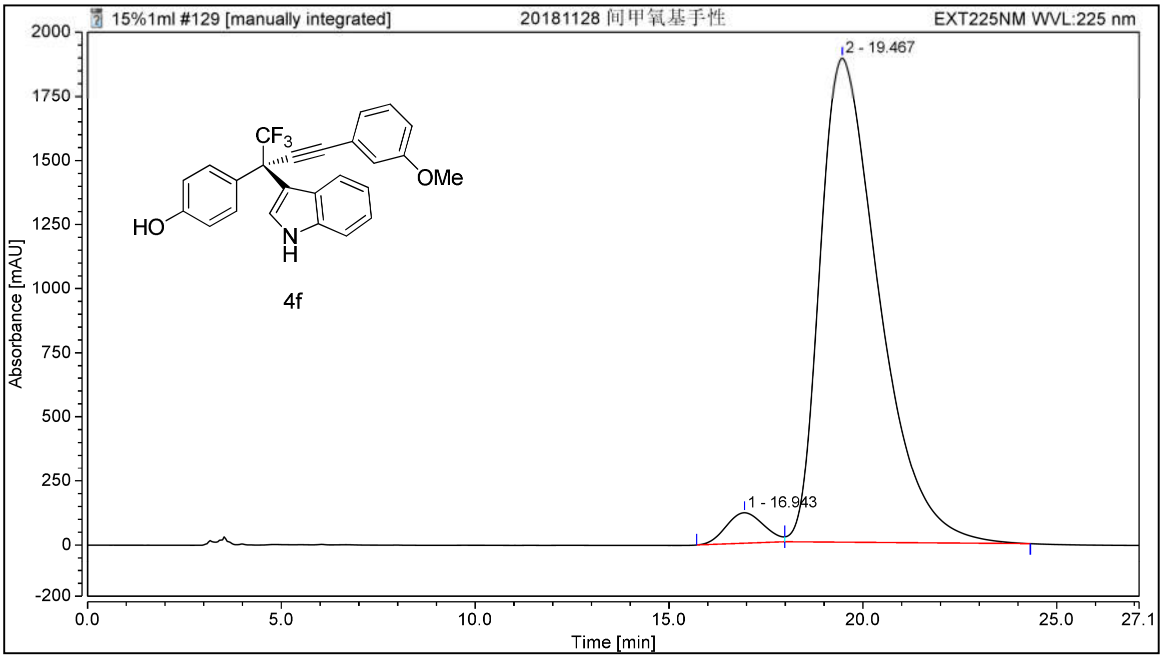

Integration Results					
No.	Peak Name	Retention Time min	Area mAU*min	Relative Area %	Amount n.a.
1		16.943	133.471	3.85	n.a.
2		19.467	3329.168	96.15	n.a.
**Total:**			**3462.639**	**100.00**	

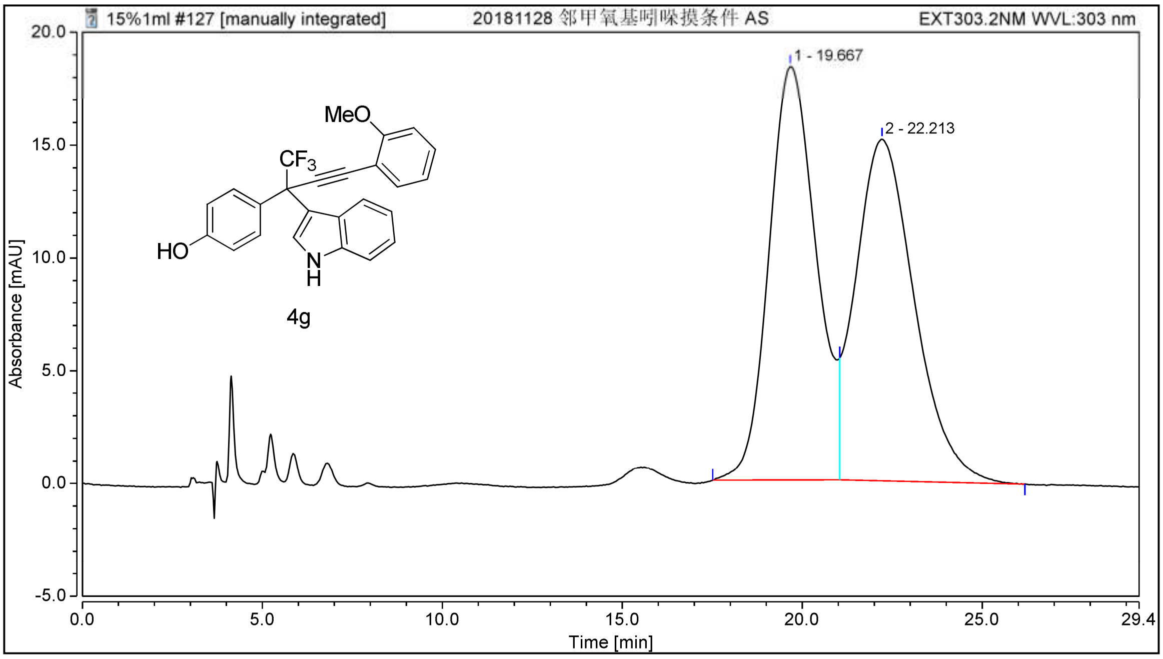

Integration Results

No.	Peak Name	Retention Time<br>min	Area<br>mAU*min	Relative Area<br>%	Amount<br>n.a.
1		19.667	27.908	49.39	n.a.
2		22.213	28.595	50.61	n.a.
**Total:**			**56.503**	**100.00**	

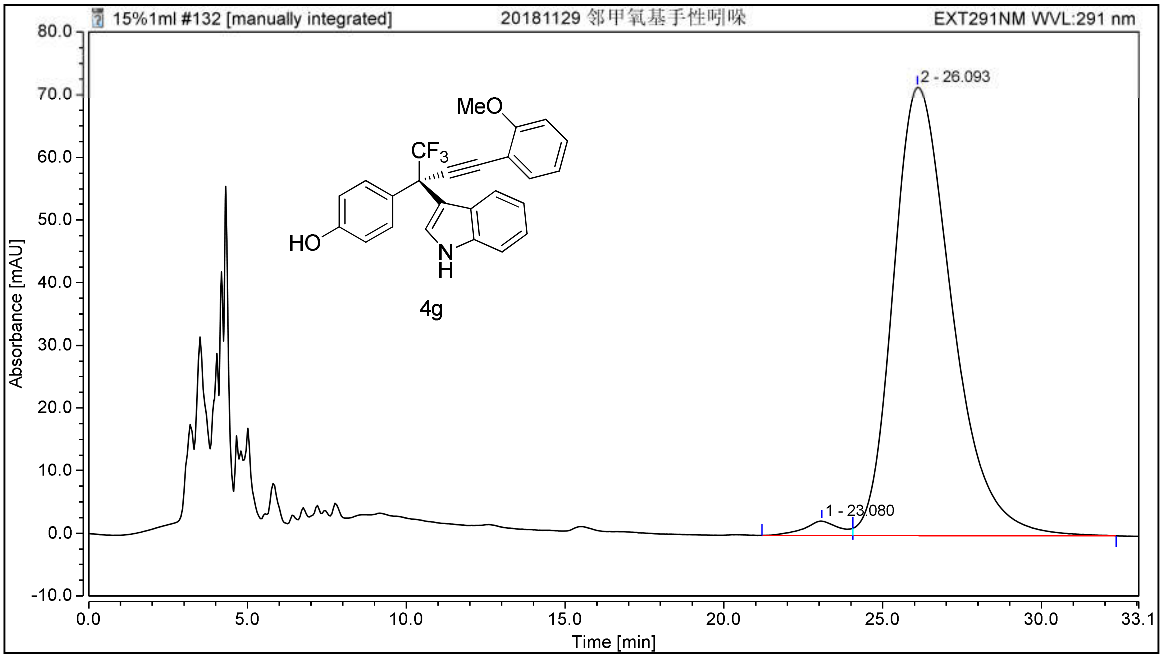

Integration Results

No.	Peak Name	Retention Time<br>min	Area<br>mAU*min	Relative Area<br>%	Amount<br>n.a.
1		23.080	2.958	1.90	n.a.
2		26.093	153.050	98.10	n.a.
**Total:**			**156.008**	**100.00**	

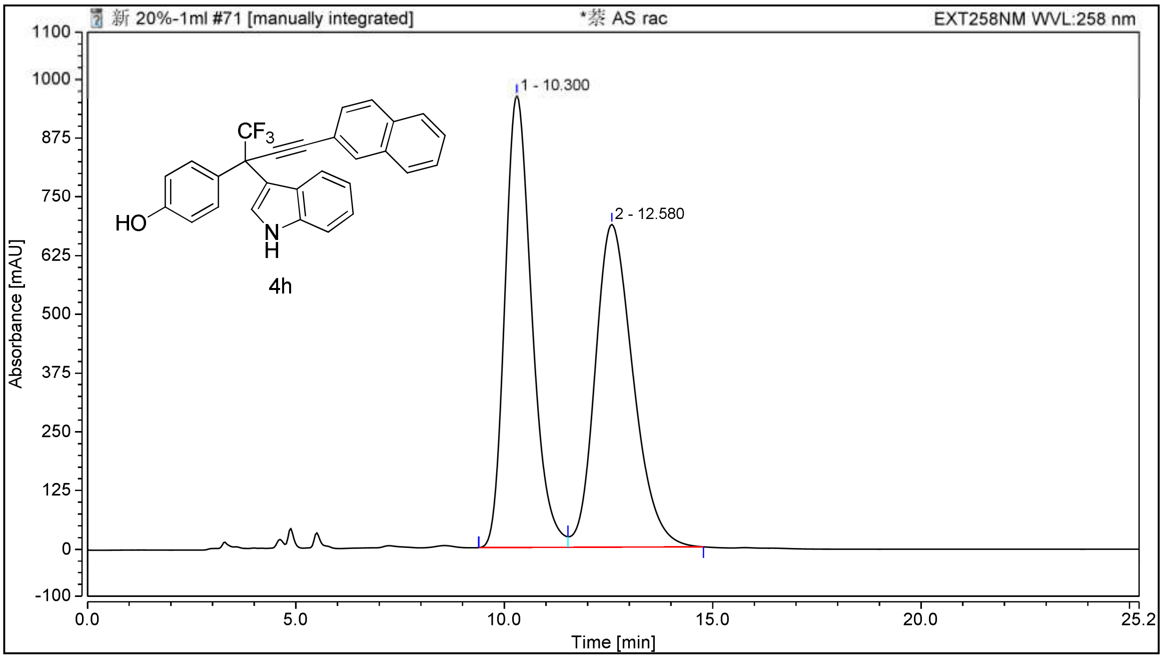

Integration Results

No.	Peak Name	Retention Time<br>min	Area<br>mAU*min	Relative Area<br>%	Amount<br>n.a.
1		10.300	717.035	49.76	n.a.
2		12.580	723.885	50.24	n.a.
**Total:**			**1440.920**	**100.00**	

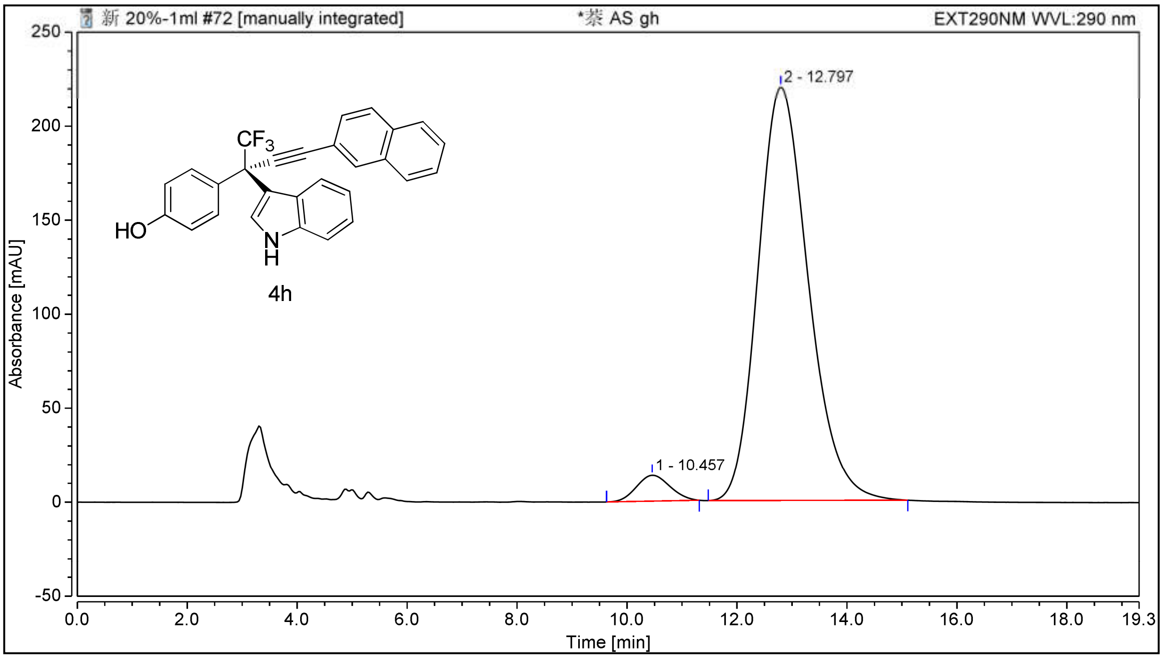

Integration Results

No.	Peak Name	Retention Time<br>min	Area<br>mAU*min	Relative Area<br>%	Amount<br>n.a.
1		10.457	9.626	3.97	n.a.
2		12.797	233.079	96.03	n.a.
**Total:**			**242.705**	**100.00**	

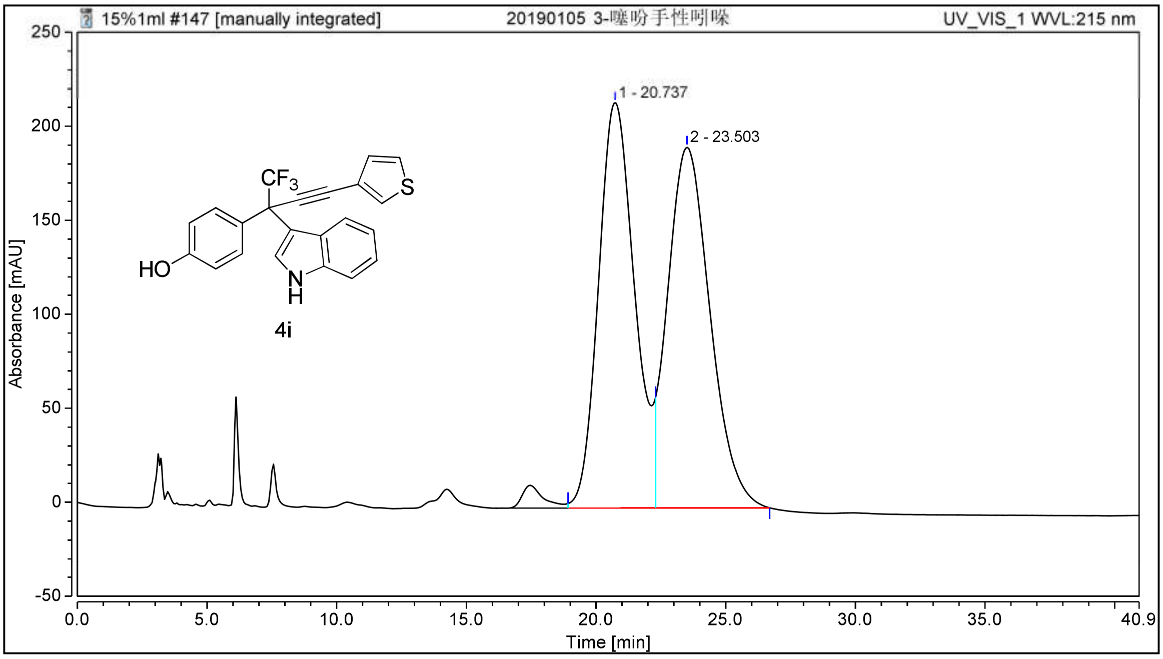

Integration Results

No.	Peak Name	Retention Time<br>min	Area<br>mAU*min	Relative Area<br>%	Amount<br>n.a.
1		20.737	348.887	49.03	n.a.
2		23.503	362.676	50.97	n.a.
**Total:**			**711.563**	**100.00**	

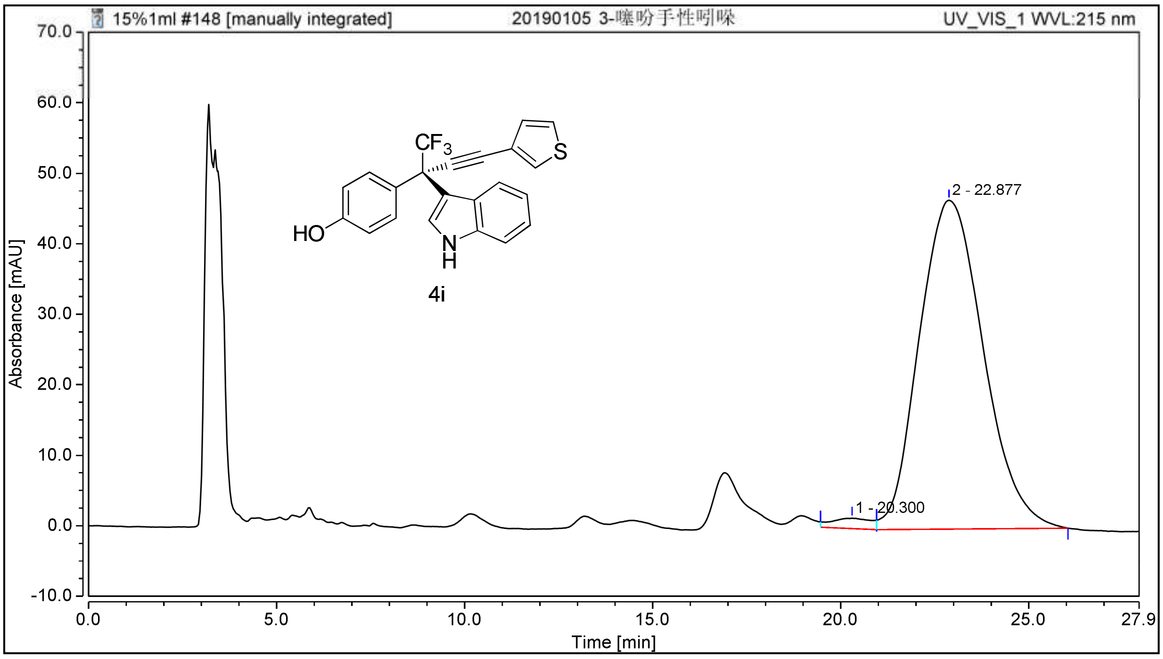

Integration Results

No.	Peak Name	Retention Time<br>min	Area<br>mAU*min	Relative Area<br>%	Amount<br>n.a.
1		20.300	1.791	1.91	n.a.
2		22.877	92.179	98.09	n.a.
**Total:**			**93.969**	**100.00**	

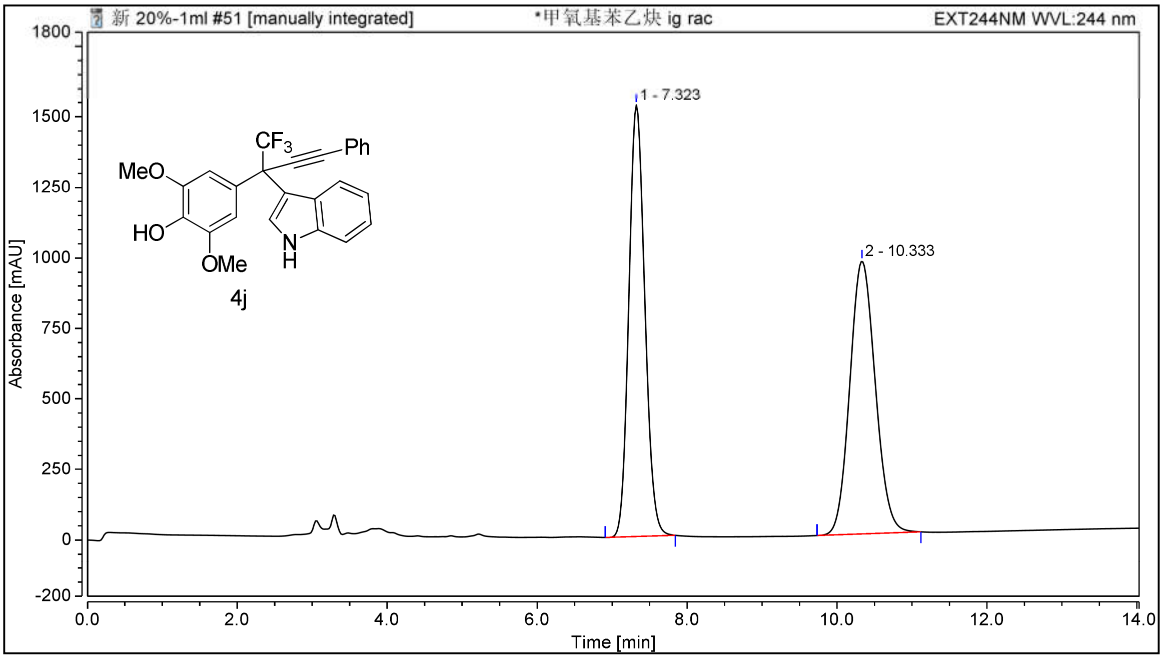

Integration Results

No.	Peak Name	Retention Time<br>min	Area<br>mAU*min	Relative Area<br>%	Amount<br>n.a.
1		7.323	377.472	49.78	n.a.
2		10.333	380.820	50.22	n.a.
**Total:**			**758.292**	**100.00**	

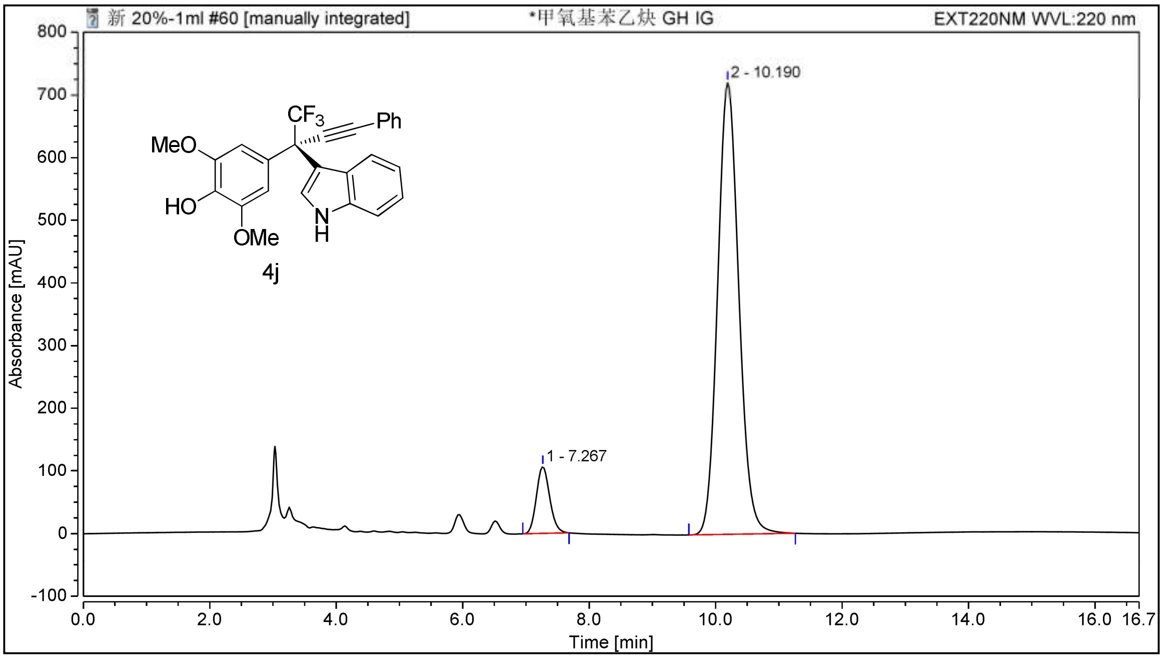

Integration Results

No.	Peak Name	Retention Time<br>min	Area<br>mAU*min	Relative Area<br>%	Amount<br>n.a.
1		7.267	25.886	8.48	n.a.
2		10.190	279.245	91.52	n.a.
**Total:**			**305.131**	**100.00**	

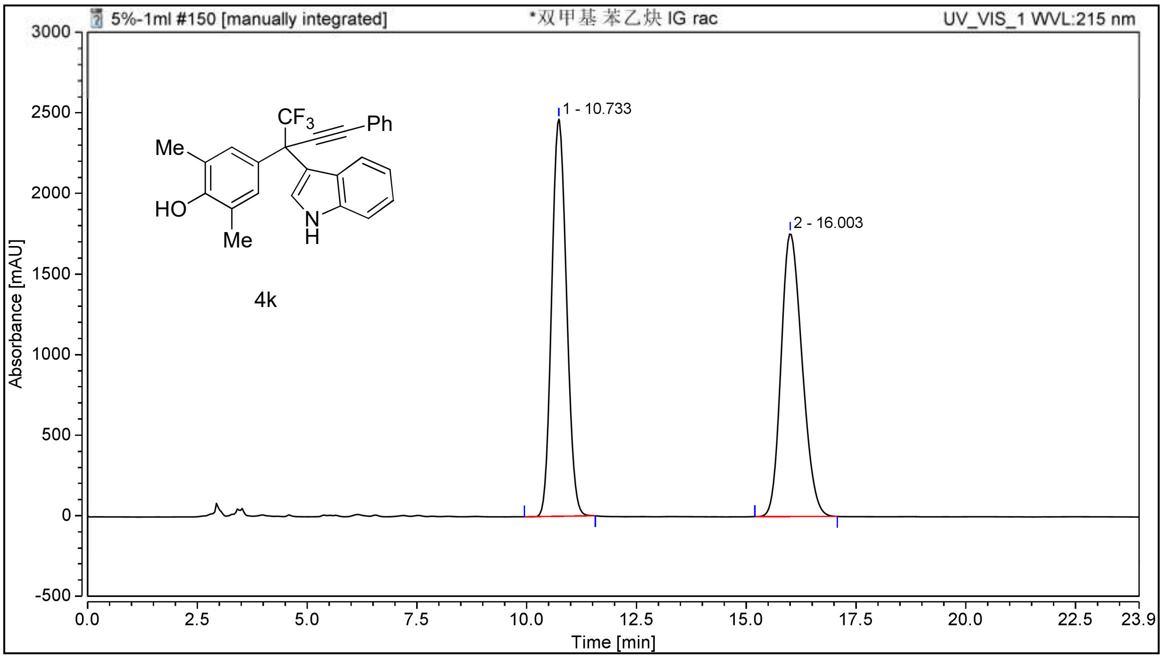

Integration Results

No.	Peak Name	Retention Time<br>min	Area<br>mAU*min	Relative Area<br>%	Amount<br>n.a.
1		10.733	964.219	49.75	n.a.
2		16.003	973.902	50.25	n.a.
**Total:**			**1938.121**	**100.00**	

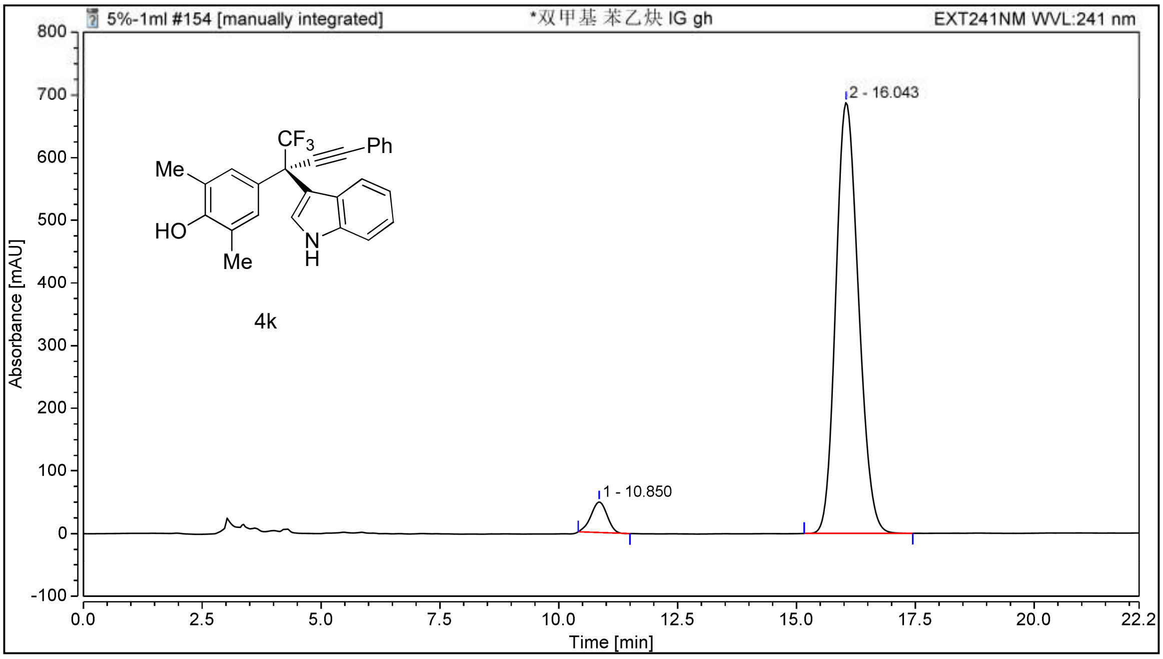

Integration Results

No.	Peak Name	Retention Time<br>min	Area<br>mAU*min	Relative Area<br>%	Amount<br>n.a.
1		10.850	19.031	4.76	n.a.
2		16.043	380.653	95.24	n.a.
**Total:**			**399.684**	**100.00**	

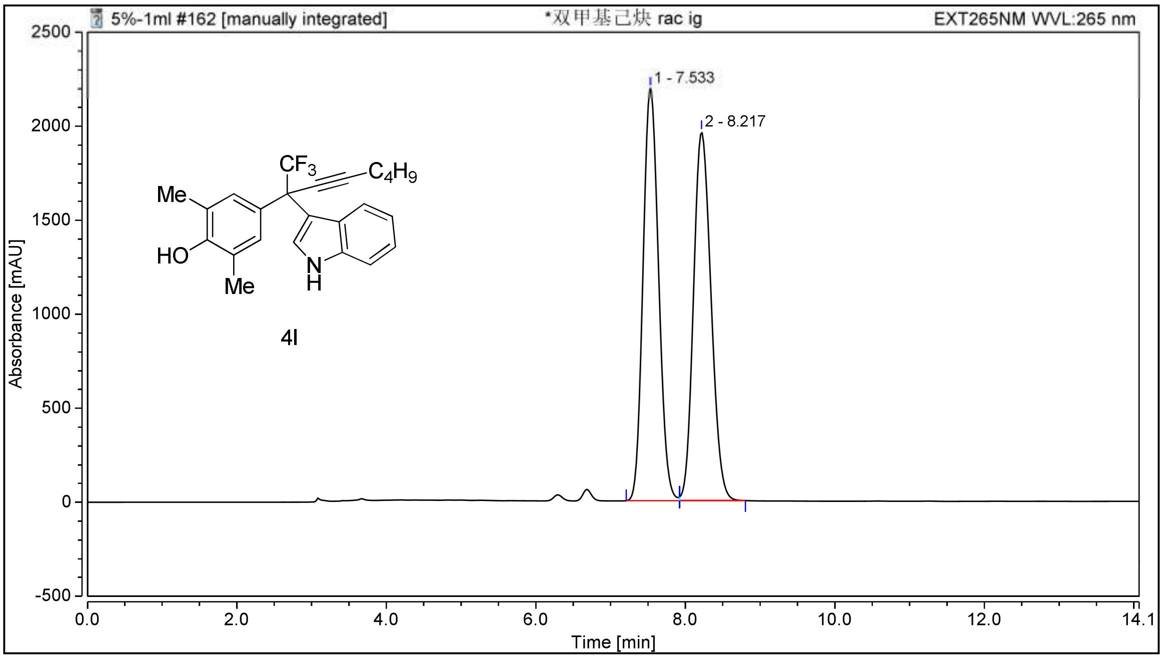

Integration Results

No.	Peak Name	Retention Time<br>min	Area<br>mAU*min	Relative Area<br>%	Amount<br>n.a.
1		7.533	529.244	49.84	n.a.
2		8.217	532.691	50.16	n.a.
**Total:**			**1061.935**	**100.00**	

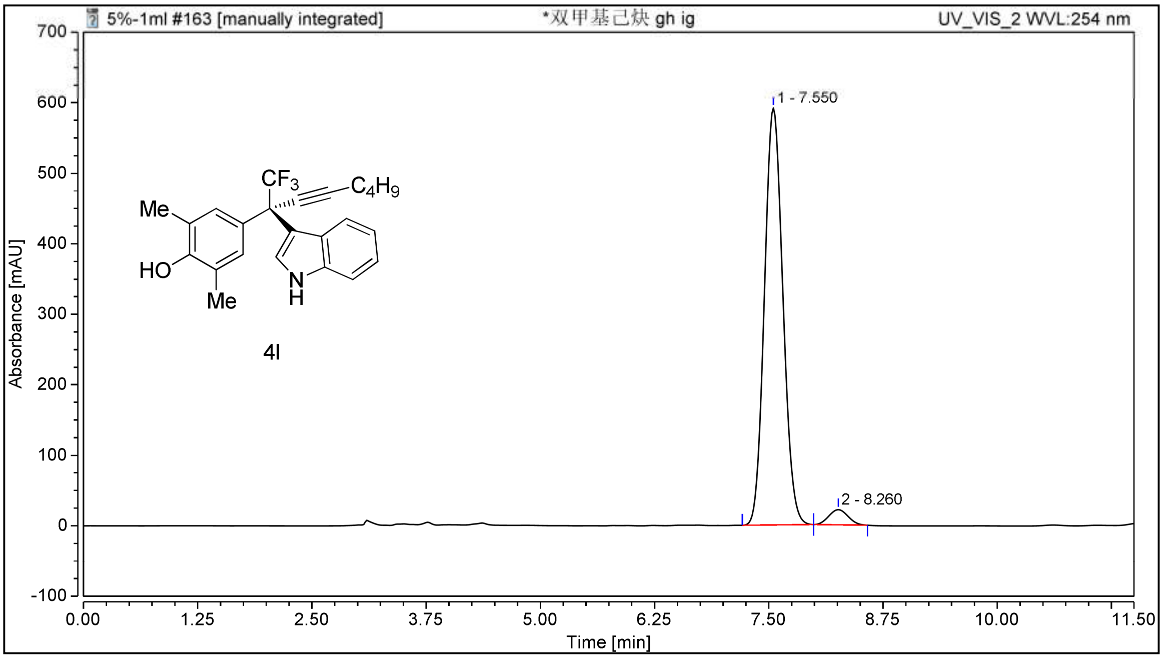

Integration Results

No.	Peak Name	Retention Time<br>min	Area<br>mAU*min	Relative Area<br>%	Amount<br>n.a.
1		7.550	138.246	96.35	n.a.
2		8.260	5.232	3.65	n.a.
**Total:**			**143.478**	**100.00**	

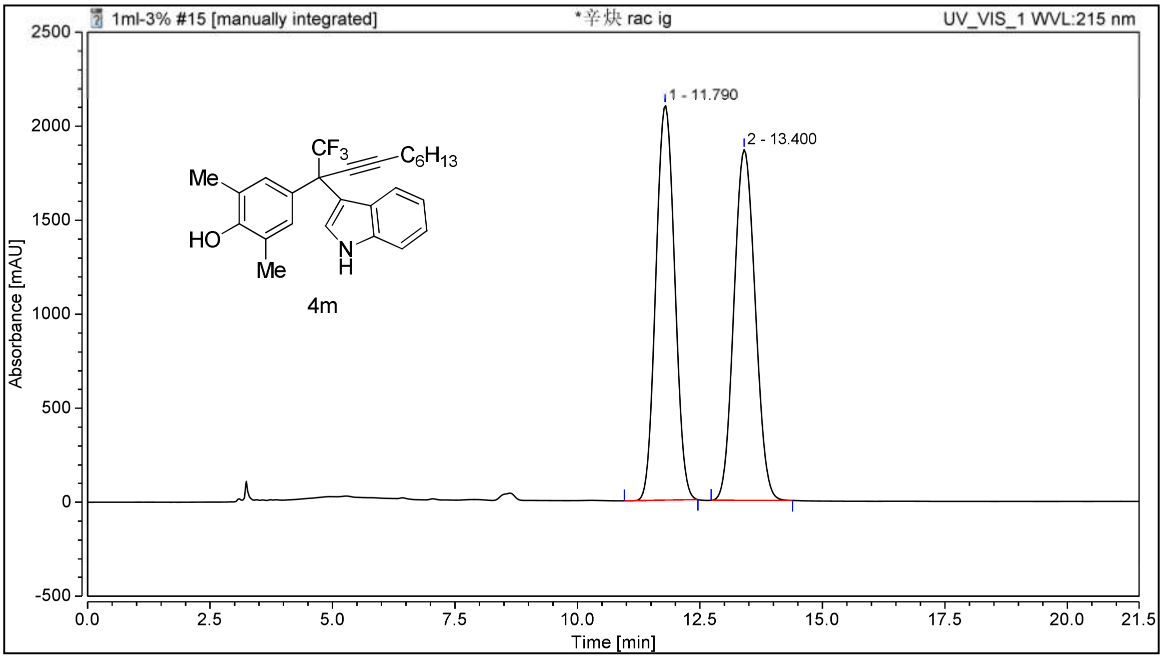

Integration Results

No.	Peak Name	Retention Time<br>min	Area<br>mAU*min	Relative Area<br>%	Amount<br>n.a.
1		11.790	936.221	49.93	n.a.
2		13.400	938.747	50.07	n.a.
**Total:**			**1874.968**	**100.00**	

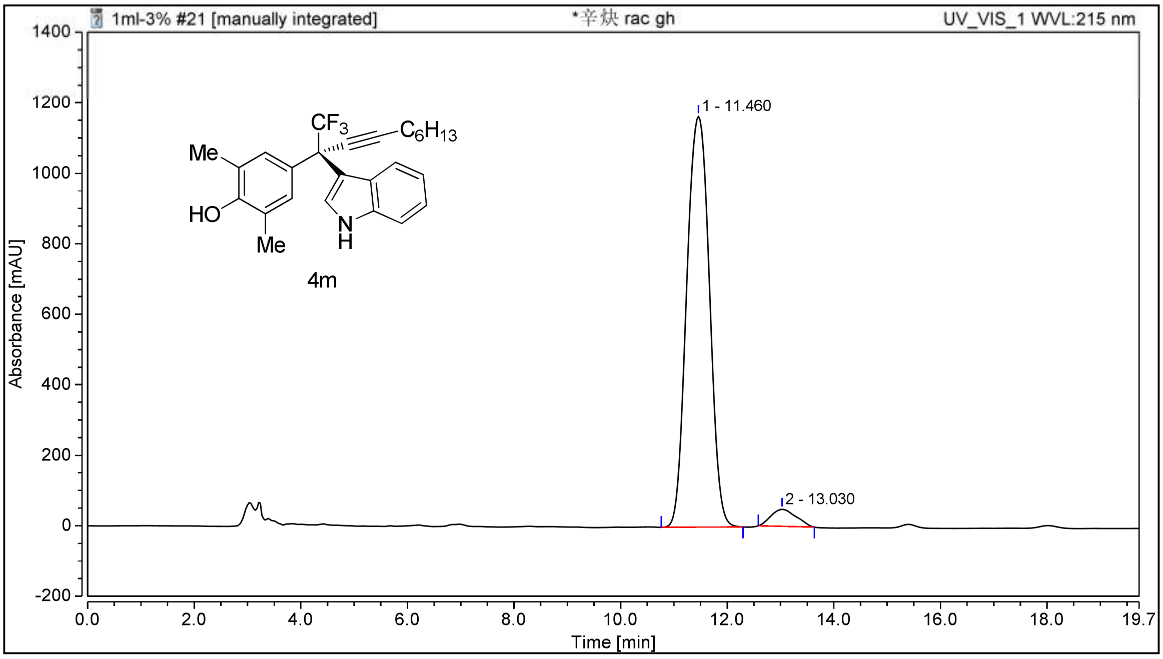

Integration Results

No.	Peak Name	Retention Time<br>min	Area<br>mAU*min	Relative Area<br>%	Amount<br>n.a.
1		11.460	574.595	95.62	n.a.
2		13.030	26.346	4.38	n.a.
**Total:**			**600.941**	**100.00**	

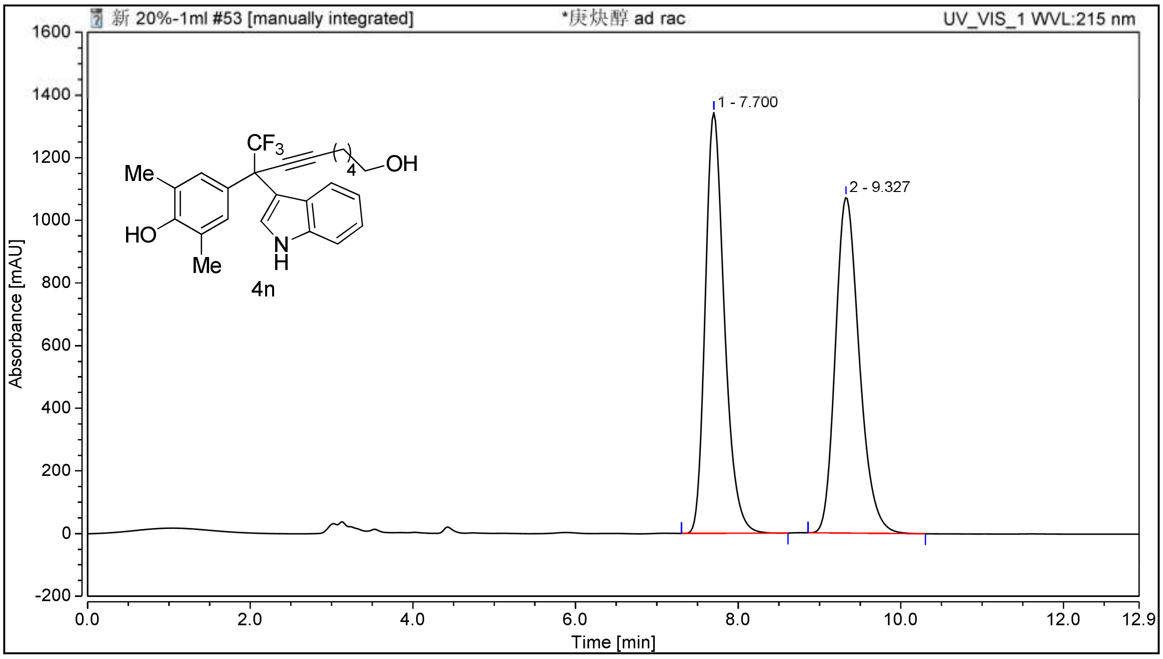

Integration Results

No.	Peak Name	Retention Time<br>min	Area<br>mAU*min	Relative Area<br>%	Amount<br>n.a.
1		7.700	365.330	50.02	n.a.
2		9.327	365.062	49.98	n.a.
**Total:**			**730.392**	**100.00**	

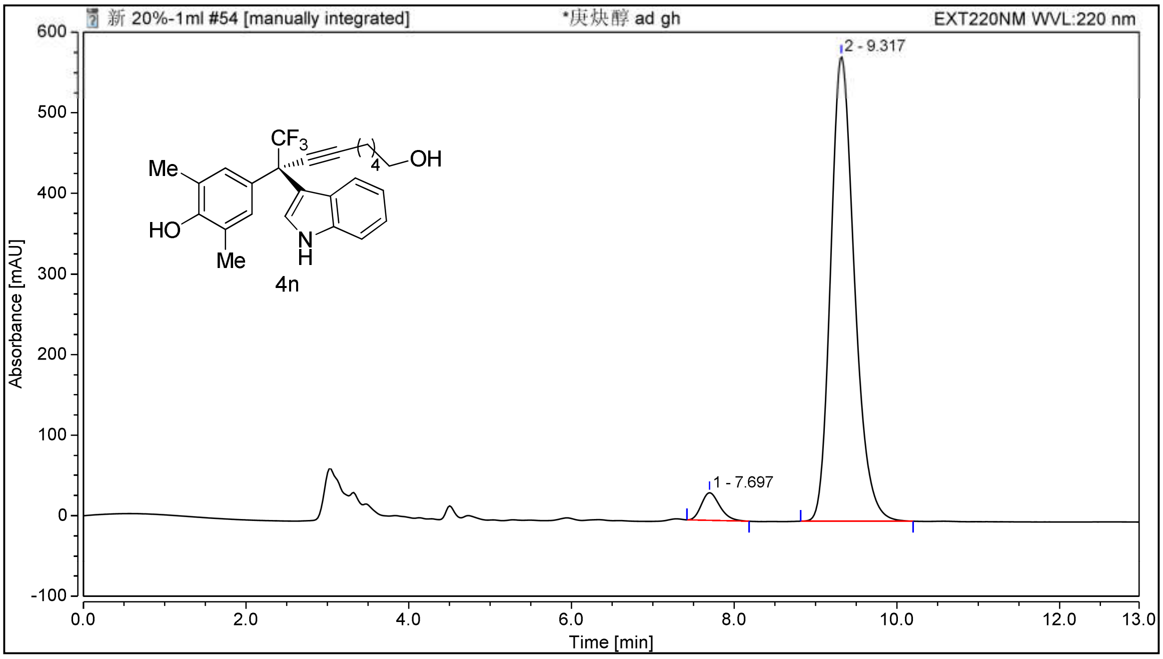

Integration Results

No.	Peak Name	Retention Time<br>min	Area<br>mAU*min	Relative Area<br>%	Amount<br>n.a.
1		7.697	8.953	4.39	n.a.
2		9.317	195.187	95.61	n.a.
**Total:**			**204.140**	**100.00**	

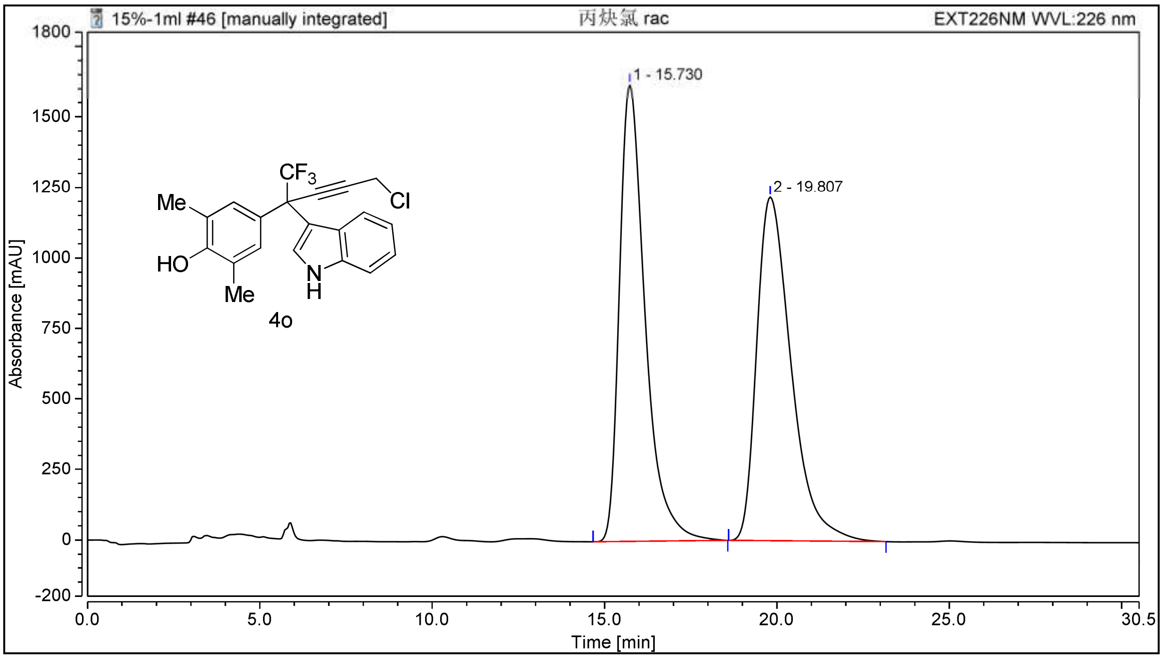

Integration Results

No.	Peak Name	Retention Time<br>min	Area<br>mAU*min	Relative Area<br>%	Amount<br>n.a.
1		15.730	1396.735	49.81	n.a.
2		19.807	1407.295	50.19	n.a.
**Total:**			**2804.030**	**100.00**	

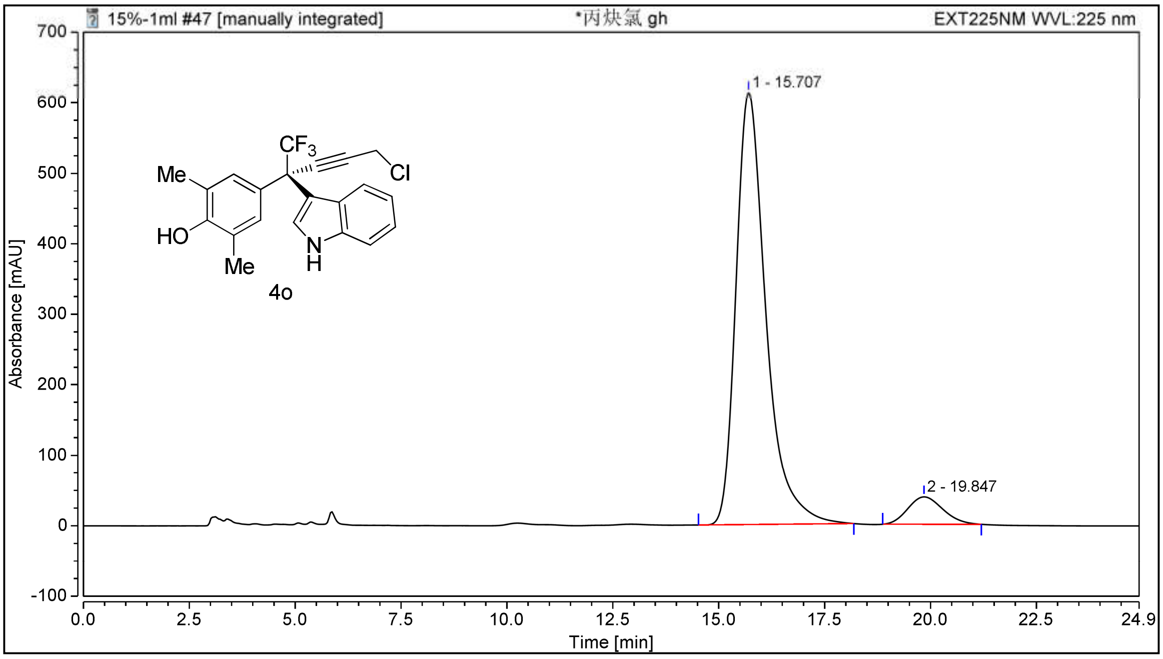

No.	Peak Name	Retention Time<br>min	Area<br>mAU*min	Relative Area<br>%	Amount<br>n.a.
1		15.707	500.964	93.11	n.a.
2		19.847	37.084	6.89	n.a.
**Total:**			**538.048**	**100.00**	

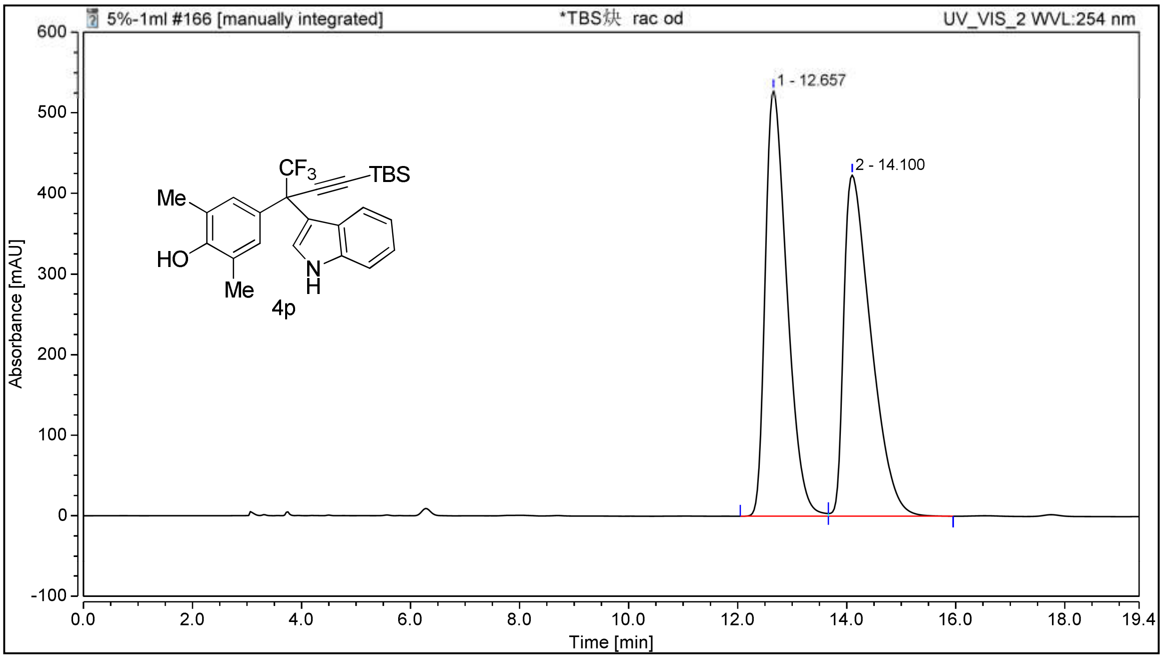

Integration Results

No.	Peak Name	Retention Time<br>min	Area<br>mAU*min	Relative Area<br>%	Amount<br>n.a.
1		12.657	244.239	49.94	n.a.
2		14.100	244.830	50.06	n.a.
**Total:**			**489.069**	**100.00**	

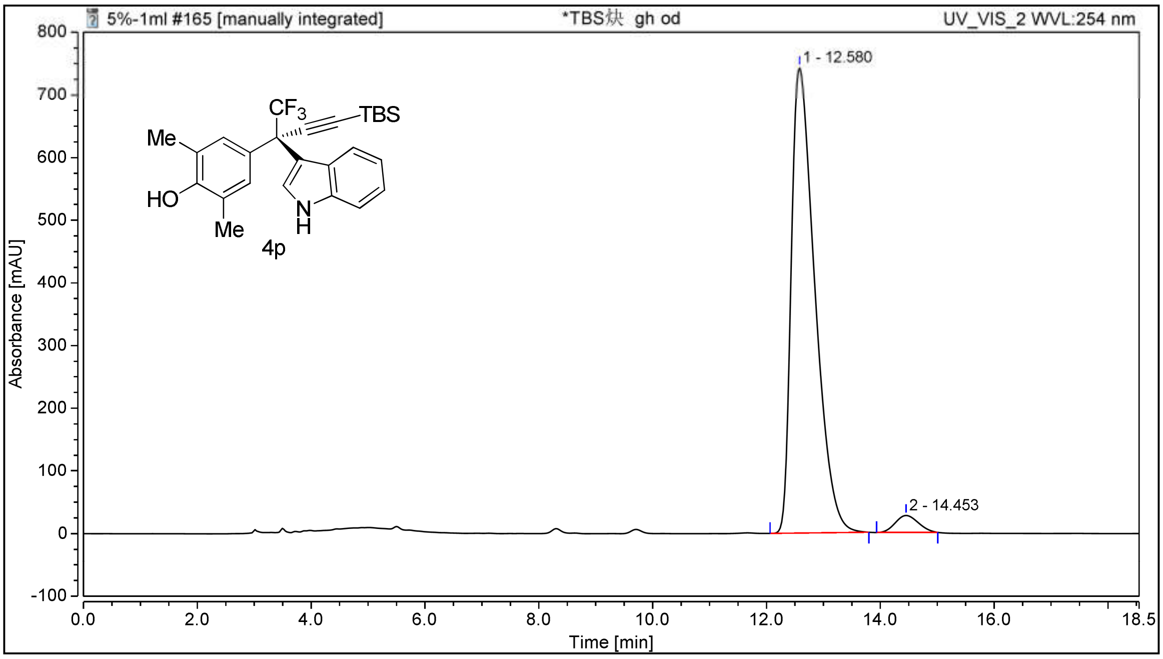

Integration Results

No.	Peak Name	Retention Time<br>min	Area<br>mAU*min	Relative Area<br>%	Amount<br>n.a.
1		12.580	355.461	96.57	n.a.
2		14.453	12.629	3.43	n.a.
**Total:**			**368.091**	**100.00**	

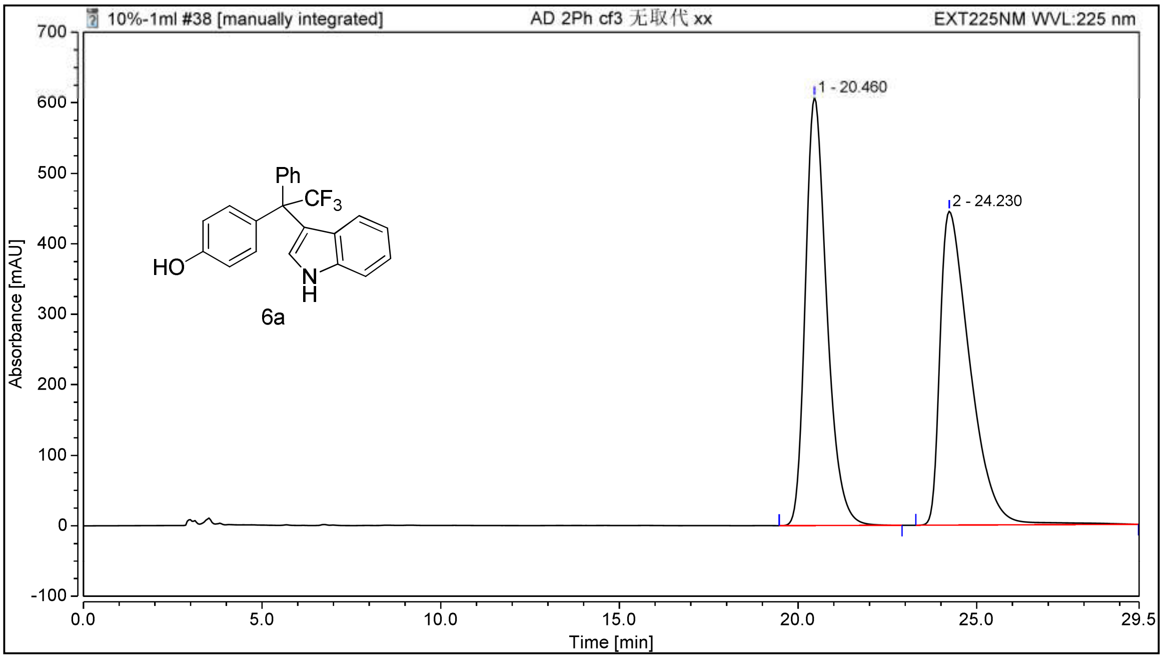

Integration Results

No.	Peak Name	Retention Time<br>min	Area<br>mAU*min	Relative Area<br>%	Amount<br>n.a.
1		20.460	421.820	49.43	n.a.
2		24.230	431.583	50.57	n.a.
**Total:**			**853.403**	**100.00**	

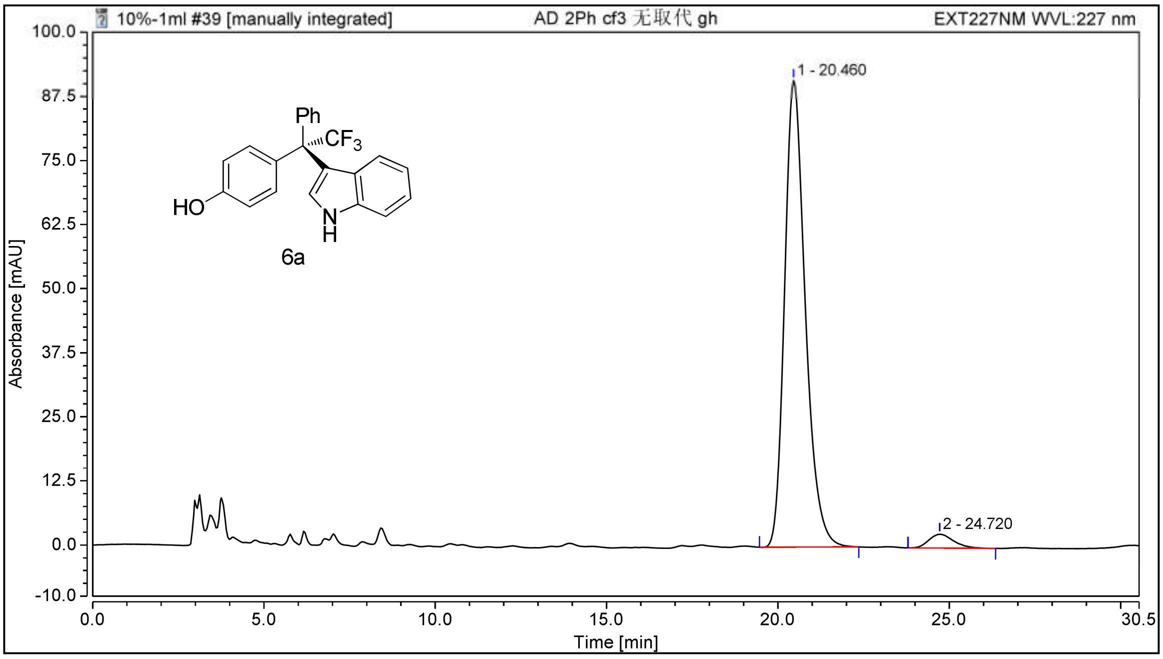

Integration Results

No.	Peak Name	Retention Time<br>min	Area<br>mAU*min	Relative Area<br>%	Amount<br>n.a.
1		20.460	63.696	96.59	n.a.
2		24.720	2.247	3.41	n.a.
**Total:**			**65.943**	**100.00**	

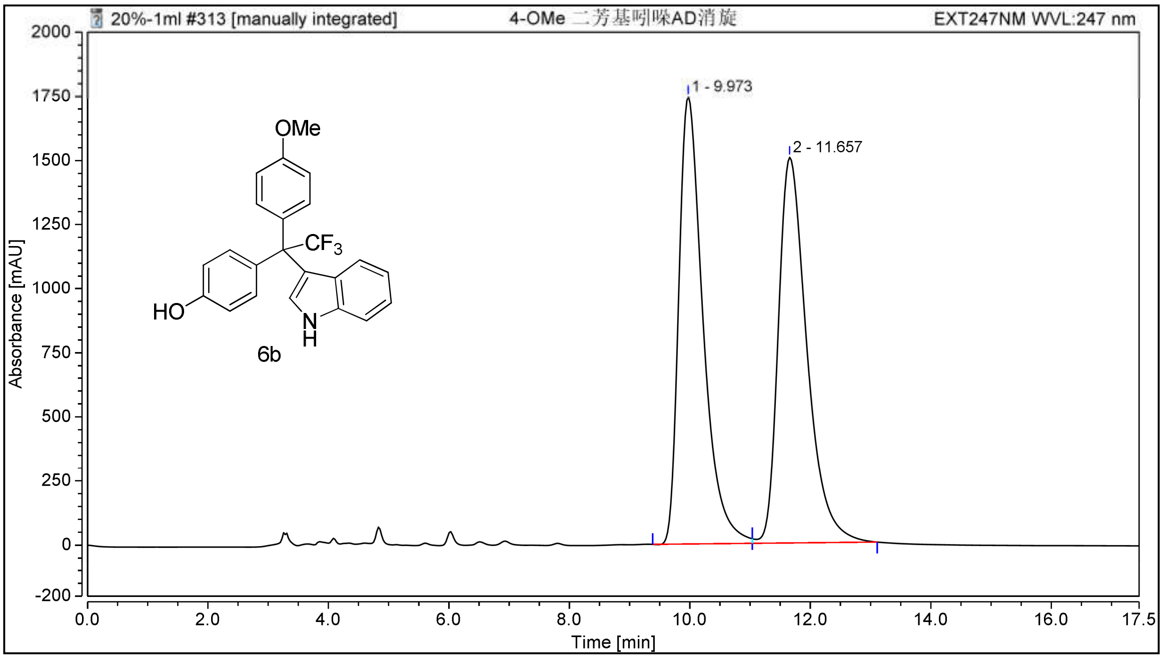

Integration Results

No.	Peak Name	Retention Time<br>min	Area<br>mAU*min	Relative Area<br>%	Amount<br>n.a.
1		9.973	811.399	49.57	n.a.
2		11.657	825.485	50.43	n.a.
**Total:**			**1636.884**	**100.00**	

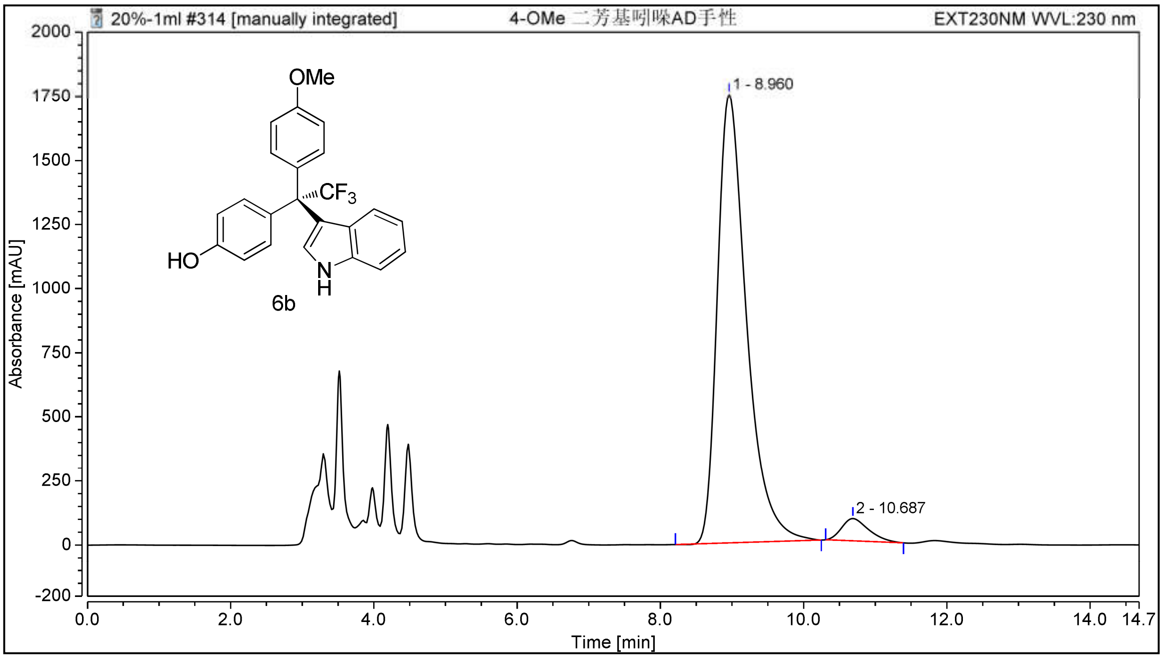

Integration Results

No.	Peak Name	Retention Time<br>min	Height<br>mAU	Relative Area<br>%	Amount<br>n.a.
1		8.960	1748.946	95.59	n.a.
2		10.687	86.571	4.41	n.a.
**Total:**			**1835.517**	**100.00**	

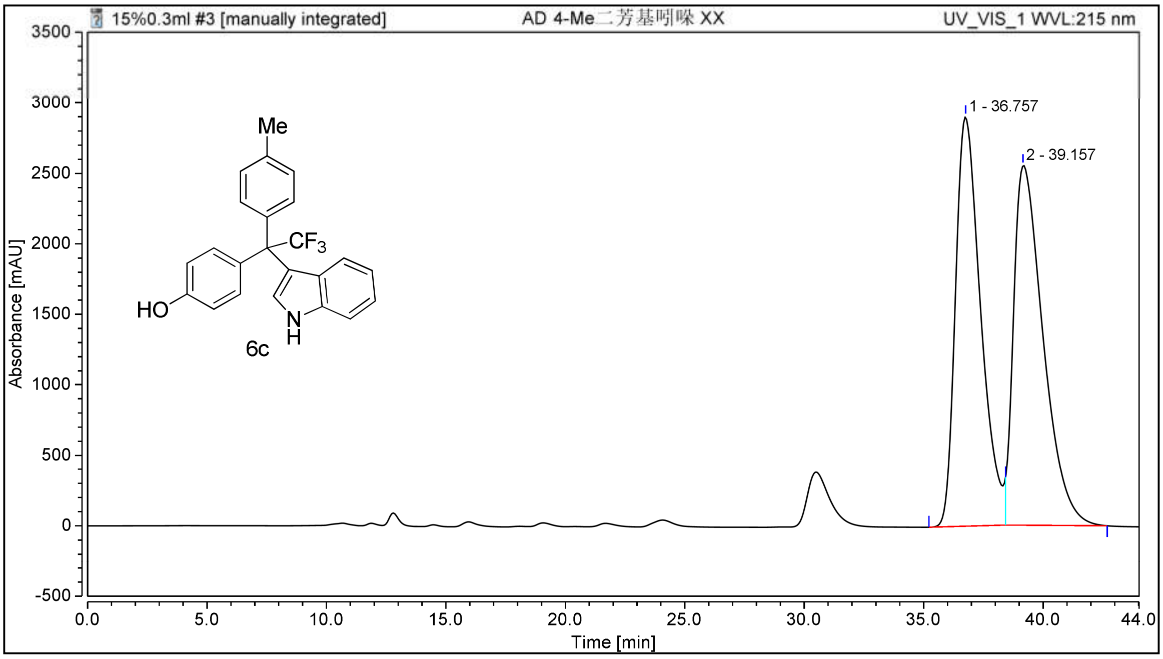

Integration Results

No.	Peak Name	Retention Time min	Area mAU*min	Relative Area %	Amount n.a.
1		36.757	3557.996	49.40	n.a.
2		39.157	3644.874	50.60	n.a.
**Total:**			**7202.870**	**100.00**	

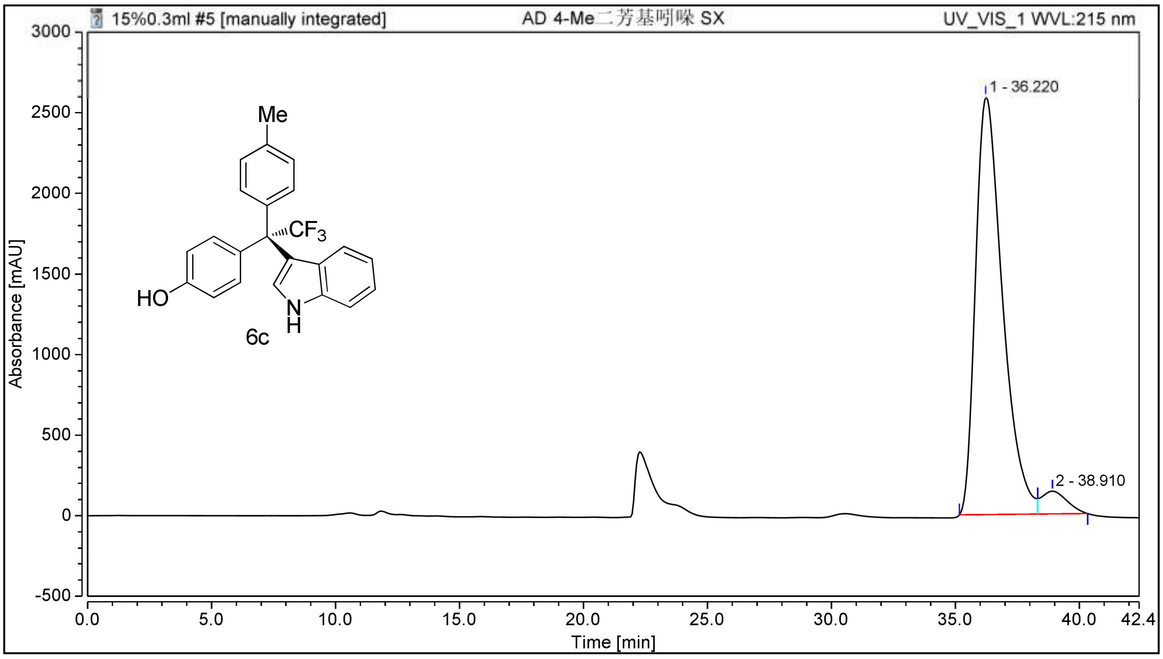

Integration Results

No.	Peak Name	Retention Time min	Area mAU*min	Relative Area %	Amount n.a.
1		36.220	3268.815	95.14	n.a.
2		38.910	166.839	4.86	n.a.
**Total:**			**3435.654**	**100.00**	

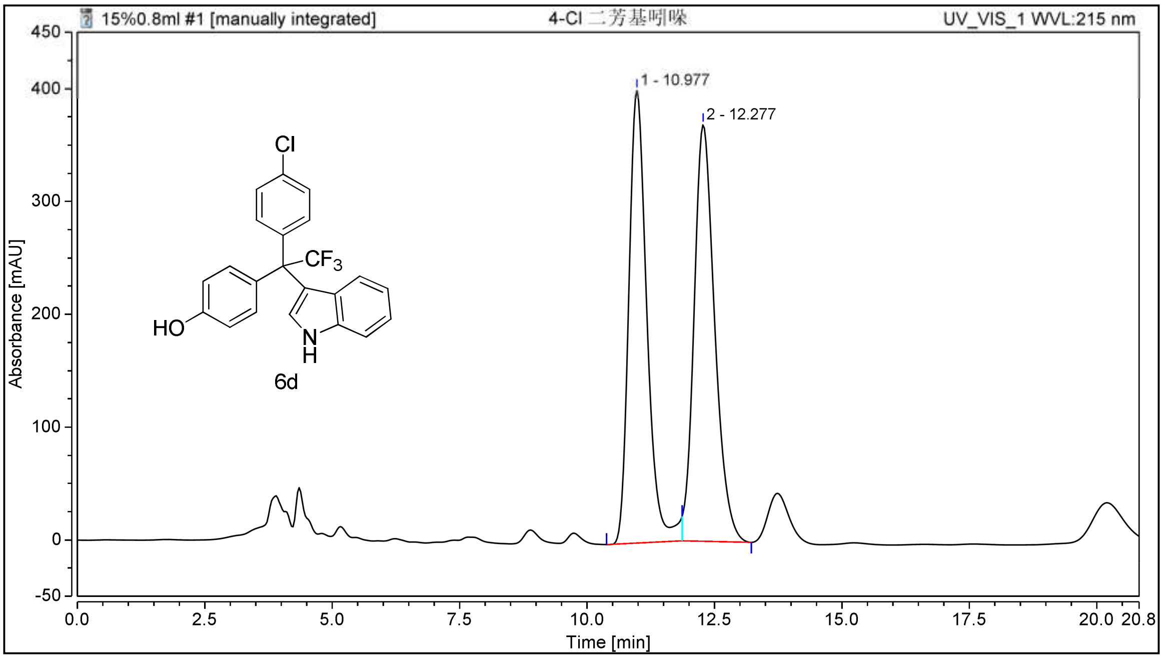

Integration Results					
No.	Peak Name	Retention Time<br>min	Area<br>mAU*min	Relative Area<br>%	Amount<br>n.a.
1		10.977	164.824	48.79	n.a.
2		12.277	172.996	51.21	n.a.
**Total:**			**337.820**	**100.00**	

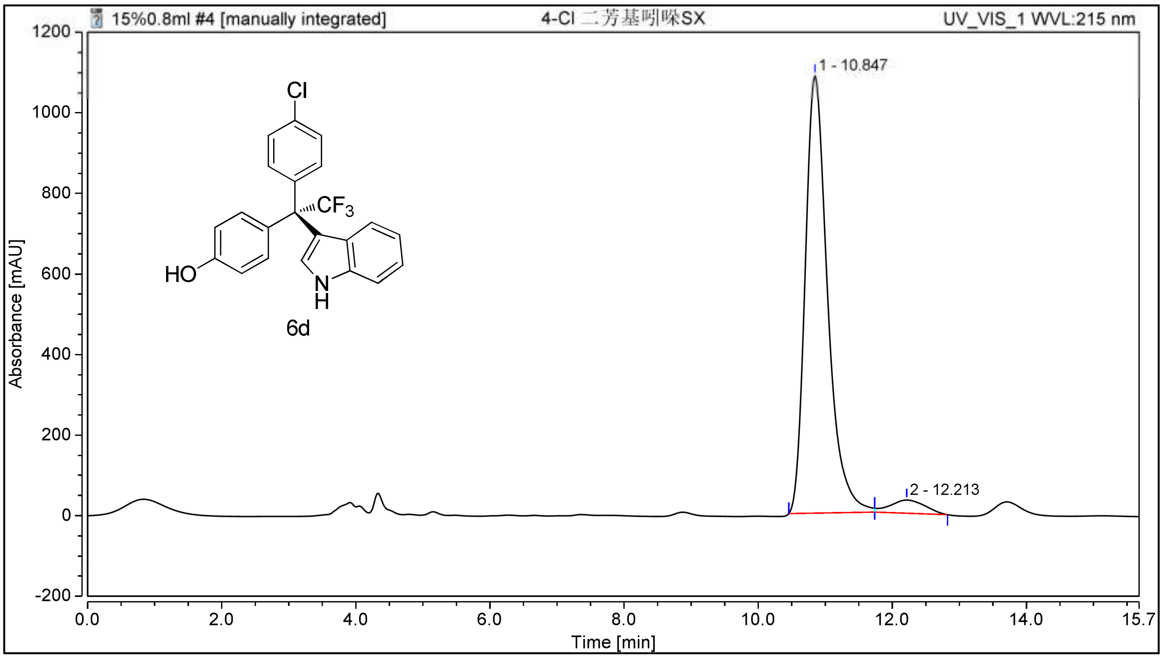

Integration Results					
No.	Peak Name	Retention Time<br>min	Area<br>mAU*min	Relative Area<br>%	Amount<br>n.a.
1		10.847	419.180	95.50	n.a.
2		12.213	19.764	4.50	n.a.
**Total:**			**438.944**	**100.00**	

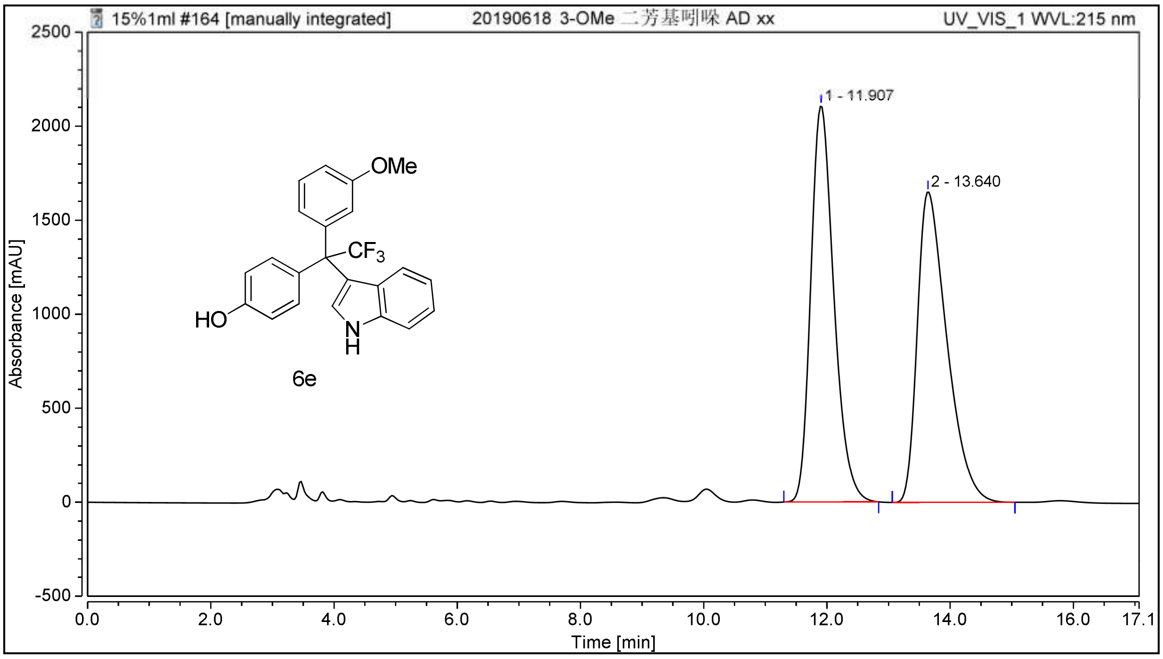

Integration Results

No.	Peak Name	Retention Time<br>min	Area<br>mAU*min	Relative Area<br>%	Amount<br>n.a.
1		11.907	915.139	49.93	n.a.
2		13.640	917.875	50.07	n.a.
**Total:**			**1833.014**	**100.00**	

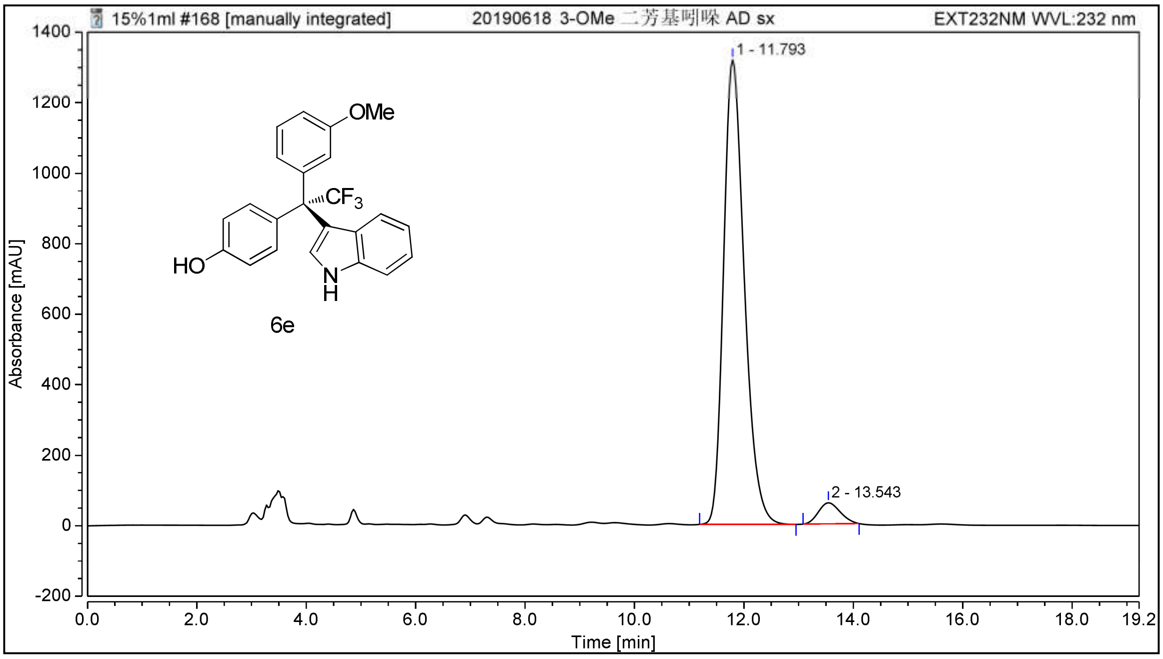

Integration Results

No.	Peak Name	Retention Time<br>min	Area<br>mAU*min	Relative Area<br>%	Amount<br>n.a.
1		11.793	578.687	95.48	n.a.
2		13.543	27.396	4.52	n.a.
**Total:**			**606.083**	**100.00**	

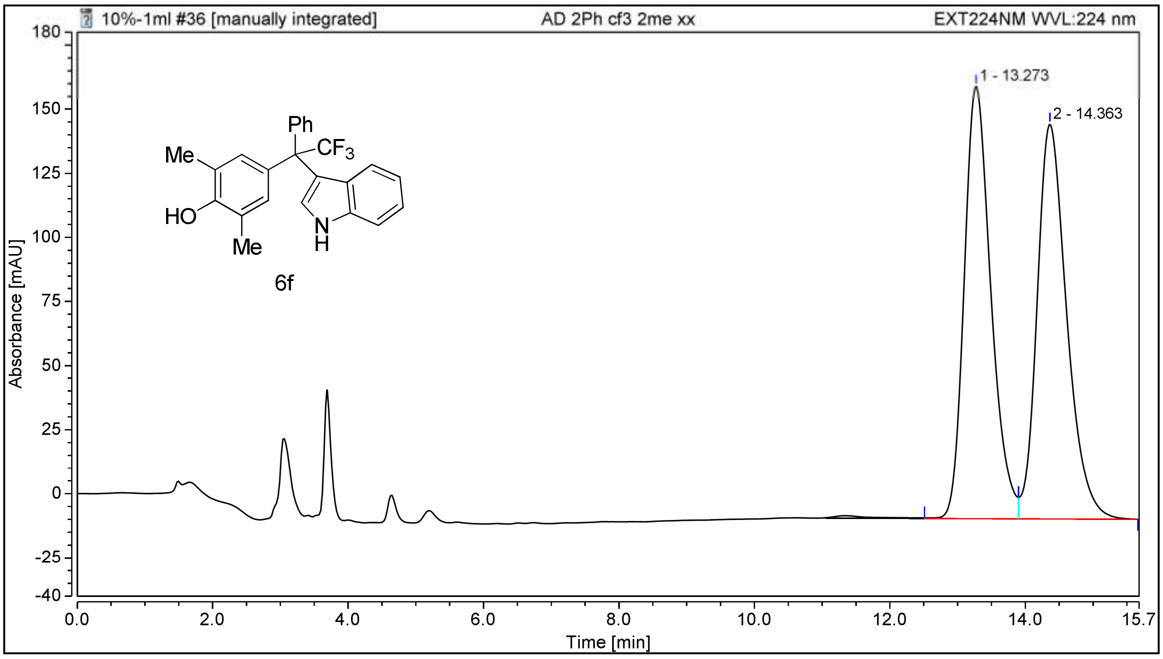

Integration Results

No.	Peak Name	Retention Time<br>min	Area<br>mAU*min	Relative Area<br>%	Amount<br>n.a.
1		13.273	76.886	49.62	n.a.
2		14.363	78.078	50.38	n.a.
**Total:**			**154.965**	**100.00**	

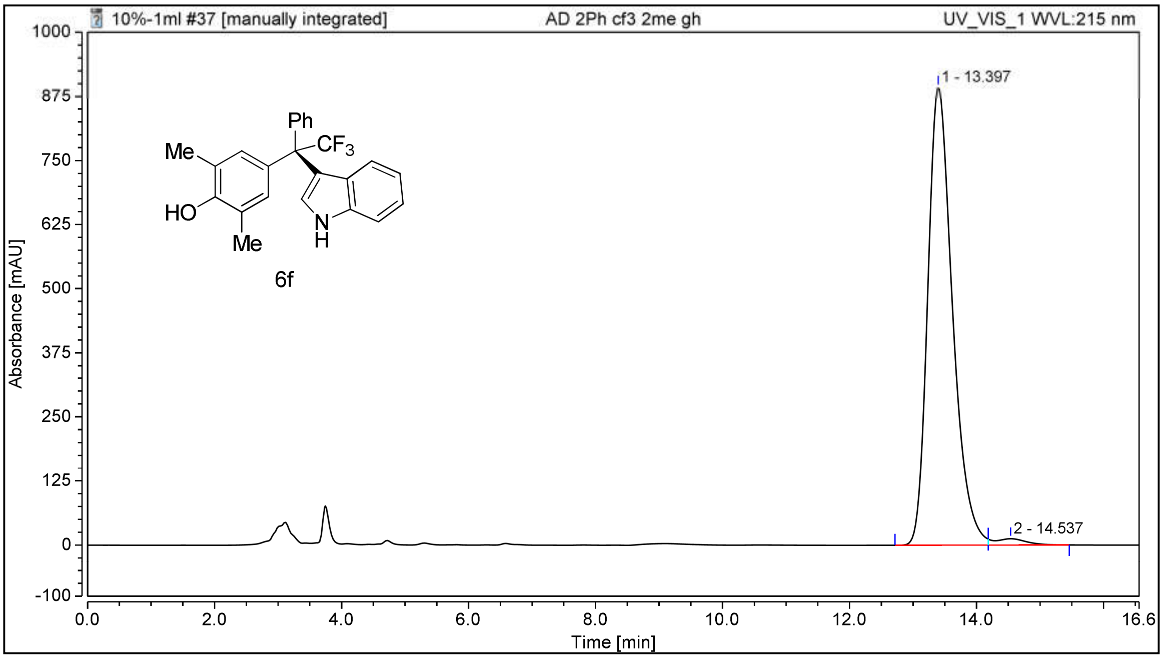

Integration Results

No.	Peak Name	Retention Time<br>min	Area<br>mAU*min	Relative Area<br>%	Amount<br>n.a.
1		13.397	401.569	98.24	n.a.
2		14.537	7.192	1.76	n.a.
**Total:**			**408.761**	**100.00**	

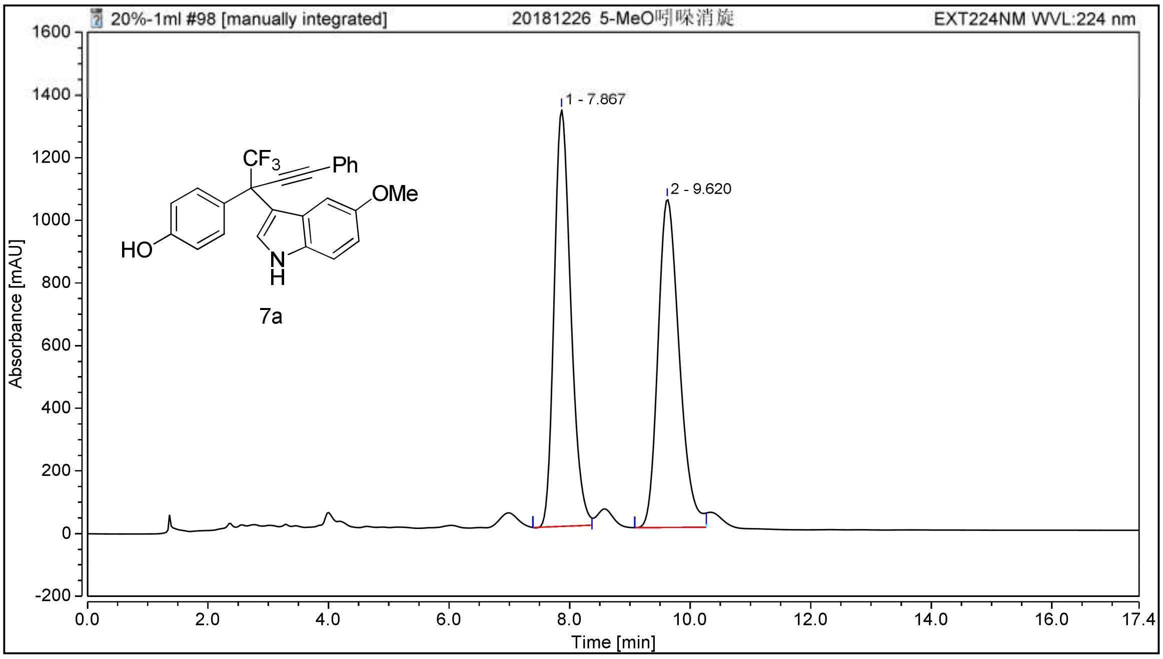

Integration Results

No.	Peak Name	Retention Time<br>min	Area<br>mAU*min	Relative Area<br>%	Amount<br>n.a.
1		7.867	424.934	49.75	n.a.
2		9.620	429.137	50.25	n.a.
**Total:**			**854.071**	**100.00**	

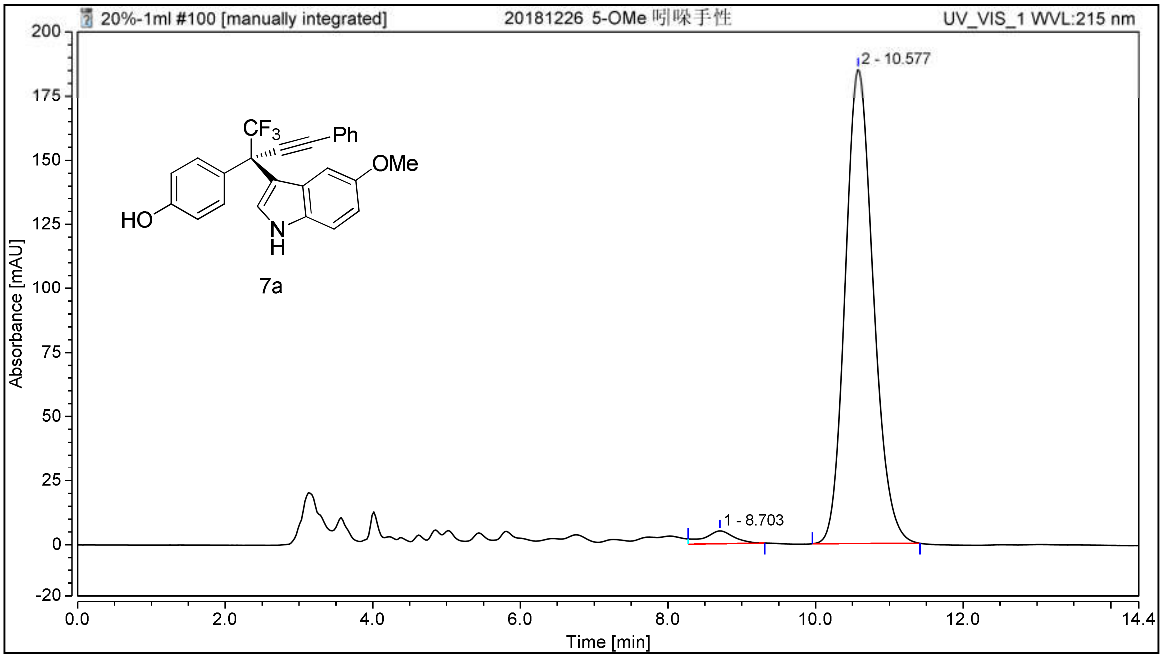

Integration Results

No.	Peak Name	Retention Time<br>min	Area<br>mAU*min	Relative Area<br>%	Amount<br>n.a.
1		8.703	1.948	2.33	n.a.
2		10.577	81.678	97.67	n.a.
**Total:**			**83.626**	**100.00**	

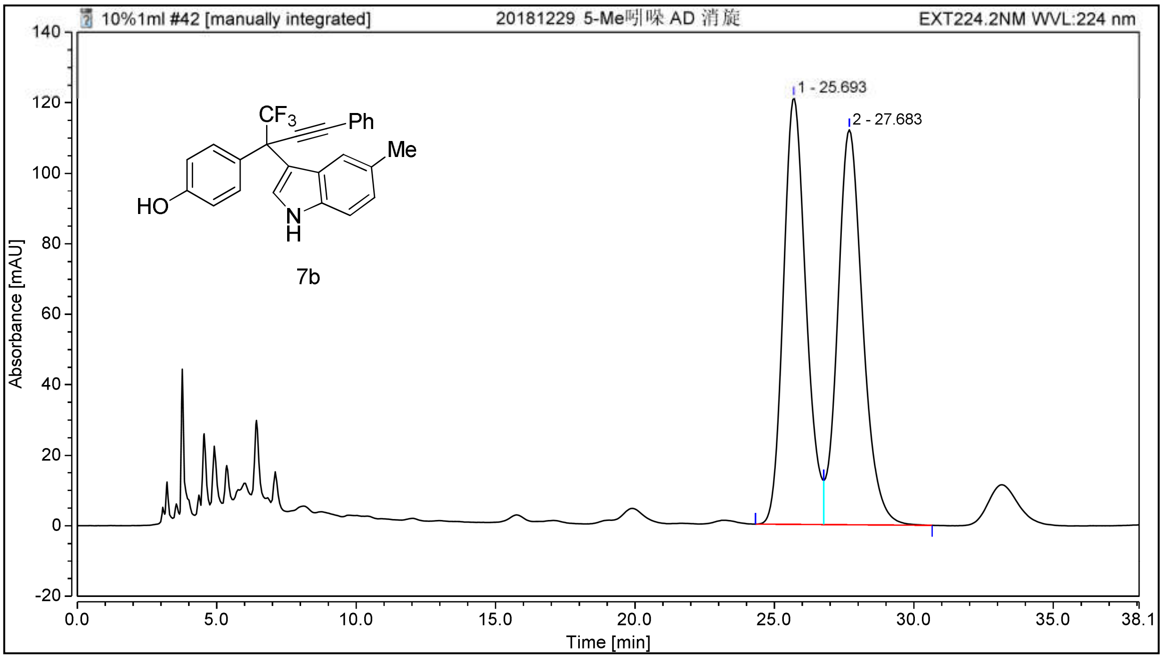

Integration Results

No.	Peak Name	Retention Time min	Area mAU*min	Relative Area %	Amount n.a.
1		25.693	113.412	49.31	n.a.
2		27.683	116.578	50.69	n.a.
**Total:**			**229.990**	**100.00**	

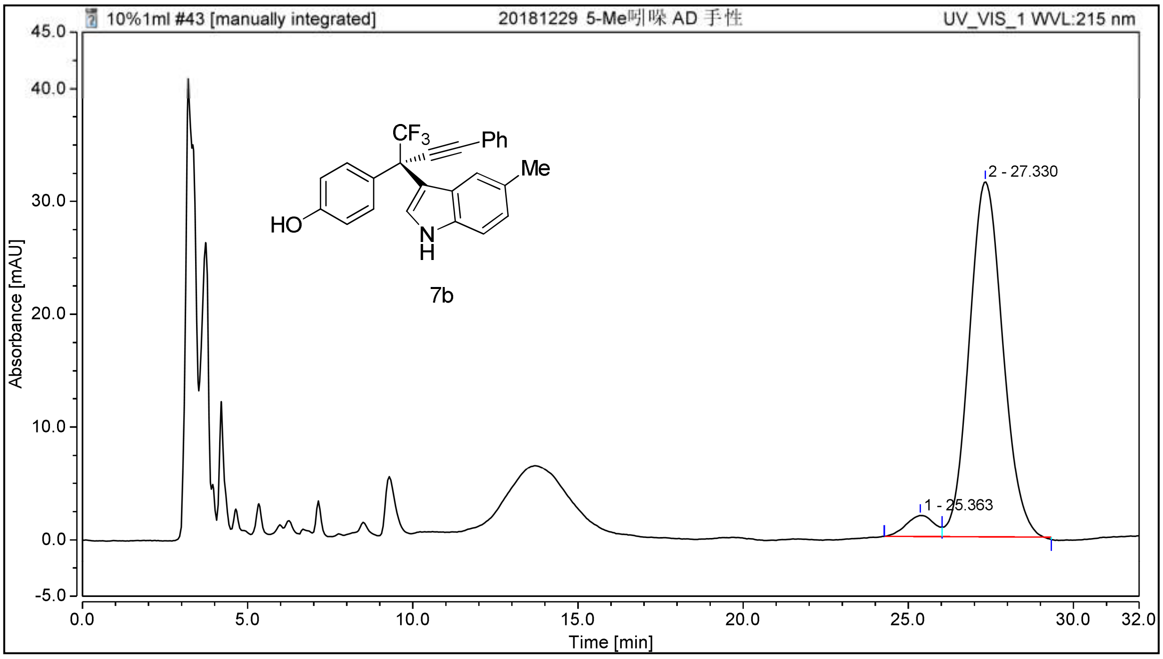

Integration Results

No.	Peak Name	Retention Time min	Area mAU*min	Relative Area %	Amount n.a.
1		25.420	1.237	4.88	n.a.
2		27.323	24.090	95.12	n.a.
**Total:**			**25.327**	**100.00**	

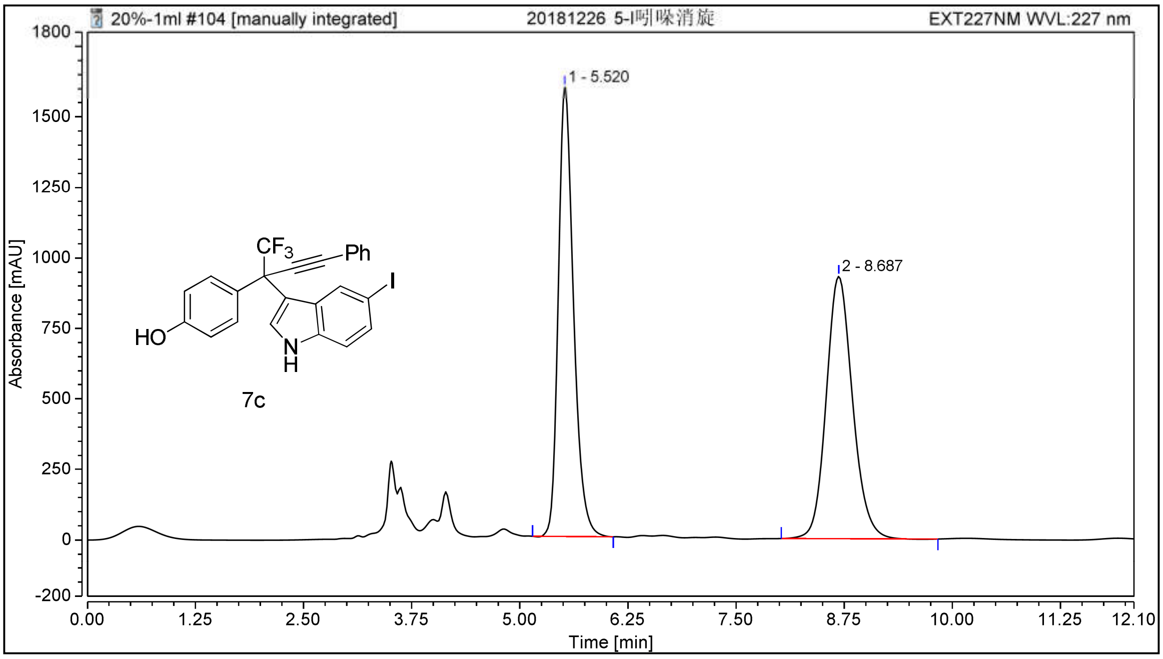

Integration Results					
No.	Peak Name	Retention Time<br>min	Area<br>mAU*min	Relative Area<br>%	Amount<br>n.a.
1		5.520	324.727	49.80	n.a.
2		8.687	327.276	50.20	n.a.
**Total:**			**652.003**	**100.00**	

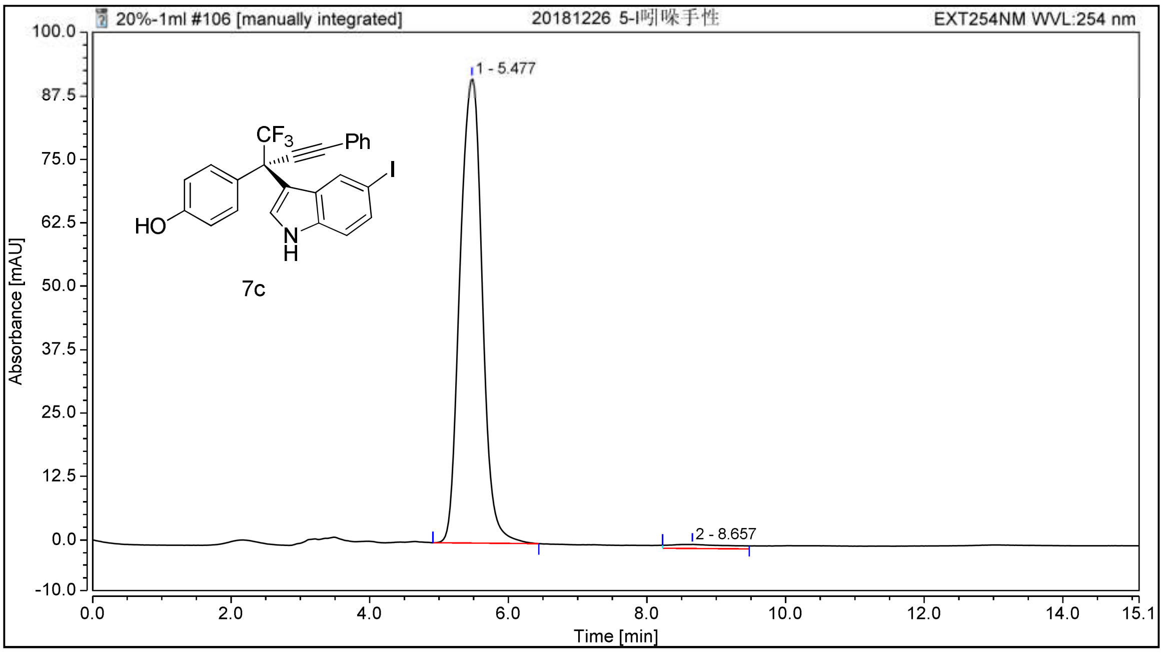

Integration Results					
No.	Peak Name	Retention Time<br>min	Area<br>mAU*min	Relative Area<br>%	Amount<br>n.a.
1		5.477	34.035	97.56	n.a.
2		8.657	0.850	2.44	n.a.
**Total:**			**34.885**	**100.00**	

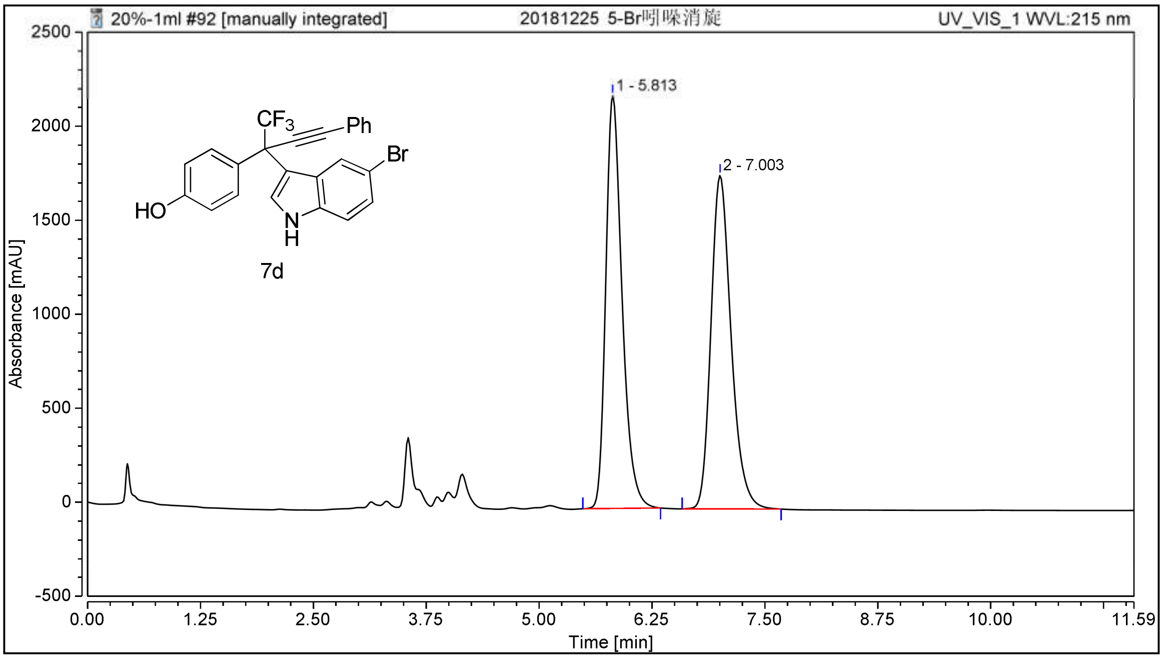

Integration Results

No.	Peak Name	Retention Time min	Area mAU*min	Relative Area %	Amount n.a.
1		5.813	458.454	49.99	n.a.
2		7.003	458.582	50.01	n.a.
**Total:**			**917.036**	**100.00**	

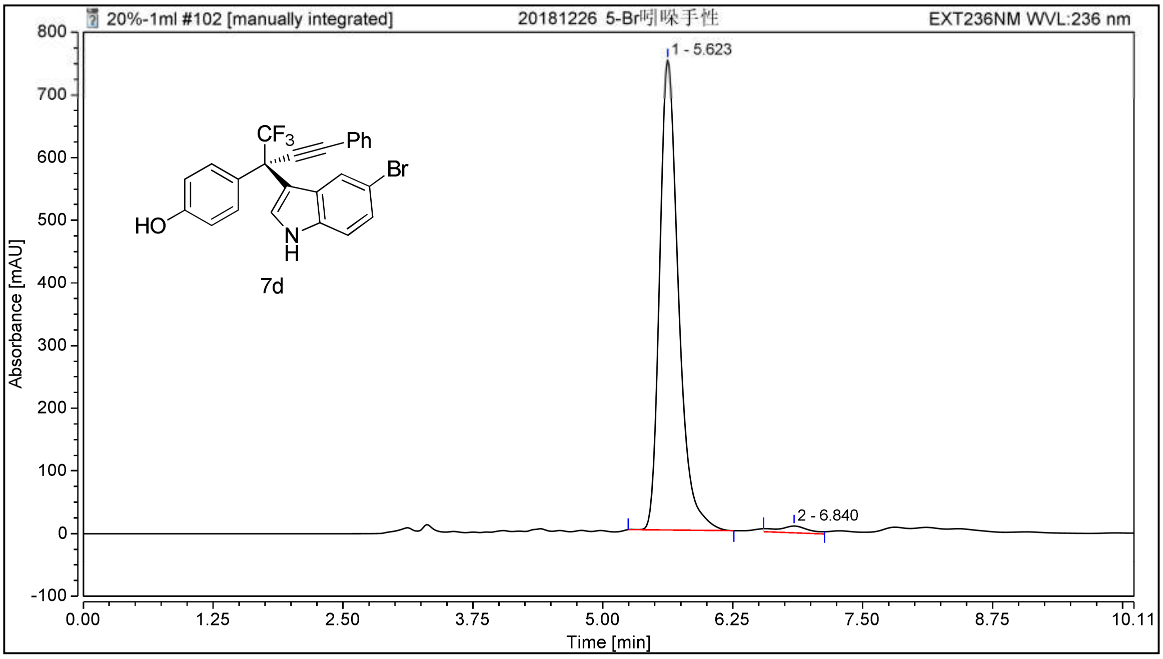

Integration Results

No.	Peak Name	Retention Time min	Area mAU*min	Relative Area %	Amount n.a.
1		5.623	157.889	97.68	n.a.
2		6.840	3.752	2.32	n.a.
**Total:**			**161.641**	**100.00**	

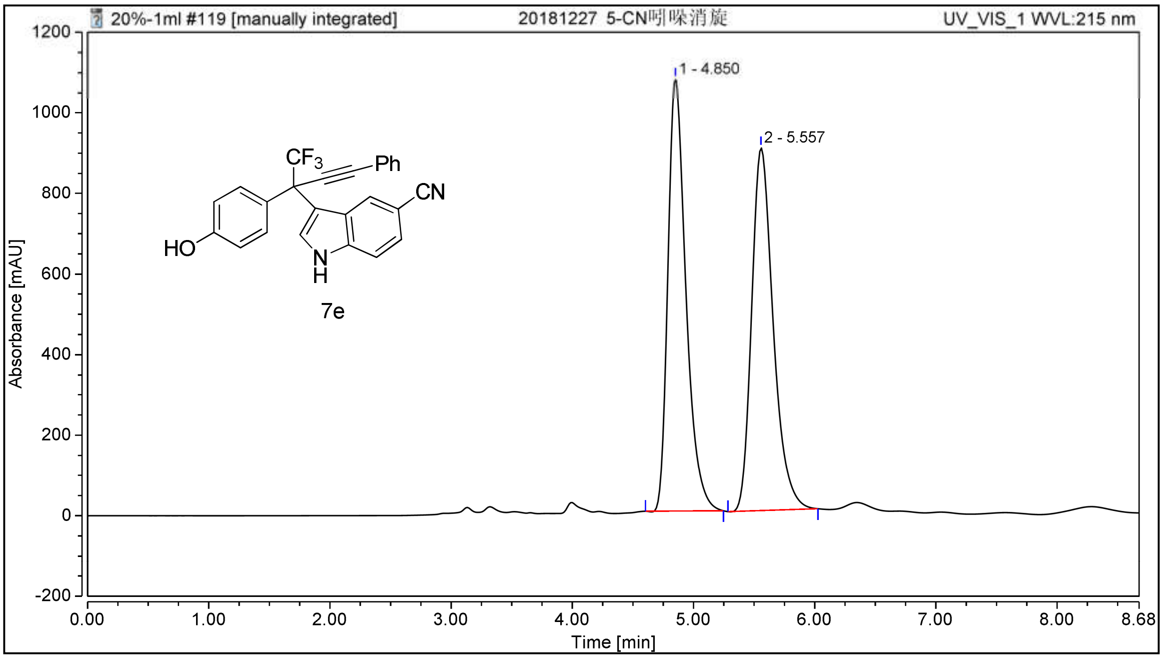

Integration Results					
No.	Peak Name	Retention Time<br>min	Area<br>mAU*min	Relative Area<br>%	Amount<br>n.a.
1		4.850	184.602	49.86	n.a.
2		5.557	185.611	50.14	n.a.
**Total:**			**370.213**	**100.00**	

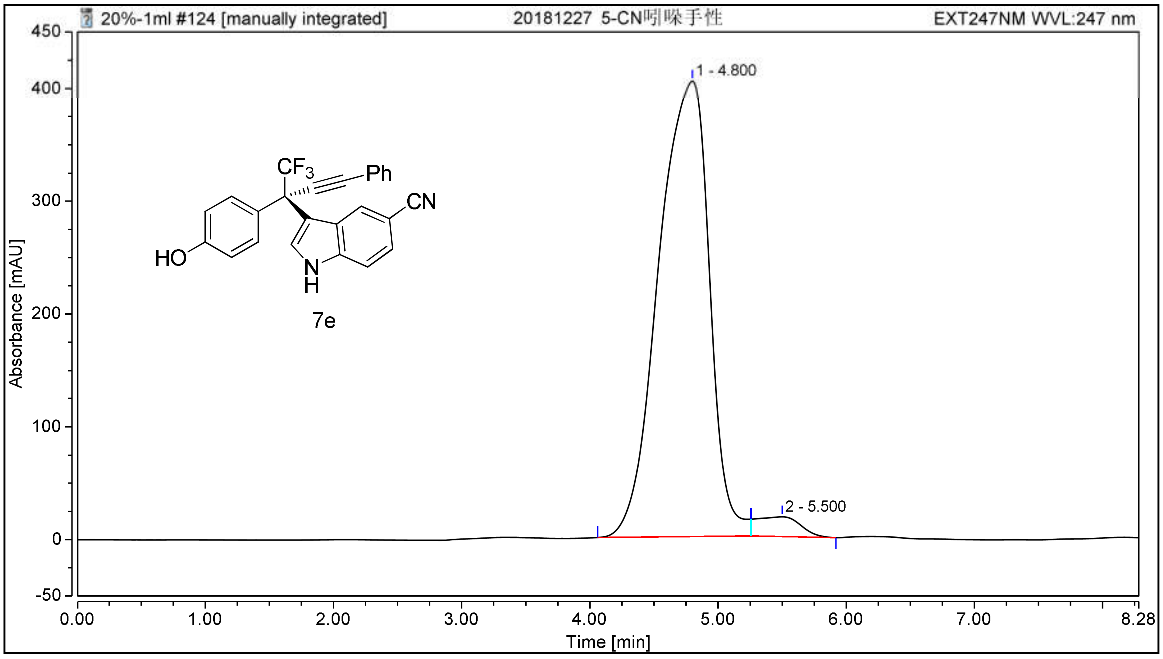

Integration Results					
No.	Peak Name	Retention Time<br>min	Area<br>mAU*min	Relative Area<br>%	Amount<br>n.a.
1		4.800	185.441	96.40	n.a.
2		5.500	6.921	3.60	n.a.
**Total:**			**192.362**	**100.00**	

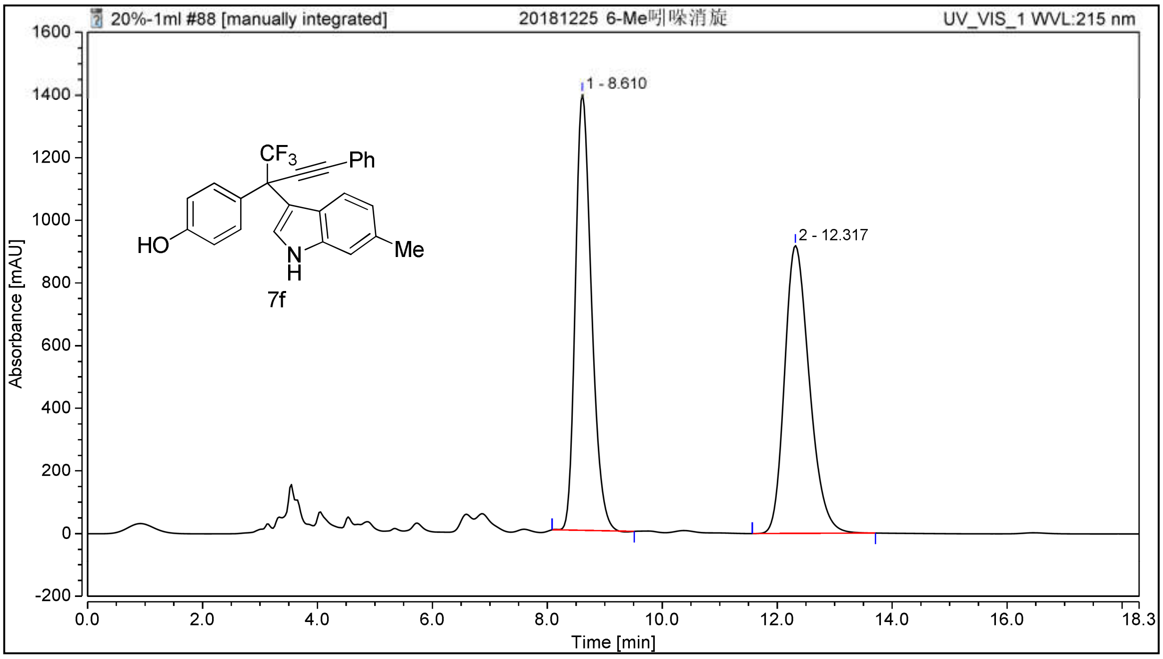

Integration Results

No.	Peak Name	Retention Time<br>min	Area<br>mAU*min	Relative Area<br>%	Amount<br>n.a.
1		8.610	451.942	49.59	n.a.
2		12.317	459.405	50.41	n.a.
**Total:**			**911.347**	**100.00**	

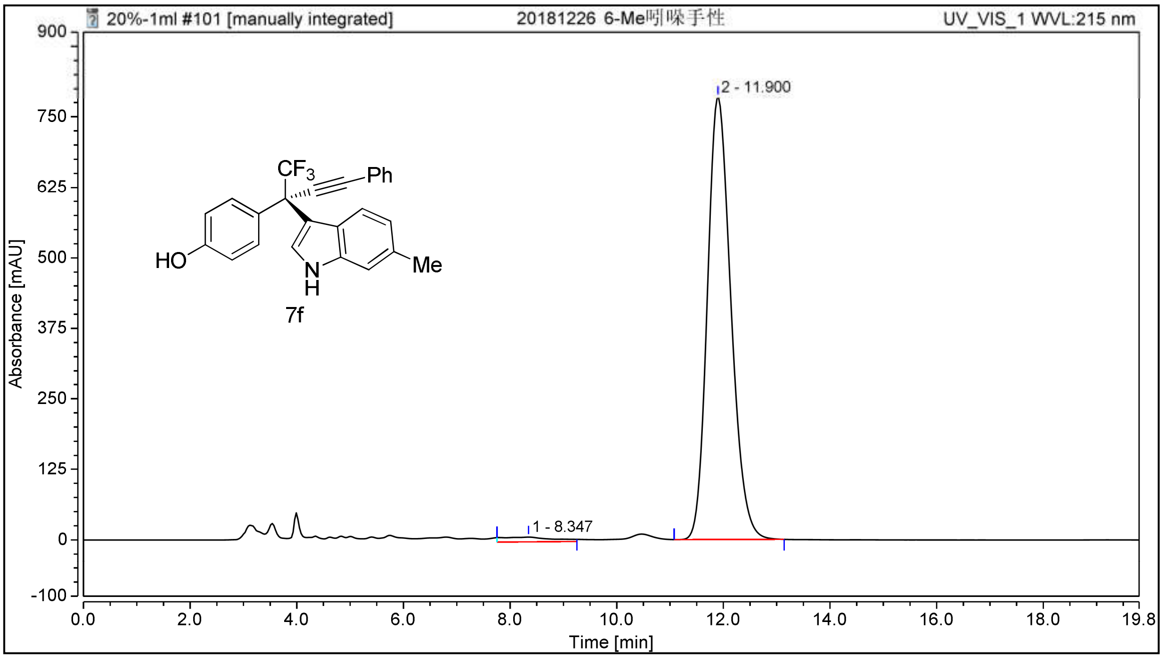

Integration Results

No.	Peak Name	Retention Time<br>min	Area<br>mAU*min	Relative Area<br>%	Amount<br>n.a.
1		8.347	8.559	2.09	n.a.
2		11.900	400.564	97.91	n.a.
**Total:**			**409.123**	**100.00**	

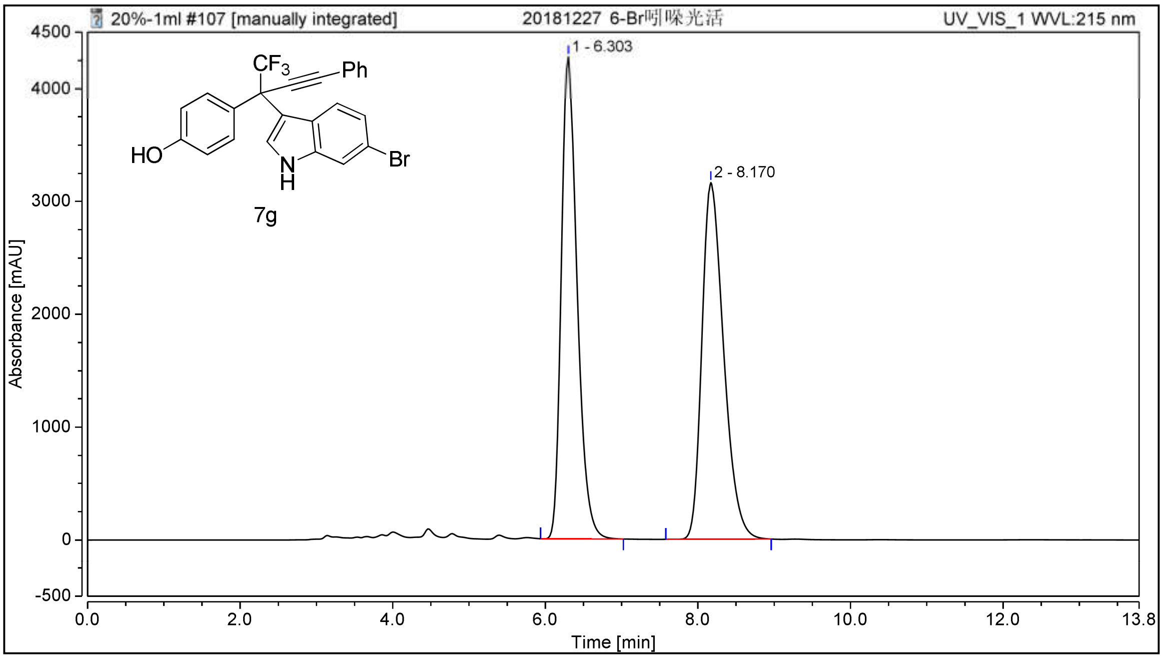

Integration Results

No.	Peak Name	Retention Time<br>min	Area<br>mAU*min	Relative Area<br>%	Amount<br>n.a.
1		6.303	1027.966	49.62	n.a.
2		8.170	1043.722	50.38	n.a.
**Total:**			**2071.687**	**100.00**	

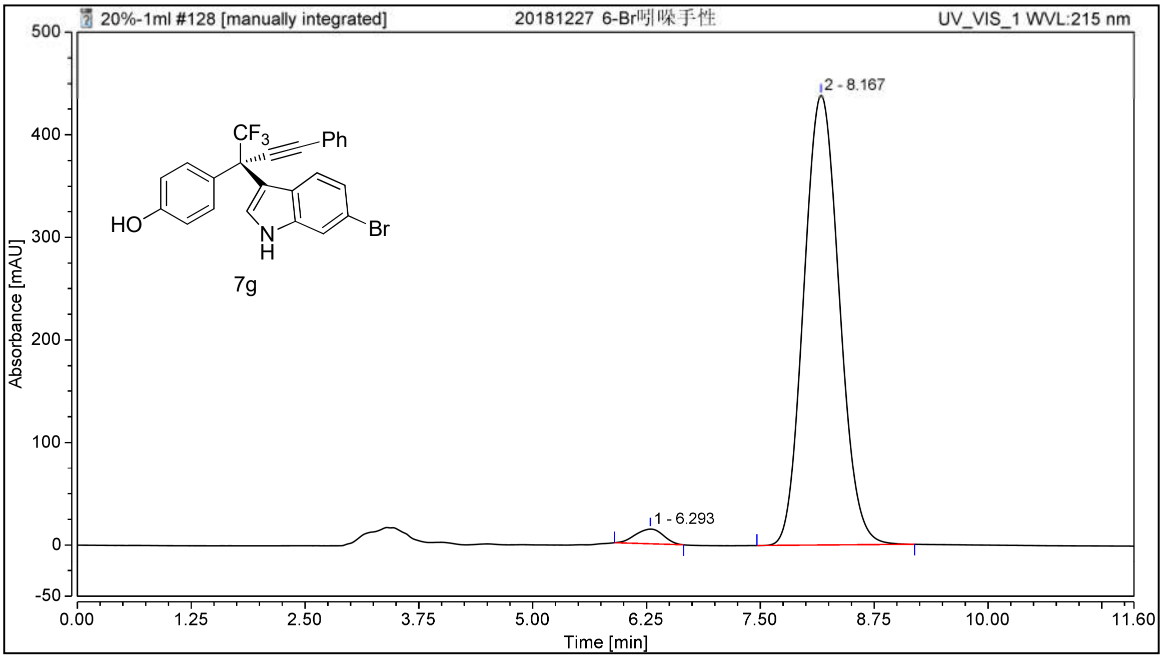

Integration Results

No.	Peak Name	Retention Time<br>min	Area<br>mAU*min	Relative Area<br>%	Amount<br>n.a.
1		6.293	5.082	2.50	n.a.
2		8.167	197.806	97.50	n.a.
**Total:**			**202.888**	**100.00**	

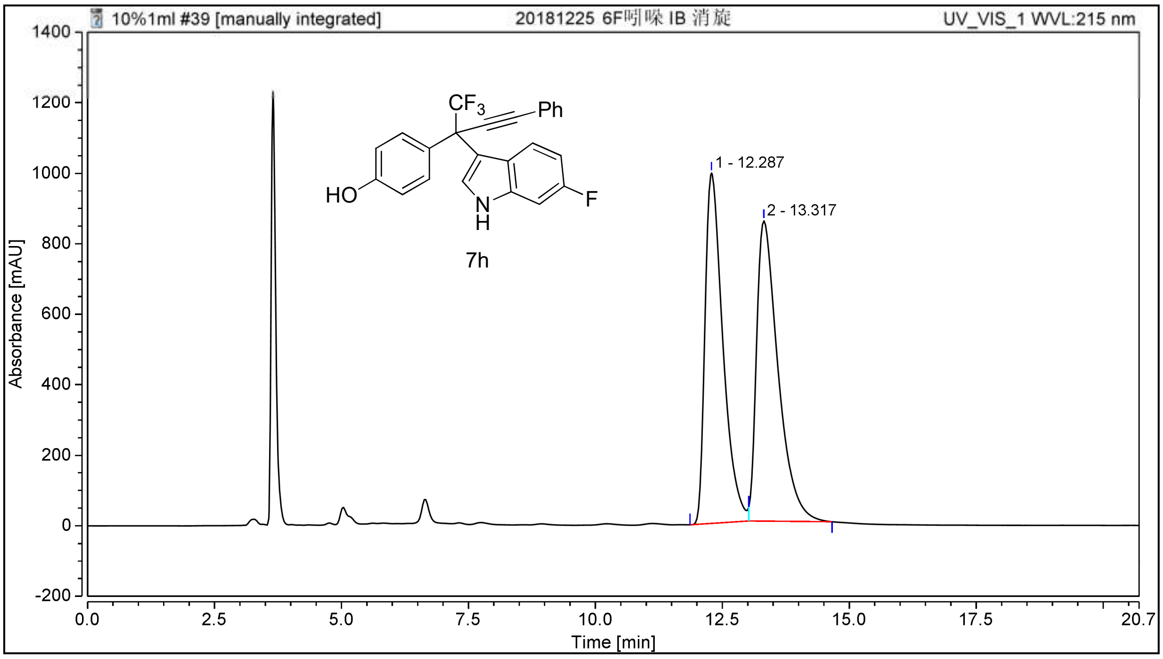

Integration Results					
No.	Peak Name	Retention Time<br>min	Area<br>mAU*min	Relative Area<br>%	Amount<br>n.a.
1		12.287	405.362	49.26	n.a.
2		13.317	417.541	50.74	n.a.
**Total:**			**822.903**	**100.00**	

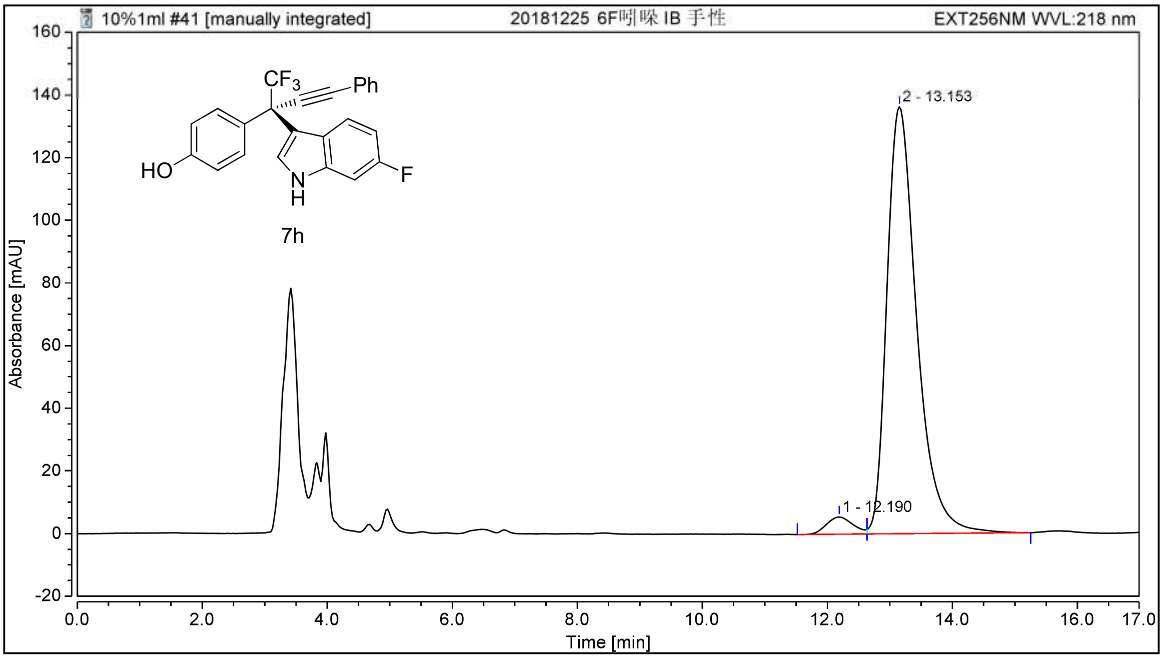

Integration Results					
No.	Peak Name	Retention Time<br>min	Area<br>mAU*min	Relative Area<br>%	Amount<br>n.a.
1		12.190	2.681	3.45	n.a.
2		13.153	75.079	96.55	n.a.
**Total:**			**77.760**	**100.00**	

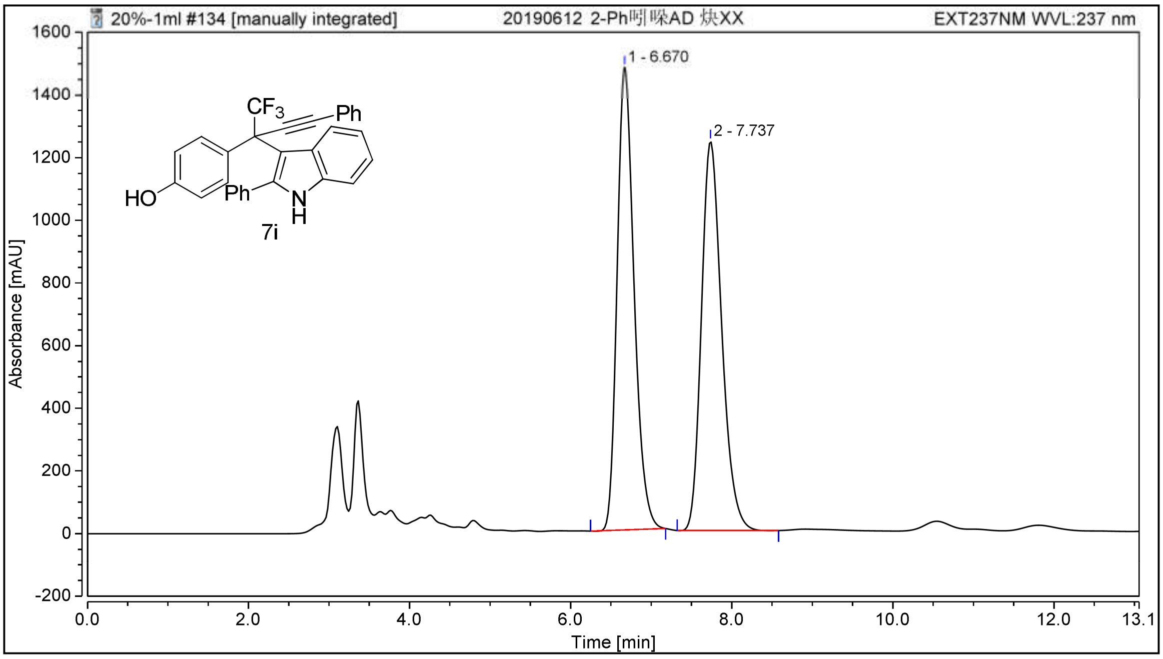

Integration Results

No.	Peak Name	Retention Time<br>min	Area<br>mAU*min	Relative Area<br>%	Amount<br>n.a.
1		6.670	359.581	49.84	n.a.
2		7.737	361.928	50.16	n.a.
**Total:**			**721.509**	**100.00**	

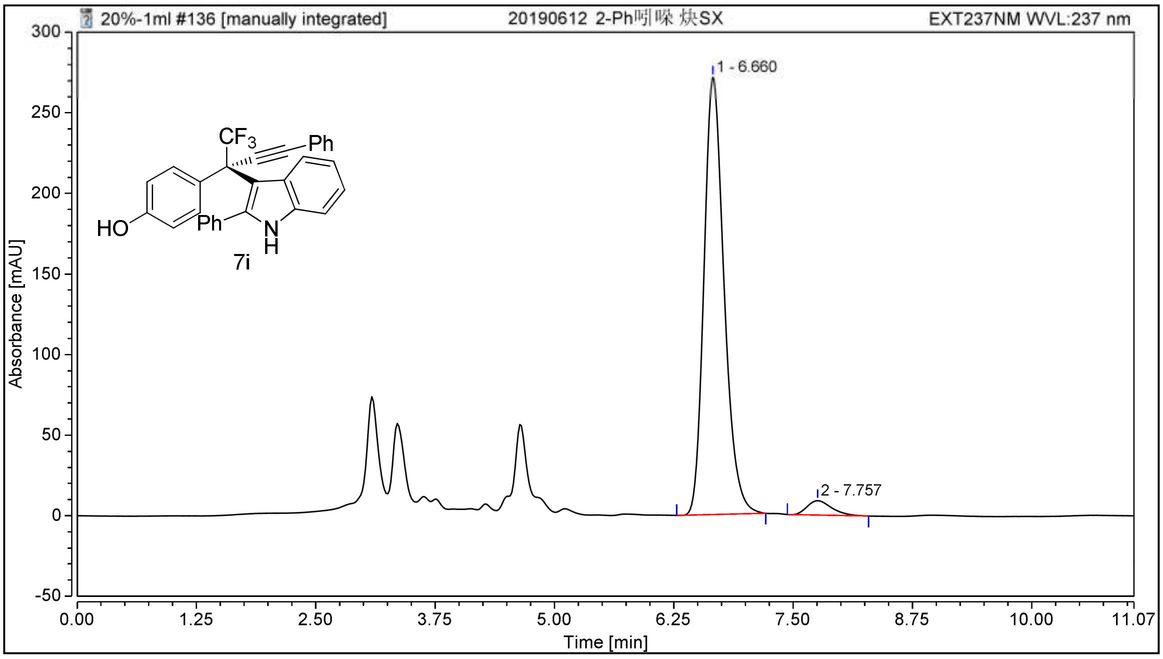

Integration Results

No.	Peak Name	Retention Time<br>min	Area<br>mAU*min	Relative Area<br>%	Amount<br>n.a.
1		6.660	66.229	96.16	n.a.
2		7.757	2.644	3.84	n.a.
**Total:**			**68.872**	**100.00**	

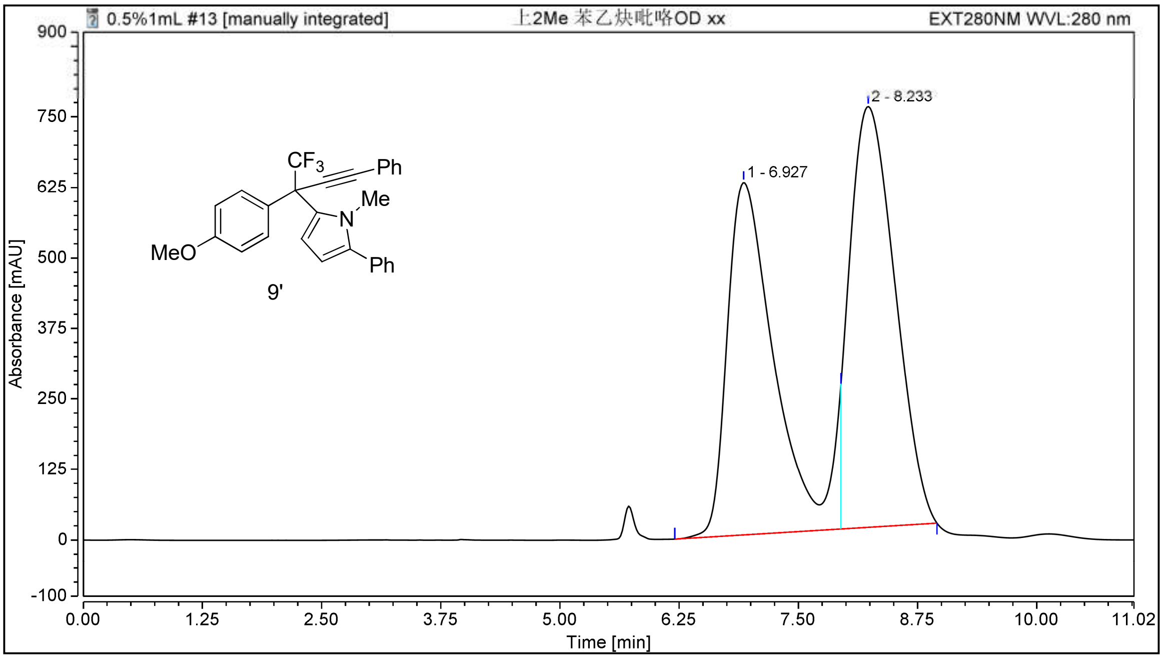

Integration Results

No.	Peak Name	Retention Time<br>min	Area<br>mAU*min	Relative Area<br>%	Amount<br>n.a.
1		6.927	366.917	47.48	n.a.
2		8.233	405.908	52.52	n.a.
**Total:**			**772.825**	**100.00**	

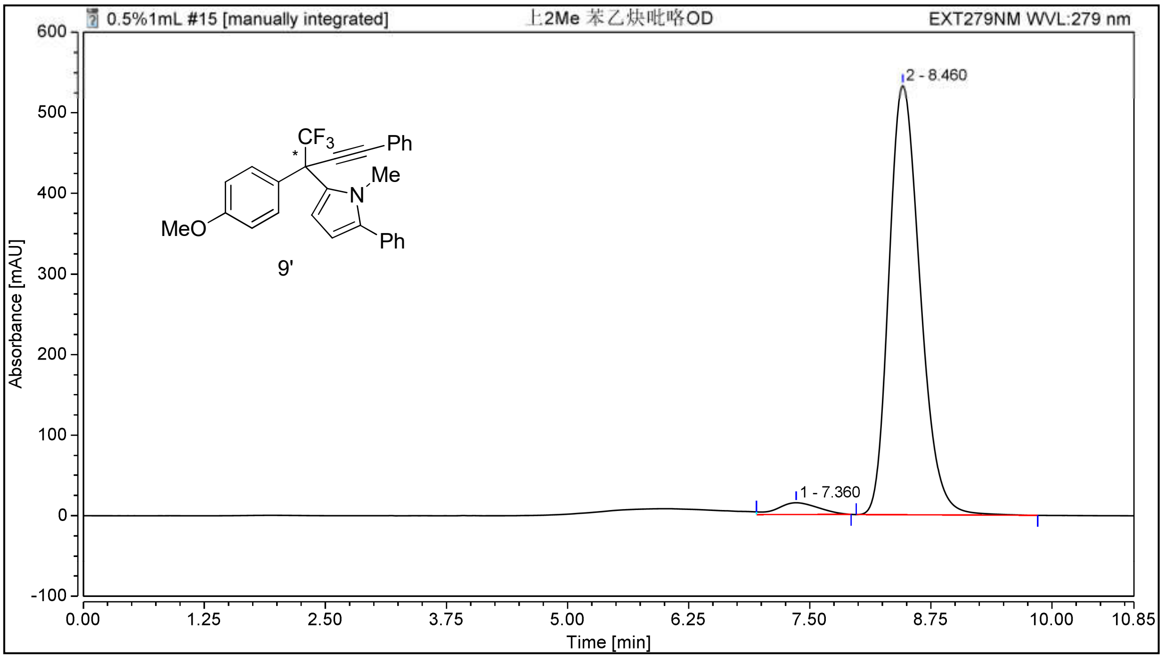

Integration Results

No.	Peak Name	Retention Time<br>min	Area<br>mAU*min	Relative Area<br>%	Amount<br>n.a.
1		7.360	7.121	3.46	n.a.
2		8.460	198.494	96.54	n.a.
**Total:**			**205.616**	**100.00**	

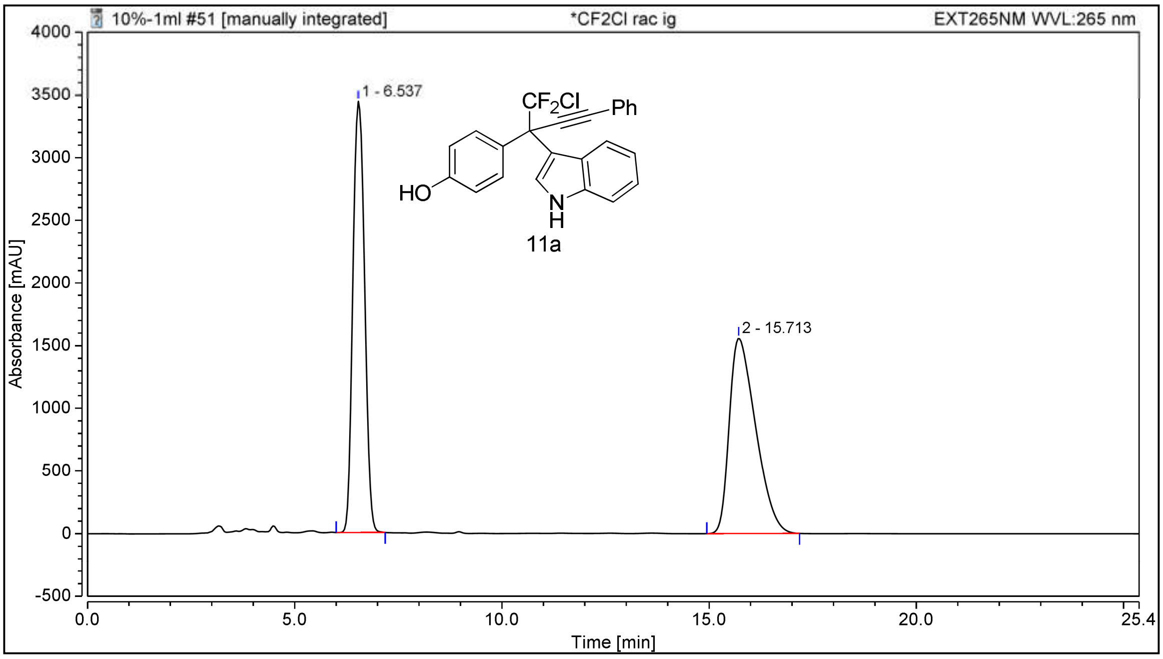

Integration Results

No.	Peak Name	Retention Time	Area	Relative Area	Amount
		min	mAU*min	%	n.a.
1		6.537	1128.852	49.58	n.a.
2		15.713	1148.016	50.42	n.a.
**Total:**			**2276.868**	**100.00**	

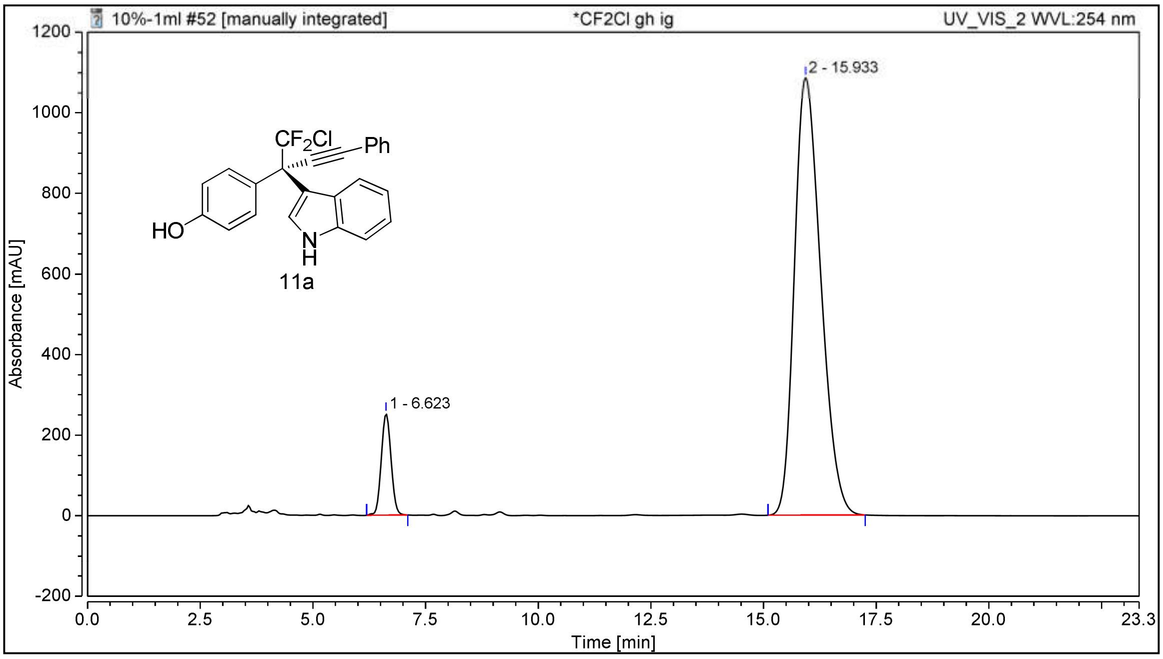

Integration Results

No.	Peak Name	Retention Time	Area	Relative Area	Amount
		min	mAU*min	%	n.a.
1		6.623	63.351	7.93	n.a.
2		15.933	735.133	92.07	n.a.
**Total:**			**798.484**	**100.00**	

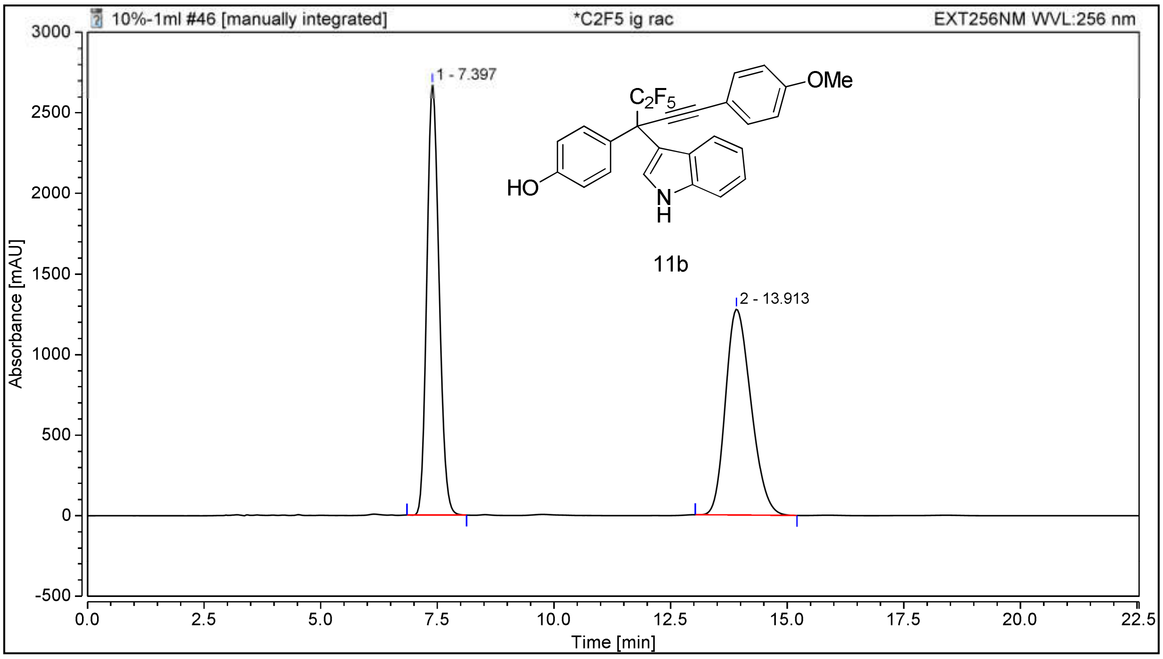

Integration Results

No.	Peak Name	Retention Time<br>min	Area<br>mAU*min	Relative Area<br>%	Amount<br>n.a.
1		7.397	780.637	49.99	n.a.
2		13.913	780.893	50.01	n.a.
**Total:**			**1561.530**	**100.00**	

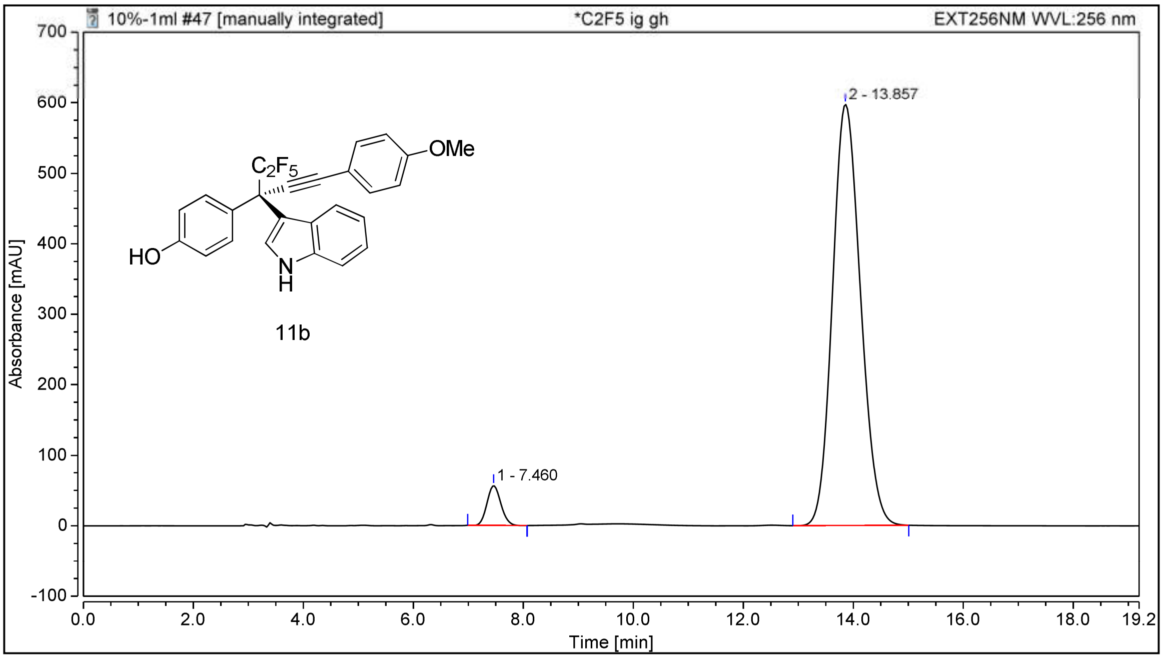

Integration Results

No.	Peak Name	Retention Time<br>min	Area<br>mAU*min	Relative Area<br>%	Amount<br>n.a.
1		7.460	16.358	4.44	n.a.
2		13.857	352.246	95.56	n.a.
**Total:**			**368.604**	**100.00**	

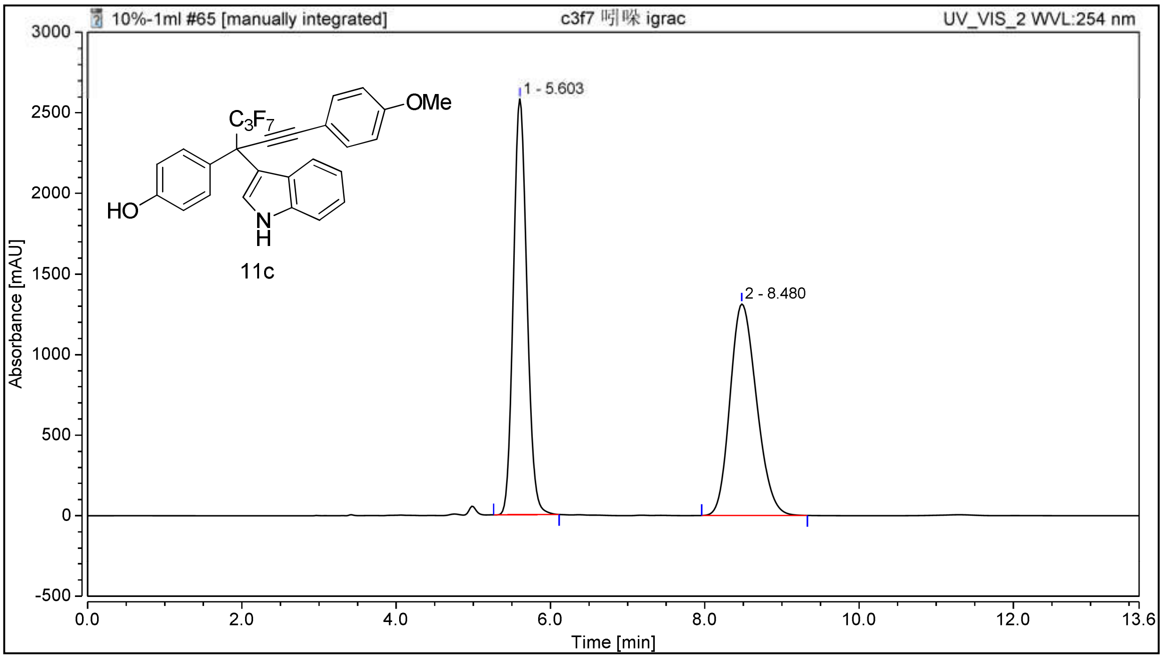

Integration Results

No.	Peak Name	Retention Time<br>min	Area<br>mAU*min	Relative Area<br>%	Amount<br>n.a.
1		5.603	525.001	50.11	n.a.
2		8.480	522.746	49.89	n.a.
**Total:**			**1047.746**	**100.00**	

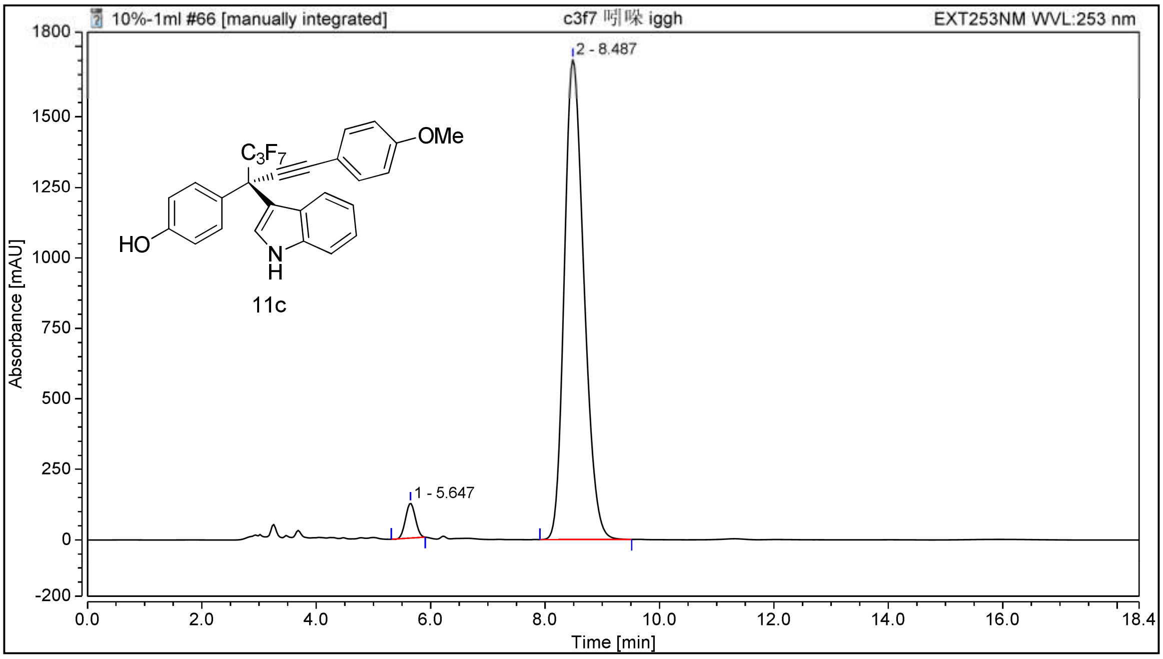

Integration Results

No.	Peak Name	Retention Time<br>min	Area<br>mAU*min	Relative Area<br>%	Amount<br>n.a.
1		5.647	24.501	3.50	n.a.
2		8.487	676.027	96.50	n.a.
**Total:**			**700.528**	**100.00**	

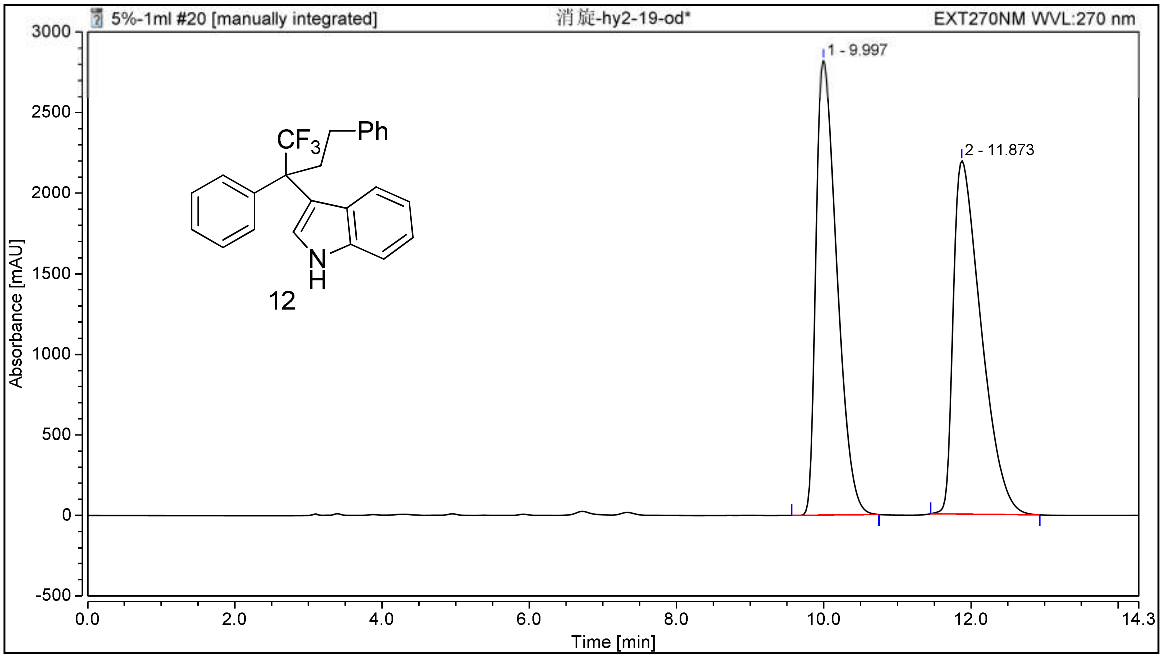

Integration Results

No.	Peak Name	Retention Time<br>min	Area<br>mAU*min	Relative Area<br>%	Amount<br>n.a.
1		9.997	930.889	49.24	n.a.
2		11.873	959.503	50.76	n.a.
**Total:**			**1890.392**	**100.00**	

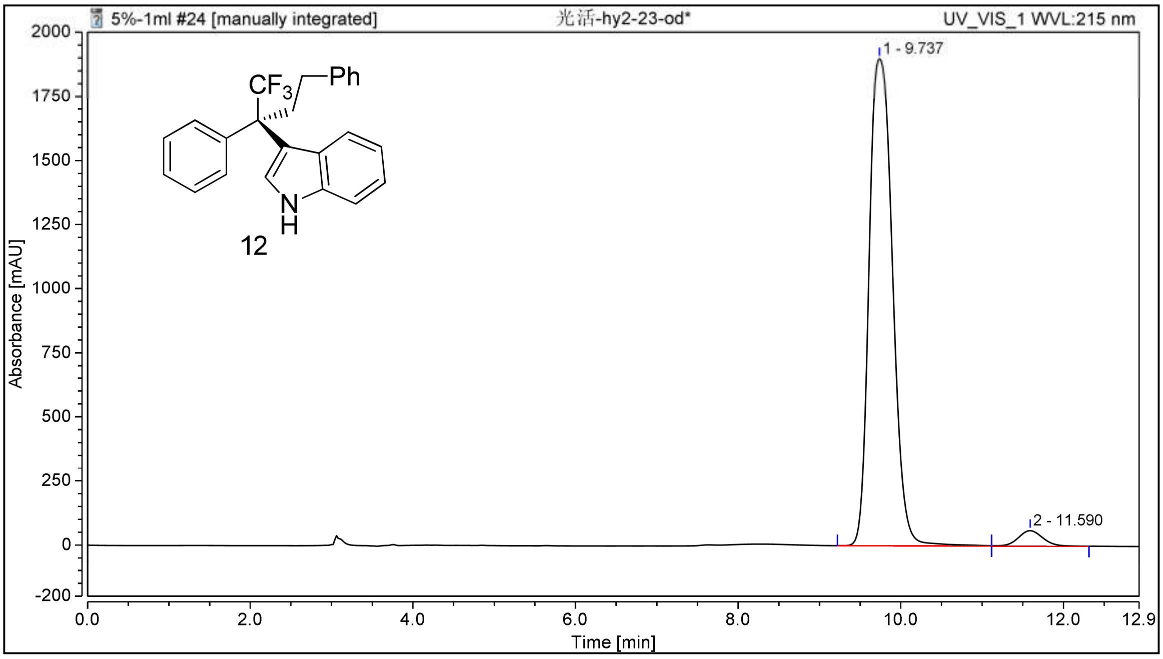

Integration Results

No.	Peak Name	Retention Time<br>min	Area<br>mAU*min	Relative Area<br>%	Amount<br>n.a.
1		9.737	633.937	96.68	n.a.
2		11.590	21.793	3.32	n.a.
**Total:**			**655.730**	**100.00**	

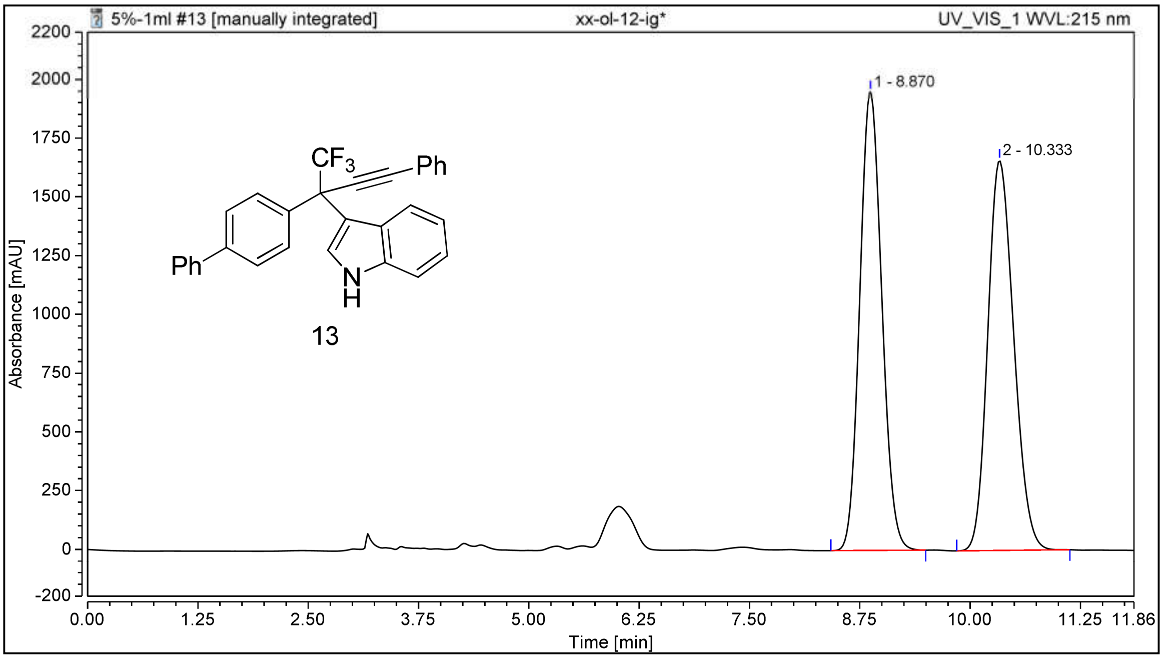

Integration Results					
No.	Peak Name	Retention Time<br>min	Area<br>mAU*min	Relative Area<br>%	Amount<br>n.a.
1		8.870	563.779	49.94	n.a.
2		10.333	565.087	50.06	n.a.
**Total:**			**1128.867**	**100.00**	

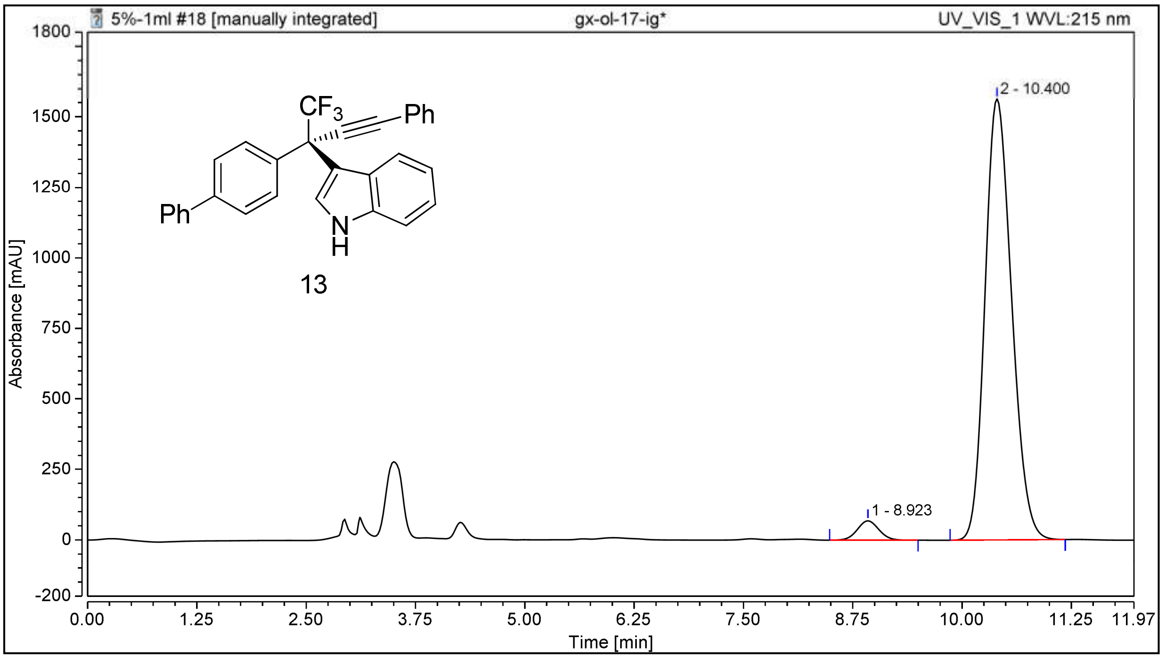

Integration Results					
No.	Peak Name	Retention Time<br>min	Area<br>mAU*min	Relative Area<br>%	Amount<br>n.a.
1		8.923	19.510	3.42	n.a.
2		10.400	551.122	96.58	n.a.
**Total:**			**570.632**	**100.00**	

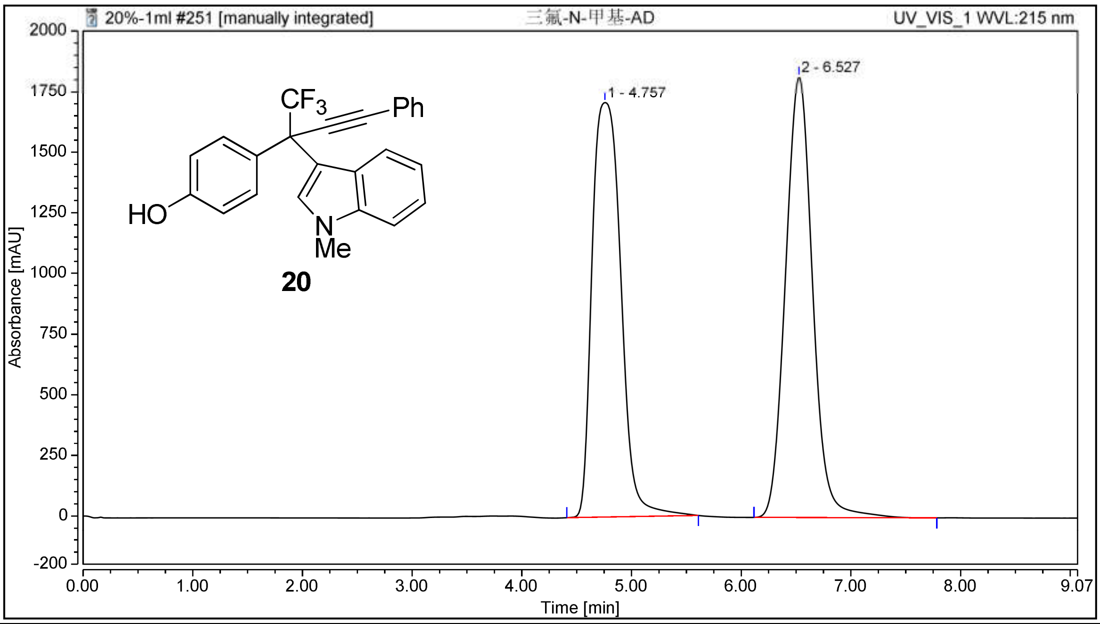

Integration Results

No.	Peak Name	Retention Time<br>min	Area<br>mAU*min	Relative Area<br>%	Amount<br>n.a.
1		4.757	519.650	49.98	n.a.
2		6.527	520.012	50.02	n.a.
**Total:**			**1039.663**	**100.00**	

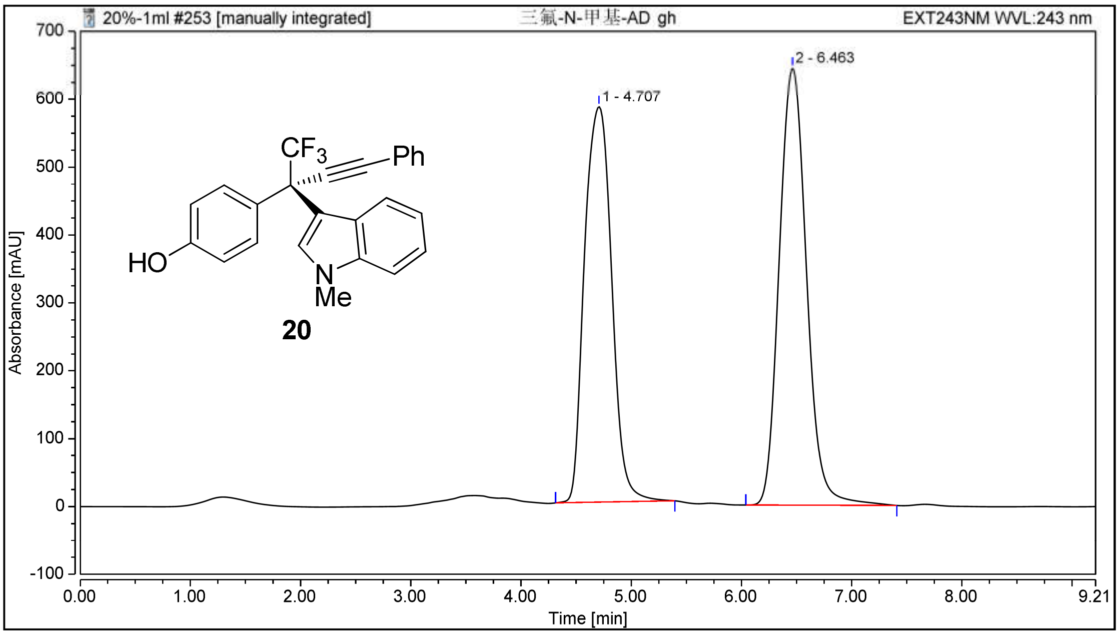

Integration Results

No.	Peak Name	Retention Time<br>min	Area<br>mAU*min	Relative Area<br>%	Amount<br>n.a.
1		4.707	166.529	47.45	n.a.
2		6.463	184.445	52.55	n.a.
**Total:**			**350.974**	**100.00**	
